# Supplementary material for: Late-stage guanine C8–H alkylation of nucleosides, nucleotides, and oligonucleotides via photo-mediated Minisci reaction
Source: Nat Commun. 2024 Mar 21;15:2549. doi: 10.1038/s41467-024-46671-4 (PMC10957873; doi:10.1038/s41467-024-46671-4)
Supplement: Supplementary file 1 — Supplementary information [file 41467_2024_46671_MOESM1_ESM.pdf]

Supplementary Information

Late-Stage Guanine C8–H Alkylation of Nucleosides, Nucleotides, and Oligonucleotides *via* Photo-mediated Minisci Reaction

Ruoqian Xie[1,2, ‡], Wanlu Li[1, ‡], Yuhua Ge[1, *], Yutong Zhou[2,3], Guolan Xiao[2], Qin Zhao[2], Yunxi Han[2], Yangyan Li[2], Gang Chen[2,3,*]

[1]School of Chemistry and Chemical Engineering, Southeast University, Nanjing 211189, People's Republic of China.

[2]Shanghai Key Laboratory for Molecular Engineering of Chiral Drugs, School of Chemistry and Chemical Engineering, Shanghai Jiao Tong University, Shanghai 200240, People's Republic of China.

[3]Key Laboratory of Green and High-End Utilization of Salt Lake Resources, Chinese Academy of Sciences, Qinghai Institute of Salt Lakes, Chinese Academy of Sciences, Xining 810008, Qinghai, People's Republic of China

Contents

1. General information 3
2. Reported examples and our approaches 15
2.1. Selected examples of C8 alkylation of purine 15
2.2. Previous methods of U-C5-alkylation........ 16
2.3. Selected examples of late-stage functionalization of purine of DNA/RNA oligonucleotides 17
2.4. Our methods of G-C8-alkylation and U-C5-alkylation 19
3. List of substrates and preparation methods 20
3.1. Preparation of boron-containing radical precursors 20
3.2. List of nucleic acid substrates 28
4. Reaction screening and proposed mechanism 51
4.1. Standard reaction setup 51
4.2. Optimization of C–H alkylation of guanosine 53
4.3. Optimization of C–H alkylation of uridine 60
4.4. Optimization of C–H alkylation of nucleotide and investigation of the pH value of reaction mixture in various conditions. 61
4.5. Proposed mechanism........ 67
5. Substrate scope study 68
5.1. Substrate scope of C–H alkylation of guanosine with diverse boron radical precursors 68
5.2. Substrate scope of C–H ethylation of guanosine analogues 81
5.3. Substrate scope of C–H alkylation of uridine 89
5.4. Substrate scope of C–H alkylation of complicated nucleotide substrates 94
6. Applications of the methodology regarding nucleosides 104
7. Microscale reactions with nucleoside/tides and oligonucleotide as substrate........ 111
7.1. Reaction setup for nucleosides and dinucleotide 111
7.2. Optimization of microscale reaction conditions 113
8. Substrate scope study for microscale reaction with nucleosides/tides and oligonucleotide as substrate 115
8.1. Substrate scope of C–H alkylation of guanosine and RNA oligonucleotides 117
8.2. Substrate scope of C–H ethylation of ssDNA oligonucleotides 147
8.3. Substrate scope of C–H ethylation of oligonucleotides containing two guanines ... 168
8.4. Substrate scope of C–H ethylation of dsDNA oligonucleotides 173
8.5. Analysis of modification site of alkylated oligonucleotides 178
9. Applications of the methodology regarding oligonucleotides 184
10. NMR spectrum 189
References 295

1. General information

All the chemicals were purchased commercially and used without further purification. General reagents were obtained from Adamas, Leyan, Innochem, Laajoo and Bidepharm. Anhydrous solvents were obtained from Adamas. The lights and their wavelength: 45 W and 85 W white light (~ 437.2-616.2 nm) (high power energy saving lamps), 10 W white LED (450-465 nm), 10 W, 24 W, and 35 W blue LED (450-455 nm). All of the oligonucleotides were custom synthesized by the Suzhou Biosyntech Co., Ltd. Analytical thin layer chromatography (TLC) was carried out on 0.25 mm SiO_2 (silica gel 60 F254, Leyan), and the spots were visualized under ultraviolet light (254 and 365 nm). ^1H and ^{13}C NMR spectra were recorded on Bruker-400 spectrometer (400 MHz for ^1H, 101 MHz for ^{13}C) and Bruker-500 spectrometer (500 MHz for ^1H, 126 MHz for ^{13}C), and were fully decoupled by broad band proton decoupling; DMSO-d_6 and D_2O were used as a solvent. The chemical shifts were reported in ppm downfield (δ) relative to the residual solvent peak (DMSO-d_6: ^1H, 2.50; ^{13}C, 39.52), and (D_2O: ^1H, 4.79). The peak patterns are indicated as follows: s, singlet; d, doublet; t, triplet; q, quartet; m, multiplet; dd, doublet of doublets; dt, doublet of triplet; br, broad. Coupling constants, *J*, were reported in Hertz unit (Hz). Reverse-phase column chromatography was performed on SepaBean® machine T from Santai Technologies in Changzhou, China, using ODS 45- 60 mm C18 Spherical silica. The high-resolution mass spectrometry (HRMS) was recorded on an Agilent Mass spectrometer using Electrospray Ionization Time-of-Flight (ESI-TOF). The MALDI-FTMS use the Bruker 7.0T SolariX Fourier transform ion cyclotron resonance mass spectrometry (Bruker Daltonics, Germany), equipped with Matrix-assisted laser desorption/ionization (MALDI) ion source. This MALDI source has a pulsed smartbeam-II UV laser with an attenuator that allows fine adjustment of laser fluency (355 nm, Azura Laser AG, Berlin, Germany). The LTQ-XL use the Thermo Scientific LTQ XL™ Linear Ion Trap Mass Spectrometer of Suzhou Biosyntech Co., Ltd. Optical rotations were measured on an Anton Paar MCP100 automatic polarimeter using a 100 mm path-length cell at 589 nm. Melting points were measured with microscope WRX-4 (Shanghai Yice). The instruments used for photoreaction of dinucleotide are multi-channel parallel photocatalytic reaction system (ROGER) and low temperature coolant circulation pump (BiLon).

Analytical LC-MS methods

For nucleosides

Analytical LC-MS was performed on Eclipse Plus C18 Column (3.5 um, 4.6 mm × 100 mm); UV detection was performed at 254 nm.

Analytical method: Flow rate: 0.4 mL/min; Temperature of column temperature oven: 40 °C. Elution was done with a gradient starting with 1 % B and staying for 3 minutes. Then, rising to 50 % B within 4 minutes. Rising to 90 % B within 2 minute and staying for 2 minutes. Followed by reducing to 1 % B within 2 minutes and maintaining for 2 minutes. Solvent A = water + 0.1 % formic acid, and B = acetonitrile.

For ***microscale reactions*** of nucleosides/tides and oligonucleotides [1]

Analytical LC-MS was performed on XBridge Premier Peptide BEH C18 Column (300Å, 2.5 um, 4.6 mm × 100 mm); UV detection was performed at 260 nm. The mass data of oligonucleotides is often observed in the form of $[M-2H]^{2-}$ in the negative ion mode.

For ethylation of nucleosides and dinucleotides

Analytical method A: Flow rate: 0.4 mL/min; Temperature of column temperature oven: 40 °C. Elution was done with a gradient starting with 0 % B and staying for 3 minutes. Then, rising to 30 % B within 4 minutes and keeping for 4.5 minutes. Followed by reducing to 0 % B within 2 minutes and maintaining for 1.5 minutes. Solvent A = water + 0.1 % formic acid, and B = acetonitrile.

For ethylation of oligonucleotides

Analytical method B: Flow rate: 0.8 mL/min; Temperature of column temperature oven: 60 °C. Elution was done with a gradient starting with 0 % B and going to 13.75 % A in 11 minutes. Then, rising to 30% B within 0.5 minutes and keeping for 1.5 minutes. Followed by reducing to

0 % B within 0.5 minutes and maintaining for 1.5 minutes. Solvent A = 0.1 M TEAA buffer (pH = 7.0), and B = acetonitrile.

For secondary and tertiary carbon alkylation of oligonucleotides

Analytical method C: Flow rate: 0.8 mL/min; Temperature of column temperature oven: 60 °C. Elution was done with a gradient starting with 0 % B and going to 25 % A in 12 minutes. Then, rising to 50 % B within 1 minute and keeping for 1 minute. Followed by reducing to 0 % B within 1 minute and maintaining for 1 minute. Solvent A = 0.1 M TEAA buffer (pH = 7.0), and B = acetonitrile.

Purification method:

For the nucleosides

The compound was purified *via* SepaBean® machine (Santai Technology Inc., China) equipped with the C18-bonded SepaFlash™ columns.

The type of column: SepaFlash™ Bonded Series, **SW012**, C18, 40-60 µm. 120 Å, max operating pressure 400 psi (27.5 bar), showed as **Supplementary Figure 4.**

Eluent: A: H_2O, B: MeOH, gradient elution;

For the dinucleotides and cyclic dinucleotides.

The compound was purified *via* SepaBean® machine (Santai Technology Inc., China) equipped with the C18-bonded SepaFlash™ columns.

The type of column: SepaFlash™ Bonded Series, **SW025**, C18, 20-45 µm. 100 Å, max operating pressure 400 psi (27.5 bar), showed as **Supplementary Figure 5**. Compared to the columns of ***SW012***, they have the same diameter, but this column is longer, which is conducive to separation.

Eluent: 0.1 M TEAA buffer (pH = 7.0), B: MeCN, gradient elution;

For gram-scale nucleosides and nucleotides.

The compound was purified *via* SepaBean® machine (Santai Technology Inc., China) equipped with the C18-bonded SepaFlash™ columns.

The type of column: SepaFlash™ Bonded Series, ***SW120***, C18, 20-45 µm. 100 Å, max operating pressure 400 psi (27.5 bar), showed as **Supplementary Figure 6**.

Eluent: A: H_2O, B: MeOH, gradient elution;

For oligonucleotides

The type of column: Bridge Premier Peptide BEH C18 Column, 2.5 um, 4.6 mm × 100 mm, 300Å.

Machine: HPLC-20AT

Flow rate: 0.8 mL/min.

Temperature of column temperature oven: 60 °C.

Eluent: 0.1 M TEAA buffer (pH = 7.0), B: MeCN.

Gradients of the elution:

Time	Concentration of B (%)
0	0
1.0	0
3.0	10
6.0	15
6.2	50
7.5	50
7.9	0
9.0	0

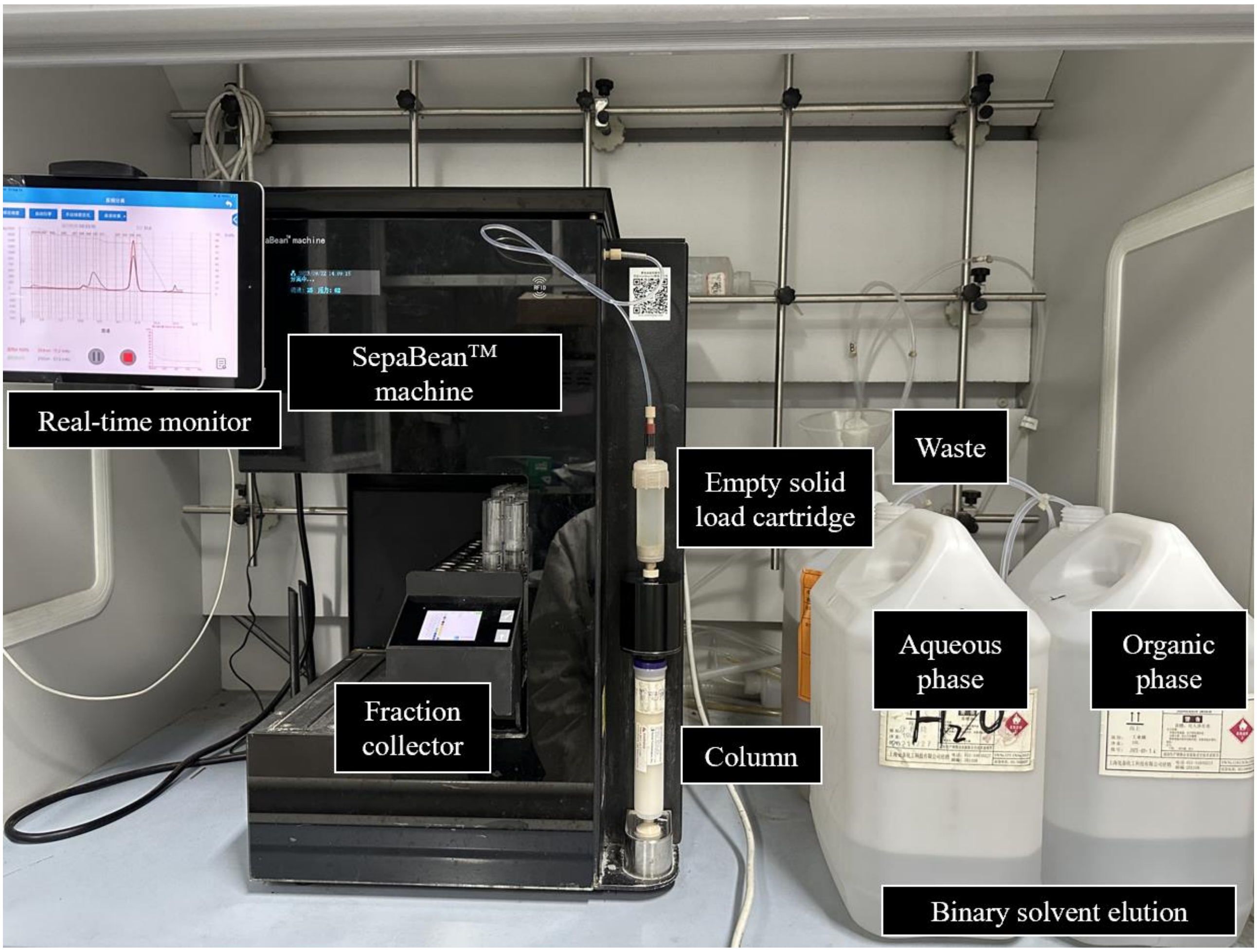

Supplementary Figure 1. SepaBean^{TM} machine flash chromatography system for separating milligram scale reactions.

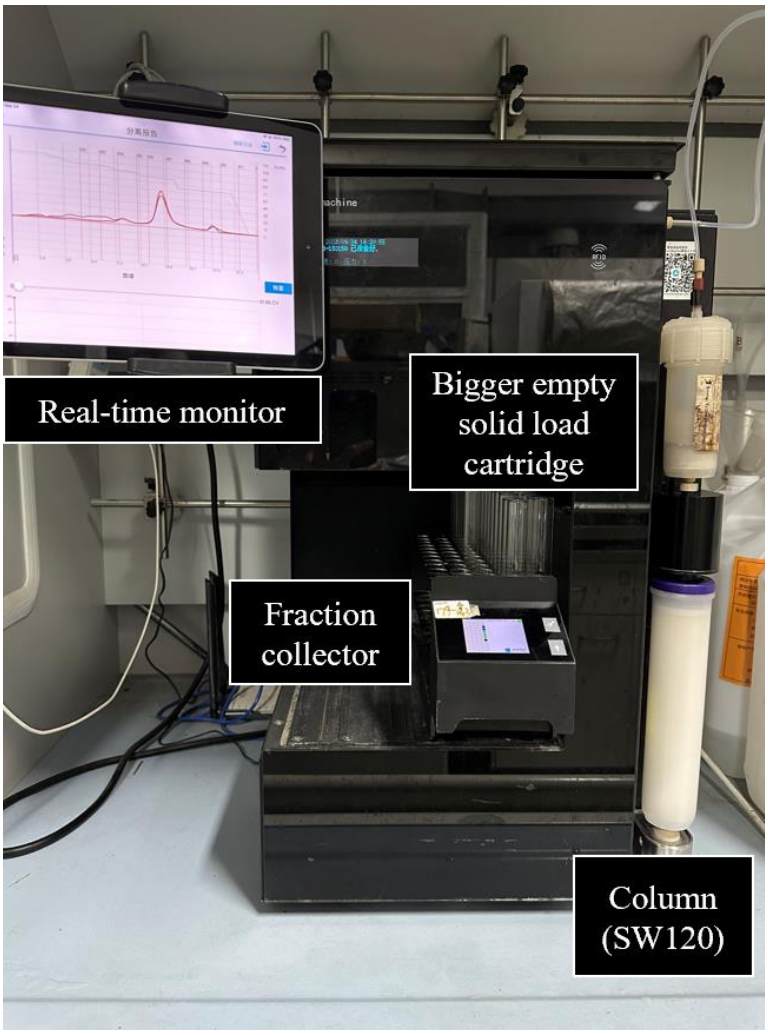

Supplementary Figure 2. SepaBean™ machine flash chromatography system for separating gram scale reactions.

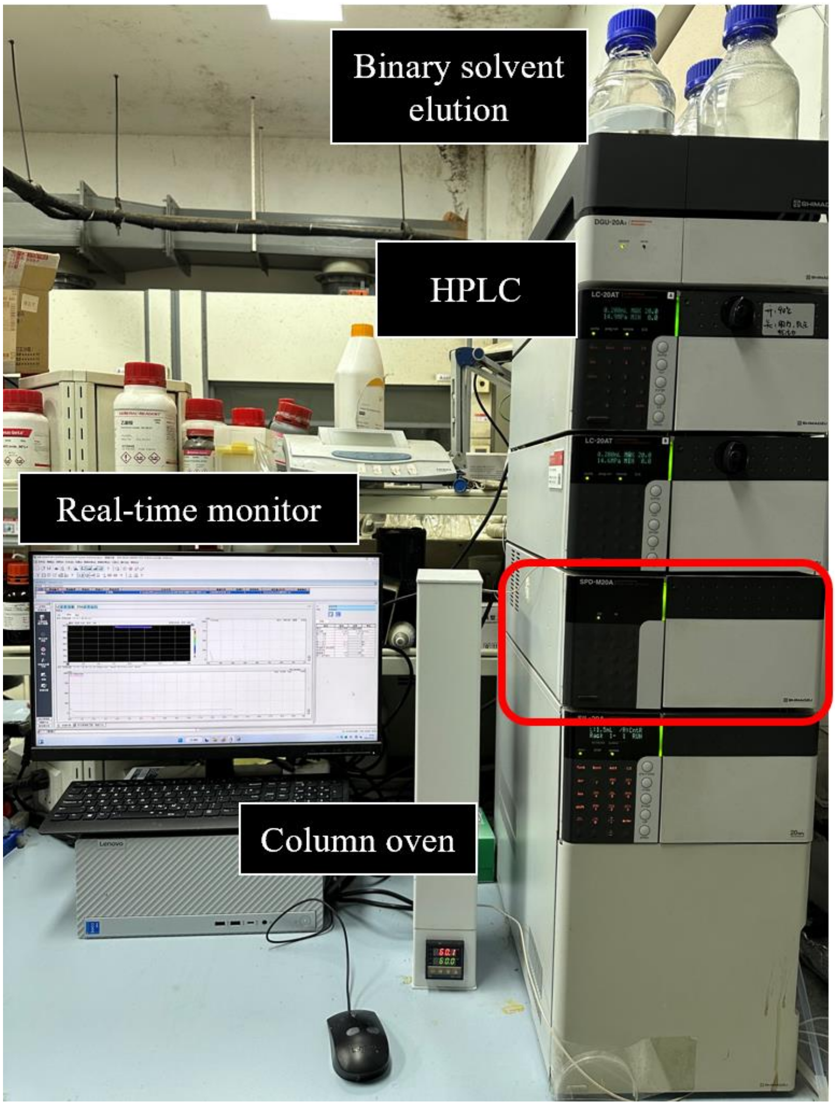
Binary solvent elution
HPLC
Real-time monitor
Column oven

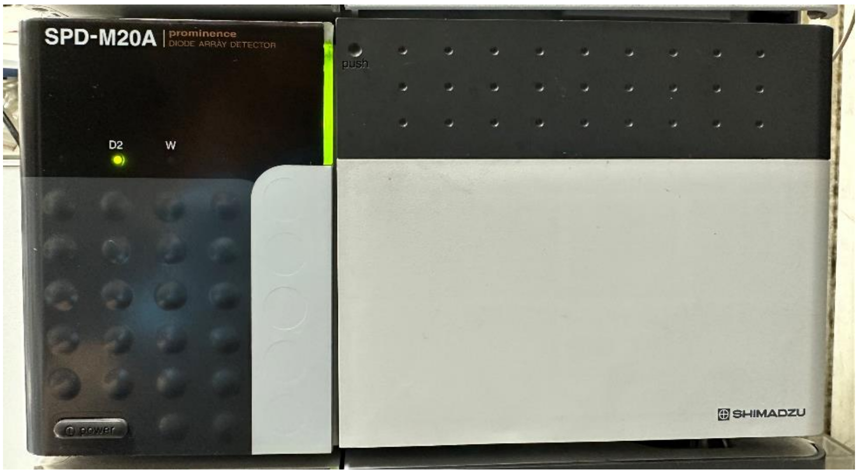
SPD-M20A
prominence
DIODE ARRAY DETECTOR
D2
W
push
SHIMADZU

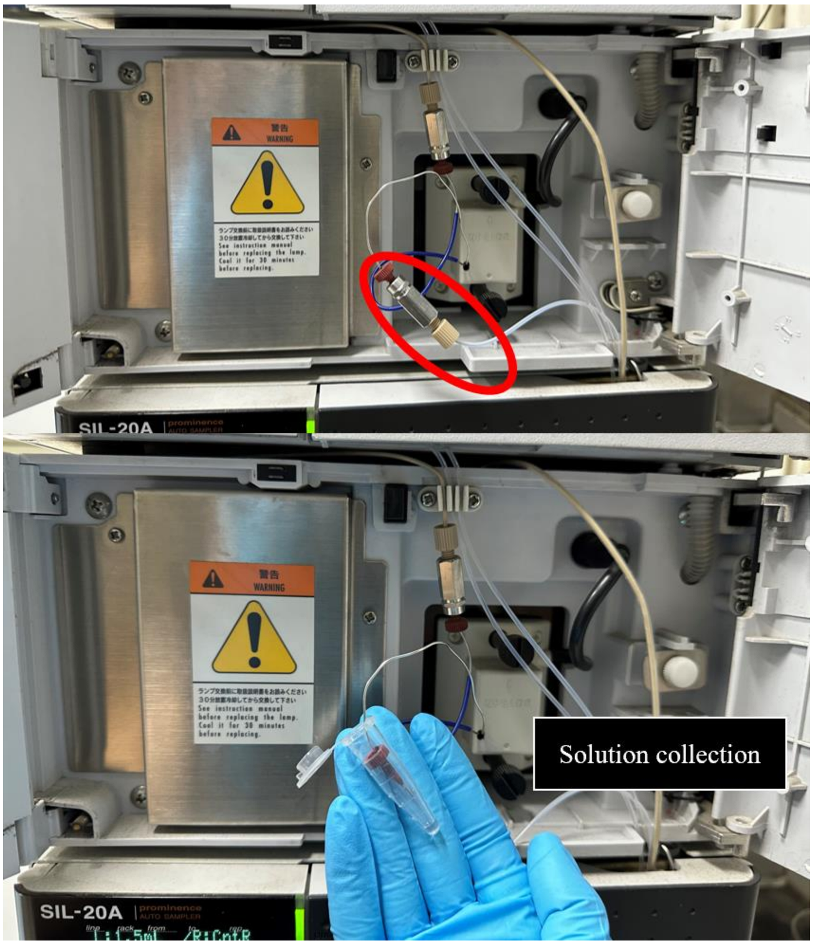

Supplementary Figure 3. Shimadzu™ HPLC for separating microscale oligonucleotides.

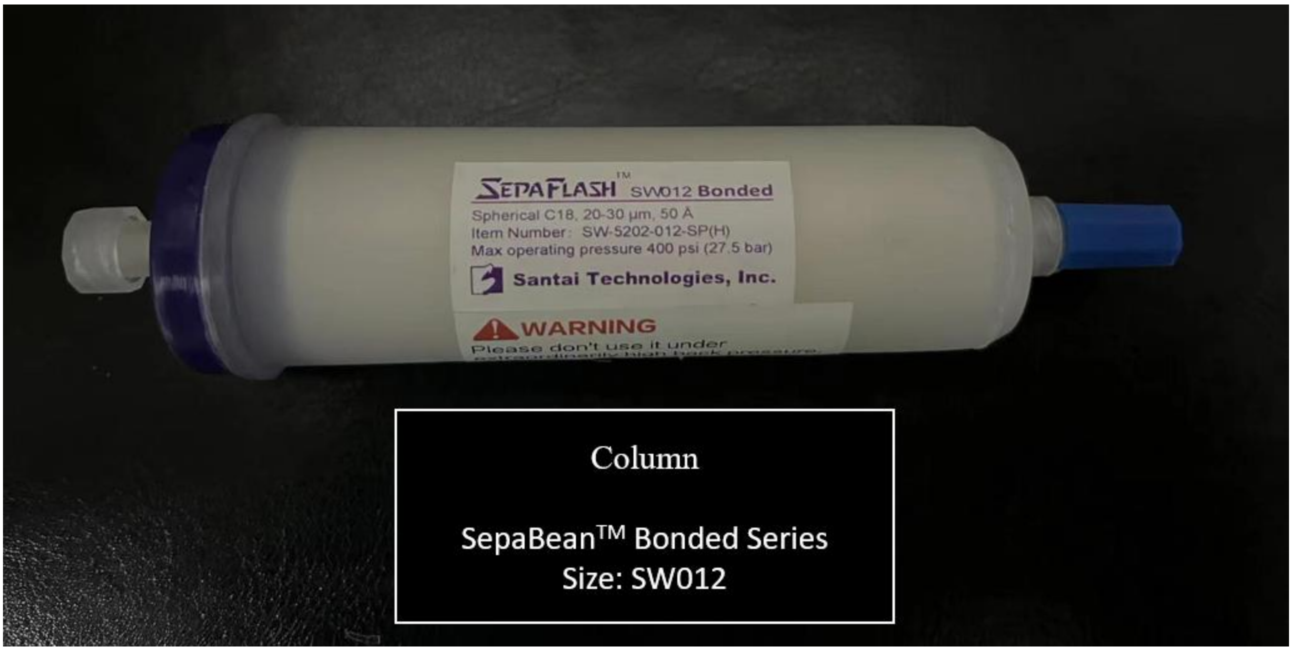

Supplementary Figure 4. SepaFlash™ Bonded Series, column for separating nucleosides (0.1 and 0.2 mmol scale).

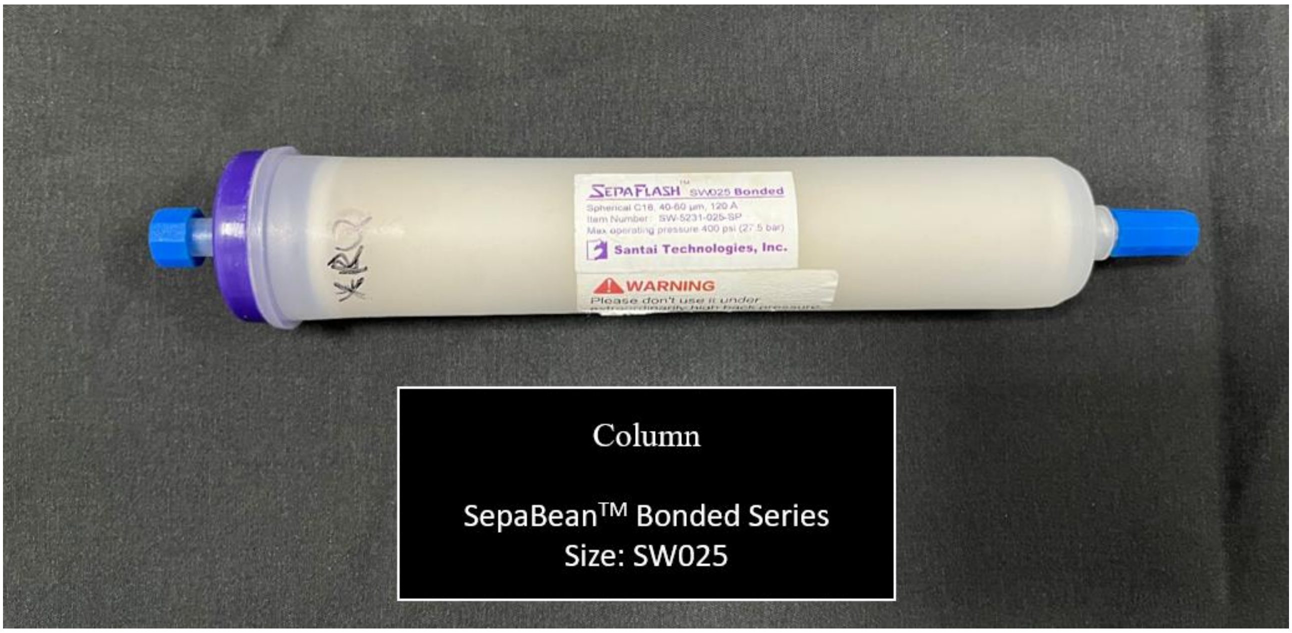

Supplementary Figure 5. SepaFlash™ Bonded Series, column for separating dinucleotides (0.06 mmol scale) and cyclic dinucleotides (0.04 mmol scale).

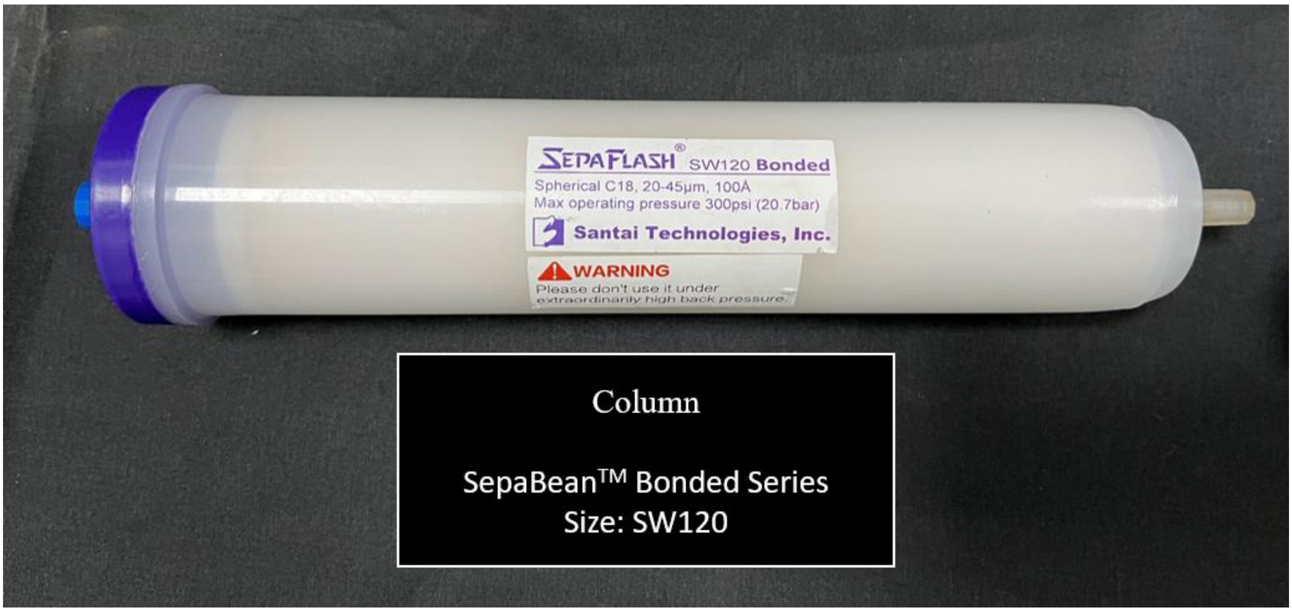

Supplementary Figure 6. SepaFlash™ Bonded Series, column for separating nucleosides and dinucleotides (gram scale).

Supplementary Table 1. Abbreviation table

Abbreviation	Definition
DNA	Deoxyribonucleic Acid
RNA	Ribonucleic acid
ON	oligonucleotide
TEAA	Triethylammonium acetate
TMEDA	Tetramethylethylenediamine
DME	1,2-dimethoxy-ethan
DMF	*N*, *N*-Dimethylformamide
DCM	Dichloromethane
DIC	*N*, *N′*-Diisopropylcarbodiimide
DMSO	Dimethyl sulfoxide
TLC	Thin layer chromatography
LC-MS	Liquid chromatography–mass spectrometry
HRMS	High-resolution mass spectrometry
ESI	Electrospray ionization
MALDI-FTMS	Matrix-assisted laser desorption ionization-Fourier transform mass spectrometry
LTQ-XL	Linear Ion Trap Mass Spectrometer
MS	Mass spectrometry

Supplementary Table 2. Oligonucleotide sequences used

Oligonucleotides	Sequence
**ON 1**	5'-GUUUCC-3'
**ON 2**	5'-UUUGCC-3'
**ON 3**	5'-UUUCCG-3'
**ON 4**	5'-GUUACC-3'
**ON 5**	5'-AUUGCC-3'
**ON 6**	5'-GCUAUCU-3'
**ON 7**	5'-GUUACCUU-3'
**ON 8**	5'-GUUACCUCU-3'
**ON 9**	5’-dGTTCC-3’
**ON 10**	5’-dTTCCG-3’
**ON 11**	5’-dCGTT-3’
**ON 12**	5’-dTTGCC-3’
**ON 13**	5’-dCATGT-3’
**ON 14**	5’-dCCCGTTT-3’
**ON 15**	5’-dCACGTTT-3’
**ON 16**	5’-dCCTTGTTCC-3'
**ON 17**	5'-dCACTTGTTC-3'
**ON 18**	5’-dTGTCGC-3’
**ON 19**	5'-CGAUGU-3'
**ON 20**	5'-d(GAT CTA TTA CGC T)-3'<br>3'-d(CTA GAT AAT GCG A)-5'
**ON 20-1**	5'-d(GAT CTA TTA CGC T)-3'
**ON 20-2**	3'-d(CTA GAT AAT GCG A)-5'

2. Reported examples and our approaches

2.1. Selected examples of C8 alkylation of purine

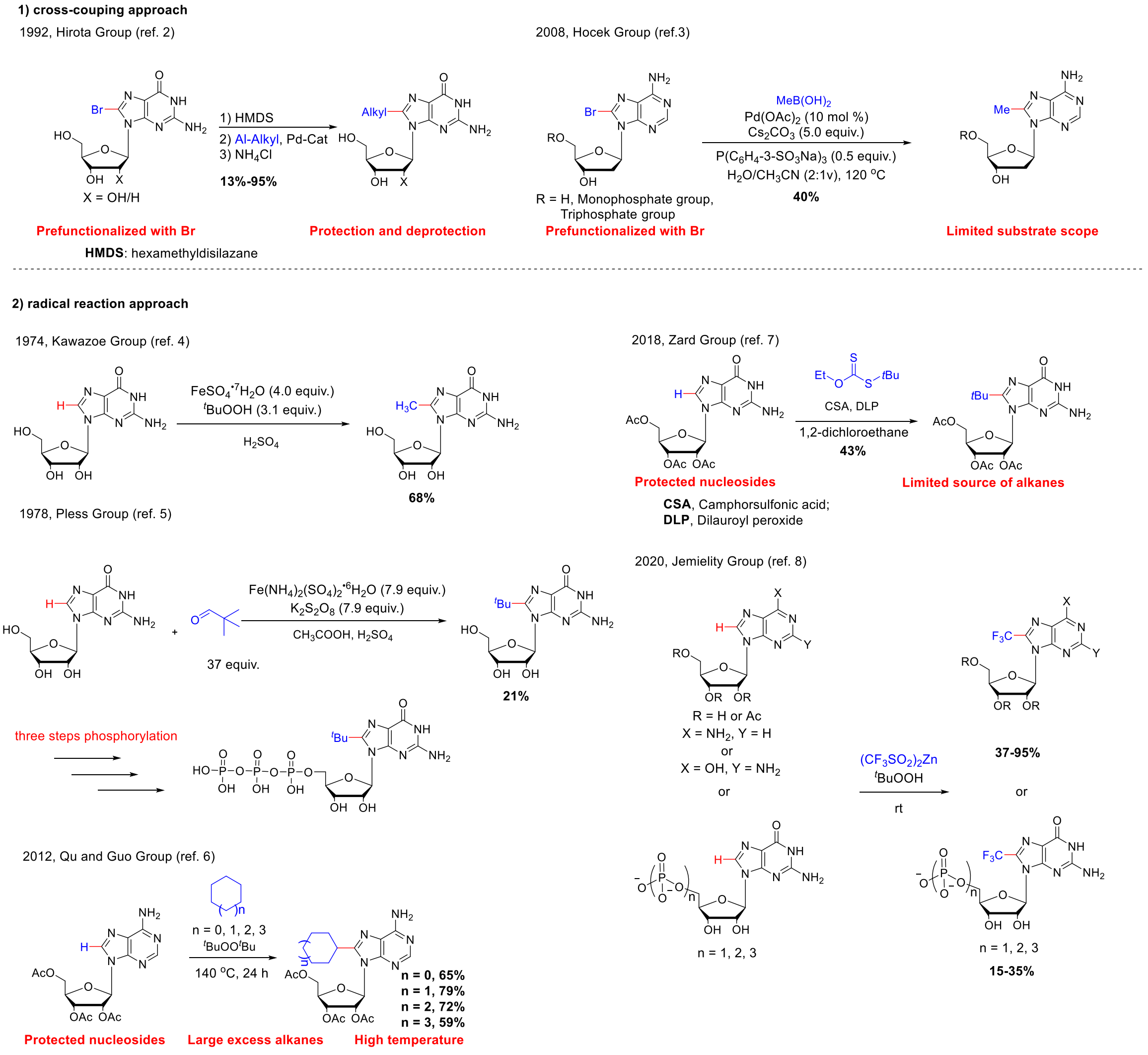

Supplementary Figure 7. Advances in purine nucleoside alkylation *via* Pd-mediated coupling reaction and free radical chemistry [2-8]

2.2. Previous methods of U-C5-alkylation

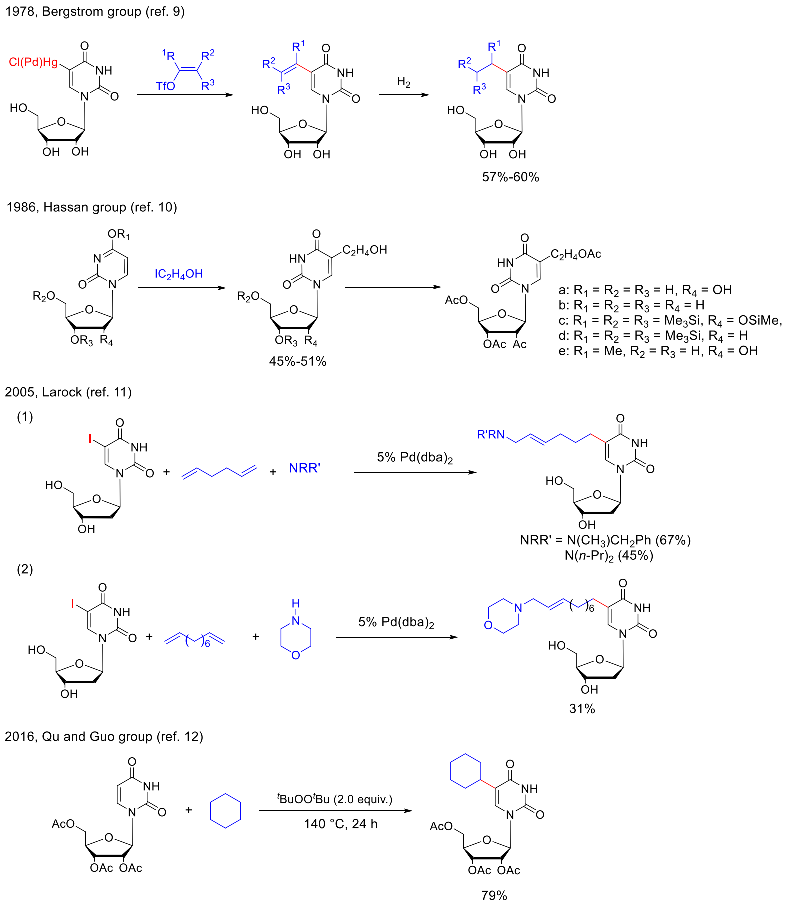

Supplementary Figure 8. Advances in pyrimidine nucleoside alkylation [9-12]

2.3. Selected examples of late-stage functionalization of purine of DNA/RNA oligonucleotide

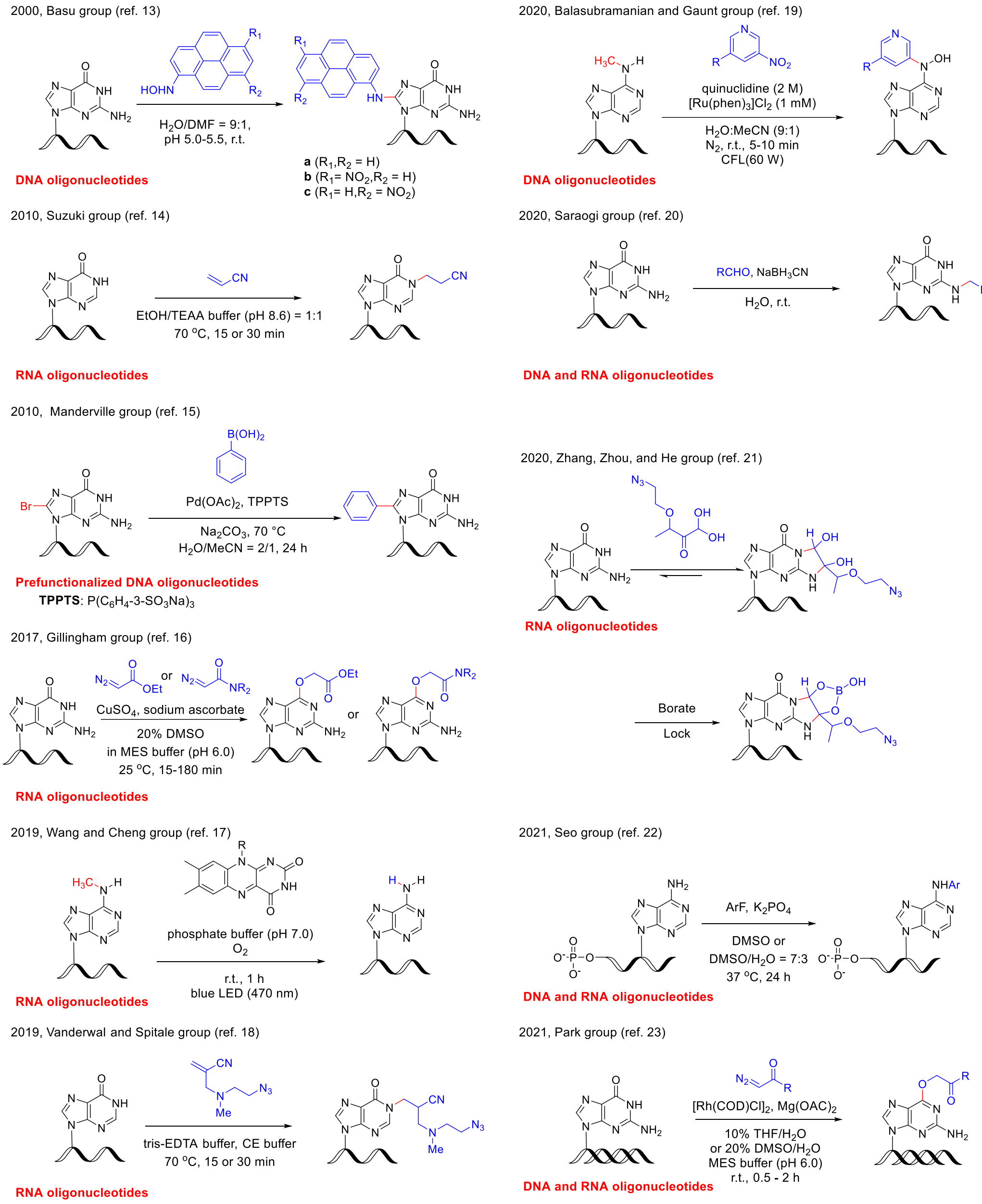

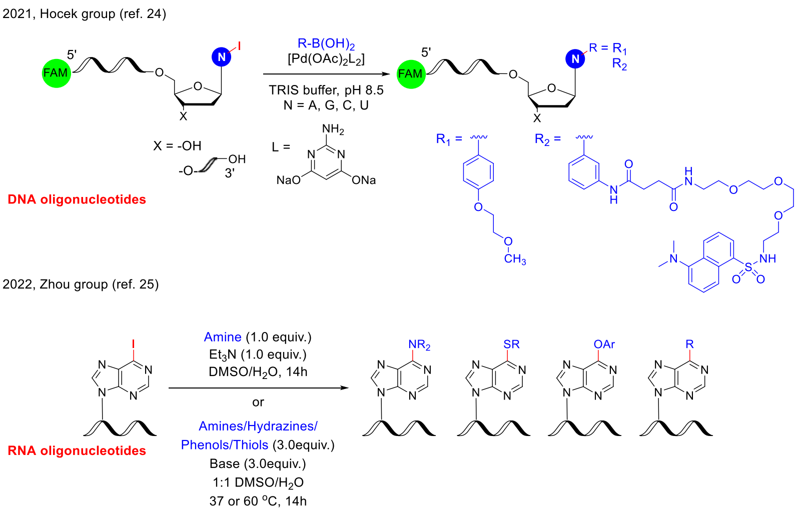

Supplementary Figure 9. examples of late-stage functionalization of purine of DNA/RNA [13-25]

2.4. Our methods of G-C8-alkylation and U-C5-alkylation

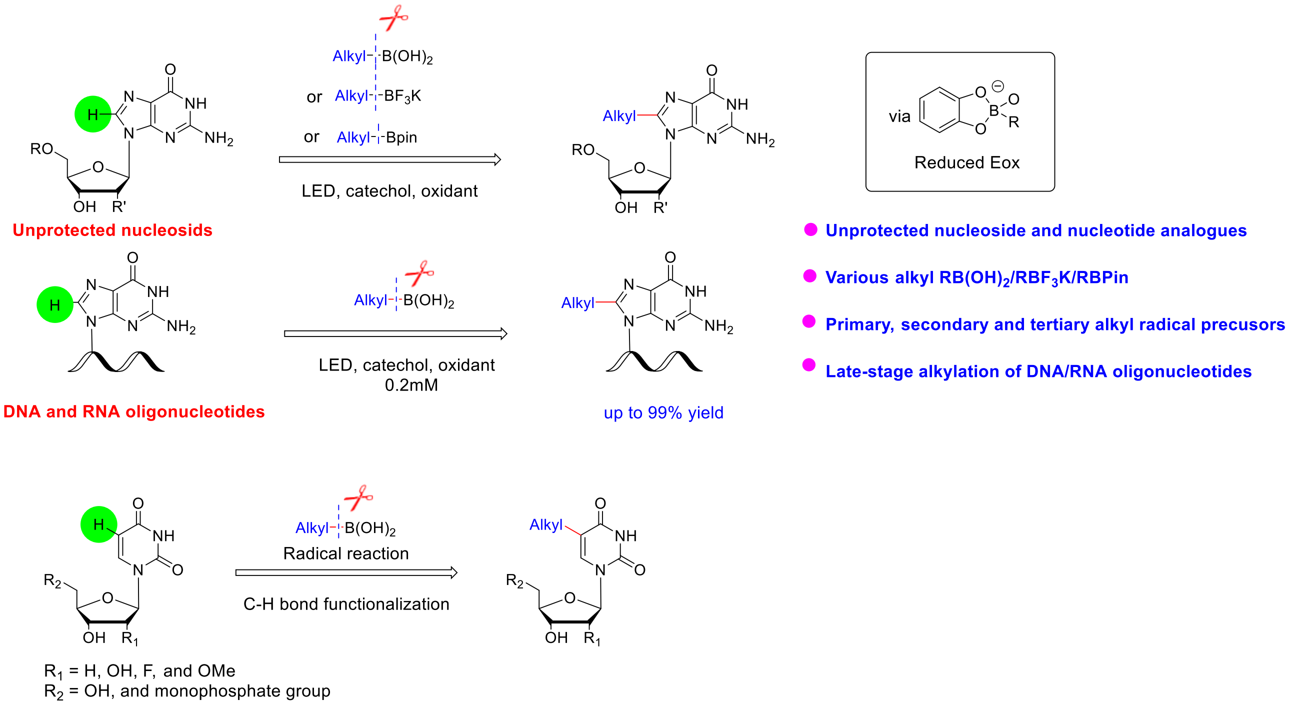

Supplementary Figure 10. Our strategy for the synthesis of C-8 alkylated nucleosides with unprotected nucleotides, RNA, and DNA oligonucleotides, as well as the C-5 alkylated uridine and their analogues.

3. List of substrates and preparation methods

3.1. Preparation of boron-containing radical precursors

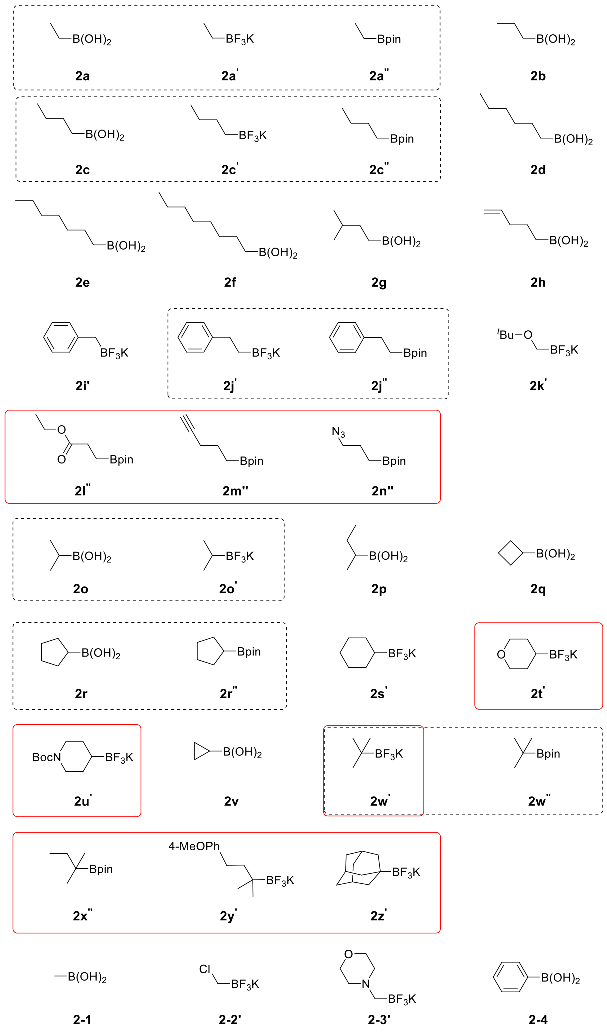

Supplementary Figure 11. List of boron-containing radical precursors

2a-2k', **2o-2s'**, **2v**, **2w''**, **2-1-2-4'** are commercially available reagents. Compounds in the red frame were prepared followed the reported literatures [6, 7, 8, 9, 10].

3.1.1. Catalytic borylation and conversion of alkyl pinacolboronate to alkyl trifluoroborate for the preparation 2t', 2u', 2w', 2y' and 2z' [26-28]

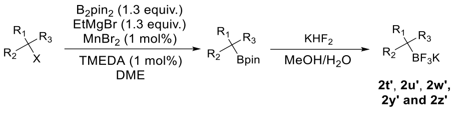

General procedure A-1:

A flame-dried vial was charged with $MnBr_2$ (1 mol %) and B_2pin_2 (1.3 equiv.) before evacuating and back-filling with N_2. DME (0.2 M) was added, followed by 1 mol % TMEDA (0.9 mol%, 4% (v/v) solution of TMEDA in DME). Next, EtMgBr (3M in Et_2O, 1.3 equiv.) was added dropwise to the reaction mixture at a rate of two drops/sec (approximately 20 secs total addition time). Finally, the substrate (1.0 equiv.) was added to the reaction mixture. The reaction mixture was stirred at room temperature for 4 hours. The reaction was quenched with 2 mL HCl (1M), extracted with Et_2O, dried over $MgSO_4$, concentrated under reduced pressure, and purified by using silica gel column chromatograph. [27]

After that, the magneton, the alkylpinacolyl boronate esters (1.0 equiv.) and sat. aq KHF_2 (5.0 equiv.) were added in a round bottom flask, dissolved in 1: 1 mixture of methanol and water as solvent. The flask was closed with a septum, and the reaction mixture was allowed to stir at room temperature for about 2 to 10 hours. The reaction mixture was evaporated to dryness, and the resulting salt was extracted several times with hot acetone. The filtrate was concentrated to about 5 mL, and precipitation was achieved by dropwise addition of the filtrate to Et_2O (100 mL) at 0 °C. The resulting product was collected by gravity filtration on a fritted funnel and dried to afford the corresponding potassium heterocyclic trifluoroborate as a white solid [26].

Trifluoro(tetrahydro-2H-pyran-4-yl)-l4-borane, potassium salt (2t') [26]

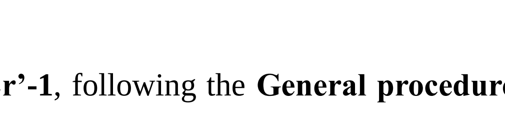

The reaction starts from the **2r'-1**, following the **General procedure A-1**, to afford the **2r'** (white solid, 743.4 mg, 21%). ^1H NMR (DMSO-d_6, 400 MHz): δ 3.75-3.72 (m, 2H), 3.15-3.09 (m, 2H),

1.28-1.15 (m, 4H), 0.22-0.17 (m, 1H). Analytical data are consistent with the previously reported methods. [26]

Tert-butyl 4-(trifluoro-l4-boraneyl)piperidine-1-carboxylate, potassium salt (2u') [26]

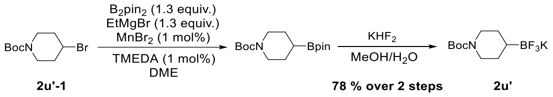

The reaction starts from the **2u'-1**, following the **General procedure A-1**, to afford the **2u'** (white solid, 1.0245 g, 78%). ^{1}H NMR (400 MHz, DMSO-d_6) δ 3.84 (d, J = 12.4 Hz, 2H), 2.49 (s, 2H),1.37 (s, 11H), 1.05-0.94 (m, 2H), 0.13-0.06 (m, 1H). Analytical data are consistent with those previously reported [26]

Tert-butyltrifluoro-l4-borane, potassium salt (2w') [27, 28]

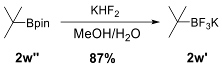

The reaction starts from the **2w''** (0.4 M in MeOH, 1.0 equiv.), following the **General procedure A-1**, to afford the **2w'** (white solid, 3.9 g, 87%). ^{1} H NMR (400 MHz, DMSO-d_6) 0.60 (s, 9H). Analytical data are consistent with those previously reported [28]

Trifluoro(4-(4-methoxyphenyl)-2-methylbutan-2-yl)-l4-borane, potassium salt (2y') [27]

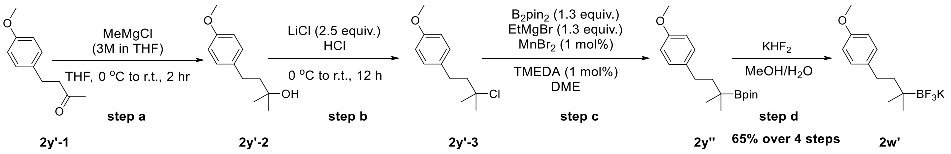

Step a

A flame-dried flask equipped with a stir bar was charged with **2y'-1** (891.0 mg, 850 µL, 5.0 mmol) and dry THF (25 mL, 0.2 M) and placed in an ice bath. Methylmagnesium chloride (3M in THF, 2.5 mL, 7.5 mmol) was added dropwise. Once the MeMgCl was added the ice bath was removed and the reaction stirred at room temperature for 2 hours. The reaction was quenched with saturated NH_4Cl (25 mL), extracted with Et_2O (3 × 25 mL), dried with $MgSO_4$, and concentrated under reduced

pressure. The reaction was purified by silica gel chromatography to afford **2y'-2** (873.3 mg, 65%) as a colorless oil [27].

Step b

A flask equipped with a stir bar was charged with LiCl (1.1 g, 2.5 equiv.). HCl (20 mL, 5M) was added and the mixture was placed in an ice bath and stirred for about 5 minutes at 0 ℃. **2y'-2** (1.9 g, 1 equiv.), neat or dissolved in a minimal amount of DCM, was added dropwise at 0 ℃. Upon addition of the **2y'-2**, the ice bath was removed and the reaction stirred for about 12 hours at room temperature. The reaction was concentrated under reduced pressure and extracted by DCM. The organic phase was washed with sat. aq $NaHCO_3$ and sat. aq NaCl, and then dried with anhydrous $NaSO_4$. After being filtered, the filtrate was concentrated under reduced pressure and purified by silica gel chromatography to afford **2y'-3** (1.1643 g, 55%) as a colorless oil [27].

Step c and step d

The reaction starts from the **2y'-3**, following the **General procedure A-1**, to afford the **2y'** (white solid, 368.0 mg, 47 %). ^{1}H NMR (400 MHz, DMSO-d_6) δ 7.00 (d, J = 8.4 Hz, 2H), 6.77 (d, J = 8.8 Hz, 2H), 3.69 (s, 3H), 2.44-2.40 (m, 2H), 1.26-1.21 (m, 2H), 0.64 (s, 6H). 6.09 (*Internal standard*, 1,3,5-Trimethoxybenzene), 3.70 (*Internal standard*, 1,3,5-Trimethoxybenzene), 3.34 (grease). Analytical data are consistent with those previously reported [27].

((3r,5r,7r)-adamantan-1-yl)trifluoro-l4-borane, potassium salt (2z') [27]

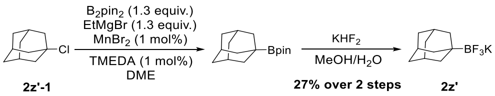

The reaction starts from the **2x'-1**, following the **General procedure A-1**, to afford the **2x'** (white solid, 332.9 mg, 27%). ^{1}H NMR (400 MHz, DMSO-d_6): δ 1.71-1.70 (m, 3H), 1.64-1.58 (m, 6H), 1.46-1.45 (m, 6H). Analytical data are consistent with those previously reported [27]

3.1.2. Copper-catalyzed conjugate addition for preparation of 2l” [29]

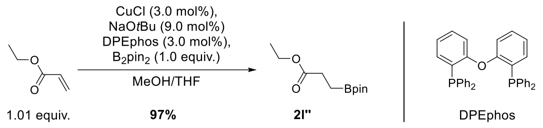

CuCl (297.0 mg, 3.0 mmol), NaO*t*-Bu (865.0 mg, 9.0 mmol) and DPEphos ligand (1.62 g, 3.0 mmol) were placed in an oven-dried Schlenk tube and THF (80 mL) was added under nitrogen. The reaction mixture was stirred for 30 min at r.t. and then, bis(pinacolato)diboron and THF (60 mL) were added. The reaction mixture was stirred for 10 min and ethyl acrylate (11.1 mL, 102 mmol) was added, followed by MeOH (16.2 mL, 400 mmol). The reaction tube was washed with THF (60 mL), sealed, and stirred until no starting material was detected by TLC. The reaction mixture was filtered through a pad of Celite and concentrated. The product **2l”** was purified by silica gel chromatography and was isolated in 97% (22.2 g) yield [29].

2l”: ^{1}H NMR (400 MHz, $CDCl_3$) δ 4.10 (q, J = 7.1 Hz, 2H), 2.41 (t, J = 7.5 Hz, 2H), 1.25-1.21 (m, 15H), 1.01 (t, J = 7.5 Hz, 2H). Analytical data are consistent with those previously reported [29].

3.1.3. Decarboxylative borylation for the preparation of 2m” [30, 31]

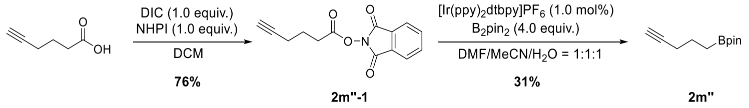

A 50 mL Schlenk tube equipped with a stir bar was charged with 5-Hexynoic acid (560.2 mg, 551 µL,1.0 equiv), N-hydroxy-phthalimide (NHPI) (815.6 mg, 1.0 equiv). Dichloromethane was added (25 mL, 0.2 M) followed by DIC (631.0 mg, 1.0 equiv), and the mixture was allowed to stir vigorously at room temperature for 45 minutes. The mixture was filtered (over Celite, SiO_2, or through a fritted funnel) and rinsed with additional CH_2Cl_2/Et_2O. The solvent was removed under reduced pressure, and purification by flash-column chromatography (eluents: DCM/ petroleum ether = 1/1) afforded the corresponding redox-active ester **2m”-1** as a white solid (983.0 mg, 76%). [30]

A solution of the **2m”-1** (257.1 mg, 1.0 equiv), $[Ir(ppy)_2dtbpy]PF_6$ (9.1 mg, 1 mol%), and B_2pin_2 (1.0 g, 4.0 equiv.) in $DMF/MeCN/H_2O$ (1/1/1, 5 mL) was added into a flame-dried Schlenk tube containing a magnetic stirring bar under N_2 environment. The reaction mixture was irradiated using a 45 W white light for 14h. After completion of the reaction as monitored by TLC, the reaction mixture was diluted by H_2O and then extracted with diethyl ether (3x). The combined organic layers

were dried over anhydrous $MgSO_4$. After the filtrate was condensed under the reduced pressure, the crude product was purified by flash-column chromatography using petroleum ether (boiling range 30-60 °C): diethyl ether (30: 1) as eluents to afford **2m”** as a colorless oil (147.5 mg, 31%) [31].

2m”-1: ^{1}H NMR (400 MHz, $CDCl_3$) δ 7.90-7.88 (m, 2H), 7.81-7.78 (m, 2H), 2.84 (t, *J* = 7.6 Hz, 2H), 2.38 (td, *J* = 6.8, 2.6 Hz, 2H), 2.05-1.98 (m, 3H). Analytical data are consistent with those previously reported [31].

2m”: ^{1}H NMR (400 MHz, $CDCl_3$) δ 2.19 (td, *J* = 7.2, 2.8 Hz, 2H), 1.93 (t, *J* = 2.7 Hz, 1H), 1.64 (p, *J* = 7.3 Hz, 2H), 1.23 (s, 12H), 0.88 (t, *J* = 7.9 Hz, 2H). Analytical data are consistent with those previously reported [31].

3.1.4. SN2 reaction for preparation of 2n” [32]

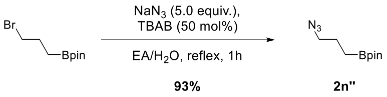

A mixture of 2-(3-bromopropyl)-4,4,5,5-tetramethyl-1,3,2-dioxaborolane (338.4 µL, 1.6 mmol, 1.0 equiv), NaN3 (520 mg, 8.0 mmol, 5.0 equiv), tetrabutylammonium bromide (250 mg, 0.8 mmol, 50 mol%), EtOAc (4 mL) and water (4 mL) was heated at reflux (about 60 °C) for 1 h. The aqueous phase was separated and extracted three times with EtOAc (3 × 1 mL). The combined organic phases were dried over $MgSO_4$, filtered, and the solvent was removed under reduced pressure. Flash S20 column chromatography on silica gel (Et_2O:pentane = 5:95) afforded the product **2n”** as a colorless liquid in 96% (315 mg). [32]

2n”: ^{1}H NMR (400 MHz, $CDCl_3$) δ 3.23 (t, *J* = 7.0 Hz, 2H), 1.70 (p, *J* = 7.3 Hz, 2H), 1.23 (s, 12H), 0.82 (t, *J* = 7.7 Hz, 2H). Analytical data are consistent with those previously reported [32].

3.1.5. Deoxygenative borylation method for preparation of 2x” [33, 34]

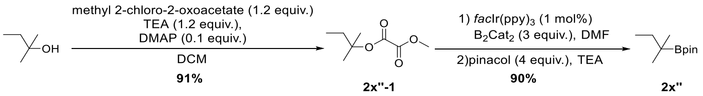

The *tert*-amyl alcohol (176.3 mg, 219 µL, 1.0 equiv.), DMAP (22.4 mg, 0.1 equiv.) and Et_3N (242.4 mg, 333 µL, 1.2 equiv.) were dissolved in CH_2Cl_2 (20 mL, 0.1 M), and methyl 2-chloro-2-oxoacetate (292.8 mg, 220µL, 1.2 equiv.) was added slowly at 0 °C. The mixture was allowed to warm to room temperature over 18 hours. Et_2O was added and the precipitate was filtered off over a plug of silica. Concentration in vacuo and subsequent purification by flash chromatography delivered the pure methyl oxalates **2x”-1** as colorless oil (319.0 mg, 91%) [33].

In a Schlenk tube equipped with a stir bar, **2x”-1** (69.6 mg, 1.0 equiv.), B_2cat_2 (285.4 mg, 3.0 equiv.) and fac. $Ir(ppy)_3$ (2.6 mg, 1.0 mol%) were dissolved in DMF (1.2 mL, 3.3 M). The solution was degassed by three freeze-pump-thaw cycles. Then, the mixture was irradiated for 15 h using a 24 W blue LED (the distance between the tube and the light source was about 1.0 cm). The setup was simultaneously cooled by a fan to keep the reaction mixture at room temperature. After completion of the reaction, Pinacol (189.0 mg, 4.0 equiv.) and Et_3N (1.9g, 2.6 mL, 47.0 equiv.) were added and

stirring was continued for 2 hours. A half saturated NH_4Cl solution was added and the aqueous layer was extracted with EtOAc (3x). The combined organic layers were washed with brine and dried over $MgSO_4$. Concentration in vacuo and subsequent purification by flash chromatography delivered the pure **2x”** as colorless oil (143.0 mg, 90%) [33].

2x”-1: ^{1}H NMR (400 MHz, $CDCl_3$) δ 3.86 (s, 3H), 1.86 (q, *J* = 7.6 Hz, 2H), 1.52 (s, 6H), 0.93 (t, *J* = 7.2 Hz, 3H). Analytical data are consistent with those previously reported [33].

2x”: ^{1}H NMR (400 MHz, $CDCl_3$) δ 1.30 (q, *J* = 7.6 Hz, 2H), 1.22 (s, 12H), 0.90 (s, 6H), 0.84 (t, *J* = 7.6 Hz, 3H). Analytical data are consistent with those previously reported [34].

3.2. List of nucleoside and nucleotide substrates

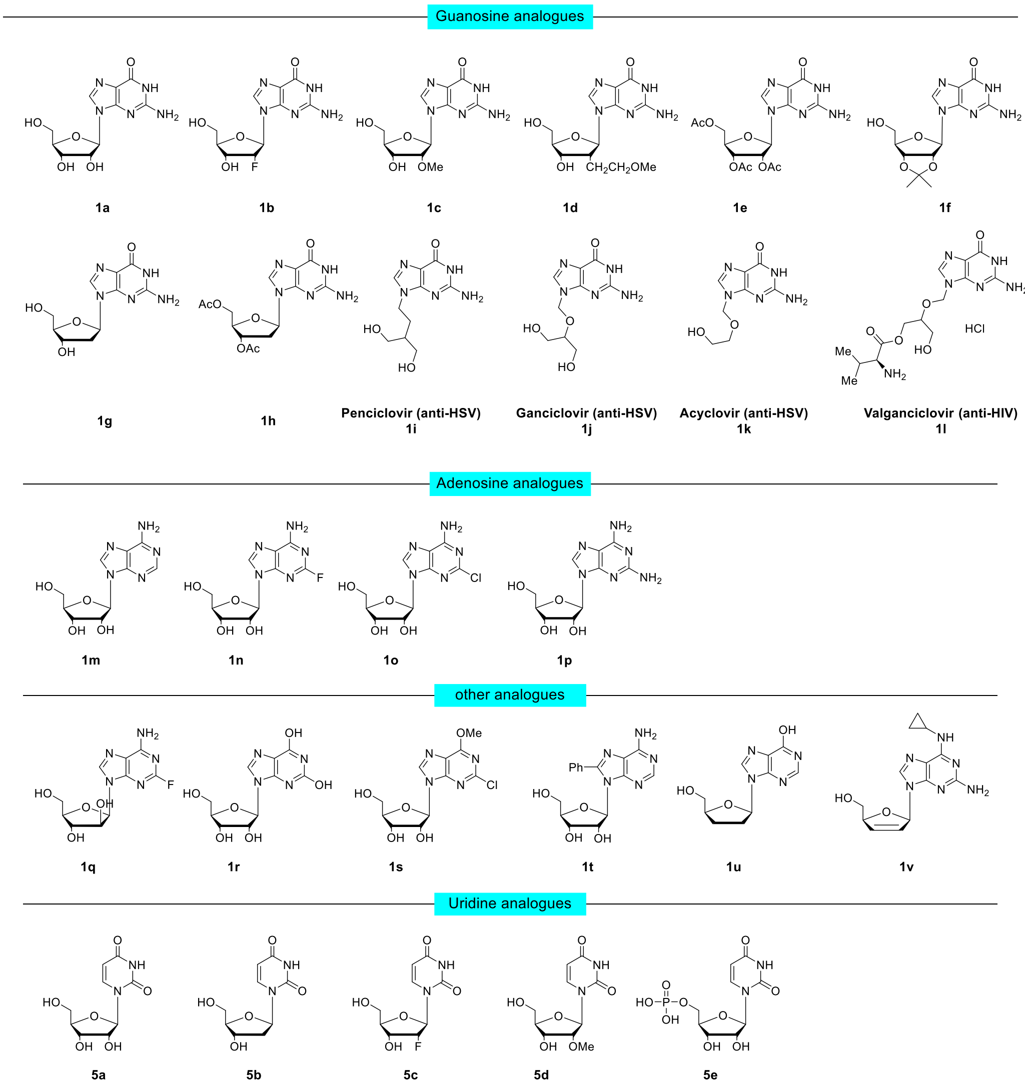

Supplementary Figure 12. List of nucleoside substrates

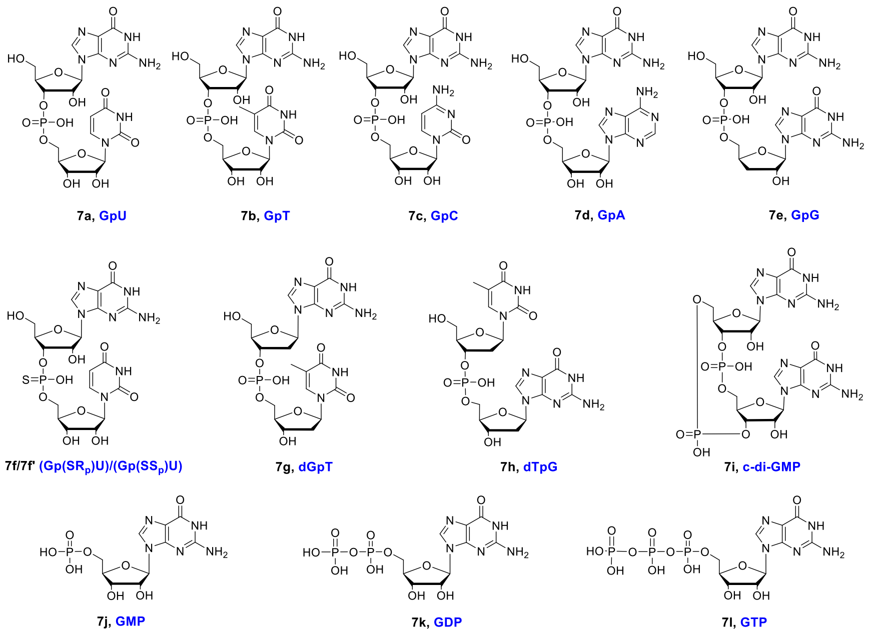

Supplementary Figure 13. List of complex nucleotide substrates

3.2.1. Preparation of compound 1e [35]

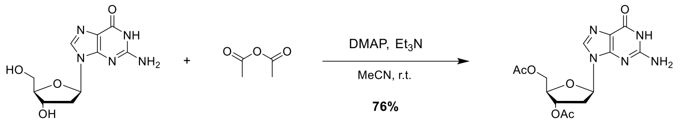

1h

Procedure: 2′-Deoxyguanosine (500.0 mg, 1.9 mmol, 1.0 equiv.), DMAP (23.0 mg, 0.19 mmol, 0.1 equiv.) and triethylamine (0.8 mL, 5.7 mmol, 3.0 equiv.) were dissolved in acetonitrile (10 mL). Acetic anhydride (0.55 mL, 5.7 mmol, 3.0 equiv.) was added and the reaction mixture was stirred for 3 h at room temperature. The reaction was stopped by addition of 1 mL methanol and the solution was concentrated to dryness. The remaining white solid was co-evaporated using acetonitrile and washed with a mixture of ethanol and diethyl ether (1: 1) to yield a white solid (504.5 mg, 76%). ^{1}H NMR (400 MHz, DMSO-d_6) δ 10.67 (s, 1H), 7.91 (s, 1H), 6.49 (s, 2H), 6.13 (dd, J = 8.6, 5.9 Hz, 1H), 5.29 (d, 6.1 Hz, 1H), 4.28-4.23 (m, 1H), 4.22-4.15 (m, 2H), 2.91 (ddd, J = 14.6, 8.6, 6.5 Hz, 1H), 2.45 (ddd, J = 14.0, 5.9, 1.7 Hz, 1H), 2.08 (s, 3H), 2.03 (s, 3H). Analytical data are consistent with those previously reported [35].

3.2.2. Preparation of dinucleotides

Preparation of RNA dinucleotides [36-38]

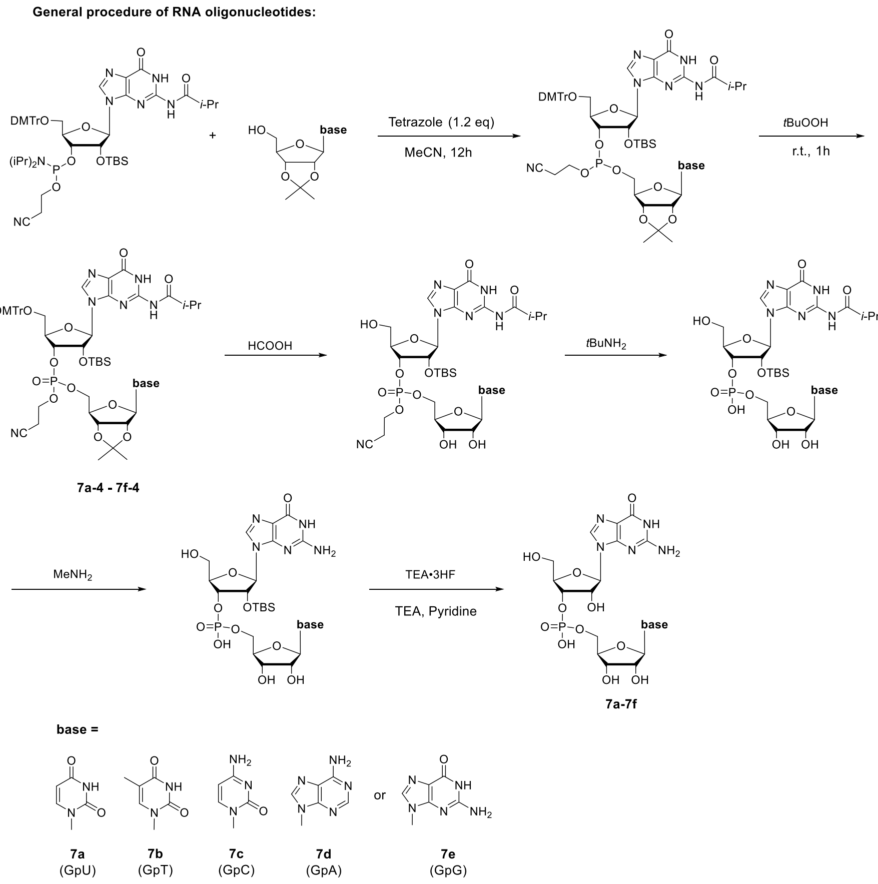

Supplementary Figure 14. Preparation of RNA dinucleotides

GpU

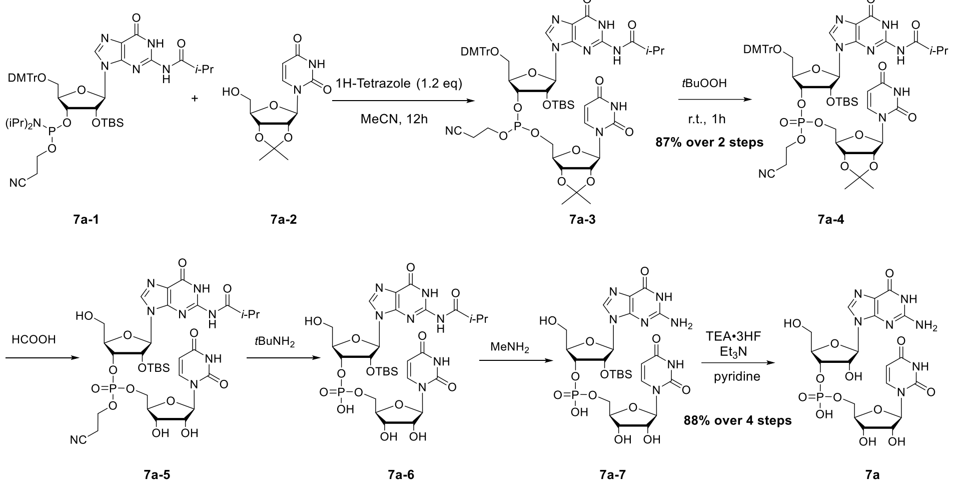

A flame-dried 25 mL Schlenk tube equipped with magnetic stir bar was charged with **7a-1** (384.0 mg, 0.4 mmol, 1.0 equiv.), compound **7a-2** (113.6 mg, 0.4 mmol, 1.0 equiv.), 1*H*-Tetrazole (33.6 mg, 0.48 mmol, 1.2 equiv.), and flame-dried 3 Å MS (powder, about 100 mg). Nitrogen was substituted for three times. The solids were suspended in anhydrous acetonitrile (2 mL, 0.2 M). TLC could be utilized to monitor the process of the reaction. After vigorous stirring at room temperature for about 12 hours, a solution of *tert*-butyl hydroperoxide (70 % in H_2O, 0.19 mL, 2.0 mmol, 5.0 equiv.) was added and the reaction mixture was stirred for about an additional 1 hour. The reaction mixture was filtered through a plug of Celite and the filter cake washed with dichloromethane. The filtrate was concentrated under reduced pressure. Then the crude material was purified by the SepaBean machine (Santai Technology Inc., China) equipped with the C18-bonded SepaFlash column to afford the desired intermediate **7a-4** (410.0 mg, 87%) as a white solid.

To a 100 mL round bottom flask equipped with magnetic stir bar, charged with compound **7a-4** (410.0 mg, 0.35 mmol), and closed tightly with rubber septum and a balloon, the formic acid (88 % in H_2O, 8 mL, 0.05 M) was added at room temperature under continuous stirring. TLC could be utilized to monitor the process of the reaction. Then the mixture was concentrated, and the residue was dissolved in a 7 mL (0.05 M) *tert*-butyl amine. After 15 minutes, the mixture was concentrated again. The 3.5 mL the methylamine (solution in methanol) (0.1 M) was added and stirred for about 2 hours. LC-MS could be utilized to monitor the process of the reaction. The solvent of this mixture was removed and the triethylamine trihydrofluoride (2.1 mL, 0.17 M), triethylamine (2.5 mL, 0.14 M), and pyridine (1.3 mL, 0.26 M) were added. Then the round bottom flask was placed in oil bath and heated at 50 °C overnight. The mixture was concentrated to a small volume and purified by the above mentioned SepaBean machine to afford the final product **7a** (181.9 mg, 88%).

(2R,3S,4R,5R)-5-(2-amino-6-oxo-1,6-dihydro-9H-purin-9-yl)-4-hydroxy-2-(hydroxymethyl)tetrahydrofuran-3-yl (((2R,3S,4R,5R)-5-(2,4-dioxo-3,4-dihydropyrimidin-1(2H)-yl)-3,4-dihydroxytetrahydrofuran-2-yl)methyl) hydrogen phosphate (Compound 7a)

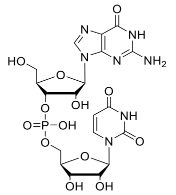

After purification by column chromatography (C18 Spherical silica) using MeOH/H_2O as the eluents, Compound **7a** was obtained as a white solid (181.9 mg, 77%). mp 224.2-226.0 °C. $[\alpha]_D^{25}$ 18.82 (c 0.170, H_2O). This was followed by exchange of the counterions [Amberlite IR120 (Na^+)], in order to improve the solubility of the product in D_2O and simplify the NMR spectrum. ^1H NMR (500 MHz, D_2O) δ 7.95 (s, 1H), 7.82 (d, *J* = 8.1 Hz, 1H), 5.89-5.83 (m, 2H), 5.70 (d, *J* = 8.0 Hz, 1H), 4.67 (dt, *J* = 8.0, 4.9 Hz, 1H), 4.39 (q, *J* = 3.9 Hz, 1H), 4.34-4.22 (m, 4H), 4.14-4.11 (m, 1H), 3.95-3.92 (dd, *J* = 13.0, 2.7 Hz, 1H), 3.84 (dd, *J* = 12.9, 3.9 Hz, 1H). Analytical data are consistent with those previously reported [51]. ^{13}C NMR (126 MHz, D_2O) δ 165.7, 158.6, 153.6, 151.4, 150.8, 141.0, 137.5, 116.6, 102.0, 89.1, 88.4, 83.9 (d, J_{C-P} = 4.7 Hz), 82.5 (d, J_{C-P} = 9.1 Hz), 73.8, 73.6 (d, J_{C-P} = 5.7 Hz), 72.7 (d, J_{C-P} = 4.0 Hz), 69.0, 64.2 (d, J_{C-P} = 5.3 Hz), 60.7. ^{31}P NMR (202 MHz, D_2O) δ -0.74. HRMS-ESI m/z calcd. for $C_{19}H_{24}N_{13}O_{12}P$ $[M+H]^+$ 590.1248. found 590.1240.

GpT

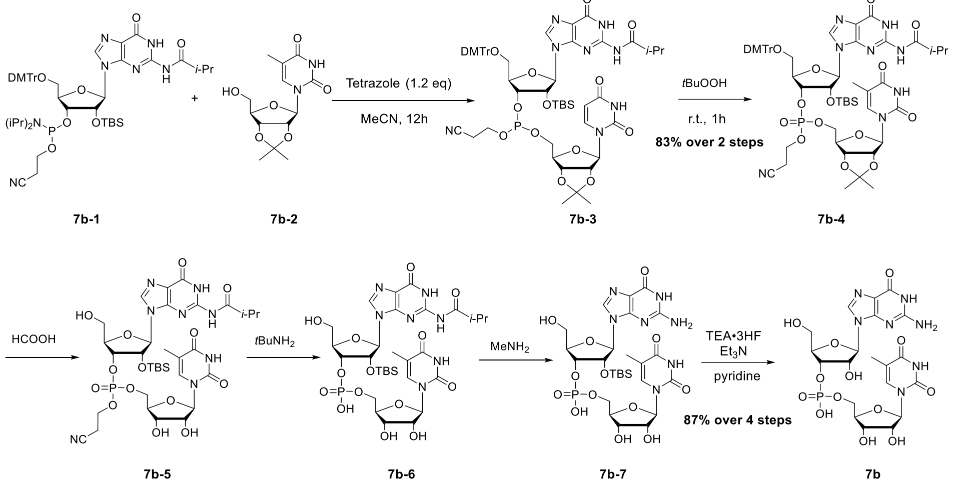

A flame-dried 25 mL Schlenk tube equipped with magnetic stir bar was charged with **7b-1** (481.5 mg, 0.5 mmol, 1.0 equiv.), compound **7b-2** (149.0 mg, 0.5 mmol, 1.0 equiv.), 1*H*-Tetrazole (42.0 mg, 0.6 mmol, 1.2 equiv.), and flame-dried 3 Å MS (powder, about 125 mg). Nitrogen was substituted for three times. The solids were suspended in anhydrous acetonitrile (2.5 mL, 0.2 M). TLC could be utilized to monitor the process of the reaction. After vigorous stirring at room temperature for about 12 hours, a solution of *tert*-butyl hydroperoxide (70 % in H_2O, 0.24 mL, 2.5 mmol, 5.0 equiv.) was added and the reaction mixture was stirred for about an additional 1 hour. The reaction mixture was filtered through a plug of Celite and the filter cake washed with dichloromethane. The filtrate was concentrated under reduced pressure. Then the crude material was purified by the SepaBean machine (Santai Technology Inc., China) equipped with the C18-bonded SepaFlash column to afford the desired intermediate **7b-4** (490.5 mg, 83%) as a white solid.

To a 100 mL round bottom flask equipped with magnetic stir bar, charged with compound **7b-4** (480.0 mg, 0.41 mmol), and closed tightly with rubber septum and a balloon, the formic acid (88 % in H_2O, 9.3 mL, 0.05 M) was added at room temperature under continuous stirring. TLC could be utilized to monitor the process of the reaction. Then the mixture was concentrated, and the residue was dissolved in an 8.2 mL (0.05 M) *tert*-butyl amine. After 15 minutes, the mixture was concentrated again. The 4.1 mL the methylamine (solution in methanol) (0.1 M) was added and stirred for about 2 hours. LC-MS could be utilized to monitor the process of the reaction. The solvent of this mixture was removed and the triethylamine trihydrofluoride (2.4 mL, 0.17 M), triethylamine (2.9 mL, 0.14 M), and pyridine (1.6 mL, 0.26 M) were added. Then the round bottom flask was placed in oil bath and heated at 50 °C overnight. The mixture was concentrated to a small volume and purified by the above mentioned SepaBean machine to afford the final product **7b** (213.1 mg, 87%).

(2R,3S,4R,5R)-5-(2-amino-6-oxo-1,6-dihydro-9H-purin-9-yl)-4-hydroxy-2-(hydroxymethyl)tetrahydrofuran-3-yl (((2R,3S,4R,5R)-3,4-dihydroxy-5-(5-methyl-2,4-dioxo-3,4-dihydropyrimidin-1(2H)-yl)tetrahydrofuran-2-yl)methyl) hydrogen phosphate (Compound 7b)

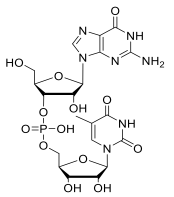

After purification by column chromatography (C18 Spherical silica) using MeOH/H2O as eluents, compound **7b** was obtained as a white solid (213.1 mg, 72%). mp 198.0-204.4 °C. $[\alpha]_D^{25}$ 37.02 (c 0.235, H_2O). This was followed by exchange of the counterions [Amberlite IR120 (Na^+)], in order to improve the solubility of the product in D_2O and simplify the NMR spectrum. ^{1}H NMR (400 MHz, D_2O) δ 7.96 (s, 1H), 7.60 (d, J = 1.4 Hz, 1H), 5.83 (d, J = 3.3 Hz, 1H), 5.79 (d, J = 2.8 Hz, 1H), 4.77-4.75 (m, 1H), 4.63-4.58 (m, 1H), 4.40-4.36 (m, 2H), 4.31-4.18 (m, 3H), 4.12-4.08 (m, 1H), 3.99 (dd, J = 13.1, 2.5 Hz, 1H), 3.84 (dd, J = 13.1, 3.8 Hz, 1H), 1.59 (s, 3H). ^{13}C NMR (126 MHz, D_2O) δ 165.6, 158.2, 153.4, 151.3, 150.4, 136.9, 136.0, 116.6, 111.0, 89.5, 89.1, 83.1 (d, J_{C-P} = 6.4 Hz), 82.1 (d, J_{C-P} = 9.6 Hz), 74.1, 72.7, 72.5 (d, J_{C-P} = 4.0 Hz), 68.6, 63.7 (d, J_{C-P} = 4.3 Hz), 63.7, 60.1, 11.3. ^{31}P NMR (202 MHz, D_2O) δ -1.01. HRMS-ESI m/z calcd. for $C_{20}H_{26}N_7O_{13}P$ $[M+H]^+$ 604.1404. found 604.1401.

GpC

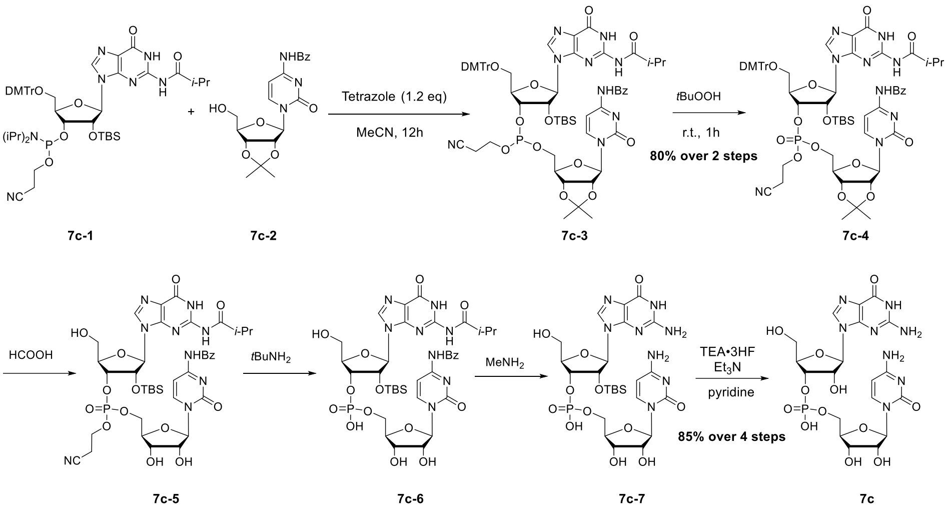

A flame-dried 25 mL Schlenk tube equipped with magnetic stir bar was charged with **7c-1** (481.5 mg, 0.5 mmol, 1.0 equiv.), compound **7c-2** (193.6 mg, 0.5 mmol, 1.0 equiv.), 1*H*-Tetrazole (42.0 mg, 0.6 mmol, 1.2 equiv.), and flame-dried 3 Å MS (powder, about 125 mg). Nitrogen was substituted for three times. The solids were suspended in anhydrous acetonitrile (2.5 mL, 0.2 M). TLC could be utilized to monitor the process of the reaction. After vigorous stirring at room temperature for about 12 hours, a solution of *tert*-butyl hydroperoxide (70 % in H_2O, 0.24 mL, 2.5 mmol, 5.0 equiv.) was added and the reaction mixture was stirred for about an additional 1 hour. The reaction mixture was filtered through a plug of Celite and the filter cake washed with dichloromethane. The filtrate was concentrated under reduced pressure. Then the crude material was purified by the SepaBean machine (Santai Technology Inc., China) equipped with the C18-bonded SepaFlash column to afford the desired intermediate **7c-4** (508.0 mg, 80%) as a white solid.

To a 100 mL round bottom flask equipped with magnetic stir bar, charged with compound **7c-4** (500.0 mg, 0.39 mmol), and closed tightly with rubber septum and a balloon, the formic acid (88 % in H_2O, 8.9 mL, 0.05 M) was added at room temperature under continuous stirring. TLC could be utilized to monitor the process of the reaction. Then the mixture was concentrated, and the residue was dissolved in a 7.8 mL (0.05 M) *tert*-butyl amine. After 15 minutes, the mixture was concentrated again. The 3.9 mL the methylamine (solution in methanol) (0.1 M) was added and stirred for about 2 hours. LC-MS could be utilized to monitor the process of the reaction. The solvent of this mixture was removed and the triethylamine trihydrofluoride (2.3 mL, 0.17 M), triethylamine (2.8 mL, 0.14 M), and pyridine (1.5 mL, 0.26 M) were added. Then the round bottom flask was placed in oil bath and heated at 50 °C overnight. The mixture was concentrated to a small volume and purified by the above mentioned SepaBean machine to afford the final product **7c** (196.6 mg, 85%).

((2R,3S,4R,5R)-5-(4-amino-2-oxopyrimidin-1(2H)-yl)-3,4-dihydroxytetrahydrofuran-2-yl)methyl ((2R,3S,4R,5R)-5-(2-amino-6-oxo-1,6-dihydro-9H-purin-9-yl)-4-hydroxy-2-(hydroxymethyl)tetrahydrofuran-3-yl) hydrogen phosphate (Compound 7c)

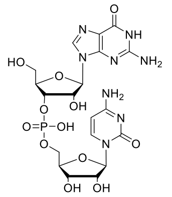

After purification by column chromatography (C18 Spherical silica) using MeOH/H_2O as the eluent, compound **7c** was obtained as a white solid (196.6 mg, 68%). mp 221.7-232.7 °C. $[\alpha]_D^{25}$ 31.03 (c 0.0870, H_2O). This was followed by exchange of the counterions [Amberlite IR120 (Na^+)], in order to improve the solubility of the product in D_2O and simplify the NMR spectrum. ^1H NMR (400 MHz, D_2O) δ 8.01 (s, 1H), 7.87 (d, J = 7.7 Hz, 1H), 5.86 (d, J = 2.5 Hz, 1H), 5.82 (d, J = 3.1 Hz, 1H), 5.75 (d, J = 7.7 Hz, 1H), 4.77-4.72 (m, 1H), 4.64-4.59 (m, 1H), 4.42-4.37 (m, 2H), 4.30-4.25 (m, 2H), 4.22-4.20 (m, 1H), 4.17-4.11 (m, 1H), 4.00 (dd, J = 13.1, 2.6 Hz, 1H), 3.87 (dd, J = 13.0, 3.9 Hz, 1H). ^{13}C NMR (126 MHz, D_2O) δ 163.5, 158.5, 154.2, 153.5, 150.3, 140.8, 136.7, 116.3, 94.7, 90.2, 89.6, 82.8 (d, J_{C-P} = 5.0 Hz), 81.6 (d, J = 9.1 Hz), 74.4, 72.5, 72.2, 67.8, 63.2, 60.0. ^{31}P NMR (202 MHz, D_2O) δ -1.10. HRMS-ESI m/z calcd. for $C_{19}H_{25}N_8O_{12}P$ $[M+H]^+$ 589.1408. found 589.1393.

GpA

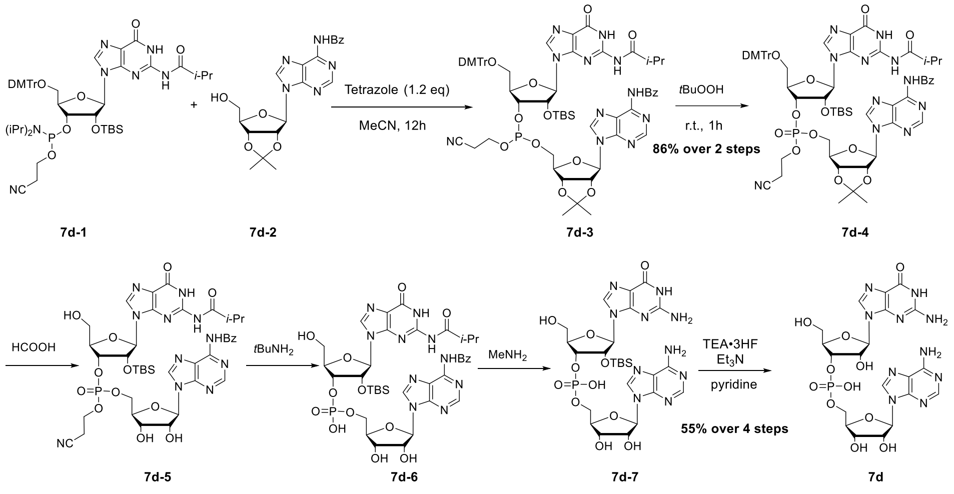

A flame-dried 50 mL Schlenk tube equipped with magnetic stir bar was charged with **7d-1** (1.94 g, 2.0 mmol, 1.0 equiv.), compound **7d-2** (822.3 mg, 2.0 mmol, 1.0 equiv.), 1*H*-Tetrazole (168.0 mg, 2.4 mmol, 1.2 equiv.), and flame-dried 3 Å MS (powder, about 625 mg). Nitrogen was substituted for three times. The solids were suspended in anhydrous acetonitrile (10 mL, 0.2 M). TLC could be utilized to monitor the process of the reaction. After vigorous stirring at room temperature for about 12 hours, a solution of *tert*-butyl hydroperoxide (70 % in H_2O, 0.96 mL, 10 mmol, 5.0 equiv.) was added and the reaction mixture was stirred for about an additional 1 hour. The reaction mixture was filtered through a plug of Celite and the filter cake washed with dichloromethane. The filtrate was concentrated under reduced pressure. Then the crude material was purified by the SepaBean machine (Santai Technology Inc., China) equipped with the C18-bonded SepaFlash column to afford the desired intermediate **7d-4** (2.22 mg, 86%) as a white solid.

To a 100 mL round bottom flask equipped with magnetic stir bar, charged with compound **7d-4** (1.53 g, 1.18 mmol), and closed tightly with rubber septum and a balloon, the formic acid (88 % in H_2O, 27 mL, 0.05 M) was added at room temperature under continuous stirring. TLC could be utilized to monitor the process of the reaction. Then the mixture was concentrated, and the residue was dissolved in a 24 mL (0.05 M) *tert*-butyl amine. After 15 minutes, the mixture was concentrated again. The 12 mL the methylamine (solution in methanol) (0.1 M) was added and stirred for about 2 hours. LC-MS could be utilized to monitor the process of the reaction. The solvent of this mixture was removed and the triethylamine trihydrofluoride (6.9 mL, 0.17 M), triethylamine (8.4 mL, 0.14 M), and pyridine (4.5 mL, 0.26 M) were added. Then the round bottom flask was placed in oil bath and heated at 50 °C overnight. The mixture was concentrated to a small volume and purified by the above mentioned SepaBean machine to afford the final product **7d** (514.1 mg, 55%).

(2R,3S,4R,5R)-5-(2-amino-6-oxo-1,6-dihydro-9H-purin-9-yl)-4-hydroxy-2-(hydroxymethyl)tetrahydrofuran-3-yl (((2R,3S,4R,5R)-5-(6-amino-9H-purin-9-yl)-3,4-dihydroxytetrahydrofuran-2-yl)methyl) hydrogen phosphate (compound 7d)

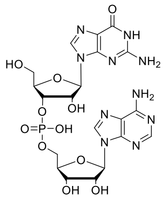

After purification by column chromatography (C18 Spherical silica) using MeOH/H_2O as the eluents, compound **7d** was obtained as a white solid (514.1 mg, 47 %). mp 235.7-245.7 °C. $[\alpha]_D^{25}$ -38.10 (c 0.0420, H_2O). This was followed by exchange of the counterions [Amberlite IR120 (Na^+)], in order to improve the solubility of the product in D_2O and simplify the NMR spectrum. ^1H NMR (400 MHz, D_2O) δ 8.30 (s, 1H), 8.11 (s, 1H), 7.87 (s, 1H), 6.04 (d, *J* = 4.4 Hz, 1H), 5.67 (d, *J* = 4.4 Hz, 1H), 4.68-4.63 (m, 2H), 4.59 (t, *J* = 4.7 Hz, 1H), 4.52 (t, *J* = 5.2 Hz, 1H), 4.40-4.25 (m, 3H), 4.19-4.14 (m, 1H), 3.85-3.75 (m, 2H). ^{13}C NMR (126 MHz, DMSO-d_6) δ 156.8, 155.6, 153.9, 152.1, 151.6, 149.5, 139.7, 135.3, 119.0, 116.6, 87.1, 85.8, 84.6, 83.6, 75.0, 73.5, 73.3, 70.8, 65.4, 61.4. ^{31}P NMR (202 MHz, D_2O) δ -0.74. HRMS-ESI m/z calcd. for $C_{20}H_{25}N_{10}O_{11}NaP$ $[M+Na]^+$ 635.1344. found 635.1344.

GpG

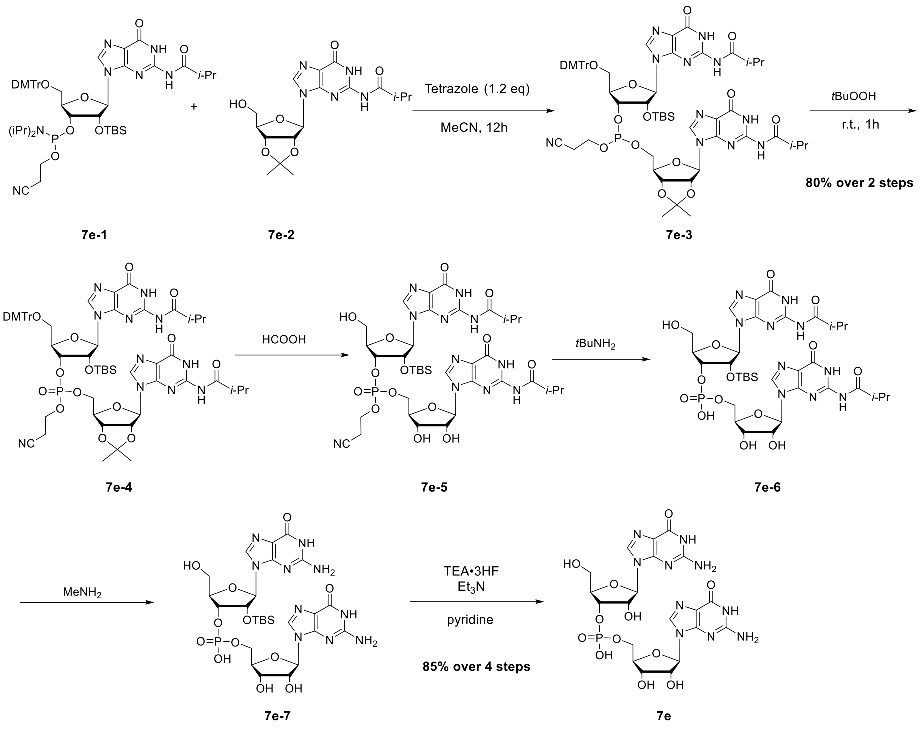

A flame-dried 25 mL Schlenk tube equipped with magnetic stir bar was charged with **7e-1** (384.0 mg, 0.4 mmol, 1.0 equiv.), compound **7e-2** (157.2 mg, 0.4 mmol, 1.0 equiv.), 1*H*-Tetrazole (33.6 mg, 0.48 mmol, 1.2 equiv.), and flame-dried 3 Å MS (powder, about 100 mg). Nitrogen was substituted for three times. The solids were suspended in anhydrous acetonitrile (2 mL, 0.2 M). TLC could be utilized to monitor the process of the reaction. After vigorous stirring at room temperature for about 12 hours, a solution of *tert*-butyl hydroperoxide (70 % in H_2O, 0.19 mL, 2.0 mmol, 5.0 equiv.) was added and the reaction mixture was stirred for about an additional 1 hour. The reaction mixture was filtered through a plug of Celite and the filter cake washed with dichloromethane. The filtrate was concentrated under reduced pressure. Then the crude material was purified by the SepaBean machine (Santai Technology Inc., China) equipped with the C18-bonded SepaFlash column to afford the desired intermediate **7e-4** (410.0 mg, 80%) as a white solid.

To a 100 mL round bottom flask equipped with magnetic stir bar, charged with compound **7e-4** (410.0 mg, 0.32 mmol), and closed tightly with rubber septum and a balloon, the formic acid (88 % in H_2O, 7.3 mL, 0.05 M) was added at room temperature under continuous stirring. TLC could be utilized to monitor the process of the reaction. Then the mixture was concentrated, and the residue was dissolved in a 6.4 mL (0.05 M) *tert*-butyl amine. After 15 minutes, the mixture was concentrated again. The 3.2 mL the methylamine (solution in methanol) (0.1 M) was added and stirred for about 2 hours. LC-MS could be utilized to monitor the process of the reaction. The solvent of this mixture was removed and

the triethylamine trihydrofluoride (1.9 mL, 0.17 M), triethylamine (2.3 mL, 0.14 M), and pyridine (1.2 mL, 0.26 M) were added. Then the round bottom flask was placed in oil bath and heated at 50 °C overnight. The mixture was concentrated to a small volume and purified by the above mentioned SepaBean machine to afford the final product **7e** (200.0 mg, 85%).

((2R,3S,4R,5R)-5-(2-amino-6-oxo-1,6-dihydro-9H-purin-9-yl)-3,4-dihydroxytetrahydrofuran-2-yl)methyl ((2R,3S,4R,5R)-5-(2-amino-6-oxo-1,6-dihydro-9H-purin-9-yl)-4-hydroxy-2-(hydroxymethyl)tetrahydrofuran-3-yl) hydrogen phosphate (Compound 7e)

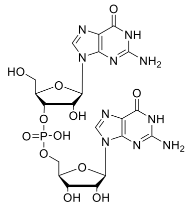

After purification by column chromatography (C18 Spherical silica) using MeOH/H_2O as the eluents, compound **7e** was obtained as a white solid (200.0 mg, 72%). mp >280 °C. $[\alpha]_D^{25}$ 25.81 (c 0.310, H_2O). ^{1}H NMR (400 MHz, DMSO-*d₆*) δ 10.69 (s, 2H), 7.98-7.96 (m, 2H), 6.53 (s, 4H), 5.77-5.71 (m, 2H), 4.66-4.59 (m, 2H), 4.49-4.40 (m, 2H), 4.17-4.10 (m, 4H), 4.08-4.04 (m, 3H), 3.61-3.50 (m, 2H). ^{13}C NMR (126 MHz, DMSO-d_6) δ 156.6, 156.6, 153.9, 153.9, 151.5, 151.4, 135.5, 135.4, 116.3, 116.2, 86.5, 85.4, 84.4, 82.9 (d, $J_{C\text{-}P}$ = 8.3 Hz), 76.3 (d, $J_{C\text{-}P}$ = 4.8 Hz), 73.3, 72.6, 70.4, 66.0 (d, $J_{C\text{-}P}$ = 5.5 Hz), 61.2. ^{31}P NMR (202 MHz, DMSO-*d6*) δ -0.137. HRMS-ESI m/z calcd. for $C_{20}H_{25}N_{10}O_{12}P$ $[M+H]^+$ 629.1469. found 629.1468.

GpU (S)

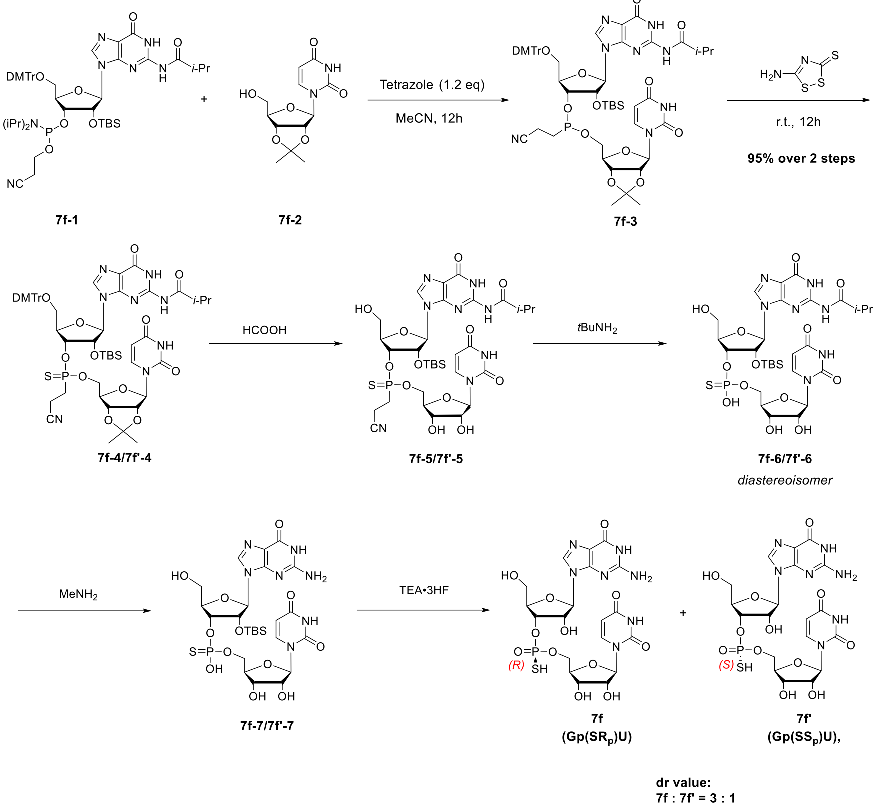

A flame-dried 50 mL Schlenk tube equipped with magnetic stir bar was charged with **7f-1** (1.94 g, 2.0 mmol, 1.0 equiv.), compound **7f-2** (568.0 mg, 2.0 mmol, 1.0 equiv.), 1*H*-Tetrazole (140.0 mg, 2.4 mmol, 1.2 equiv.), and flame-dried 3 Å MS (powder, about 500 mg). Nitrogen was substituted for three times. The solids were suspended in anhydrous acetonitrile (10 mL, 0.2 M). TLC could be utilized to monitor the process of the reaction. After vigorous stirring at room temperature for about 12 hours, 5-Amino-3H-1,2,4-dithiazole-3-thione (915.0 mg, 6.1mmol, 1.54 equiv.) was added and the reaction mixture was stirred for about an additional 6 hours. The reaction mixture was filtered through a plug of Celite and the filter cake washed with dichloromethane. The filtrate was concentrated under reduced pressure. Then the crude material was purified by the SepaBean machine (Santai Technology Inc., China) equipped with the C18-bonded SepaFlash column to afford the diastereoisomer **7f-4** and **7f'-4** (1.7 g, 95%).

To a 100 mL round bottom flask equipped with magnetic stir bar, charged with diastereoisomer **7f-4** and **7f'-4** (1.6 g, 1.37 mmol), and closed tightly with rubber septum and a balloon, the formic acid (88 % in H_2O, 31 mL, 0.05 M) was added at room temperature under continuous stirring. TLC could be utilized to monitor the process of the reaction. Then the mixture was concentrated, and the residue was dissolved in a 27.4 mL (0.05 M) *tert*-butyl amine. After 15 minutes, the mixture was concentrated

again. The 13.7 mL the methylamine (solution in methanol) (0.1 M) was added and stirred for about 2 hours. LC-MS could be utilized to monitor the process of the reaction. The solvent of this mixture was removed and the triethylamine trihydrofluoride (8.1 mL, 0.17 M), triethylamine (9.8 mL, 0.14 M), and pyridine (5.3 mL, 0.26 M) were added. Then the round bottom flask was placed in oil bath and heated at 50 °C overnight. The mixture was concentrated to a small volume and purified by the above mentioned SepaBean machine to afford the final products **7f** (488.0 mg) and **7f'** (160.0 mg).

After purification by column chromatography (C18 Spherical silica) using MeOH/H_2O as the eluents, **7f** was obtained as a white solid (488.0 mg). mp 144.5-149.5 °C. $[\alpha]_D^{25}$ -3.98 (c 0.176, H_2O). **7f'** was obtained as a white solid (160.0 mg). mp 221.7-230.2 °C. $[\alpha]_D^{25}$ -1.72 (c 0.116, H_2O). The total yield was 44%.

Sodium O-((2R,3S,4R,5R)-5-(2-amino-6-oxo-1,6-dihydro-9H-purin-9-yl)-4-hydroxy-2-(hydroxymethyl)tetrahydrofuran-3-yl) O-(((2R,3S,4R,5R)-5-(2,4-dioxo-3,4-dihydropyrimidin-1(2H)-yl)-3,4-dihydroxytetrahydrofuran-2-yl)methyl) (R)-phosphorothioate (Compound 7f) (Gp(SRp)U)

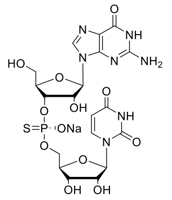

This was followed by exchange of the counterions [Amberlite IR120 (Na^+)], in order to improve the solubility of the product in D2O and simplify the NMR spectrum. The sodium salt of **7f** was obtained as a white solid. ^{1}H NMR (400 MHz, D_2O) δ 7.94 (s, 1H), 7.91 (d, *J* = 8.2 Hz, 1H), 5.83 (d, *J* = 1.8 Hz, 1H), 5.82 (d, *J* = 1.3 Hz, 1H), 5.67 (d, *J* = 8.1 Hz, 1H), 4.89-4.84 (m, 1H), 4.78-4.77 (m, 1H), 4.43-4.40 (m, 1H), 4.34 (ddd, *J* = 11.8, 5.4, 2.2 Hz, 1H), 4.30-4.23 (m, 3H), 4.15 (ddd, *J* = 11.7, 5.2, 2.5 Hz, 1H), 3.97 (dd, *J* = 13.0, 2.6 Hz, 1H), 3.87 (dd, *J* = 13.0, 3.9 Hz, 1H). ^{13}C NMR (126 MHz, D_2O) δ 165.6, 158.4, 153.5, 151.3, 150.7, 141.2, 137.4, 116.6, 102.0, 89.1, 88.8, 83.6 (d, J_{C-P} = 5.0 Hz), 82.4 (d, J_{C-P} = 9.4 Hz), 74.0, 73.9 (d, J_{C-P} = 5.2 Hz), 72.6 (d, J_{C-P} = 4.2 Hz), 69.0, 64.0 (d, J_{C-P} = 7.0 Hz), 60.6. ^{31}P NMR (202 MHz, D_2O) δ 56.70. HRMS-ESI m/z calcd. for $C_{19}H_{24}N_7O_{12}PS$ $[M+H]^+$ 606.1019. found 606.1008.

Sodium O-((2R,3S,4R,5R)-5-(2-amino-6-oxo-1,6-dihydro-9H-purin-9-yl)-4-hydroxy-2-(hydroxymethyl)tetrahydrofuran-3-yl) O-(((2R,3S,4R,5R)-5-(2,4-dioxo-3,4-dihydropyrimidin-1(2H)-yl)-3,4-dihydroxytetrahydrofuran-2-yl)methyl) (S)-phosphorothioate (Compound 7f') (Gp(SSp)U)

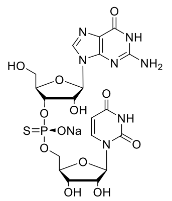

This was followed by exchange of the counterions [Amberlite IR120 (Na^+)], in order to improve the solubility of the product in D2O and simplify the NMR spectrum. The sodium salt of **7f’** was obtained as a white solid. ^{1}H NMR (400 MHz, D_2O) δ 7.94 (s, 1H), 7.88 (d, *J* = 8.1 Hz, 1H), 5.86 (d, *J* = 3.9 Hz, 1H), 5.83 (d, *J* = 5.1 Hz, 1H), 5.73 (d, *J* = 8.1 Hz, 1H), 4.88-4.83 (m, 1H), 4.81(m, 1H), 4.40 (q, *J* = 3.6 Hz, 1H), 4.31-4.25 (m, 4H), 4.21-4.17 (m, 1H), 3.90 (dd, *J* = 13.0, 2.7 Hz, 1H), 3.82 (dd, *J* = 12.9, 3.8 Hz, 1H). ^{13}C NMR (126 MHz, D_2O) δ 165.8, 158.7, 153.6, 151.5, 151.0, 141.3, 137.6, 116.6, 102.1, 89.0, 88.2, 94.0 (d, $J_{C\text{-}P}$ = 4.3 Hz), 82.6 (d, $J_{C\text{-}P}$ = 9.4 Hz), 74.0, 74.0 (d, $J_{C\text{-}P}$ = 6.3 Hz), 72.6 (d, $J_{C\text{-}P}$ = 4.4 Hz), 69.2, 64.8 (d, $J_{C\text{-}P}$ = 5.7 Hz), 60.8. ^{31}P NMR (202 MHz, D_2O) δ 55.98. HRMS-ESI m/z calcd. for $C_{19}H_{24}N_7O_{12}PS$ $[M+H]^+$ 606.1019. found 606.1008.

Preparation of DNA dinucleotides [39-41]

dGpT

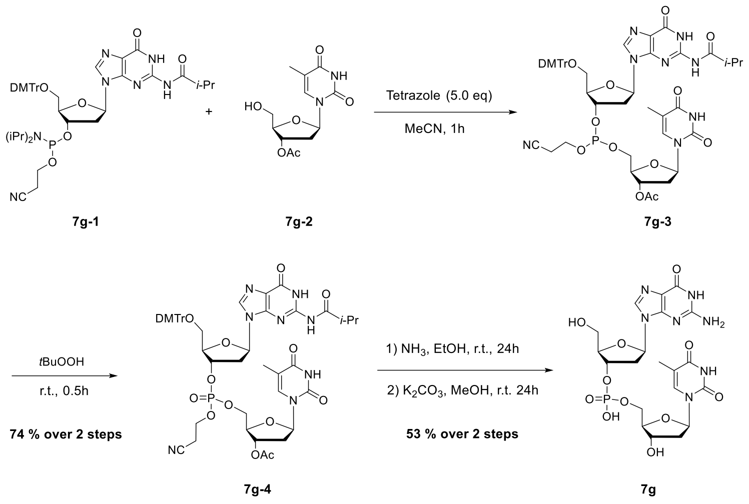

A flame-dried 50 mL Schlenk tube equipped with magnetic stir bar was charged with **7g-1** (2.52 g, 3.0 mmol, 1.0 equiv.), compound **7b-2** (852.0 mg, 3.0 mmol, 1.0 equiv.), 1*H*-Tetrazole (840.0 mg, 12.0 mmol, 4.0 equiv.), and flame-dried 3 Å MS (powder, about 750 mg). Nitrogen was substituted for three times. The solids were suspended in anhydrous acetonitrile (25 mL, 0.12 M). TLC could be utilized to monitor the process of the reaction. After vigorous stirring at room temperature for about 1 hour, a solution of *tert*-butyl hydroperoxide (70 % in H_2O, 1.44 mL, 15 mmol, 5.0 equiv.) was added and the reaction mixture was stirred for about an additional 0.5 hour. The reaction mixture was filtered through a plug of Celite and the filter cake washed with dichloromethane. The filtrate was concentrated under reduced pressure. Then the crude material was purified by the SepaBean machine (Santai Technology Inc., China) equipped with the C18-bonded SepaFlash column to afford the desired intermediate **7g-4** (2.30 g, 74 %) as a white solid.

To a 100 mL round bottom flask equipped with magnetic stir bar, charged with compound **7g-4** (2.30 g, 2.25 mmol), and closed tightly with rubber septum and a balloon, 25 mL ammonia (7.0 M solution in MeOH) was added at room temperature under continuous stirring for 24 hours. LC-MS and TLC could be utilized to monitor the process of the reaction. The mixture was concentrated, K_2CO_3 (828.0 mg) was added and the residue was dissolved in 25 mL methanol stirring for 24 hours. This step is to remove the acetyl protecting group. The mixture then was concentrated again. Finally, the acetic acid (80 % in H_2O, 25 mL) was added at room temperature under continuous stirring. LC-MS could be utilized to monitor the process of the reaction. The mixture was concentrated and purified by the above mentioned SepaBean machine to afford the final product **7g** (905.4 mg, 53 %).

(2R,3S,5R)-5-(2-amino-6-oxo-1,6-dihydro-9H-purin-9-yl)-2-(hydroxymethyl)tetrahydrofuran-3-yl (((2R,3S,5R)-3-hydroxy-5-(5-methyl-2,4-dioxo-3,4-dihydropyrimidin-1(2H)-yl)tetrahydrofuran-2-yl)methyl) hydrogen phosphate (Compound 7g)

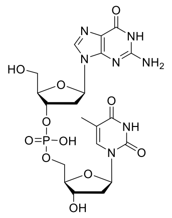

After purification by column chromatography (C18 Spherical silica) using MeOH/H_2O as the eluents, compound **7g** was obtained as a white solid (905.4 mg, 39 %). mp 178.7-182.4 °C. $[\alpha]_D^{25}$ 15.96 (c 0.0940, MeOH). This was followed by exchange of the counterions [Amberlite IR120 (Na^+)], in order to improve the solubility of the product in D_2O and simplify the NMR spectrum. ^1H NMR (400 MHz, D_2O) δ 7.94 (s, 1H), 7.55 (s, 1H), 6.24 (t, J = 6.8 Hz, 1H), 6.18-6.15 (m, 1H), 4.93-4.85 (m, 1H), 4.58 (q, J = 5.2 Hz, 1H), 4.26 (q, J = 3.6 Hz, 1H), 4.20-4.16 (m, 1H), 4.12-4.07 (m, 2H), 3.87-3.77 (m, 2H), 2.78-2.66 (m, 2H), 2.37-2.28 (m, 2H), 1.70 (s, 3H). Analytical data are consistent with those previously reported [52]. ^{13}C NMR (126 MHz, D_2O) δ 165.6, 158.4, 153.5, 151.3, 150.6, 137.6, 137.0, 116.4, 111.1, 86.1 (d, J_{C-P} = 8.3 Hz), 84.6, 84.6, 84.4, 75.5 (d, J_{C-P} = 5.3 Hz), 69.8, 64.4 (d, J_{C-P} = 5.3 Hz), 61.3, 38.5, 37.5, 11.3. ^{31}P NMR (202 MHz, D_2O) δ -1.04. HRMS-ESI m/z calcd. for $C_{20}H_{26}N_7NaO_{11}P$ $[M+Na]^+$ 594.1320. found 594.1317.

dTpG

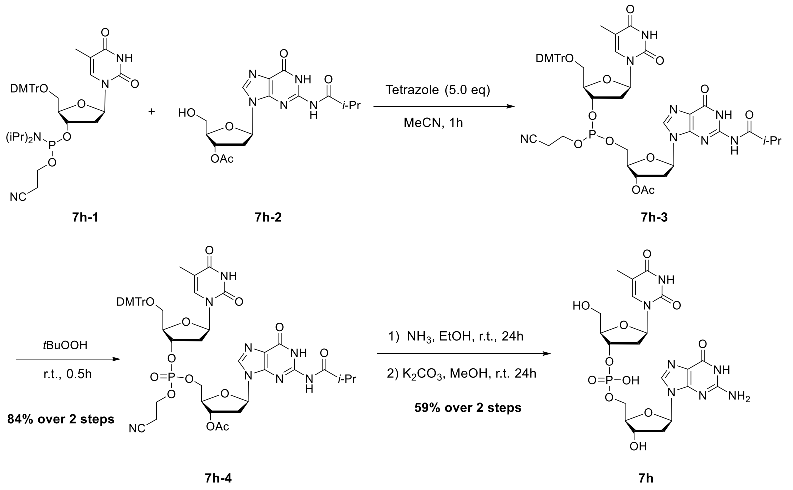

A flame-dried 50 mL Schlenk tube equipped with magnetic stir bar was charged with **7h-1** (2.23 g, 3.0 mmol, 1.0 equiv.), compound **7h-2** (1.14 g, 3.0 mmol, 1.0 equiv.), 1*H*-Tetrazole (840.0 mg, 12.0 mmol, 4.0 equiv.), and flame-dried 3 Å MS (powder, about 750 mg). Nitrogen was substituted for three times. The solids were suspended in anhydrous acetonitrile (25 mL, 0.12 M). TLC could be utilized to monitor the process of the reaction. After vigorous stirring at room temperature for about 1 hour, a solution of *tert*-butyl hydroperoxide (70 % in H_2O, 1.44 mL, 15 mmol, 5.0 equiv.) was added and the reaction mixture was stirred for about an additional 0.5 hour. The reaction mixture was filtered through a plug of Celite and the filter cake washed with dichloromethane. The filtrate was concentrated under reduced pressure. Then the crude material was purified by the SepaBean machine (Santai Technology Inc., China) equipped with the C18-bonded SepaFlash column to afford the desired intermediate **7h-4** (2.63 g, 84 %) as a white solid.

To a 100 mL round bottom flask equipped with magnetic stir bar, charged with compound **7h-4** (2.63 g, 2.53 mmol), and closed tightly with rubber septum and a balloon, 25 mL ammonia (7.0 M solution in MeOH) was added at room temperature under continuous stirring for 24 hours. LC-MS and TLC could be utilized to monitor the process of the reaction. The mixture was concentrated, K_2CO_3 (828.0 mg) was added and the residue was dissolved in 25 mL methanol stirring for 24 hours. This step is to remove the acetyl protecting group. The mixture then was concentrated again. Finally, the acetic acid (80 % in H_2O, 25 mL) was added at room temperature under continuous stirring. LC-MS could be utilized to monitor the process of the reaction. The mixture was concentrated and purified by the above mentioned SepaBean machine to afford the final product **7h** (1.02 g, 59 %).

((2R,3S,5R)-5-(2-amino-6-oxo-1,6-dihydro-9H-purin-9-yl)-3-hydroxytetrahydrofuran-2-

yl)methyl ((2R,3S,5R)-2-(hydroxymethyl)-5-(5-methyl-2,4-dioxo-3,4-dihydropyrimidin-1(2H)-yl)tetrahydrofuran-3-yl) hydrogen phosphate (Compound 7h)

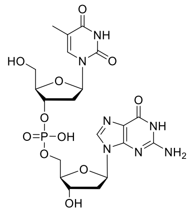

After purification by column chromatography (C18 Spherical silica) using MeOH/H_2O as the eluents, compound **7h** was obtained as a white solid (1.017 g, 50 %). mp 177.0-177.1 °C. $[\alpha]_D^{25}$ 20.00 (c 0.110, MeOH). This was followed by exchange of the counterions [Amberlite IR120 (Na^+)], in order to improve the solubility of the product in D_2O and simplify the NMR spectrum. ^1H NMR (400 MHz, D_2O) δ 8.10 (s, 1H), 7.41 (s, 1H), 6.16 (t, J = 6.7 Hz, 1H), 6.06-6.02 (m, 1H), 4.75-4.71 (m, 1H), 4.63-4.59 (m, 1H), 4.19-4.16 (m, 1H), 4.05-4.02 (m, 3H), 3.66-3.61 (m, 2H), 2.83-2.76 (m, 1H), 2.55-2.48 (m, 1H), 2.29-2.21 (m, 1H), 1.81 (s, 3H), 1.77-1.70 (m, 1H). ^{13}C NMR (126 MHz, D_2O) δ 166.0, 157.9, 153.8, 151.2, 137.0, 136.8, 115.0, 111.5, 85.8 (d, J_{C-P} = 7.8 Hz), 85.4 (d, J_{C-P} = 9.2 Hz), 84.8, 83.1, 75.8 (d, J_{C-P} = 5.6 Hz), 70.6, 64.8 (d, J_{C-P} = 5.3 Hz), 61.14, 38.33, 36.75, 11.54. ^{31}P NMR (202 MHz, D_2O) δ -1.09. HRMS-ESI m/z calcd. for $C_{20}H_{26}N_7NaO_{11}P$ $[M+Na]^+$ 594.1320. found 594.1311.

3.2.3. Preparation of cyclic dinucleotides c-di-GMP [36, 42-44]

c-di-GMP

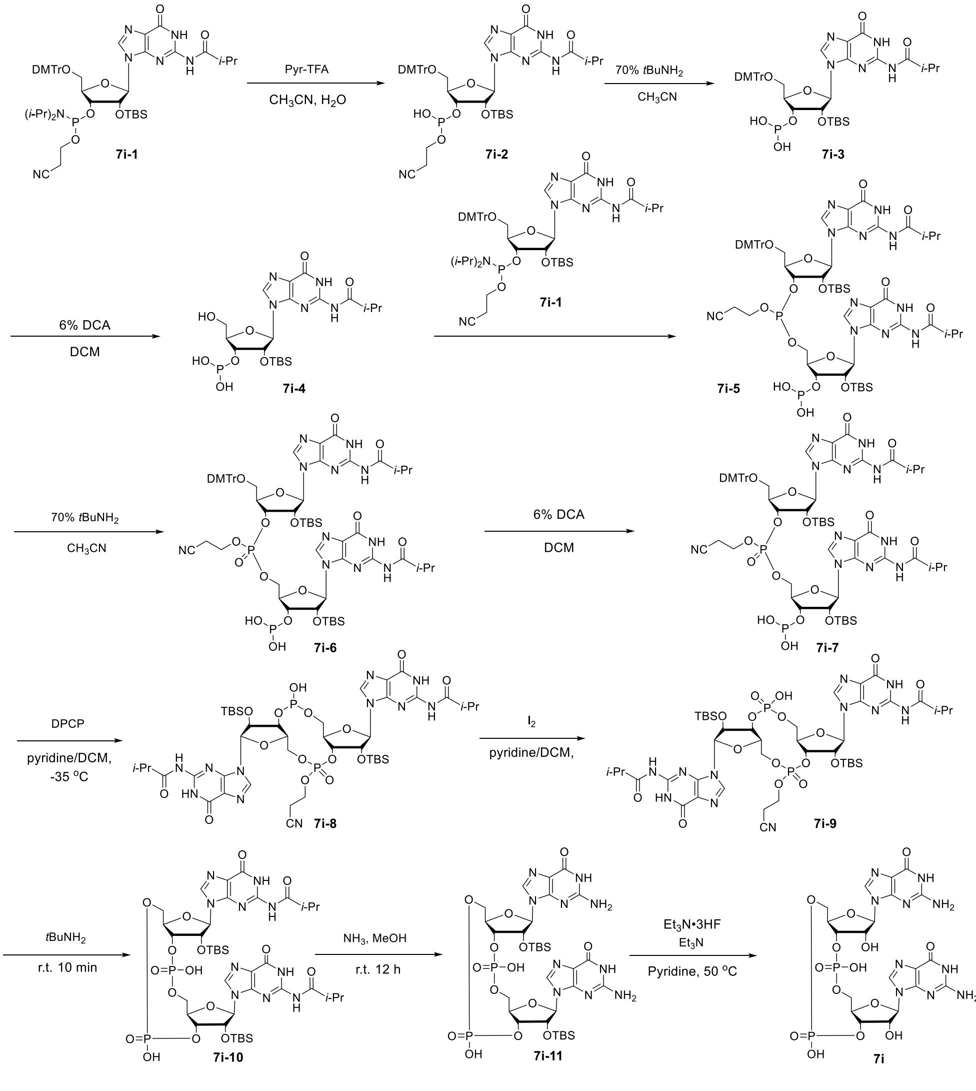

Preparation of a dry solution of 7i-1 in CH_3CN.

One portion of guanosine phosphoramidite, **7i-1** (1.75 g, 1.8 mmol, 1.2 equiv.), was dried three times by concentration from 12 mL portions of CH_3CN, the last time leaving 6 mL. The 3Å molecular sieves were added, getting the first portion of **7i-1**.

Hydrolysis, β-elimination, and detritylation.

To a second portion of **7i-1** (1.46 g, 1.5 mmol) dissolved in CH_3CN (7.5 mL) and H_2O (0.054 mL, 3.0 mmol, 2.0 equiv.) was added pyridinium trifluoroacetate (0.348 g, 1.8 mmol, 1.2 equiv.), getting **7i-2**. After 1 min a 7.5 mL portion of *t*-$BuNH_2$ was added. After 10 min the mixture was concentrated to a foam, getting **7i-3**, the residue was dissolved in a 15 mL portion of CH_3CN, and concentrated

again to a foam. This addition of CH_3CN and concentration was repeated one more time. To the residue dissolved in a 18 mL portion of CH_2Cl_2 was added H_2O (0.27 mL, 15 mmol, 10 equiv.), followed by a 18 mL portion of 6% dichloroacetic acid in CH_2Cl_2 (13.2 mmol). After 10 min the reaction was quenched by addition of pyridine (2.1 mL, 26.1 mmol, 2.0 equiv. rel to DCA), getting **7i-4**. The mixture was then concentrated, and the residue **7i-4** was dissolved in a 12 mL portion of dry CH_3CN and concentrated again. This process was repeated two more times, the last time leaving 3.6 mL.

Linear coupling, oxidation, and detritylation.

To the above solution was added the dried solution of **7i-1** (the first portion) using a Schlenk tube and nitrogen pressure, followed by two 1 mL rinses of dry CH_3CN, to get **7i-5**. After 2 minutes, anhydrous *tert*-butyl hydroperoxide (70 % in H_2O) (0.819 mL, 1.35 mmol, 3.0 equiv.) was added, to get the **7i-6**. After 30 min the solution was cooled in an ice bath, and 0.375 g $NaHSO_3$ dissolved in 0.75 mL H_2O was added. The ice bath was removed, the mixture was stirred 5 min, and then concentrated to a small volume. The residual oil was dissolved in an 24 mL portion of CH_2Cl_2, followed by H_2O (0.27 mL, 15 mmol, 10 equiv.) and then 24 mL 6% dichloroacetic acid in CH_2Cl_2 (17.4 mmol), to getting **7i-7**. After 10 min the reaction was quenched with a 15 mL portion of pyridine. The mixture was concentrated to a small volume, a 45 mL portion of pyridine was added, and the solution was concentrated to 25 mL.

Cyclization and oxidation.

To the above solution was added 50 mL DCM. The diphenyl chlorophosphate (DPCP, 17.5 mmol, 3.5 equiv.) was dissolved in in 25mL of pyridine. Cool the above two solution to -40 °C. The solution of DPCP was dropwise added to the solution of **7i-7** within 10 min. After 20 min, the reaction was quenched by addition of H_2O (3.6 mL), and I_2 (390.0 mg, 1.5 mmol) was added immediately, to get **7i-9**. After 5 min the mixture was poured into 25 mL H_2O containing 300 mg $NaHSO_3$. The mixture was filtered and concentrated. Then the crude material was purified by the SepaBean machine (Santai Technology Inc., China) equipped with the C18-bonded SepaFlash column to afford the desired intermediate **7i-9** as a white solid.

Remove protective groups.

To the above pure **7i-9** dissolved in a 100 mL round bottom flask equipped with magnetic stir bar, and closed tightly with rubber septum and a balloon, a 10 mL portion of *t*-$BuNH_2$ was added. After 10 min the mixture was concentrated to a foam, getting **7i-10**. a 10 mL ammonia (7.0 M solution in MeOH) was added at room temperature under continuous stirring for 12 hours, to get **7i-11**. LC-MS

and TLC could be utilized to monitor the process of the reaction. If the isobutyryl group (-CO*i*Pr) is not completely removed, concentrate the reaction solution, then add the 25 mL ammonia (7.0 M solution in MeOH) again. Finally, the mixture was concentrated and the triethylamine trihydrofluoride (2.5 mL), triethylamine (1.0 mL), and pyridine (1.0 mL) were added. Then the round bottom flask was placed in oil bath and heated at 50 °C overnight. The mixture was concentrated to a small volume and purified by the above mentioned SepaBean machine to afford the final product **7e** (48 mg, total yield = 3.2%).

9,9'-((2R,3R,3aS,7aR,9R,10R,10aS,14aR)-3,5,10,12-tetrahydroxy-5,12-dioxidooctahydro-2H,7H-difuro[3,2-d:3',2'-j][1,3,7,9]tetraoxa[2,8]diphosphacyclododecine-2,9-diyl)bis(2-amino-1,9-dihydro-6H-purin-6-one) (Compound 7i)

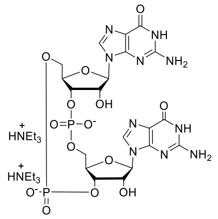

^{1}H NMR (400 MHz, D_2O) 55 ℃, δ 8.34 (s, 2H), 6.26 (s, 2H), 5.35 (br, 2H), 5.15 (br, 2H), 4.46-4.44 (m, 2H). Analytical data are consistent with those previously reported [35]. HRMS-ESI m/z calcd. for $C_{20}H_{23}N_{10}O_{14}P_2$ [M-H]$^-$ 689.0876. found 689.0876.

4. Reaction screening and proposed mechanism

4.1. Reaction setup for nucleosides and dinucleotides

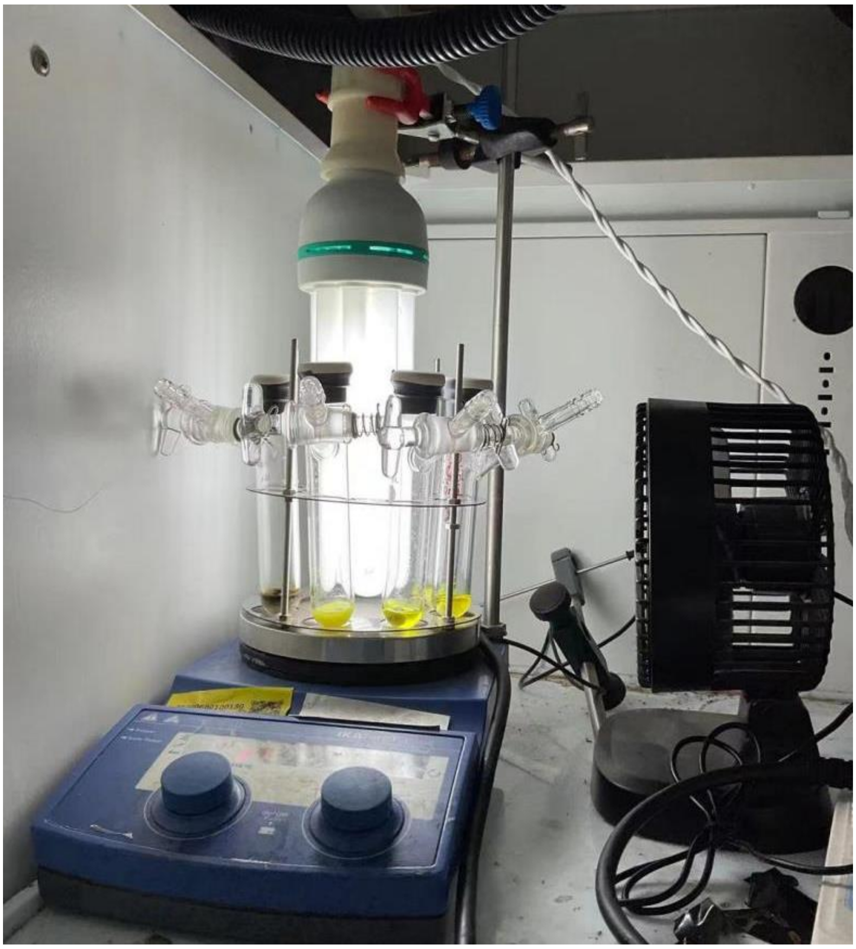

Supplementary Figure 15. Standard reaction setup for ***nucleosides***

(a) 10 mL Schlenk tube was used (LH LABWARE). The tube was plugged with a rubber plug and sealed with tape to maintain the satisfactory air tightness.

(b) The vial was irradiated with one 85W white light and was placed about 1-2 cm away from the light. Cooling fan was utilized to maintain the reaction temperature at room temperature.

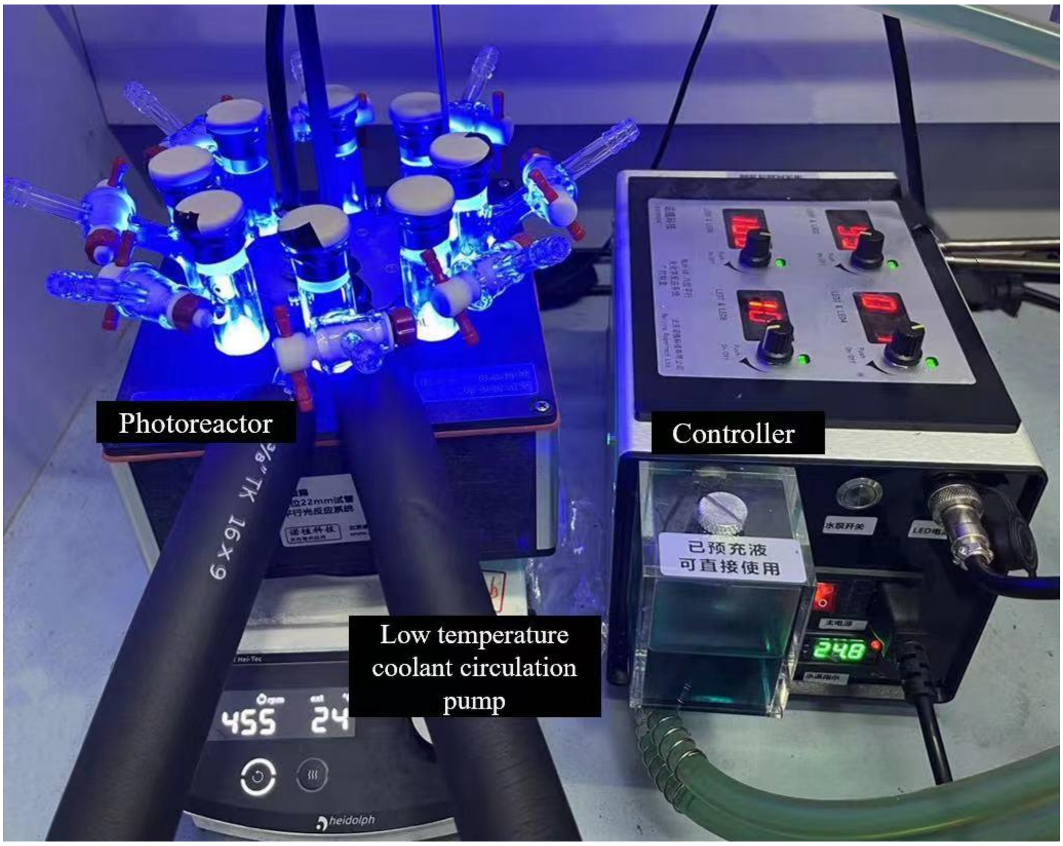

Supplementary Figure 16. Reaction setup of ***dinucleotides***

(a) 10 mL Schlenk tube was used (LH LABWARE). The tube was plugged with a rubber plug and sealed with tape to maintain the satisfactory air tightness.

(b) The vial was irradiated with one 10W blue LED (ROGER, Multi-channel parallel photocatalytic reaction system). Low temperature coolant circulation pump (BiLon) was utilized to maintain the reaction temperature at about 10 degrees Celsius.

4.2. Optimization of C–H alkylation of guanosine

4.2.1. Initial trial

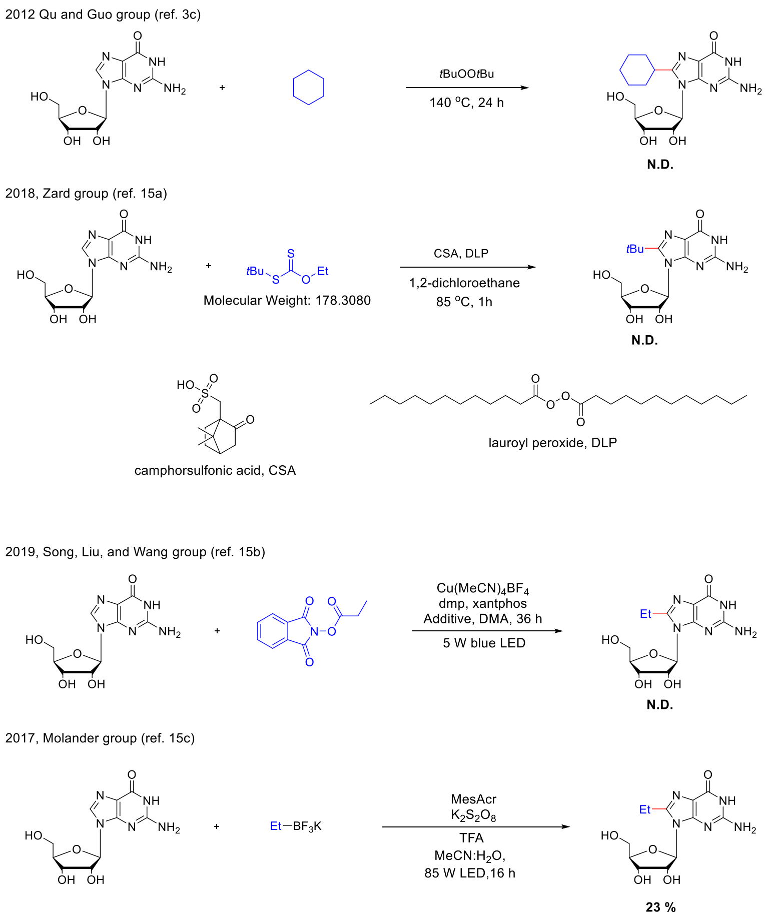

Supplementary Figure 17. Different approach of radical C-8 ethylation [6, 45-47]

4.2.2. Reaction condition screening

Supplementary Table 3. Photocatalyst screening [a,b]

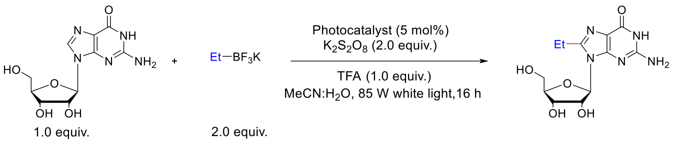

Entry	Photocatalyst	Yield of 3 (%)
1	MesAcr	23
2	$Ir[dF(CF_3)ppy]_2(dtbpy)PF_6$	12
3	$Ru(bpz)_3(PF_6)_2$	15
4	$Ru(DMB)_3(PF_6)_2$	14
5	Eosin Y	19

[a] Condition: Guanosine (0.1 mmol), ethylboric acids (0.4 mmol, 4.0 equiv.), photocatalyst (0.005 mmol, 0.05 equiv.), $(NH_4)_2S_2O_8$ (0.2 mmol, 2.0 equiv.), TFA (0.1 mmol, 1.0 equiv.), MeCN (0.5 mL), H_2O (0.5 mL), 85 W white light. [b] Yields were determined by LC-MS.

Supplementary Table 4. Solvent screening [a,b]

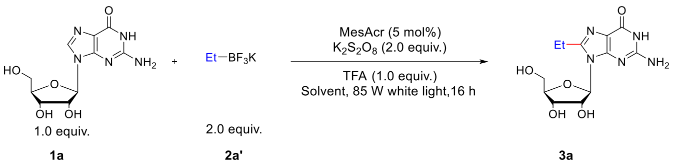

Entry	Solvent	Yield of 3 (%)
1	MeCN:H_2O(1:1)	22
2	DMSO:H_2O(1:1)	2
3	Acetone:H_2O(1:1)	6
4	THF:H_2O(1:1)	6

[a] Condition: Guanosine (0.1 mmol), ethylboric acids (0.4 mmol, 4.0 equiv.), MesAcr (0.005 mmol, 0.05 equiv.), $(NH_4)_2S_2O_8$ (0.2 mmol, 2.0 equiv.), solvent (1.0 mL), TFA (0.1 mmol, 1.0 equiv.), 85 W white light.

[b] Yields were determined by LC-MS.

Supplementary Table 5. Ethylboric acid equivalent screening [a,b]

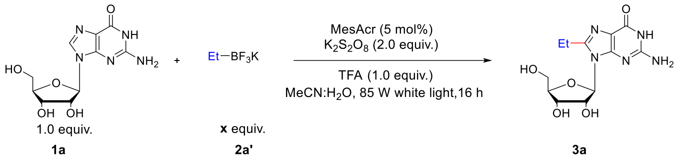

Entry	Equivalent of Et-BF_3K	Yield of 3 (%)
1	2	23
2	3	34
3	4	37
4	6	36

[a] Condition: Guanosine (0.1 mmol), ethylboric acid (x equiv.), MesAcr (0.005 mmol, 0.05 equiv.), $(NH_4)_2S_2O_8$ (0.2 mmol, 2.0 equiv.), MeCN (0.5 mL), H_2O (0.5 mL), TFA (0.1 mmol, 1.0 equiv.), 85 W white light. [b] Yields were determined by LC-MS.

Supplementary Table 6. Additive screening [a,b] [16]

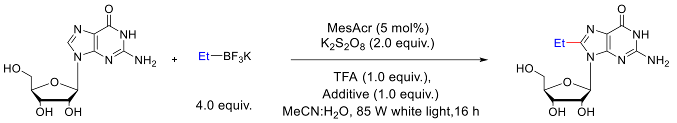

Entry	Additive	Yield of 3 (%)
1	none	37
2	$AgNO_3$	9
3	$PhI(TFA)_2$	0
4	Catechol (2.0 equiv.)	90
5	Catechol (1.0 equiv.)	90
6	Catechol (0.5 equiv.)	88
7 [c]	Catechol (1.0 equiv.)	92
8 [c]	$EtB(OH)_2$,Catechol (1.0 equiv.)	87
9 [c]	$EtB(OH)_2$	18

[a] Condition: Guanosine (0.1 mmol), ethylboric acids (0.4 mmol, 4.0 equiv.), MesAcr (0.005 mmol,0.05 equiv.), $(NH_4)_2S_2O_8$ (0.2 mmol, 2.0 equiv.), catechol (x equiv.), TFA (0.1 mmol, 1.0 equiv.), MeCN (0.5 mL), H_2O (0.5 mL), 85 W white light. [b] Yields were determined by LC-MS. [c] $(NH_4)_2S_2O_8$ is oxidant instead of $K_2S_2O_8$.

Supplementary Table 7. Catechol analogues screening [a,b] [48, 49]

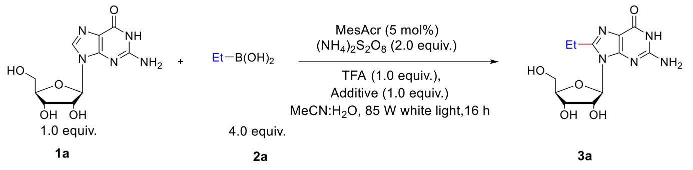

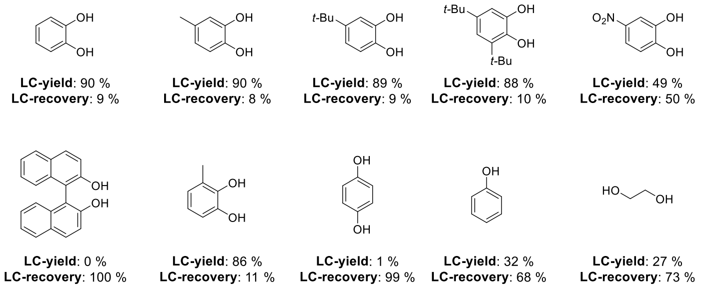

[a] Condition: Guanosine (0.1 mmol), ethylboric acids (0.4 mmol, 4.0 equiv.), MesAcr (0.005 mmol,0.05 equiv.), $(NH_4)_2S_2O_8$ (0.2 mmol, 2.0 equiv.), Additive (1.0 equiv.), TFA (0.1 mmol, 1.0 equiv.), MeCN (0.5 mL), H_2O (0.5 mL), 85 W white light. [b] Yields were determined by LC-MS.

Supplementary Table 8. Alkylpinacolyl boronate esters screening [a,b] [50]

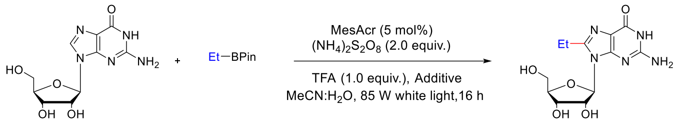

Entry	Additive	Yield of 3 (%)
1	TFA (1.0 equiv.), Catechol (1.0 equiv.)	49
2	$MeB(OH)_2$ (8.0 eq), TFA (3.0 equiv.), Catechol (1.0 equiv.)	81
3	$MeB(OH)_2$ (5.0 eq), TFA (3.0 equiv.), Catechol (1.0 equiv.)	73
4 [c]	$MeB(OH)_2$ (5.0 eq), TFA (1.0 equiv.), Catechol (1.0 equiv.)	73
5 [c]	$MeB(OH)_2$ (5.0 eq), TFA (3.0 equiv.), Catechol (1.0 equiv.)	75
6 [c]	$MeB(OH)_2$ (5.0 equiv.), TFA (3.0 equiv.)	0

[a] Condition: Guanosine (0.1 mmol), EtBPin (0.4 mmol, 4.0 equiv.), MesAcr (0.005 mmol, 0.05 equiv.), $(NH_4)_2S_2O_8$ (0.2 mmol, 2.0 equiv.), Catechol (1.0 equiv.), TFA (x equiv.), methylboronic acid (y equiv.), MeCN (0.5 mL), H_2O (0.5 mL), 85 W white light. [b] Yields were determined by LC-MS. [c] The equivalent of ethyl pinacol borate is 2.5 equiv.

4.3. Optimization of C–H alkylation of uridine

Supplementary Table 9. The amount and type of solvent. Type and equivalent of additives. The requirement of light and photocatalyst. [a,b]

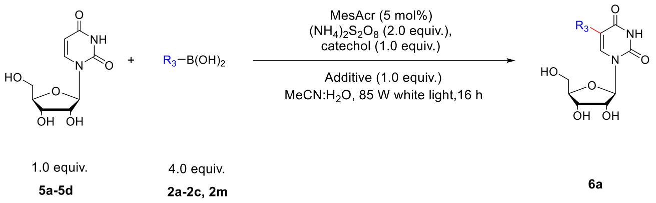

Entry	Solvent (mL/mL)	Additive	LC Yield %	LC recovery %
**1** [c]	MeCN:H_2O (0.5/0.5)	TFA	3	97
**2**	MeCN:H_2O (0.5/0.5)	TFA	12	88
**3**	DMA:H_2O (0.5/0.5)	TFA	4	96
**4**	DMF:H_2O (0.5/0.5)	TFA	7	93
**5**	DMSO:H_2O (0.5/0.5)	TFA	31	69
**6**	DMSO:H_2O (0.2/0.2)	TFA	42	58
**7**	DMSO:H_2O (0.2/0.2)	TFA	40	60
**8**	DMSO:H_2O (0.2/0.2)	-	40	60
**9**	DMSO:H_2O (0.2/0.2)	$MgCl_2$	42	58
**10**	DMSO:H_2O (0.2/0.2)	$AgNO_3$	40	60
**11**	DMSO:H_2O (0.2/0.2)	LiF	25	75
**12**	DMSO:H_2O (0.2/0.2)	Na_2CO_3	13	87
**13**	DMSO:H_2O (0.2/0.2)	K_2HPO_4	33	67
**14** [d]	DMSO:H_2O (0.2/0.2)	$MgCl_2$	47	46
**15** [e]	DMSO:H_2O (0.2/0.2)	$MgCl_2$	**45**	**52**
**16** [f]	DMSO:H_2O (0.2/0.2)	$MgCl_2$	20	80

[a] General condition: Uridine (1.0 equiv.), alkylboronic acid (4.0 equiv.), $(NH_4)_2S_2O_8$ (4.0 equiv.), $MgCl_2$ (2.0 equiv.) and catechol (1.0 equiv.) in DMSO: H_2O (1: 1, 0.1 M) on 0.2 mmol scale; irradiated by 36 W blue LED at r.t. for 24 h. [b] Yields were determined by LC-MS. [c] Irradiated by white 85 W light for 16h. [d] 2.0 equiv. $MgCl_2$ was used. [e] The reaction was carried out without MesAcr. [f] The reaction was carried out without MesAcr and light.

4.4. Optimization of C–H isopropylation of guanosine triphosphate and investigation of the pH value of reaction mixture in various conditions

Supplementary Table 10. Stability experiments of guanosine triphosphate under acidic conditions [a,b]

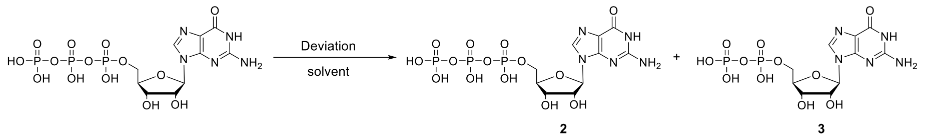

Entry	-R	Solvent	Deviation	Yield of 2 (%)[b]	Yield of 3 (%)[b]	pH value [c]	
						Before reaction	After reaction
1	H	MeCN:H_2O=1:1	TFA (3.0 equiv.)	84	16	2.5	1.0
2	H	H_2O	TFA (3.0 equiv.)	80	20	2.5	1.0

[a] Reaction condition: GTP (0.05 mmol, 1.0 equiv.) MeCN (0.25 mL), total volume of solvent (0.5 mL). [b] Yields were determined by HPLC. [c] pH values were determined by pH test paper.

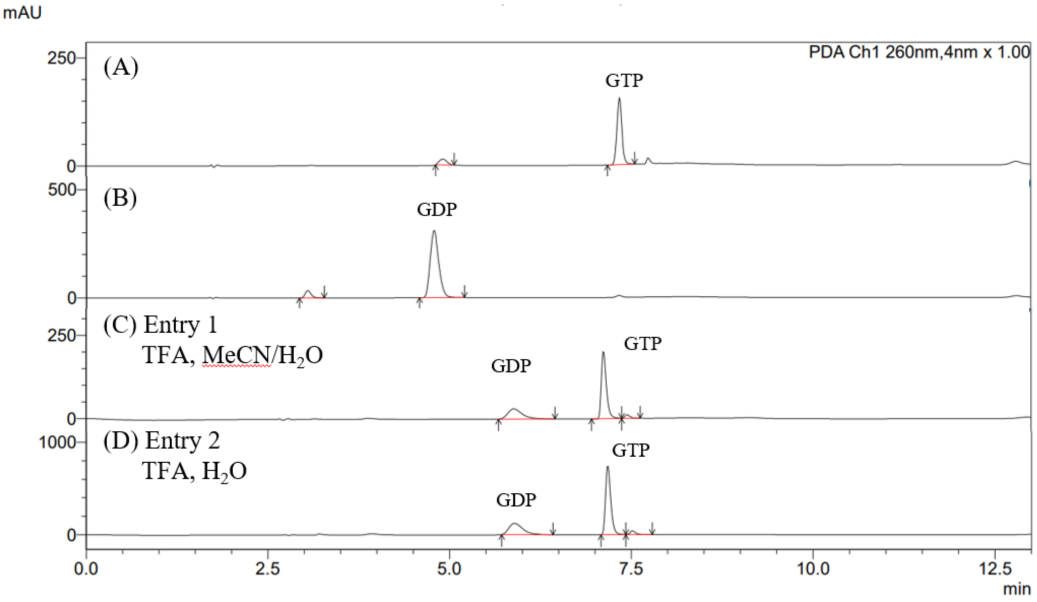

Supplementary Figure 18. Reverse-phase HPLC traces of (A) the standard sample of GTP, (B) the standard sample of GDP, (C) reaction mixture following the treatment of 3.0 equiv. TFA in MeCN/H_2O (specific conditions are shown in **Entry 1** in **Supplementary Table 10**), and (D) reaction mixture following the treatment of 3.0 equiv. TFA in H_2O (specific conditions are shown in **Entry 2** in **Supplementary Table 10**).

Supplementary Table 11. Buffer screening [a,b]

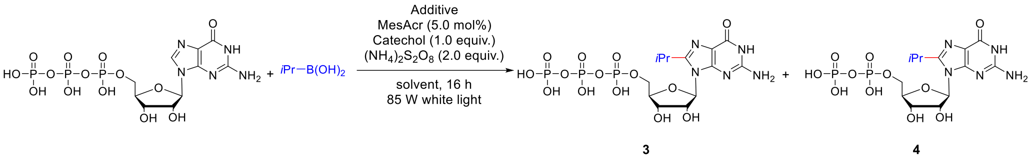

Entry	Solvent	Additive	Yield of 3 [b]	Yield of 4 [b]	pH value [c] (after reaction)
1	MeCN:H_2O=1:1	TFA (1 equiv.)	65%	27%	0.5
2	MeCN:H_2O=1:1	-	70%	24%	1.0
3	MeCN:10 mM Tris-HCl (pH7.0)=1:1	-	73%	17%	1.5
4	MeCN:10 mM PBS (pH7.2)=1:1	-	73%	20%	1.5
5	MeCN:10 mM NH_4HCO_3=1:1	-	71% (43% [d])	14%	1.5
6	MeCN:20 mM NH_4HCO_3=1:1	-	70%	14%	1.5

[a] Reaction condition: GTP (0.05 mmol, 1.0 equiv.), isopropylboronic acid (4.0 equiv.), MesAcr (0.0025 mmol,0.05 equiv.), $(NH_4)_2S_2O_8$ (0.1 mmol, 2.0 equiv.), catechol (0.05 mmol, 1.0 equiv.), MeCN (0.25 mL), aqueous phase (0.5 mL), irradiated by 85 W white light at r.t. for 16 h. [b] Yields were determined by HPLC. [c] pH values were determined by pH test paper. [d] Isolated yield.

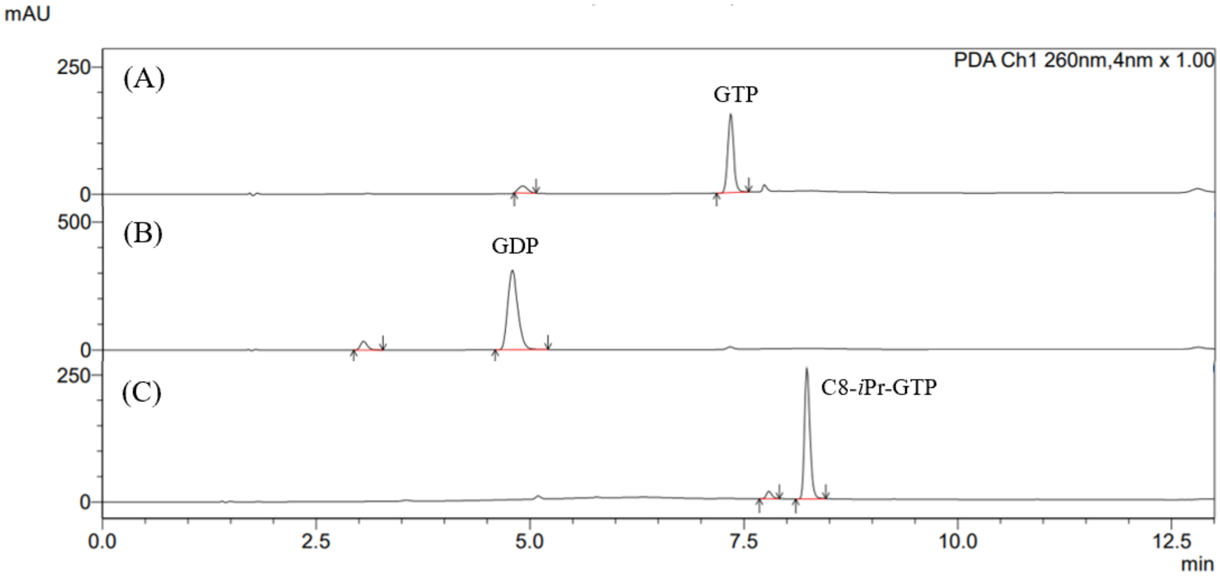

Supplementary Figure 19. Reverse-phase HPLC traces of (A) the standard sample of GTP, (B) the standard sample of GDP, and (C) the standard sample of C8-isopropylated GTP.

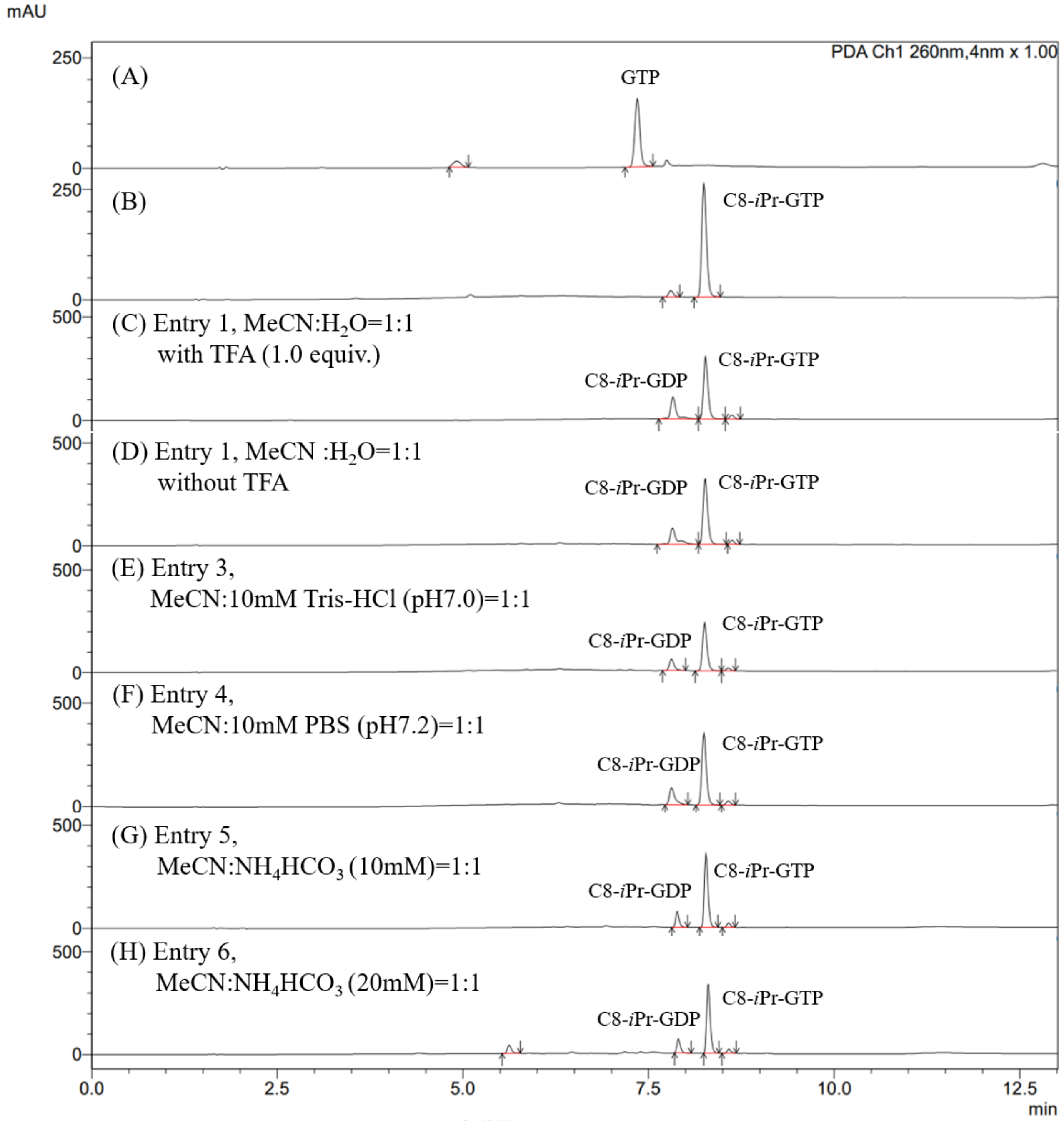

Supplementary Figure 20. Reverse-phase HPLC traces of (A) the standard sample of GTP, (B) the standard sample of C8-isopropylated GTP, (C) reaction mixture following catechol-promoted photoredox C–H alkylation of GTP with isopropyl boronic acid (specific conditions are shown in **Entry 1** in **Supplementary Table 11**), (D) reaction mixture following catechol-promoted photoredox C–H alkylation of GTP with isopropyl boronic acid (specific conditions are shown in **Entry 2** in **Supplementary Table 11**), (E) reaction mixture following catechol-promoted photoredox C–H alkylation of GTP with isopropyl boronic acid (specific conditions are shown in **Entry 3** in **Supplementary Table 11**), (F) reaction mixture following catechol-promoted photoredox C–H alkylation of GTP with isopropyl boronic acid (specific conditions are shown in **Entry 4** in **Supplementary Table 11**), (G) reaction

mixture following catechol-promoted photoredox C–H alkylation of GTP with isopropyl boronic acid (specific conditions are shown in **Entry 5** in **Supplementary Table 11**), and (H) reaction mixture following catechol-promoted photoredox C–H alkylation of GTP with isopropyl boronic acid (specific conditions are shown in **Entry 6** in **Supplementary Table 11**)

Supplementary Table 12. Investigation of the pH value of reaction mixture on various conditions.

(1) pH value of various reagents [a]

Entry	Solution	Reagents (0.1 M)	pH value [a]
1	MeCN/H_2O	None	6.85
2	MeCN/H_2O	*i*PrB(OH)$_2$	5.02
3	MeCN/H_2O	Catechol	5.59
4	MeCN/H_2O	$(NH_4)_2S_2O_8$	2.94
5	MeCN/H_2O	*i*PrB(OH)$_2$ & Catechol	3.77
6	MeCN/H_2O	*i*PrB(OH)$_2$, Catechol & $(NH_4)_2S_2O_8$	3.11
7	MeCN/10mM NH_4HCO_3	None	8.33
8	MeCN/10mM NH_4HCO_3	*i*PrB(OH)$_2$	8.33
9	MeCN/10mM NH_4HCO_3	Catechol	8.35
10	MeCN/10mM NH_4HCO_3	$(NH_4)_2S_2O_8$	7.58
11	MeCN/10mM NH_4HCO_3	*i*PrB(OH)$_2$ & Catechol	6.13
12	MeCN/10mM NH_4HCO_3	*i*PrB(OH)$_2$, Catechol & $(NH_4)_2S_2O_8$	5.79

[a] The measurement of pH value needs to be completed within 2 minutes, since prolonged exposure of solution to the air will result in a decrease in pH value.

(2) pH value of reactions using guanosine as starting material

Entry	TFA	pH value	
		Before reaction	After reaction
1	+	1.16	1.05

2	-	2.13	1.57

[a] General condition: Guanosine (1.0 equiv.), ethylboronic acid (4.0 equiv.), MesAcr (5 mol%), $(NH_4)_2S_2O_8$ (2.0 equiv.), TFA (1.0 equiv.) and catechol (1.0 equiv.) in MeCN: H_2O (1: 1, 0.1M); irradiated by 85 W white light at r.t. for 16 h.

(3) pH value of reactions using GTP as starting material

**Entry**	**Solution**	**pH value**	
		**Before reaction**	**After reaction**
1	MeCN/H_2O	3.21	1.97
2	MeCN/10mM NH_4HCO_3	4.53	2.72

[a] General condition: Guanosine-5'-triphosphate disodium salt (1.0 equiv.), isopropylboronic acid (4.0 equiv.), MesAcr (5 mol%), $(NH_4)_2S_2O_8$ (2.0 equiv.), and catechol (1.0 equiv.) in MeCN: H_2O (1: 1, 0.1M); irradiated by 85 W white light at r.t. for 16 h. [b] The measurement of pH value needs to be completed within 2 minutes, since prolonged exposure of solution to the air will result in a decrease in pH value.

4.5. Proposed mechanism

The phenethylboronic acid was chosen, as the products generated by the capture of ethylboronic acid by TEMPO are relatively difficult to separate. [48]

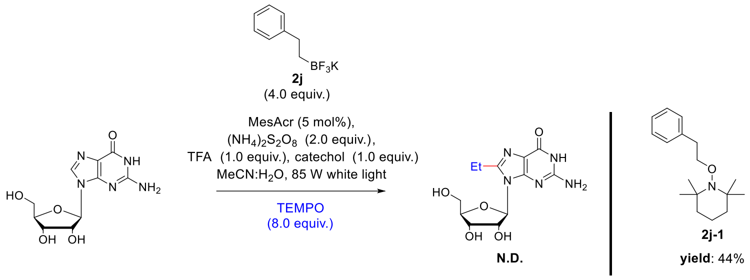

Supplementary Figure 21. Investigations of mechanism.

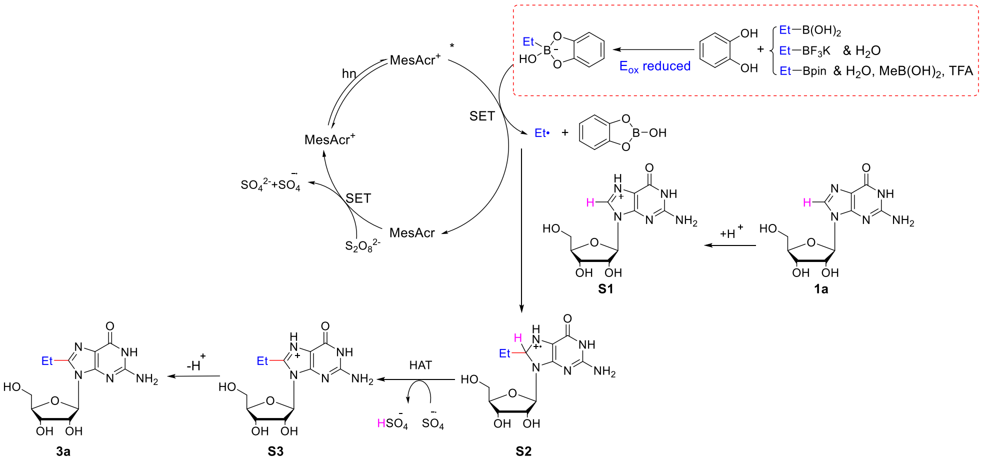

Supplementary Figure 22. Alkylation mechanism

5. Substrate scope study

5.1. Substrate scope of C–H alkylation of guanosine with diverse boron radical precursors

Supplementary Table 13. Substrate scope of alkylboronic acids and derivatives

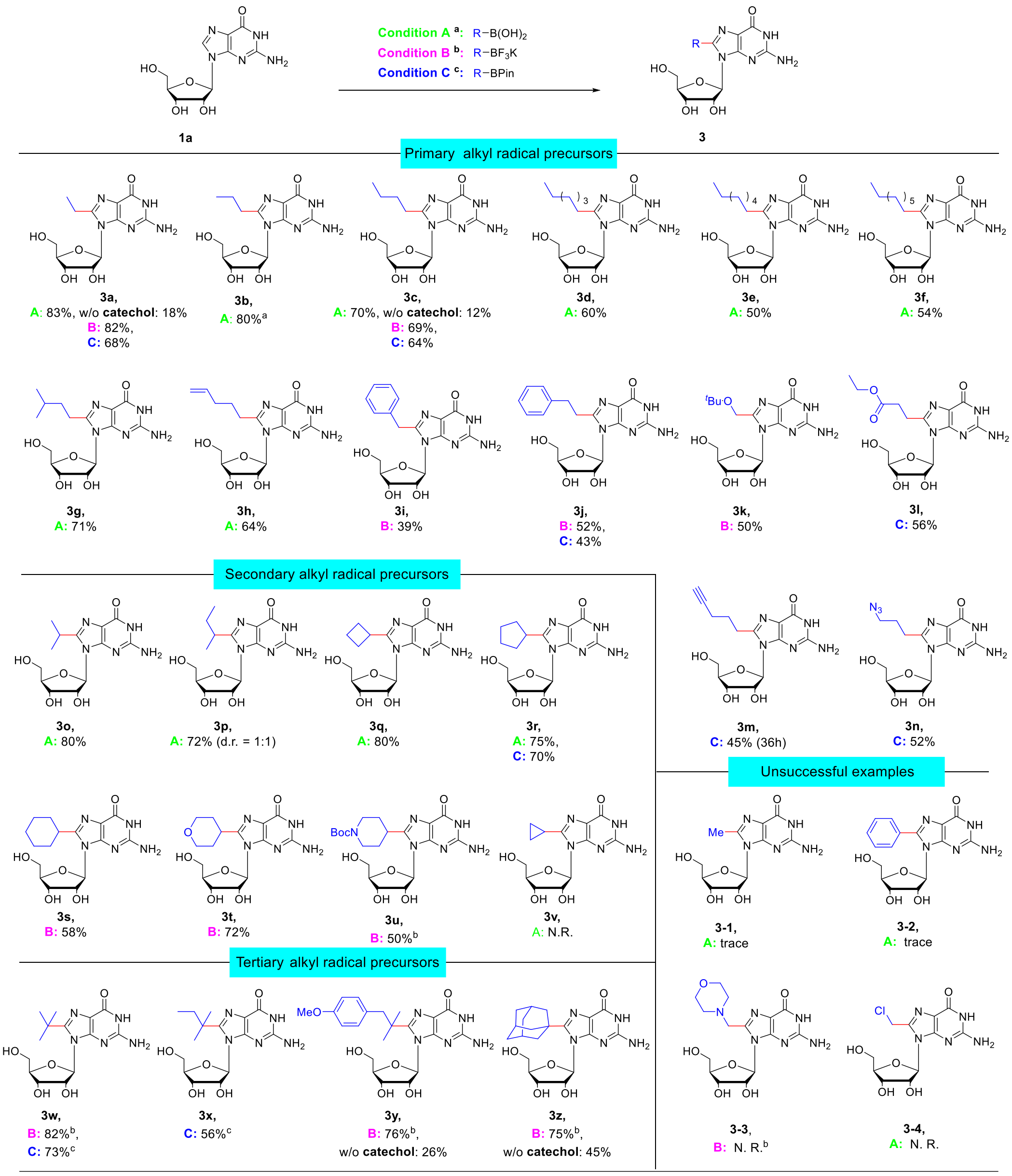

[a] Condition A: Guanosine (1.0 equiv.), alkylboronic acid (4.0 equiv.), MesAcr (5 mol%), $(NH_4)_2S_2O_8$ (2.0 equiv.), TFA (1.0 equiv.) and catechol (1.0 equiv.) in MeCN: H_2O (1: 1, 0.1 M) on 0.2 mmol scale; irradiated by 85 W white light at r.t. for 16 h; isolated yield. [b] Condition B: Alkyltrifluoroborate (2.0 equiv.) was used instead of alkylboronic acid. [c] Condition C: Alkylpinacolyl boronate esters (2.5 equiv.) was used instead of alkylboronic acid, adding methylboronic acid (5.0 equiv.).

General procedure of alkylation for the guanosine

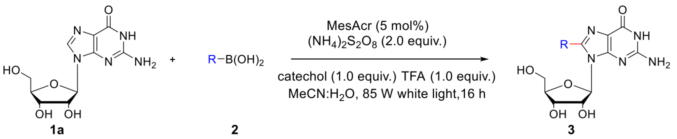

Method A General Procedure A (0.2 mmol scale): To a 10 mL Schlenk tube containing a Teflon stir bar was charged with nucleoside substrate (0.2 mmol, 1.0 equiv.), alkylboronic acid (0.8 mmol, 4.0 equiv.), MesAcr (0.01 mmol, 0.05 equiv.), $(NH_4)_2S_2O_8$ (0.4 mmol, 2.0 equiv.), catechol (0.2 mmol, 1.0 equiv.), 1.0 mL CH_3CN and 1.0 mL H_2O, and trifluoroacetic acid (0.2 mmol, 1.0 equiv.) sequentially. The Schlenk tube was sealed with a rubber plug and taped, utilizing the freeze-pump-thaw (FPT) method for deaeration. The reaction system was exposed to 85 W white light for **16 h**, monitoring the progress of the reaction by thin layer chromatography (TLC) (DCM/MeOH = 5/1 or 10/1) or the liquid chromatography-mass spectrometry (LC-MS). The mixture was concentrated under reduced pressure, and the crude product was purified by silica gel flash column chromatography (C18 Spherical silica) using MeOH/H_2O as eluents to give the pure product.

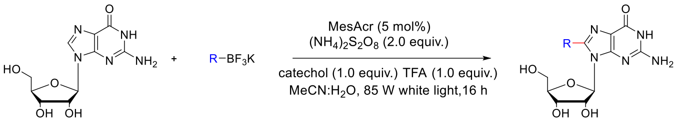

Method B General Procedure B (0.2 mmol scale): To a 10 mL Schlenk tube containing a Teflon stir bar was charged with nucleoside substrate (0.2 mmol, 1.0 equiv.), alkylboronic acid (0.4 mmol, 2.0 equiv.), MesAcr (0.01 mmol, 0.05 equiv.), $(NH_4)_2S_2O_8$ (0.4 mmol, 2.0 equiv.), 1.0 mL CH_3CN and 1.0 mL H_2O, and trifluoroacetic acid (0.1 mmol, 1.0 equiv.) sequentially. The Schlenk tube was sealed with a rubber plug and taped, utilizing the freeze-pump-thaw (FPT) method for deaeration. The reaction system was exposed to 85 W white light for **16 h**, monitoring the progress of the reaction by thin layer chromatography (TLC) (DCM/MeOH = 5/1) or the liquid chromatography-mass spectrometry (LC-MS). The mixture was concentrated under reduced pressure, and the crude product was purified by silica gel flash column chromatography (C18 Spherical silica) using MeOH/H_2O as eluents to give the pure product.

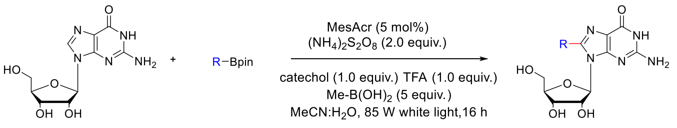

Method C General Procedure C (0.1 mmol scale): To a 10 mL Schlenk tube containing a Teflon stir bar was charged with nucleoside substrate (0.1 mmol, 1.0 equiv.), alkylpinacolyl boronate esters

(0.4 mmol, 4.0 equiv.), MesAcr (0.005 mmol, 0.05 equiv.), $(NH_4)_2S_2O_8$ (0.2 mmol, 2.0 equiv.), catechol (0.1 mmol, 1.0 equiv.), methylboronic acid (0.5 mmol, 5.0 equiv.), 0.5 mL CH_3CN and 0.5 mL H_2O, and trifluoroacetic acid (0.5 mmol, 5.0 equiv.) sequentially. The Schlenk tube was sealed with a rubber plug and taped, utilizing the freeze-pump-thaw (FPT) method for deaeration. The reaction system was exposed to 85 W white light for **16 h**, monitoring the progress of the reaction by thin layer chromatography (TLC) (DCM/MeOH = 5/1) or the liquid chromatography-mass spectrometry (LC-MS). The mixture was concentrated under reduced pressure, and the crude product was purified by silica gel flash column chromatography (C18 Spherical silica) using MeOH/H_2O as eluents to give the pure product.

Characterization data for compounds of alkylated guanosine

2-amino-9-((2R,3R,4S,5R)-3,4-dihydroxy-5-(hydroxymethyl)tetrahydrofuran-2-yl)-8-ethyl-1,9-dihydro-6H-purin-6-one (Compound 3a)

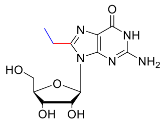

3a was obtained following the general procedure **A**. After purification by column chromatography (C18 Spherical silica) using MeOH/H_2O as the eluents, **3a** was obtained as a white solid (51.3 mg, 83%). mp 177.4-181.6 °C. $[\alpha]_D^{25}$ -49.35 (c 0.310, MeOH). ^{1}H NMR (400 MHz, DMSO-*d*$_6$) δ 10.61 (s, 1H), 6.27 (s, 2H), 5.63 (d, *J* = 6.8 Hz, 1H), 5.33 (d, *J* = 6.8 Hz, 1H), 5.15-5.12 (m, 1H), 5.08-5.07 (m, 1H), 4.76 (q, *J* = 6.4 Hz, 1H), 4.11-4.07 (m, 1H), 3.86 (q, *J* = 4.0 Hz, 1H), 3.66-3.61 (m, 1H), 3.55-3.49 (m, 1H), 2.75-2.69 (m, 2H), 1.22 (t, *J* = 7.6 Hz, 3H). ^{13}C NMR (101 MHz, DMSO-*d*$_6$) δ 156.4, 152.9, 151.8, 149.6, 115.6, 87.7, 85.7, 71.2, 70.6, 62.1, 20.8, 11.8. HRMS-ESI m/z calcd. for $C_{12}H_{18}N_5O_5$ $[M+H]^+$ 312.1308. found 312.1309.

2-amino-9-((2R,3R,4S,5R)-3,4-dihydroxy-5-(hydroxymethyl)tetrahydrofuran-2-yl)-8-propyl-1,9-dihydro-6H-purin-6-one (Compound 3b)

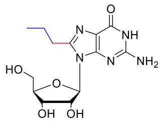

3b was obtained following the general procedure **A**. After purification by column chromatography (C18 Spherical silica) using MeOH/H_2O as the eluents, **3b** was obtained as a white solid (51.8 mg, 80%). mp 166.0-168.3 °C. $[\alpha]_D^{25}$ -49.64 (c 0.235, MeOH). ^{1}H NMR (400 MHz, DMSO-*d*$_6$) δ 10.64 (s, 1H), 6.29 (s, 2H), 5.63 (d, *J* = 7.2 Hz, 1H), 5.33 (d, *J* = 4.4 Hz, 1H), 5.16–5.14 (m, 1H), 5.09 (s, 1H), 4.80–4.79 (m, 1H), 4.10 (s, 1H), 3.88-3.85 (m, 1H), 3.66-3.63 (m, 1H), 3.56-3.50 (m, 5.2 Hz, 1H), 2.68 (t, *J* = 7.2 Hz, 2H), 1.74-1.65 (m, 2H), 0.95 (t, *J* = 7.2 Hz, 3H). ^{13}C NMR (101 MHz, DMSO-*d*$_6$) δ 156.4, 152.9, 151.5, 148.3, 115.7, 87.7, 85.7, 71.1, 70.6, 62.0, 29.2, 20.6, 13.8. HRMS-ESI m/z calcd. for $C_{13}H_{20}N_5O_5$ $[M+H]^+$ 326.1464. found 326.1456.

2-amino-8-butyl-9-((2R,3R,4S,5R)-3,4-dihydroxy-5-(hydroxymethyl)tetrahydrofuran-2-yl)-1,9-dihydro-6H-purin-6-one (Compound 3c)

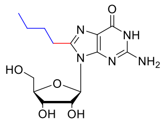

3c was obtained following the general procedure **A**. After purification by column chromatography (C18 Spherical silica) using MeOH/H_2O as the eluents, **3c** was obtained as a white solid (47.5 mg,

70%). mp 181.2-183.5 °C. $[\alpha]_D^{25}$ -42.48 (c 0.175, MeOH). ^{1}H NMR (400 MHz, DMSO-d_6) δ 10.61 (s, 1H), 6.26 (s, 2H), 5.63 (d, J = 6.8 Hz, 1H), 5.32 (d, J = 6.8 Hz, 1H), 5.15–5.12 (m, 1H), 5.08 (d, J = 4.8 Hz, 1H), 4.78 (q, J = 6.5 Hz, 1H), 4.10 (q, J = 4.0 Hz, 1H), 3.86(q, J = 4.0 Hz, 1H), 3.67-3.62 (m, 1H), 3.56-3.50 (m, 1H), 2.72-3.68 (m, 2H), 1.70-1.62 (m, 2H), 1.41-1.32 (m, 2H), 0.90 (t, J = 7.2 Hz, 3H). ^{13}C NMR (101 MHz, DMSO-d_6) δ 156.3, 152.8, 151.5, 148.4, 115.7, 87.7, 85.7, 71.1, 70.6, 62.0, 29.2, 26.9, 21.8, 13.7. HRMS-ESI m/z calcd. for $C_{14}H_{22}N_5O_5$ $[M+H]^+$ 340.1621. found 340.1615.

2-amino-9-((2R,3R,4S,5R)-3,4-dihydroxy-5-(hydroxymethyl)tetrahydrofuran-2-yl)-8-hexyl-1,9-dihydro-6H-purin-6-one (Compound 3d)

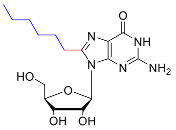

3d was obtained following the general procedure **A**. After purification by column chromatography (C18 Spherical silica) using MeOH/H_2O as the eluents, **3d** was obtained as a white solid (44.0 mg, 60%). mp 174.5-178.3 °C. $[\alpha]_D^{25}$ -13.54 (c 0.0517, MeOH). ^{1}H NMR (400 MHz, DMSO-d_6) δ 10.65 (s, 1H), 6.30 (s, 2H), 5.62 (d, J = 6.8 Hz, 1H), 5.33 (d, J = 6.4 Hz, 1H), 5.16-5.14 (m, 1H), 5.09 (d, J = 4.8 Hz, 1H), 4.80-4.76 (m, 1H), 4.11-4.08 (m, 1H), 3.86 (q, J = 4.0 Hz, 1H), 3.67-3.62 (m, 1H), 3.55-3.49 (m, 1H), 2.71-2.67 (m, 2H), 1.71-1.63 (m, 2H), 1.36-1.23 (m, 6H), 0.87 (t, J = 6.8 Hz, 3H). ^{13}C NMR (101 MHz, DMSO-d_6) δ 156.3, 152.8, 151.5, 148.4, 115.7, 87.7, 85.7, 71.1, 70.6, 62.0, 31.0, 28.3, 27.2, 27.0, 22.1, 14.0. HRMS-ESI m/z calcd for $C_{16}H_{26}N_5O_5$ $[M+H]^+$ 368.1934. found 368.1924.

2-amino-9-((2R,3R,4S,5R)-3,4-dihydroxy-5-(hydroxymethyl)tetrahydrofuran-2-yl)-8-heptyl-1,9-dihydro-6H-purin-6-one (Compound 3e)

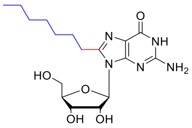

3e was obtained following the general procedure **A**. After purification by column chromatography (C18 Spherical silica) using MeOH/H_2O as the eluents, **3e** was obtained as a white solid (38.0 mg, 50%). mp 135.4-139.1 °C. $[\alpha]_D^{25}$ -13.01 (c 0.123, MeOH). ^{1}H NMR (400 MHz, DMSO-d_6) δ 10.63 (s, 1H), 6.28 (s, 2H), 5.62 (d, J = 6.8 Hz, 1H), 5.32 (d, J = 6.8 Hz, 1H), 5.15-5.12 (m, 1H), 5.09 (d, J = 4.8 Hz, 1H), 4.80-4.76 (m, 1H), 4.11-4.08 (m, 1H), 3.88-3.85 (m, 1H), 3.66-3.62 (m, 1H), 3.55-3.49 (m, 1H), 2.71-3.67 (m, 2H), 1.71-1.64 (m, 2H), 1.34-1.23 (m, 8H), 0.86 (t, J = 7.2 Hz, 3H). ^{13}C NMR (101 MHz, DMSO-d_6) δ 156.3, 152.8, 151.5, 148.4, 115.7, 87.7, 85.7, 71.1, 70.6, 62.0, 31.2, 28.6, 28.5, 27.2, 27.0, 22.1, 14.0. HRMS-ESI m/z calcd for $C_{17}H_{28}N_5O_5$ $[M+H]^+$ 382.2090. found 382.2090.

2-amino-9-((2R,3R,4S,5R)-3,4-dihydroxy-5-(hydroxymethyl)tetrahydrofuran-2-yl)-8-octyl-1,9-dihydro-6H-purin-6-one (Compound 3f)

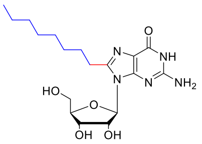

3f was obtained following the general procedure **A.** After purification by column chromatography (C18 Spherical silica) using MeOH/H_2O as the eluents, **3f** was obtained as a white solid (42.7 mg, 54%). mp 138.7-141.9 °C. $[\alpha]_D^{25}$ -40.97 (c 0.240, MeOH). ^1H NMR (400 MHz, DMSO-d_6) δ 10.63 (s, 1H), 6.26 (s, 2H), 5.63 (d, J = 6.8 Hz, 1H), 5.32 (d, J = 6.8 Hz, 1H), 5.16-5.13 (m, 1H), 5.08 (d, J = 4.4 Hz, 1H), 4.80-4.76 (m, 1H), 4.11-4.08 (m, 1H), 3.88-3.85 (m, 1H), 3.67-3.62 (m, 1H), 3.55-3.50 (m, 1H), 2.71-2.67 (m, 2H), 1.70-1.63 (m, 2H), 1.33-1.25 (m, 10H), 0.87-0.84 (m, 3H). ^{13}C NMR (101 MHz, DMSO-d_6) δ 156.4, 152.8, 151.6, 148.5, 115.7, 87.7, 85.7, 71.1, 70.6, 62.1, 31.3, 28.8, 28.7, 28.7, 27.2, 27.1, 22.2, 14.0. HRMS-ESI m/z calcd for $C_{18}H_{29}N_5NaO_5$ $[M+Na]^+$ 418.2061. found 418.2068.

2-amino-9-((2R,3R,4S,5R)-3,4-dihydroxy-5-(hydroxymethyl)tetrahydrofuran-2-yl)-8-isopentyl-1,9-dihydro-6H-purin-6-one (Compound 3g)

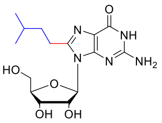

3g was obtained following the general procedure **A.** After purification by column chromatography (C18 Spherical silica) using MeOH/H_2O as the eluents, **3g** was obtained as a white solid (50.2 mg, 71%). mp 175.1-178.9 °C. $[\alpha]_D^{25}$ -44.08 (c 0.245, MeOH). ^1H NMR (400 MHz, DMSO-d_6) δ 10.63 (s, 1H), 6.27 (s, 2H), 5.64 (d, J = 6.8 Hz, 1H), 5.33 (d, J = 4.8 Hz, 1H), 5.15-5.12 (m, 1H), 5.08 (s, 1H), 4.78 (d, J = 5.2 Hz, 1H), 4.10 (s, 1H), 3.88-3.86 (m, 1H), 3.67-3.64 (m, 1H), 3.56-3.50 (m, 1H), 2.73-2.64 (m, 2H), 1.64-1.56 (m, 3H), 0.91 (d, J = 6.4 Hz, 6H). ^{13}C NMR (101 MHz, DMSO-d_6) δ 156.3, 152.8, 151.6, 148.5, 115.6, 87.7, 85.7, 71.1, 70.6, 62.0, 36.0, 27.1, 25.2, 22.3, 22.3. HRMS-ESI m/z calcd for $C_{15}H_{23}N_5NaO_5$ $[M+Na]^+$ 376.1591. found 376.1597.

2-amino-9-((2R,3R,4S,5R)-3,4-dihydroxy-5-(hydroxymethyl)tetrahydrofuran-2-yl)-8-(pent-4-en-1-yl)-1,9-dihydro-6H-purin-6-one (Compound 3h)

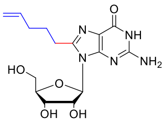

3h was obtained following the general procedure **A**. After purification by column chromatography (C18 Spherical silica) using MeOH/H_2O as the eluents, **3h** was obtained as a white solid (44.9 mg,

64%). mp 87.0-89.1 °C. $[\alpha]_D^{25}$ -8.00 (c 0.0500, MeOH). ^{1}H NMR (400 MHz, DMSO-d_6) δ 10.62 (s, 1H), 6.28 (d, J = 7.2 Hz, 2H), 5.88-5.81 (m, 1H), 5.63 (d, J = 6.8 Hz, 1H), 5.34-5.31 (m, 1H), 5.13-5.10 (m, 1H), 5.08-4.98 (m, 3H), 4.80-4.75 (m, 1H), 4.11-4.08 (m, 1H), 3.88-3.85 (m, 1H), 3.67-3.62 (m, 1H), 3.55-3.49 (m, 1H), 2.73-2.68 (m, 2H), 2.15-2.09 (m, 2H), 1.82-1.74 (m, 2H). ^{13}C NMR (101 MHz, DMSO-d_6) δ 156.3, 152.9, 151.6, 148.2, 138.2, 115.7, 115.2, 87.7, 85.7, 71.1, 70.6, 62.0, 32.6, 26.6, 26.2. HRMS-ESI m/z calcd. for $C_{15}H_{22}N_5O_5$ $[M+H]^+$ 352.1621. found 352.1621.

3-amino-8-benzyl-9-((2R,3R,4S,5R)-3,4-dihydroxy-5-(hydroxymethyl)tetrahydrofuran-2-yl)-1,9-dihydro-6H-purin-6-one (Compound 3i)

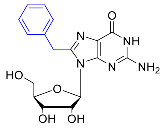

3i was obtained following the general procedure **A**. After purification by column chromatography (C18 Spherical silica) using MeOH/H_2O as the eluents, **3i** was obtained as a white solid (29.1 mg, 39%). mp 166.4-168.9 °C. $[\alpha]_D^{25}$ -64.98 (c 0.375, MeOH). ^{1}H NMR (400 MHz, DMSO-d_6) δ 10.68 (s, 1H), 7.32-7.28 (m, 2H), 7.24-7.20 (m, 3H), 6.31 (s, 2H), 5.70 (d, J = 6.8 Hz, 1H), 5.26 (d, J = 6.8 Hz, 1H), 5.19-5.16 (m, 1H), 5.07 (d, J = 4.8 Hz, 1H), 4.70-4.65 (m, 1H), 4.15 (s, 2H), 4.11-4.08 (m, 1H), 3.85-3.82 (m, 1H), 3.68-3.62 (m, 1H), 3.56-3.50 (m, 1H). ^{13}C NMR (101 MHz, DMSO-d_6) δ 156.3, 153.0, 151.6, 146.8, 137.1, 128.6, 128.4, 126.4, 116.0, 88.0, 85.7, 71.6, 70.4, 61.9, 33.2. HRMS-ESI m/z calcd for $C_{17}H1_9N_5NaO_5$ $[M+Na]^+$ 396.1278. found 396.1288.

2-amino-9-((2R,3R,4S,5R)-3,4-dihydroxy-5-(hydroxymethyl)tetrahydrofuran-2-yl)-8-phenethyl-1,9-dihydro-6H-purin-6-one (Compound 3j)

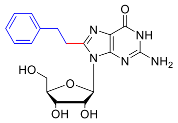

3j was obtained following the general procedure **A**. After purification by column chromatography (C18 Spherical silica) using MeOH/H_2O as the eluents, **3j** was obtained as a white solid (40.2 mg, 52%). mp >280 °C. $[\alpha]_D^{25}$ -25.37 (c 0.0513, MeOH：H_2O=1:1). ^{1}H NMR (400 MHz, DMSO-d_6) δ 10.64 (s, 1H), 7.29 (d, J = 4.4 Hz, 4H), 7.23-7.16 (m, 1H), 6.30 (s, 2H), 5.71 (d, J = 6.8 Hz, 1H), 5.34 (d, J = 6.8 Hz, 1H), 5.16-5.10 (m, 2H), 4.81-4.76 (m, 1H), 4.11-4.08 (m, 1H), 3.90-3.87 (m, 1H), 3.68-3.62 (m, 1H), 3.56-3.50 (m, 1H), 3.09-3.01 (m, 4H). ^{13}C NMR (101 MHz, DMSO-d_6) δ 156.3, 152.9, 151.7, 147.6, 141.1, 128.3, 126.0, 115.7, 87.6, 85.7, 71.2, 70.5, 61.9, 32.7, 29.1. HRMS-ESI m/z calcd for $C_{18}H_{21}N_5NaO_5$ $[M+Na]^+$ 410.1435. found 410.1437.

2-amino-8-(tert-butoxymethyl)-9-((2R,3R,4S,5R)-3,4-dihydroxy-5-(hydroxymethyl)tetrahydrofuran-2-yl)-1,9-dihydro-6H-purin-6-one (Compound 3k)

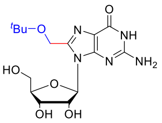

3k was obtained following the general procedure **A**. After purification by column chromatography (C18 Spherical silica) using MeOH/H_2O as the eluents, **3k** was obtained as a white solid (36.9 mg, 50%). mp 198.5-201.2 °C. $[\alpha]_D^{25}$ -22.55 (c 0.235, MeOH). ^{1}H NMR (400 MHz, DMSO-d_6) δ 10.77 (s, 1H), 6.38 (s, 2H), 5.79 (d, J = 6.4 Hz, 1H), 5.25-4.85 (m, 3H), 4.76 (t, J = 6.0 Hz, 1H), 4.55-4.36 (m, 2H), 4.16-4.14 (m, 1H), 3.86-3.83 (m, 1H), 3.69-3.65 (m, 1H), 3.55-3.51 (m, 1H), 1.20 (s, 9H). ^{13}C NMR (101 MHz, DMSO-d_6) δ 156.8, 153.4, 151.8, 145.5, 115.9, 88.5, 85.4, 73.9, 71.6, 70.4, 62.0, 57.2, 27.3. HRMS-ESI m/z calcd for $C_{15}H_{23}N_5NaO_6$ $[M+Na]^+$ 392.1541. found 392.1547.

ethyl 3-(2-amino-9-((2R,3R,4S,5R)-3,4-dihydroxy-5-(hydroxymethyl)tetrahydrofuran-2-yl)-6-oxo-6,9-dihydro-1H-purin-8-yl)propanoate (Compound 3l)

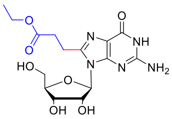

3l was obtained following the general procedure **C** (0.2 mmol). After purification by column chromatography (C18 Spherical silica) using MeOH/H_2O as the eluents, **3l** was obtained as a white solid (43 mg, 56%). mp 119.3-119.9 °C. $[\alpha]_D^{25}$ -37.000 (c 0.1, MeOH). ^{1}H NMR (400 MHz, DMSO) δ 10.60 (s, 1H), 6.29 (s, 2H), 5.67 (d, J = 6.8 Hz, 1H), 5.34 (d, J = 6.6 Hz, 1H), 5.11-5.08 (m, 2H), 4.75 (q, J = 6.7 Hz, 1H), 4.07 (q, J = 7.2 Hz, 3H), 3.89-3.83 (m, 1H), 3.67-3.62 (m, 1H), 3.55-3.50 (m, 1H), 2.98 (q, J = 7.0, 6.6 Hz, 2H), 2.77 (t, J = 7.0 Hz, 2H), 1.19 (t, J = 7.1 Hz, 3H). ^{13}C NMR (101 MHz, DMSO) δ 172.0, 156.3, 152.9, 151.9, 146.9, 115.5, 87.5, 85.6, 71.2, 70.5, 61.9, 60.0, 30.5, 22.6, 14.1. HRMS-ESI m/z calcd for $C_{13}H_{18}N_8NaO_5$ $[M+Na]^+$ 389.1295. found 389.1292.

2-amino-9-((2R,3R,4S,5R)-3,4-dihydroxy-5-(hydroxymethyl)tetrahydrofuran-2-yl)-8-(pent-4-yn-1-yl)-1,9-dihydro-6H-purin-6-one (Compound 3m)

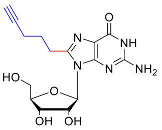

3m was obtained following the general procedure **C**, **while the reaction was irradiated for 36 hours**. After purification by column chromatography (C18 Spherical silica) using MeOH/H_2O as the eluents, **3m** was obtained as a white solid (7.8 mg, 45%). mp 239.6-240.6 °C. $[\alpha]_D^{25}$ -31.62 (c 0.0917, MeOH). ^{1}H NMR (400 MHz, DMSO-d_6) δ 10.63 (s, 1H), 6.30 (s, 2H), 5.64 (d, J = 6.7 Hz, 1H), 5.36 (d, J = 6.5 Hz, 1H), 5.17-5.11 (m, 1H), 5.09 (d, J = 4.7 Hz, 1H), 4.77 (q, J = 6.3 Hz, 1H), 4.09 (q, J = 4.2 Hz, 1H), 3.86 (q, J = 3.9 Hz, 1H), 3.67-3.61 (m, 4.6 Hz, 1H), 3.56-4.50 (m, 5.6 Hz, 1H), 2.30-2.26 (m, 2.4 Hz, 2H), 1.87 (p, J = 7.4 Hz, 2H). ^{13}C NMR (126 MHz, DMSO-d_6) δ 156.3, 152.9, 151.7,

147.7, 115.7, 87.6, 85.7, 84.2, 71.7, 71.1, 70.6, 62.0, 26.2, 26.0, 17.4. HRMS-ESI m/z calcd. for $C_{15}H_{19}N_5NaO_5$ $[M+Na]^+$ 372.1278. found 372.1285.

2-amino-8-(3-azidopropyl)-9-((2R,3R,4S,5R)-3,4-dihydroxy-5-(hydroxymethyl)tetrahydrofuran-2-yl)-1,9-dihydro-6H-purin-6-one (Compound 3n)

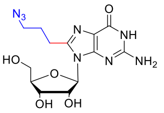

3n was obtained following the general procedure **C**. After purification by column chromatography (C18 Spherical silica) using MeOH/H_2O as the eluents, **3n** was obtained as a white solid (19 mg, 52%). mp 139.0-139.8 °C. $[\alpha]_D^{25}$ -43.000 (c 0.1, MeOH). ^1H NMR (400 MHz, DMSO-d_6) δ 10.66 (s, 1H), 6.32 (s, 2H), 5.65 (d, J = 6.8 Hz, 1H), 5.34 (s, 1H), 5.14-5.08 (m, 2H), 4.77-4.74 (m, 1H), 4.11-4.08 (m, 1H), 3.91-3.83 (m, 1H), 3.67-3.62 (m, 1H), 3.56-3.50 (m, 1H), 3.46 (t, J = 6.8 Hz, 2H), 2.86-2.73 (m, 2H), 1.96 (p, J = 7.1 Hz, 2H). ^{13}C NMR (126 MHz, DMSO-d_6) δ 156.4, 153.0, 151.8, 147.4, 115.7, 87.5, 85.7, 71.1, 70.5, 61.9, 50.1, 26.1, 24.4. HRMS-ESI m/z calcd for $C_{15}H_{21}N_5NaO_7$ $[M+Na]^+$ 406.1336. found 406.1333.

2-amino-9-((2R,3R,4S,5R)-3,4-dihydroxy-5-(hydroxymethyl)tetrahydrofuran-2-yl)-8-isopropyl-1,9-dihydro-6H-purin-6-one (Compound 3o)

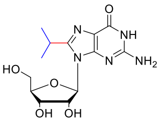

3o was obtained following the general procedure **A**. After purification by column chromatography (C18 Spherical silica) using MeOH/H_2O as the eluents, **3o** was obtained as a white solid (52.1 mg, 80%). mp 185.7-187.1 °C. $[\alpha]_D^{25}$ -36.52 (c 0.345, MeOH). ^1H NMR (400 MHz, DMSO-d_6) δ 10.76 (s, 1H), 6.37 (s, 2H), 5.64 (d, J = 6.8 Hz, 1H), 5.39-5.37 (m, 1H), 5.19-5.16 (m, 1H), 5.11 (s, 1H), 4.90-4.87 (m, 1H), 4.13-4.11 (m, 1H), 3.90-3.87 (m, 1H), 3.68-3.63 (m, 1H), 3.55-3.47 (m, 1H), 3.15-3.08 (m, 1H), 1.24 (d, J = 6.8 Hz, 6H). ^{13}C NMR (101 MHz, DMSO-d_6) δ 156.4, 153.2, 152.9, 151.4, 115.7, 87.8, 85.9, 71.0, 70.8, 62.2, 26.0, 21.8, 21.5. HRMS-ESI m/z calcd. for $C_{13}H1_9N_5NaO_5$ $[M+Na]^+$ 348.1278. found 348.1285.

2-amino-9-((2R,3R,4S,5R)-3,4-dihydroxy-5-(hydroxymethyl)tetrahydrofuran-2-yl)-8-isopentyl-1,9-dihydro-6H-purin-6-one (Compound 3p)

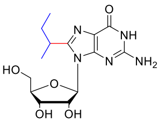

3p was obtained following the general procedure **A**. After purification by column chromatography (C18 Spherical silica) using MeOH/H_2O as the eluents, **3p** was obtained as a white solid (48.8 mg,

72%). mp 246.5.7-247.1 °C. $[\alpha]_D^{25}$ -16.28 (c 0.0430, MeOH). ^{1}H NMR (400 MHz, DMSO-d_6) δ 10.68 (s, 1H), 6.28 (d, J = 10.0 Hz, 2H), 5.64 (t, J = 6.8 Hz, 1H), 5.34 (s, 1H), 5.21-5.07 (m, 2H), 4.85 (s, 1H), 4.11 (s, 1H), 3.98 (s, 1H), 3.65 (s, 1H), 3.54 (s, 1H), 2.95-2.89 (m, 1H), 1.84-1.77 (m, 1H), 1.57-1.50 (m, 1H), 1.21 (d, J = 6.4 Hz, 3H), 0.84 (q, J = 7.2 Hz, 3H). ^{13}C NMR (101 MHz, DMSO-*d6*, dr = 1:1) δ 156.6, 152.9, 152.4, 152.3, 151.4, 115.9, 115.8, 87.7, 87.5, 85.9, 85.7, 71.1, 70.8, 70.7, 62.2, 32.6, 32.5, 28.4, 28.0, 19.5, 19.2, 11.7, 11.6. HRMS-ESI m/z calcd. for $C_{14}H_{22}N_5NaO_5$ $[M+H]^+$ 340.1621 found 340.1621.

2-amino-8-cyclobutyl-9-((2R,3R,4S,5R)-3,4-dihydroxy-5-(hydroxymethyl)tetrahydrofuran-2-yl)-1,9-dihydro-6H-purin-6-one (Compound 3q)

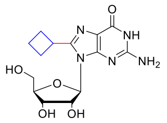

3q was obtained following the general procedure **A**. After purification by column chromatography (C18 Spherical silica) using MeOH/H_2O as the eluents, **3q** was obtained as a white solid (53.9 mg, 80%). mp 202.1-203.2 °C. $[\alpha]_D^{25}$ -32.00 (c 0.425, MeOH). ^{1}H NMR (400 MHz, DMSO-d_6) δ 10.63 (s, 1H), 6.28 (s, 2H), 5.52 (d, J = 6.8 Hz, 1H), 5.30 (d, J = 6.8 Hz, 1H), 5.17-5.14 (m, 1H), 5.07 (d, J = 4.8 Hz, 1H), 4.71 (q, J = 6.4 Hz, 1H), 4.11-4.08 (m, 1H), 3.87-3.84 (m, 1H), 3.70-3.62 (m, 2H), 3.56-3.50 (m, 1H), 2.43-2.36 (m, 1H), 2.34-2.21 (m, 3H), 2.03-1.94 (m, 1H), 1.89-1.80 (m, 1H). ^{13}C NMR (101 MHz, DMSO-d_6) δ 156.4, 152.9, 151.7, 151.0, 115.8, 87.7, 85.7, 71.3, 70.6, 62.1, 31.8, 26.9, 26.7, 17.9. HRMS-ESI m/z calcd for $C_{14}H_{19}N_5NaO_5$ $[M+Na]^+$ 360.1278. found 360.1281.

2-amino-8-cyclopentyl-9-((2R,3R,4S,5R)-3,4-dihydroxy-5-(hydroxymethyl)tetrahydrofuran-2-yl)-1,9-dihydro-6H-purin-6-one (Compound 3r)

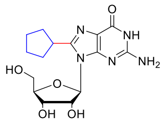

3r was obtained following the general procedure **A**. After purification by column chromatography (C18 Spherical silica) using MeOH/H_2O as the eluents, **3r** was obtained as a white solid (52.1 mg, 80%). mp 262.1-264.2 °C. $[\alpha]_D^{25}$ -14.00 (c 0.0500, MeOH). ^{1}H NMR (400 MHz, DMSO-d_6) δ 10.64 (s, 1H), 6.26 (s, 2H), 5.67 (d, J = 6.8 Hz, 1H), 5.32 (s, 1H), 5.17-5.14 (m, 1H), 5.07 (s, 1H), 4.87 (s, 1H), 4.12 (s, 1H), 3.89-3.86 (, 1H), 3.68-3.64 (m, 1H), 3.56-3.50 (m, 1H), 3.28-3.20 (m, 1H), 1.96-1.81 (m, 4H), 1.73-1.58 (m, 4H). ^{13}C NMR (101 MHz, DMSO-d_6) δ 156.4, 152.8, 151.9, 151.6, 115.6, 87.8, 85.8, 71.1, 70.7, 62.2, 36.6, 31.7, 31.4, 25.1. HRMS-ESI m/z calcd for $C_{15}H_{22}N_5O_5$ $[M+H]^+$ 352.1621. found 352.1612.

2-amino-8-cyclohexyl-9-((2R,3R,4S,5R)-3,4-dihydroxy-5-(hydroxymethyl)tetrahydrofuran-2-yl)-1,9-dihydro-6H-purin-6-one (Compound 3s)

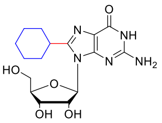

3s was obtained following the general procedure **A**. After purification by column chromatography (C18 Spherical silica) using MeOH/H_2O as the eluents, **3s** was obtained as a white solid (42.0 mg, 58%). mp >280 °C. $[\alpha]_D^{25}$ -43.08 (c 0.0650, MeOH). ^{1}H NMR (400 MHz, DMSO-d_6) δ 10.59 (s, 1H), 6.23 (s, 2H), 5.63 (d, *J* = 6.8 Hz, 1H), 5.34 (d, *J* = 6.8 Hz, 1H), 5.14-5.11 (m, 1H), 5.07 (d, *J* = 4.8 Hz, 1H), 4.88-4.83 (m, 1H), 4.13-4.10 (m, 1H), 3.89-3.87 (m, 1H), 3.68-3.63 (m, 1H), 3.56-3.50 (m, 1H), 2.82-2.76 (m, 1H), 1.88 (t, *J* = 14.4 Hz, 2H), 1.76-1,67 (m, 3H), 1.57-1.45 (m, 2H), 1.40-1.31 (m, 2H), 1.27-1.19 (m, 2H). ^{13}C NMR (101 MHz, DMSO-d_6) δ 156.4, 152.7, 152.4, 151.3, 115.8, 87.6, 85.8, 71.1, 70.7, 62.2, 35.3, 31.8, 31.5, 25.6. HRMS-ESI m/z calcd for $C_{16}H_{23}N_5NaO_5$ $[M+Na]^+$ 388.1591. found 388.1597.

2-amino-9-((2R,3R,4S,5R)-3,4-dihydroxy-5-(hydroxymethyl)tetrahydrofuran-2-yl)-8-(tetrahydro-2H-pyran-4-yl)-1,9-dihydro-6H-purin-6-one (Compound 3t)

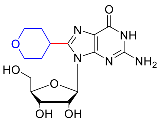

3t was obtained following the general procedure **B**. After purification by column chromatography (C18 Spherical silica) using MeOH/H_2O as the eluents, **3t** was obtained as a white solid (53.3 mg, 72%). mp 257.1-258.0 °C. $[\alpha]_D^{25}$ -40.00 (c 0.0400, MeOH). ^{1}H NMR (400 MHz, DMSO-d_6) δ 10.61 (s, 1H), 6.26 (s, 2H), 5.68 (d, *J* = 6.8 Hz, 1H), 5.34 (d, *J* = 6.8 Hz, 1H), 5.15-5.12 (m, 1H), 5.08 (d, *J* = 4.8 Hz, 1H), 4.86-4.81 (m, 1H), 4.13-4.10 (m, 1H), 3.94-3.87 (m, 3H), 3.69-3.63 (m, 1H), 3.57-3.51 (m, 1H), 3.48-3.42 (m, 2H), 3.17-3.09 (m, 1H), 1.87-1.67(m, 4H). ^{13}C NMR (101 MHz, DMSO-d_6) δ 156.4, 152.8, 151.4, 151.1, 115.8, 87.5, 85.9, 71.2, 70.7, 66.6, 62.0, 32.7, 31.8, 31.1. HRMS-ESI m/z calcd for $C_{15}H_{21}N_5NaO_6$ $[M+Na]^+$ 390.1384. found 390.1387.

tert-butyl-4-(2-amino-9-((2R,3R,4S,5R)-3,4-dihydroxy-5-(hydroxymethyl)tetrahydrofuran-2-yl)-6-oxo-6,9-dihydro-1H-purin-8-yl)piperidine-1-carboxylate (Compound 3u)

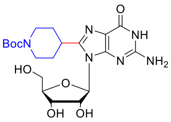

3u was obtained following the general procedure **B**. After purification by column chromatography (C18 Spherical silica) using MeOH/H_2O as the eluents, **3u** was obtained as a white solid (46.6 mg, 50%). mp 265.2-267.9 °C. $[\alpha]_D^{25}$ -26.67 (c 0.0300, MeOH). ^{1}H NMR (400 MHz, DMSO-d_6) δ 10.64

(s, 1H), 6.29 (s, 2H), 5.69 (d, J = 7.2 Hz, 1H), 5.34 (d, J = 6.8 Hz, 1H), 5.14-5.12 (m, 1H), 5.08-5.07 (m, 1H), 4.83-4.78 (m, 1H), 4.14-4.10 (m, 1H), 4.02-3.95 (m, 2H), 3.90-3,87 (m, 1H), 3.69-3.63 (m, 1H), 3.57-3.52 (m, 1H), 3.14-3.07 (m, 1H), 2.94-2.78 (s, 2H), 1.88-1.79 (m, 2H), 1.65-1.49 (m, 2H), 1.41 (s, 9H). ^{13}C NMR (101 MHz, DMSO-d_6) δ 156.4, 153.9, 152.9, 151.4, 151.0, 115.8, 87.4, 85.9, 78.6, 71.3, 70.6, 62.0, 33.3, 31.1, 30.3, 28.1. HRMS-ESI m/z calcd for $C_{20}H_{30}N_6NaO_7$ $[M+Na]^+$ 489.2068. found 489.2076.

2-amino-8-(tert-butyl)-9-((2R,3R,4S,5R)-3,4-dihydroxy-5-(hydroxymethyl)tetrahydrofuran-2-yl)-1,9-dihydro-6H-purin-6-one (Compound 3w)

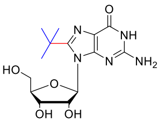

3w was obtained following the general procedure **B**. After purification by column chromatography (C18 Spherical silica) using MeOH/H_2O as the eluents, **3w** was obtained as a white solid (56.2 mg, 82%). mp 203.1-205.8 °C. $[\alpha]_D^{25}$ -35.49 (c 0.170, MeOH). ^{1}H NMR (400 MHz, DMSO-d_6) δ 10.58 (s, 1H), 6.20 (s, 2H), 5.95 (d, J = 6.0 Hz, 1H), 5.34 (d, J = 6.4 Hz, 1H), 5.09-5.06 (m, 2H), 4.99 (d, J = 5.6 Hz, 1H), 4.18-4.15 (m, 1H), 3.88-3.85 (m, 1H), 3.71-3.66 (m, 1H), 3.57-3.51 (m, 1H), 1.39 (s, 9H). Analytical data are consistent with those previously reported [53].

2-amino-8-(tert-amyl)-9-((2R,3R,4S,5R)-3,4-dihydroxy-5-(hydroxymethyl)tetrahydrofuran-2-yl)-1,9-dihydro-6H-purin-6-one (Compound 3x)

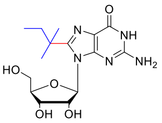

3x was obtained following the general procedure **C**. After purification by column chromatography (C18 Spherical silica) using MeOH/H_2O as the eluents, **3x** was obtained as a white solid (19.6 mg, 56%). mp 202.4-213.5 °C. $[\alpha]_D^{25}$ -14.71 (c 0.170, MeOH). ^{1}H NMR (400 MHz, DMSO-d_6) δ 10.89 (s, 1H), 6.29 (s, 2H), 5.91 (d, J = 4.0 Hz, 1H), 5.33 (s, 1H), 5.22 (s, 1H), 5.05 (t, J = 12.0 Hz, 1H), 5.03 (s, 1H), 4.15 (m, 1H), 3.87 (m, 1H), 3.71-3.68 (m, 1H), 3.55-3.52 (m, 1H), 1.81-1.69 (m, 2H), 1.35 (d, J = 4.0 Hz, 6H), 0.69 (t, J = 12.0 Hz, 3H). ^{13}C NMR (101 MHz, DMSO-d_6) δ 153.0, 152.9, 152.3, 115.4, 89.2, 85.8, 70.9, 70.7, 62.4, 37.6, 33.8, 28.0, 27.7, 9.3. HRMS-ESI m/z calcd for $C_{15}H_{23}N_5NaO_5$ $[M+Na]^+$ 376.1591. found 376.1592.

2-amino-9-((2R,3R,4S,5R)-3,4-dihydroxy-5-(hydroxymethyl)tetrahydrofuran-2-yl)-8-(4-(4-methoxyphenyl)-2-methylbutan-2-yl)-1,9-dihydro-6H-purin-6-one (Compound 3y)

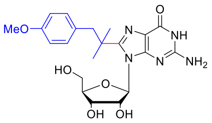

3y was obtained following the general procedure **B**. After purification by column chromatography (C18 Spherical silica) using MeOH/H_2O as the eluents, **3y** was obtained as a white solid (34.8 mg, 76%). mp 170.4-172.5 °C. $[\alpha]_D^{25}$ -13.35 (c 0.130, MeOH). ^{1}H NMR (400 MHz, DMSO-d_6) δ 10.66 (s, 1H), 7.14-7.12 (m, 2H), 6.79-6.77 (m, 2H), 6.33 (s, 2H), 5.97 (d, J = 6.0 Hz, 1H), 5.39 (d, J = 6.4 Hz, 1H), 5.18-5.13 (m, 1H), 5.11-5.07 (m, 1H), 5.01 (d, J = 5.2 Hz, 1H), 4.19-4.15 (m, 1H), 3.90-3.87 (m, 1H), 3.74-3.69 (m, 4H), 3.59-3.52 (m, 1H), 2.30-2.22 (m, 2H), 2.01-1.90 (m, 3H), 1.44 (s, 6H). ^{13}C NMR (126 MHz, DMSO-d_6) δ 157.3, 156.4, 152.7, 152.4, 134.1, 129.3, 115.4, 113.6, 89.3, 85.7, 70.8, 70.4, 62.3, 54.9, 44.3, 37.4, 30.0, 28.6, 28.2. HRMS-ESI m/z calcd for $C_{22}H_{30}N_5O_6$ $[M+H]^+$ 460.2196. found 460.2196.

8-((3R,5R,7R)-adamantan-1-yl)-2-amino-9-((2R,3R,4S,5R)-3,4-dihydroxy-5-(hydroxymethyl)tetrahydrofuran-2-yl)-1,9-dihydro-6H-purin-6-one (Compound 3z)

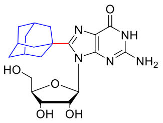

3z was obtained following the general procedure **B**. After purification by column chromatography (C18 Spherical silica) using MeOH/H_2O as the eluents, **3z** was obtained as a white solid (31.6 mg, 76%). mp >280 °C. $[\alpha]_D^{25}$ -21.58 (c 0.190, MeOH). ^{1}H NMR (400 MHz, DMSO-d_6) δ 10.99 (s, 1H), 6.45 (s, 2H), 6.09 (d, J = 6.0 Hz, 1H), 5.40 (s, 1H), 5.24 (s, 1H), 5.04 (t, J = 5.6 Hz, 2H), 4.19-4.17 (m, 1H), 3.90-3.87 (m, 1H), 3.71-3.68 (m, 1H), 3.54-3.53 (m, 1H), 2.11-2.00 (m, 9H), 1.72 (s, 6H). ^{13}C NMR (101 MHz, DMSO-d_6) δ 157.1, 153.5, 153.1, 152.2, 115.4, 89.3, 85.7, 70.9, 62.4, 40.6, 36.1, 36.0, 27.8. HRMS-ESI m/z calcd for $C_{20}H_{27}N_5NaO_5$ $[M+Na]^+$ 440.1904. found 440.1905.

5.2. Substrate scope of C–H ethylation of guanosine analogues

Supplementary Table 14. Substrate scope of C–H ethylation of guanosine analogues [a]

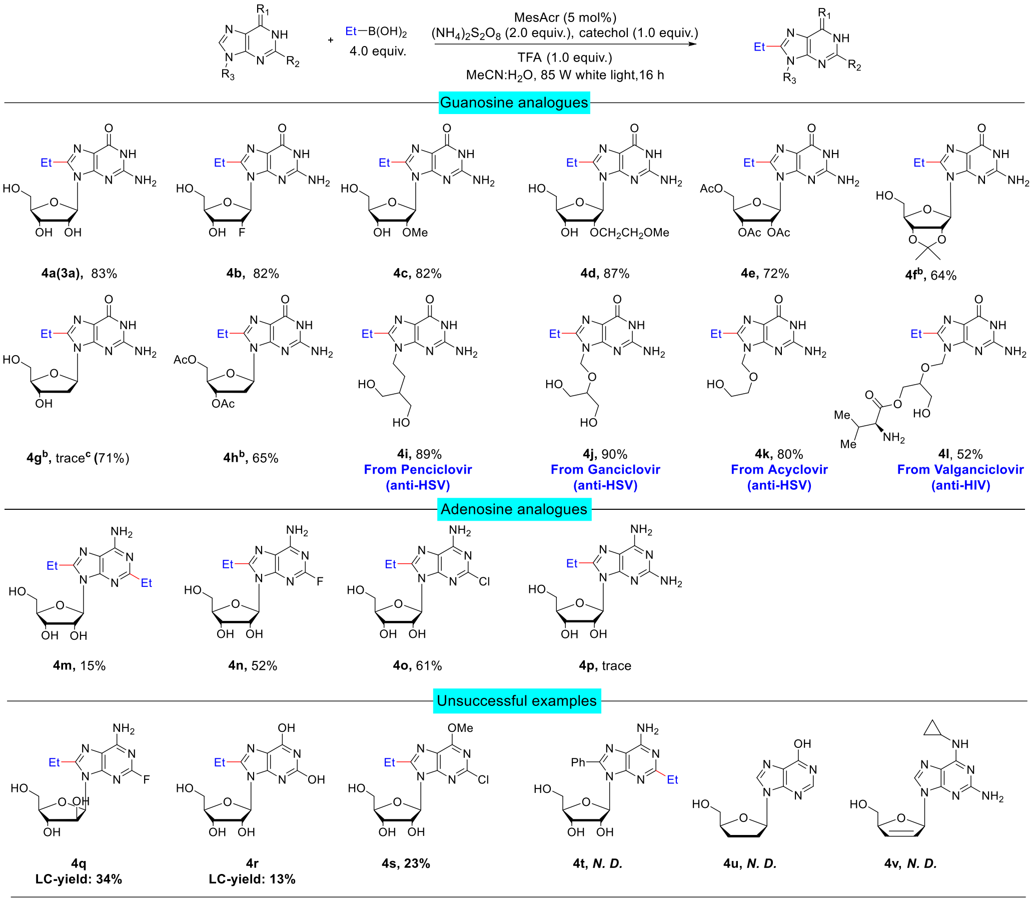

[a] General condition: Guanosine (1.0 equiv.), alkylboronic acid (4.0 equiv.), MesAcr (5 mol%), $(NH_4)_2S_2O_8$ (2.0 equiv.), TFA (1.0 equiv.) and catechol (1.0 equiv.) in MeCN: H_2O (1: 1, 0.1 M) on 0.2 mmol scale; irradiated by 85 W white light at r.t. for 16 h; isolated yield. [b] No TFA was used. [c] Ribose cleaved byproduct **4g'** was obtained in 71% yield.

General procedure of alkylation of guanosine analogues

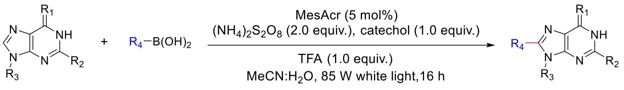

General procedure D (0.1 mmol scale): To a 10 mL Schlenk tube containing a Teflon stir bar was charged with nucleoside substrate (0.1 mmol, 1.0 equiv.), alkylboronic acid (0.4 mmol, 4.0 equiv.), MesAcr (0.005 mmol, 0.05 equiv.), $(NH_4)_2S_2O_8$ (0.2 mmol, 2.0 equiv.), catechol (0.1 mmol, 1.0 equiv.), 0.5 mL CH_3CN and 0.5 mL H_2O, and trifluoroacetic acid (0.1 mmol, 1.0 equiv.) sequentially. The Schlenk tube was sealed with a rubber plug and taped, utilizing the freeze-pump-thaw (FPT) method for deaeration. The reaction system was exposed to 85 W white light for **16 h**, monitoring the progress of the reaction by thin layer chromatography (TLC) (DCM/MeOH = 10/1) or the liquid chromatography-mass spectrometry (LC-MS). The mixture was concentrated under reduced pressure, and the crude product was purified by silica gel flash column chromatography (C18 Spherical silica) using MeOH/H_2O as eluents to give the pure product.

Characterization data for compounds of alkylated guanosine analogues

2-amino-8-ethyl-9-((2R,3R,4R,5R)-3-fluoro-4-hydroxy-5-(hydroxymethyl)tetrahydrofuran-2-yl)-1,9-dihydro-6H-purin-6-one (Compound 4b)

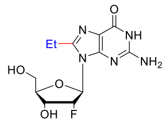

4b was obtained following the general procedure **A.** After purification by column chromatography (C18 Spherical silica) using MeOH/H_2O as the eluents, **4b** was obtained as a white solid (51.3 mg, 82%). mp 273.3-275.4 °C. $[\alpha]_D^{25}$ -12.73 (c 0.0550, MeOH). ^{1}H NMR (400 MHz, DMSO-d_6) δ 10.67 (s, 1H), 6.44 (s, 2H), 5.93 (dd, J = 21.2, 3.2 Hz, 1H), 5.66-5.50 (m, 2H), 4.96 (s, 1H), 4.52-4.45 (m, 1H), 3.88-3.84 (m, 1H), 3.68 (d, J = 12.0 Hz, 1H), 3.55-3.50 (m, 1H), 2.79-2.70 (m, 2H), 1.22 (t, J = 7.6 Hz, 3H). ^{13}C NMR (101 MHz, DMSO-d_6) δ 156.4, 153.3, 151.7, 148.9, 115.3, 92.0 ($J_{C\text{-}F}$ = 186.1 Hz), 85.7 ($J_{C\text{-}F}$ = 34.5 Hz), 83.9, 68.6 ($J_{C\text{-}F}$ = 15.9 Hz), 61.2, 20.4, 11.5. ^{19}F NMR (377 MHz, DMSO-d_6) δ -201.29. HRMS-ESI m/z calcd for $C_{12}H_{17}FN_5O4$ $[M+H]^+$ 314.1265. found 314.1258.

2-amino-8-ethyl-9-((2R,3R,4R,5R)-4-hydroxy-5-(hydroxymethyl)-3-methoxytetrahydrofuran-2-yl)-1,9-dihydro-6H-purin-6-one (Compound 4c)

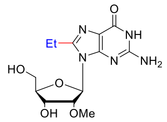

4c was obtained following the general procedure **A**. After purification by column chromatography (C18 Spherical silica) using MeOH/H_2O as the eluents, **4c** was obtained as a white solid (53.3 mg, 82%). mp 144.3-147.0 °C. $[\alpha]_D^{25}$ -45.85 (c 0.103, MeOH). ^{1}H NMR (400 MHz, DMSO-d_6) δ 10.95 (s, 1H), 6.38 (s, 2H), 5.75 (d, J = 6.8 Hz, 1H), 5.17 (s, 2H), 4.50 (dd, J = 6.8, 5.3 Hz, 1H), 4.30-4.28 (m, 1H), 3.89-3.86 (m, 1H), 3.66-3.62 (m, 1H), 3.55-3.51 (m, 1H), 3.28 (s, 3H), 2.76-2.71 (m, 2H), 1.22 (t, J = 7.2 Hz, 3H). ^{13}C NMR (101 MHz, DMSO-d_6) δ 156.6, 153.2, 151.8, 149.2, 115.5, 86.0, 85.6, 80.1, 68.8, 61.7, 57.5, 20.8, 11.7. HRMS-ESI m/z calcd for $C_{13}H_{20}N_5O_5$ $[M+H]^+$ 326.1464. found 326.1455.

2-amino-8-ethyl-9-((2R,3R,4R,5R)-4-hydroxy-5-(hydroxymethyl)-3-(2-methoxyethoxy)tetrahydrofuran-2-yl)-1,9-dihydro-6H-purin-6-one (Compound 4d)

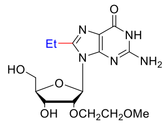

4d was obtained following the general procedure **A**. After purification by column chromatography (C18 Spherical silica) using MeOH/H_2O as the eluents, **4d** was obtained as a white solid (64.2 mg,

87%). mp 128.9-131.4 °C. $[\alpha]_D^{25}$ -53.67 (c 0.127, MeOH). ^{1}H NMR (400 MHz, DMSO-d_6) δ 10.77 (s, 1H), 6.40 (s, 2H), 5.75 (d, J = 6.8 Hz, 1H), 5.19 (s, 1H), 5.03 (s, 1H), 4.67 (dd, J = 6.9, 5.3 Hz, 1H), 4.28-4.26 (m, 1H), 3.88 (q, J = 4.0 Hz, 1H), 3.68-3.62 (m, 2H), 3.54-3.45 (m, 2H), 3.37 (s, 2H), 3.13 (s, 3H), 2.76-2.70 (m, 2H), 1.23 (t, J = 7.2 Hz, 3H). ^{13}C NMR (101 MHz, DMSO-d_6) δ 156.6, 153.2, 151.8, 149.2, 115.5, 85.9, 85.8, 79.0, 71.2, 69.1, 69.0, 61.8, 58.0, 20.8, 11.7. HRMS-ESI m/z calcd for $C_{15}H_{24}N_5O_6$ $[M+H]^+$ 370.1727. found 370.1722.

(2R,3R,4R,5R)-2-(acetoxymethyl)-5-(2-amino-8-ethyl-6-oxo-1,6-dihydro-9H-purin-9-yl)tetrahydrofuran-3,4-diyl diacetate (Compound 4e)

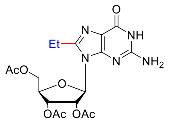

4e was obtained following the general procedure **A**. After purification by column chromatography (C18 Spherical silica) using MeOH/H_2O as the eluents, **4e** was obtained as a white solid (62.9 mg, 72%). mp 128.5-130.1 °C. $[\alpha]_D^{25}$ -7.27 (c 0.385, MeOH). ^{1}H NMR (400 MHz, DMSO-d_6) δ 10.73 (s, 1H), 6.45 (s, 2H), 5.91 (d, J = 4.4 Hz, 1H), 5.84 (dd, J = 6.4, 4.5 Hz, 1H), 5.65 (t, J = 6.4 Hz, 1H), 4.40 (dd, J = 11.7, 3.2 Hz, 1H), 4.31-4.27 (m, 1H), 4.24-4.20 (m, 1H), 2.71 (q, J = 7.6 Hz, 2H), 2.11 (s, 3H), 2.05 (s, 3H), 2.00 (s, 3H), 1.21 (t, J = 7.2 Hz, 3H). ^{13}C NMR (101 MHz, DMSO-d_6) δ 170.2, 169.6, 169.5, 156.4, 153.3, 151.7, 148.7, 115.4, 85.9, 79.0, 71.8, 70.1, 63.1, 20.5, 20.4, 20.4, 20.3, 11.5. HRMS-ESI m/z calcd for $C_{18}H_{24}N_5O_8$ [M+Na]+ 438.1625. found 438.1613.

2-amino-8-ethyl-9-((3aR,4R,6R,6aR)-6-(hydroxymethyl)-2,2-dimethyltetrahydrofuro[3,4-d][1,3]dioxol-4-yl)-1,9-dihydro-6H-purin-6-one (Compound 4f)

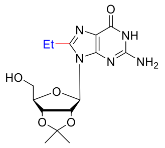

4f was obtained following the general procedure **A**, **while the reaction was performed without the TFA**. After purification by column chromatography (C18 Spherical silica) using MeOH/H_2O as the eluents, **4f** was obtained as a white solid (45.1 mg, 64%). mp 175.1-177.9 °C. $[\alpha]_D^{25}$ -21.94 (c 0.155, MeOH). ^{1}H NMR (400 MHz, DMSO-d_6) δ 10.71 (s, 1H), 6.50 (s, 2H), 5.88 (d, J = 2.4 Hz, 1H), 5.37-5.35 (m, 1H), 5.08-5.06 (m, 1H), 4.94 (s, 1H), 4.04-4.00 (m, 1H), 3.57-3.52 (m, 1H), 3.48-3.44 (m, 1H), 2.82-2.66 (m, 2H), 1.51 (s, 3H), 1.31 (s, 3H), 1.22 (t, J = 7.6 Hz, 3H). ^{13}C NMR (101 MHz, DMSO-d_6) δ 156.5, 153.2, 151.3, 148.7, 115.2, 113.2, 88.1, 87.1, 82.9, 81.2, 61.6, 27.1, 25.3, 20.4, 11.5. HRMS-ESI m/z calcd for $C_{15}H_{22}N_5O_5$ [M+H]+ 352.1621. found 352.1617.

2-amino-8-ethyl-1,9-dihydro-6H-purin-6-one (Compound 4g')

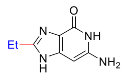

4g’ was obtained following the general procedure **D**, **while the reaction was performed without the TFA**. After purification by column chromatography (C18 Spherical silica) using MeOH/H_2O as the eluents, **4g’** was obtained as a white solid (12.7 mg, 71%). mp >280 °C. ^{1}H NMR (400 MHz, DMSO-d_6) δ 12.27 (d, J = 176.7 Hz, 1H), 10.59 (s, 1H), 6.27 (d, J = 38.9 Hz, 2H), 2.59 (dd, J = 15.1, 7.8 Hz, 2H), 1.20 (t, J = 7.7 Hz, 3H). ^{13}C NMR (176 MHz, DMSO-d_6) δ 156.6, 153.2, 152.6, 149.0, 115.7, 21.8, 12.2. HRMS-ESI m/z calcd. for $C_7H_9N_5NaO$ $[M+Na]^+$ 202.0699. found 202.0697

((2R,3S,5R)-3-acetoxy-5-(2-amino-8-ethyl-6-oxo-1,6-dihydro-9H-purin-9-yl)tetrahydrofuran-2-yl)methyl acetate (Compound 4h)

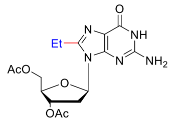

4h was obtained following the general procedure **D**, **while the reaction was performed without TFA and irradiated for 8 hours.** After purification by column chromatography (C18 Spherical silica) using MeOH/H_2O as the eluents, **4h** was obtained as a white solid (26.5 mg, 65%). mp 125.4-127.8 °C. $[\alpha]_D^{25}$ -97.56 (c 0.410, MeOH). ^{1}H NMR (400 MHz, DMSO-d_6) δ 10.63 (s, 1H), 6.39 (s, 2H), 6.16 (t, J = 7.3 Hz, 1H), 5.37-5.33 (m, 3.3 Hz, 1H), 4.39-4.35 (m, 4.3 Hz, 1H), 4.24-4.20 (m, 6.3 Hz, 1H), 4.18-4.13 (m, 1H), 3.31-3.24 (m, 7.6 Hz, 1H), 2.84-2.69 (m, 4.8 Hz, 2H), 2.36-2.30 (m, 6.8, 2.6 Hz, 1H), 2.07 (s, 3H), 2.01 (s, 3H), 1.23 (t, J = 7.4 Hz, 3H). ^{13}C NMR (101 MHz, DMSO-d_6) δ 170.2, 170.2, 156.4, 152.9, 151.8, 149.0, 115.4, 83.2, 81.3, 74.5, 63.6, 34.3, 20.8, 20.8, 20.6, 11.5. HRMS-ESI m/z calcd. for $C_{16}H_{21}N_5O_6$ $[M+H]^+$ 380.1570. found 380.1565.

2-amino-8-ethyl-9-(4-hydroxy-3-(hydroxymethyl)butyl)-1,9-dihydro-6H-purin-6-one (Compound 4i)

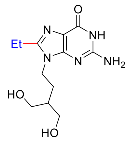

4i was obtained following the general procedure **A**. After purification by column chromatography (C18 Spherical silica) using MeOH/H_2O as the eluents, **4i** was obtained as a white solid (50.3 mg, 89%). mp 259.7-263.1 °C. ^{1}H NMR (400 MHz, DMSO-d_6) δ 10.47 (s, 1H), 6.41 (s, 2H), 4.46 (s, 2H), 3.96-3.92 (m, 2H), 3.46-3.42 (m, 2H), 3.37 (s, 2H), 2.68 (q, J = 7.2 Hz, 2H), 1.63-1.58 (m, 2H), 152-1.46 (m, 1H), 1.24 (t, J = 7.6 Hz, 3H). ^{13}C NMR (101 MHz, DMSO-d_6) δ 156.5, 153.2, 152.0, 148.7, 115.0, 61.4, 41.0, 40.1, 28.7, 19.9, 11.3. HRMS-ESI m/z calcd for $C_{12}H_{20}N_5O_3$ $[M+H]^+$ 282.1566. found 282.1559.

2-amino-9-(((1,3-dihydroxypropan-2-yl)oxy)methyl)-8-ethyl-1,9-dihydro-6H-purin-6-one (Compound 4j)

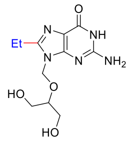

4j was obtained following the general procedure **A**. After purification by column chromatography (C18 Spherical silica) using MeOH/H_2O as the eluents, **4j** was obtained as a white solid (51.0 mg, 90%). mp 235.9-340.1 °C. ^{1}H NMR (400 MHz, DMSO-d_6) δ 10.55 (s, 1H), 6.46 (s, 2H), 5.41 (s, 2H), 4.61 (s, 2H), 3.49-3.38 (m, 4H), 3.29 (d, J = 5.9 Hz, 1H), 2.75 (q, J = 7.6 Hz, 2H), 1.24 (t, J = 7.2 Hz, 3H). ^{13}C NMR (101 MHz, DMSO-d_6) δ 156.6, 153.5, 152.3, 149.7, 114.7, 79.8, 70.2, 60.8, 19.9, 11.0. HRMS-ESI m/z calcd for $C_{11}H17N_5NaO_4$ [M+Na]$^+$ 284.1359. found 284.1353.

2-amino-8-ethyl-9-((2-hydroxyethoxy)methyl)-1,9-dihydro-6H-purin-6-one (Compound 4k)

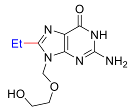

4k was obtained following the general procedure **A**. After purification by column chromatography (C18 Spherical silica) using MeOH/H_2O as the eluents, **4k** was obtained as a white solid (45.5 mg, 80%). mp 208.6-209.4 °C. ^{1}H NMR (400 MHz, DMSO-d_6) δ 10.56 (s, 1H), 6.48 (s, 2H), 5.32 (s, 2H), 4.67-4.66 (m, 1H), 3.46-3.42 (m, 4H), 2.72 (q, J = 7.6 Hz, 2H), 1.24 (t, J = 7.6 Hz, 3H). ^{13}C NMR (101 MHz, DMSO-d_6) δ 156.5, 153.6, 152.4, 149.5, 114.7, 70.7, 70.1, 59.9, 19.9, 11.2. HRMS-ESI m/z calcd for $C_{10}H_{16}N_5O_3$ [M+H]$^+$ 254.1253. found 254.1248.

2-((2-amino-8-ethyl-6-oxo-1,6-dihydro-9H-purin-9-yl)methoxy)-3-hydroxypropyl L-valinate (Compound 4l)

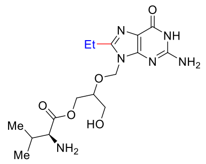

4l was obtained following the general procedure **D**. After purification by column chromatography (C18 Spherical silica) using MeOH/H_2O as the eluents, **4l** was obtained as a white solid (20.0 mg, 52%). mp 208.6-209.4 °C. $[\alpha]_D^{25}$ -4.80 (c 0.167, MeOH), ^{1}H NMR (400 MHz, DMSO-d_6) δ 10.57 (s, 1H), 8.32 (s, 3H), 6.45 (s, 2H), 5.48-5.33 (m, 2H), 4.94 (s, 1H), 4.32 (dd, J = 11.8, 3.1 Hz, 1H), 4.02 (dd, J = 11.7, 6.2 Hz, 1H), 3.83-3.65 (m, 2H), 3.47 (t, J = 7.0 Hz, 3H), 2.72 (qd, J = 7.7, 3.7 Hz, 2H), 1.24 (t, J = 7.4 Hz, 3H), 0.86 (d, J = 6.9 Hz, 6H). ^{13}C NMR (126 MHz, DMSO-d_6) δ 169.0, 156.4, 153.6, 152.3, 149.5, 114.6, 76.3, 69.8, 64.8, 59.9, 57.2, 29.4, 19.9, 17.8, 17.5, 11.0. HRMS-ESI m/z

calcd for $C_{16}H_{26}N_6NaO_5$ $[M+Na]^+$ 405.1857. found 405.1856.

(2R,3R,4S,5R)-2-(6-amino-2,8-diisopropyl-9H-purin-9-yl)-5-(hydroxymethyl)tetrahydrofuran-3,4-diol (Compound 4m)

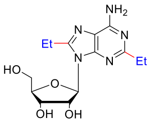

4m was obtained following the general procedure **D**. After purification by column chromatography (C18 Spherical silica) using MeOH/H_2O as the eluents, **4m** was obtained as a white solid (18.1 mg, 26%). mp 127.2-132.3 °C. $[\alpha]_D^{25}$ -50.65 (c 0.310, MeOH). ^{1}H NMR (400 MHz, DMSO-*d₆*) δ 7.18 (s, 2H), 6.25 (d, *J* = 9.9 Hz, 1H), 5.74 (d, *J* = 7.3 Hz, 1H), 5.39 (s, 1H), 5.24 (s, 1H), 4.90 (t, *J* = 6.1 Hz, 1H), 4.16 (d, *J* = 5.1 Hz, 1H), 4.03 (s, 1H), 3.71 (d, *J* = 12.2 Hz, 1H), 3.56 (t, *J* = 11.0 Hz, 1H), 3.37 (d, *J* = 6.7 Hz, 3H), 2.87 (p, *J* = 7.5 Hz, 2H), 2.61 (q, *J* = 7.6 Hz, 2H), 1.29 (t, *J* = 7.5 Hz, 3H), 1.20 (t, *J* = 7.7 Hz, 3H). ^{13}C NMR (126 MHz, DMSO-d_6) δ 164.6, 155.4, 152.9, 150.3, 116.5, 88.4, 86.9, 71.9, 71.3, 62.5, 31.5, 20.8, 13.4, 12.2. HRMS-ESI m/z calcd. for $C_{14}H_{22}N_5O_4$ $[M+H]^+$ 324.1666. found 324.1665

(2R,3R,4S,5R)-2-(6-amino-8-ethyl-2-fluoro-9H-purin-9-yl)-5-(hydroxymethyl)tetrahydrofuran-3,4-diol (Compound 4n)

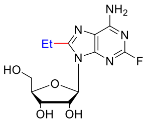

4n was obtained following the general procedure **D**. After purification by column chromatography (C18 Spherical silica) using MeOH/H_2O as the eluents, **4n** was obtained as a white solid (32.3 mg, 52%). mp 248.4-249.0 ℃. $[\alpha]_D^{25}$ -61.13 (c 0.265, MeOH). ^{1}H NMR (400 MHz, DMSO-d_6) δ 7.73 (s, 2H), 5.71 (d, *J* = 6.8 Hz, 1H), 5.36 (d, *J* = 6.6 Hz, 1H), 5.21 (d, *J* = 4.5 Hz, 1H), 5.09-5.06 (m, 4.3 Hz, 1H), 4.84 (q, *J* = 6.1 Hz, 1H), 4.16-4.13 (m, 3.6 Hz, 1H), 3.94 (q, *J* = 3.8 Hz, 1H), 3.75-3.65 (m, 4.3 Hz, 1H), 3.56-3.50 (m, 4.4 Hz, 1H), 2.88 (q, *J* = 7.5 Hz, 2H), 1.30 (t, 3H). ^{13}C NMR (101 MHz, DMSO-d_6) δ 157.603 (J_{C-F} = 204.6 Hz), 157.391 (J_{C-F} = 21.0 Hz), 153.851 (J_{C-F} = 2.8 Hz), 151.074 (J_{C-F} = 19.7 Hz), 116.391 (J_{C-F} = 4.0 Hz), 88.073, 86.129, 71.335, 70.591, 61.938, 20.870, 11.984. ^{19}F NMR (376 MHz, DMSO-d_6) δ -52.67. HRMS-ESI m/z calcd. for $C_{12}H_{16}FN_5NaO_4$ $[M+Na]^+$ 336.1079. found 336.1079.

(2R,3R,4S,5R)-2-(6-amino-2-chloro-8-ethyl-9H-purin-9-yl)-5-(hydroxymethyl)tetrahydrofuran-3,4-diol (Compound 4o)

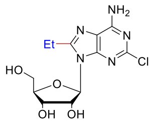

4o was obtained following the general procedure **D**, **while the reaction was irradiated for 20 hours**. After purification by column chromatography (C18 Spherical silica) using MeOH/H2O as the eluents, **4o** was obtained as a white solid (20.0 mg, 61%). mp 116.0-120.9 °C. $[\alpha]_D^{25}$ -50.29 (c 0.350, MeOH). ^{1}H NMR (400 MHz, DMSO-d_6) δ 7.74 (s, 2H), 5.73 (d, J = 7.0 Hz, 1H), 5.40 (d, J = 6.4 Hz, 1H), 5.31-5.20 (m, 1H), 5.11-5.01 (m, 4.2 Hz, 1H), 4.83 (t, J = 5.8 Hz, 1H), 4.15-4.13 (m, 2.8 Hz, 1H), 3.97-3.94 (m, 2.2 Hz, 1H), 3.72-3.64 (m, 1H), 3.57-3.52 (m, 3.9 Hz, 1H), 2.89 (q, J = 7.5 Hz, 2H), 1.30 (t, J = 7.5 Hz, 3H). ^{13}C NMR (126 MHz, DMSO-d_6) δ 156.1, 154.1, 151.8, 151.0, 117.2, 88.1, 86.4, 71.6, 70.8, 62.0, 20.9, 12.0. HRMS-ESI m/z calcd for $C_{12}H_{16}ClN_5NaO_4$ $[M+Na]^+$ 352.0783. found 352.0788.

5.3. Substrate scope of C–H alkylation of uridine

Supplementary Table 15. Substrate scope of C–H alkylation of uridine

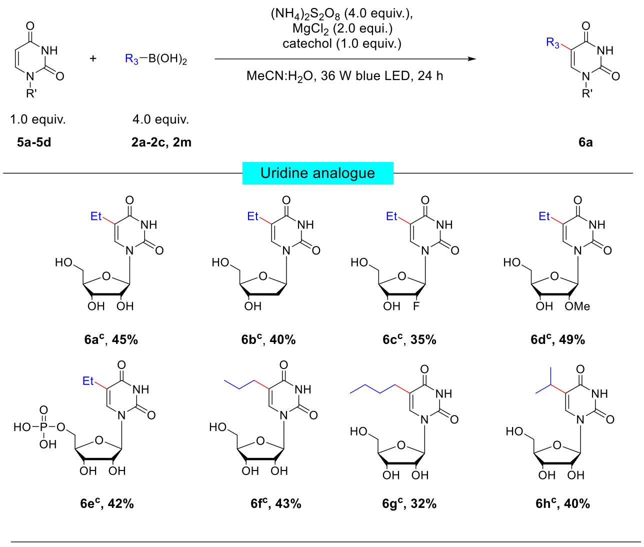

[c] General condition: Uridine (1.0 equiv.), alkylboronic acid (4.0 equiv.), $(NH_4)_2S_2O_8$ (4.0 equiv.), $MgCl_2$ (2.0 equiv.) and catechol (1.0 equiv.) in DMSO: H_2O (1: 1, 0.1 M) on 0.2 mmol scale; irradiated by 36 W blue LED at r.t. for 24 h.

General procedure of alkylation of uridine, deoxyuridine, uridine monophosphate and its analogues

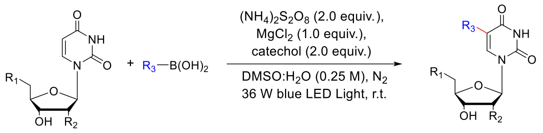

1 **2** **3**

General procedure E (0.2 mmol scale): Nucleoside or Nucleotide substrate (0.2 mmol), alkylboronic acid (0.8 mmol, 4.0 equiv.), $(NH_4)_2S_2O_8$ (0.4 mmol, 2.0 equiv.), $MgCl_2$ (0.2 mmol, 1.0 equiv.), and catechol (0.4 mmol, 2.0 equiv.) were dissolved in 0.4 mL DMSO and 0.4 mL H_2O. The reaction system was exposed to 36 W blue LED for 24 h under nitrogen (or argon) environment. The solvent was concentrated under reduced pressure and the residue was purified by column chromatography (C18 Spherical silica) to give the pure product.

Characterization data for compounds of alkylated uridine, deoxyuridine, uridine monophosphate and its analogues

1-((2*R*,3*R*,4*S*,5*R*)-3,4-dihydroxy-5-(hydroxymethyl)tetrahydrofuran-2-yl)-5-ethylpyrimidine-2,4(1H,3H)-dione (Compound 6a)

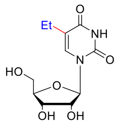

6a was obtained following the general procedure **E**. After purification by column chromatography (C18 Spherical silica) using MeOH/H_2O as the eluents, **6a** was obtained as a green solid (24.5 mg, 45%). mp >300 °C. $[\alpha]_D^{25}$ -13.28 (c 0.620, MeOH). ^{1}H NMR (400 MHz, CD_3OD-*d_4*) δ 7.86 (s, 1H), 5.92 (d, *J* = 4.4 Hz, 1H), 4.23-4.15 (m, 2H), 4.04-3.98 (m, 1H), 3.86 (dd, *J* = 12.2, 2.6 Hz, 1H), 3.74 (dd, *J* = 12.4, 2.8 Hz, 1H), 2.33 (q, *J* = 7.4 Hz, 2H), 1.12 (t, *J* = 7.4 Hz, 3H). ^{13}C NMR (101 MHz, CD_3OD-*d_4*) δ 166.0, 152.6, 137.8, 117.4, 90.5, 86.3, 75.7, 71.4, 62.2, 21.0, 13.3. HRMS-ESI m/z calcd for $C_{11}H_{16}N_2NaO_6$ $[M+Na]^+$ 295.0901. found 295.0902.

5-ethyl-1-((2*R*,3*R*,4*S*,5*R*)-4-hydroxy-5-(hydroxymethyl)tetrahydrofuran-2-yl)pyrimidine-2,4(1H,3H)-dione (Compound 6b)

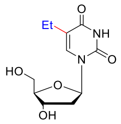

6b was obtained following the general procedure **E**. After purification by column chromatography (C18 Spherical silica) using MeOH/H_2O as the eluents, **6b** was obtained as a yellow brown solid (21.0 mg, 40%). mp >300 °C. $[\alpha]_D^{25}$ 5.67 (c 0.617, DMSO). ^{1}H NMR (400 MHz, DMSO-*d_6*) δ 11.27 (s, 1H), 7.69 (s, 1H), 6.17 (t, *J* = 6.8 Hz, 1H), 4.30-4.20 (m, 1H), 3.77 (dd, *J* = 6.2, 3.4 Hz, 1H), 3.59 (dd, *J* = 11.8, 3.4 Hz, 1H), 3.55 (dd, *J* = 11.8, 3.4 Hz, 1H), 2.24–2.16 (m, 2H), 2.11–2.03 (m, 2H), 1.02 (t, *J* = 7.4 Hz, 3H). ^{13}C NMR (101 MHz, DMSO-*d_6*) δ 163.4, 150.4, 135.6, 115.2, 87.4, 83.9, 70.5, 61.3, 19.7, 12.9. HRMS-ESI m/z calcd for $C_{11}H_{16}N_2NaO_5$ $[M+Na]^+$ 279.0951. found 279.0952.

5-ethyl-1-((2*R*,3*R*,4*S*,5*R*)-3-fluoro-4-hydroxy-5-(hydroxymethyl)tetrahydrofuran-2-yl)pyrimidine-2,4(1H,3H)-dione (Compound 6c)

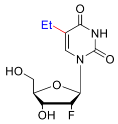

6c was obtained following the general procedure **E**. After purification by column chromatography (C18 Spherical silica) using MeOH/H_2O as the eluents, **6c** was obtained as light green oily (19.2 mg, 35%). $[\alpha]_D^{25}$ -3.79 (c 0.299, DMSO). ^{1}H NMR (400 MHz, DMSO-*d_6*) δ 7.77 (s, 1H), 5.91 (dd, *J* = 17.6, 2.0 Hz, 1H), 5.10-5.05 (m, 1H), 4.97-4.92 (m, 1H), 4.23-4.11 (m, 2H), 3.89-3.84 (m, 1H), 3.78 (d, *J*

= 2.4 Hz, 1H), 3.75 (d, J = 2.4 Hz, 1H), 2.18 (q, J = 7.6 Hz, 2H), 1.01 (t, J = 7.6 Hz, 3H). ^{13}C NMR (101 MHz, CD_3OD -d_4) δ 163.7, 150.4, 135.7, 115.2, 93.8 (J_{C-F} = 185.9 Hz), 87.2 (J_{C-F} = 33.8 Hz), 83.3, 67.5 (J_{C-F} = 15.8 Hz), 59.4, 19.8, 12.9. ^{19}F NMR (376 MHz, DMSO-d_6) δ -202.3. HRMS-ESI m/z calcd for $C_{11}H_{15}FN_2NaO_5$ $[M+Na]^+$ 297.0857. found 297.0859.

5-ethyl-1-((2R,3R,4R,5R)-4-hydroxy-5-(hydroxymethyl)-3-methoxytetrahydrofuran-2-yl) pyrimidine-2,4(1H,3H)-dione (Compound 6d)

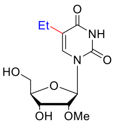

6d was obtained following the general procedure **E**. After purification by column chromatography (C18 Spherical silica) using MeOH/H_2O as the eluents, **6d** was obtained as a brown solid (27.8 mg, 49%). mp 186.5-189.7 °C. $[\alpha]_D^{25}$ -4.08 (c 0.695, DMSO). ^{1}H NMR (400 MHz, DMSO-d_6) δ 11.31 (s, 1H), 7.79 (s, 1H), 5.86 (d, J = 5.2 Hz, 1H), 5.30-5.09 (m, 2H), 4.18-4.12 (m, 1H), 3.84 (dd, J = 7.0, 3.0 Hz, 1H), 3.79 (t, J = 5.0 Hz, 1H), 3.66 (dd, J = 11.8, 1.8 Hz, 1H), 3.57 (dd, J = 12.0, 2.0 Hz, 1H), 3.31 (s, 1H), 3.25-3.07 (m, 1H), 2.20 (q, J = 7.2 Hz, 2H), 1.03 (t, J = 7.6 Hz, 3H). ^{13}C NMR (101 MHz, DMSO-d_6) δ 163.4, 150.4, 135.5, 115.2, 85.9, 85.1, 82.7, 68.3, 60.4, 57.5, 19.7, 12.8. HRMS-ESI m/z calcd for $C_{12}H_{18}N_2NaO_6$ $[M+Na]^+$ 309.1057. found 309.1060.

((2R,3S,4R,5R)-5-(5-ethyl-2,4-dioxo-3,4-dihydropyrimidin-1(2H)-yl)-3,4-dihydroxytetrahydrofuran-2-yl)methyl phosphate (Compound 6e)

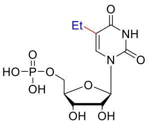

6e was obtained following the general procedure **E**. After purification by column chromatography (C18 Spherical silica) using MeOH/H_2O as the eluents, **6e** was obtained as brown oily (29.4 mg, 42%). $[\alpha]_D^{25}$ -14.01 (c 0.483, H_2O). ^{1}H NMR (400 MHz, D_2O) 7.64 (s, 1H), 5.95 (d, J = 5.2 Hz, 1H), 4.34 (t, J = 5.4 Hz, 1H), 4.32-4.28 (m, 1H), 4.26-4.22 (m, 1H), 4.17-4.06 (m, 2H), 2.31 (q, J = 7.6 Hz, 2H), 1.05 (t, J = 7.6 Hz, 3H). ^{13}C NMR (101 MHz, D_2O) δ 166.0, 151.8, 136.6, 117.6, 88.0, 83.5 (d, J = 8.9 Hz), 73.5, 69.9, 64.3, 38.6, 19.7, 12.5. ^{31}P NMR (162 MHz, D_2O) δ -0.2. HRMS-ESI m/z calcd for $C_{11}H_{17}N_2NaO_9P$ $[M+Na]^+$ 375.0564. found 375.0563.

1-((2*R*,3*R*,4*S*,5*R*)-3,4-dihydroxy-5-(hydroxymethyl)tetrahydrofuran-2-yl)-5-propyl pyrimidine-2,4(1H,3H)-dione (Compound 6f)

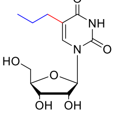

6f was obtained following the general procedure **E**. After purification by column chromatography (C18 Spherical silica) using MeOH/H_2O as the eluents, **6f** was obtained as a yellow brown solid (24.7 mg, 43 %). mp 214.5-216.6 °C. $[\alpha]_D^{25}$ -12.32 (c 1.055, DMSO). ^{1}H NMR (400 MHz, DMSO-*d6*) δ 11.27 (s, 1H), 7.75 (s, 1H), 5.77 (d, *J* = 5.2 Hz, 1H), 5.24-5.09 (m, 2H), 4.06-4.01 (m, 1H), 4.00-3.95 (m, 1H), 3.85-3.81 (m, 1H), 3.65-3.59 (m, 1H), 3.58-3.52 (m, 2H), 2.20-2.10 (m, 2H), 1.46-1.39 (m, 2H), 0.85 (t, *J* = 7.4 Hz, 4H). ^{13}C NMR (101 MHz, DMSO-*d6*) δ 163.5, 150.7, 136.5, 113.3, 87.7, 84.8, 73.6, 69.9, 60.8, 28.3, 21.2, 13.6. HRMS-ESI m/z calcd for $C_{12}H_{18}N_2NaO_6$ $[M+Na]^+$ 309.1057. found 309.1051.

5-butyl-1-((2*R*,3*R*,4*S*,5*R*)-3,4-dihydroxy-5-(hydroxymethyl)tetrahydrofuran-2-yl)pyrimidine-2,4(1H,3H)-dione (Compound 6g)

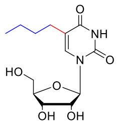

6g was obtained following the general procedure **E**. After purification by column chromatography (C18 Spherical silica) using MeOH/H_2O as the eluents, **6g** was obtained as yellow brown oily (19.2 mg, 32%). $[\alpha]_D^{25}$ -16.79 (c 0.980, DMSO). ^{1}H NMR (400 MHz, DMSO-*d6*) δ 11.26 (s, 1H), 7.74 (s, 1H), 5.77 (d, *J* = 5.6 Hz, 1H), 5.25-5.02 (m, 2H), 4.05-4.01 (m, 1H), 4.00-3.95 (m, 1H), 3.85-3.81 (m, 1H), 3.63 (dd, *J* = 12.0, 3.2 Hz, 1H), 3.55 (dd, *J* = 12.2, 3.0 Hz, 1H), 2.25-2.11 (m, 2H), 1.44-1.35 (m, 2H), 1.31-1.21 (m, 3H), 0.87 (t, *J* = 7.3 Hz, 3H). ^{13}C NMR (101 MHz, DMSO-*d6*) δ 163.5, 150.7, 136.4, 113.5, 87.6, 84.8, 73.6, 69.9, 60.9, 30.1, 25.9, 21.7, 13.8. HRMS-ESI m/z calcd for $C_{13}H_{20}N_2NaO_6$ $[M+Na]^+$ 323.1214. found 323.1213.

1-((2*R*,3*R*,4*S*,5*R*)-3,4-dihydroxy-5-(hydroxymethyl)tetrahydrofuran-2-yl)-5-isopropylpyrimidine-2,4(1H,3H)-dione (Compound 6h)

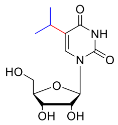

6h was obtained following the general procedure **E**. After purification by column chromatography (C18 Spherical silica) using MeOH/H_2O as the eluents, **6h** was obtained as light brown oily (22.9 mg, 40%). $[\alpha]_D^{25}$ -12.52 (c 1.265, DMSO). ^{1}H NMR (400 MHz, DMSO-*d6*) δ 11.24 (s, 1H), 7.76 (s, 1H), 5.80 (d, *J* = 5.2 Hz, 1H), 5.36 (d, *J* = 5.6 Hz, 1H), 5.19 (t, *J* = 4.4 Hz, 1H), 5.08 (d, *J* = 4.8 Hz, 1H), 4.04 (dd, *J* = 10.4, 5.2 Hz, 1H), 3.98 (dd, *J* = 8.6, 4.6 Hz, 1H), 3.88–3.83 (m, 1H), 3.69-3.62 (m, 1H), 3.61-3.53 (m, 1H), 2.77-2.68 (m, 1H), 1.09-1.03 (m, 6H). ^{13}C NMR (101 MHz, DMSO-*d6*) δ 163.1, 150.5, 134.9, 119.5, 87.9, 84.8, 73.9, 70.0, 60.7, 25.5, 21.5, 21.5. HRMS-ESI m/z calcd for $C_{12}H_{18}N_2NaO_6$ $[M+Na]^+$ 309.1057. found 309.1058.

5.4. Substrate scope of C–H alkylation of complex nucleotide substates

Supplementary Table 16. Substrate scope of C–H alkylation of complex nucleotide substates [a]

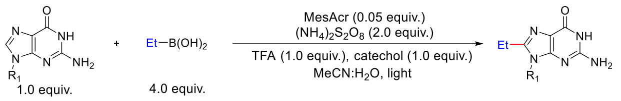

7a-7k **8**

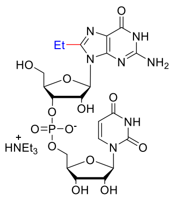

8a, 44%

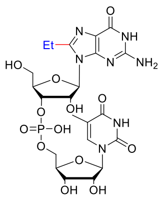

8b, 80%

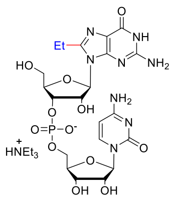

8c, 61%

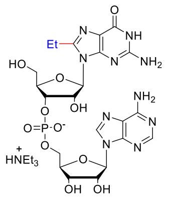

8d, 58%

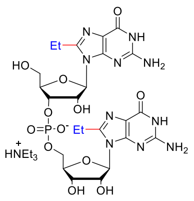

8e, 41%

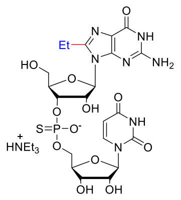

8f/8f' (Gp(SR$_p$)U)/(Gp(SS$_p$)U), 61%/ 64%

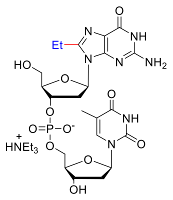

8g[b], 60%

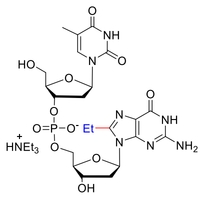

8h[b], 60%

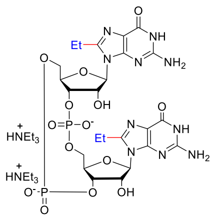

8i, 53%

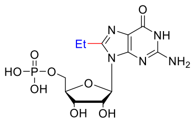

8j[c], 52%(LC-yield: 91%)

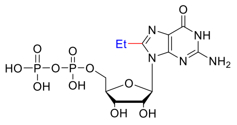

8k[d], 51%(LC-yield: 85%)

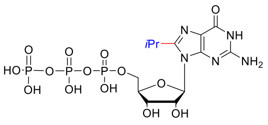

8l[d,e], 43%(LC-yield: 71%)

[a] General condition: Dinucleotides or nucleotides (1.0 equiv.), alkylboronic acid (4.0 equiv.), MesAcr (5 mol%), $(NH_4)_2S_2O_8$ (2.0 equiv.), TFA (1.0 equiv.) and catechol (1.0 equiv.) in MeCN: H_2O (1: 1, 0.1M) on 0.06 mmol scale; irradiated by 10 W blue LED at about 10 °C for 24 h; isolated yield. [b] No TFA was used and shortened the reaction time to 5 hours. [c] Irradiated by 36 W blue LED at r.t. for 16 h. [d] Irradiated by 85 W white light at r.t. for 16 h. 10 mM NH_4HCO_3 (aq.) was used instead of H_2O, and No TFA was used. [e] *i*-PrB(OH)$_2$ was used instead of EtB(OH)$_2$ as C8-Et-GTP cannot be separated from GTP.

General procedure of alkylation of complex nucleotide substrates

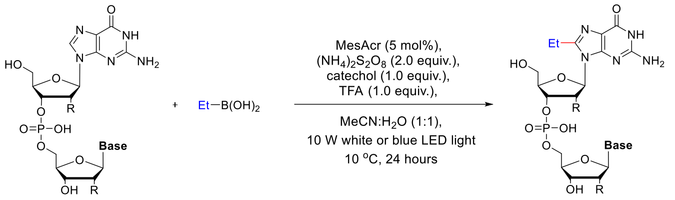

7a-7f 2a 8a-8f

R = OH, H

General procedure F (0.06 mmol scale): To a 10 mL Schlenk tube containing a Teflon stir bar was charged with **dinucleotide** (0.06 mmol, 1.0 equiv.), ethylboronic acid (0.24 mmol, 4.0 equiv.), MesAcr (0.0003 mmol, 0.05 equiv.), $(NH_4)_2S_2O_8$ (0.12 mmol, 2.0 equiv.), catechol (0.06 mmol, 1.0 equiv.), 0.3 mL CH_3CN and 0.3 mL H_2O, and trifluoroacetic acid (0.06 mmol, 1.0 equiv.) sequentially. The Schlenk tube was sealed with a rubber plug and taped, utilizing the freeze-pump-thaw (FPT) method for deaeration. The reaction system was exposed to 10 W white or blue LED at 10 °C for **24 h**, monitoring the progress of the reaction by the liquid chromatography-mass spectrometry (LC-MS). The mixture was concentrated under reduced pressure, and the crude product was purified by silica gel flash column chromatography (C18 Spherical silica) using MeCN/TEAA buffer as eluents to give the pure product.

General procedure G (0.06 mmol scale): To a 10 mL Schlenk tube containing a Teflon stir bar was charged with **deoxy-dinucleotide** (0.06 mmol, 1.0 equiv.), ethylboronic acid (0.24 mmol, 4.0 equiv.), MesAcr (0.0003 mmol, 0.05 equiv.), $(NH_4)_2S_2O_8$ (0.12 mmol, 2.0 equiv.), catechol (0.06 mmol, 1.0 equiv.), and 0.3 mL CH_3CN and 0.3 mL H_2O sequentially. The Schlenk tube was sealed with a rubber plug and taped, utilizing the freeze-pump-thaw (FPT) method for deaeration. The reaction system was exposed to 10 W blue LED at 10 °C for **5 h**, monitoring the progress of the reaction by the liquid chromatography-mass spectrometry (LC-MS). About 4.5 mL TEAA buffer (pH = 7.0) was added to the reaction solution for dilution (ensure that the concentration of CH_3CN was less than 10%), and the mixture was directly purified by silica gel flash column chromatography (C18 Spherical silica), without concentration, using MeCN/TEAA buffer as eluents to give the pure product.

Characterization data for compounds of alkylated complex nucleotide substates

Dinucleotides

2R,3S,4R,5R)-5-(2-amino-8-ethyl-6-oxo-1,6-dihydro-9H-purin-9-yl)-4-hydroxy-2-(hydroxymethyl)tetrahydrofuran-3-yl(((2R,3S,4R,5R)-5-(2,4-dioxo-3,4-dihydropyrimidin-1(2H)-yl)-3,4-dihydroxytetrahydrofuran-2-yl)methyl)hydrogen-phosphate (Compound 8a)

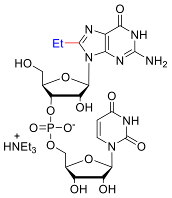

8a was obtained following the general procedure **F**. After purification by column chromatography (C18 Spherical silica) using MeOH/H_2O as the eluents, **8a** was obtained as a white solid (18.9 mg, 44%). mp 251.6-252.7 °C. $[\alpha]_D^{25}$ -8.39 (c 0.143, H_2O). ^{1}H NMR (400 MHz, DMSO-d_6) δ 11.32 (s, 1H), 10.77 (s, 1H), 7.87 (d, J = 8.1 Hz, 1H), 6.58 (s, 1H), 6.45 (s, 2H), 5.81 (d, J = 5.8 Hz, 1H), 5.71 (d, J = 7.7 Hz, 1H), 5.62 (d, J = 8.0 Hz, 1H), 5.46 (s, 1H), 5.35 (s, 1H), 4.74 (t, J = 6.6 Hz, 1H), 4.50 (d, J = 6.6 Hz, 1H), 4.08 (t, J = 5.5 Hz, 1H), 4.02 (d, J = 4.7 Hz, 2H), 3.96 (d, J = 10.4 Hz, 2H), 3.88-3.84 (m, 2H), 3.63-3.54 (m, 3H), 2.94 (q, J = 7.4 Hz, 6H), 2.78-2.70 (m, 2H), 1.21 (t, J = 7.3 Hz, 3H), 1.13 (t, J = 7.2 Hz, 9H). ^{13}C NMR (126 MHz, DMSO-d$_6$) δ 163.2, 156.4, 153.1, 152.0, 150.9, 149.3, 140.9, 115.4, 102.0, 87.6, 87.2, 84.6, 83.4, 74.2, 73.2, 71.0, 70.4, 64.8, 61.8, 45.6, 21.0, 11.7, 8.7. ^{31}P NMR (202 MHz, DMSO-d_6) δ -0.50. HRMS-ESI m/z calcd. for $C_{21}H_{27}N_7O_{13}P$ [M-H]$^-$ 616.1410. found 616.1400.

(2R,3S,4R,5R)-5-(2-amino-8-ethyl-6-oxo-1,6-dihydro-9H-purin-9-yl)-4-hydroxy-2-(hydroxymethyl)tetrahydrofuran-3-yl (((2R,3S,4R,5R)-3,4-dihydroxy-5-(5-methyl-2,4-dioxo-3,4-dihydropyrimidin-1(2H)-yl)tetrahydrofuran-2-yl)methyl) hydrogen phosphate (Compound 8b)

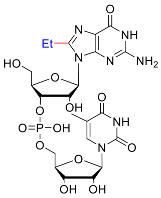

8b was obtained following the general procedure **F**, **while the scale was 0.04 mmol.** After purification by column chromatography (C18 Spherical silica) using MeCN/H_2O as the eluents, **8b** was obtained as a white solid (20.2 mg, 80%). mp 218.0-218.7 °C. $[\alpha]_D^{25}$ -8.33 (c 0.0600, H_2O). ^{1}H NMR (400 MHz, D_2O) δ 7.67 (d, J = 0.8 Hz, 1H), 5.91 (s, 1H), 5.90 (d, J = 2.0 Hz, 1H), 5.04 (t, J = 5.8 Hz, 1H), 4.92-4.88 (m, 1H), 4.40 (dd, J = 6.4, 3.6 Hz, 1H), 4.38-4.3 (m, 2H), 4.29-4.21 (m, 2H),

4.18-4.12 (m, 1H), 3.88 (qd, J = 12.7, 3.5 Hz, 2H), 3.03-2.96 (m, 2H), 1.87 (s, 3H), 1.35 (t, J = 7.5 Hz, 3H). ^{13}C NMR (101 MHz, D_2O) δ 166.2, 153.1, 152.9, 151.7, 151.6, 137.1, 115.3, 111.6, 88.7, 88.4, 84.8, 82.7 (d, J_{C-P} = 8.6 Hz), 74.7 (d, J_{C-P} = 6.9 Hz), 73.3, 71.4, 69.5, 64.7 (d, J_{C-P} = 5.9 Hz), 61.6, 38.6, 20.5, 11.5, 11.1. ^{31}P NMR (162 MHz, D_2O) δ -0.68. HRMS-ESI m/z calcd. for $C_{22}H_{31}N_7O_{13}P$ [M+H]$^+$ 632.1717. found 632.1733.

((2R,3S,4R,5R)-5-(4-amino-2-oxopyrimidin-1(2H)-yl)-3,4-dihydroxytetrahydrofuran-2-yl)methyl ((2R,3S,4R,5R)-5-(2-amino-8-ethyl-6-oxo-1,6-dihydro-9H-purin-9-yl)-4-hydroxy-2-(hydroxymethyl)tetrahydrofuran-3-yl) hydrogen phosphate (Compound 8c)

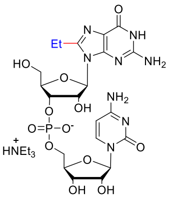

8c was obtained following the general procedure **F**. After purification by column chromatography (C18 Spherical silica) using MeCN/TEAA buffer (pH = 7.0) as the eluents. **8c** was obtained as a white solid (26.1 mg, 61%). mp 234.3-239.4 °C. $[\alpha]_D^{25}$ -1.09 (c 0.0920, H_2O). This was followed by exchange of the counterions [Amberlite IR120 (Na^+)], in order to improve the solubility of the product in D_2O and simplify the NMR spectrum. ^{1}H NMR (400 MHz, D_2O) δ 7.86 (d, J = 7.6 Hz, 1H), 5.99 (d, J = 7.5 Hz, 1H), 5.92 (d, J = 3.6 Hz, 1H), 5.81 (d, J = 6.6 Hz, 1H), 5.01 (t, J = 6.1 Hz, 1H), 4.89-4.84 (m, 1H), 4.39 (q, J = 3.1 Hz, 1H), 4.37-4.22 (m, 4H), 4.16 (dt, J = 11.8, 4.1 Hz, 1H), 3.86 (qd, J = 12.8, 3.1 Hz, 2H), 2.86-2.74 (m, 2H), 1.27 (t, J = 7.5 Hz, 3H). ^{13}C NMR (126 MHz, D_2O) δ 165.8, 158.0, 157.3, 153.1, 152.8, 151.5, 141.4, 115.2, 96.2, 89.8, 88.3, 84.8, 82.3 (d, J_{C-P} = 8.6 Hz), 74.8 (d, J_{C-P} = 4.3 Hz), 74.0, 71.3 (d, J_{C-P} = 4.8 Hz), 69.2, 64.4, 61.7, 20.5, 11.1. ^{31}P NMR (202 MHz, D_2O) δ -0.39. HRMS-ESI m/z calcd. for $C_{21}H_{28}N_8O_{12}P$ [M-H]$^-$ 615.1570. found 615.1554.

(2R,3S,4R,5R)-5-(2-amino-8-ethyl-6-oxo-1,6-dihydro-9H-purin-9-yl)-4-hydroxy-2-(hydroxymethyl)tetrahydrofuran-3-yl (((2R,3S,4R,5R)-5-(6-amino-9H-purin-9-yl)-3,4-dihydroxytetrahydrofuran-2-yl)methyl) phosphate (Compound 8d)

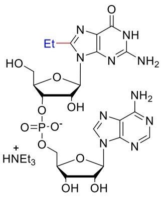

8d was obtained following the general procedure **F**. After purification by column chromatography (C18 Spherical silica) using MeCN/TEAA buffer (pH = 7.0) as the eluents. **8d** was obtained as a white solid (22.1 mg, 58%). mp 223.5-227.1 °C. $[\alpha]_D^{25}$ -50.00 (c 0.0620, H_2O). This was followed by

exchange of the counterions [Amberlite IR120 (Na^+)], in order to improve the solubility of the product in D_2O and simplify the NMR spectrum. ^{1}H NMR (400 MHz, D_2O) δ 8.30 (s, 1H), 8.06 (s, 1H), 5.98 (d, *J* = 5.0 Hz, 1H), 5.60 (d, *J* = 5.4 Hz, 1H), 4.77-4.73 (m, 2H), 4.62 (t, *J* = 5.5 Hz, 1H), 4.51 (t, *J* = 5.2 Hz, 1H), 4.36-4.33 (m, 1H), 4.28-4.24 (m, 1H), 4.19 (q, *J* = 3.8 Hz, 1H), 4.17-4.10 (m, 1H), 3.70 (qd, *J* = 12.8, 3.4 Hz, 2H), 2.76-2.70 (m, 2H), 1.22 (t, *J* = 7.5 Hz, 3H). ^{13}C NMR (126 MHz, D_2O) δ 158.2, 155.2, 152.9, 152.7, 152.6, 151.6, 149.2, 139.5, 118.6, 115.7, 88.4, 87.2, 83.6, 83.1, 74.1, 73.5, 71.5, 69.7, 64.9, 61.3, 20.4, 11.2. ^{31}P NMR (202 MHz, D_2O) δ -0.53. HRMS-ESI m/z calcd. for $C_{22}H_{29}N_{10}NaO_{11}P$ $[M+Na]^+$ 663.1647. found 663.1656.

((2R,3S,4R,5R)-5-(2-amino-8-ethyl-6-oxo-1,6-dihydro-9H-purin-9-yl)-3,4-dihydroxytetrahydrofuran-2-yl)methyl((2R,3S,4R,5R)-5-(2-amino-8-ethyl-6-oxo-1,6-dihydro-9H-purin-9-yl)-4-hydroxy-2-(hydroxymethyl)tetrahydrofuran-3-yl) hydrogen phosphate (Compound 8e)

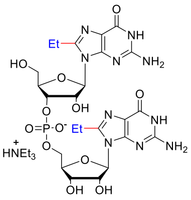

8e was obtained following the general procedure **F**. After purification by column chromatography (C18 Spherical silica) using MeOH/H_2O as the eluents, **8e** was obtained as a white solid (19.3 mg, 41%). mp >280 °C. $[\alpha]_D^{25}$ -10.00 (c 0.15, H_2O). ^{1}H NMR (400 MHz, D_2O) δ 5.81 (d, *J* = 5.1 Hz, 1H), 5.72 (d, *J* = 6.0 Hz, 1H), 5.17 (t, *J* = 5.5 Hz, 1H), 4.75-4.72 (m, 1H), 4.67 (t, *J* = 5.3 Hz, 2H), 4.33-4.17 (m, 4H), 3.60 (qd, *J* = 12.6, 3.1 Hz, 2H), 2.89-2.73 (m, 4H), 1.27 (dt, *J* = 11.5, 7.5 Hz, 6H). ^{13}C NMR (176 MHz, D_2O) δ 158.9, 158.2, 153.4, 153.2, 153.0, 153.0, 152.4, 151.6, 115.3, 114.9, 88.3, 88.2, 84.2 (d, J_{C-P} = 3.4 Hz), 82.6 (d, J_{C-P} = 8.6 Hz), 74.4 (d, J_{C-P} = 5.5 Hz), 71.5 (d, J_{C-P} = 4.9 Hz), 71.1, 69.4, 65.2 (d, J_{C-P} = 5.1 Hz), 61.4, 20.5, 20.5, 11.3, 11.2. ^{31}P NMR (202 MHz, D_2O) δ -0.61. HRMS-ESI m/z calcd. for $C_{24}H_{32}N_{10}O_{12}$ P $[M-H]^-$ 683.1944. found 683.1948.

O-((2R,3S,4R,5R)-5-(2-amino-8-ethyl-6-oxo-1,6-dihydro-9H-purin-9-yl)-4-hydroxy-2-(hydroxymethyl)tetrahydrofuran-3-yl) O-(((2R,3S,4R,5R)-5-(2,4-dioxo-3,4-dihydropyrimidin-1(2H)-yl)-3,4-dihydroxytetrahydrofuran-2-yl)methyl) phosphorothioate (Compound 8f, Gp(SR$_P$)U)

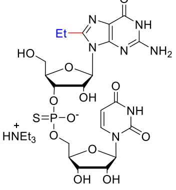

8f was obtained following the general procedure **F**, **while the scale was 0.04 mmol**. After purification by column chromatography (C18 Spherical silica) using MeCN/ TEAA buffer (pH = 7.0) as the eluents, **8f** was obtained as a white solid (27.0 mg, 61%). mp 208.7-216.5 °C. $[\alpha]_D^{25}$ -14.24 (c 0.148, H_2O). This was followed by exchange of the counterions [Amberlite IR120 (Na^+)], in order to improve the solubility of the product in D_2O and simplify the NMR spectrum. ^1H NMR (400 MHz, D_2O) δ 7.97 (d, J = 8.1 Hz, 1H), 5.92 (d, J = 3.7 Hz, 1H), 5.88 (d, J = 8.1 Hz, 1H), 5.83 (d, J = 6.2 Hz, 1H), 5.11-4.99 (m, 2H), 4.48 (q, J = 2.8 Hz, 1H), 4.41-4.20 (m, 5H), 3.92-3.91 (m, 2H), 2.90-2.70 (m, 2H), 1.27 (t, J = 7.5 Hz, 3H). ^{13}C NMR (126 MHz, D_2O) δ 165.9, 157.9, 153.0, 152.7, 151.6, 151.4, 141.7, 115.2, 102.4, 88.9, 88.4, 85.0, 83.0 (d, $J_{C\text{-}P}$ = 9.3 Hz), 75.4, 73.8, 71.3 (d, $J_{C\text{-}P}$ = 5.8 Hz), 69.7, 64.8 (d, $J_{C\text{-}P}$ = 6.0 Hz), 61.8, 20.6, 11.1. ^{31}P NMR (202 MHz, D_2O) δ 56.32. HRMS-ESI m/z calcd. for $C_{21}H_{27}N_7O_{12}PS$ $[M\text{-}H]^-$ 632.1182. found 632.1176.

O-((2R,3S,4R,5R)-5-(2-amino-8-ethyl-6-oxo-1,6-dihydro-9H-purin-9-yl)-4-hydroxy-2-(hydroxymethyl)tetrahydrofuran-3-yl) O-(((2R,3S,4R,5R)-5-(2,4-dioxo-3,4-dihydropyrimidin-1(2H)-yl)-3,4-dihydroxytetrahydrofuran-2-yl)methyl) phosphorothioate (Compound 8f', Gp(SS$_P$)U)

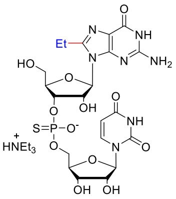

8f' was obtained following the general procedure **F**. After purification by column chromatography (C18 Spherical silica) using MeCN/TEAA buffer (pH = 7.0) as the eluents. **8f'** was obtained as a white solid (28.2 mg, 64%). mp 199.4-206.2 °C. $[\alpha]_D^{25}$ -40.48 (c 0.0840, H_2O). This was followed by exchange of the counterions [Amberlite IR120 (Na^+)], in order to improve the solubility of the product in D_2O and simplify the NMR spectrum. ^1H NMR (400 MHz, D_2O) δ 7.97 (d, J = 8.1 Hz, 1H), 5.93 (d, J = 3.4 Hz, 1H), 5.85-5.83 (m, 2H), 5.87-5.80 (m, 1H), 5.08-5.02 (qt, J = 8.2, 3.7 Hz, 2H), 4.46 (q, J = 2.8 Hz, 1H), 4.38-4.35 (m, 2H), 4.35-4.18 (m, 3H), 3.98-3.82 (m, 2H), 2.91-2.72 (m, 2H), 1.28 (t, J = 7.5 Hz, 3H). ^{13}C NMR (101 MHz, D_2O) δ 166.0, 158.0, 153.1, 152.8, 151.6, 151.5, 141.7, 115.3, 102.4, 88.9, 88.3, 84.8, 82.9 (d, $J_{C\text{-}P}$ = 9.4 Hz), 75.2 (d, $J_{C\text{-}P}$ = 5.7 Hz), 73.8, 71.2 (d, $J_{C\text{-}P}$ = 5.1 Hz), 69.6, 64.9 (d, $J_{C\text{-}P}$ = 5.4 Hz), 61.8, 20.6, 11.1. ^{31}P NMR (202 MHz, D_2O) δ 56.25. HRMS-ESI m/z calcd. for $C_{21}H_{27}N_7O_{12}PS$ $[M\text{-}H]^-$ 632.1182. found 632.1186.

(2R,3S,5R)-5-(2-amino-8-ethyl-6-oxo-1,6-dihydro-9H-purin-9-yl)-2-(hydroxymethyl)tetrahydrofuran-3-yl (((2R,3S,5R)-3-hydroxy-5-(5-methyl-2,4-dioxo-3,4-dihydropyrimidin-1(2H)-yl)tetrahydrofuran-2-yl)methyl) phosphate (Compound 8g)

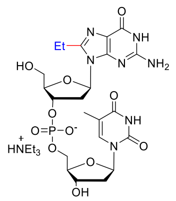

8g was obtained following the general procedure **G**. After purification by column chromatography (C18 Spherical silica) using MeCN/TEAA buffer (pH = 7.0) as the eluents. **8g** was obtained as a white solid (25.3 mg, 60 %). mp 238.5-239.0 °C. $[\alpha]_D^{25}$ 21.33 (c 0.0375, MeOH). ^{1}H NMR (400 MHz, D_2O) δ 7.51 (s, 1H), 6.22 (q, J = 9.7, 8.4 Hz, 2H), 5.00-4.99 (m, 1H), 4.67-4.62 (m, 1H), 4.30-4.29 (m, 1H), 4.25-4.17 (m, 1H), 4.12-4.06 (m, 2H), 3.90-3.84 (m, 2H), 2.97 (ddd, J = 14.4, 9.0, 5.8 Hz, 1H), 2.83 (qd, J = 7.7, 3.6 Hz, 2H), 2.69 (dd, J = 13.6, 6.3 Hz, 1H), 2.40 (dt, J = 14.5, 7.3 Hz, 1H), 2.32-2.26 (m, 1H), 1.77 (s, 3H), 1.28 (t, J = 7.5 Hz, 3H). ^{13}C NMR (176 MHz, D_2O) δ 165.5, 158.3, 152.9, 152.8, 151.6, 151.3, 137.5, 115.5, 111.1, 86.4 (d, J_{C-P} = 9.9 Hz), 84.9, 84.1 (d, J_{C-P} = 9.7 Hz), 83.8, 77.0 (d, J_{C-P} = 6.2 Hz), 69.2, 64.0 (d, J_{C-P} = 5.1 Hz), 62.1, 38.7, 37.9, 20.4, 11.2, 11.0. ^{31}P NMR (202 MHz, D_2O) δ -0.95. HRMS-ESI m/z calcd. for $C_{22}H_{31}N_7O_{11}P$ $[M+H]^+$ 600.1814. found 600.1807.

((2R,3S,5R)-5-(2-amino-8-ethyl-6-oxo-1,6-dihydro-9H-purin-9-yl)-3-hydroxytetrahydrofuran-2-yl)methyl ((2R,3S,5R)-2-(hydroxymethyl)-5-(5-methyl-2,4-dioxo-3,4-dihydropyrimidin-1(2H)-yl)tetrahydrofuran-3-yl) phosphate (Compound 8h)

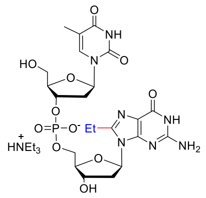

8h was obtained following the general procedure **G**. After purification by column chromatography (C18 Spherical silica) using MeCN/ TEAA buffer (pH = 7.0) as the eluents. **8h** was obtained as a white solid (25.0 mg, 60 %). mp 217.4-222.1 °C. $[\alpha]_D^{25}$ 5.87 (c 0.375, H_2O). ^{1}H NMR (400 MHz, D_2O) δ 7.49 (s, 1H), 6.27 (t, J = 7.1 Hz, 1H), 6.15-6.11 (m, 1H), 4.73-4.66 (m, 2H), 4.24-4.09 (m, 3H), 4.00-3.99 (m, 1H), 3.59-3.45 (m, 2H), 3.24-3.17 (m, 1H), 2.86 (q, J = 7.6 Hz, 2H), 2.47 (dd, J = 13.9, 5.9 Hz, 1H), 2.41-2.32 (m, 1H), 2.05-1.98 (m, 1H), 1.87 (s, 3H), 1.29 (t, J = 7.5 Hz, 3H). ^{13}C NMR (176 MHz, D_2O) δ 166.3, 158.2, 153.0, 152.8, 152.4, 151.4, 136.9, 115.0, 111.6, 85.6 (d, J_{C-P} = 6.3 Hz), 84.9 (d, J_{C-P} = 9.3 Hz), 84.8, 83.4, 75.8 (d, J_{C-P} = 6.0 Hz), 70.3, 65.2 (d, J_{C-P} = 5.3 Hz), 61.0, 37.3, 36.6, 20.7, 11.6, 11.2. ^{31}P NMR (202 MHz, D_2O) δ -0.92. HRMS-ESI m/z calcd. for $C_{22}H_{29}N_7Na_2O_{11}P$ $[M+2Na]^+$ 644.1453. found 644.1451.

c-di-GMP

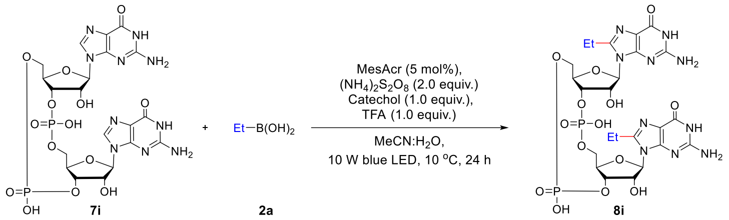

General procedure H (0.04 mmol scale): To a 10 mL Schlenk tube containing a Teflon stir bar was charged with cyclic-dinucleotides (0.04 mmol, 1.0 equiv.), ethylboronic acid (0.16 mmol, 4.0 equiv.), MesAcr (0.0002 mmol, 0.05 equiv.), $(NH_4)_2S_2O_8$ (0.08 mmol, 2.0 equiv.), catechol (0.04 mmol, 1.0 equiv.), 0.2 mL CH_3CN and 0.2 mL H_2O, and trifluoroacetic acid (0.04 mmol, 1.0 equiv.) sequentially. The Schlenk tube was sealed with a rubber plug and taped, utilizing the freeze-pump-thaw (FPT) method for deaeration. The reaction system was exposed to 10 W white or blue LED at 10 °C for **24 h**, monitoring the progress of the reaction by the liquid chromatography-mass spectrometry (LC-MS). The mixture was concentrated under reduced pressure, and the crude product was purified by silica gel flash column chromatography (C18 Spherical silica) using MeCN/TEAA buffer as eluents to give the pure product**.**

9,9'-((2R,3R,3aS,7aR,9R,10R,10aS,14aR)-3,5,10,12-tetrahydroxy-5,12-dioxidooctahydro-2H,7H-difuro[3,2-d:3',2'-j][1,3,7,9]tetraoxa[2,8]diphosphacyclododecine-2,9-diyl)bis(2-amino-8-ethyl-1,9-dihydro-6H-purin-6-one) (Compound 8i)

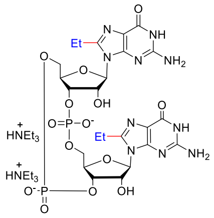

8i was obtained following the general procedure **H**. After purification by column chromatography (C18 Spherical silica) using TEAA buffer (pH=7.0)/MeCN as the eluents, **8i** was obtained as a white solid (15.8 mg, 53%). mp >300 °C. $[\alpha]_D^{25}$ 21.429 (c 0.056, MeOH). ^{1}H NMR (700 MHz, D_2O) 55 ℃, δ 6.16 (s, 2H), 5.50-5.43 (m, 4H), 4.62-4.57 (m, 4H), 4.43-4.40 (m, 2H), 3.47 (q, J = 7.3 Hz, 4H), 1.60 (t, J = 6.9 Hz, 6H). ^{13}C NMR (176 MHz, D_2O) δ 158.8, 153.7, 153.6, 152.9, 115.4, 89.1, 75.8 (dd, $J_{C\text{-}P}$ = 9.5, 7.6 Hz), 72.6 (d, $J_{C\text{-}P}$ = 5.3 Hz), 71.3, 63.1 (d, $J_{C\text{-}P}$ = 5.3 Hz), 47.1, 20.9. ^{31}P NMR (283 MHz, D_2O) δ -0.26. HRMS-ESI m/z calcd. for $C_{24}H_{31}N_{10}O_{14}P_2$ $[M-H]^-$ 745.1502. found 745.1513.

Guanosine monophosphate, guanosine diphosphate, and guanosine triphosphate

((2R,3S,4R,5R)-5-(2-amino-8-ethyl-6-oxo-1,6-dihydro-9H-purin-9-yl)-3,4-dihydroxytetrahydrofuran-2-yl)methyl tetrahydrogen mono-phosphate (Compound 8j)

8j was obtained following the general procedure **D**, **while the reaction was irradiated by the 36 W blue LED**. After purification by column chromatography (C18 Spherical silica) using 10 mM NH_4HCO_3 (aq.)/MeCN as the eluents, **8j** was obtained as a white solid (20.3 mg, 52%). ^1H NMR (400 MHz, D_2O) δ 5.77 (d, J = 6.4 Hz, 1H), 5.14 (t, J = 6.1 Hz, 1H), 4.50 (dd, J = 5.8, 3.6 Hz, 1H), 4.20 (q, J = 4.5 Hz, 1H), 4.16-4.05 (m, 2H), 2.79 (q, J = 7.5 Hz, 2H), 1.24 (t, J = 7.5 Hz, 3H). ^{13}C NMR (101 MHz, D_2O) δ 156.5, 153.9, 153.3, 151.6, 110.7, 88.1, 84.0 (d, $J_{C\text{-}P}$ = 8.7 Hz), 70.9, 69.9, 64.5 (d, $J_{C\text{-}P}$ = 4.9 Hz), 20.2, 10.9. ^{31}P NMR (162 MHz, D_2O) δ 0.52 (s, 1P). HRMS-ESI m/z calcd. for $C_{12}H_{17}N_5O_8P$ $[M-H]^-$ 390.0820. found 390.0832.

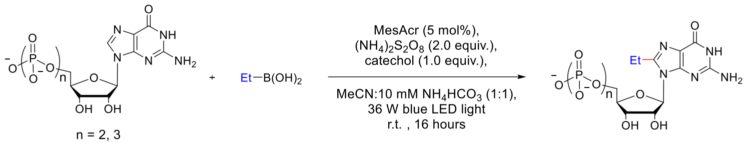

7j, 7k 2a 8j, 8k

General procedure I (0.025 mmol scale): To a 10 mL Schlenk tube containing a Teflon stir bar was charged with guanosine diphosphate disodium salt or guanosine triphosphate disodium salt (0.025 mmol), alkylboronic acid (0.1 mmol, 4.0 equiv.), MesAcr (0.00125 mmol, 0.05 equiv.), $(NH_4)_2S_2O_8$ (0.05 mmol, 2.0 equiv.), catechol (0.025 mmol, 1.0 equiv.), and 0.125 mL CH_3CN and 0.125 mL aqueous 10 mM NH_4HCO_3 sequentially. The Schlenk tube was sealed with a rubber plug and taped, utilizing the freeze-pump-thaw (FPT) method for deaeration. The reaction system was exposed to 85 W white light for **16 h**, monitoring the progress of the reaction by the liquid chromatography-mass spectrometry (LC-MS). The mixture was concentrated under reduced pressure, and the crude product was purified by Semi-PrepHPLC using 0.1M TEAB/ 0.1M TEAB as eluents to give the pure product.

((2R,3S,4R,5R)-5-(2-amino-8-isopropyl-6-oxo-1,6-dihydro-9H-purin-9-yl)-3,4-dihydroxytetrahydrofuran-2-yl)methyl tetrahydrogen diphosphate (Compound 8k)

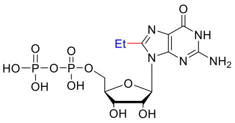

8k was obtained following the general procedure **I.** After purification by column chromatography (Semi-PrepHPLC) using 0.1M TEAB/ 0.1M TEAB in 20% MeCN as the eluents, **8k** was obtained as a white solid (6.0 mg, 51%). ^1H NMR (400 MHz, D_2O) δ 5.92 (d, J = 6.2 Hz, 1H), 5.17 (t, J = 5.9 Hz,

1H), 4.67-4.56 (m, 1H), 4.34-4.27 (m, 1H), 4.26-4.19 (m, 2H), 3.10 (q, J = 7.6 Hz, 2H), 1.37 (t, J = 7.5 Hz, 3H). ^{13}C NMR (101 MHz, D_2O) δ 158.4, 153.4, 153.1, 152.8, 114.9, 87.8, 83.3 (d, J_{C-P} = 8.3 Hz), 70.8, 69.9, 64.6 (d, J_{C-P} = 4.5 Hz), 20.6, 11.3. ^{31}P NMR (162 MHz, D_2O). ^{31}P NMR (162 MHz, D_2O) δ -10.87 (d, J = 20.5 Hz), -11.39 (d, J = 20.4 Hz). HRMS-ESI m/z calcd. for $C_{12}H_{18}N_5O_{11}P_2$ [M-H]$^-$ 470.0484. found 470.0468.

((2R,3S,4R,5R)-5-(2-amino-8-isopropyl-6-oxo-1,6-dihydro-9H-purin-9-yl)-3,4-dihydroxytetrahydrofuran-2-yl)methyl tetrahydrogen triphosphate (Compound 8l)

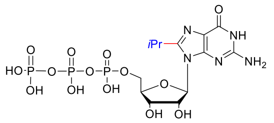

8l was obtained following the general procedure **I**. After purification by column chromatography (Semi-PrepHPLC) using 0.1M TEAB/ 0.1M TEAB in 20% MeCN as the eluents, **8l** was obtained as a white solid (6.0 mg, 43%). ^{1}H NMR (400 MHz, D_2O) δ 5.89 (d, J = 6.5 Hz, 1H), 5.26 (t, J = 6.0 Hz, 1H), 4.62 (s, 1H), 4.29 (s, 3H), 3.28-3.22 (m, 1H), 1.48-1.28 (m, 6H). ^{13}C NMR (101 MHz, D_2O) δ 160.9, 159.6, 155.6, 155.2, 117.5, 90.0, 85.9, 73.1, 72.4, 67.7, 28.8, 23.2, 22.4. ^{31}P NMR (162 MHz, D_2O) δ -5.69 (d, J = 19.5 Hz, 1P), -10.76 (d, J = 18.7 Hz, 1P), -21.29 (t, J = 19.1 Hz, 1P). HRMS-ESI m/z calcd. for $C_{13}H_{21}N_5O_{14}P_3$ [M-H]$^-$ 564.0287. found 564.0276.

6. Applications of the methodology regarding nucleosides

Gram-scale synthesis of alkylated nucleoside

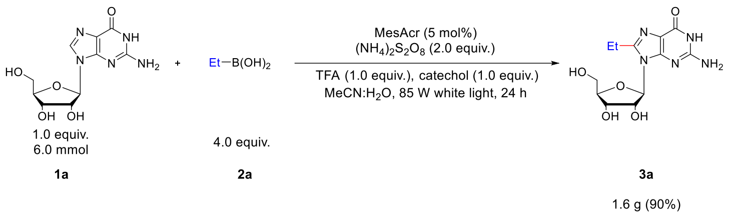

To verify the practicability of the current protocol for the synthesis of nucleobase-functionalized nucleosides, the reaction was scale up to 1.0 g (6.0 mmol). The desired product (**3a)** was obtained in 90% yield (1.6 g, 5.2 mmol).

To a 10 mL Schlenk tube containing a Teflon stir bar was charged with Guanosine (6.0 mmol, 1.0 equiv.), ethylboronic acid (24.0 mmol, 4.0 equiv.), MesAcr (0.3 mmol, 0.05 equiv.), $(NH_4)_2S_2O_8$ (12.0 mmol, 2.0 equiv.), catechol (6.0 mmol, 1.0 equiv.), 30 mL CH_3CN and 30 mL H_2O, and trifluoroacetic acid (6.0 mmol, 1.0 equiv.) sequentially. The Schlenk tube was sealed with a rubber plug and taped, utilizing the freeze-pump-thaw (FPT) method for deaeration. The reaction system was exposed to 85 W white light for **24 h**, monitoring the progress of the reaction by thin layer chromatography (TLC) (DCM/MeOH = 5/1) or the liquid chromatography-mass spectrometry (LC-MS). The mixture was concentrated under reduced pressure, and the crude product was purified by silica gel flash column chromatography (C18 Spherical silica) using MeOH/H_2O as eluents to give the pure product.

Sequential functionalization of G with radical alkylation and reductive amination reaction [18]

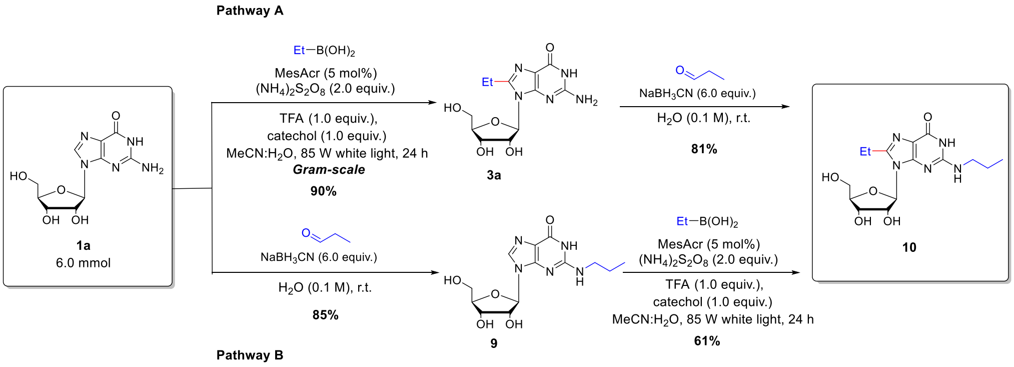

Supplementary Figure 23. Sequential functionalization of G

Pathway A

The method of synthesis compound **3a** have mentioned before. Next, the compound **3a** (0.06 mmol, 1.0 equiv.) was dissolved in water (0.6 mL, 0.1 M), and to these were added propionaldehyde (0.21 mL, 3.0 mmol, 50.0 equiv.) and sodium cyanoborohydride (22.0 mg, 0.36 mmol, 6.0 equiv.). This solution was stirred vigorously at room temperature in a tightly capped vial, and the progress of the reaction was monitored by TLC or LC-MS. The solvent was concentrated under the reduced pressure, and the crude product was purified by silica gel flash column chromatography (C18 Spherical silica) using MeOH/H_2O as eluents to give the pure product. The desired product (**10**) was obtained in 81% yield (17.1 mg).

Compound **10**. mp 189.2-193.2 °C. $[\alpha]_D^{25}$ -4.06 (c 0.320, MeOH). ^{1}H NMR (400 MHz, DMSO-d_6) δ 10.48 (s, 1H), 6.43 (t, J = 5.6 Hz, 1H), 5.58 (d, J = 5.8 Hz, 1H), 5.33 (d, J = 6.3 Hz, 1H), 5.10 (d, J = 5.3 Hz, 1H), 4.94-4.91 (m, 1H), 4.78 (t, J = 5.8 Hz, 1H), 4.14 (d, J = 5.8 Hz, 1H), 3.82 (q, J = 4.9 Hz, 1H), 3.64 (dt, J = 10.6, 5.1 Hz, 1H), 3.50 (dt, J = 11.7, 5.9 Hz, 1H), 3.19 (q, J = 6.6 Hz, 2H), 2.72 (qd, J = 7.7, 2.7 Hz, 2H), 1.53 (h, J = 7.3 Hz, 2H), 1.22 (t, J = 7.4 Hz, 3H), 0.89 (t, J = 7.4 Hz, 3H). ^{13}C NMR (126 MHz, DMSO-d_6) δ 156.5, 152.0, 151.5, 149.4, 115.6, 88.2, 85.0, 70.8, 70.4, 62.1, 42.3, 22.1, 20.6, 11.9, 11.3. HRMS-ESI m/z calcd. for $C_{15}H_{24}N_5O_5$ $[M+H]^+$ 354.1772. found 354.1774.

Pathway B

The guanosine (1.132 g, 4.0 mmol, 1.0 equiv.) was dissolved in water (40 mL, 150 mM), and to these were added 50 equiv. of aldehyde and sodium cyanoborohydride (1.4976g, 24.0 mmol, 6.0 equiv.). This solution was stirred vigorously at room temperature in a tightly capped glass bottle for the

indicated time and then analyzed by TCL or LC-MS to monitor consumption of the starting material. The solvent was concentrated under the reduced pressure, and the crude product was purified by flash column chromatography (C18 Spherical silica) using MeOH/H_2O as eluents to give the pure product. The desired product (**9**) was obtained in 85% yield (1.1102 g).

Next, the compound **9** (0.1 mmol, 1.0 equiv.), ethylboronic acid (0.4 mmol, 4.0 equiv.), MesAcr (0.005 mmol, 0.05 equiv.), $(NH_4)_2S_2O_8$ (0.2 mmol, 2.0 equiv.), catechol (0.1 mmol, 1.0 equiv.), and trifluoroacetic acid (0.1 mmol, 1.0 equiv.) were dissolved in 0.5 mL CH_3CN and 0.5 mL H_2O and under the protection of nitrogen (or argon). The reaction system was exposed to 85 W white light for 24 hours. The solvent was concentrated and the residue was purified by column chromatography (C18 Spherical silica) to give the pure products. The desired product (**10**) was obtained in 61 % yield (21.6 mg).

Compound **9**. mp 130.8-145.8 °C. $[\alpha]_D^{25}$ -11.67 (c 0.360, MeOH). ^{1}H NMR (400 MHz, DMSO-*d6*) δ 10.66 (s, 1H), 7.93 (s, 1H), 6.58 (t, *J* = 5.6 Hz, 1H), 5.72 (d, *J* = 5.8 Hz, 1H), 5.45 (s, 1H), 5.29-5.11 (m, 1H), 4.96 (s, 1H), 4.54 (t, *J* = 5.4 Hz, 1H), 4.13 (t, *J* = 4.2 Hz, 1H), 3.89 (q, *J* = 4.1 Hz, 1H), 3.62 (dd, *J* = 11.8, 4.3 Hz, 1H), 3.58-3.47 (m, 1H), 3.24 (q, *J* = 6.6 Hz, 2H), 1.54 (q, *J* = 7.2 Hz, 2H), 0.90 (t, *J* = 7.5 Hz, 3H). ^{13}C NMR (101 MHz, DMSO-*d6*) δ 157.1, 152.8, 151.1, 136.4, 116.9, 87.0, 85.4, 73.4, 70.7, 61.8, 42.3, 22.1, 11.4. HRMS-ESI m/z calcd. for $C_{13}H_{19}N_5NaO_5$ $[M+Na]^+$ 348.1278. found 348.1278

Bioconjugate with Zidovudine and fluorescent molecular 12 *via* click reaction [54, 55]

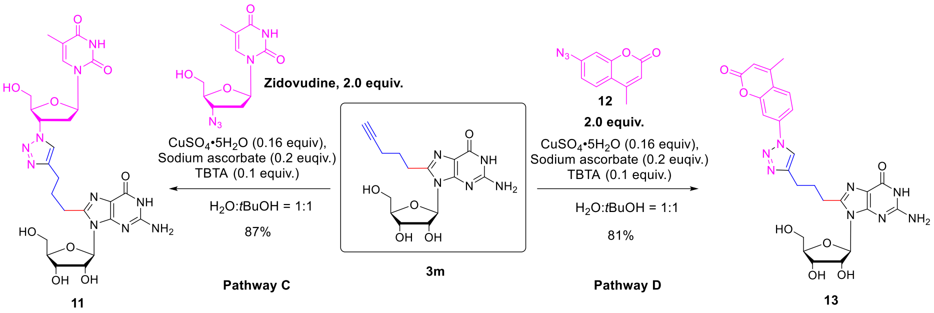

Supplementary Figure 24. Bioconjugation of **3m**

Pathway C

Zidovudine (26.7 mg, 0.1 mmol, 2.0 equiv.), compound **3m** (17.5 mg, 0.05 mmol, 1.0 equiv.), copper (II) sulfate pentahydrate (2.0 mg, 0.008 mmol, 0.16 equiv.), sodium ascorbate (2.0 mg, 0.01 mmol, 0.2 equiv.), and the tris-(benzyltriazolylmethyl)amine (2.6 mg, 0.005, 0.1 equiv.) were dissolved in 0.5 mL *t*BuOH and 0.5 mL H_2O. The solution was stirred vigorously at room temperature, and the progress of the reaction was monitored by TLC or LC-MS. The solvent was concentrated under the reduced pressure, and the crude product was purified by flash column chromatography (C18 Spherical silica) using MeOH/H_2O as eluents to give the pure product. The desired product (**11**) was obtained in 87% yield (26.7 mg).

Compound **11**. mp 194.8-195.0 °C. $[\alpha]_D^{25}$ -10.87 (c 0.0460, MeOH). ^{1}H NMR (400 MHz, DMSO-d_6) δ 11.33 (s, 1H), 10.65 (s, 1H), 8.09 (s, 1H), 7.91-7.78 (m, 1H), 6.41 (t, *J* = 6.6 Hz, 1H), 6.28 (s, 2H), 5.64 (d, *J* = 6.8 Hz, 1H), 5.43-5.29 (m, 2H), 5.27 (q, *J* = 5.3, 4.3 Hz, 1H), 5.14 (dd, *J* = 7.0, 4.7 Hz, 1H), 5.08 (d, *J* = 4.8 Hz, 1H), 4.77 (d, *J* = 6.2 Hz, 1H), 4.19 (dt, *J* = 5.8, 3.6 Hz, 1H), 4.09 (dt, *J* = 7.4, 3.5 Hz, 1H), 3.86 (q, *J* = 4.0 Hz, 1H), 3.69 (dt, *J* = 12.8, 4.8 Hz, 1H), 3.66-3.58 (m, 2H), 3.51 (ddd, *J* = 11.8, 7.0, 4.6 Hz, 1H), 2.77 (dt, *J* = 18.7, 7.5 Hz, 5H), 2.62 (ddd, *J* = 14.2, 8.7, 6.4 Hz, 1H), 2.04 (p, *J* = 7.4 Hz, 2H), 1.81 (s, 3H). ^{13}C NMR (126 MHz, DMSO-d_6) δ 163.8, 156.4, 152.9, 151.6, 150.5, 148.0, 146.8, 136.3, 121.6, 115.7, 109.6, 87.6, 85.7, 84.5, 83.9, 71.1, 70.6, 62.0, 60.8, 59.0, 37.1, 26.7, 26.7, 24.6, 12.3. HRMS-ESI m/z calcd. for $C_{25}H_{33}N_{10}O_9$ $[M+H]^+$ 617.2426. found 617.2427.

Pathway D

Compound **12** (20.1 mg, 0.1 mmol, 2.0 equiv.), compound **3m** (17.5 mg, 0.05 mmol, 1.0 equiv.),

copper (II) sulfate pentahydrate (2.0 mg, 0.008 mmol, 0.16 equiv.), sodium ascorbate (2.0 mg, 0.01 mmol, 0.2 equiv.), and the tris-(benzyltriazolylmethyl)amine (2.6 mg, 0.005 mmol, 0.1 equiv.) were dissolved in 0.5 mL *t*BuOH and 0.5 mL H_2O. The solution was stirred vigorously at room temperature, and the progress of the reaction was monitored by TLC or LC-MS. The solvent was concentrated under the reduced pressure, and the crude product was purified by flash column chromatography (C18 Spherical silica) using MeOH/H_2O as eluents to give the pure product. The desired product (**13**) was obtained in 81% yield (22.2 mg).

Compound **13**. mp 219.9-224.5 °C. $[\alpha]_D^{25}$ -6.58 (c 0.0760, MeOH). ^1H NMR (400 MHz, DMSO-d_6) δ 10.64 (s, 1H), 8.79 (s, 1H), 7.97 (s, 3H), 6.47 (s, 1H), 6.30 (s, 2H), 5.67 (d, *J* = 6.8 Hz, 1H), 5.34 (d, *J* = 6.5 Hz, 1H), 5.14 (t, *J* = 5.9 Hz, 1H), 5.09 (d, *J* = 4.8 Hz, 1H), 4.78 (q, *J* = 6.1 Hz, 1H), 4.09 (s, 1H), 3.86 (q, *J* = 3.9 Hz, 1H), 3.64 (dt, *J* = 12.5, 4.4 Hz, 1H), 3.52 (dt, *J* = 11.9, 5.5 Hz, 1H), 2.84 (d, *J* = 7.8 Hz, 4H), 2.48 (s, 3H), 2.14 (p, *J* = 7.5 Hz, 2H). ^{13}C NMR (126 MHz, DMSO-d_6) δ 159.5, 156.4, 153.7, 152.9, 152.8, 151.6, 148.2, 147.9, 138.7, 127.2, 120.5, 119.2, 115.7, 115.3, 114.5, 107.2, 87.6, 85.7, 71.2, 70.5, 62.0, 26.7, 26.3, 24.6, 18.1. HRMS-ESI m/z calcd. for $C_{25}H_{26}N_8NaO_7$ $[M+Na]^+$ 573.1817. found 573.1815.

Bioconjugate with the biotin alkyne *via* click reaction [54, 55]

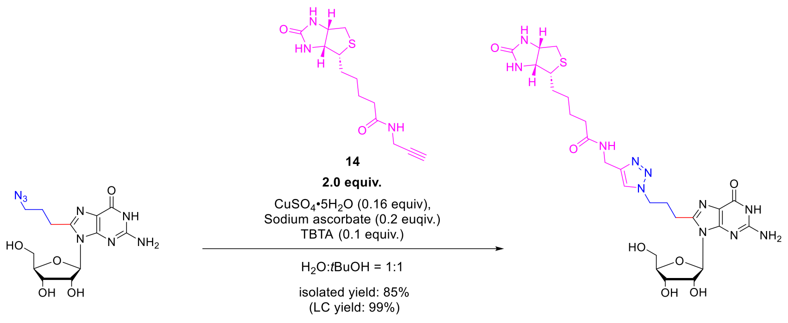

Supplementary Figure 25. Bioconjugation of **3n**

Pathway E

Biotin alkyne (2.81 mg, 0.01 mmol, 2.0 equiv.), compound **3n** (1.94 mg, 0.005 mmol, 1.0 equiv.), copper (II) sulfate pentahydrate (0.2 mg, 0.0008 mmol, 0.16 equiv.), sodium ascorbate (0.2 mg, 0.001 mmol, 0.2 equiv.), and the tris-(benzyltriazolylmethyl)amine (0.26 mg, 0.0005, 0.1 equiv.) were dissolved in 0.05 mL *t*BuOH and 0.05 mL H_2O. The solution was stirred vigorously at room temperature, and the progress of the reaction was monitored by LC-MS. The desired product (**15**) was obtained in 99% LC-yield and 85% isolated yield. HRMS-ESI m/z calcd. for $C_{26}H_{38}N_{11}O_7S$ $[M+H]^+$ 648.2671. found 648.2672.

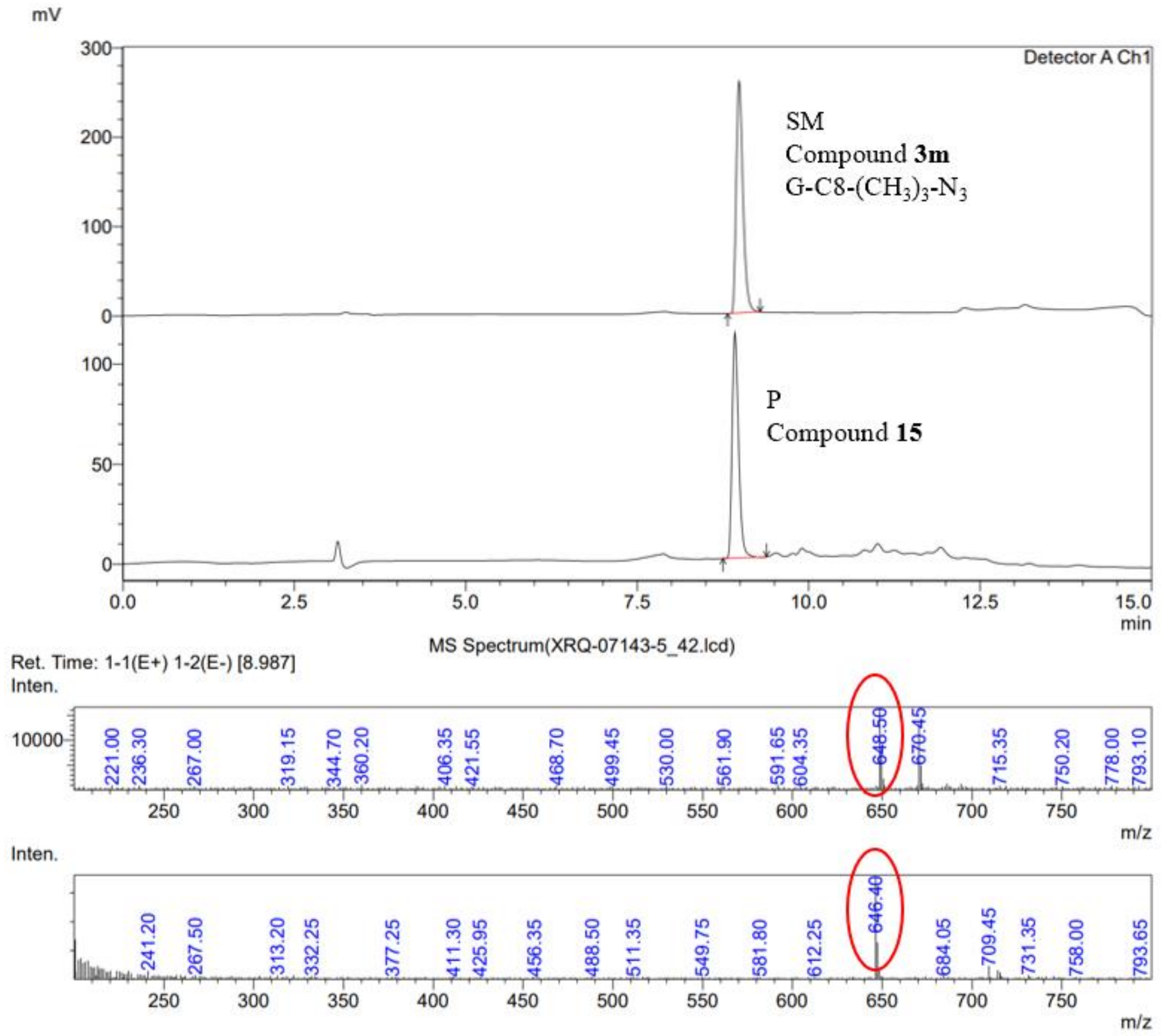

Supplementary Figure 26. HPLC-MS analysis of compound **15.** (A) reverse-phase HPLC traces of compound **15** (C18 Spherical silica), and (B) mass spectrogram of compound **15** (MS-ESI).

7. Microscale reactions with nucleosides/tides and oligonucleotide as substrate

7.1. Reaction setup for nucleosides and dinucleotides

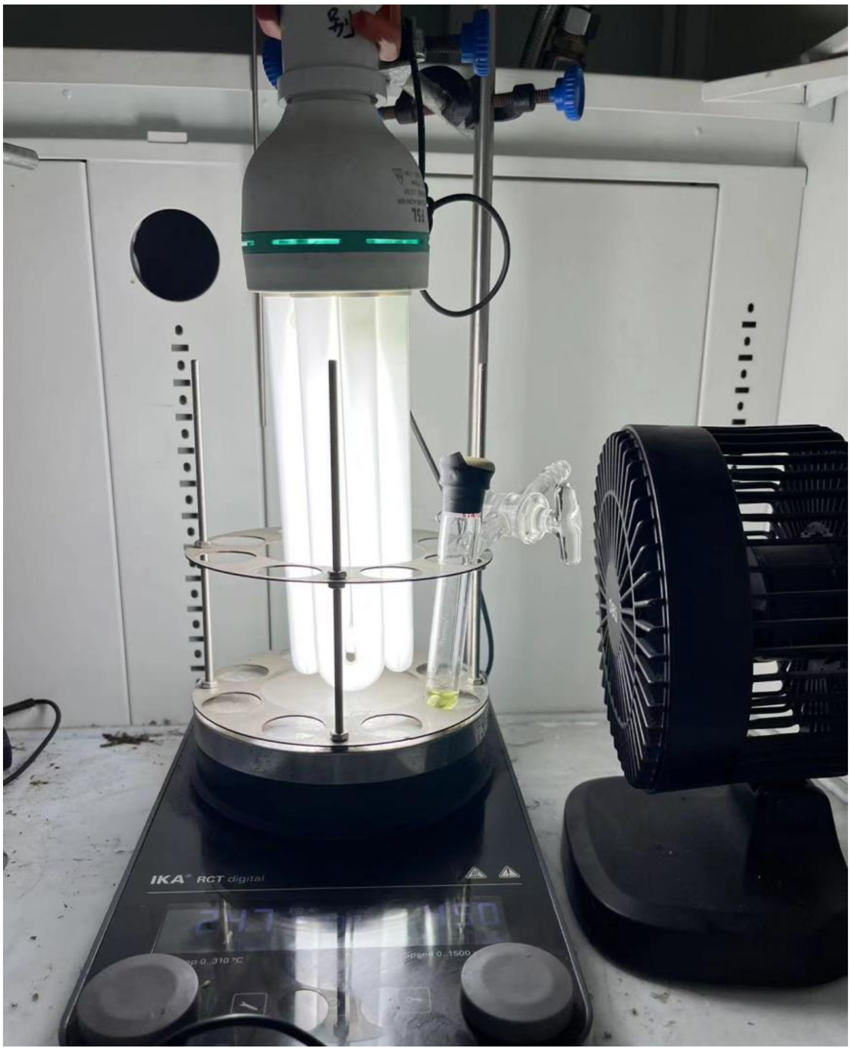

Supplementary Figure 27. Reaction setup of **oligonucleotides**

(a) 5 mL Schlenk tube was used (LH LABWARE). The tube was plugged with a rubber plug and sealed with tape to maintain the satisfactory air tightness.

(b) The vial was irradiated with one 85W white light and was placed about 1-2 cm away from the light. Cooling fan was utilized to maintain the reaction temperature at room temperature.

(c) This device is designed for oligonucleotide reactions that are relatively stable to temperature.

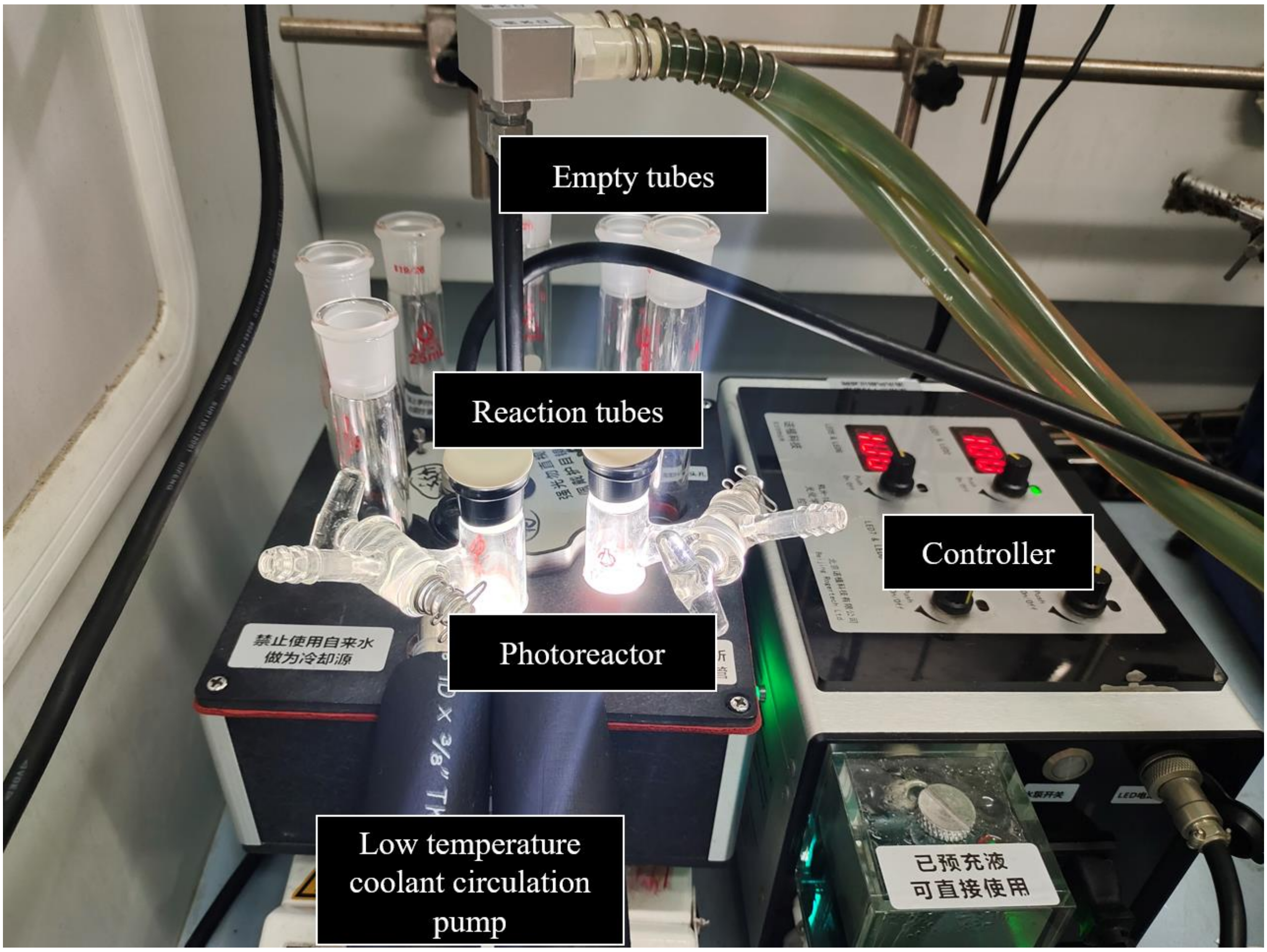

Supplementary Figure 28. Reaction setup of **oligonucleotides**

(a) 10 mL Schlenk tube was used (LH LABWARE). The tube was plugged with a rubber plug and sealed with tape to maintain the satisfactory air tightness.

(b) The vial was irradiated with one 10W **white** LED (ROGER, Multi-channel parallel photocatalytic reaction system). Low temperature coolant circulation pump (BiLon) was utilized to maintain the reaction temperature at about 10 degrees Celsius.

(c) These empty tubes are designed to minimize the accumulation of condensed water in the photoreactor.

(d) This device is designed for oligonucleotide reactions that are relatively **NOT** stable to temperature.

7.2. Optimization of microscale reaction conditions

Supplementary Table 17. Equivalent of the ethylboric acid, catechol, $(NH_4)_2S_2O_8$, TFA, and MesAcr screening. [a,b]

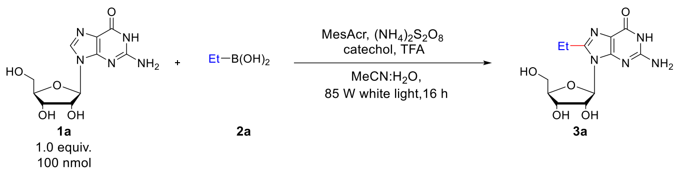

Entry	Et-B(OH)2	Catechol	[O]	TFA	MesAcr	Recovery (%)	Yield (%)
1	**4**	**1**	**2**	**1**	**0.05**	100	0
2	**40**	**10**	**20**	**10**	**0.5**	100	0
3	**200**	**50**	**100**	**50**	**2.5**	51	49
4	**400**	**100**	**200**	**100**	**5**	11	89
5	400	100	200	100	**2.5**	4	96
6	400	100	200	100	**1**	8	92
7	400	100	200	100	**0.5**	5	95
8	400	100	200	100	**0.1**	26	74
9	400	100	200	100	/	65	35
10	400	100	200	**50**	0.5	12	88
11	400	100	200	**25**	0.5	22	78
12	400	100	200	/	0.5	53	47
13	400	100	**100**	50	0.5	16	84
14	400	100	**50**	50	0.5	66	34
15	400	100	**10**	50	0.5	86	14
16	400	100	/	50	0.5	/	/
17	400	**50**	200	50	0.5	45	55
18	400	**0**	200	50	0.5	0	0

19	**200**	100	200	50	0.5	45	55
20	**100**	100	200	50	0.5	100	0
21	**4**	100	200	50	0.5	100	0

[a] Condition: Guanosine (100 nmol), MeCN (0.25 mL), H_2O (0.25 mL), 85 W white light. [b] Yields were determined by LC-MS.

8. Substrate scope study for microscale reactions with nucleosides/tides and oligonucleotide as substrate

General procedure of C8–H alkylation of nucleoside/tide substrates and RNA/DNA oligonucleotide

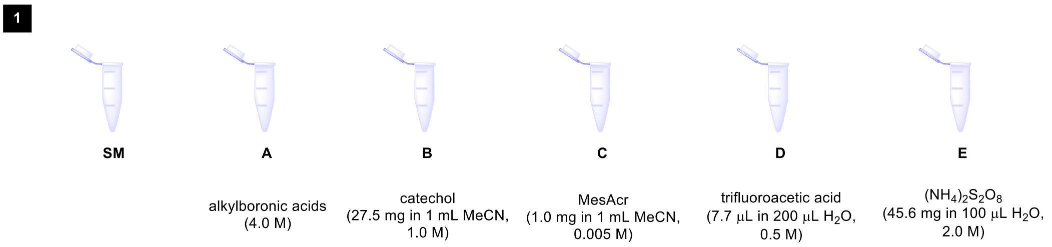

Step 1 and points: Solution preparation:

A: ethylboronic acid (4.0 M).
B: catechol (27.5 mg in 1.0 mL MeCN, 1.0 M).
C: MesAcr (1.0 mg in 1.0 mL MeCN, 0.005 M).
D: trifluoroacetic acid (7.7 μL in 200 μL H_2O, 0.5 M).
E: $(NH_4)_2S_2O_8$ (45.6 mg in 100 μL H_2O, 2.0 M).

System (1): SM: guanosine

SM: 28.6 mg guanosine in 20 mL H_2O (0.005 M)

System (2): SM: oligonucleotides

DEPC water (RNA enzyme and DNA enzyme free) was used instead of H_2O;

SM: dissolving the sub packed RNA/DNA Oligonucleotide (100 nmol) with 90 μ L (30 μ L*3 times) DEPC water.

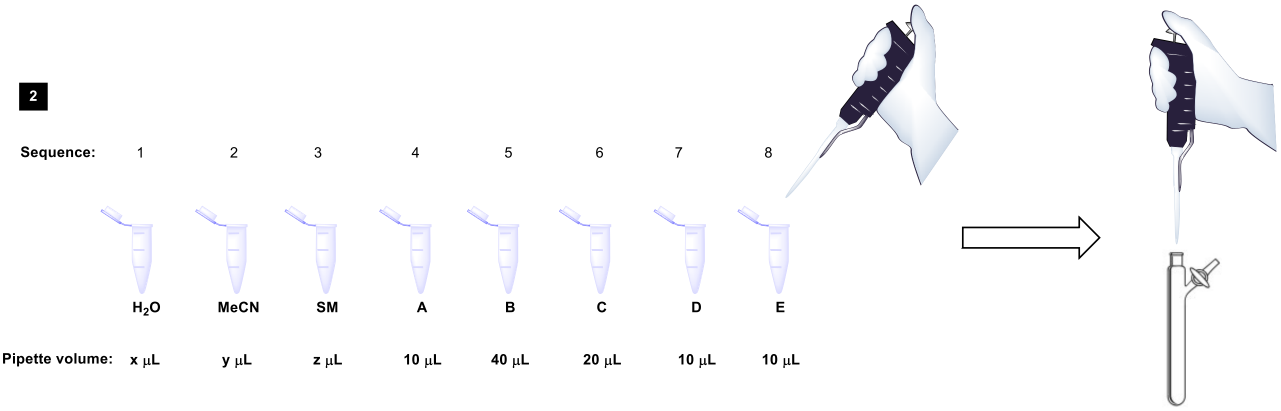

Step 2 and points: the way of adding reagents solution

Sequence: 1) Water for constant volume (**x** μL); **2)** MeCN for constant volume (**y** μL); **3)** A ; **4)** B; **5)** C; **6)** D; **7)** E.

Device: 5 mL Schlenk tube containing a Teflon stir bar, seal with rubber plugs, and wrap with tape to prevent air leakage.

Ensuring the total amount of H_2O (or DEPC water) and acetonitrile is 250 mL respectively in the end.

The time from adding various solvents to freezing and vacuuming the reaction system should not exceed about 10 minutes.

System (1): SM: guanosine

x = 200

y = 190

z = 20

System (2): SM: oligonucleotides

x = 130

y = 190

z = 90

Step 3 and points:

After this, use liquid nitrogen to freeze the reaction solution, use Schlenk Line and oil pump to vacuum the Schlenk tube, and then replace it with inert gas. The time from adding various solvents to freezing and vacuuming the reaction system should not exceed about 10 minutes. The reaction system was exposed to 85 W white light after thawing (as shown in **Supplementary Figure 27**). The yield and recovery of the reaction was detected by LC-MS.

8.1. Substrate scope of C–H alkylation of guanosine and RNA oligonucleotides

Supplementary Table 18. Substrate scope of C–H ethylation of the guanosine and RNA oligonucleotides [a]

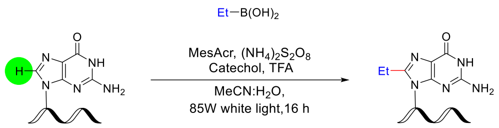

Entry	Oligonucleotides & Sequence	Yield [%] [b]	Calcd. Exact mass	Exptl. m/z (MALDI-FTMS)
1	**Guanosine** G	99 [c]	311.1230	$[M\text{-}H]^-$ = 312.1309 [d]
2	**GpA** 5'-GA-3'	71 [c]	640.1755	$[M\text{+}Na]^-$ = 663.1656 [d]
3	**GpG** 5'-GG-3'	59 [c]	684.2017	$[M\text{-}H]^-$ = 683.1948 [d]
4	**GpC** 5'-GC-3'	66 [c]	616.1643	$[M\text{-}H]^-$ = 615.1554 [d]
5	**ON 1** 5'-GUUUCC-3'	78	1839.2814	$[M\text{+}H]^+$ = 1840.31214
6	**ON 2** 5'-UUUGCC-3'	71	1839.2814	$[M\text{+}H]^+$ = 1840.28383
7	**ON 3** 5'-UUUCCG-3'	80	1839.2814	$[M\text{+}H]^+$ = 1840.29030
8	**ON 4** 5'-GUUACC-3'	78	1862.3087	$[M\text{+}H]^+$ = 1863.31278
9	**ON 5** 5'-AUUGCC-3'	69	1862.3087	$[M\text{+}H]^+$ = 1863.31867
10	**ON 6** 5'-GCUAUCU-3'	63	2168.3340	$[M\text{+}H]^+$ = 2169.36146
11	**ON 7** 5'-GUUACCUU-3'	73	2474.3593	$[M\text{+}H]^+$ = 2475.35981
12	**ON 8** 5'-GUUACCUCU-3'	74	2779.4006	$[M\text{+}H]^+$ = 2780.42431

[a] Condition: Oligonucleotide (100 nmol), ethylboronic acid (400.0 equiv.), MesAcr (50 mol%), $(NH_4)_2S_2O_8$ (200.0 equiv.), TFA (50.0 equiv.), and catechol (100.0 equiv.) in MeCN (0.25 mL), H_2O (0.25 mL), irradiated by 85 W white light at r.t. for 16 h. [b] Yields were determined by LC-MS and used the analytical method B. [c] Yields were determined by LC-MS and used the analytical method A. [d] The mass data was collected by using HRMS.

RP-HPLC profiles of RNA oligonucleotides

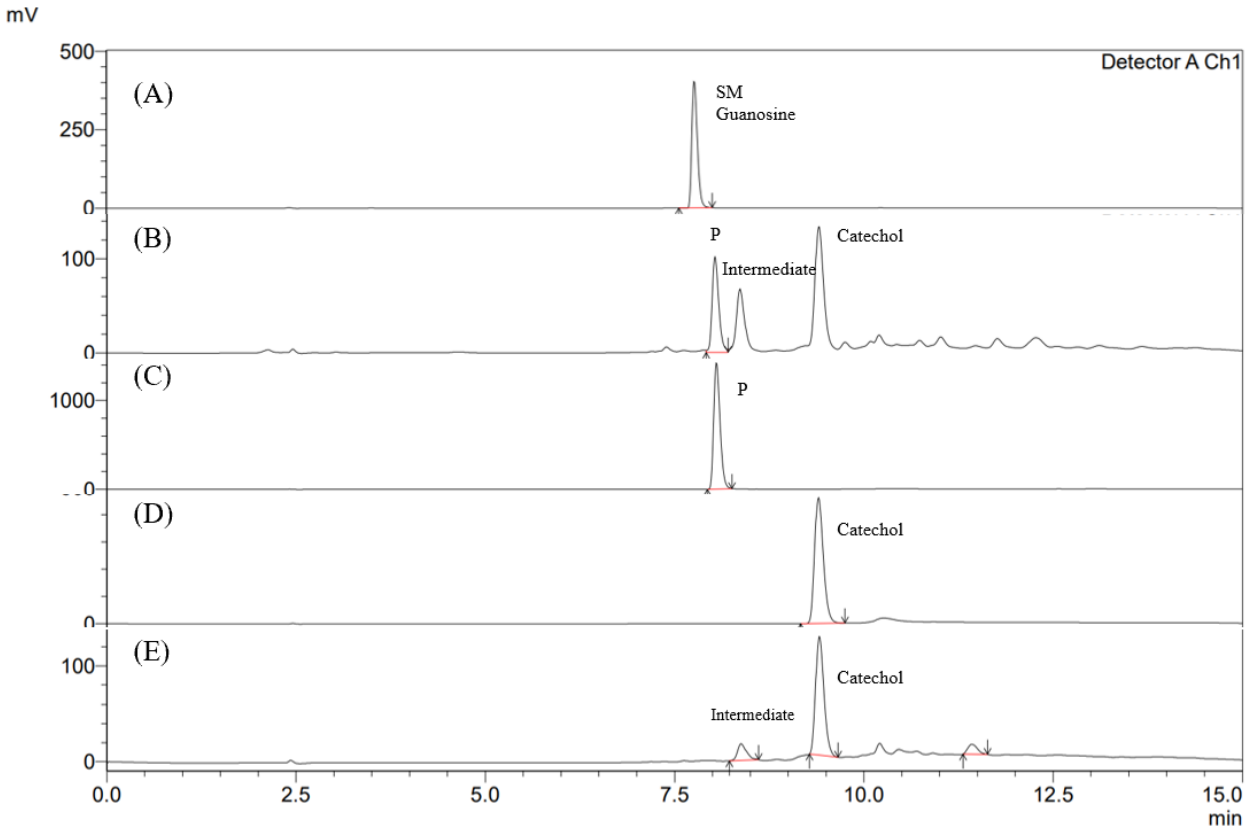

Supplementary Figure 29. Reverse-phase HPLC traces of (A) the starting material (Guanosine), (B) the reaction mixture following catechol-promoted photoredox C–H alkylation of Guanosine with ethyl boronic acid, (C) the corresponding ethylated product was used as a standard sample, (D) the catechol, and (E) the reaction system is the absence of the starting material.

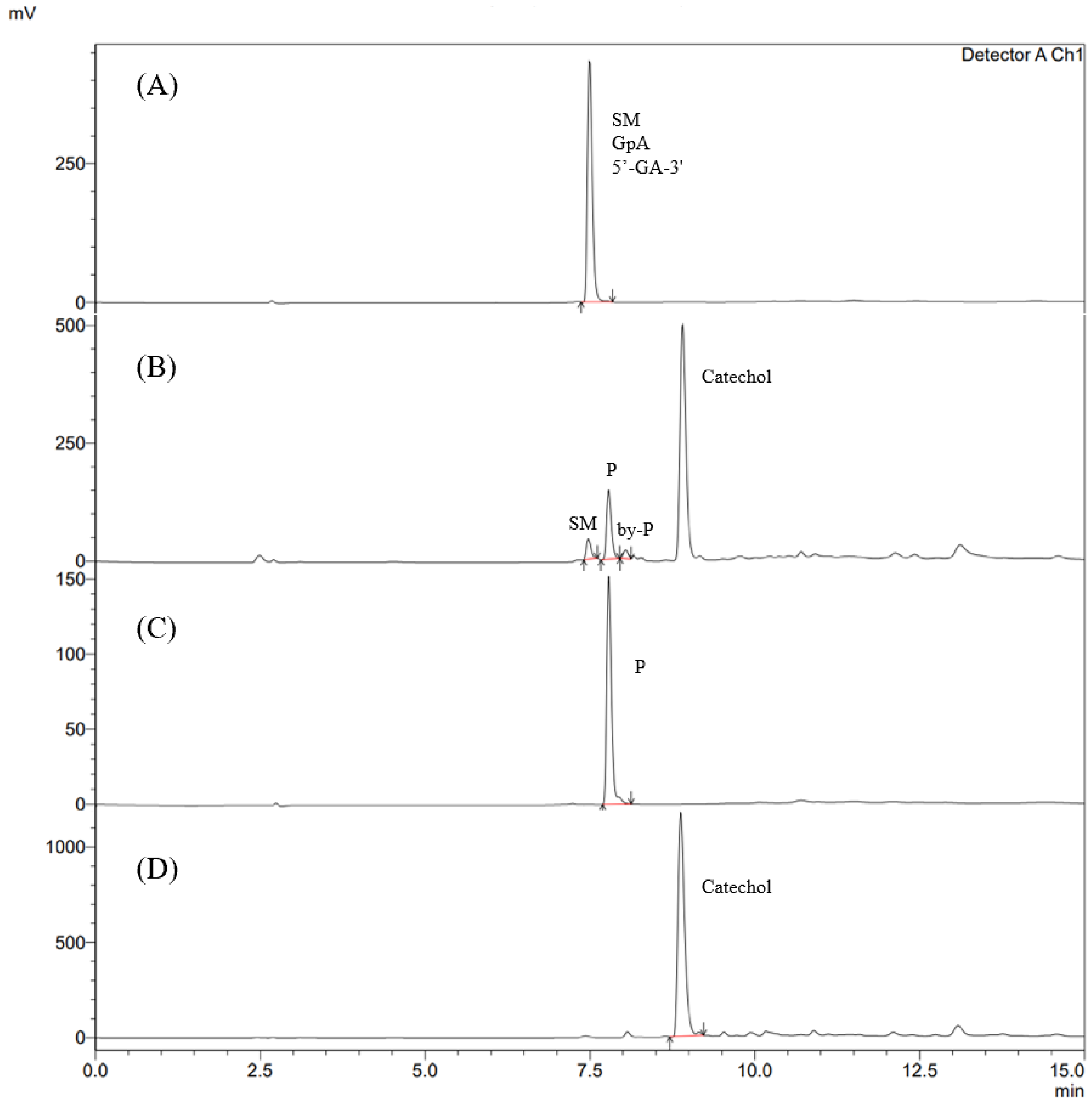

Supplementary Figure 30. Reverse-phase HPLC traces of (A) the starting material (GpA), (B) the reaction mixture following catechol-promoted photoredox C–H alkylation of GpA with ethyl boronic acid, (C) the corresponding ethylated product was used as a standard sample, and (D) the catechol.

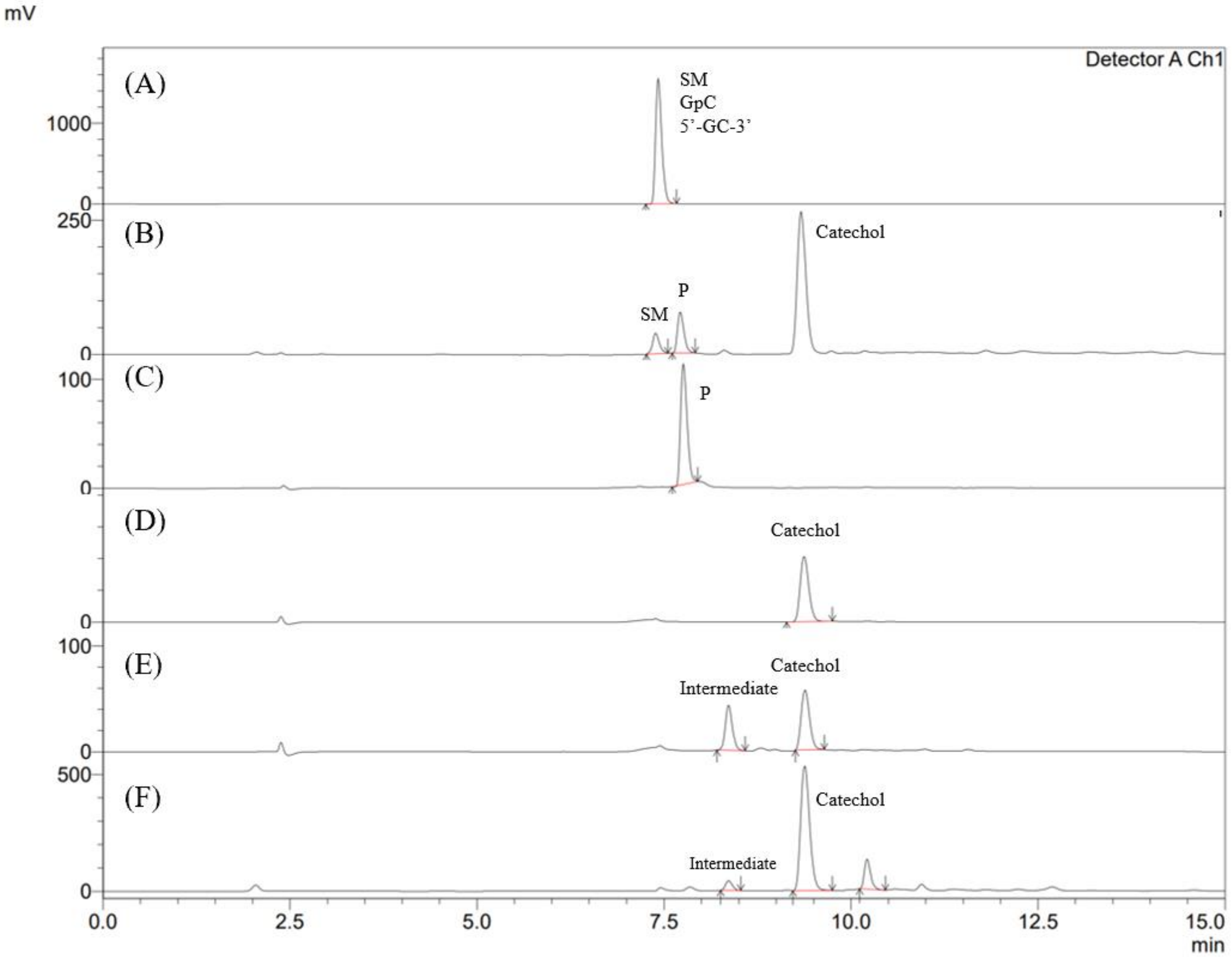

Supplementary Figure 31. Reverse-phase HPLC traces of (A) the starting material (GpC), (B) the reaction mixture following catechol-promoted photoredox C–H alkylation of GpC with ethyl boronic acid, (C) the corresponding ethylated product was used as a standard sample, (D) the catechol, (E) the catechol and the intermediate that prepared by mixing the catechol and ethyl boronic acid, and (F) the reaction system is the absence of the starting material.

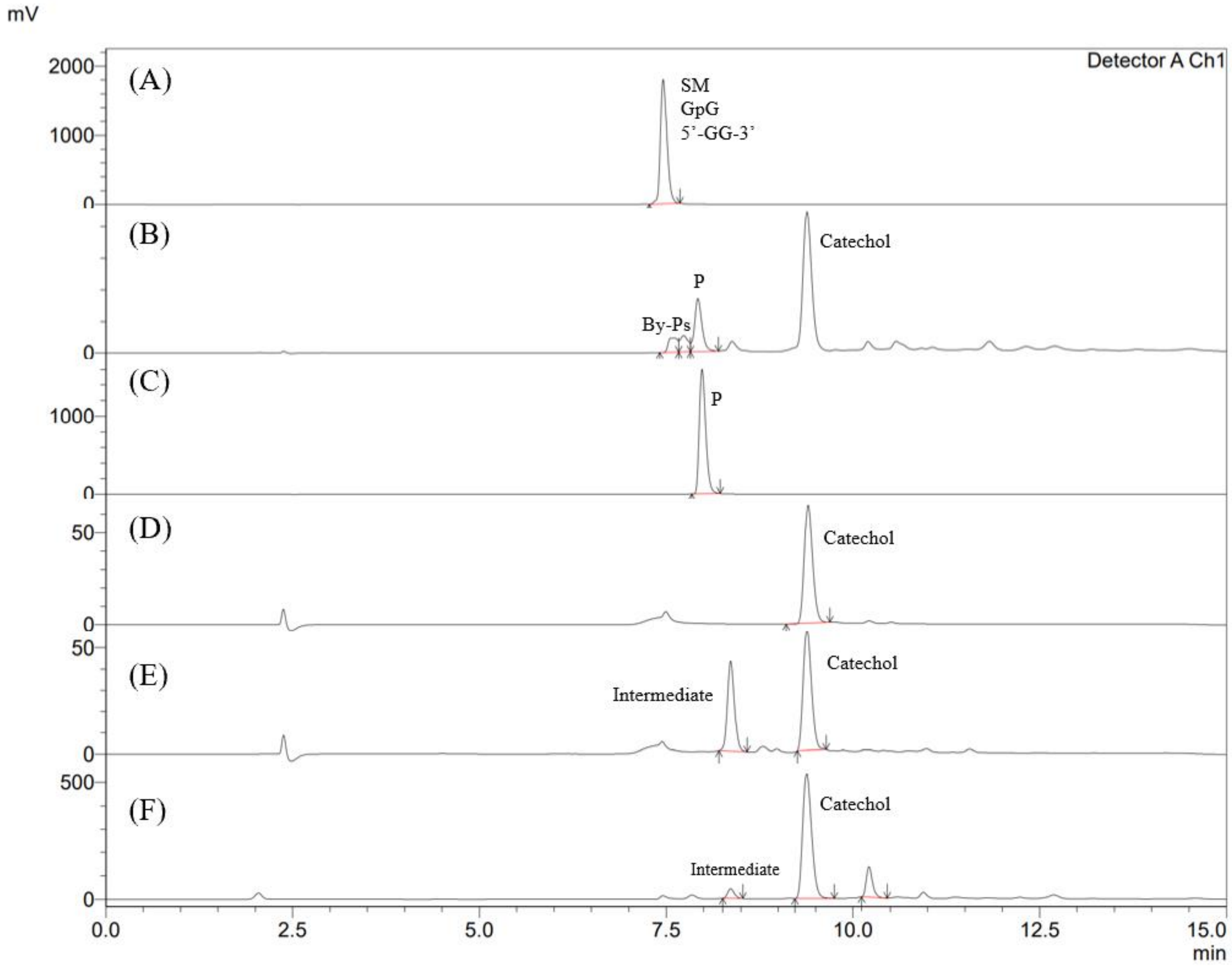

Supplementary Figure 32. Reverse-phase HPLC traces of (A) the starting material (GpG), (B) the reaction mixture following catechol-promoted photoredox C–H alkylation of GpG with ethyl boronic acid, (C) the corresponding ethylated product was used as a standard sample, (D) the catechol, (E) the catechol and the intermediate that prepared by mixing the catechol and ethyl boronic acid, and (F) the reaction system is the absence of the staringt material.

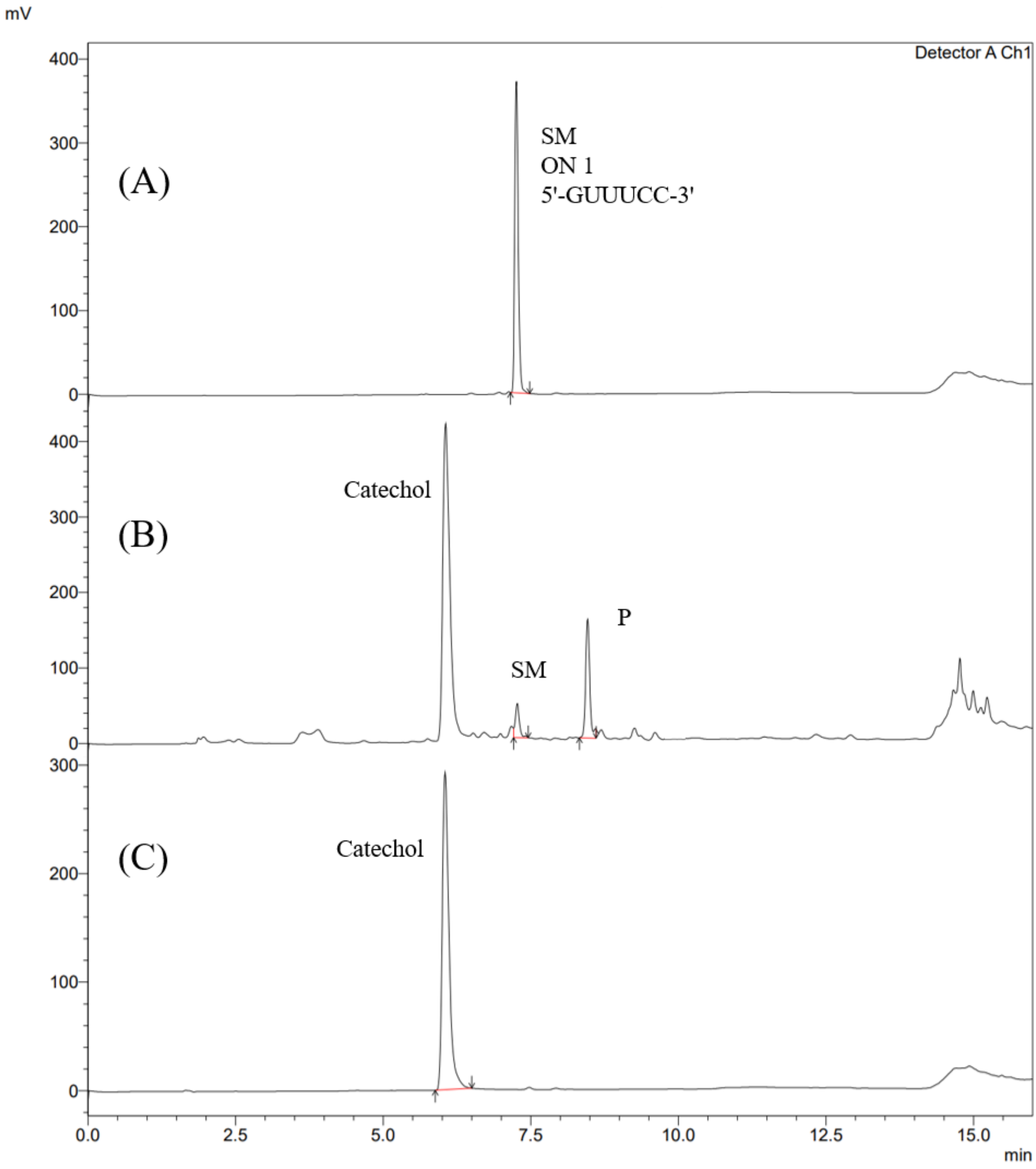

Supplementary Figure 33. Reverse-phase HPLC traces of (A) **ON 1**_a prepared by solid-phase RNA synthesis, (B) reaction mixture following catechol-promoted photoredox C–H alkylation of **ON 1** with ethyl boronic acids, (C) the catechol, and (E) the catechol and the intermediate that prepared by mixing the catechol and ethyl boronic acid.

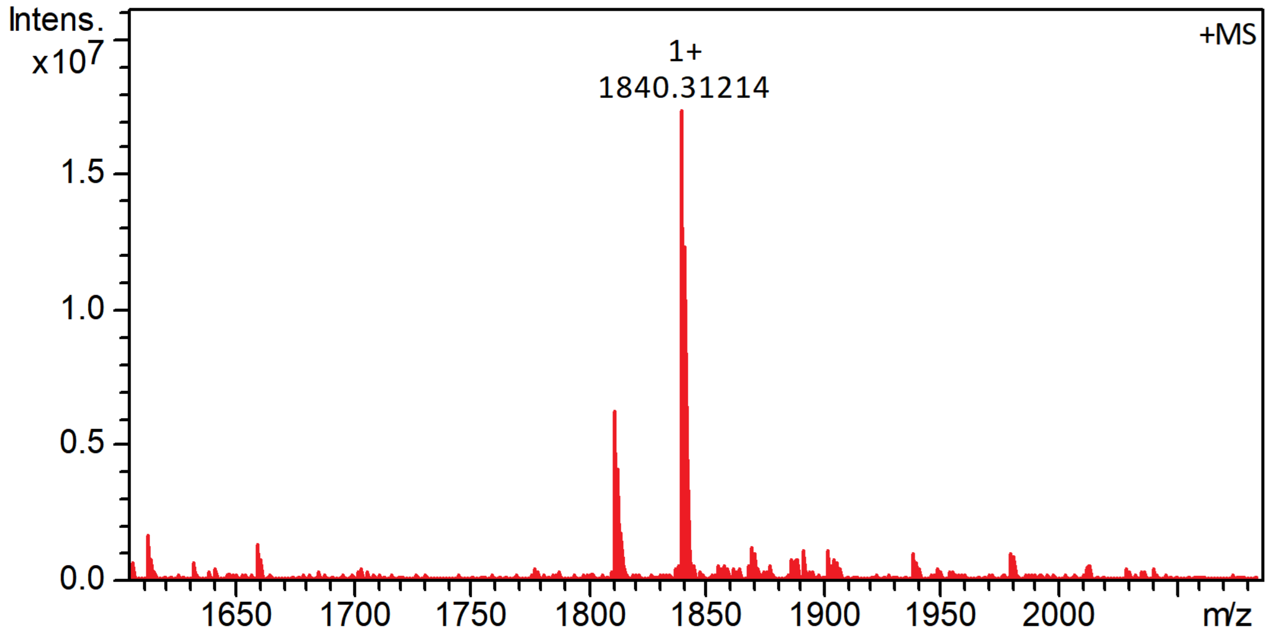

Supplementary Figure 34. Identification of G modification by MALDI-FTMS analysis of the reaction system of **ON 1**. Mass spectrum (MS) of desire product after ethylation of **ON 1**.

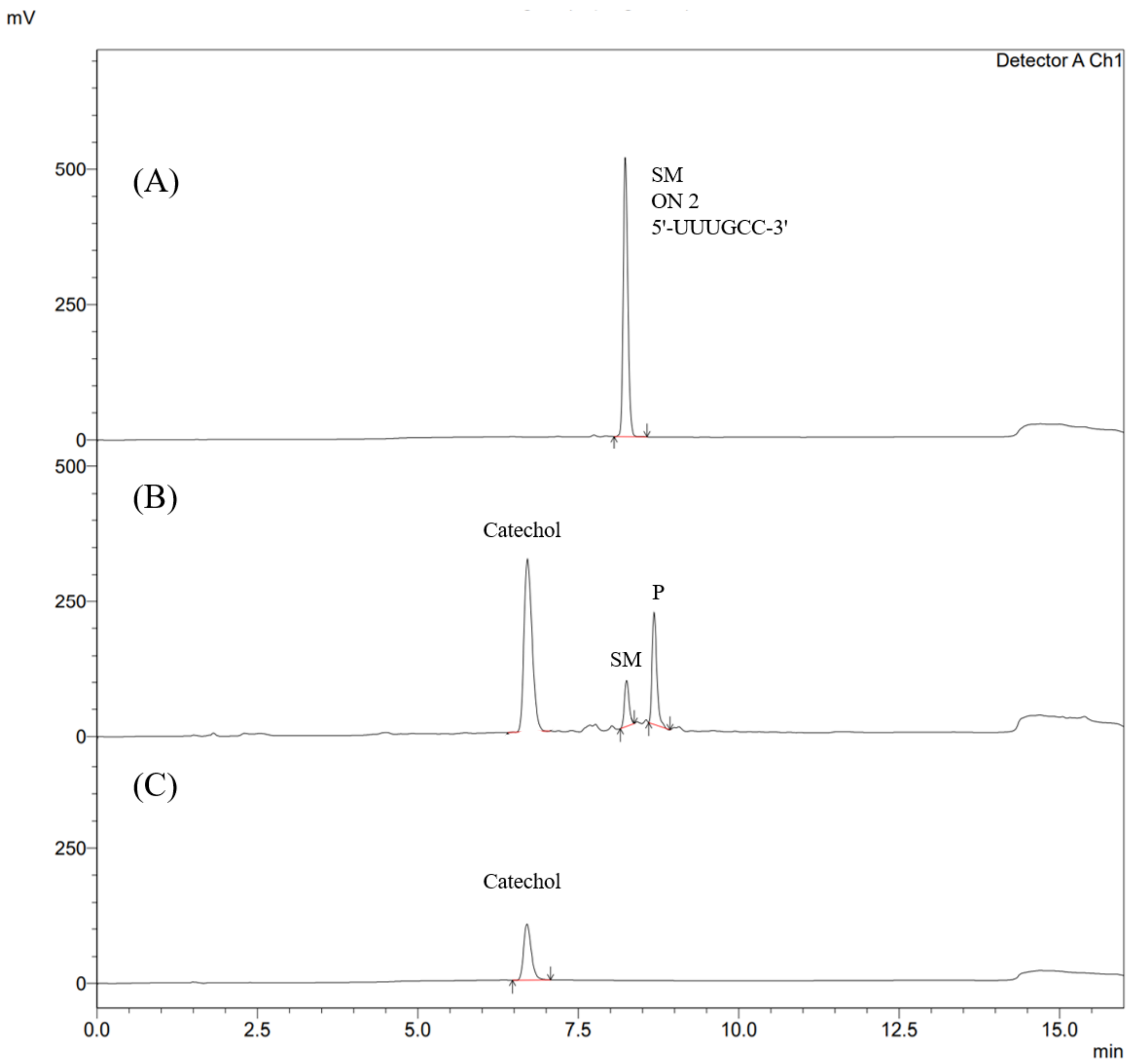

Supplementary Figure 35. Reverse-phase HPLC traces of (A) **ON 2**_a prepared by solid-phase RNA synthesis, (B) reaction mixture following catechol-promoted photoredox C–H alkylation of **ON 2** with ethyl boronic acids, and (C) the catechol.

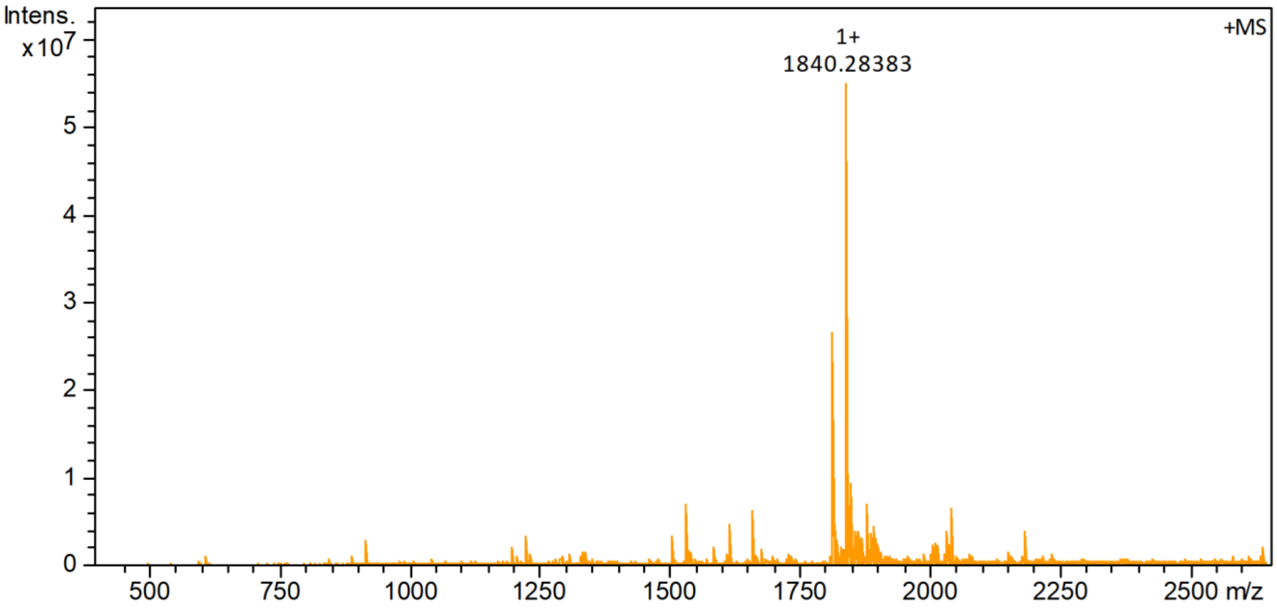

Supplementary Figure 36. Identification of G modification by MALDI-FTMS analysis of the reaction system of **ON 2**. Mass spectrum (MS) of desire product after ethylation of **ON 2**.

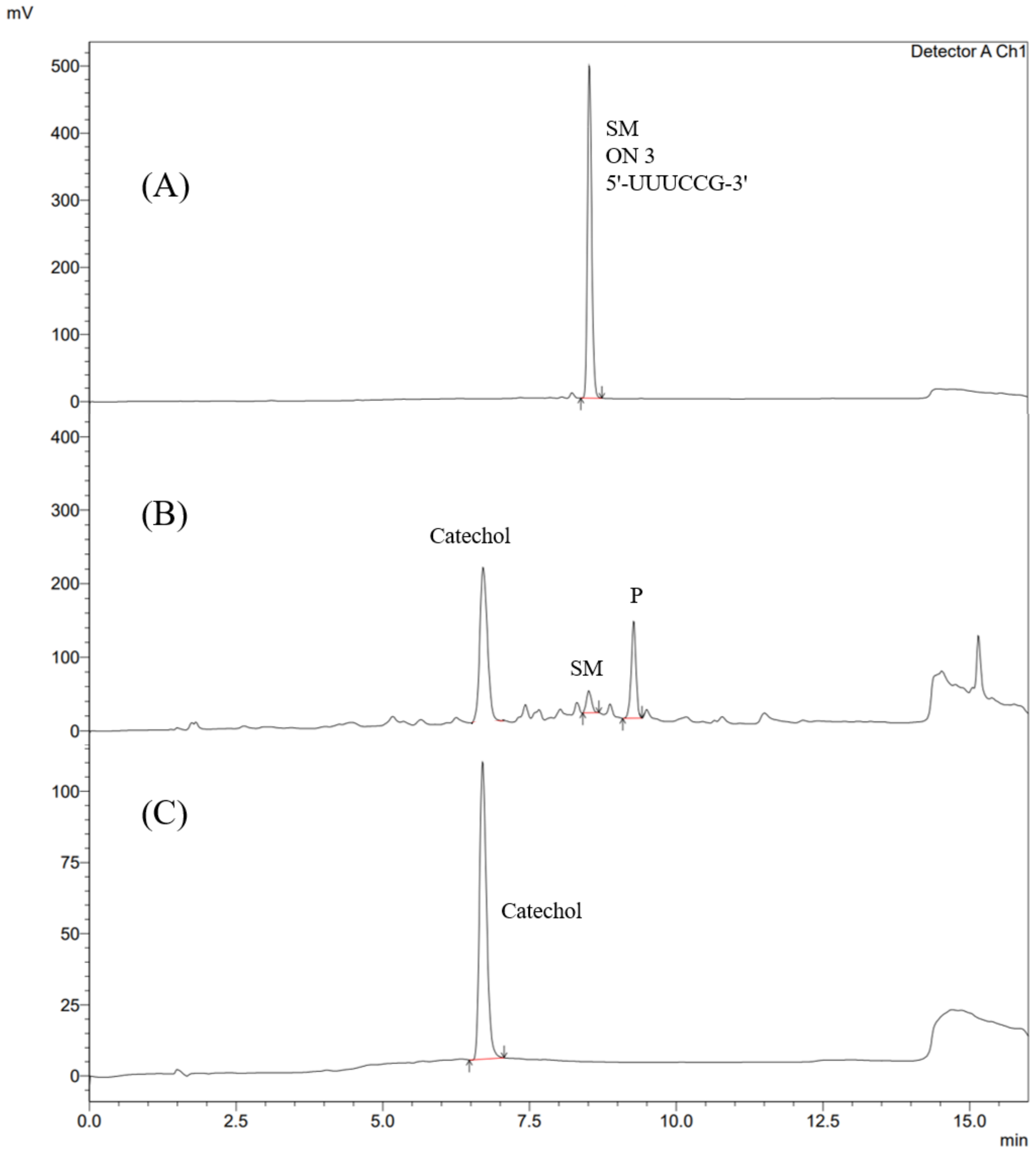

Supplementary Figure 37. Reverse-phase HPLC traces of (A) **ON 3**_a prepared by solid-phase RNA synthesis, (B) reaction mixture following catechol-promoted photoredox C–H alkylation of **ON 3** with ethyl boronic acids, and (C) the catechol.

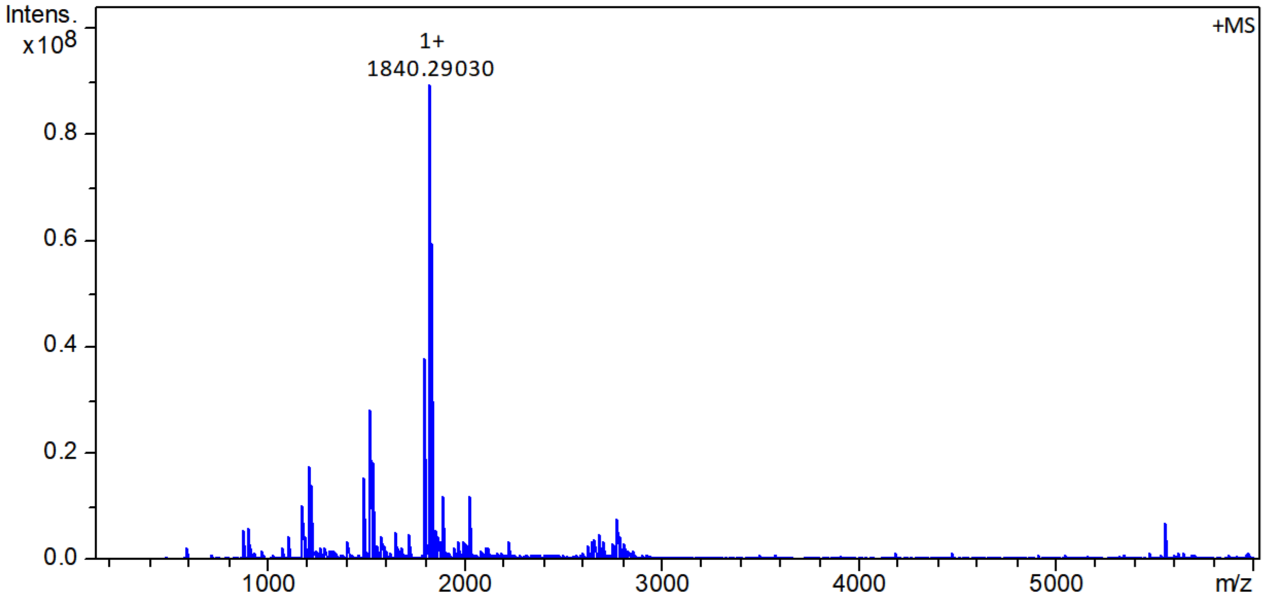

Supplementary Figure 38. Identification of G modification by MALDI-FTMS analysis of the reaction system of **ON 3**. Mass spectrum (MS) of desire product after ethylation of **ON 3**.

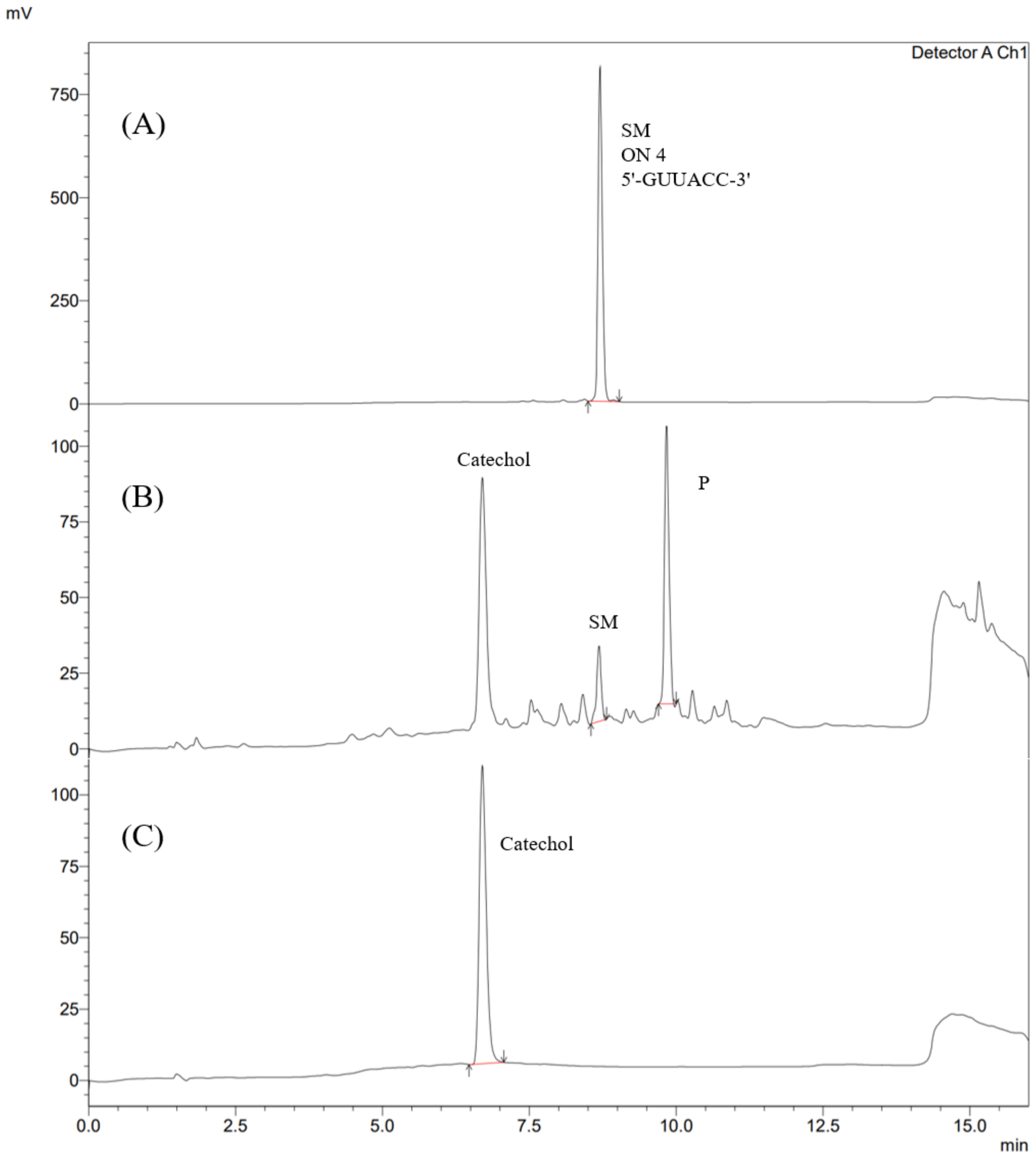

Supplementary Figure 39. Reverse-phase HPLC traces of (A) **ON 4**_a prepared by solid-phase RNA synthesis, (B) reaction mixture following catechol-promoted photoredox C–H alkylation of **ON 4** with ethyl boronic acids, and (C) the catechol.

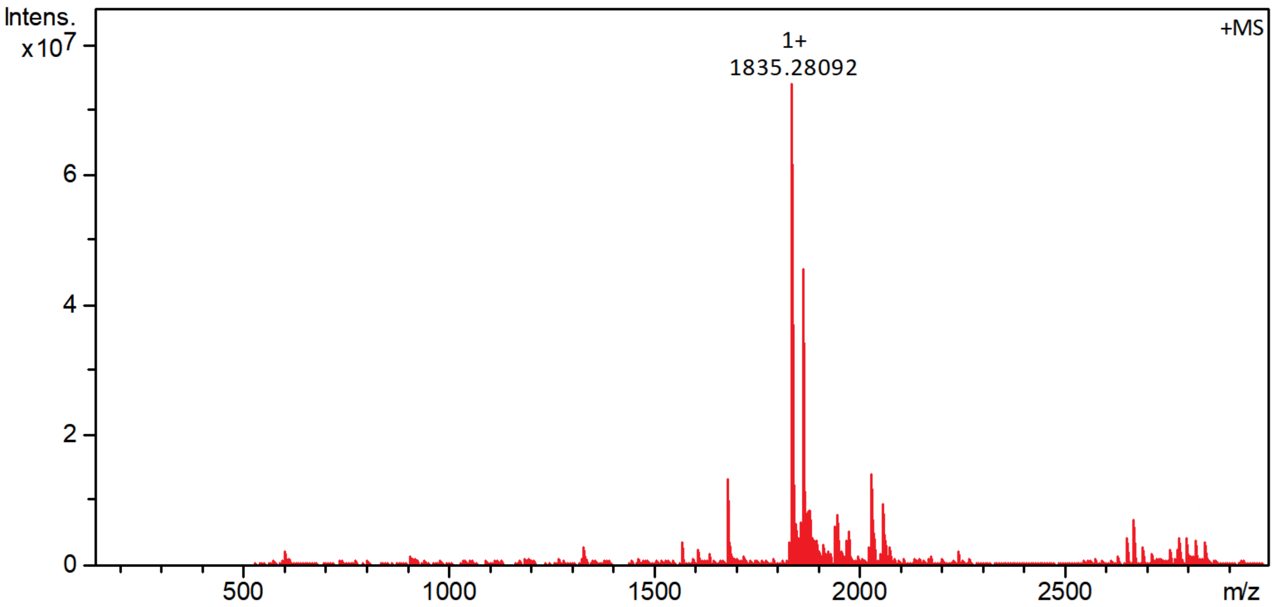

Supplementary Figure 40. Identification of G modification by MALDI-FTMS analysis of the reaction system of **ON 4**. Mass spectrum (MS) of desire product after ethylation of **ON 4**.

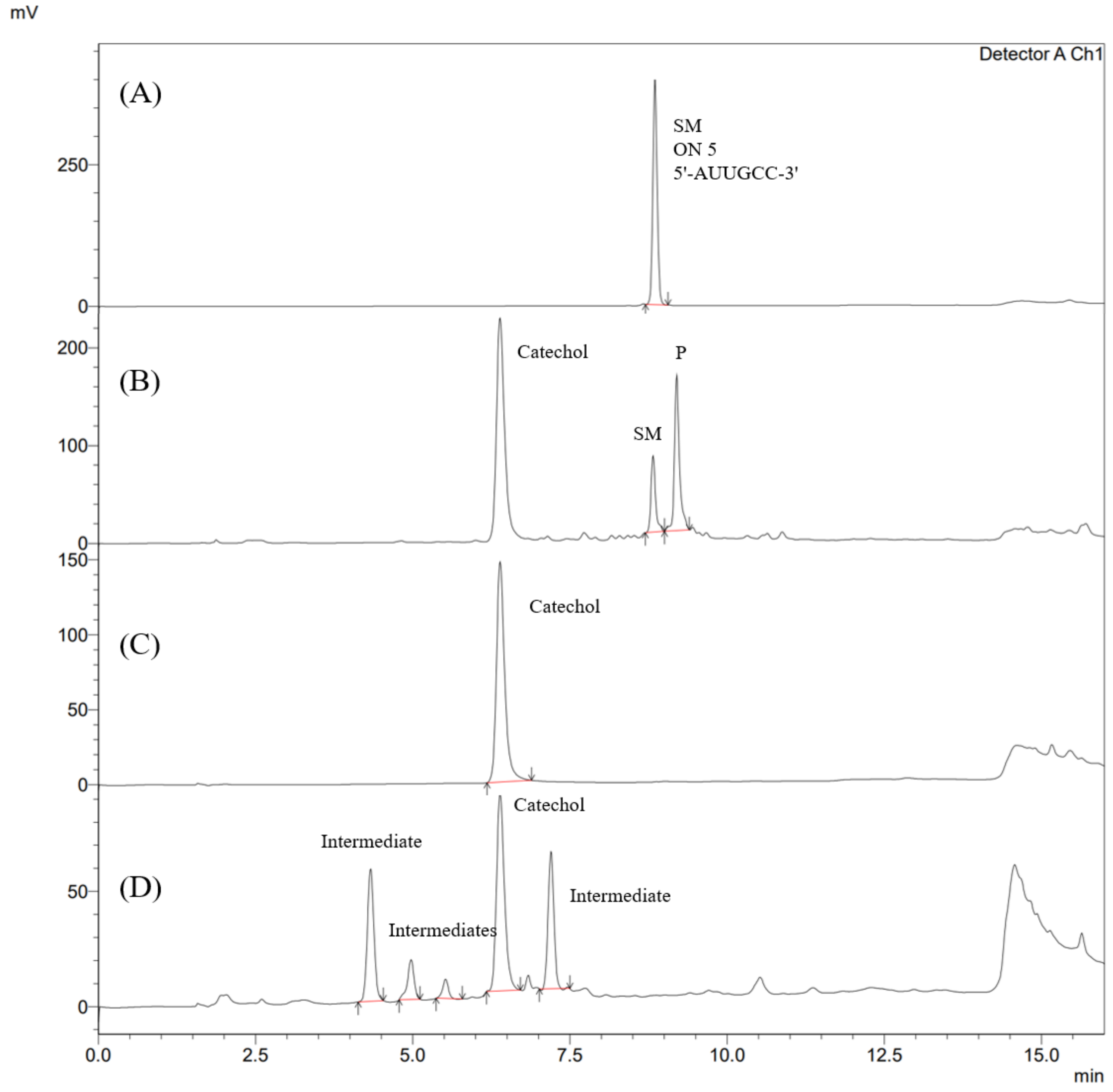

Supplementary Figure 41. Reverse-phase HPLC traces of (A) **ON 5**_a prepared by solid-phase RNA synthesis, (B) reaction mixture following catechol-promoted photoredox C–H alkylation of **ON 5** with ethyl boronic acids, (C) the catechol, and (D) the catechol and the intermediates that prepared by mixing the catechol and ethyl boronic acid.

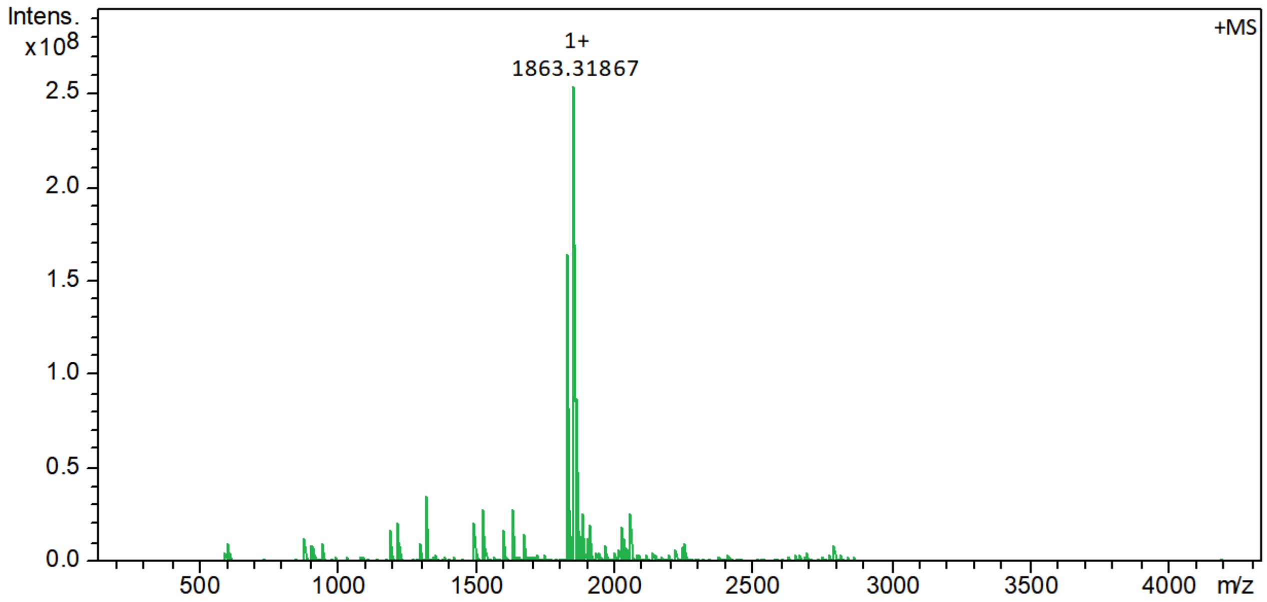

Supplementary Figure 42. Identification of G modification by MALDI-FTMS analysis of the reaction system of **ON 5**. Mass spectrum (MS) of desire product after ethylation of **ON 5**.

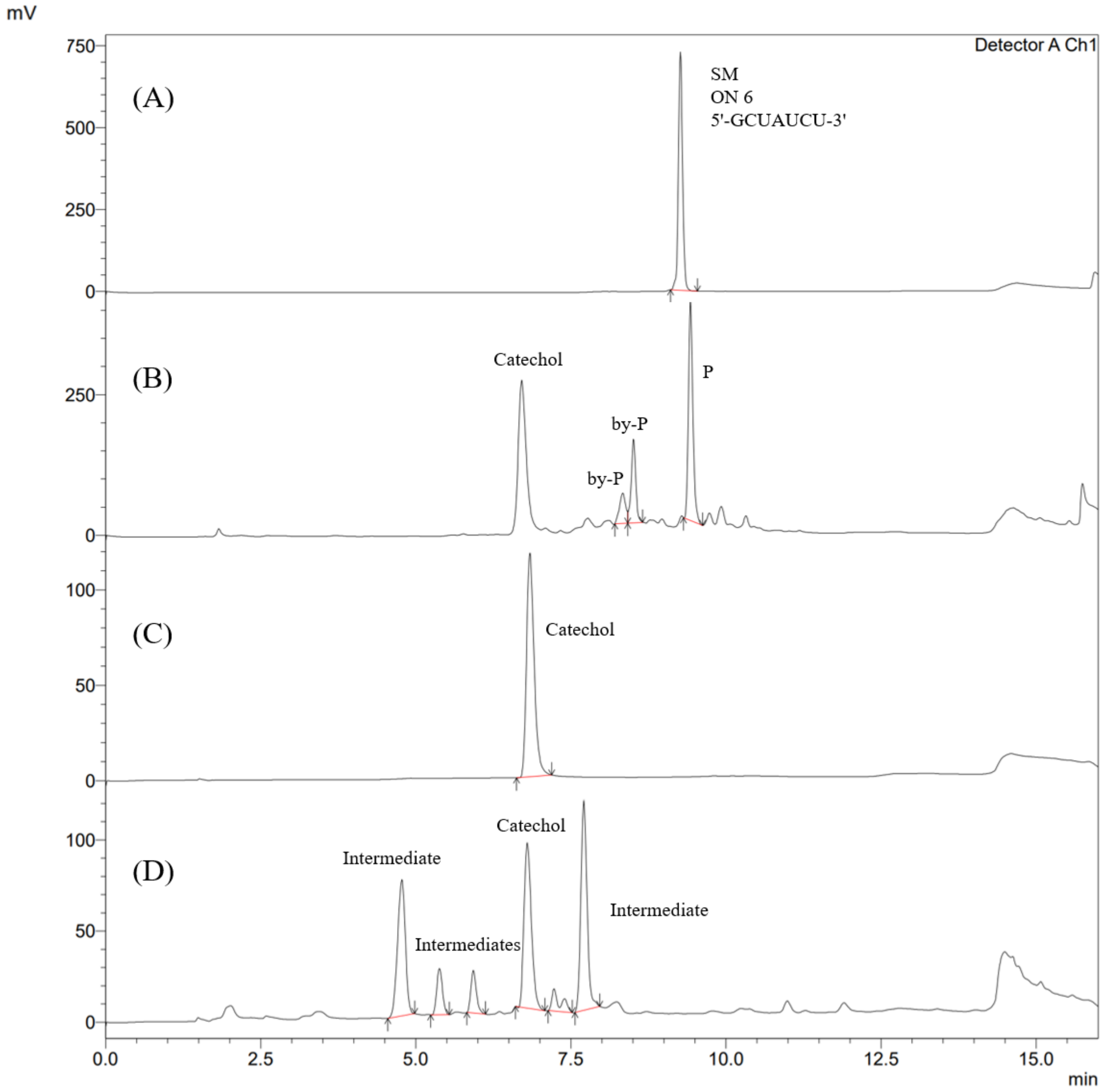

Supplementary Figure 43. Reverse-phase HPLC traces of (A) **ON 6**_a prepared by solid-phase RNA synthesis, (B) reaction mixture following catechol-promoted photoredox C–H alkylation of **ON 6** with ethyl boronic acids, (C) the catechol, and (D) the catechol and the intermediate that prepared by mixing the catechol and ethyl boronic acid.

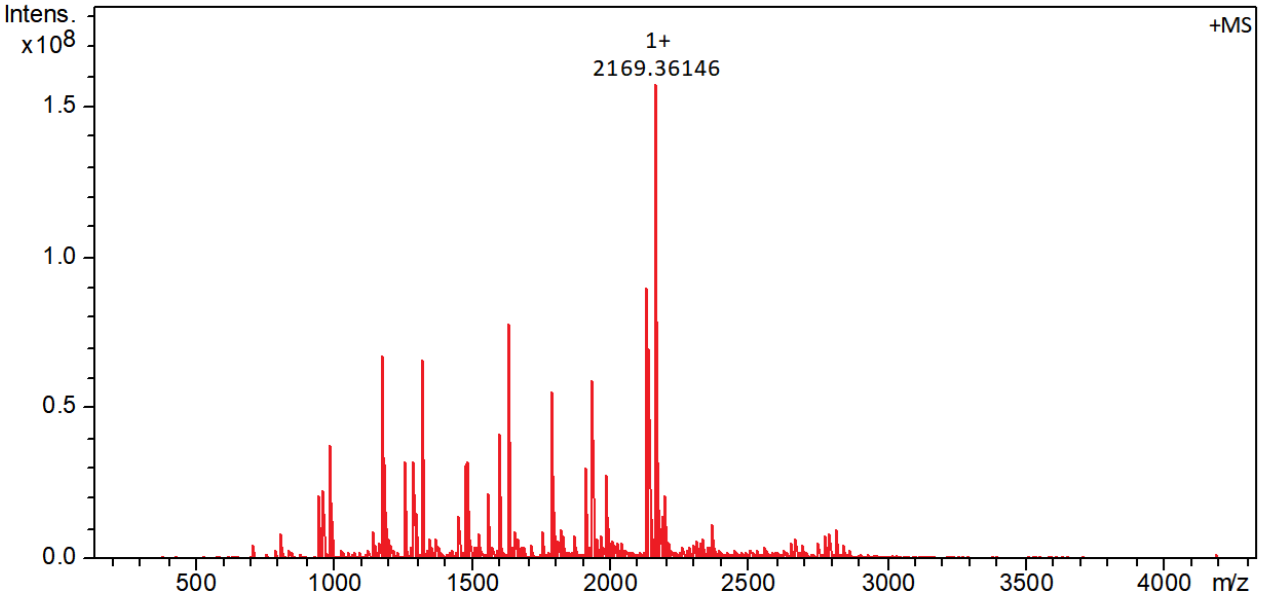

Supplementary Figure 44. Identification of G modification by MALDI-FTMS analysis of the reaction system of **ON 6**. Mass spectrum (MS) of desire product after ethylation of **ON 6**.

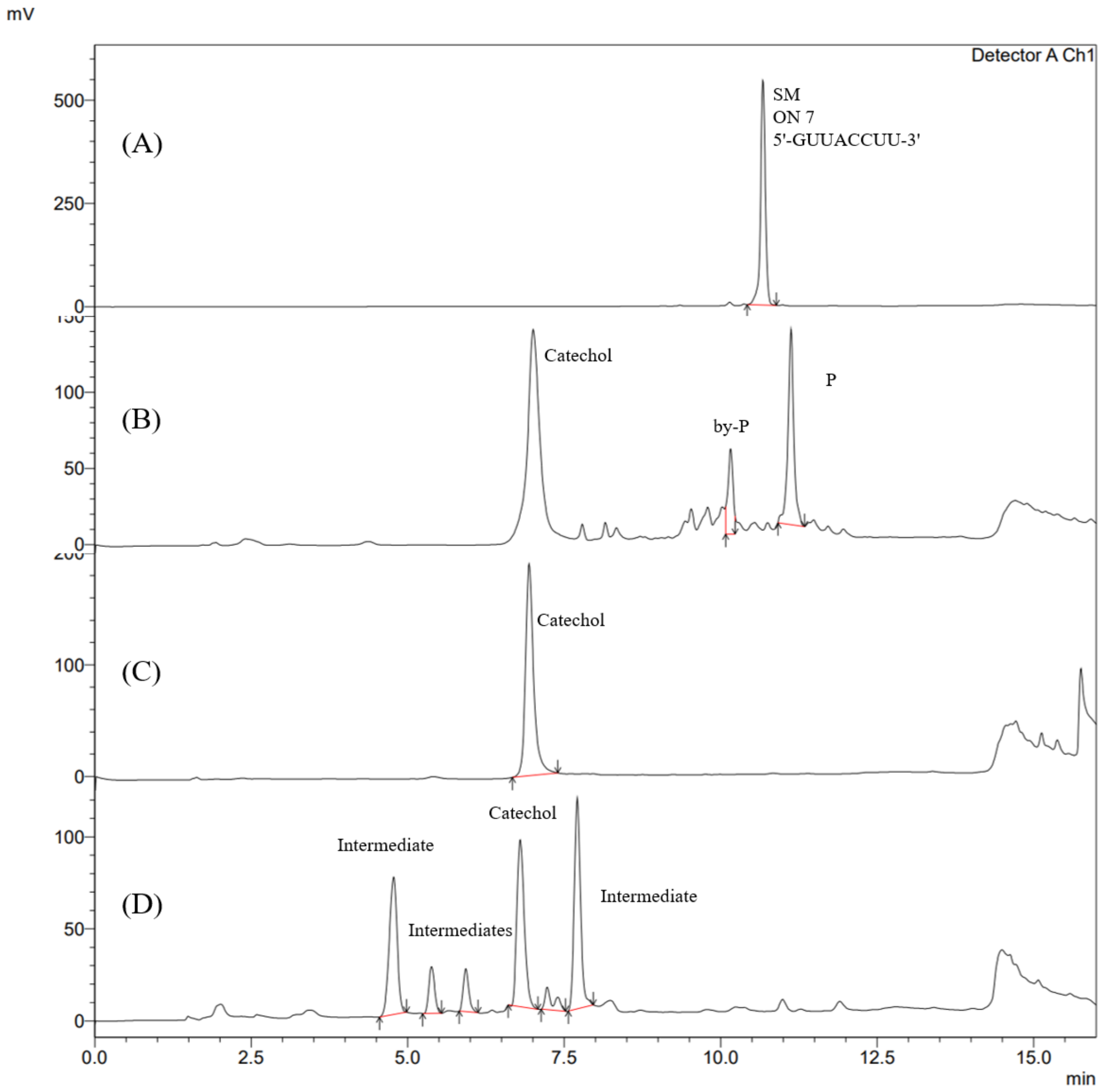

Supplementary Figure 45. Reverse-phase HPLC traces of (A) **ON 7**_a prepared by solid-phase RNA synthesis, (B) reaction mixture following catechol-promoted photoredox C–H alkylation of **ON 7** with ethyl boronic acids, (C) the catechol, and (D) the catechol and the intermediate that prepared by mixing the catechol and ethyl boronic acid.

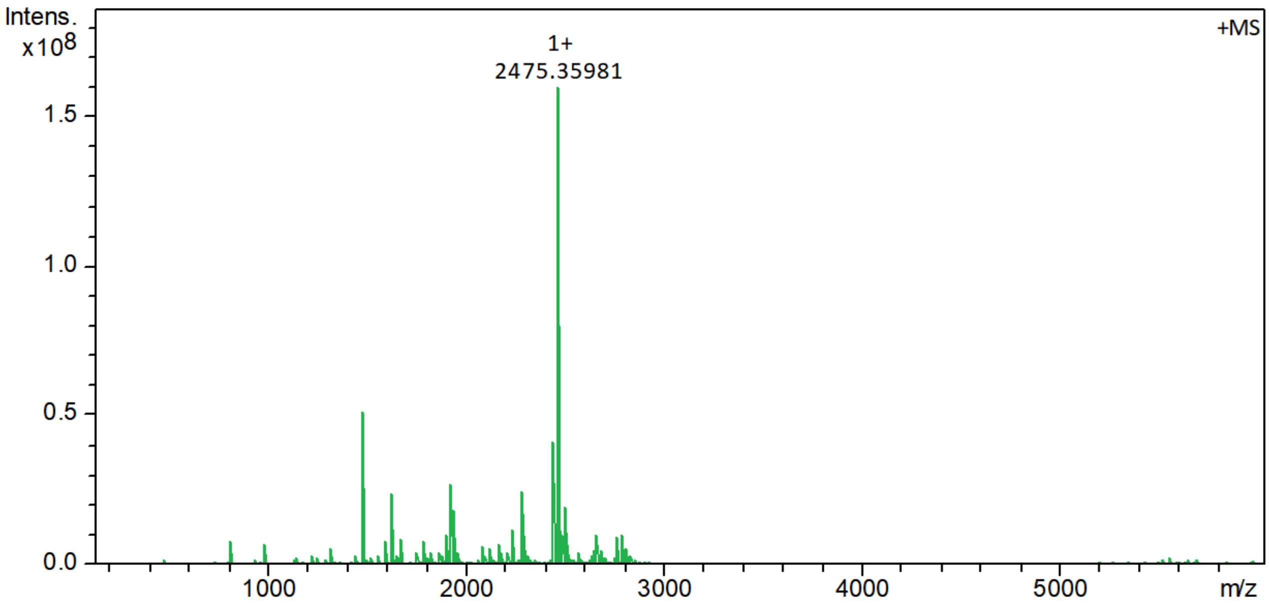

Supplementary Figure 46. Identification of G modification by MALDI-FTMS analysis of the reaction system of **ON 7**. Mass spectrum (MS) of desire product after ethylation of **ON 7**.

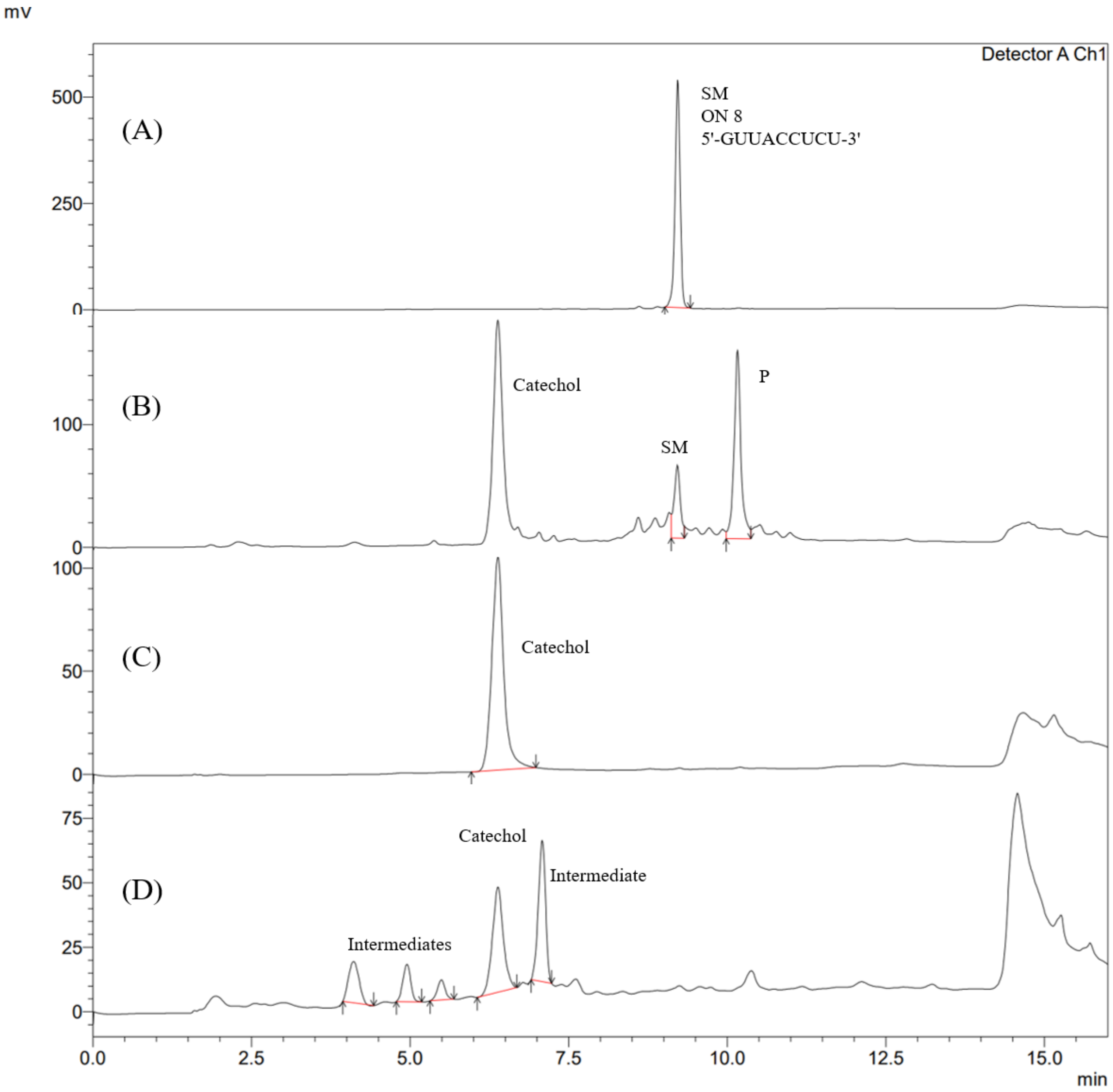

Supplementary Figure 47. Reverse-phase HPLC traces of (A) **ON 8**_a prepared by solid-phase RNA synthesis, (B) reaction mixture following catechol-promoted photoredox C–H alkylation of **ON 8** with ethyl boronic acids, (C) the catechol, and (D) the catechol and the intermediate that prepared by mixing the catechol and ethyl boronic acid.

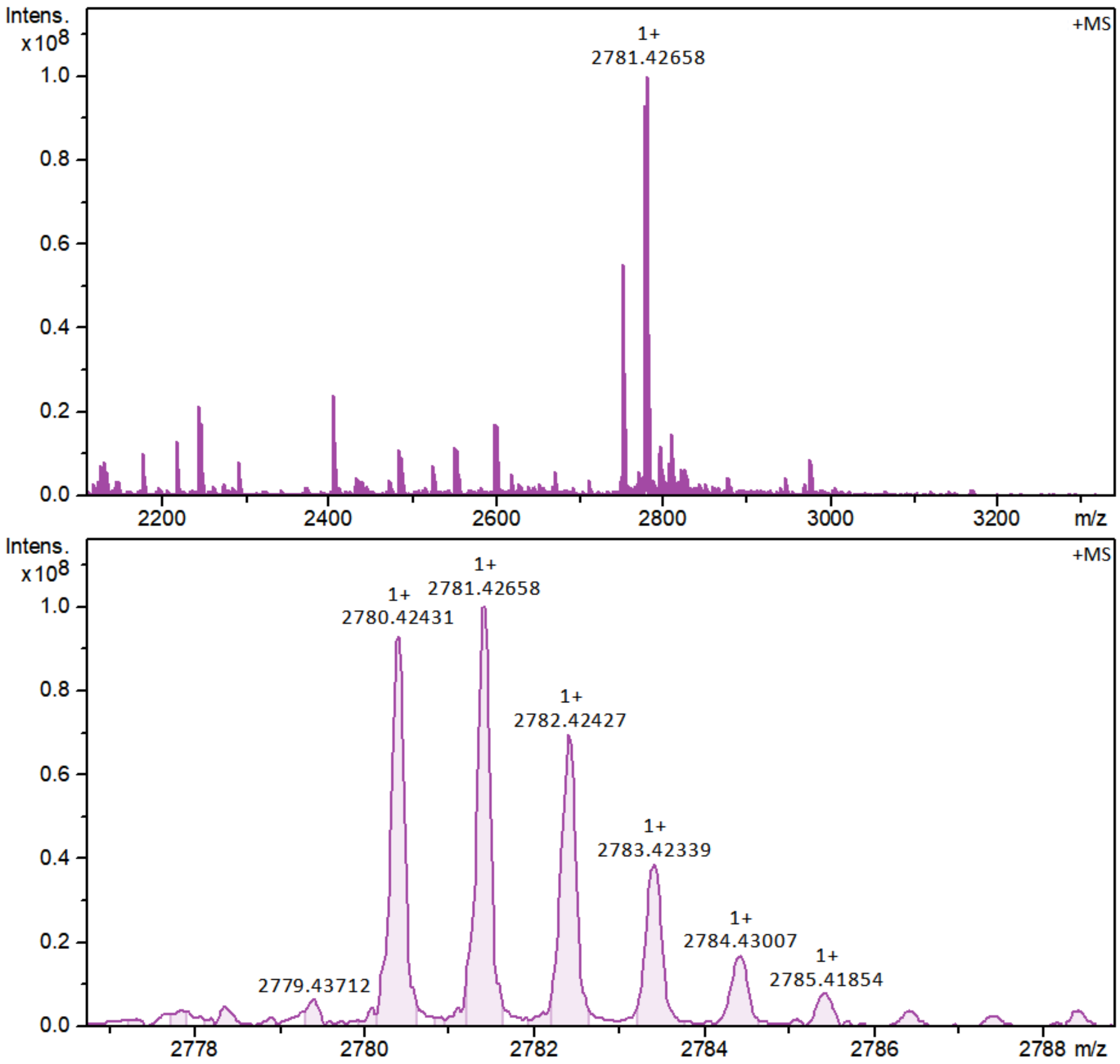

Supplementary Figure 48. Identification of G modification by MALDI-FTMS analysis of the reaction system of **ON 8**. Mass spectrum (MS) of desire product after ethylation of **ON 8**.

Supplementary Table 19. Substrate scope of C–H alkylation of the RNA oligonucleotides with diverse types of free radical precursors

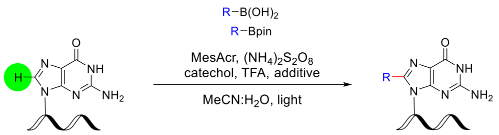

Entry	Oligonucleotides & Sequence	Yield [%]	Free radical precursors	Calcd. Exact mass	Exptl. m/z (MALDI-FTMS)
**1** [a]	**ON 5** 5'-AUUGCC-3'	59 [e]	*n*Pr-B(OH)$_2$	1876.3243	$[M+H]^+$ = 1877.31866
**2** [b]	**ON 5** 5'-AUUGCC-3'	31 [e]	$N_3(CH_2)_3$-Bpin	1917.3257	$[M+H]^+$ = 1918.30266
**3** [c]	**ON 5** 5'-AUUGCC-3'	57 [e]	Cyclobutyl-B(OH)$_2$	1888.3243	$[M+H]^+$ = 1889.30149
**4** [d]	**ON 5** 5'-AUUGCC-3'	63 [f]	Cyclopentyl-B(OH)$_2$	1902.3400	$[M+H]^+$ = 1903.33673

[a] Condition: Oligonucleotide (100 nmol), alkylboronic acid (400.0 equiv.), MesAcr (50 mol%), $(NH_4)_2S_2O_8$ (200.0 equiv.), TFA (50.0 equiv.), and catechol (100.0 equiv.) in MeCN (0.25 mL), H_2O (0.25 mL), irradiated by 85 W white light at r.t. for 24 h. [b] Condition: Oligonucleotide (100 nmol), alkylpinacolyl boronate esters (400.0 equiv.), MesAcr (50 mol%), $(NH_4)_2S_2O_8$ (200.0 equiv.), TFA (50.0 equiv.), catechol (100.0 equiv.), and methylboronic acid (400.0 equiv.) in MeCN (0.25 mL), H_2O (0.25 mL), irradiated by 85 W white light at r.t. for 16 h. [c] Condition: Oligonucleotide (100 nmol), alkylboronic acid (200.0 equiv.), MesAcr (50 mol%), $(NH_4)_2S_2O_8$ (200.0 equiv.), TFA (50.0 equiv.), and catechol (50.0 equiv.) in MeCN (0.25 mL), H_2O (0.25 mL), irradiated by 10 W white LED at 10°C for 16 h. [d] Condition: Oligonucleotide (100 nmol), alkylboronic acid (400.0 equiv.), MesAcr (50 mol%), $(NH_4)_2S_2O_8$ (200.0 equiv.), TFA (50.0 equiv.), and catechol (100.0 equiv.) in MeCN (0.25 mL), H_2O (0.25 mL), irradiated by 10 W white LED at 4°C for 16 h. [e] Yields were determined by LC-MS and used the analytical method B. [f] Yields were determined by LC-MS and used the analytical method C.

RP-HPLC profiles of RNA oligonucleotides with diverse types of free radical precursors

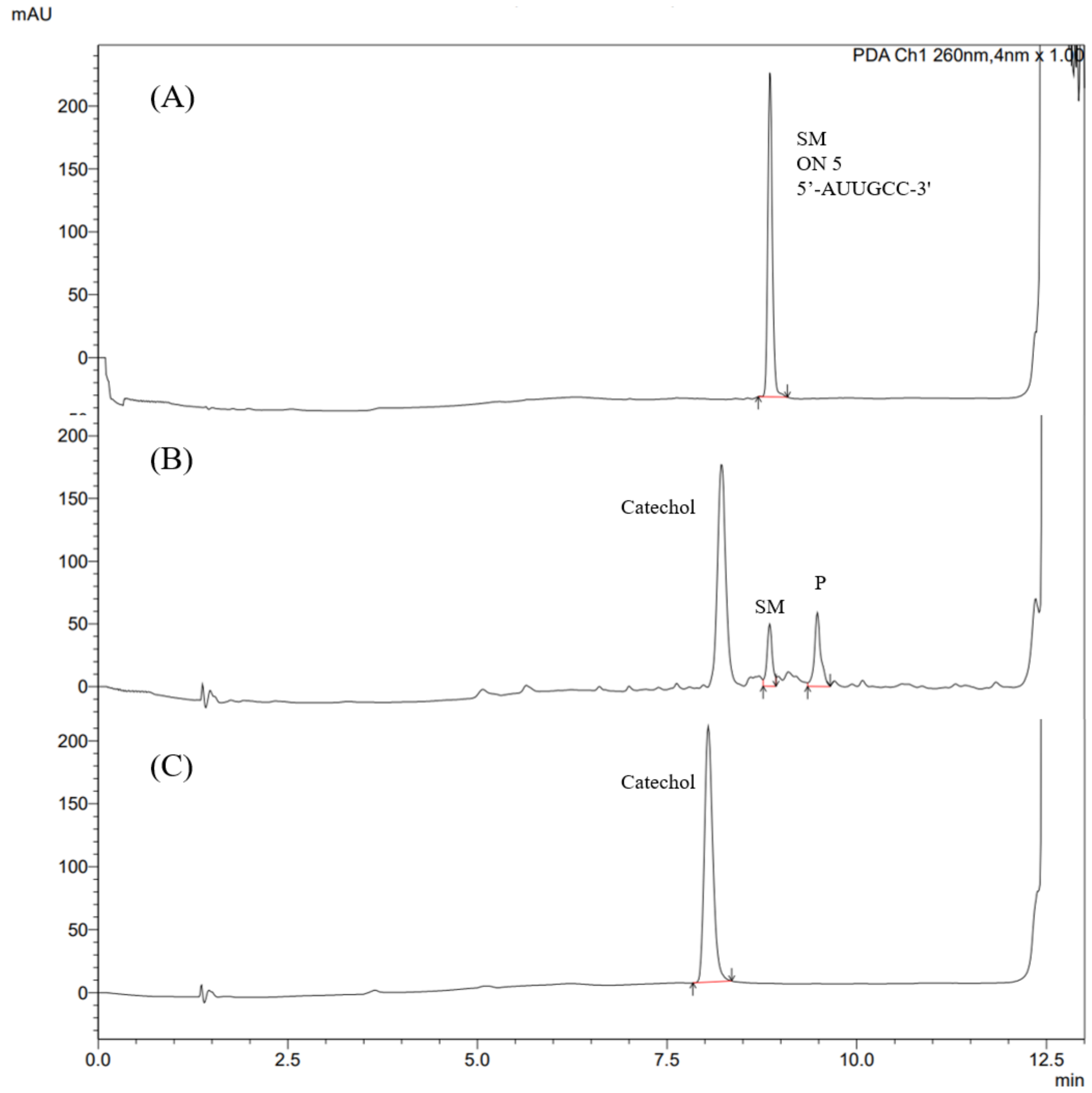

Supplementary Figure 49. Reverse-phase HPLC traces of (A) **ON 5**_a prepared by solid-phase RNA synthesis, (B) reaction mixture following catechol-promoted photoredox C–H alkylation of **ON 5** with *N*-propyl boronic acids, and (C) the catechol.

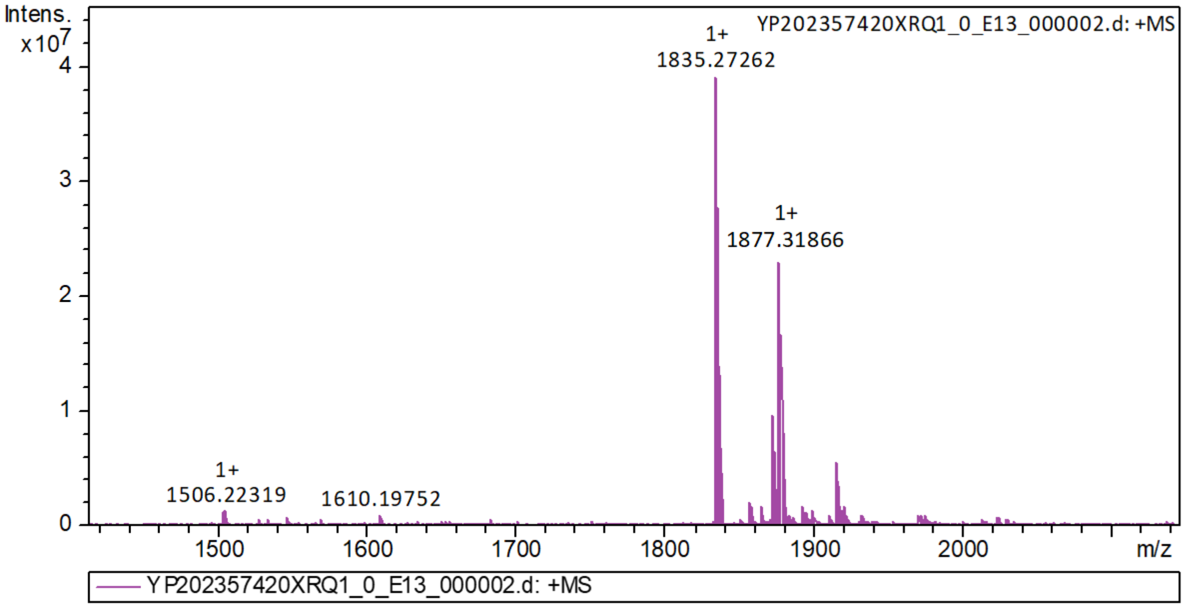

Supplementary Figure 50. Identification of G modification by MALDI-FTMS analysis of the reaction system of **ON 5**. Mass spectrum (MS) of desire product after *N*-propylization of **ON 5**.

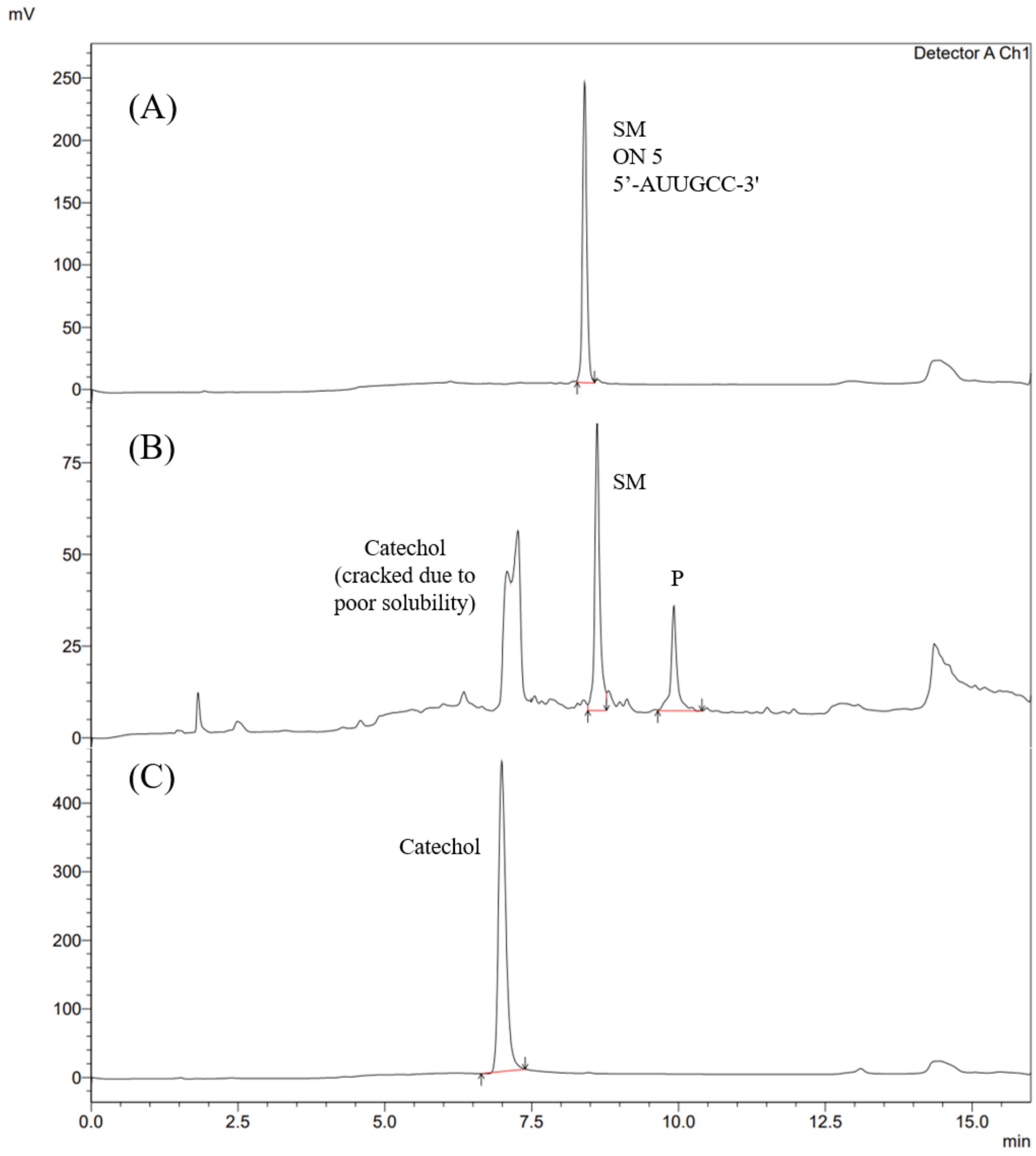

Supplementary Figure 51. Reverse-phase HPLC traces of (A) **ON 5**_a prepared by solid-phase RNA synthesis, (B) reaction mixture following catechol-promoted photoredox C–H alkylation of **ON 5** with compound **2n”** (P: compound **18**), and (C) the catechol.

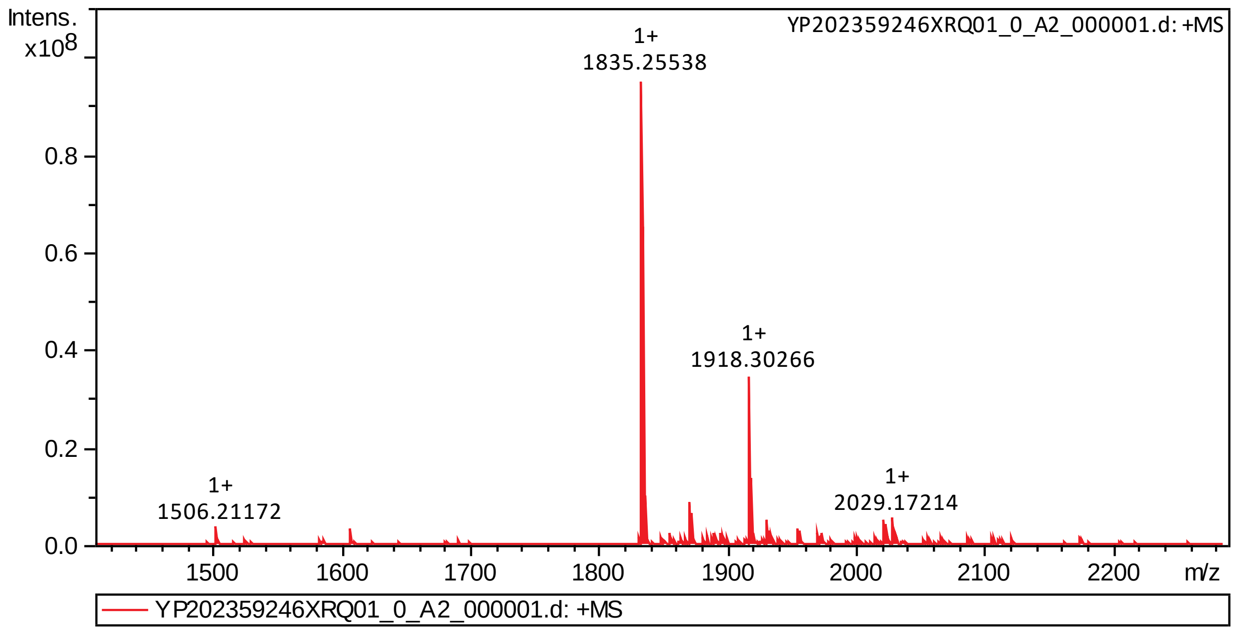

Supplementary Figure 52. Identification of G modification by MALDI-FTMS analysis of the reaction system of **ON 5**. Mass spectrum (MS) of desire product after alkylation of **ON 5**.

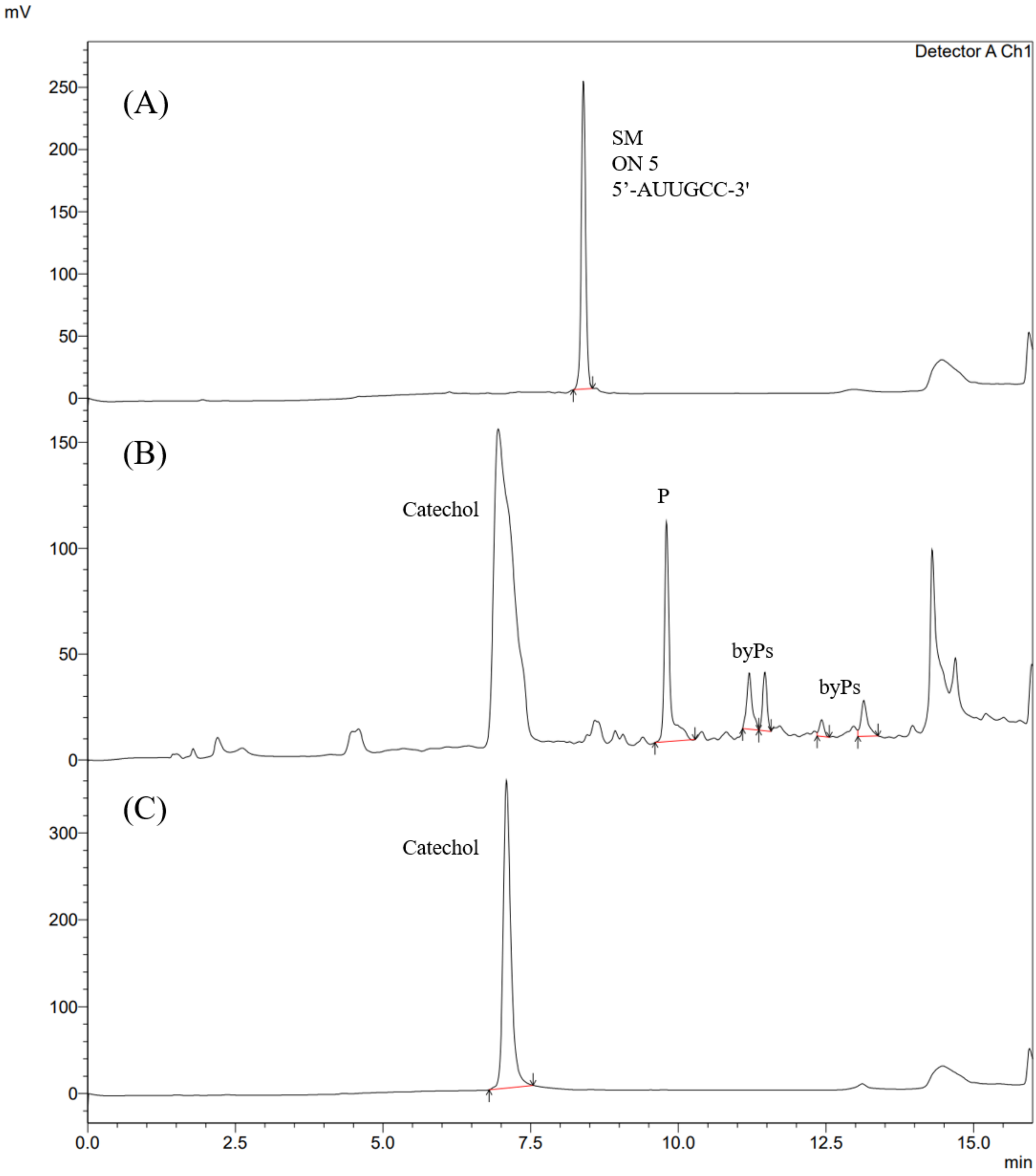

Supplementary Figure 53. Reverse-phase HPLC traces of (A) **ON 5**_a prepared by solid-phase RNA synthesis, (B) reaction mixture following catechol-promoted photoredox C–H alkylation of **ON 5** with cyclobutyl boronic acids, and (C) the catechol.

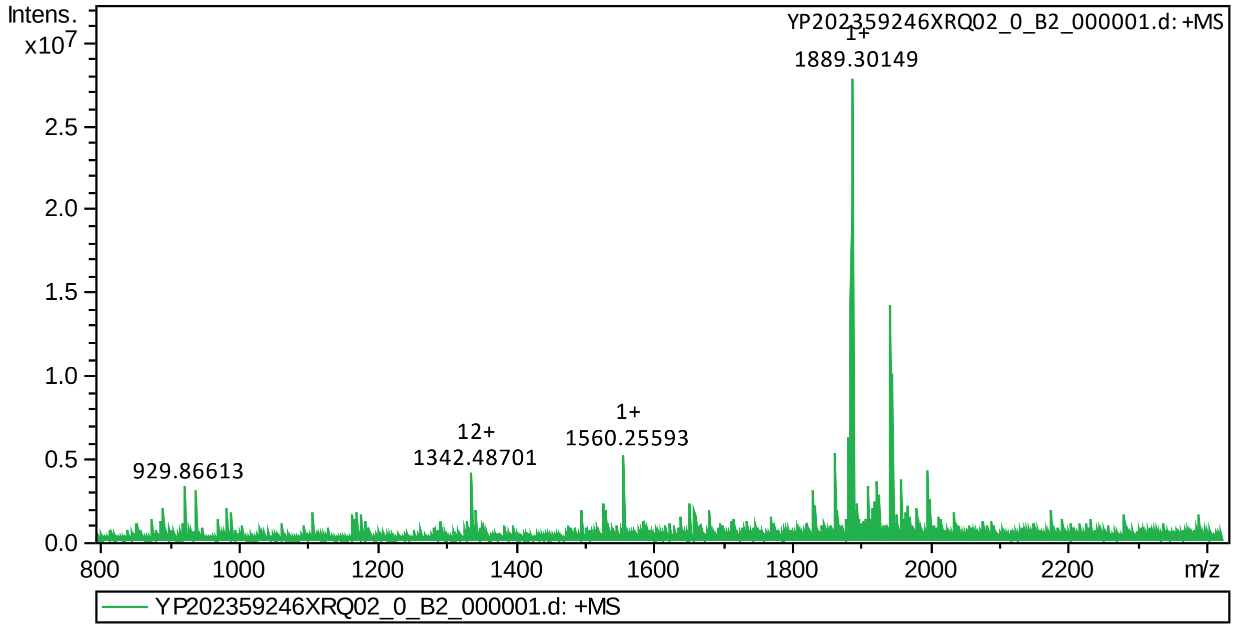

Supplementary Figure 54. Identification of G modification by MALDI-FTMS analysis of the reaction system of **ON 5**. Mass spectrum (MS) of desire product after cyclobutylation of **ON 5**.

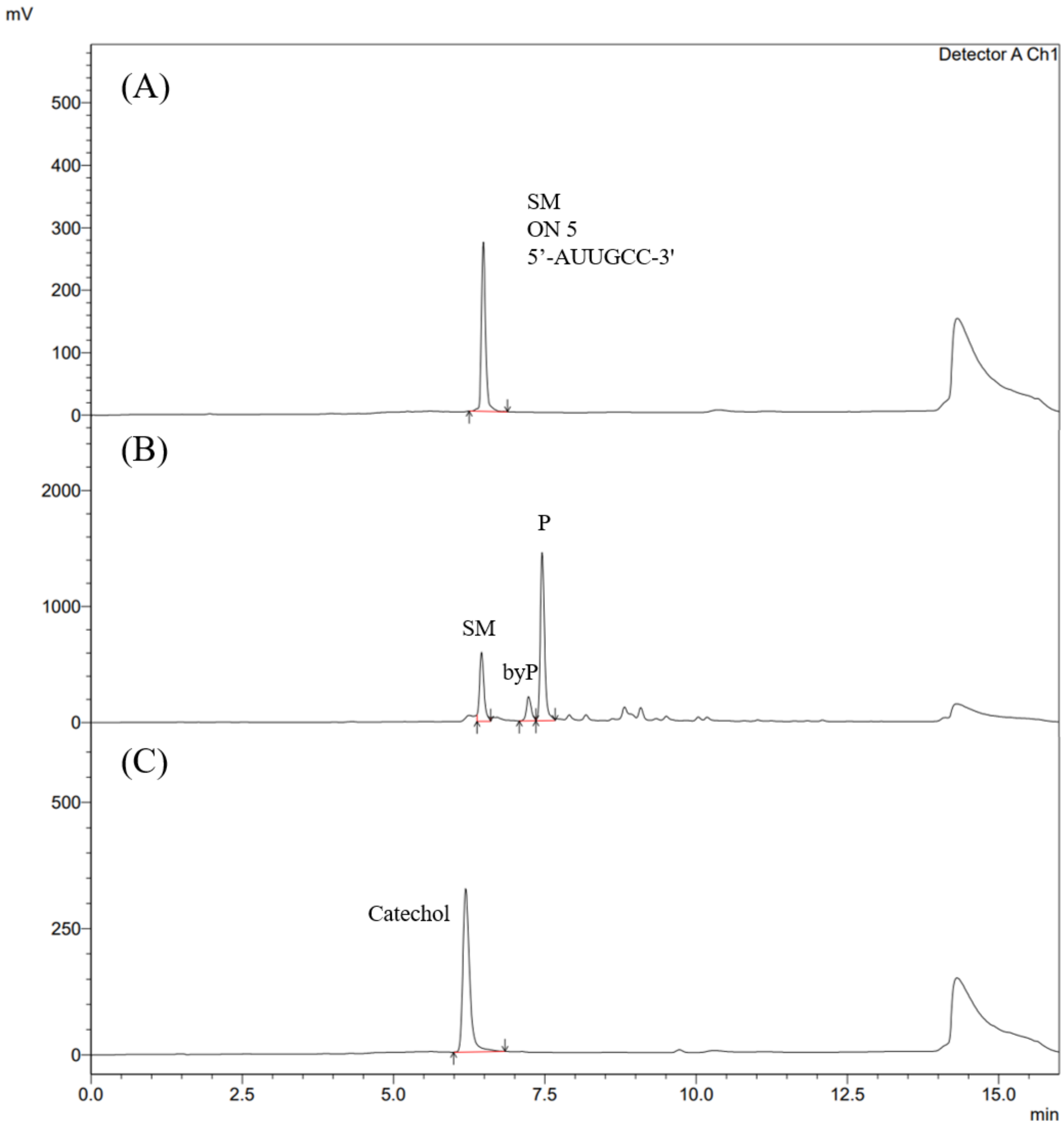

Supplementary Figure 55. Reverse-phase HPLC traces of (A) **ON 5**_a prepared by solid-phase RNA synthesis, (B) reaction mixture following catechol-promoted photoredox C–H alkylation of **ON 5** with cyclopentyl boronic acids, and (C) the catechol.

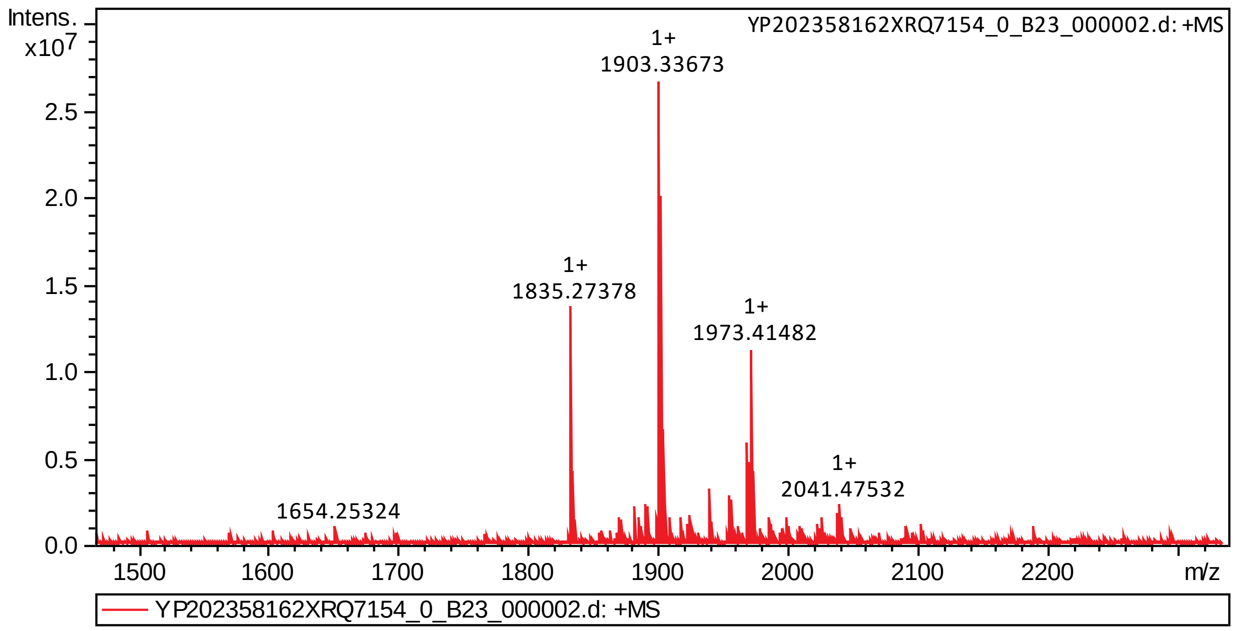

Supplementary Figure 56. Identification of G modification by MALDI-FTMS analysis of the reaction system of **ON 5**. Mass spectrum (MS) of desire product after cyclopentanylation of **ON 5**.

8.2. Substrate scope of C–H ethylation of ssDNA oligonucleotides

Supplementary Table 20. Substrate scope of C–H ethylation of the ssDNA oligonucleotides [a]

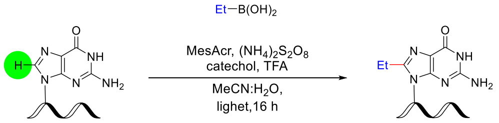

Entry	Oligonucleotides & Sequence	Yield [%] [c]	Calcd. Exact mass	Exptl. m/z (MALDI-FTMS)
**1** [b]	**dGpT** 5'-dGT-3'	70 [d]	599.1741	$[M+H]^+$ = 600.1807 [e]
**2** [b]	**dTpG** 5'-dTG-3'	65 [d]	599.1741	$[M+2Na]^+$ = 644.1451 [e]
**3**	**ON 9** 5'-dGTTCC-3'	43	1481.3129	$[M+H]^+$ = 1482.32446
**4**	**ON 10** 5'-dTTCCG-3'	33	1481.3129	$[M+H]^+$ = 1482.32241
**5**	**ON 11** 5'-dCGTT-3'	63	1192.2665	$[M+H]^+$ = 1193.27804
**6**	**ON 12** 5'-dTTGCC-3'	80	1481.3129	$[M+H]^+$ = 1482.32368
**7**	**ON 13** 5'-dCATGT-3'	64	1505.3241	$[M+H]^+$ = 1506.34015
**8**	**ON 14** 5'-dCCCGTTT-3'	62	2074.4053	$[M+H]^+$ = 2075.40114
**9**	**ON 15** 5'-dCACGTTT-3'	69	2098.4165	$[M+H]^+$ = 2099.43403
**10**	**ON 16** 5'-dCCTTGTTCC-3'	51	2667.4977	$[M+H]^+$ = 2668.47158
**11**	**ON 17** 5'-dCACTTGTTC-3'	59	2691.5089	$[M+H]^+$ = 2692.49906

[a] Condition: Oligonucleotide (100 nmol), ethylboronic acid (400.0 equiv.), MesAcr (50 mol%), $(NH_4)_2S_2O_8$ (200.0 equiv.), TFA (50.0 equiv.), and catechol (100.0 equiv.) in MeCN (0.25 mL), H_2O (0.25 mL), irradiated by 85 W white light at r.t. for 16 h. [b] The reaction was irradiated by 10 W blue LED and carried out at 0 °C for 5 h. [c] Yields were determined by LC-MS and used the analytical method B. [d] Yields were determined by LC-MS and used the analytical method A. [e] The mass data was collected by using HRMS.

RP-HPLC profiles of ssDNA oligonucleotides

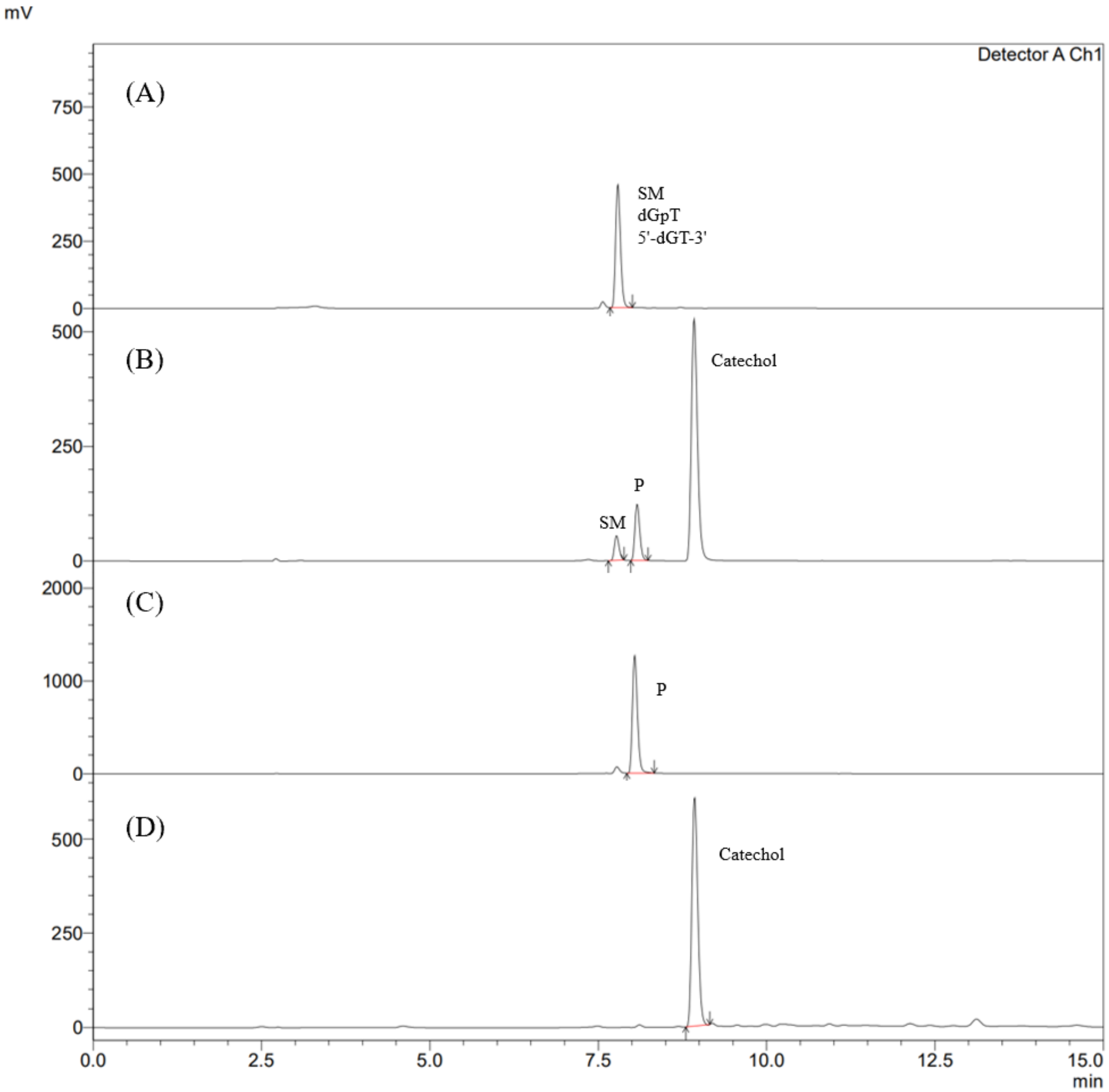

Supplementary Figure 57. Reverse-phase HPLC traces of (A) the starting material (dGpT), (B) the reaction mixture following catechol-promoted photoredox C–H alkylation of dGpT with ethyl boronic acid, (C) the corresponding ethylated product was used as a standard sample, and (D) the catechol.

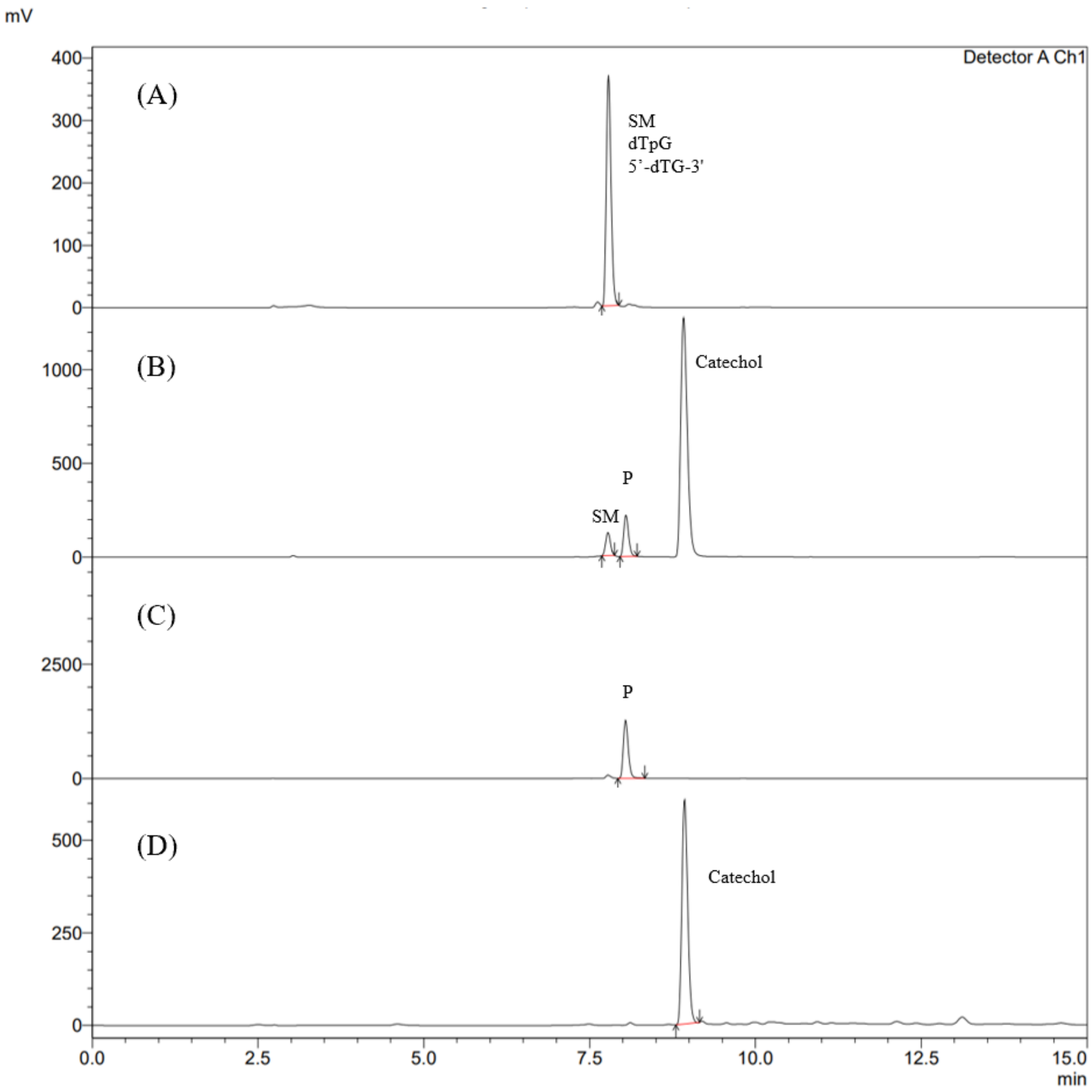

Supplementary Figure 58. Reverse-phase HPLC traces of (A) the starting material (dTpG), (B) the reaction mixture following catechol-promoted photoredox C–H alkylation of dTpG with ethyl boronic acid, (C) the corresponding ethylated product was used as a standard sample, and (D) the catechol.

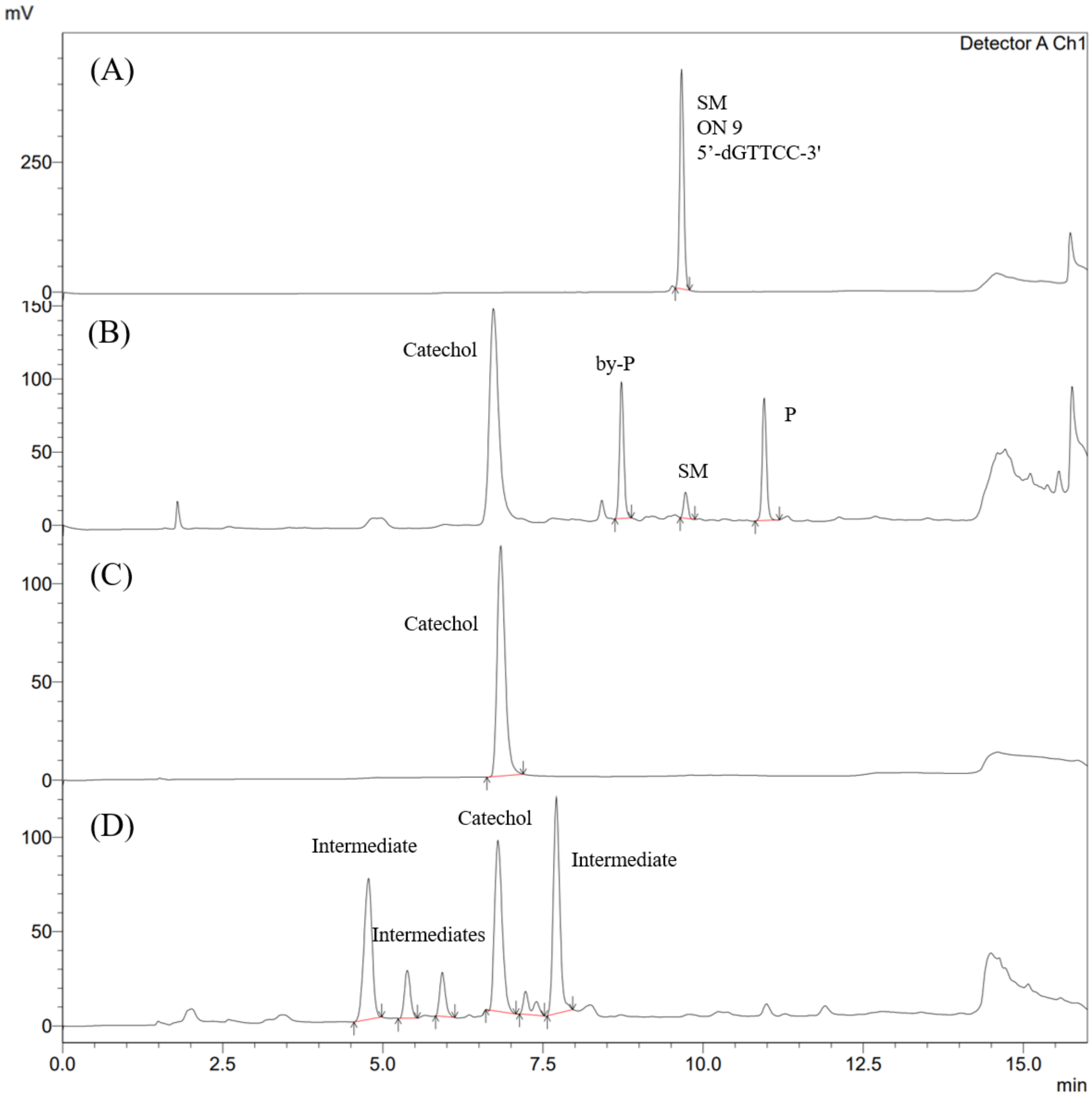

Supplementary Figure 59. Reverse-phase HPLC traces of (A) **ON 9**_a prepared by solid-phase DNA synthesis, (B) reaction mixture following catechol-promoted photoredox C–H alkylation of **ON 9** with ethyl boronic acids, (C) the catechol, and (D) the catechol and the intermediate that prepared by mixing the catechol and ethyl boronic acid.

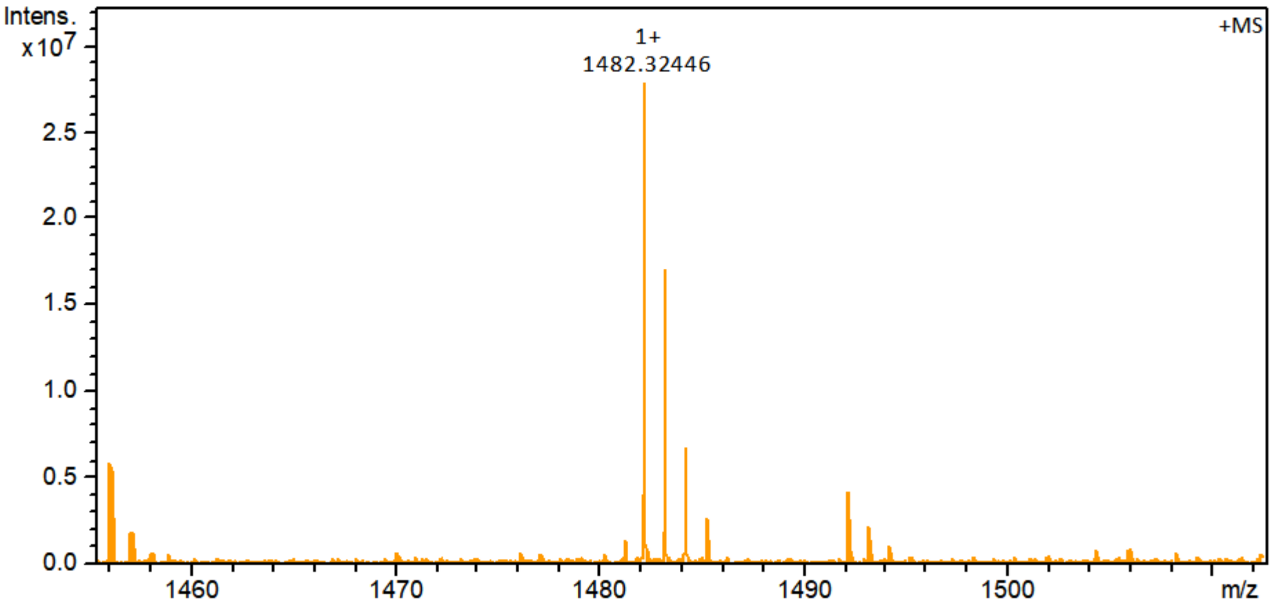

Supplementary Figure 60. Identification of G modification by MALDI-FTMS analysis of the reaction system of **ON 9**. Mass spectrum (MS) of desired product after ethylation of **ON 9**.

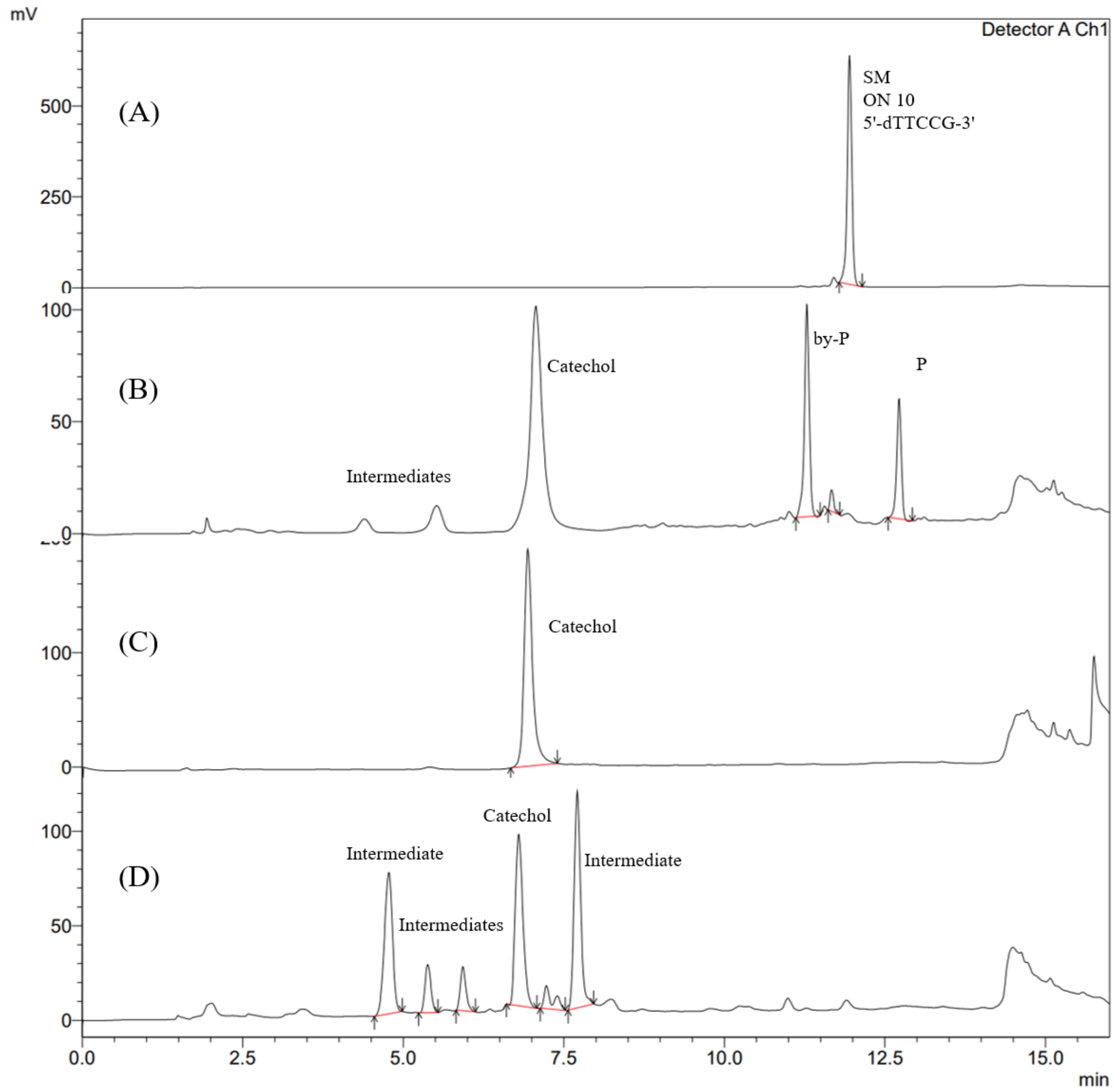

Supplementary Figure 61. Reverse-phase HPLC traces of (A) **ON 10**_a prepared by solid-phase DNA synthesis, (B) reaction mixture following catechol-promoted photoredox C–H alkylation of **ON 10** with ethyl boronic acids, (C) the catechol, and (D) the catechol and the intermediate that prepared by mixing the catechol and ethyl boronic acid.

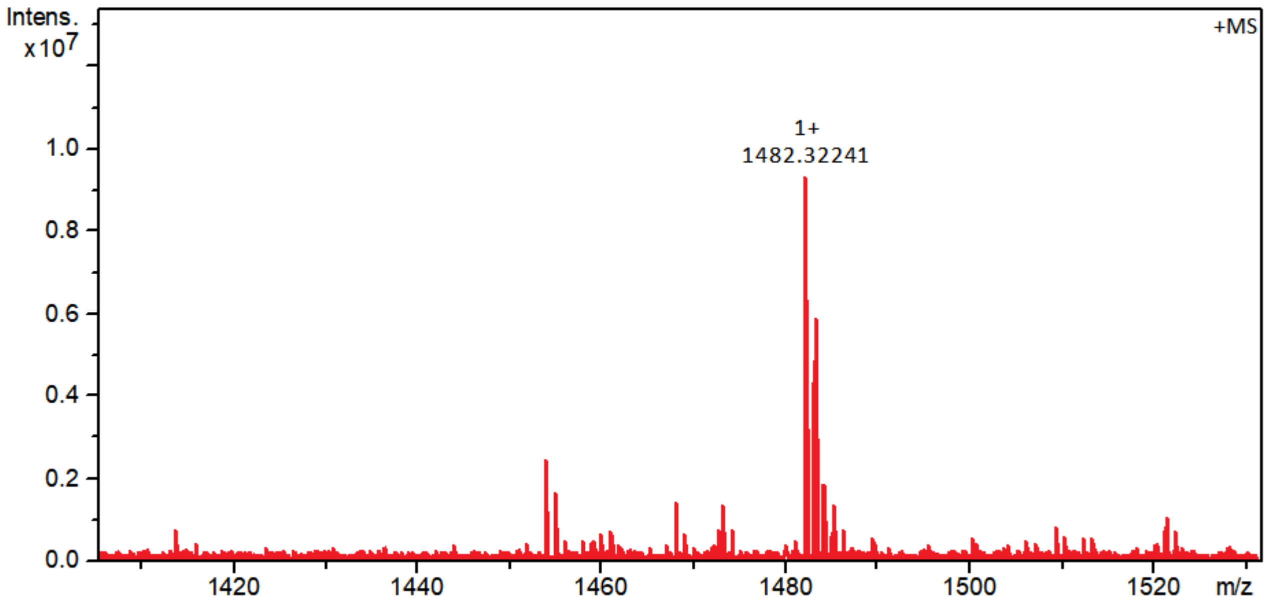

Supplementary Figure 62. Identification of G modification by MALDI-FTMS analysis of the reaction system of **ON 10**. Mass spectrum (MS) of desire product after ethylation of **ON 10**.

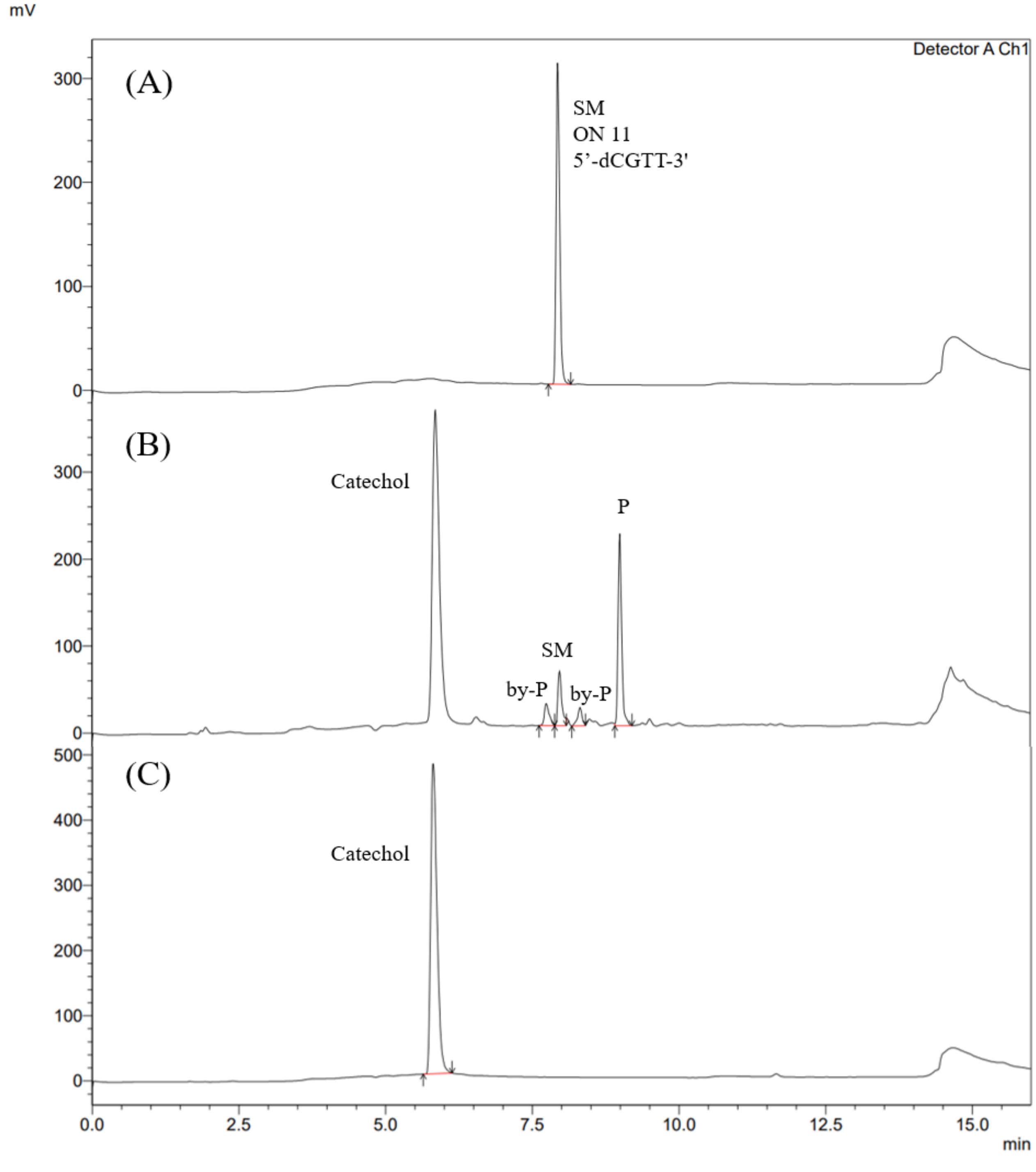

Supplementary Figure 63. Reverse-phase HPLC traces of (A) **ON 11**_a prepared by solid-phase DNA synthesis, (B) reaction mixture following catechol-promoted photoredox C–H alkylation of **ON 11** with ethyl boronic acids, and (C) the catechol.

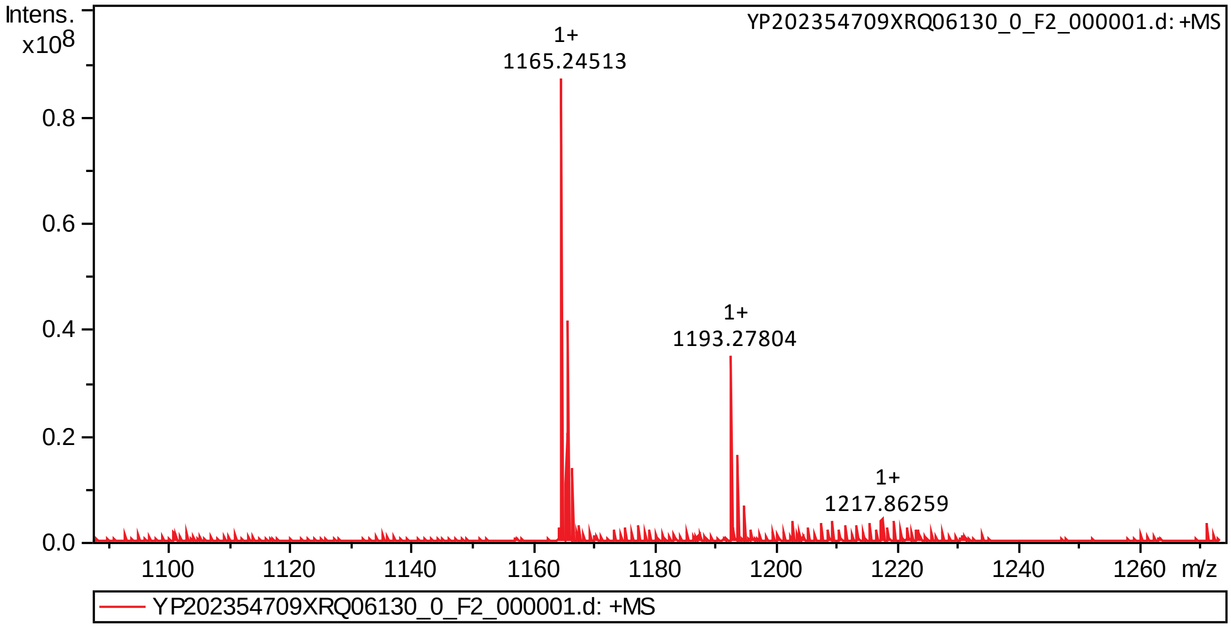

Supplementary Figure 64. Identification of G modification by MALDI-FTMS analysis of the reaction system of **ON 11**. Mass spectrum (MS) of desired product after ethylation of **ON 11**.

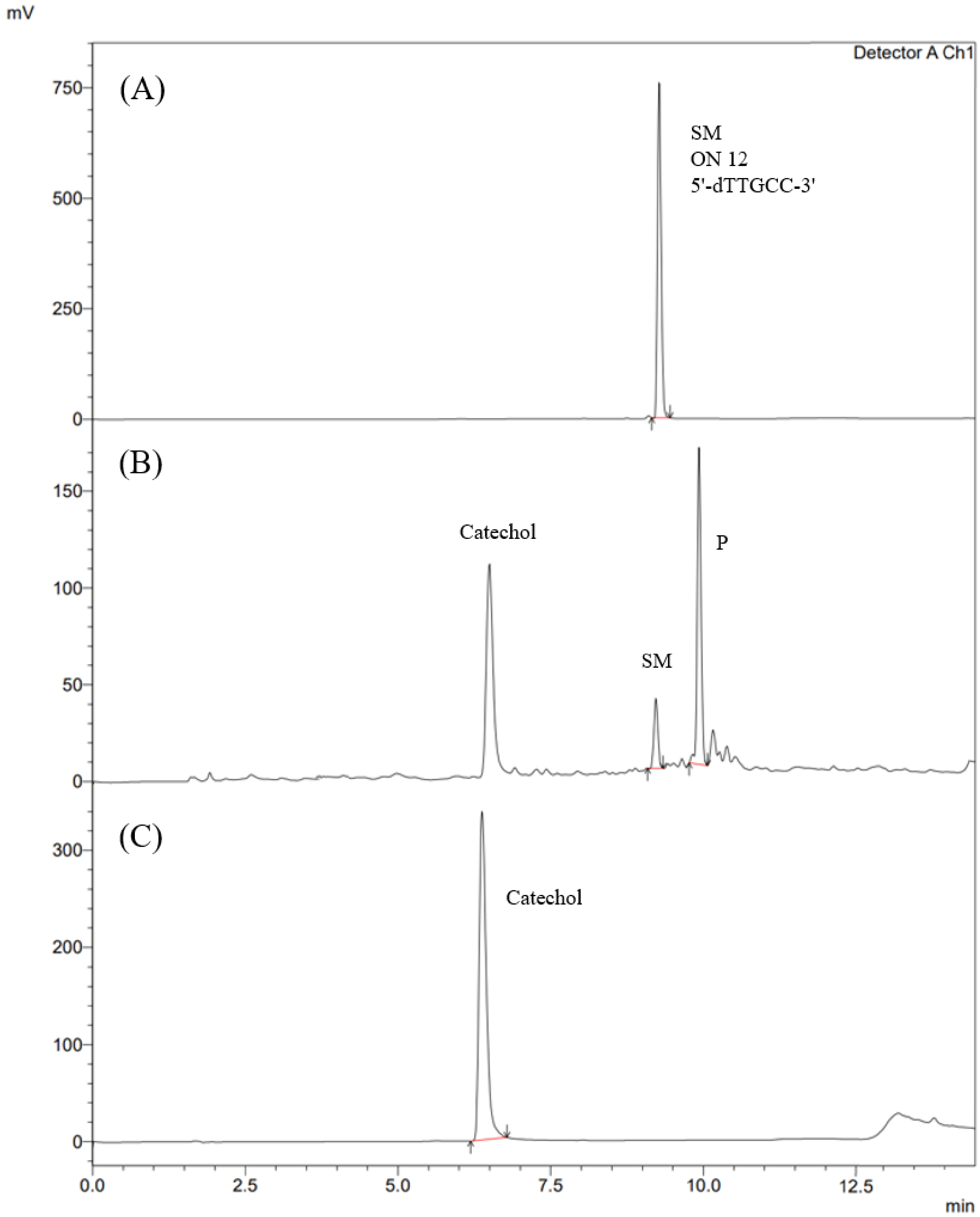

Supplementary Figure 65. Reverse-phase HPLC traces of (A) **ON 12**_a prepared by solid-phase DNA synthesis, (B) reaction mixture following catechol-promoted photoredox C–H alkylation of **ON 12** with ethyl boronic acids, (C) the catechol, and (D) the catechol and the intermediate that prepared by mixing the catechol and ethyl boronic acid.

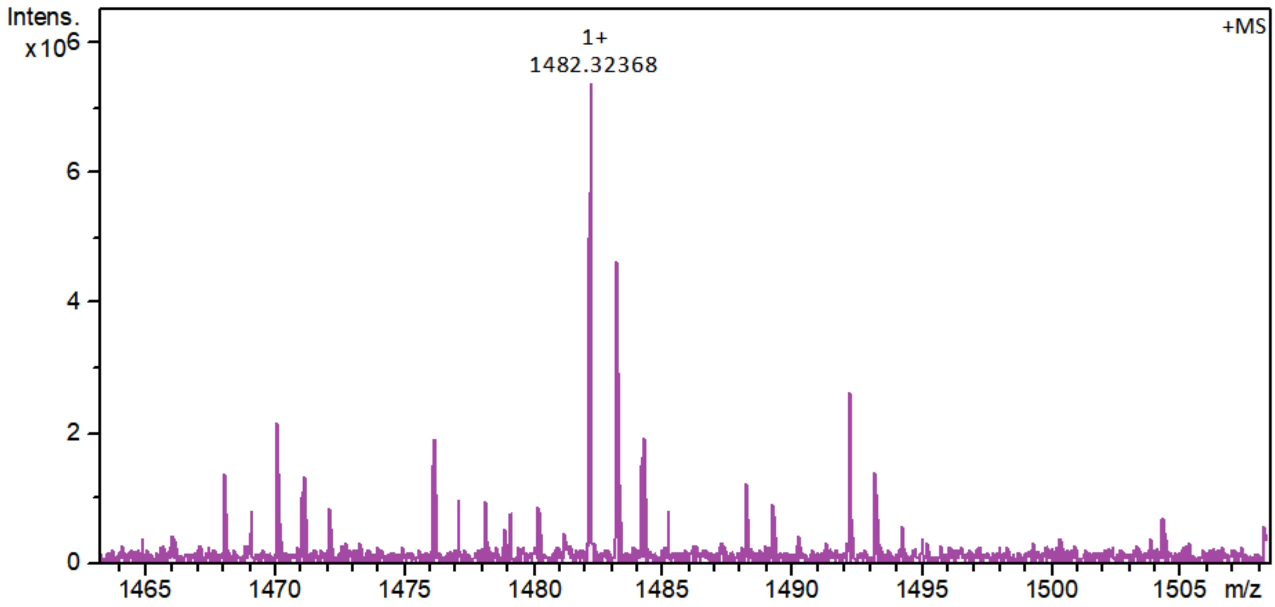

Supplementary Figure 66. Identification of G modification by MALDI-FTMS analysis of the reaction system of **ON 12**. Mass spectrum (MS) of desired product after ethylation of **ON 12**.

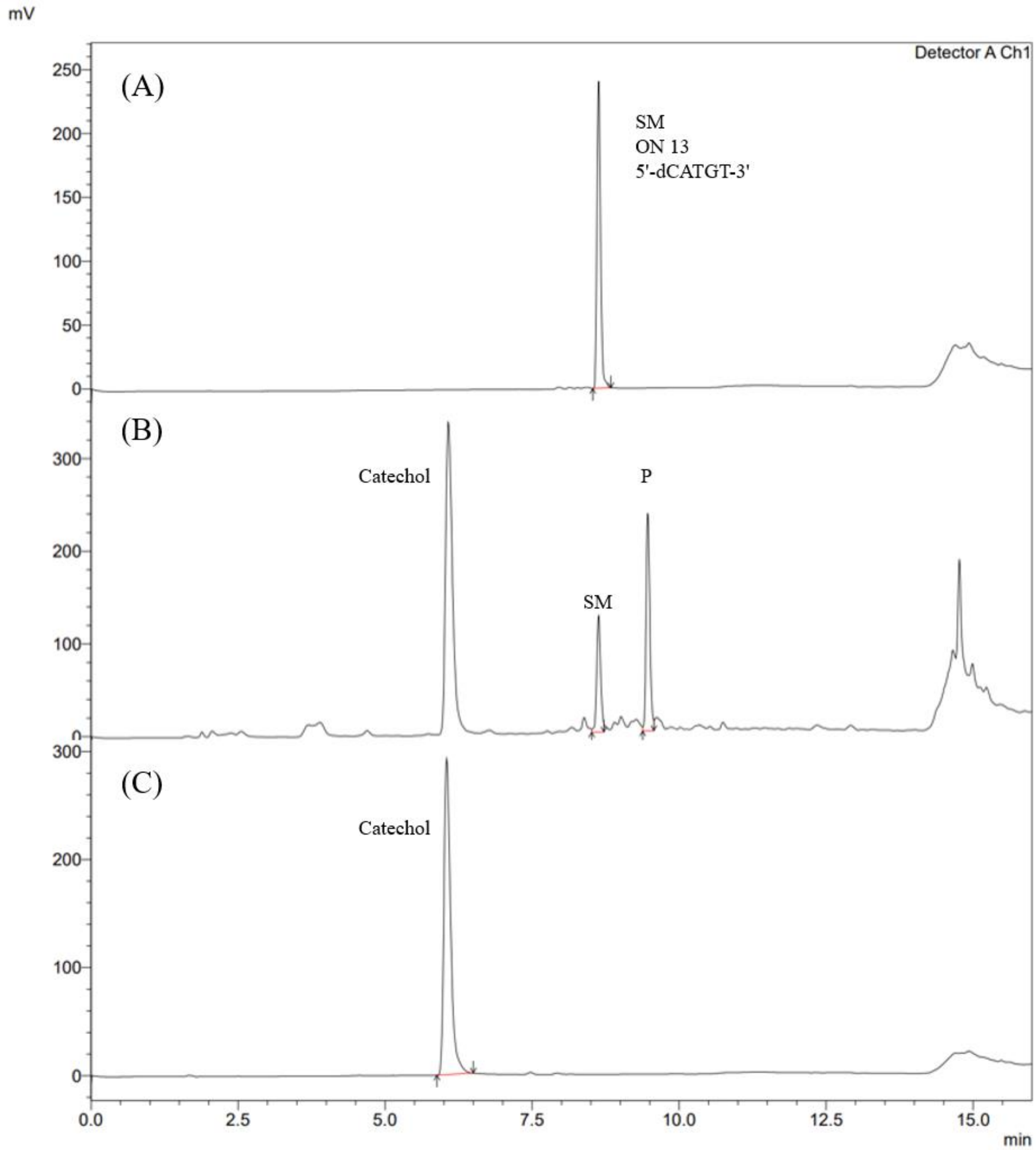

Supplementary Figure 67. Reverse-phase HPLC traces of (A) **ON 13**_a prepared by solid-phase DNA synthesis, (B) reaction mixture following catechol-promoted photoredox C–H alkylation of **ON 13** with ethyl boronic acids, (C) the catechol, and (D) the catechol and the intermediate that prepared by mixing the catechol and ethyl boronic acid.

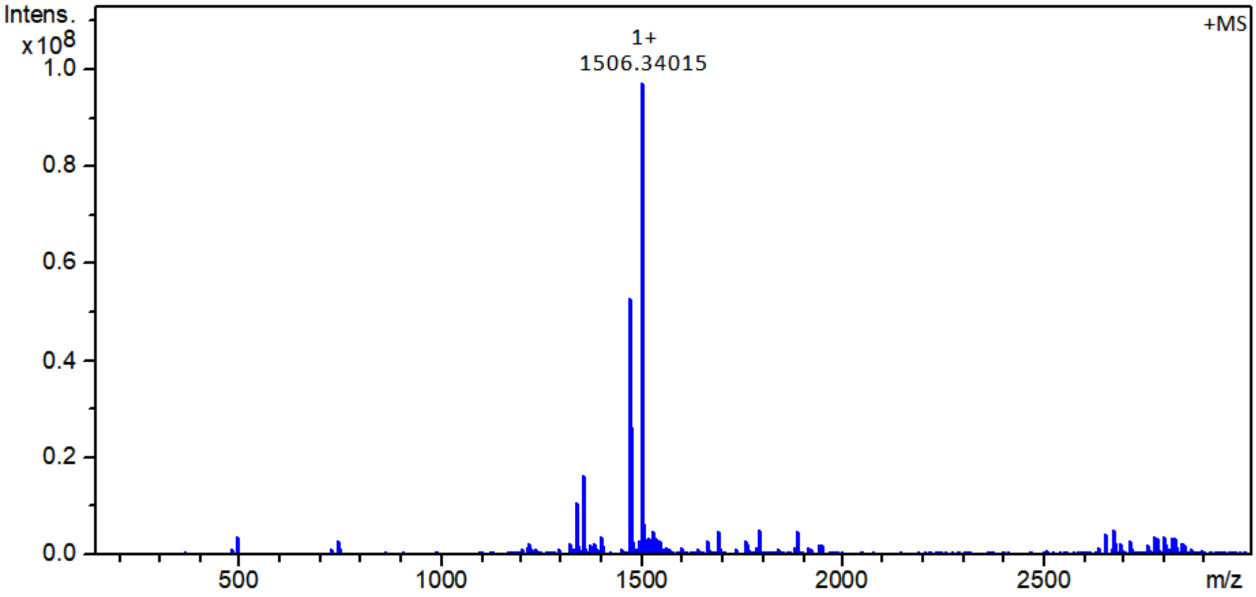

Supplementary Figure 68. Identification of G modification by MALDI-FTMS analysis of the reaction system of **ON 13**. Mass spectrum (MS) of desired product after ethylation of **ON 13**.

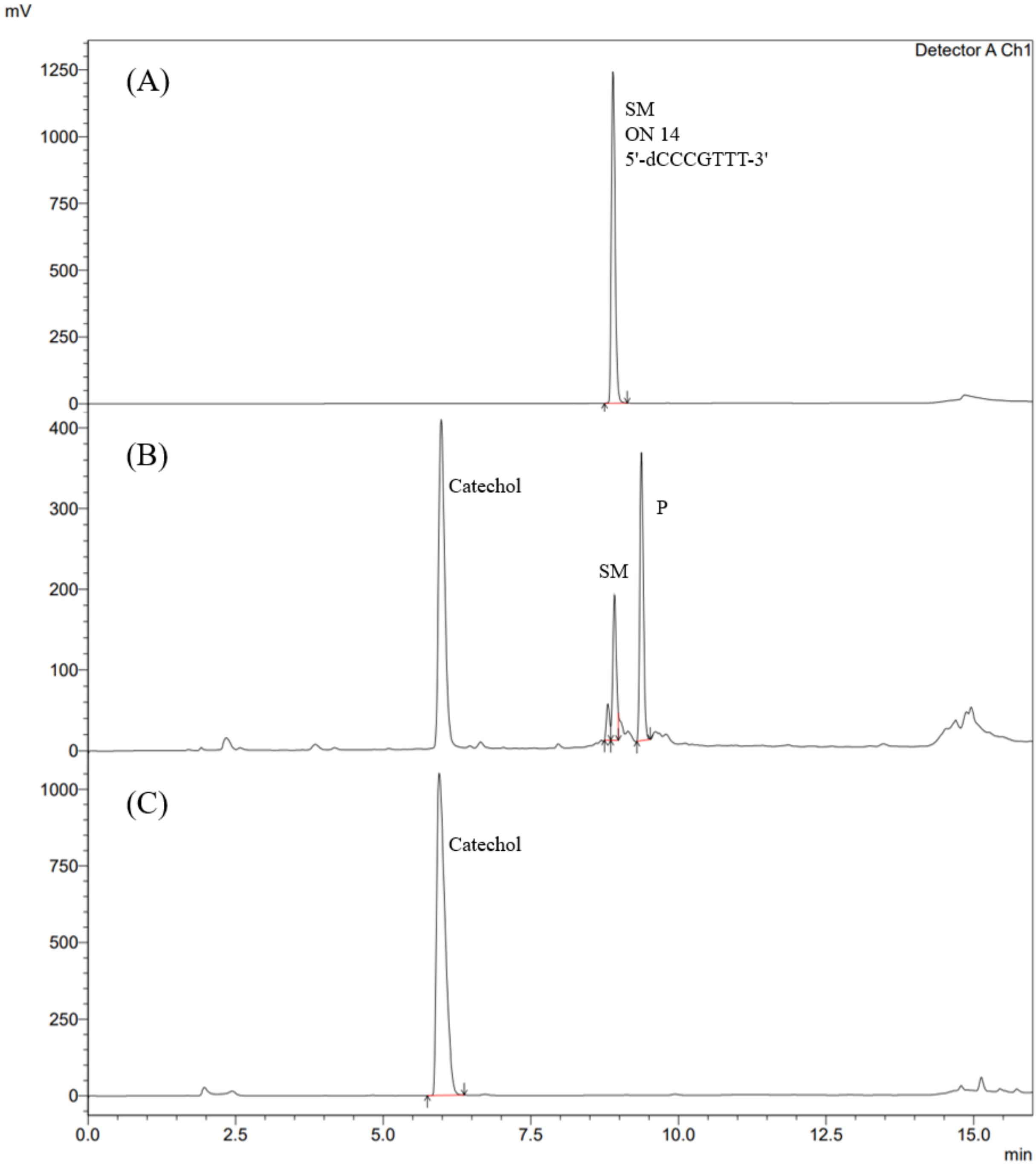

Supplementary Figure 69. Reverse-phase HPLC traces of (A) **ON 14**_a prepared by solid-phase DNA synthesis, (B) reaction mixture following catechol-promoted photoredox C–H alkylation of **ON 14** with ethyl boronic acids, and (C) the catechol.

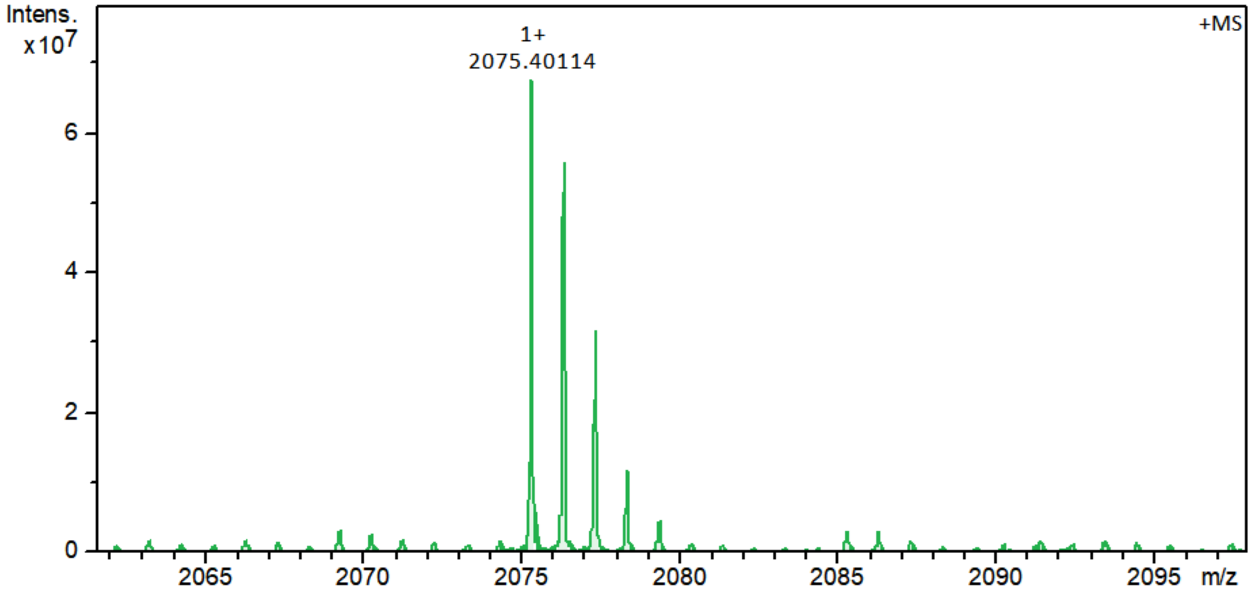

Supplementary Figure 70. Identification of G modification by MALDI-FTMS analysis of the reaction system of **ON 14**. Mass spectrum (MS) of desired product after ethylation of **ON 14**.

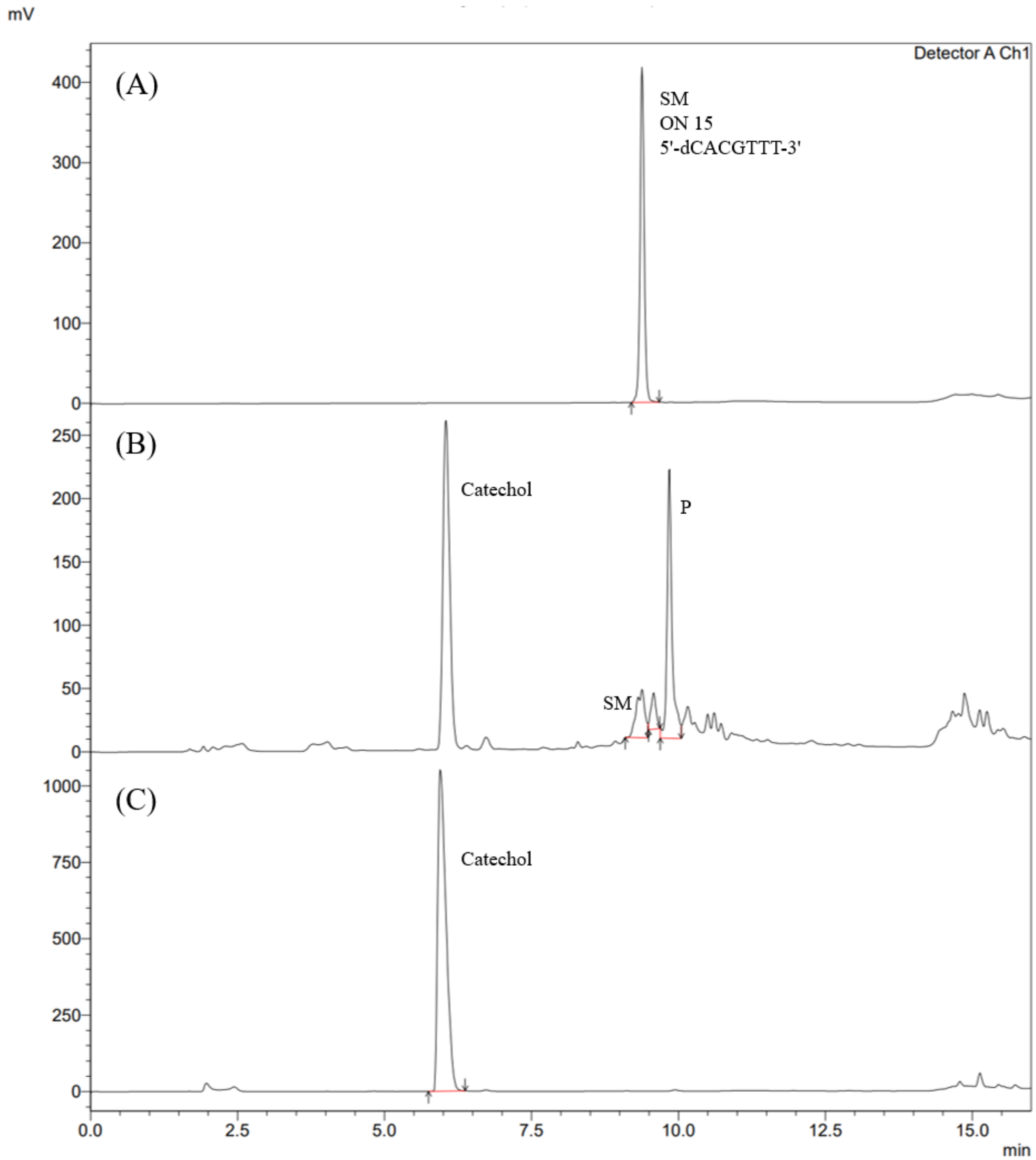

Supplementary Figure 71. Reverse-phase HPLC traces of (A) **ON 15**_a prepared by solid-phase DNA synthesis, (B) reaction mixture following catechol-promoted photoredox C–H alkylation of **ON 15** with ethyl boronic acids, and (C) the catechol.

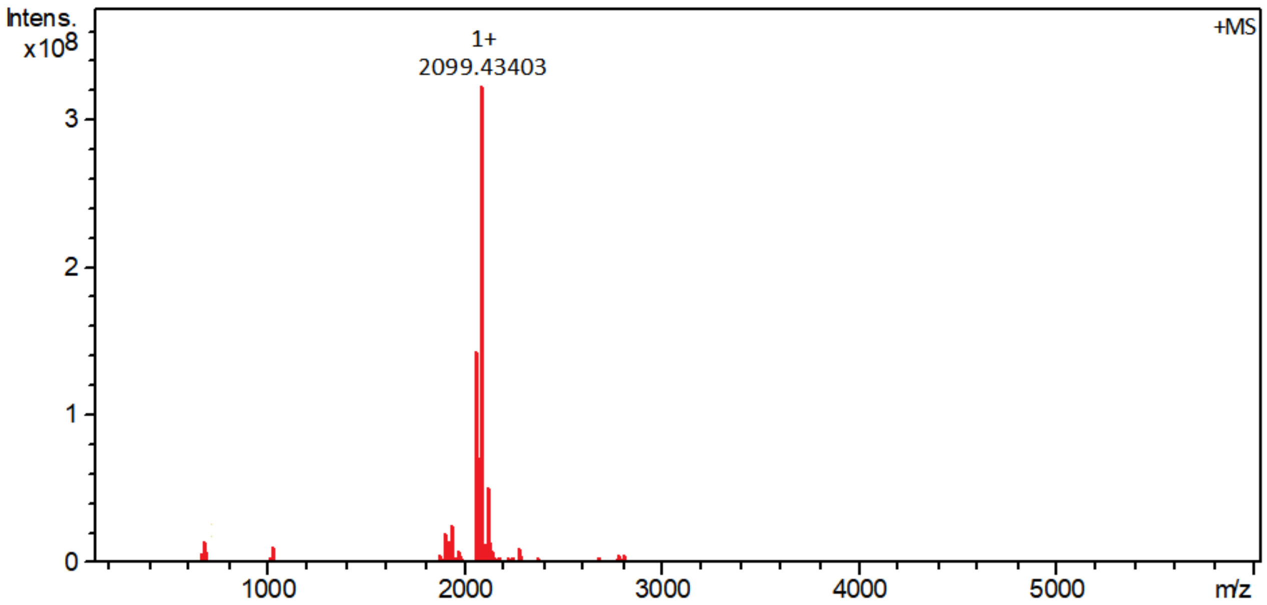

Supplementary Figure 72. Identification of G modification by MALDI-FTMS analysis of the reaction system of **ON 15**. Mass spectrum (MS) of desired product after ethylation of **ON 15**.

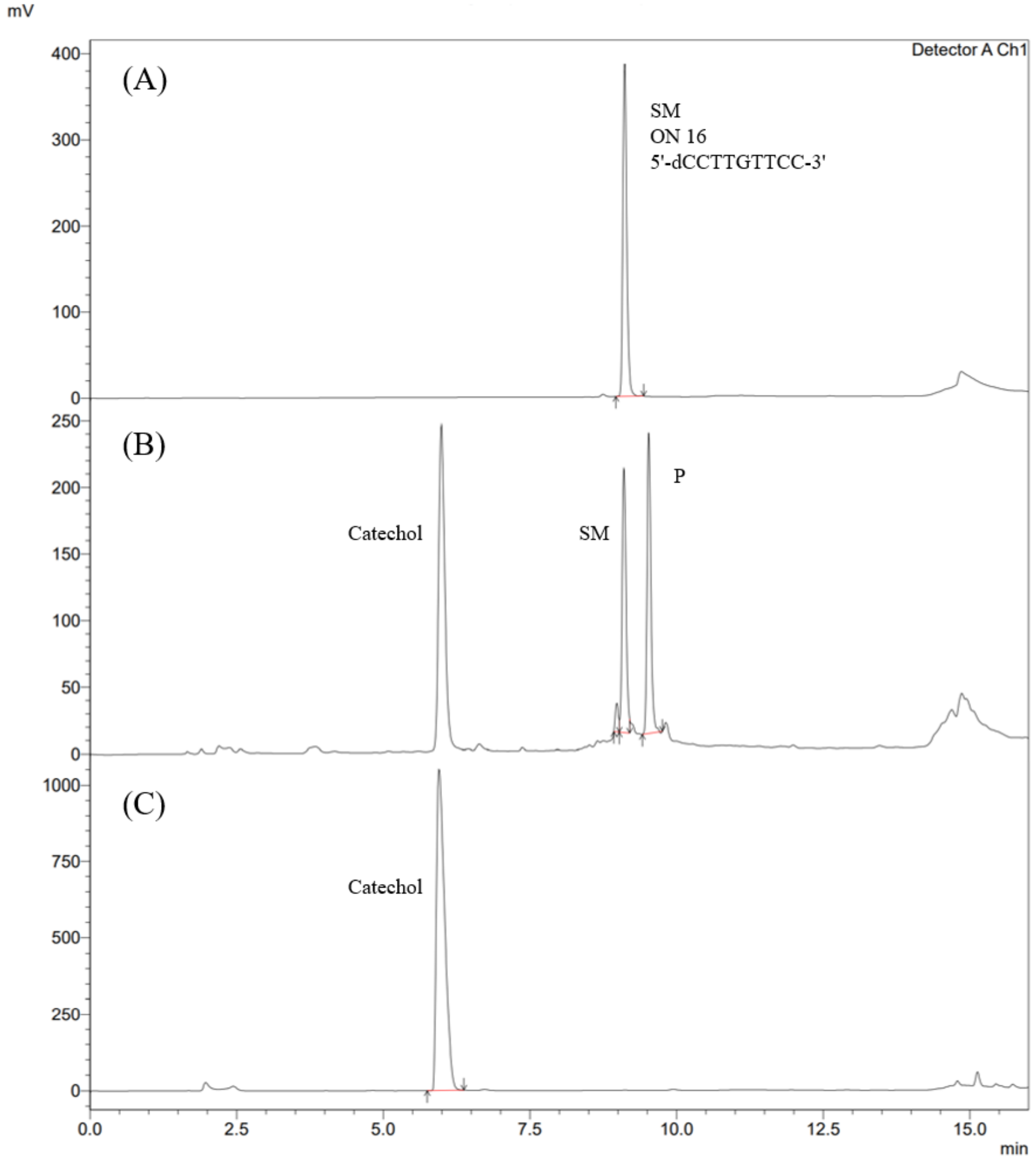

Supplementary Figure 73. Reverse-phase HPLC traces of (A) **ON 16**_a prepared by solid-phase DNA synthesis, (B) reaction mixture following catechol-promoted photoredox C–H alkylation of **ON 16** with ethyl boronic acids, and (C) the catechol.

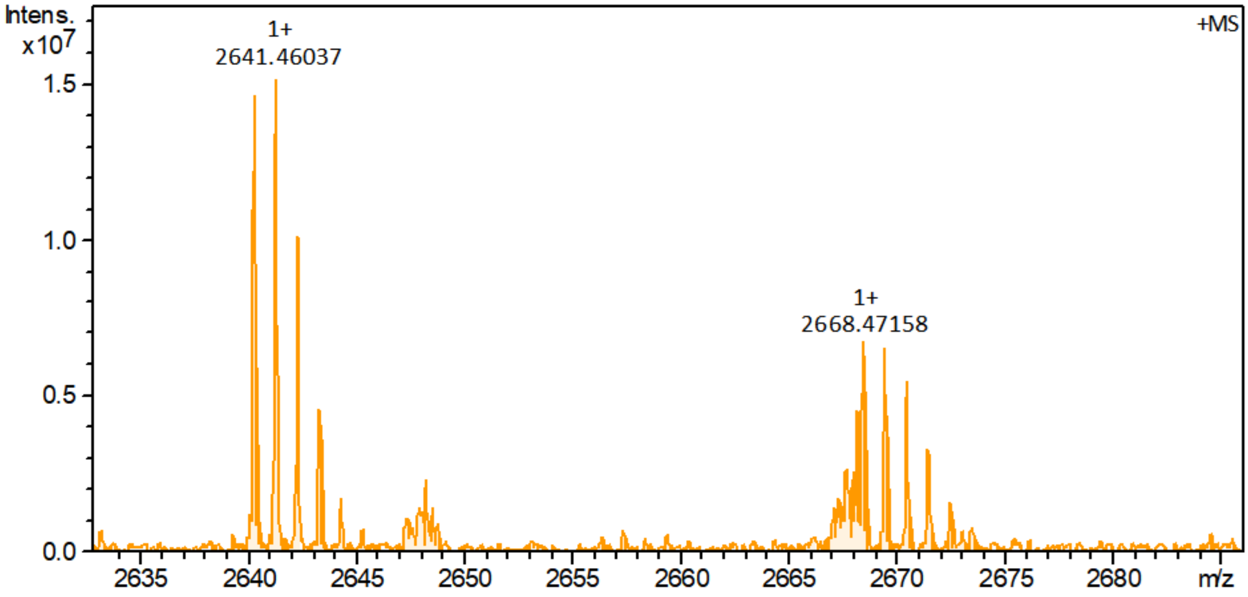

Supplementary Figure 74. Identification of G modification by MALDI-FTMS analysis of the reaction system of **ON 16**. Mass spectrum (MS) of desired product after ethylation of **ON 16**.

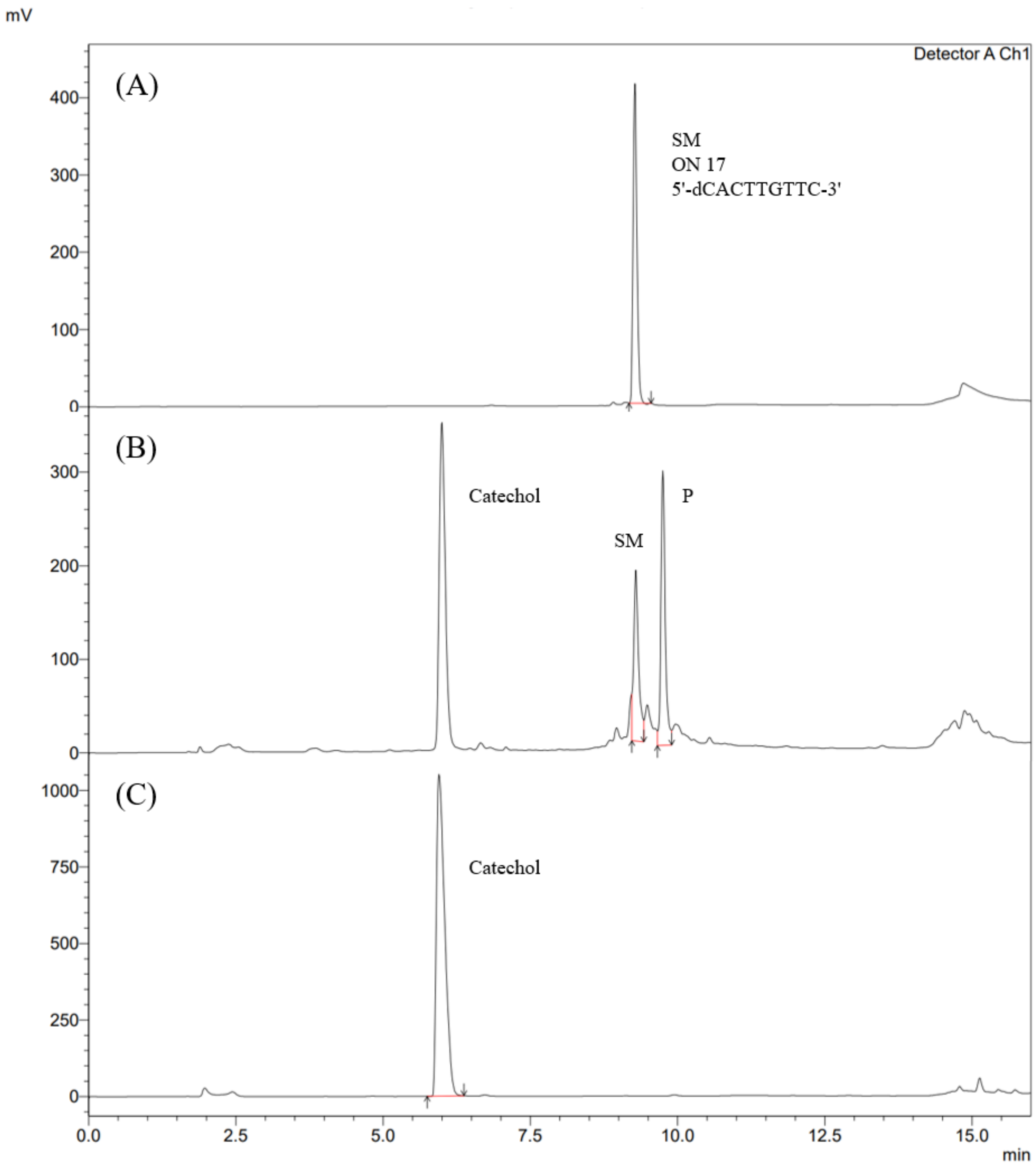

Supplementary Figure 75. Reverse-phase HPLC traces of (A) **ON 17**_a prepared by solid-phase DNA synthesis, (B) reaction mixture following catechol-promoted photoredox C–H alkylation of **ON 17** with ethyl boronic acids, and (C) the catechol.

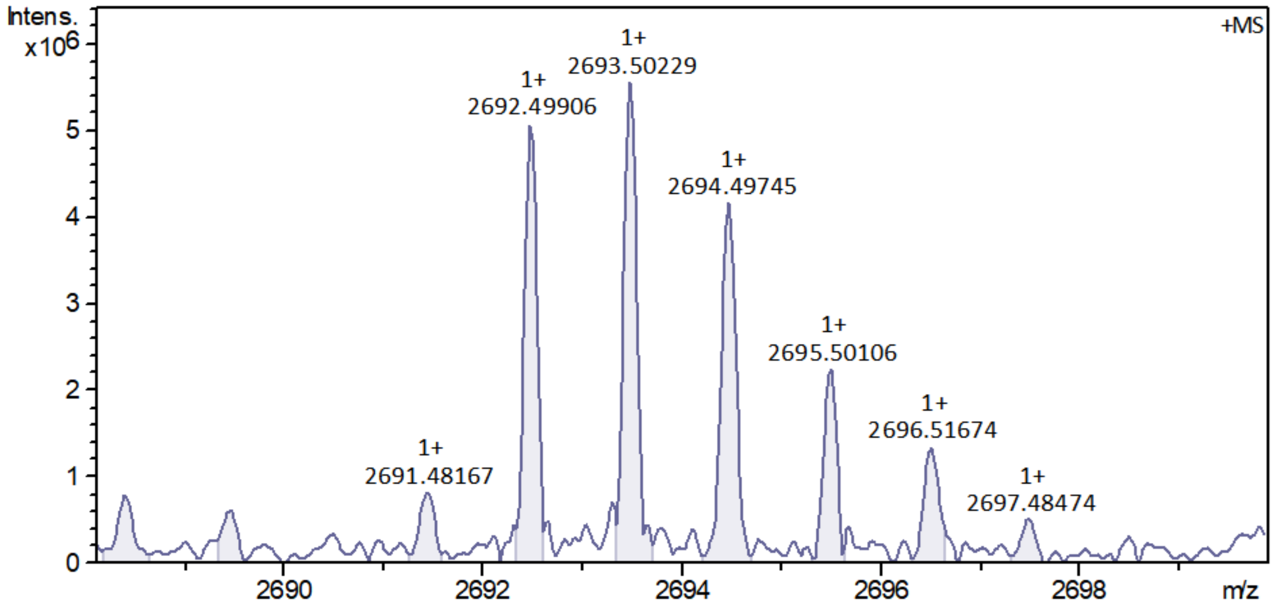

Supplementary Figure 76. Identification of G modification by MALDI-FTMS analysis of the reaction system of **ON 17**. Mass spectrum (MS) of desired product after ethylation of **ON 17**.

8.3. Substrate scope of C–H ethylation of oligonucleotides containing two guanines

Supplementary Table 21. Substrate scope of C–H ethylation of the oligonucleotides containing two guanines [a]

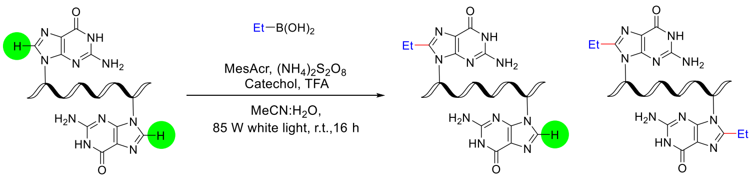

16a, 16b **17a, 17b**

Entry	Oligonucleotides & Sequence	Yield [%] [b]	Calcd. Exact mass	Exptl. m/z (MALDI-FTMS)
1	**ON 18** 5’-dTGTCGC-3’	33 [c]/67 [d]	1810.3654[c]/1838.3967 [d]	$[M+H]^+$ =1811.37688[c]/1839.41817 [d]
2	**ON 19** 5'-CGAUGU-3'	33 [c]/56 [d]	1902.3148[c]/1930.346 [d]	$[M+H]^+$ = 1903.31515[c]/1931.34806 [d]

[a] Condition: Oligonucleotide (100 nmol), ethylboronic acid (400.0 equiv.), MesAcr (50 mol%), $(NH_4)_2S_2O_8$ (200.0 equiv.), TFA (50.0 equiv.), and catechol (100.0 equiv.) in MeCN (0.25 mL), H_2O (0.25 mL), irradiated by 85 W white light at r.t. for 16 h. [b] Yields were determined by LC-MS and used the analytical method B. [c] Data of monosubstituted product(s). [d] Data of disubstituted product.

RP-HPLC profiles of oligonucleotides containing two guanines

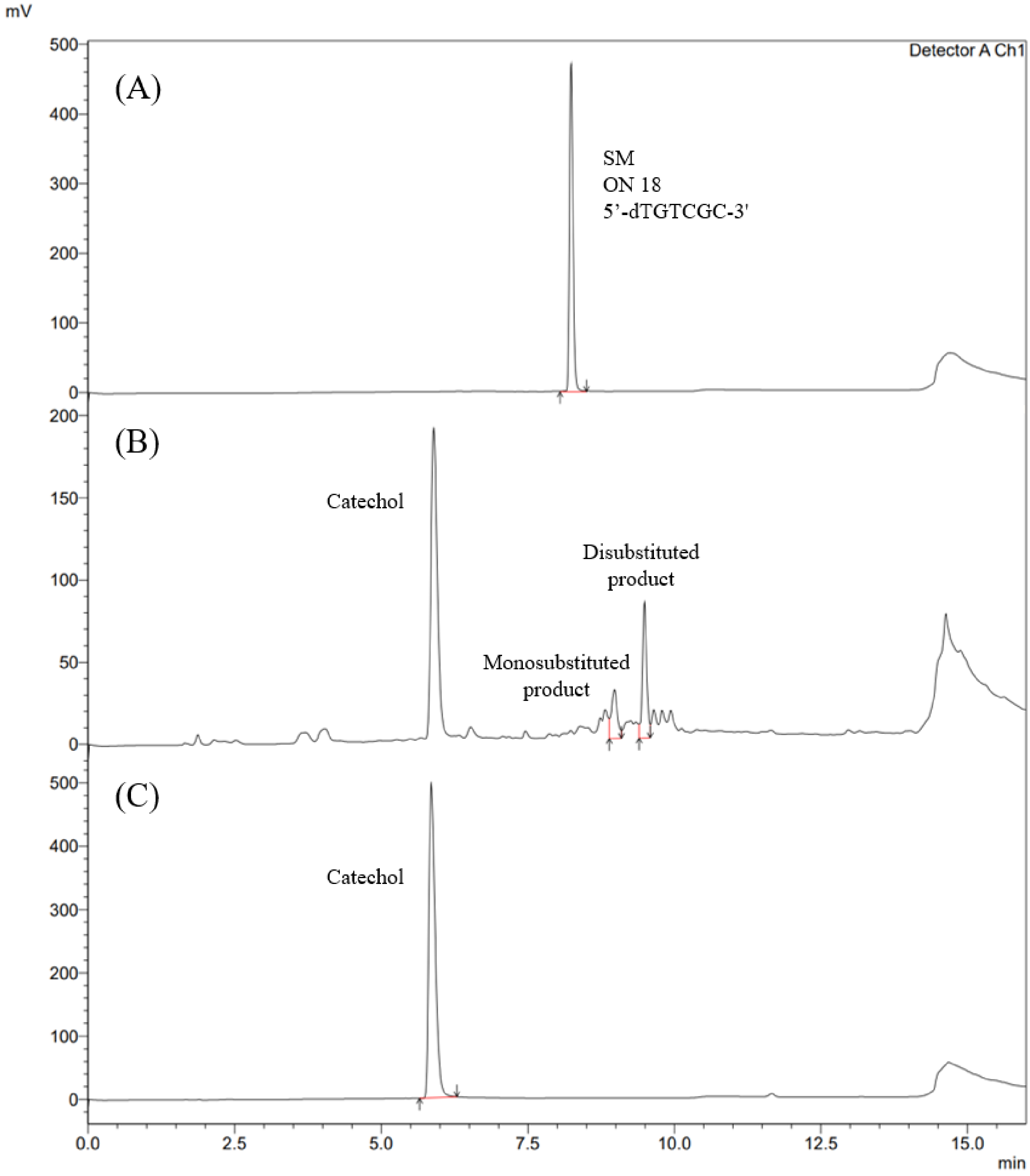

Supplementary Figure 77. Reverse-phase HPLC traces of (A) **ON 18**_a prepared by solid-phase DNA synthesis, (B) reaction mixture following catechol-promoted photoredox C–H alkylation of **ON 18** with ethyl boronic acids, and (C) the catechol.

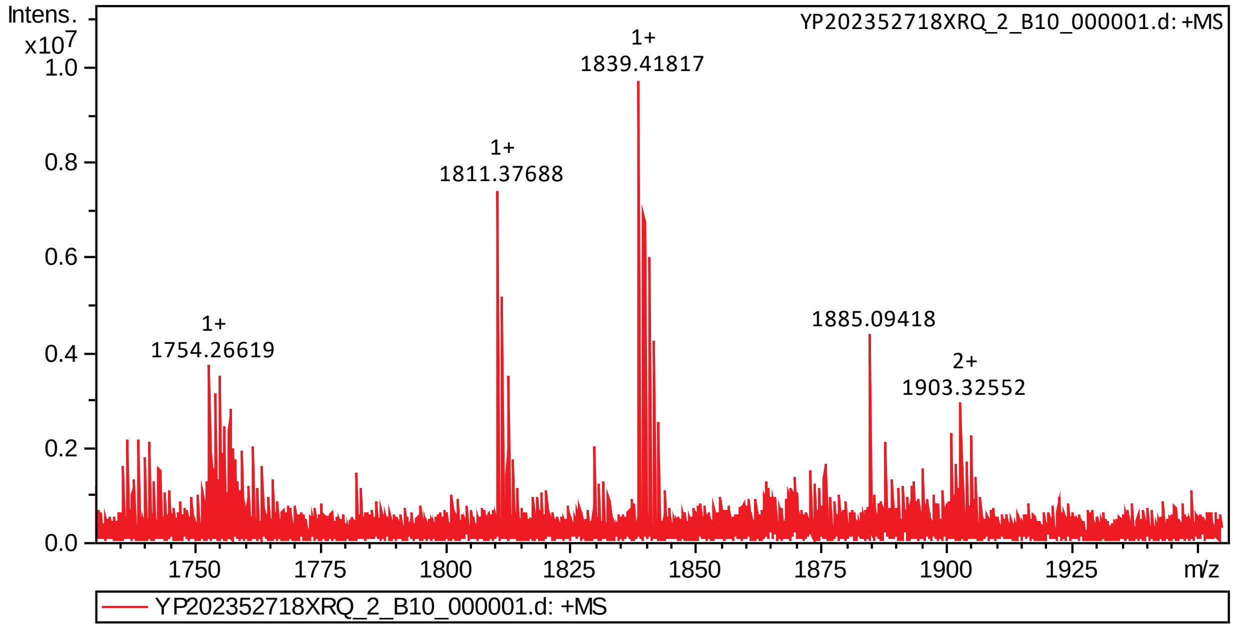

Supplementary Figure 78. Identification of G modification by MALDI-FTMS analysis of the reaction system of **ON 18**. Mass spectrum (MS) of desired product after ethylation of **ON 18**.

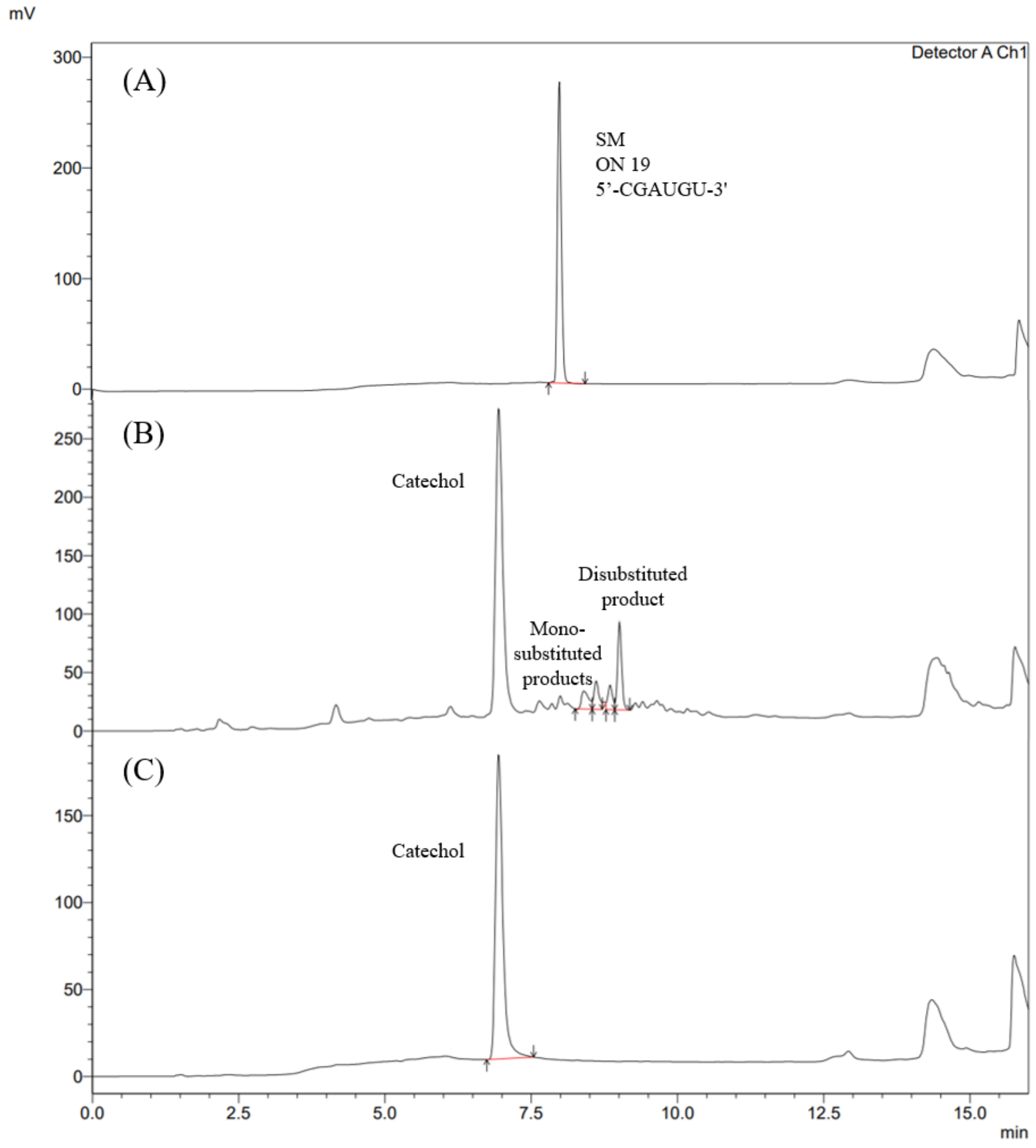

Supplementary Figure 79. Reverse-phase HPLC traces of (A) **ON 19**_a prepared by solid-phase RNA synthesis, (B) reaction mixture following catechol-promoted photoredox C–H alkylation of **ON 19** with ethyl boronic acids, and (C) the catechol.

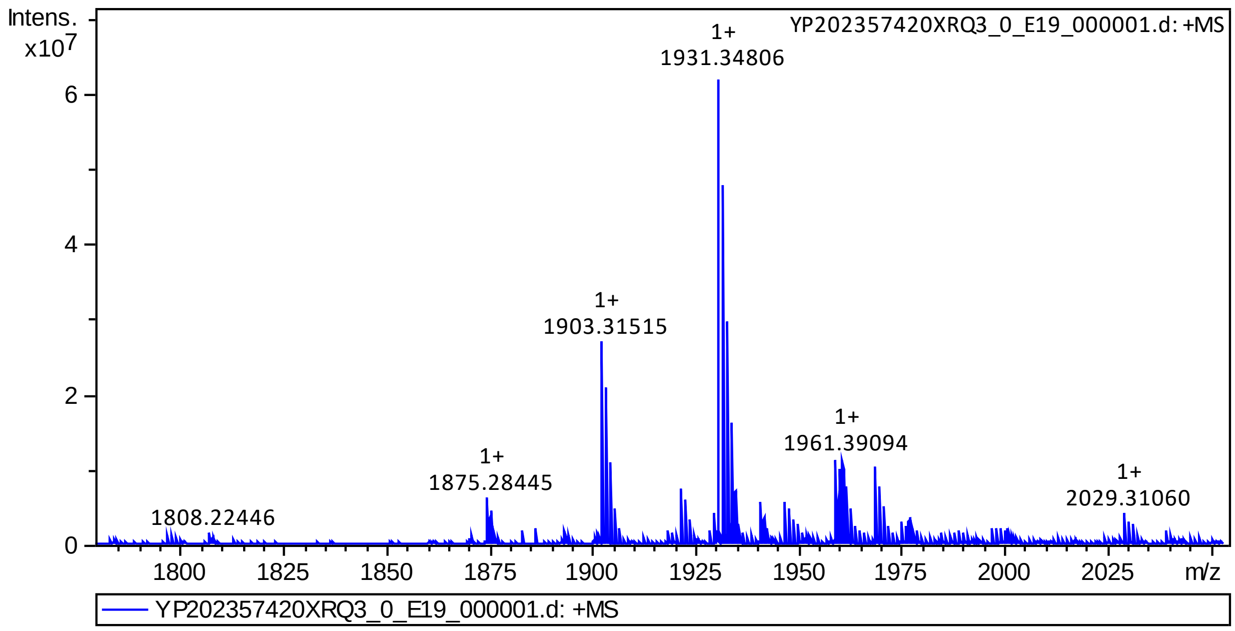

Supplementary Figure 80. Identification of G modification by MALDI-FTMS analysis of the reaction system of **ON 19**. Mass spectrum (MS) of desired product after ethylation of **ON 19**.

8.4. Substrate scope of C–H ethylation of dsDNA oligonucleotides

Supplementary Table 22. Substrate scope of C–H ethylation of the dsDNA oligonucleotides [a]

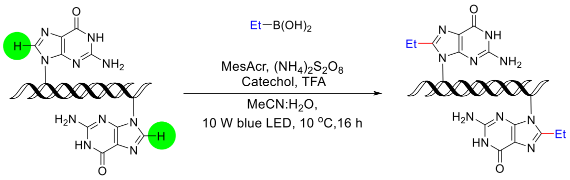

Entry	Oligonucleotides & Sequence	Calcd. molecular weight	Exptl. m/z (LTQ-XL)
1[e]	**ON 20** 5'-d(GAT CTA TTA CGC T)-3' 3'-d(CTA GAT AAT GCG A)-5'	3952.6621 [b]	3952.9
		3980.7161 [c]	*N.D.*
		4010.7171 [b]	4011.2 [M+H]$^+$
		4038.7711 [c]	*N.D.*
		4066.8251 [d]	*N.D.*
2	**ON 20-1** 5'-d(GAT CTA TTA CGC T)-3'	3952.6621 [b]	3952.8
		3980.7161 [c]	3982.1 [M+2H]$^+$
3	**ON 20-2** 3'-d(CTA GAT AAT GCG A)-5'	4010.7171 [b]	4009.0 [M-2H]$^-$
		4038.7711 [c]	*N.D.*
		4066.8251 [d]	*N.D.*

[a] Condition: Oligonucleotide (100 nmol), ethylboronic acid (400.0 equiv.), MesAcr (50 mol%), $(NH_4)_2S_2O_8$ (200.0 equiv.), TFA (50.0 equiv.), and catechol (100.0 equiv.) in MeCN (0.25 mL), H_2O (0.25 mL), irradiated by 10 W blue LED at 4 °C for 16 h. [b] Data of monosubstituted product(s). [c] Data of disubstituted product. [d] Data of trisubstituted product. **LTQ-XL**, Linear Ion Trap Mass Spectrometer. [e] The cosolvent and lack of salts may not support double-stranded hybridization, and it is possible that the strands are reacting in single-stranded form.

RP-HPLC profiles of dsDNA oligonucleotides

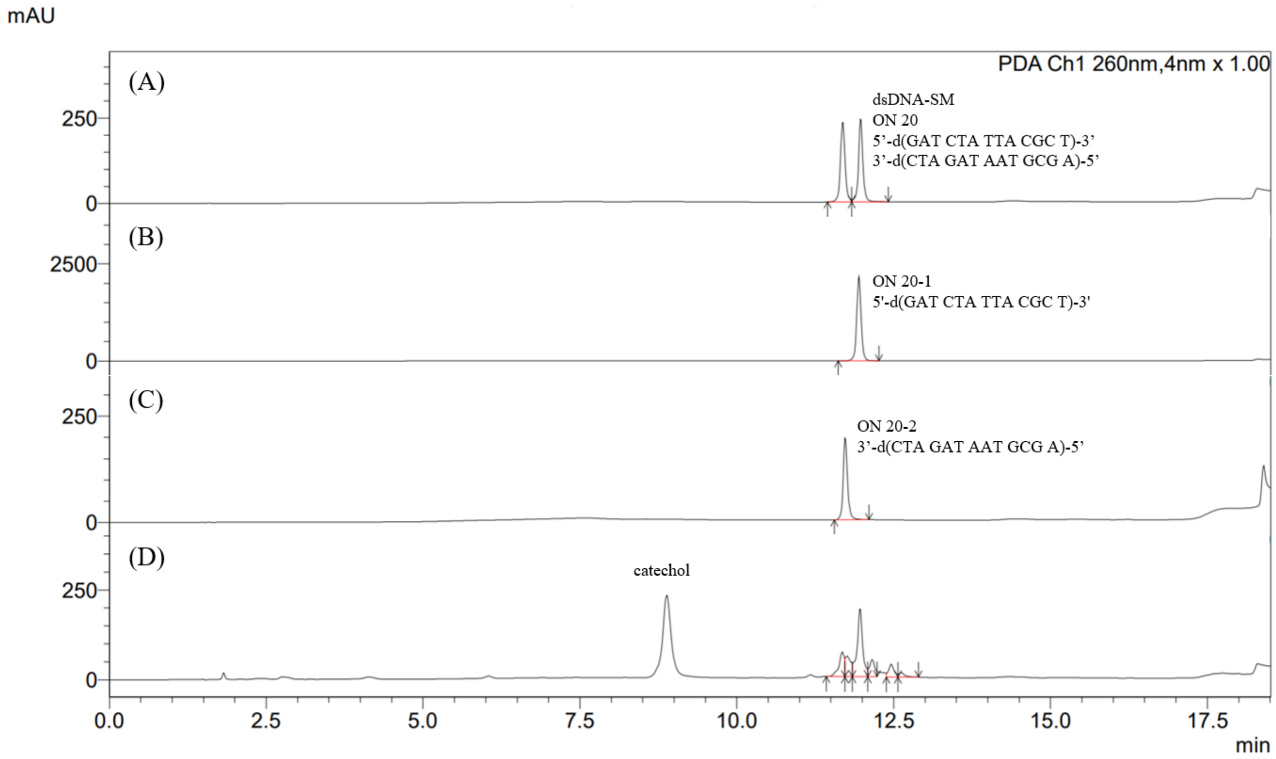

Supplementary Figure 81. Reverse-phase HPLC traces of (A) **ON 20**_a prepared by solid-phase DNA synthesis, (B) **ON 20-1**_a prepared by solid-phase DNA synthesis, (C) **ON 20-2**_a prepared by solid-phase DNA synthesis, and (D) reaction mixture following catechol-promoted photoredox C–H alkylation of **ON 20** with ethyl boronic acids.

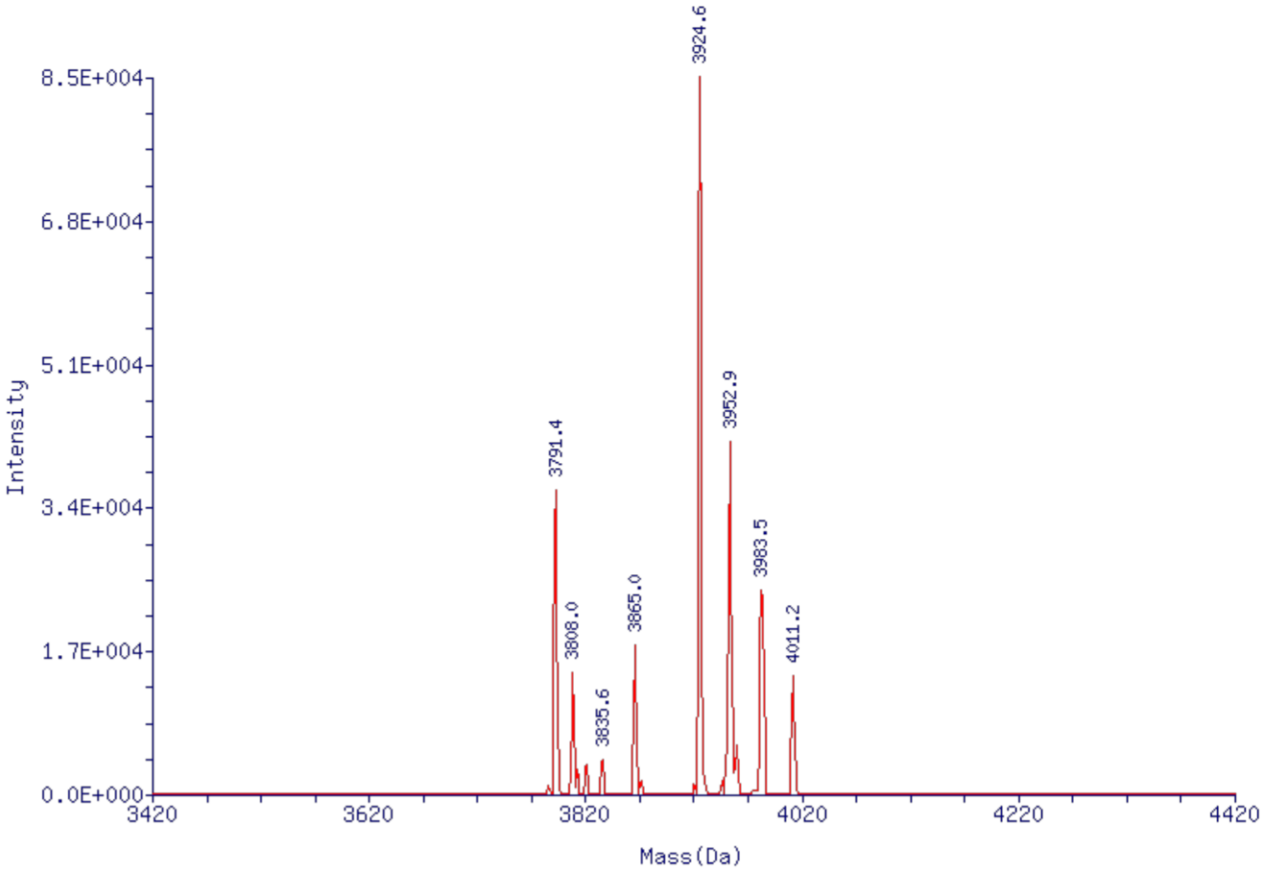

Supplementary Figure 82. Identification of G modification by LTQ XL analysis of the reaction system of **ON 20**. Mass spectrum (MS) of desired product after ethylation of **ON 20**.

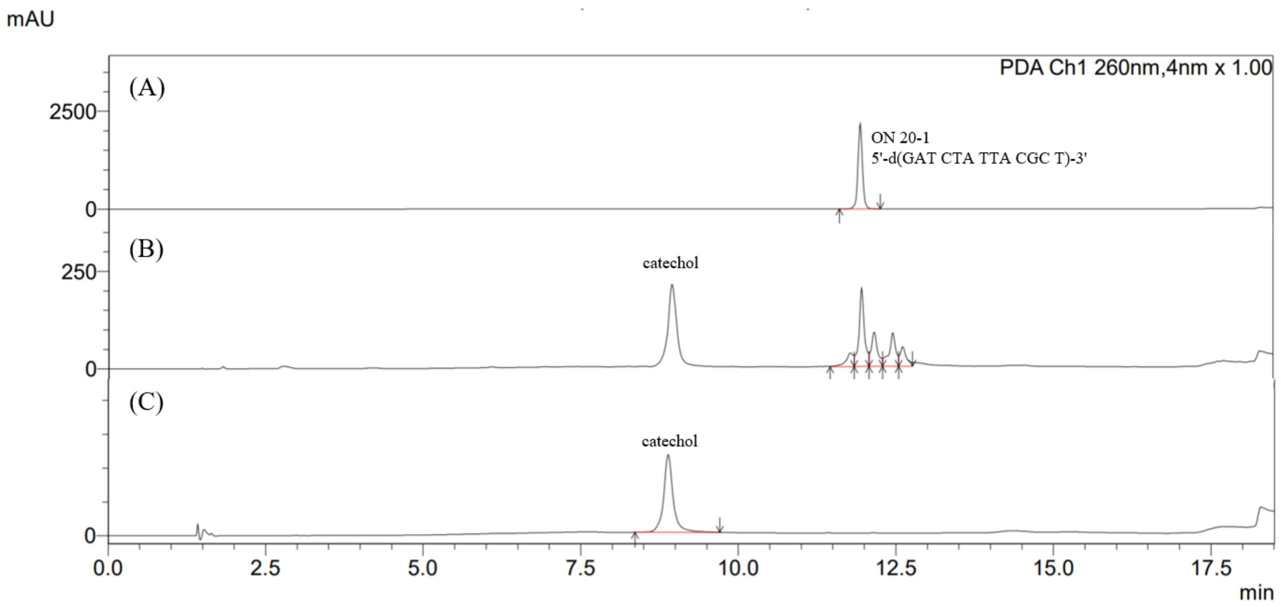

Supplementary Figure 83. Reverse-phase HPLC traces of (A) **ON 20-1**_a prepared by solid-phase DNA synthesis, and (B) reaction mixture following catechol-promoted photoredox C–H alkylation of **ON 20-1** with ethyl boronic acids.

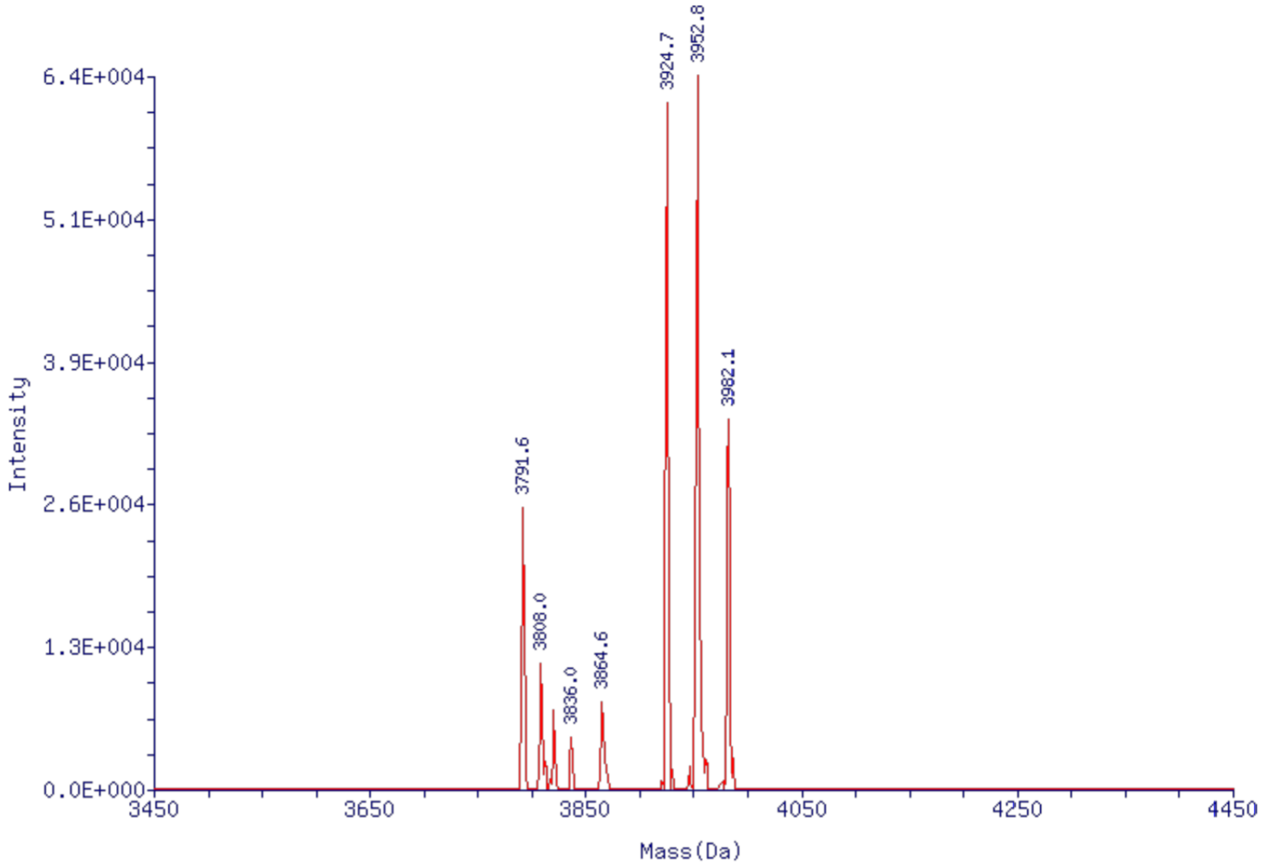

Supplementary Figure 84. Identification of G modification by LTQ XL analysis of the reaction system of **ON 20-1**. Mass spectrum (MS) of desired product after ethylation of **ON 20-1**.

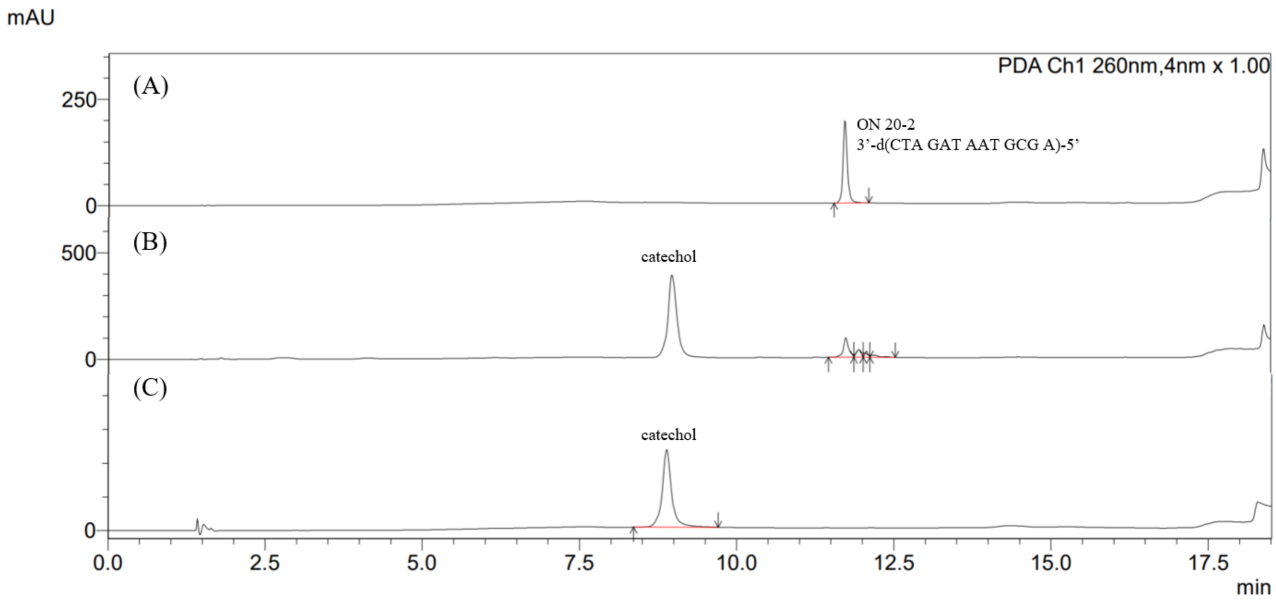

Supplementary Figure 85. Reverse-phase HPLC traces of (A) **ON 20-2**_a prepared by solid-phase DNA synthesis, and (B) reaction mixture following catechol-promoted photoredox C–H alkylation of **ON 20-2** with ethyl boronic acids.

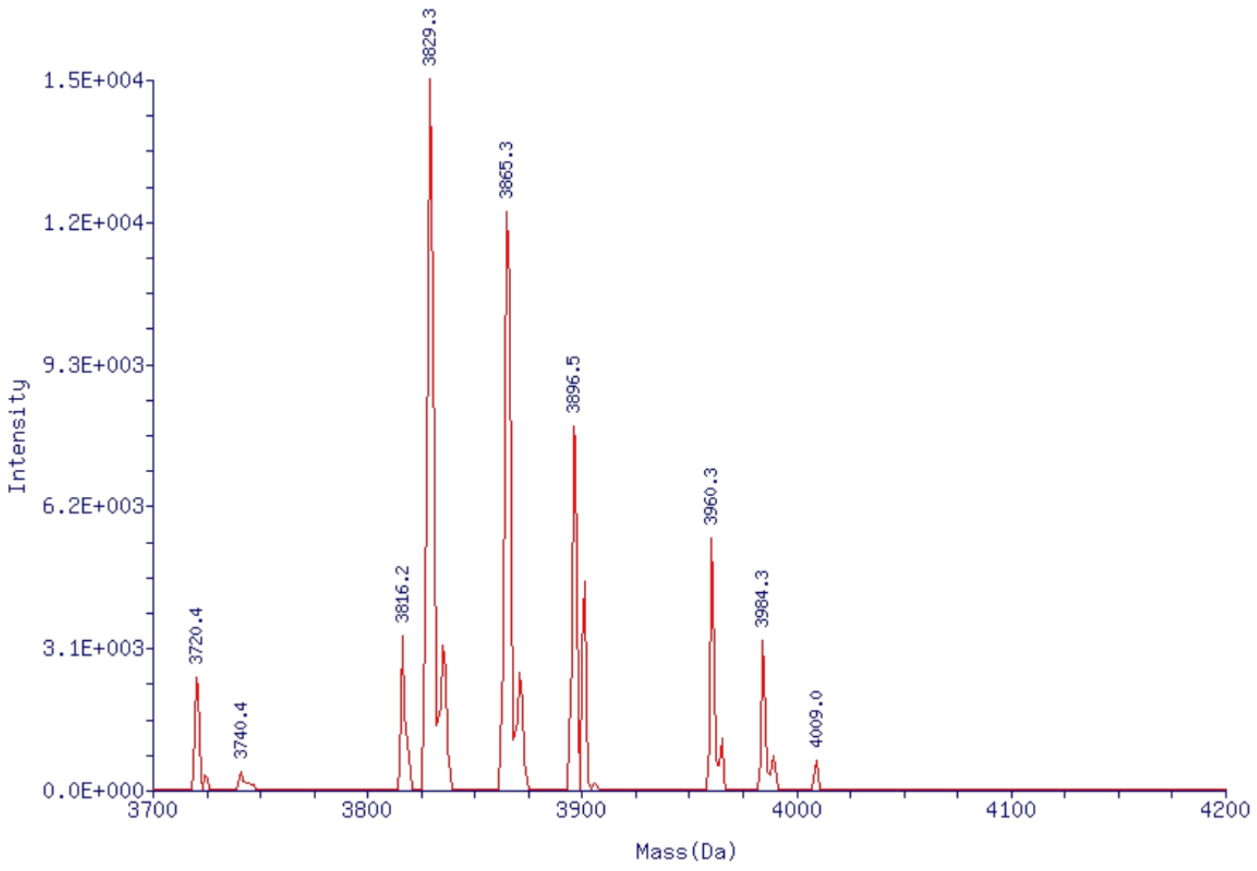

Supplementary Figure 86. Identification of G modification by LTQ XL analysis of the reaction system of **ON 20-2**. Mass spectrum (MS) of desired product after ethylation of **ON 20-2**.

8.5. Analysis of modification site of alkylated oligonucleotides

8.5.1. Tandem mass spectrometry on ethylated oligonucleotides

Alkylation of oligonucleotides ON 1 with ethylboronic acid

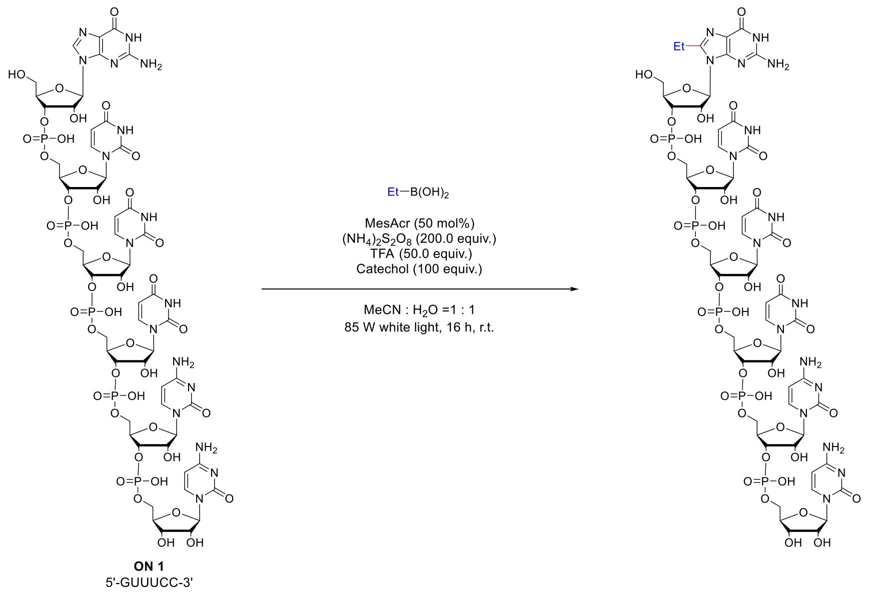

A) Fragment 1 [M +1]

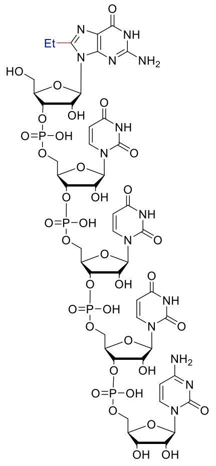

Chemical Formula: $C_{48}H_{62}N_{14}O_{36}P_4$
Exact Mass: 1534.2402
observed mass: 1535.14818

Supplementary Figure 87. Alkylation of oligonucleotides **ON 1** with ethylboronic acid, and fragment of ethylated **ON 1**

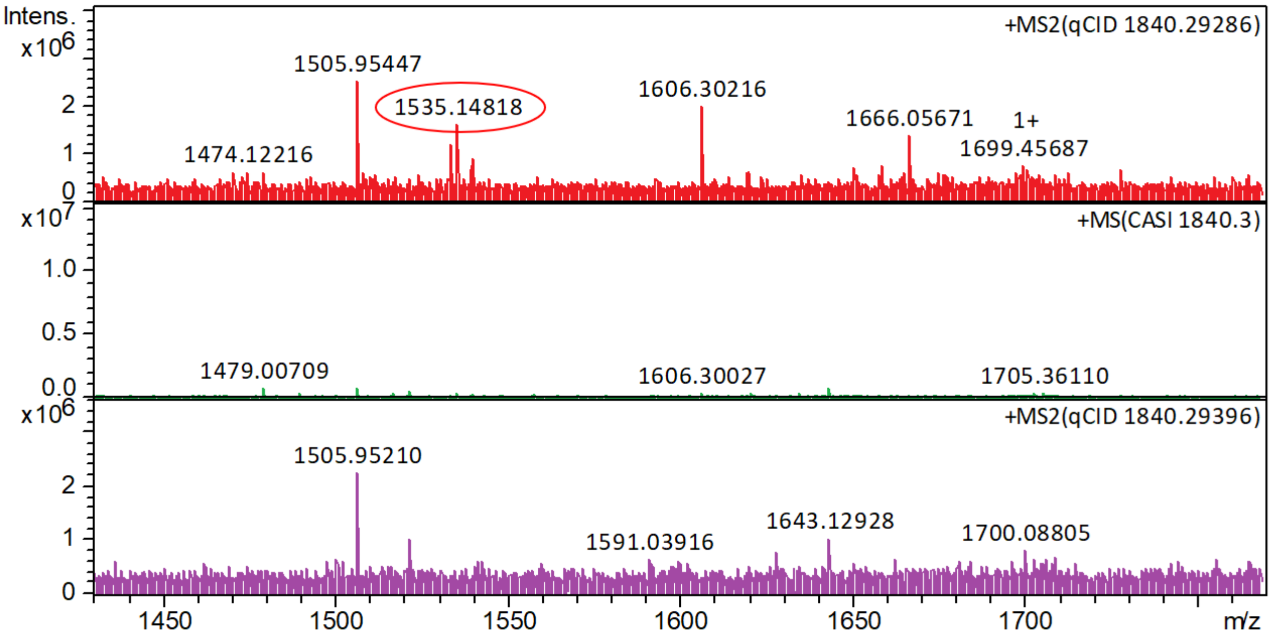

Supplementary Figure 88. Mass spectrometric analysis of modified oligonucleotide **ON 1** after reaction with the ethylboronic acid, analysis by MALDI-FTMS.

Alkylation of oligonucleotides ON 11 with ethylboronic acid

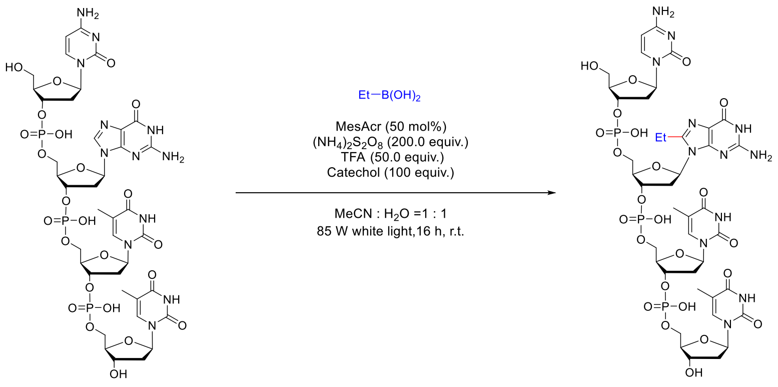

ON 11
5'-CGTT-3'

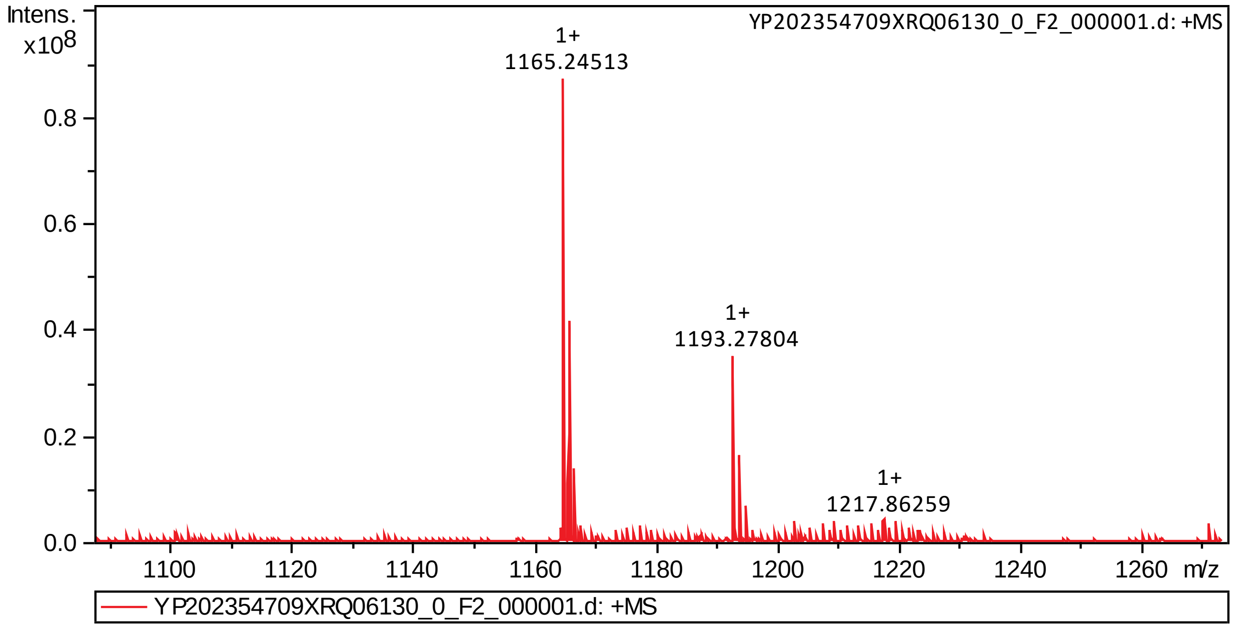

Supplementary Figure 89. Mass spectrum (MS) of ethylated **ON 11**, analysis by MALDI-FTMS.

a

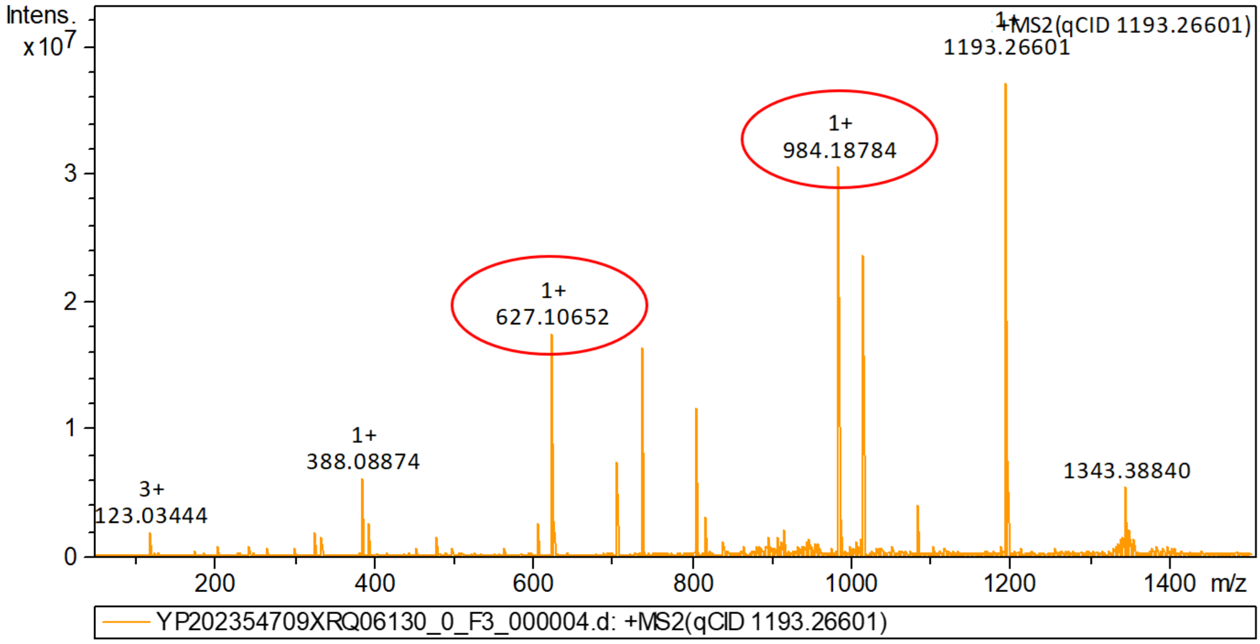

b

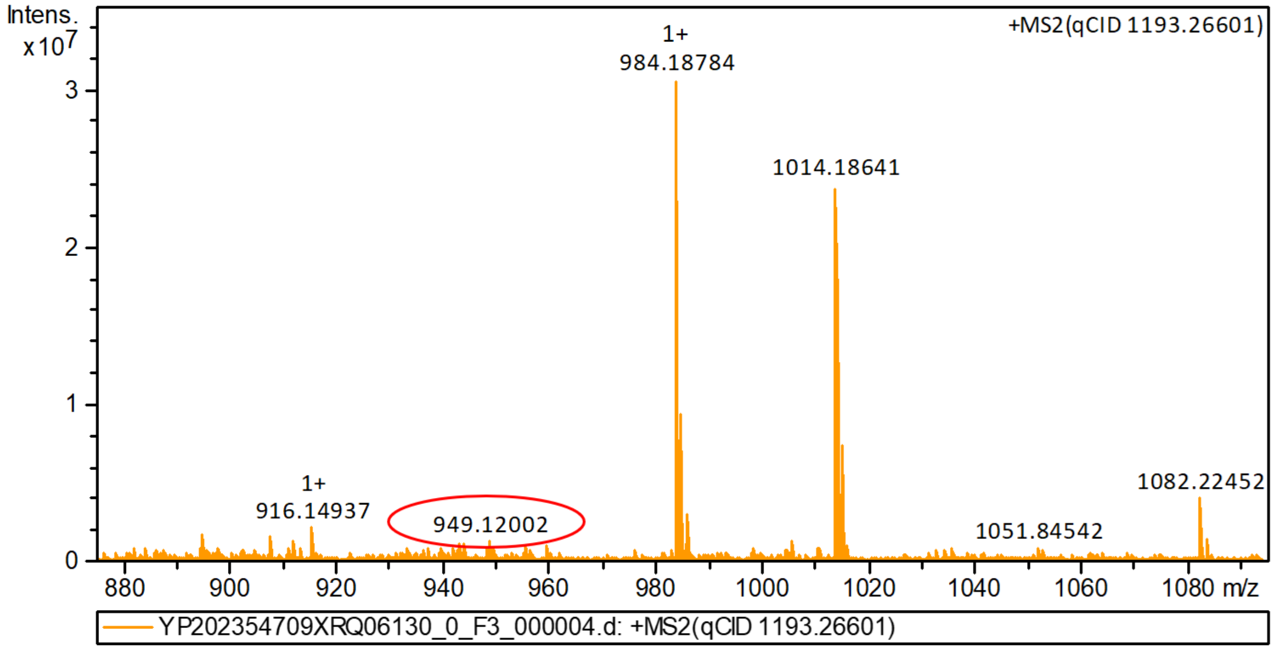

Supplementary Figure 90. MS/MS fragmentation of ethylated **ON 11**, analysis by MALDI-FTMS.

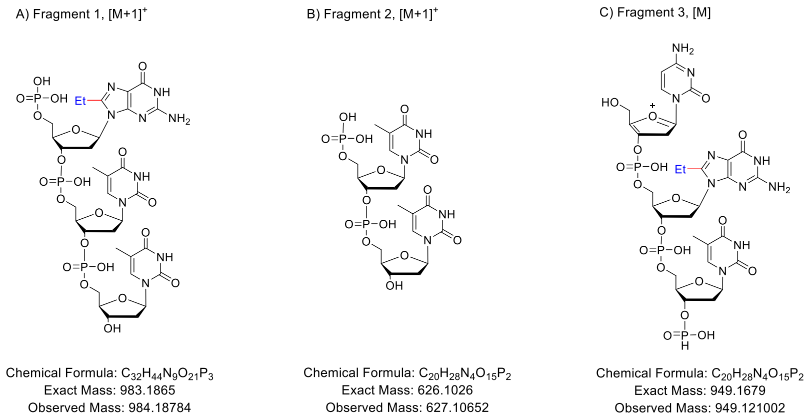

Supplementary Figure 91. Fragment of ethylated **ON 11**

8.5.2. Enzyme digestion of C8-ethylated ON 11

Steps of using Nucleoside Digestion Mix (NEB #M0649) to digest ethylated ON 11.

1. After the ethylation of **ON 11** is completed, the mixture was directly purified by HPLC without concentration. The column XBridge Premier Peptide BEH C18 Column (300Å, 2.5 um, 4.6 mm × 100mm) and eluents MeCN/TEAA buffer (pH=7.0) were used to separate the pure product. Finally, utilizing a freeze dryer to concentrate the product solution, ensuring that TEAA is completely

removed to avoid affecting the pH value of subsequent enzymatic digestion reaction.

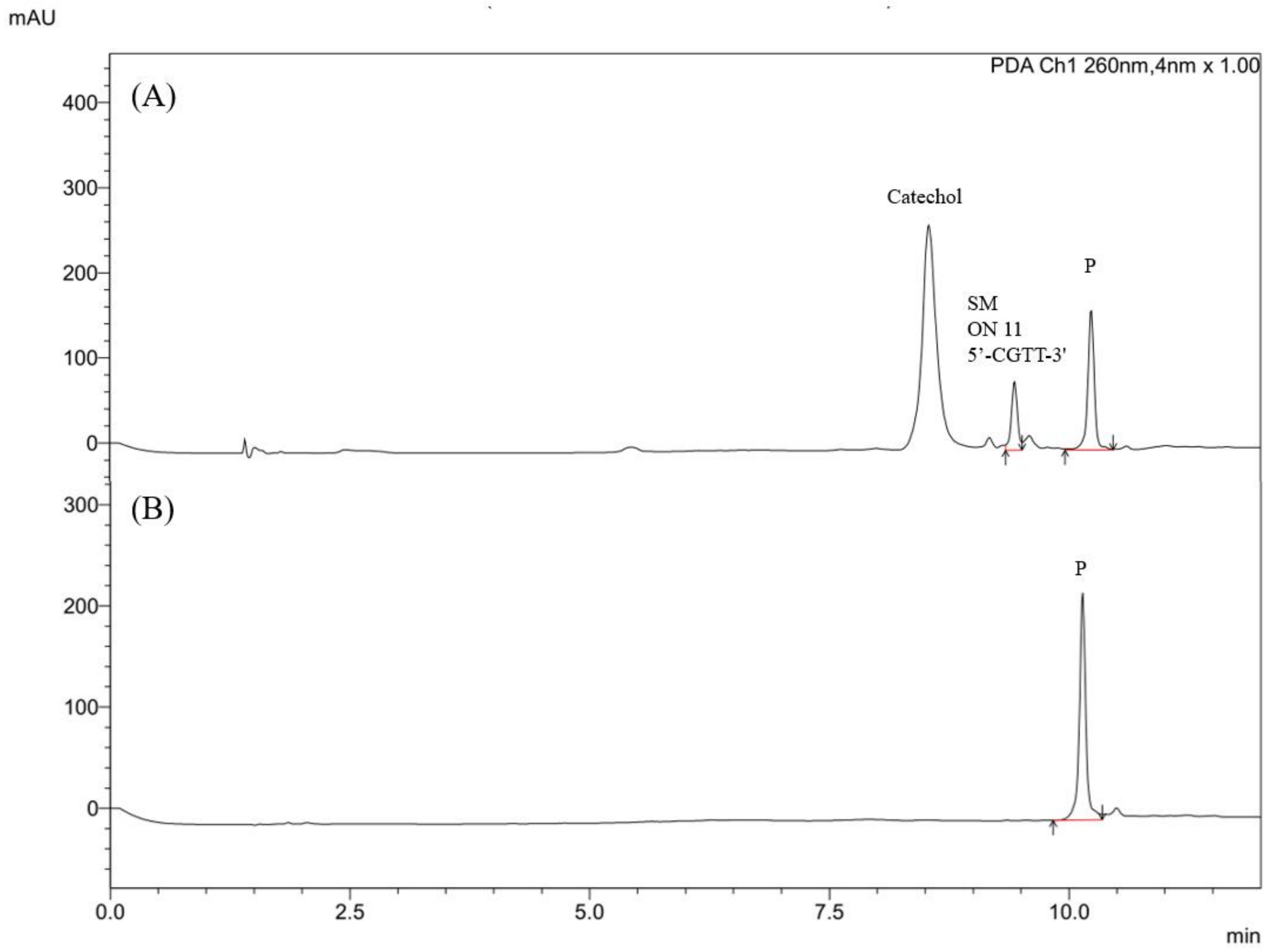

Supplementary Figure 92. Reverse-phase HPLC traces of (A) reaction mixture following catechol-promoted photoredox C–H alkylation of **ON 11** with ethyl boronic acids, and (B) the isolated product of **ON 11** with ethylation.

2. Set up the following reaction.

**Reagents and Substrate**	**Amount**
Double Distilled Water	up to 20 μl
Nucleoside Digestion Mix Reaction Buffer (10X)	2 μl
DNA or RNA Substrate	up to 1 μg
Nucleoside Digestion Mix	1 μl

3. Incubate at 37°C for 1 hour.

4. Use HPLC to directly analyze the reaction mixture solution. The following HPLC analysis showed the reaction mixture, standard samples of C8-ethylated dexoyguanosine, 2’-deoxycytidine, and thymidine.

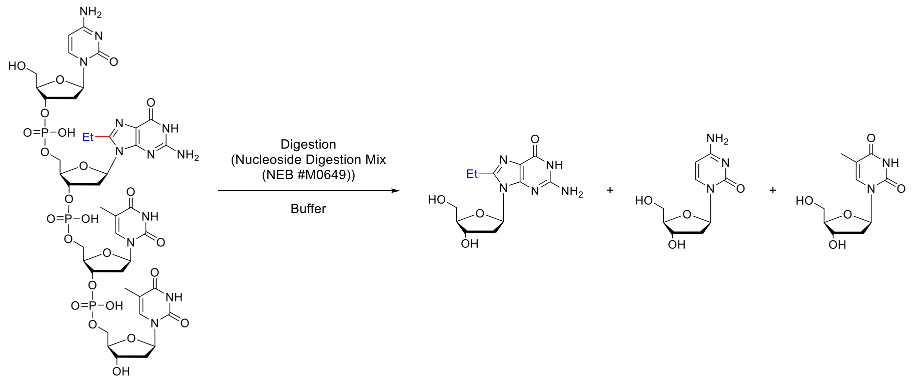

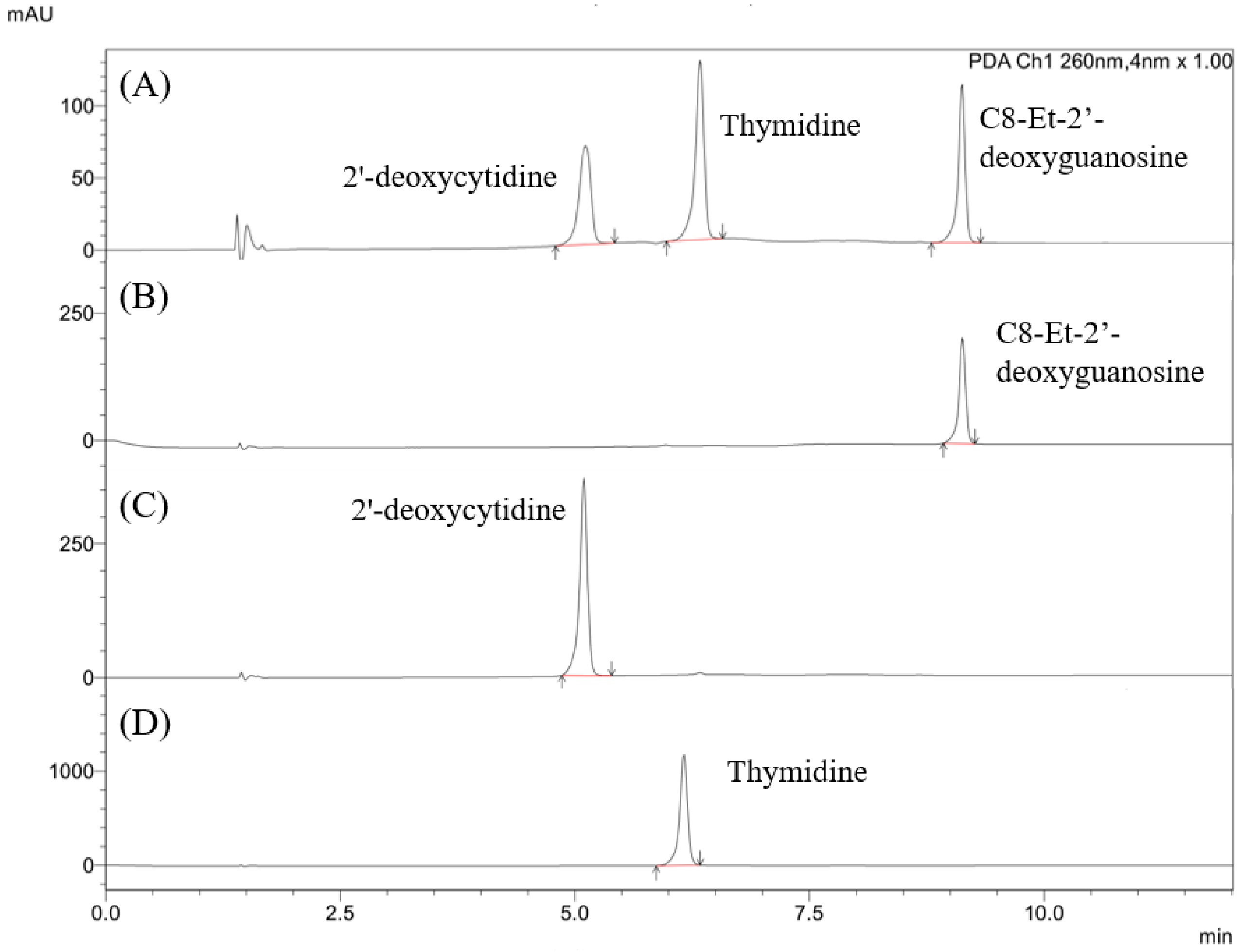

Supplementary Figure 93. Reverse-phase HPLC traces of (A) reaction mixture following nuclease digestion (M0649S) of the isolated product of **ON 11** with ethylation, (B) the standard sample of C8-Et deoxyguanosine, (C) the standard sample of 2’-deoxycytidine, and (D) the standard sample of thymidine.

9. Applications of the methodology regarding oligonucleotides

Bioconjugate with the biotin alkyne *via* click reaction [54, 55]

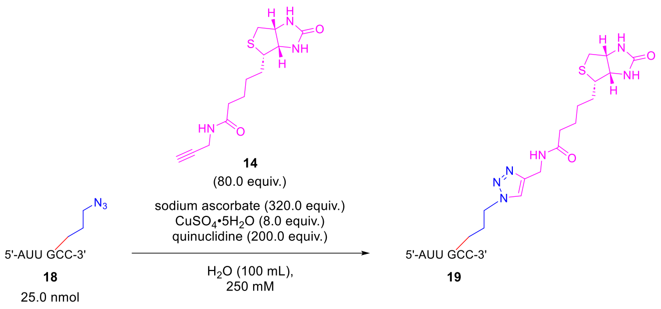

Supplementary Figure 94. Bioconjugation of **18**

Separation of compound 18

The method of synthesis compound **18** (see **Supplementary Table 19**, **Entry 2**). Next, the compound 19 was separated by utilizing the purification method for oligonucleotides (see General Information and **Supplementary Figure 3**).

Calculated and found MS data (see **Supplementary Figure 95** (B) & **Supplementary Figure 96**): MS-ESI m/z calculated exact mass: 1917.33; calculated $[M-2H]^{2-}$ 957.66; found $[M-2H]^{2-}$ 957.85. MALDI-FTMS m/z calcd. for $[M+H]^{+}$ 1918.3325 (Exact Mass); found 1918.30266.

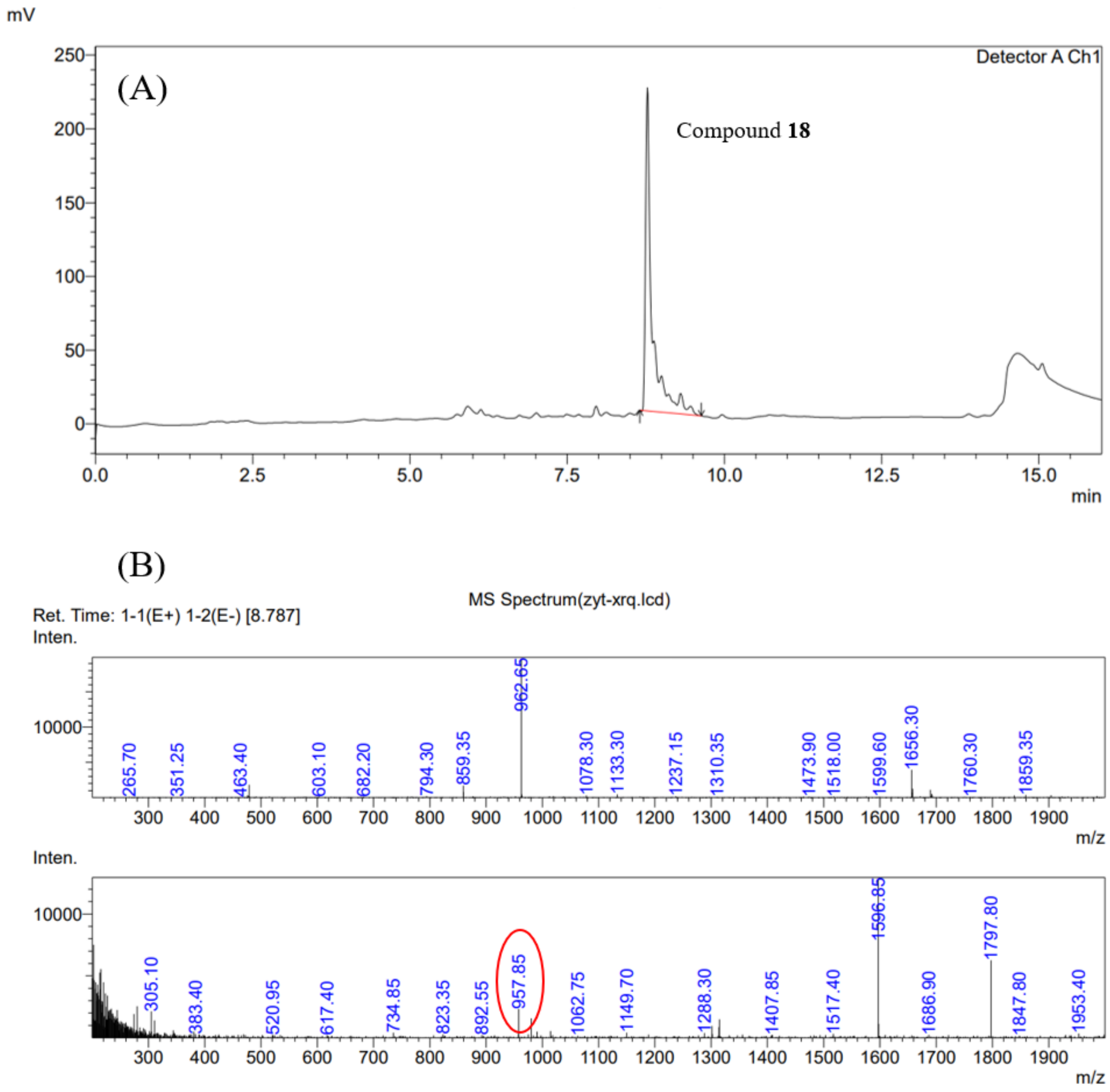

Supplementary Figure 95. HPLC-MS analysis of compound **18** (A) reverse-phase HPLC traces of compound **18**_separated by HPLC, and (B) mass spectrogram of compound **18**.

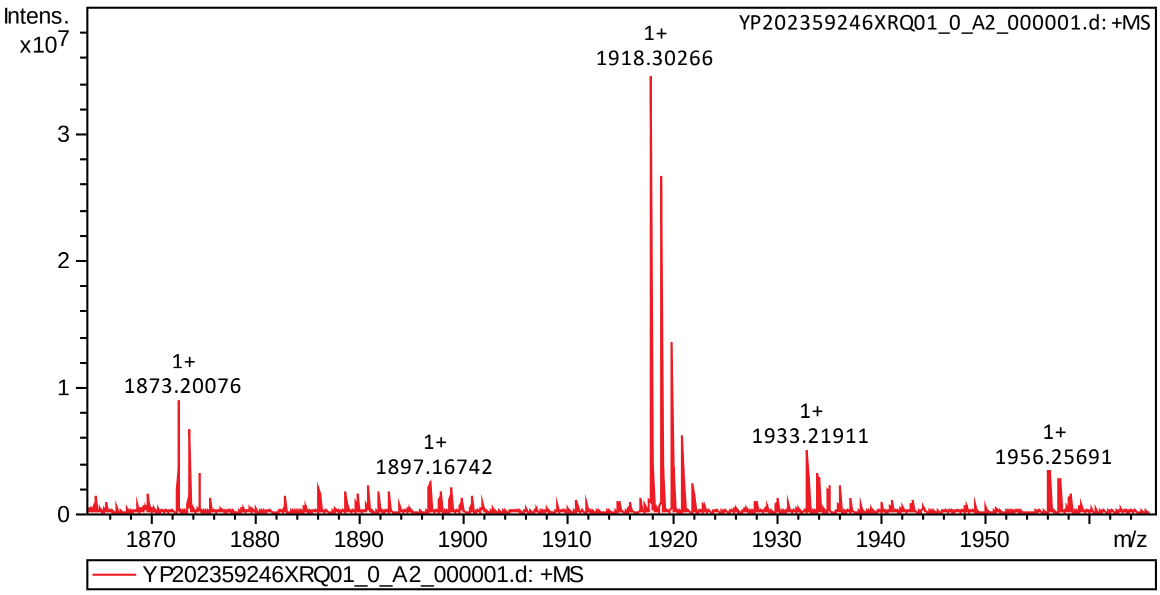

Supplementary Figure 96. Identification of G modification by MALDI-FTMS analysis of the reaction system of **ON 5**. Mass spectrum (MS) of desired product after alkylation of **ON 5**.

Pathway F

Biotin alkyne (0.56 mg, 2.0 μmol, 80.0 equiv.), compound **18** (0.048 mg, 25 nmol, 1.0 equiv.), copper (II) sulfate pentahydrate (0.05 mg, 0.2 μmol, 8.0 equiv.), sodium ascorbate (1.584 mg, 8.0 μmol, 320.0 equiv.), and the quinuclidine (0.56 mg, 5.0 μmol, 200.0 equiv.) were dissolved in 0.01 mL H_2O. The solution was stirred vigorously at room temperature, and the progress of the reaction was monitored by LC-MS, using the analytical method A. The desired product (compound **19**) was obtained in 44% LC-yield. MALDI-FTMS m/z calcd. for $[M+H]^+$ 2199.4523. found 2199.41382.

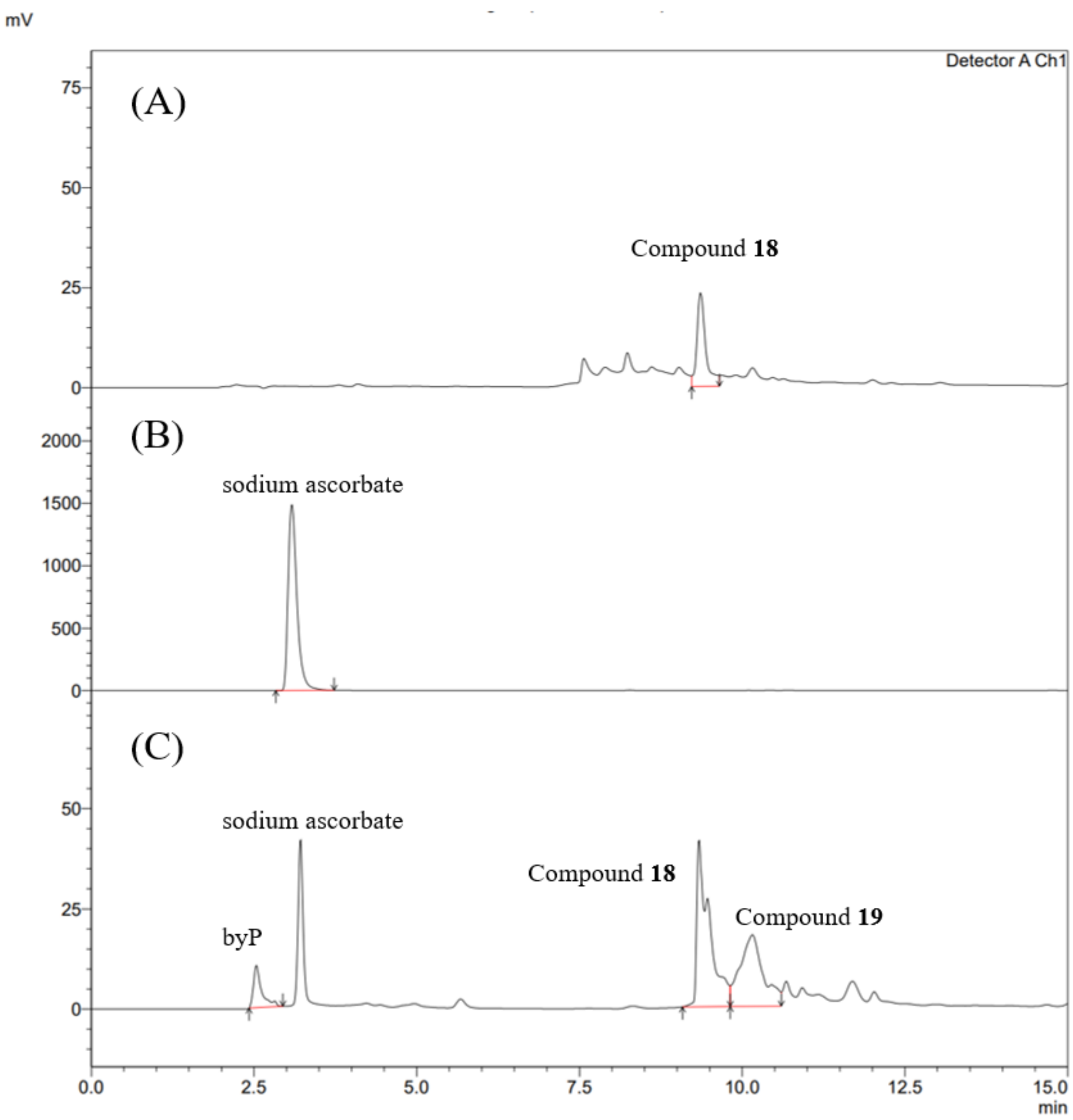

Supplementary Figure 97. Reverse-phase HPLC traces of (A) compound **18**_separated by HPLC, (B) the sodium ascorbate, and (C) reaction mixture following click reaction of compound **18** with biotin alkyne (compound **14**).

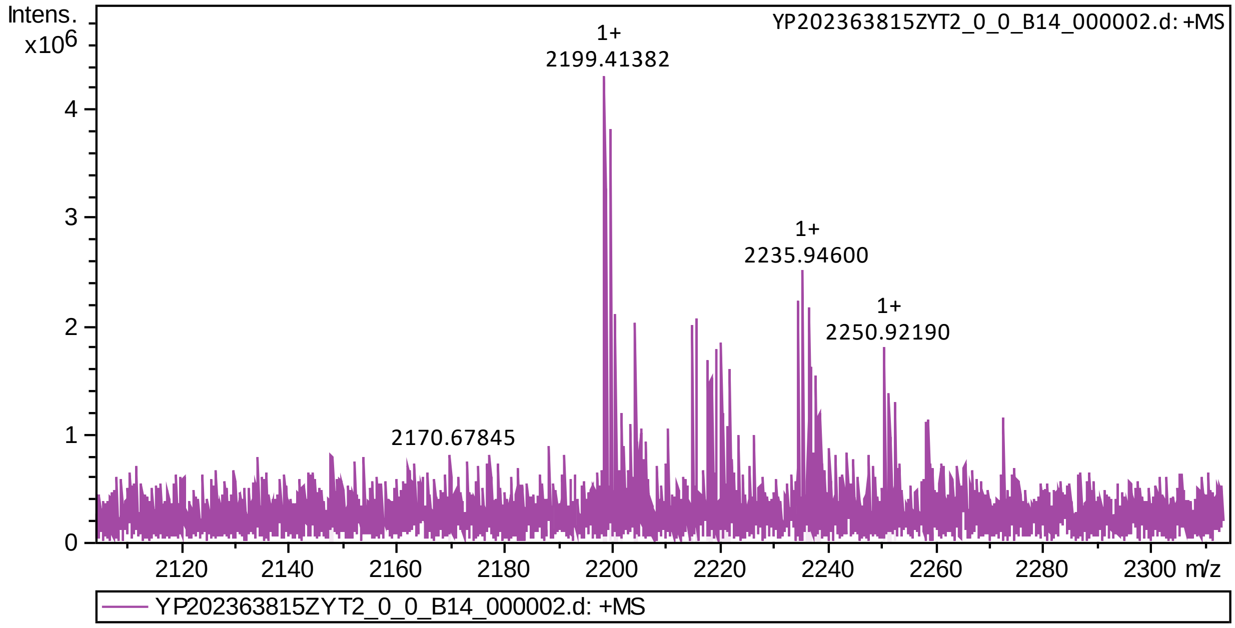

Supplementary Figure 98. Identification of G modification by MALDI-FTMS analysis of the reaction system of compound **18**. Mass spectrum (MS) of desired product compound **19** after click reaction of compound **18**.

10. NMR spectrum

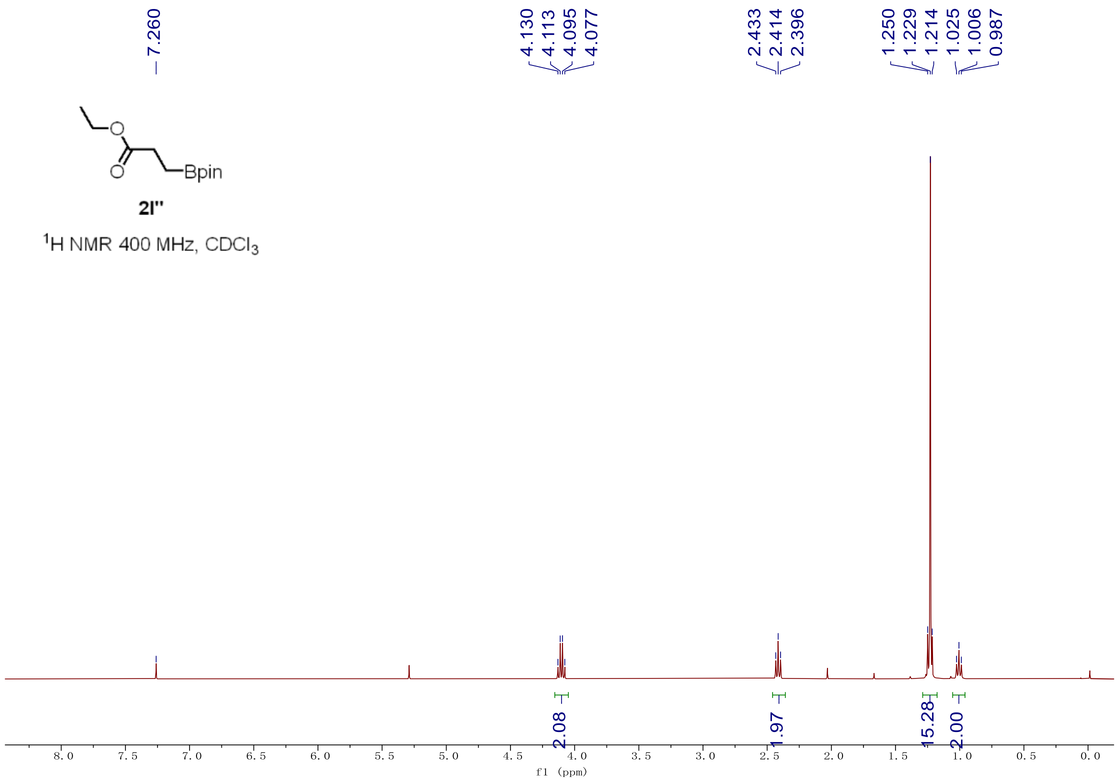

Supplementary Figure 99. ^{1}H NMR spectra of compound **2l''**

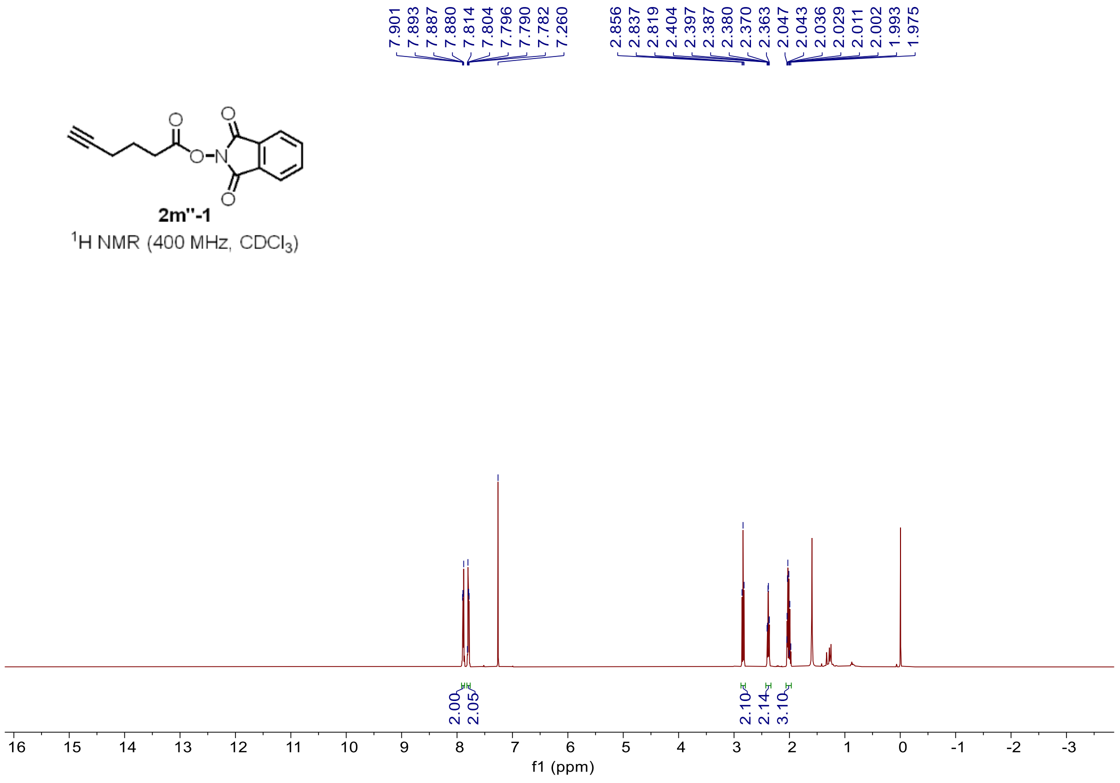

Supplementary Figure 100. ^{1}H NMR spectra of compound **2m''-1**

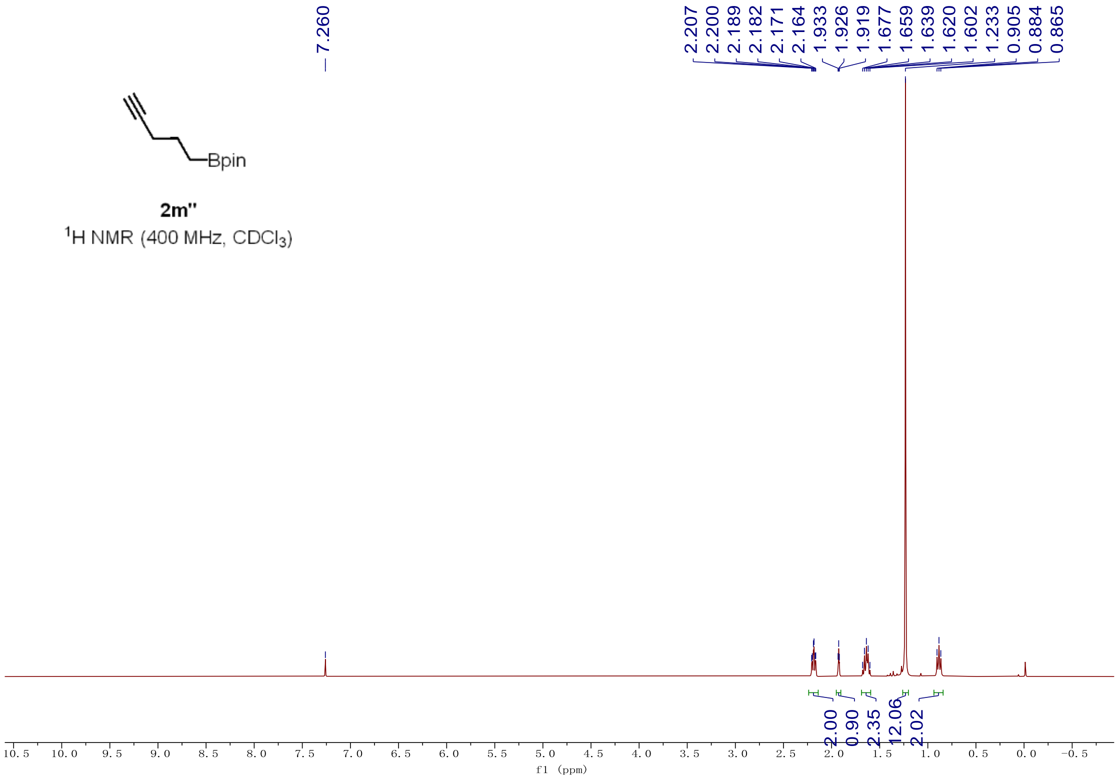

Supplementary Figure 101. ^{1}H NMR spectra of compound **2m''**

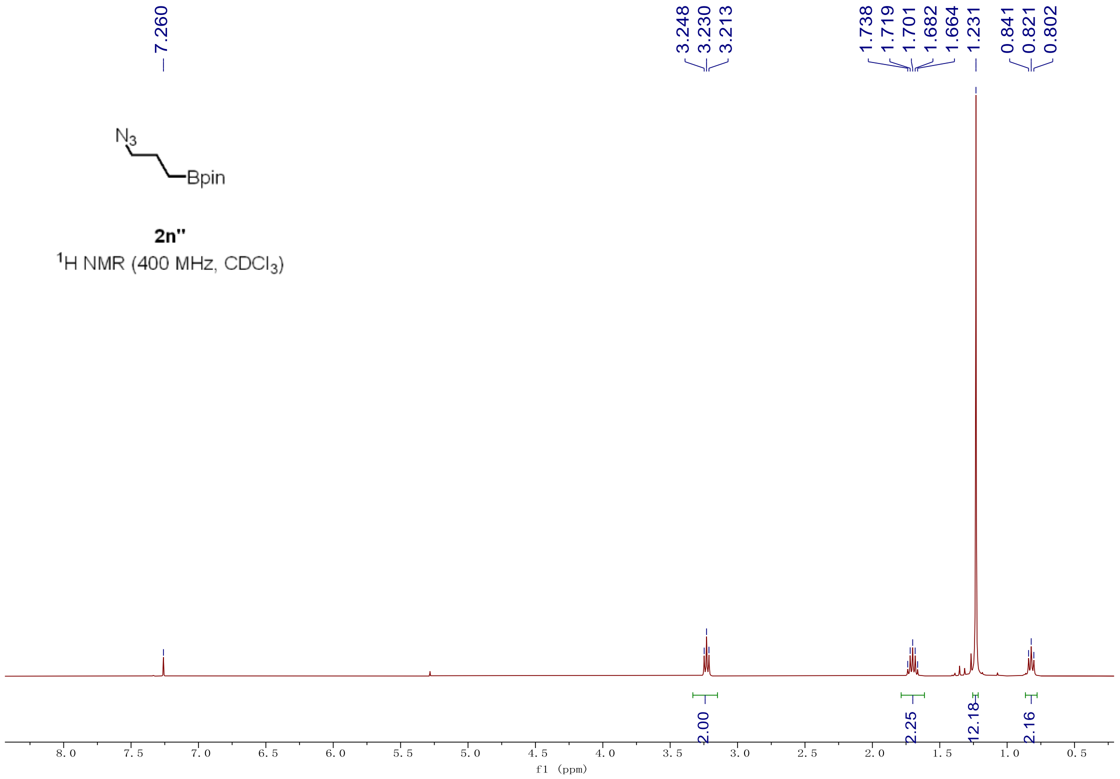

Supplementary Figure 102. ^{1}H NMR spectra of compound **2n''**

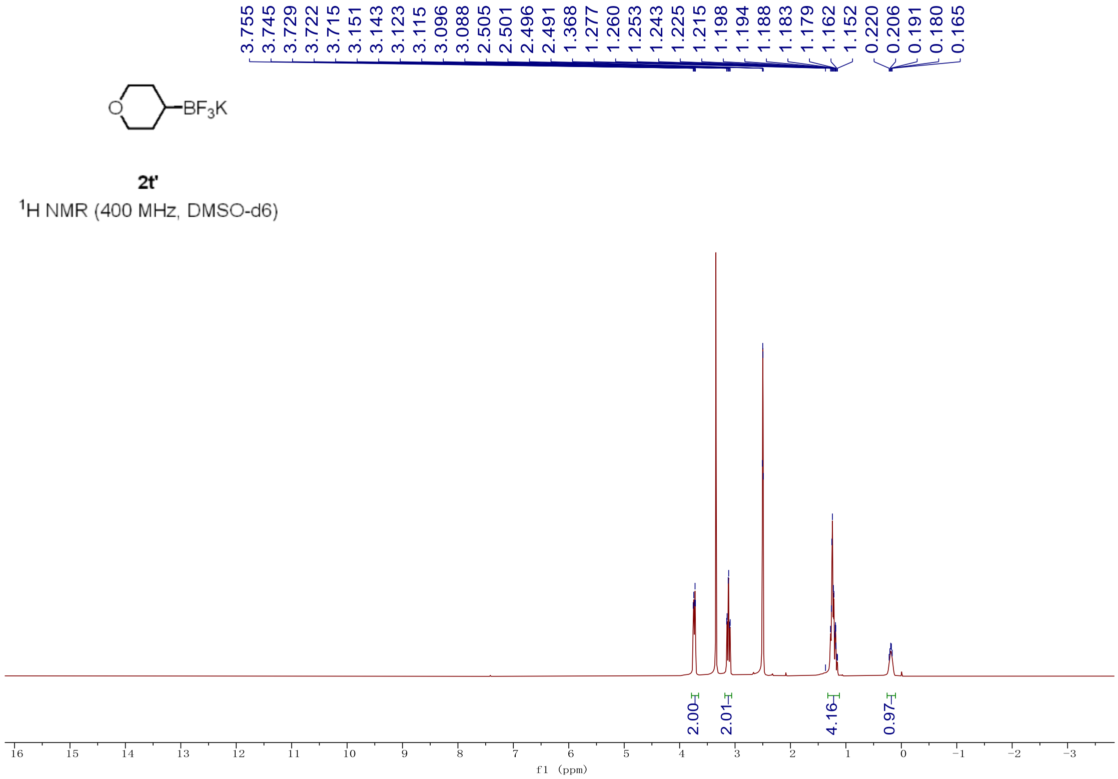

Supplementary Figure 103. ^{1}H NMR spectra of compound **2t'**

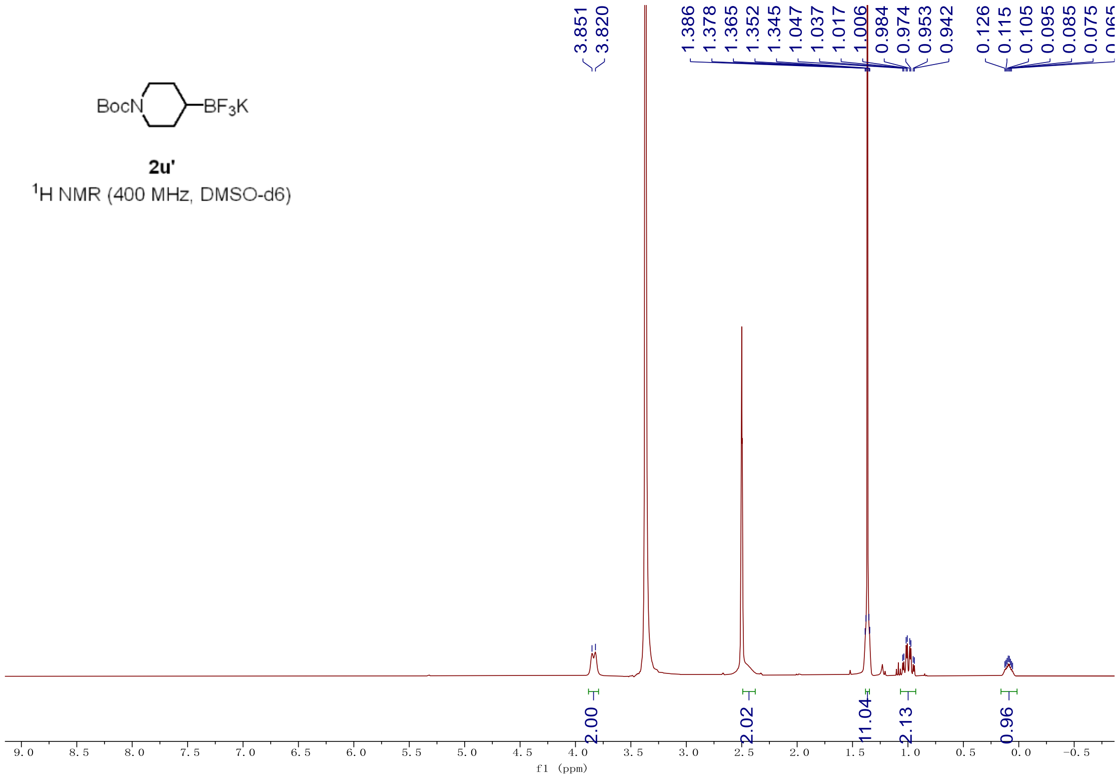

Supplementary Figure 104. ^{1}H NMR spectra of compound **2u'**

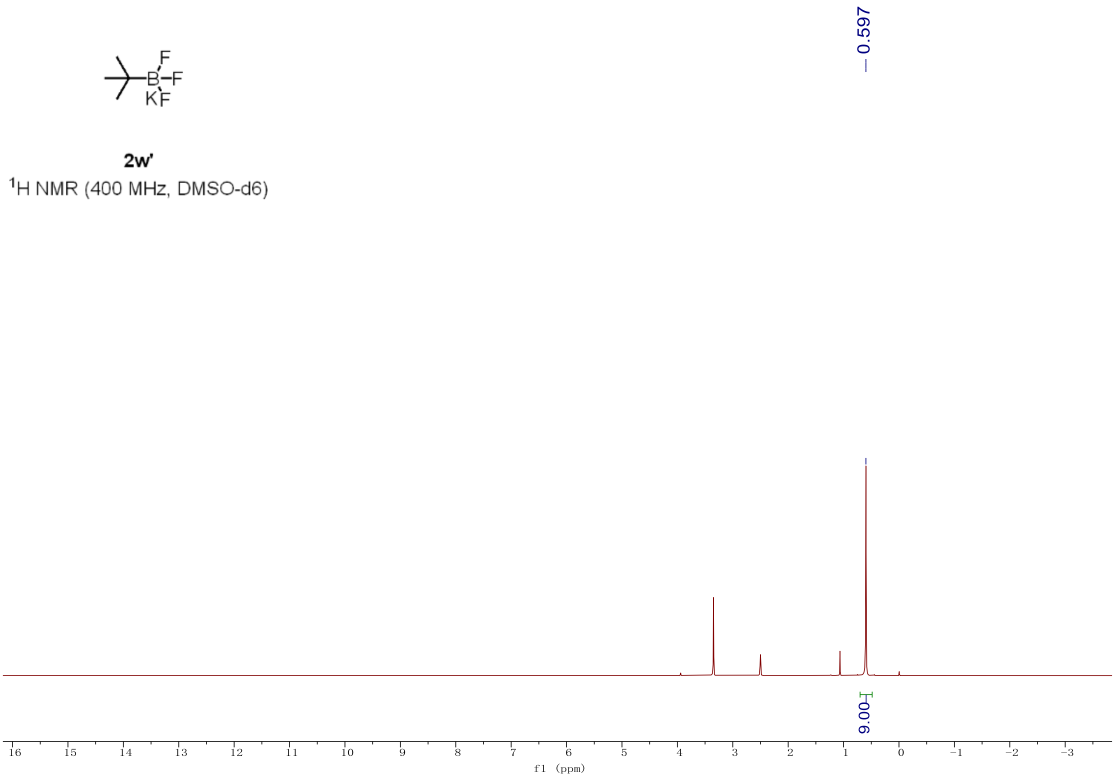

Supplementary Figure 105. ^{1}H NMR spectra of compound **2w'**

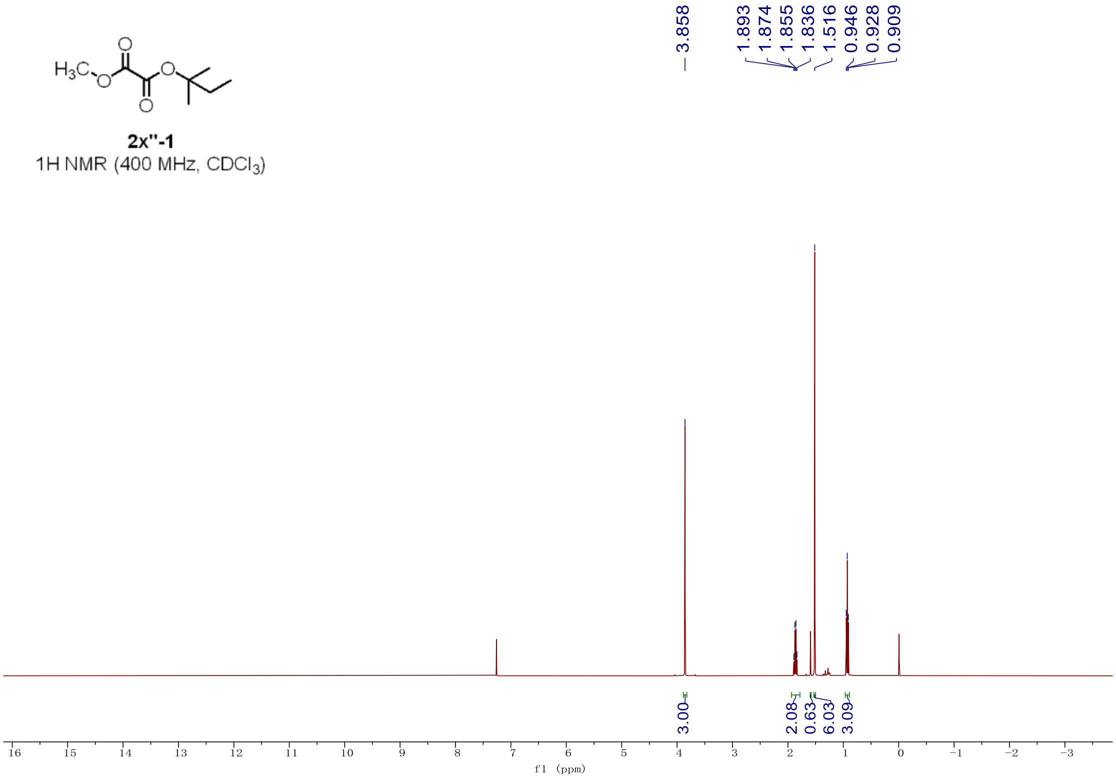

Supplementary Figure 106. ^{1}H NMR spectra of compound **2x''-1**

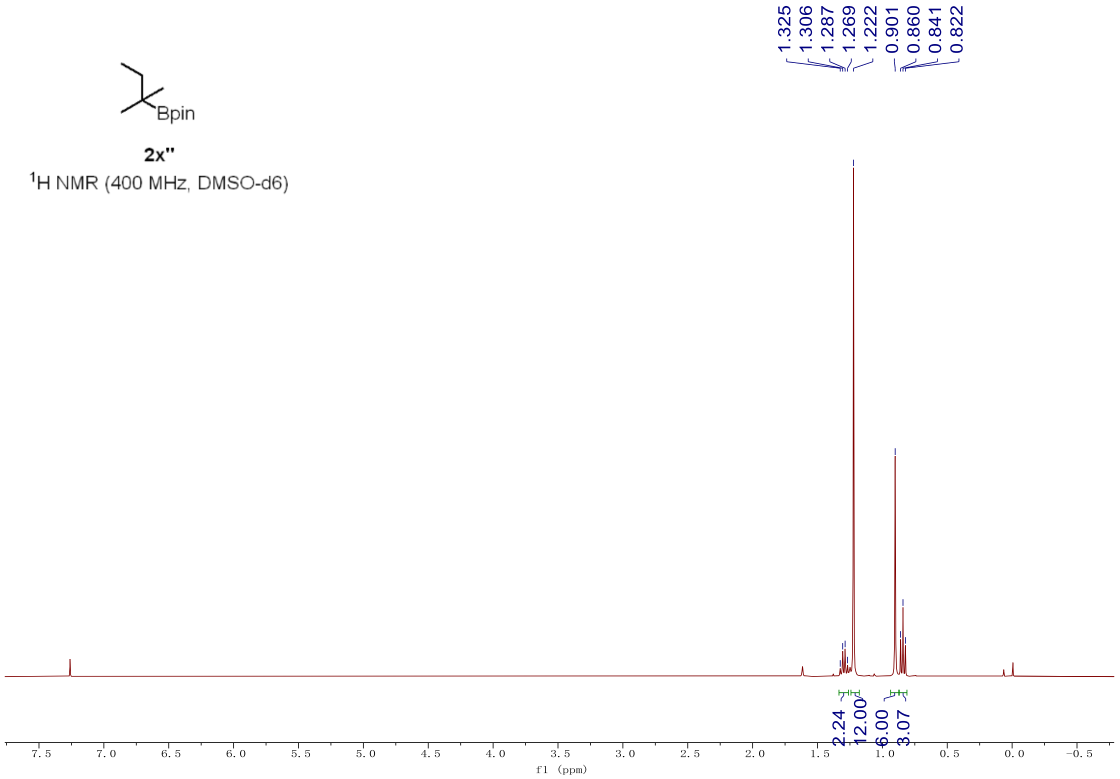

Supplementary Figure 107. ^{1}H NMR spectra of compound **2x''**

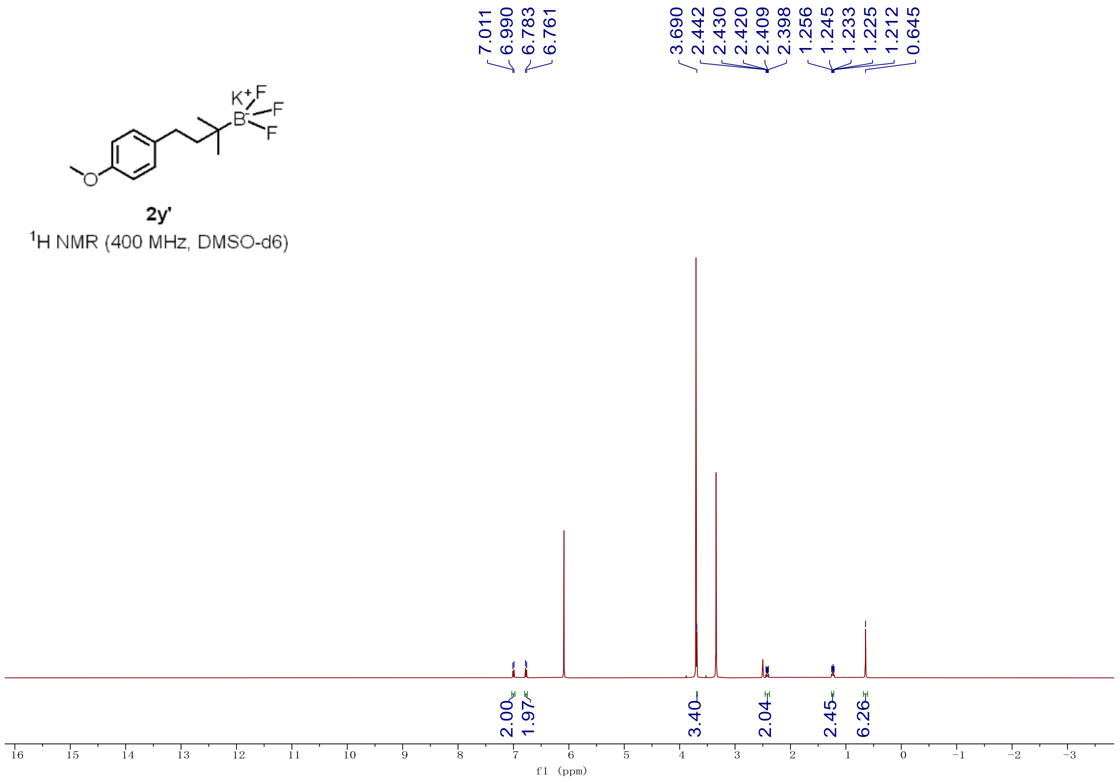

Supplementary Figure 108. ^{1}H NMR spectra of compound **2y'**

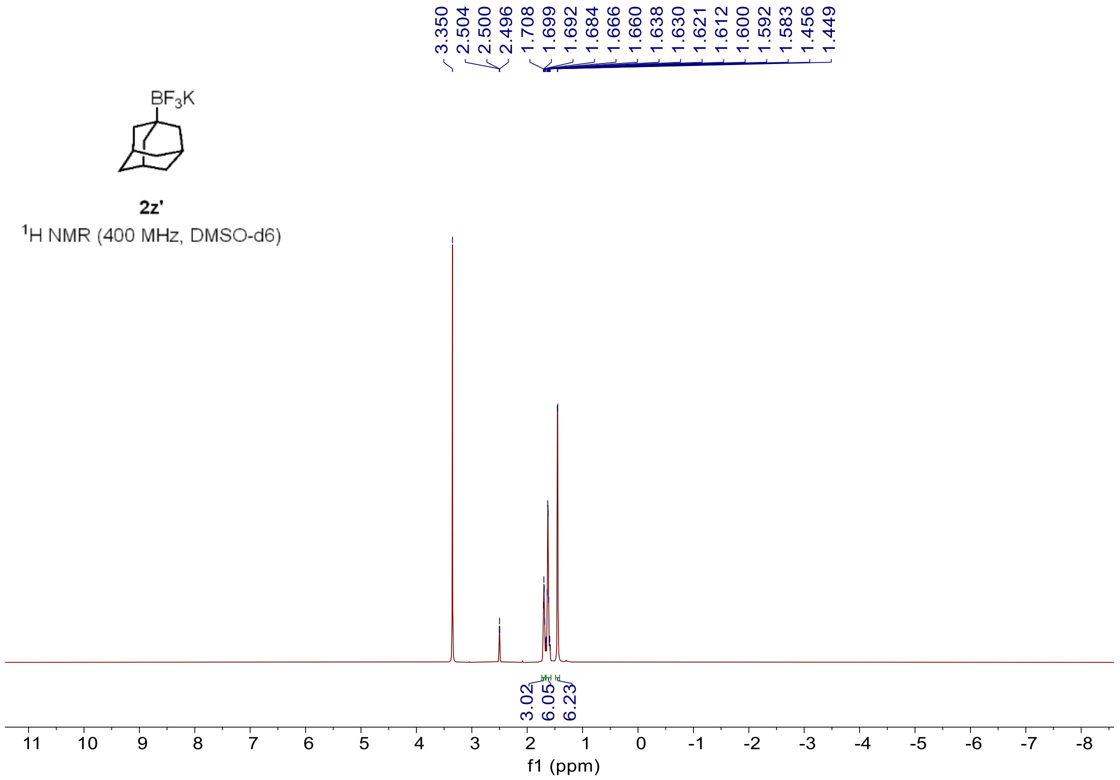

Supplementary Figure 109. ^{1}H NMR spectra of compound **2z'**

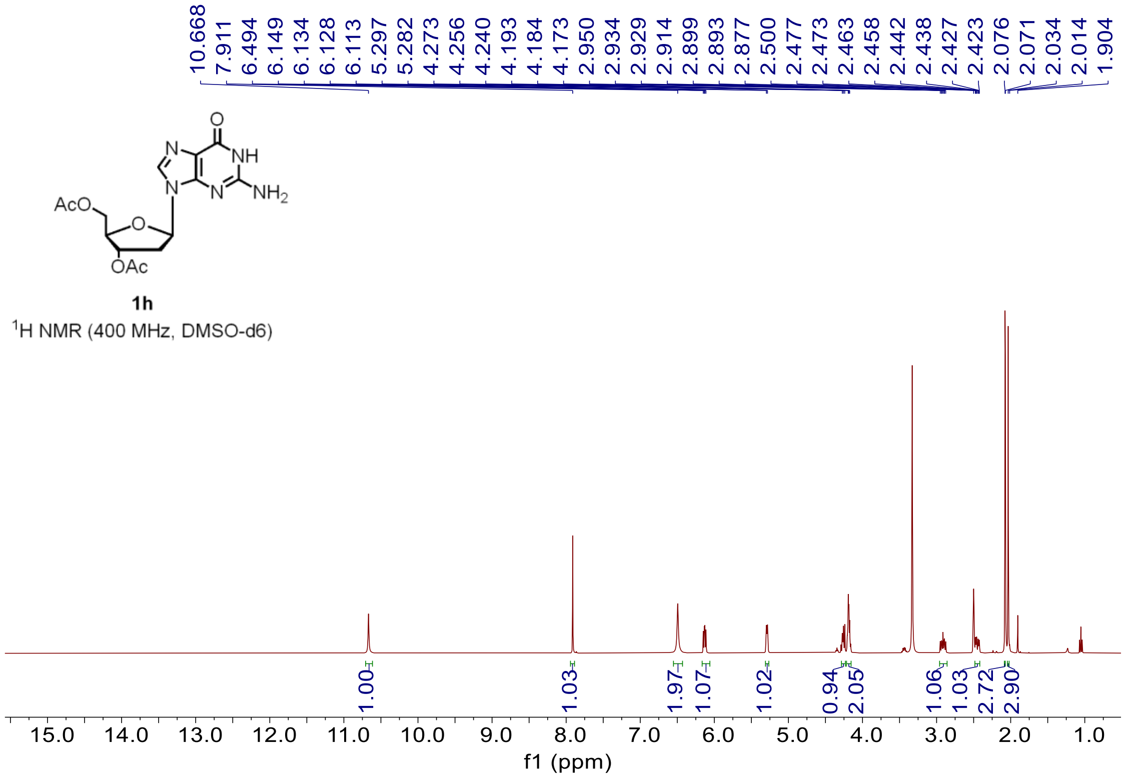

Supplementary Figure 110. ^{1}H NMR spectra of compound **1h**

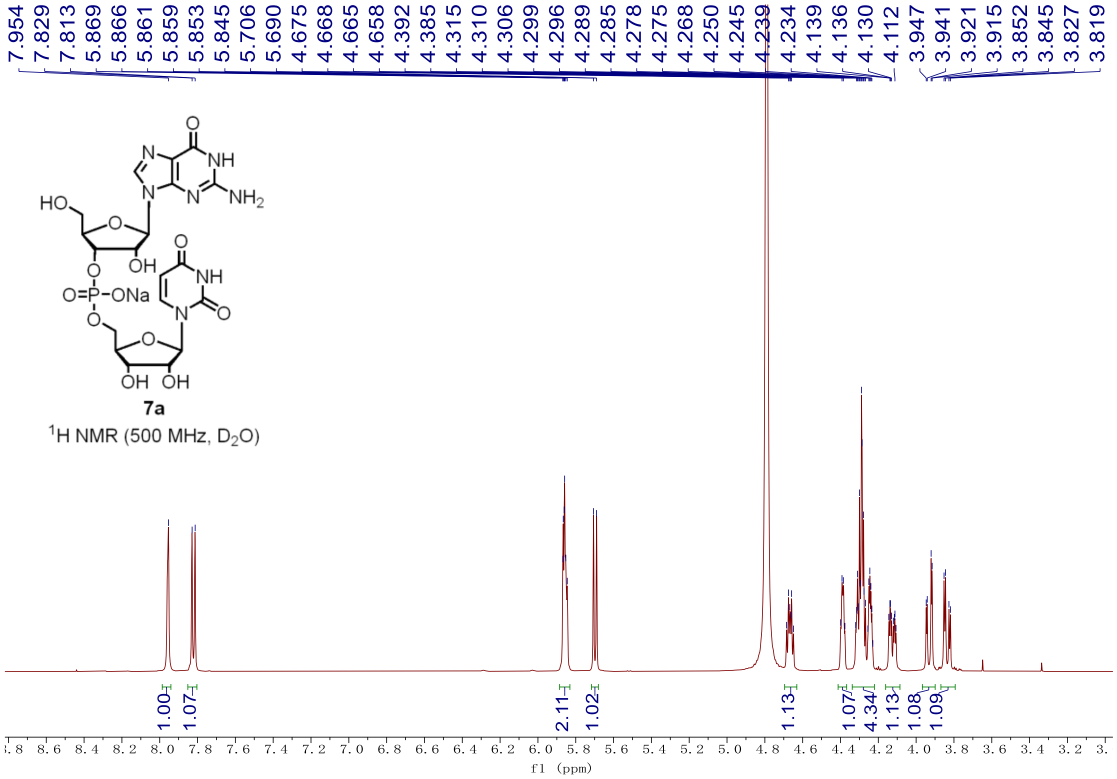

Supplementary Figure 111. ^{1}H NMR spectra of compound **7a**

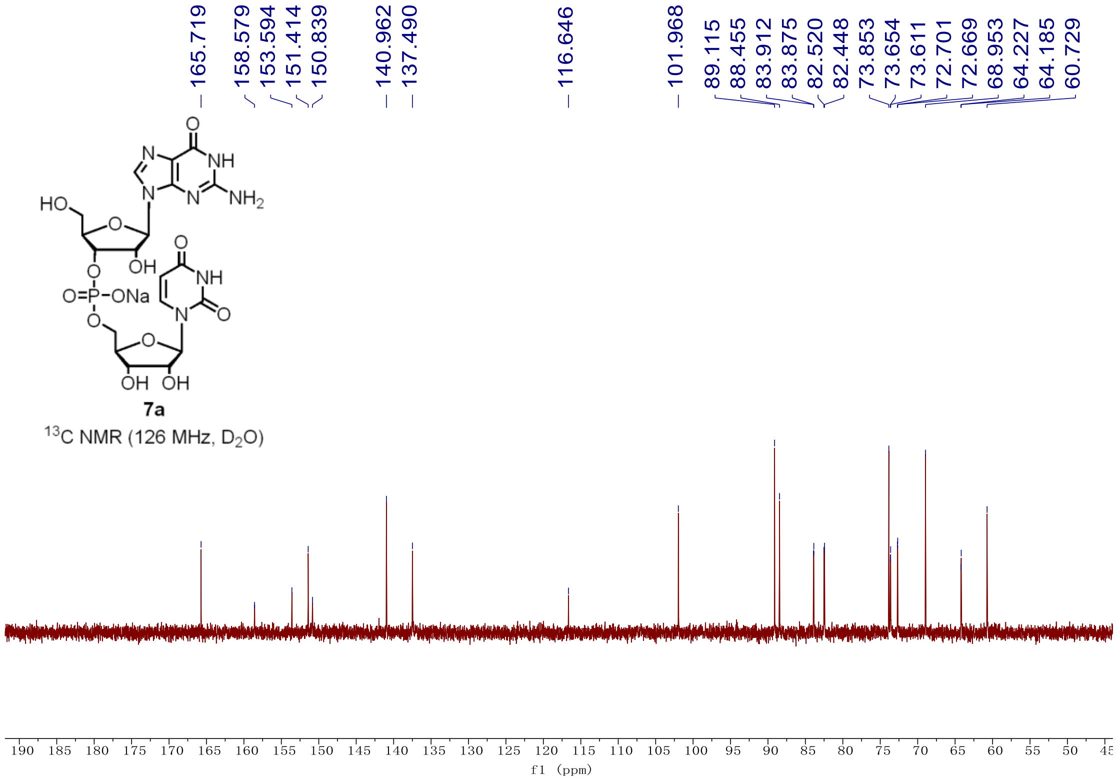

Supplementary Figure 112. ^{13}C NMR spectra of compound **7a**

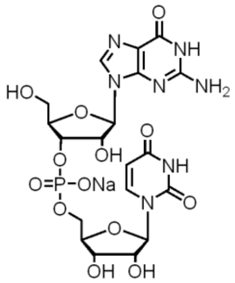

7a

^{31}P NMR (202 MHz, D_2O)

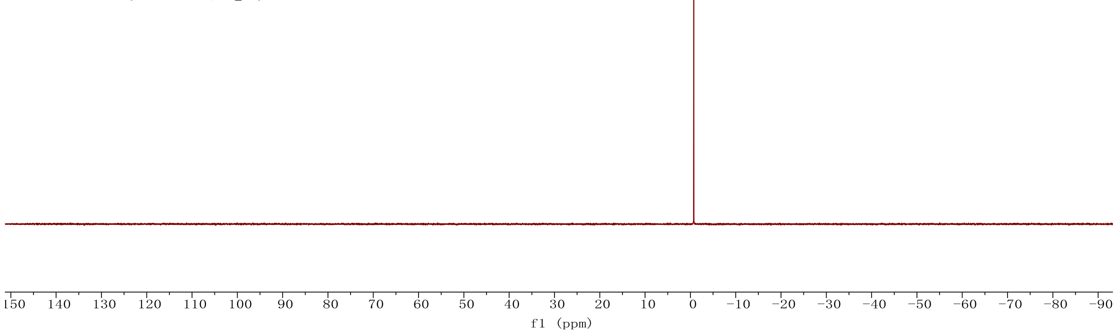

Supplementary Figure 113. ^{31}P NMR spectra of compound **7a**

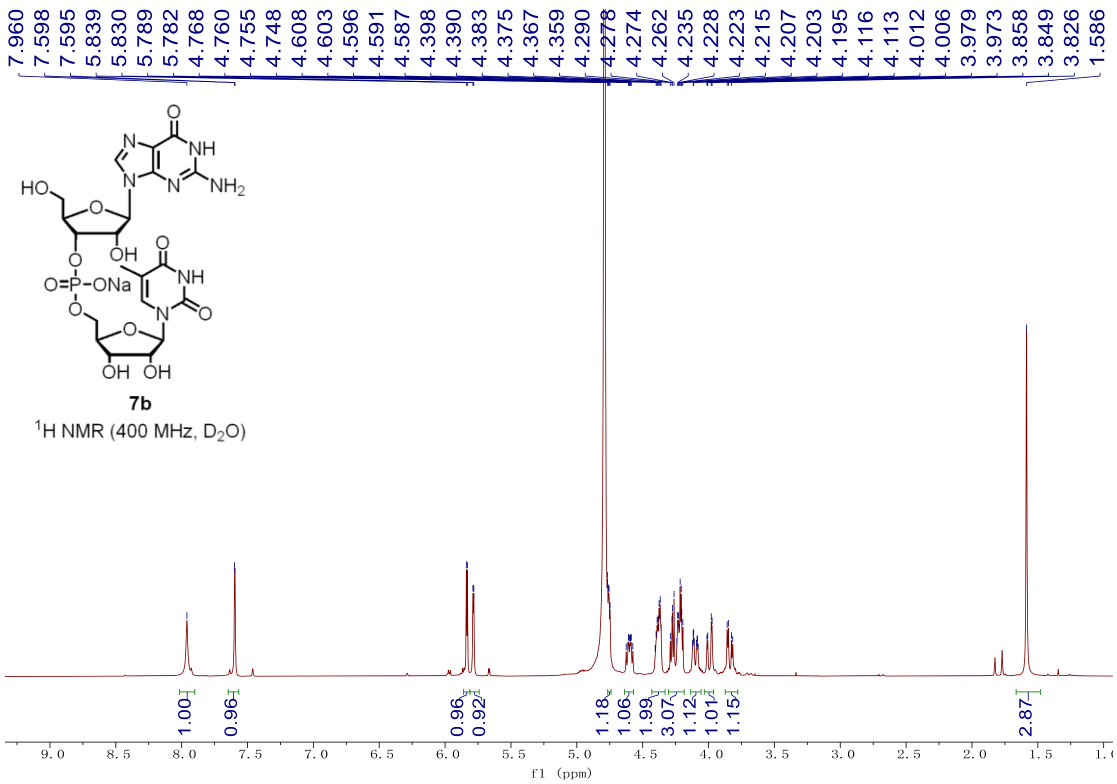

Supplementary Figure 114. ^{1}H NMR spectra of compound **7b**

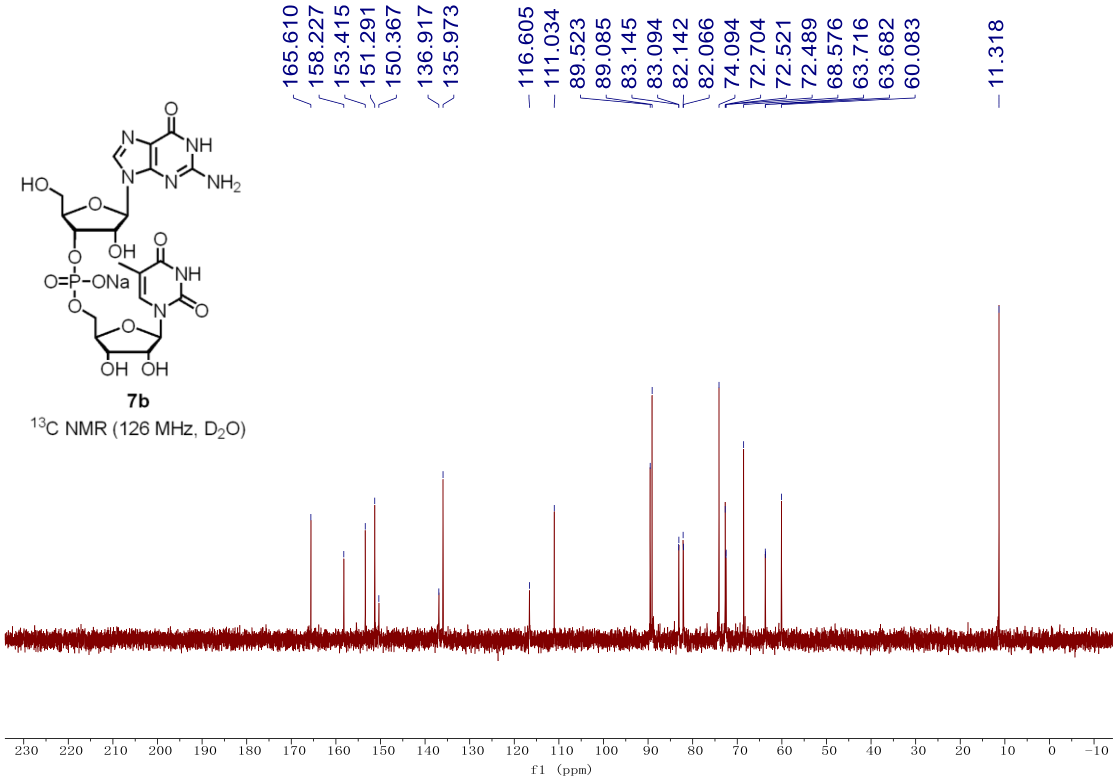

Supplementary Figure 115. ^{13}C NMR spectra of compound **7b**

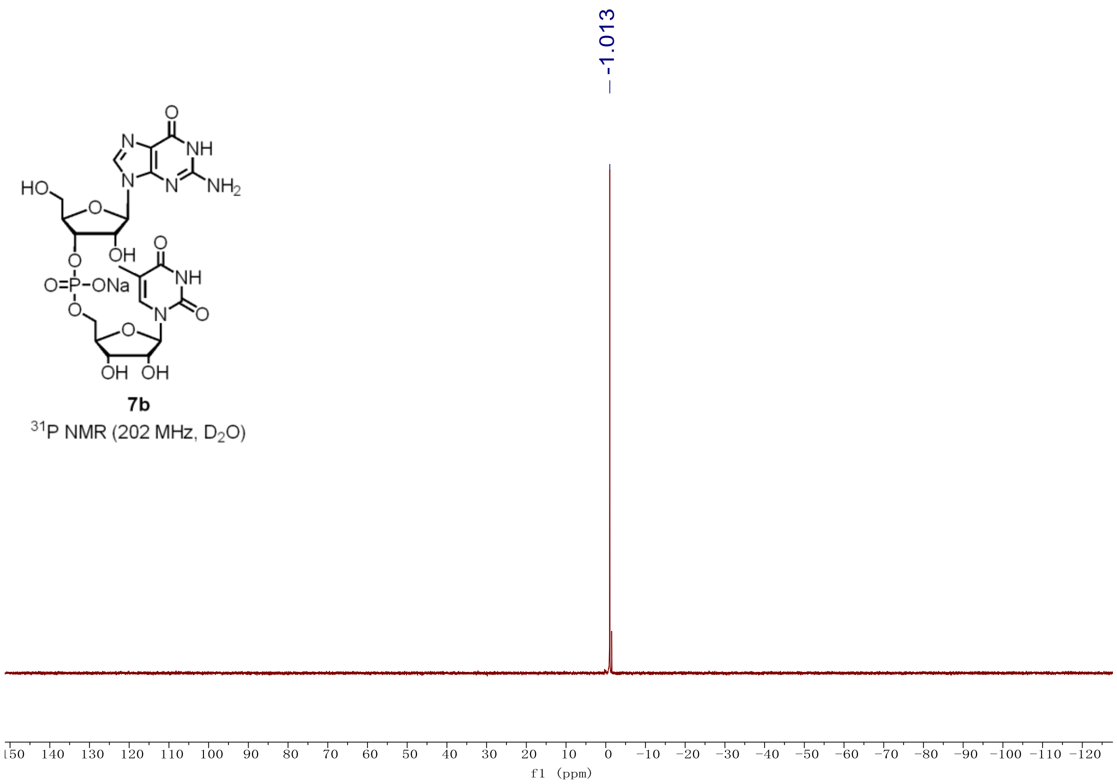

Supplementary Figure 116. ^{31}P NMR spectra of compound **7b**

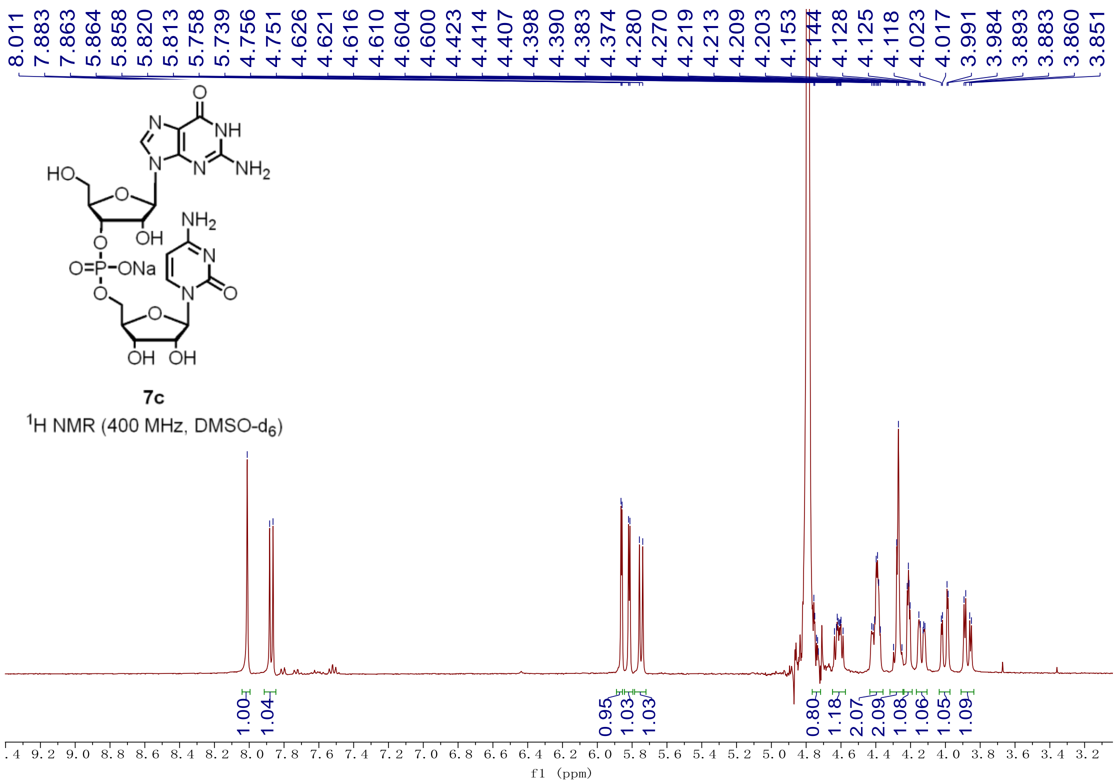

Supplementary Figure 117. ^{1}H NMR spectra of compound **7c**

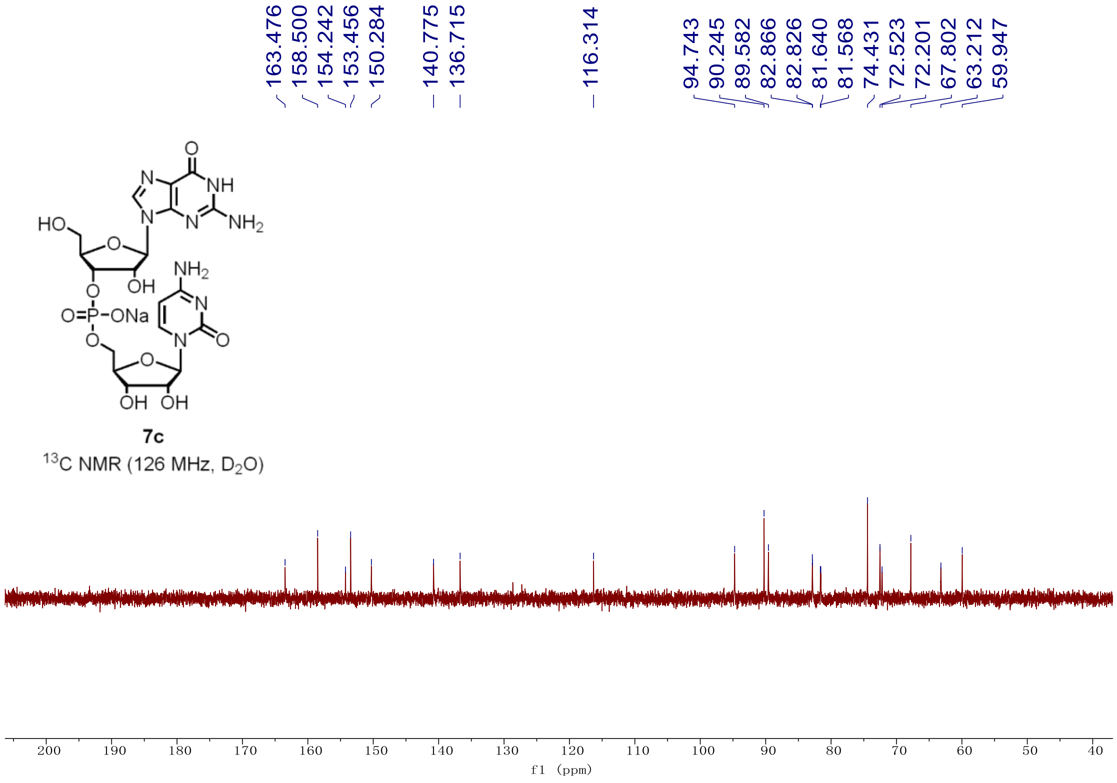

Supplementary Figure 118. ^{13}C NMR spectra of compound **7c**

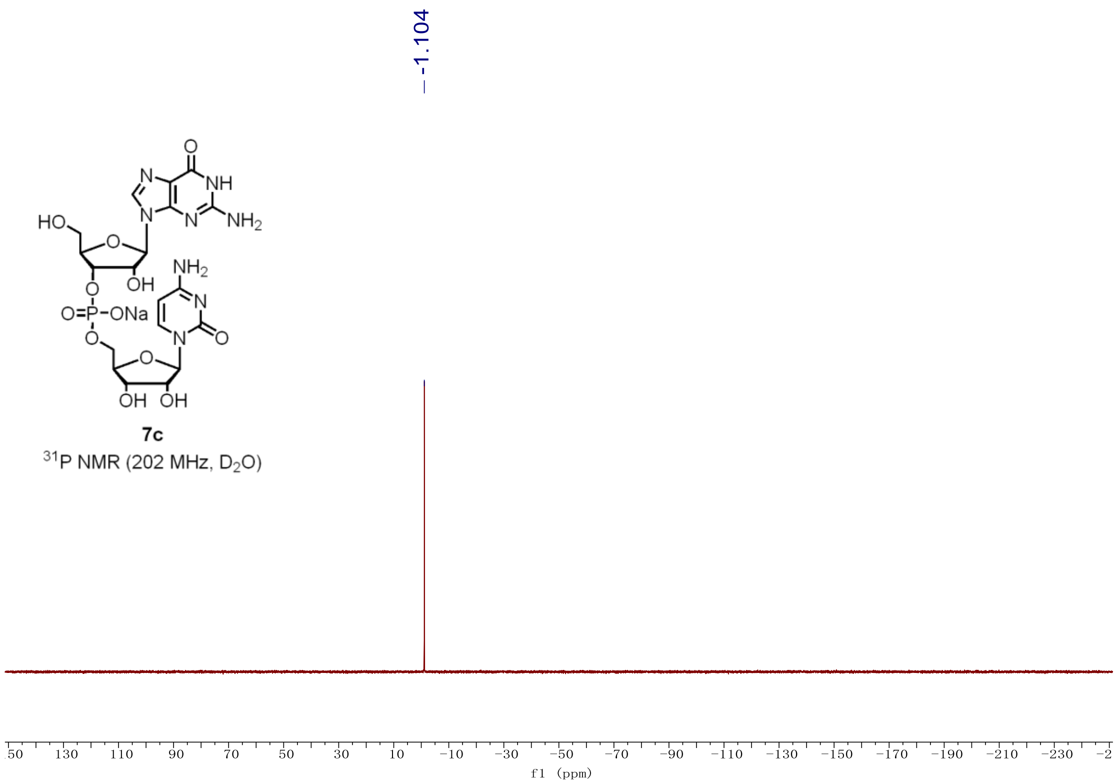

Supplementary Figure 119. ^{31}P NMR spectra of compound **7c**

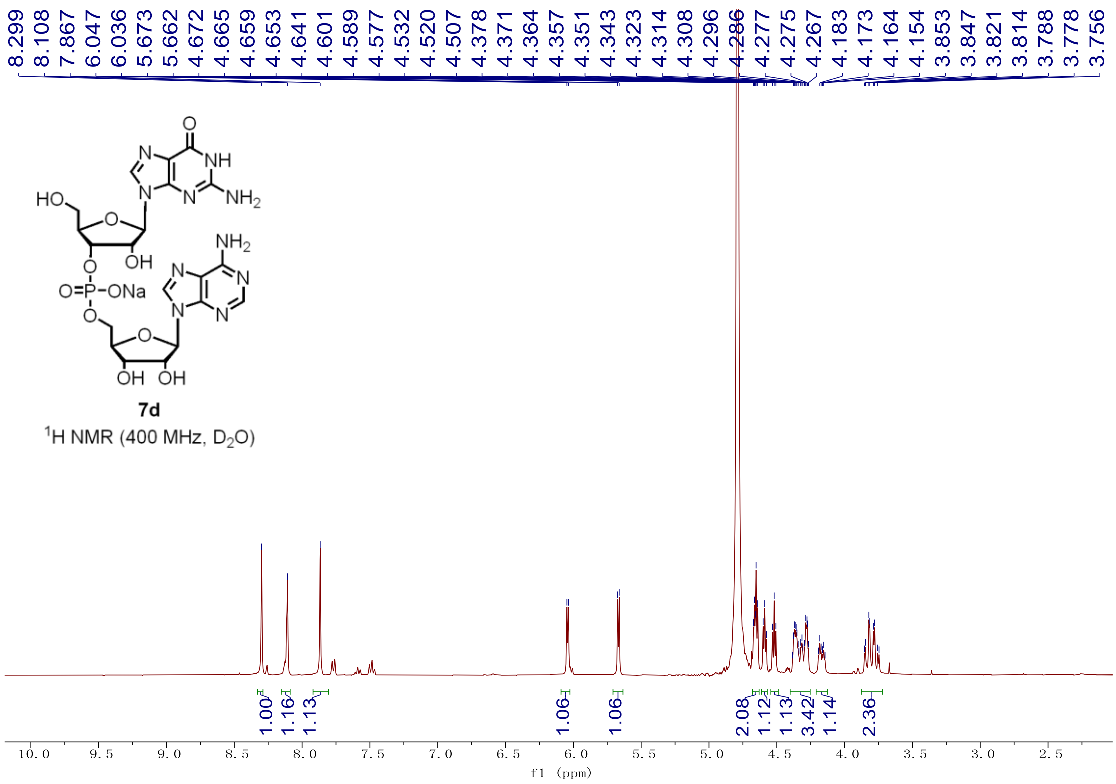

Supplementary Figure 120. ^{1}H NMR spectra of compound **7d**

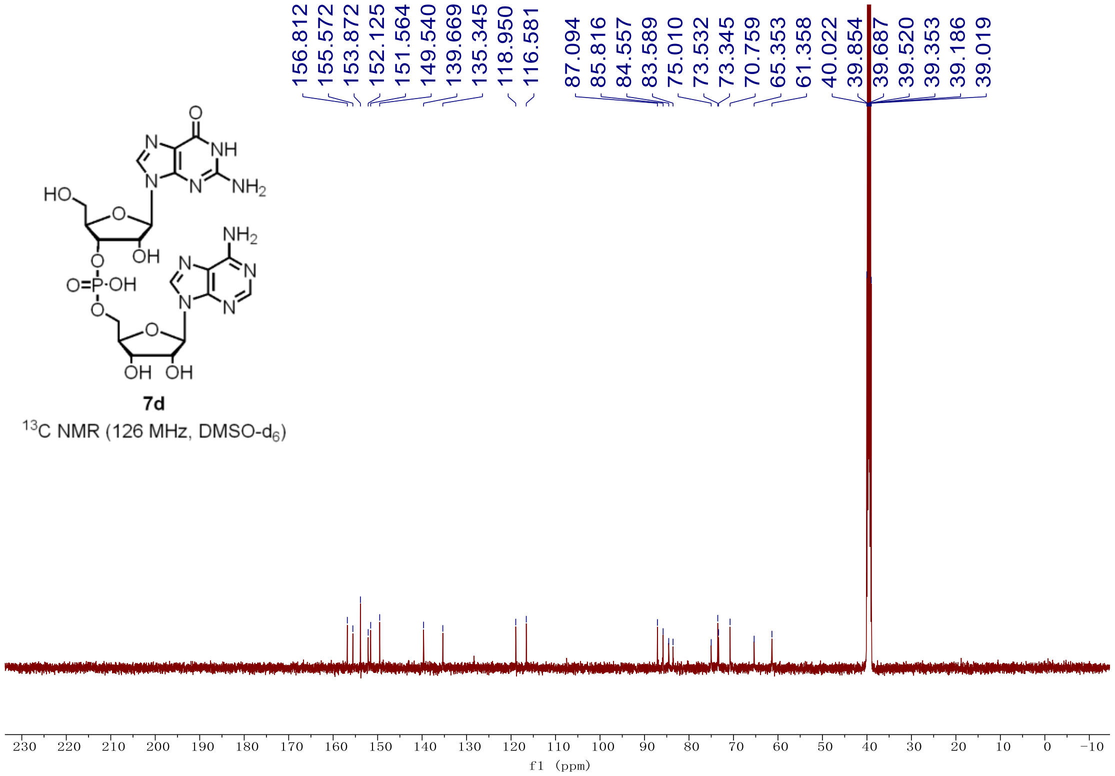

Supplementary Figure 121. ^{13}C NMR spectra of compound **7d**

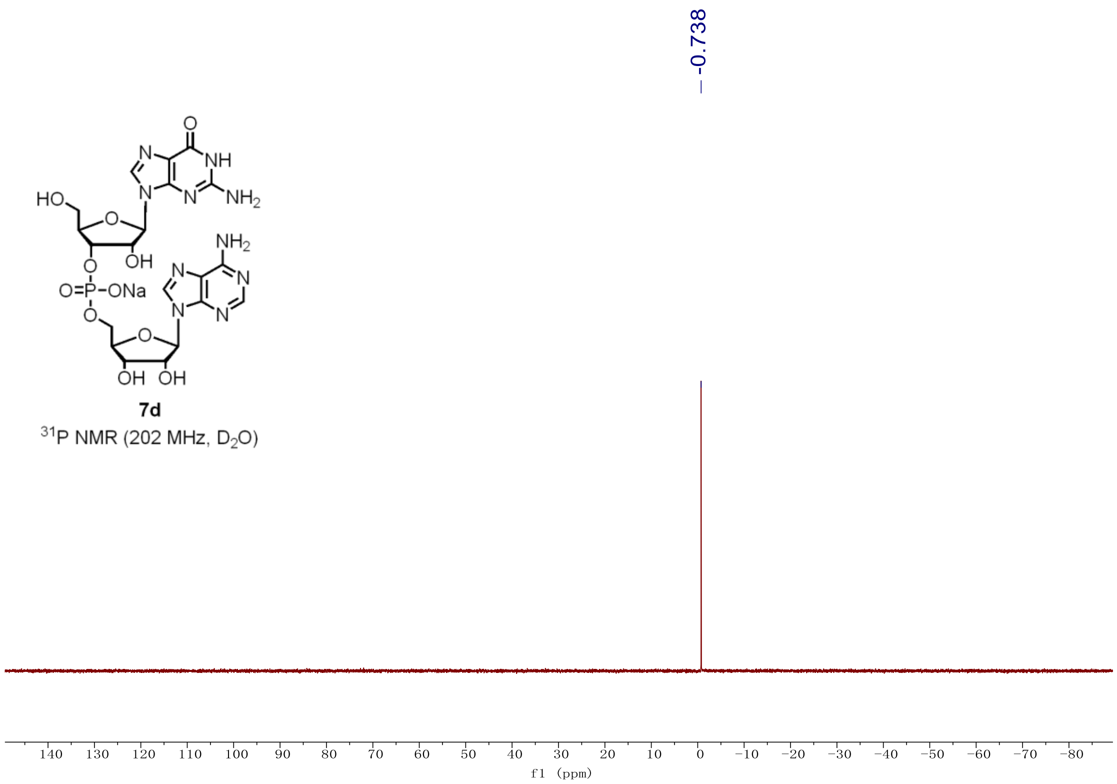

Supplementary Figure 122. ^{31}P NMR spectra of compound **7d**

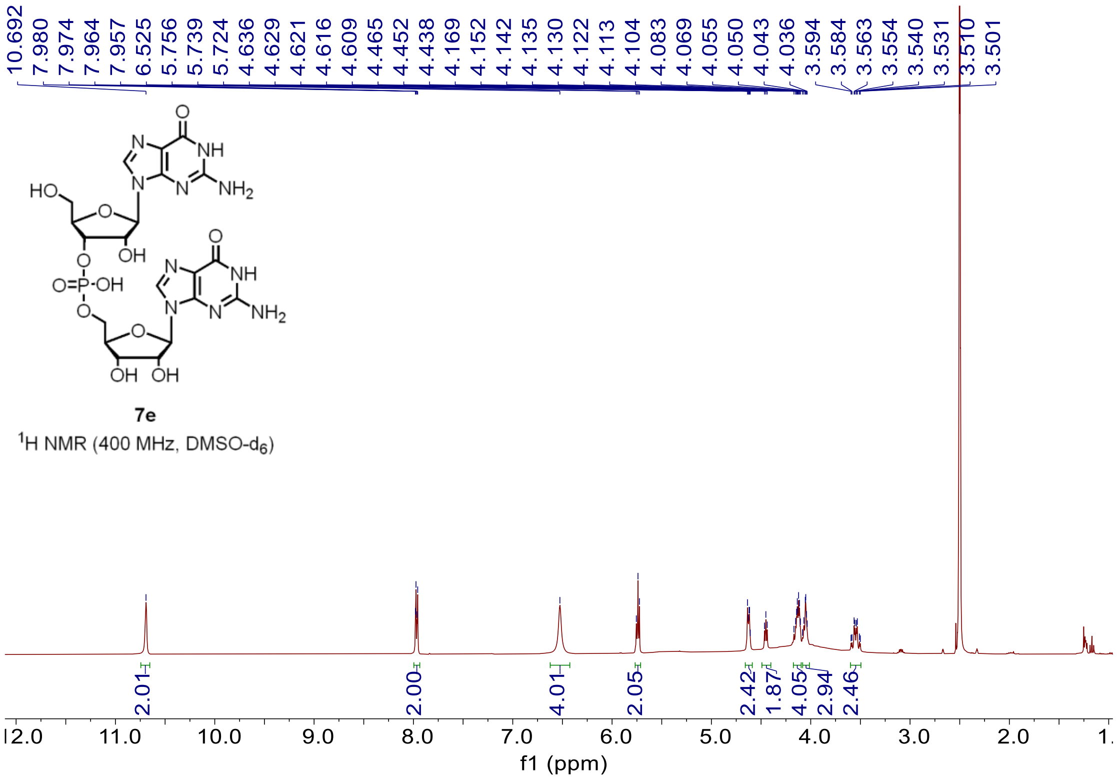

Supplementary Figure 123. ^{1}H NMR spectra of compound **7e**

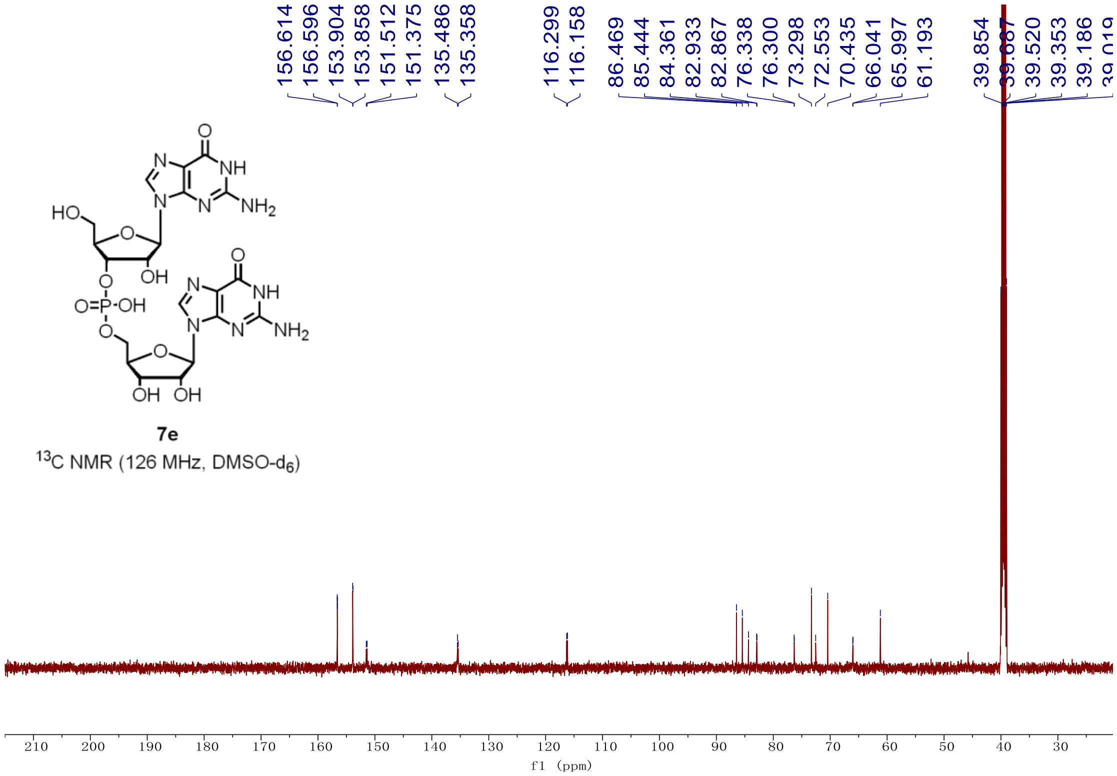

Supplementary Figure 124. ^{13}C NMR spectra of compound **7e**

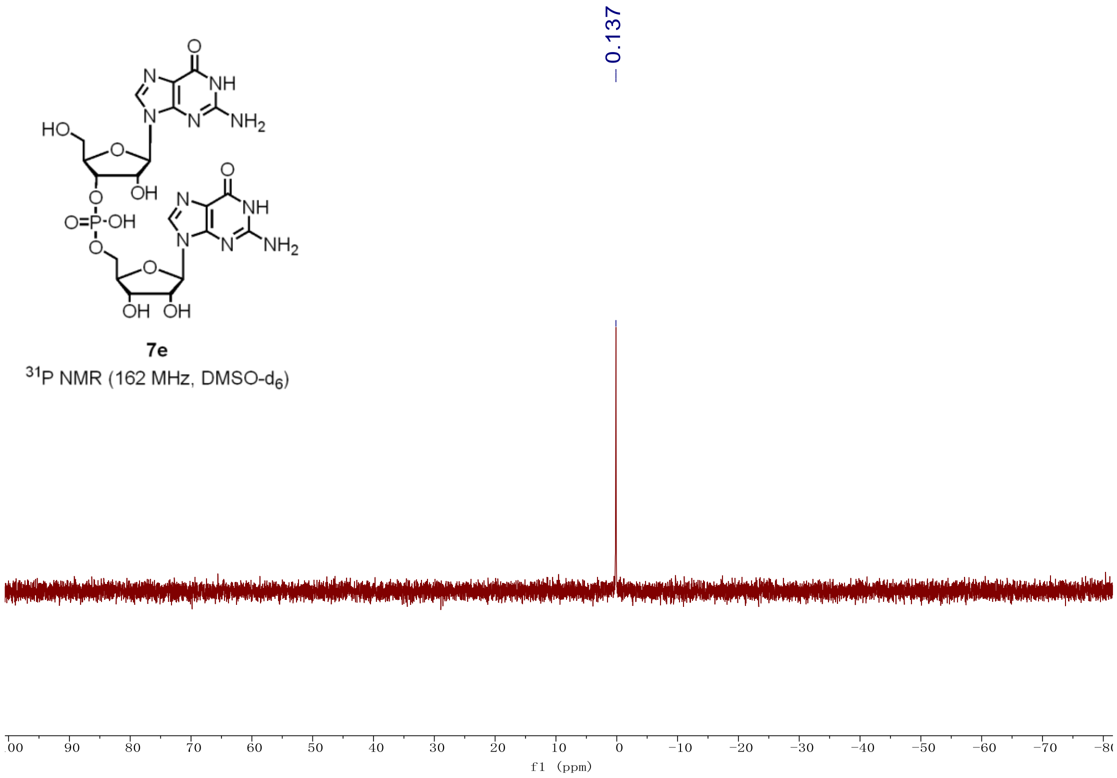

Supplementary Figure 125. ^{31}P NMR spectra of compound **7e**

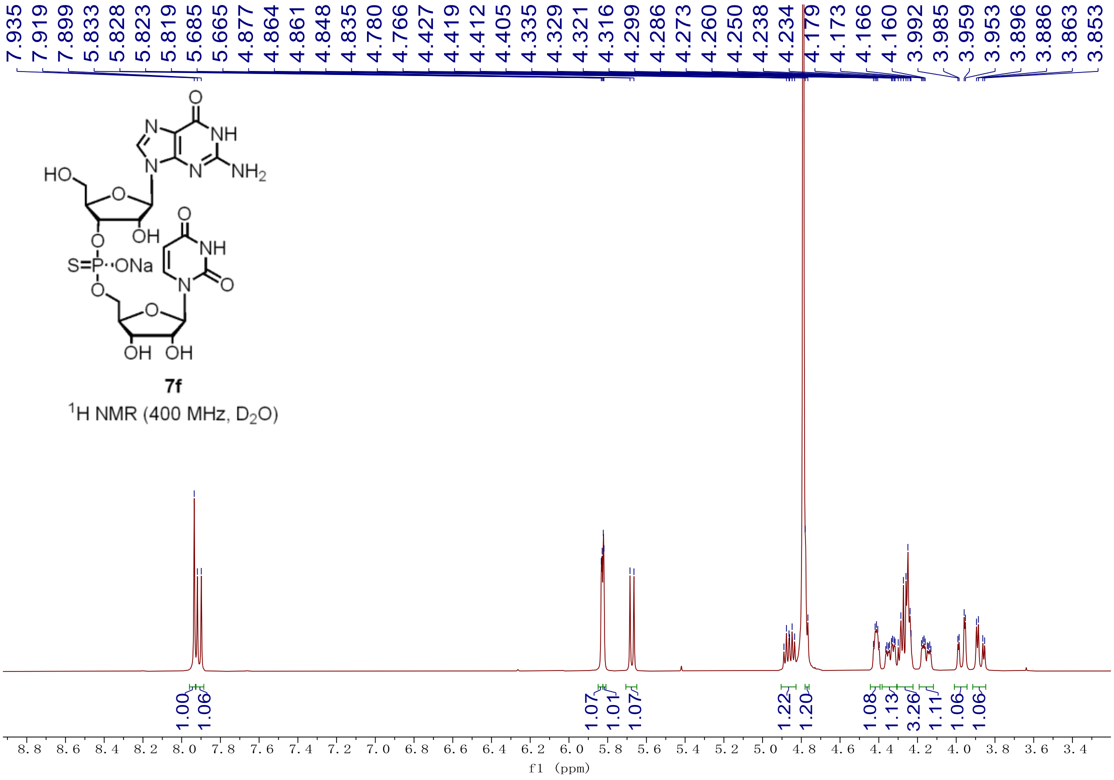

Supplementary Figure 126. ^{1}H NMR spectra of compound **7f**

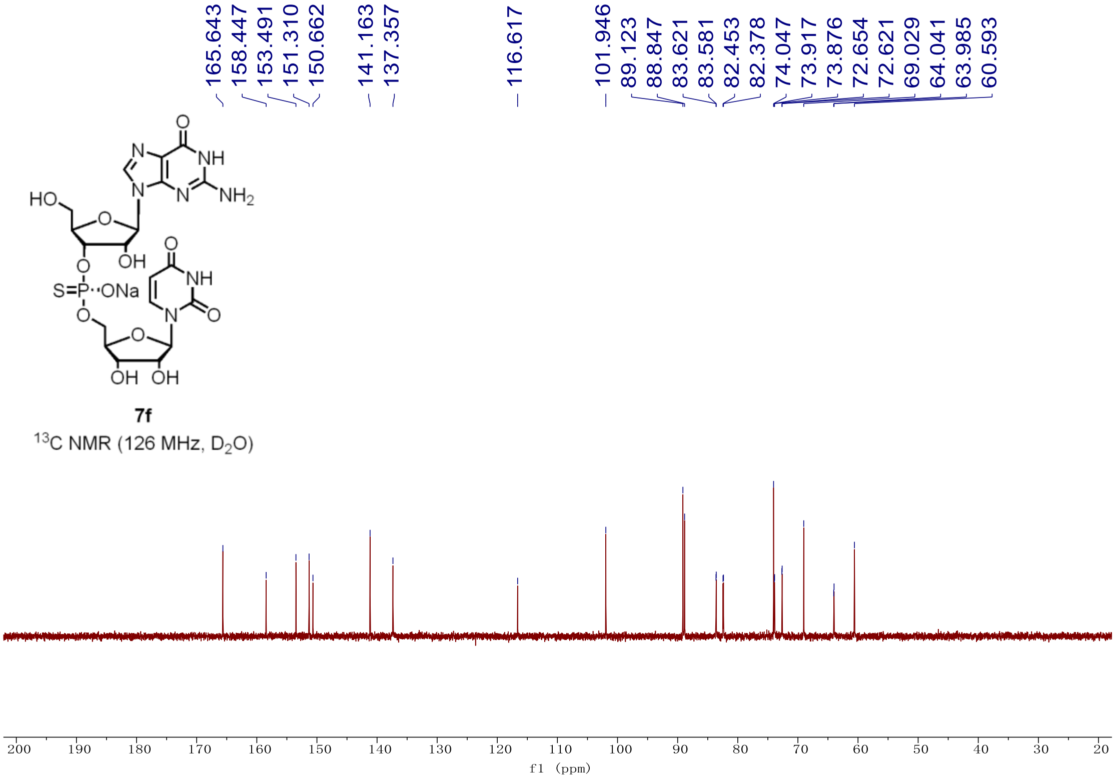

Supplementary Figure 127. ^{13}C NMR spectra of compound **7f**

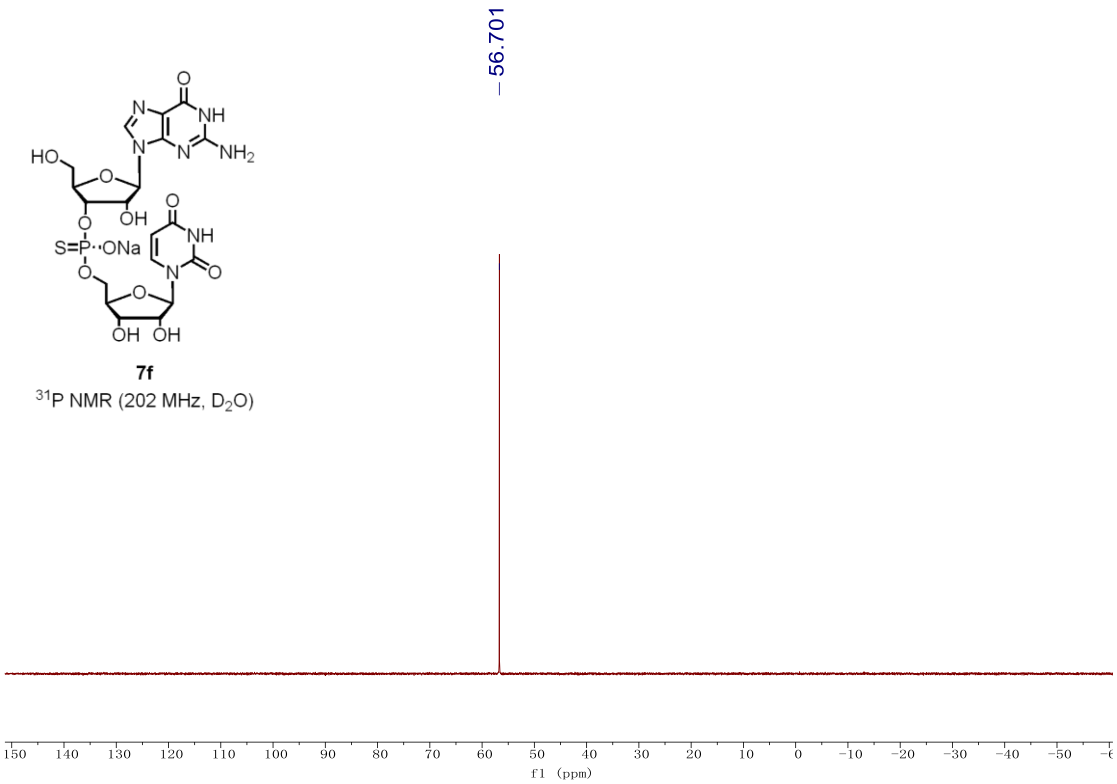

Supplementary Figure 128. ^{31}P NMR spectra of compound **7f**

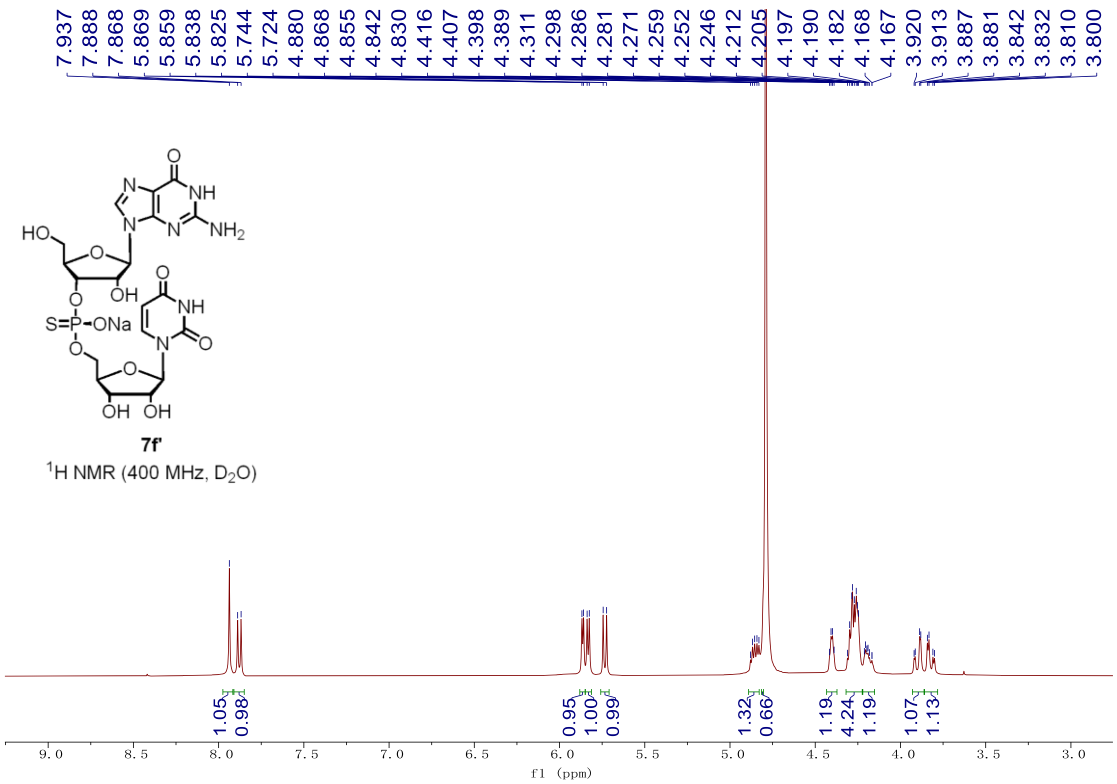

Supplementary Figure 129. ^{1}H NMR spectra of compound **7f'**

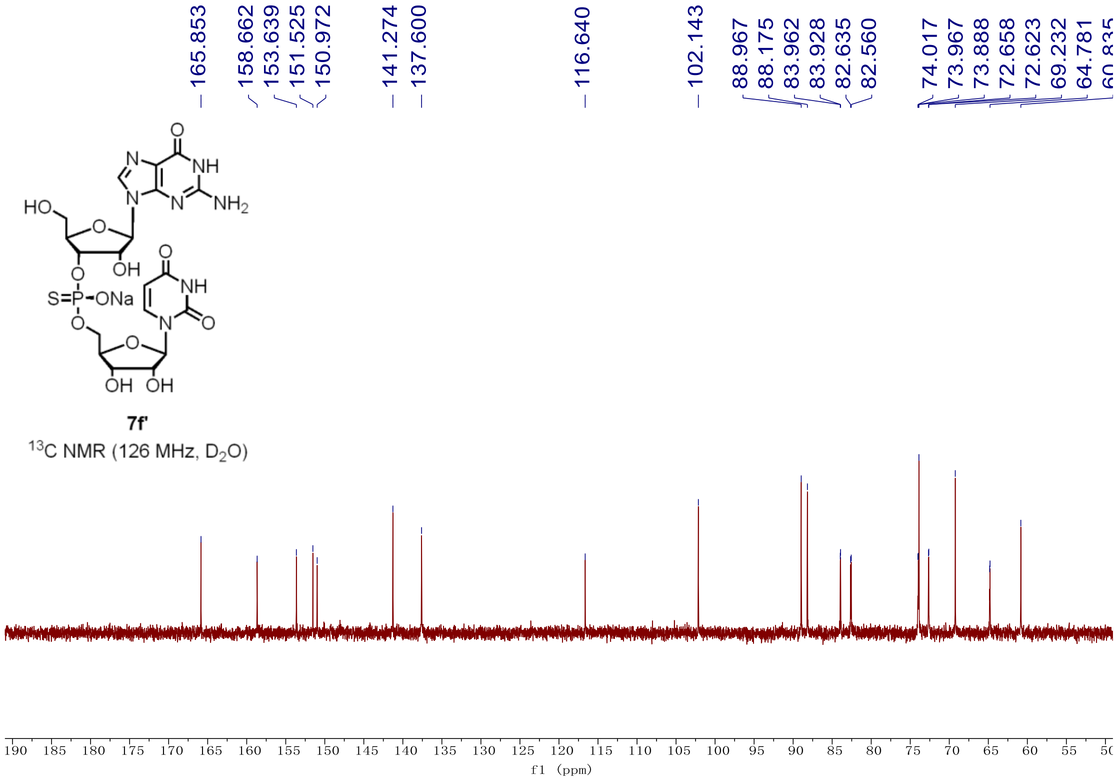

Supplementary Figure 130. ^{13}C NMR spectra of compound **7f'**

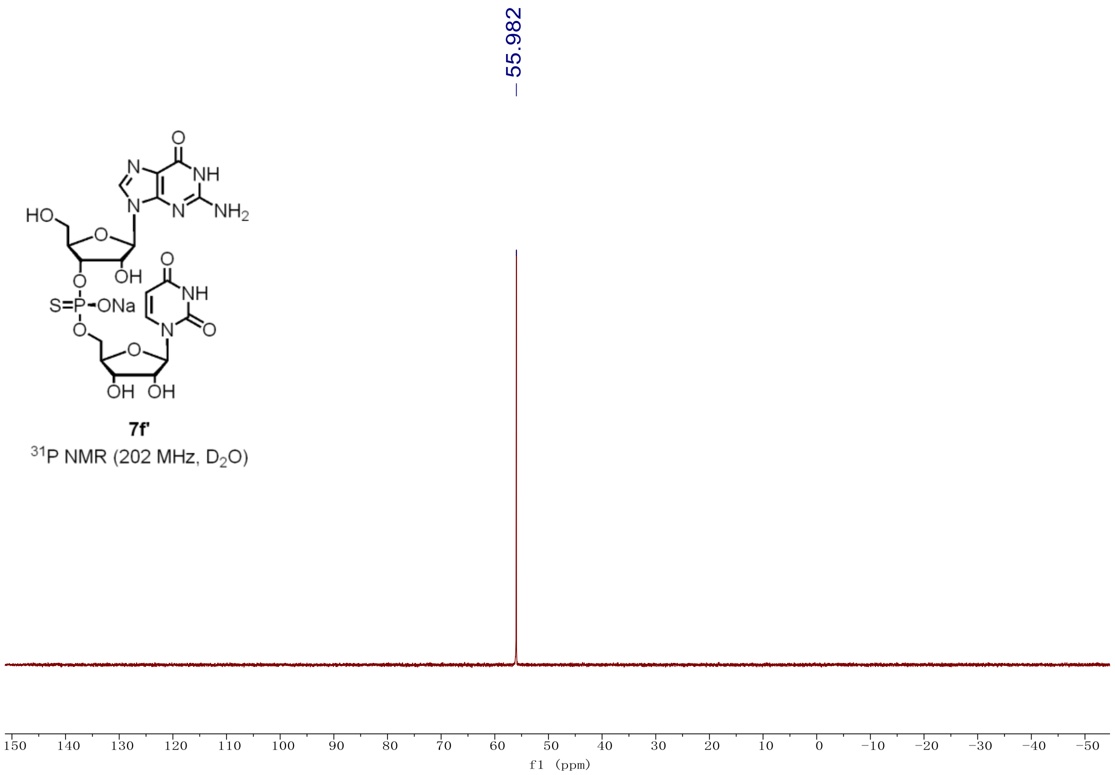

Supplementary Figure 131. ^{31}P NMR spectra of compound **7f'**

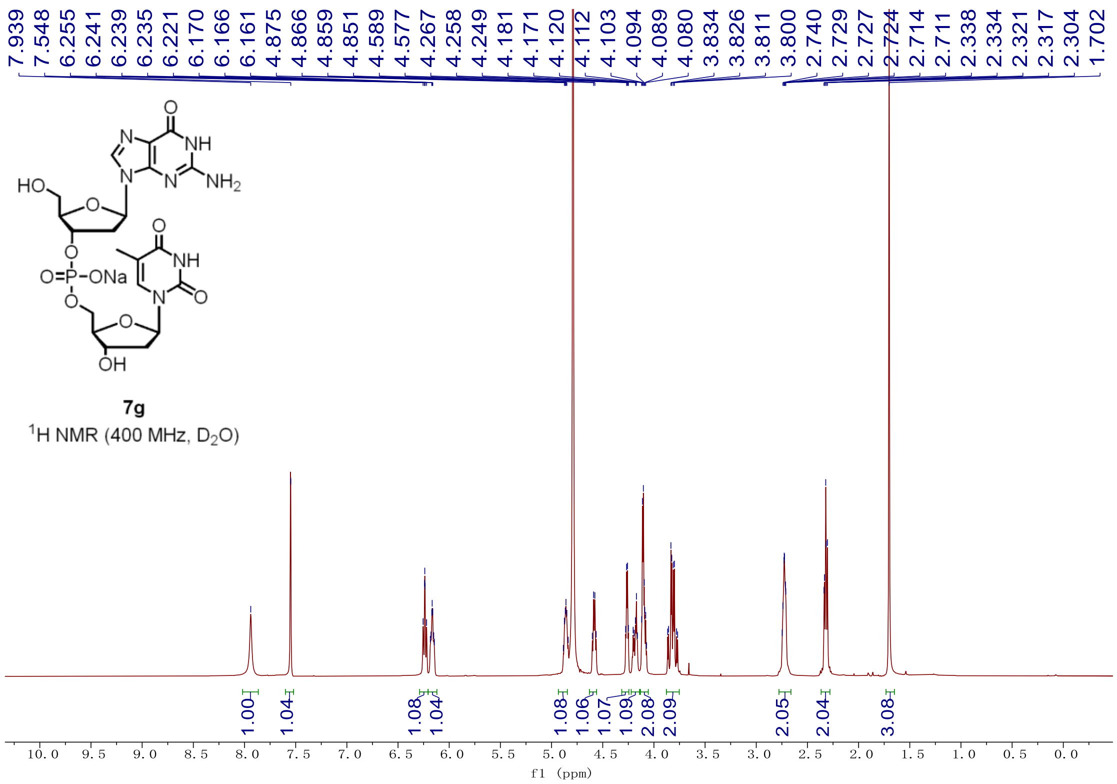

Supplementary Figure 132. ^{1}H NMR spectra of compound **7g**

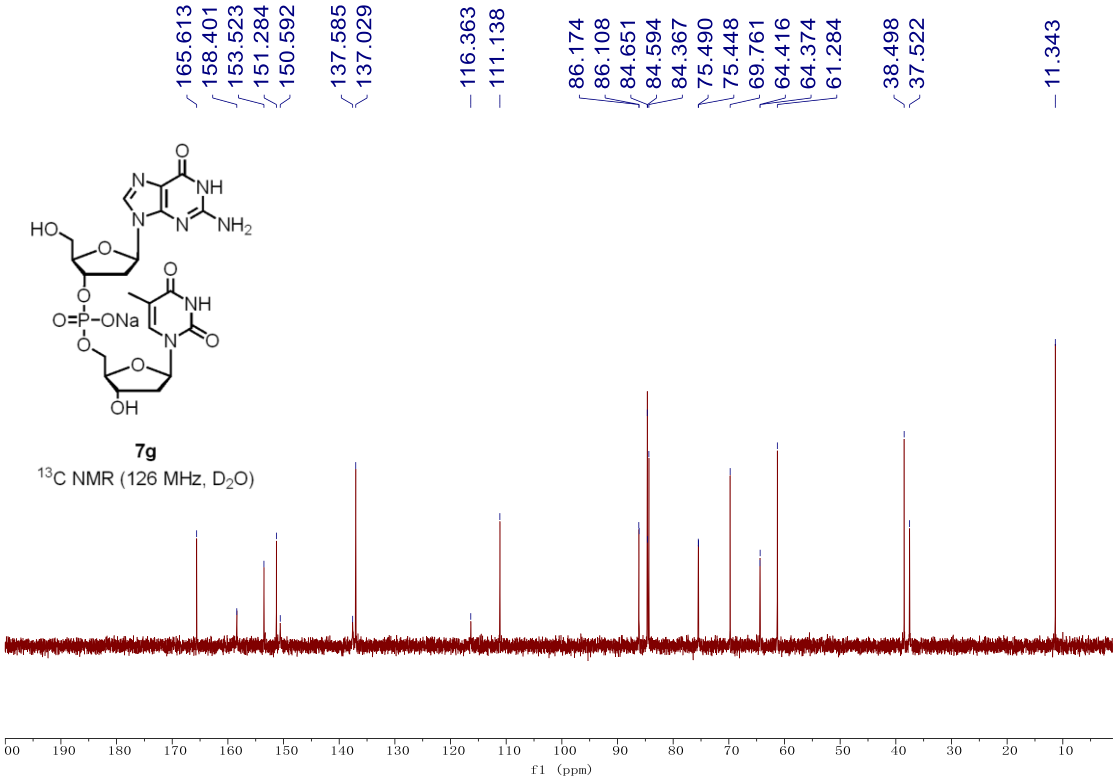

Supplementary Figure 133. ^{13}C NMR spectra of compound **7g**

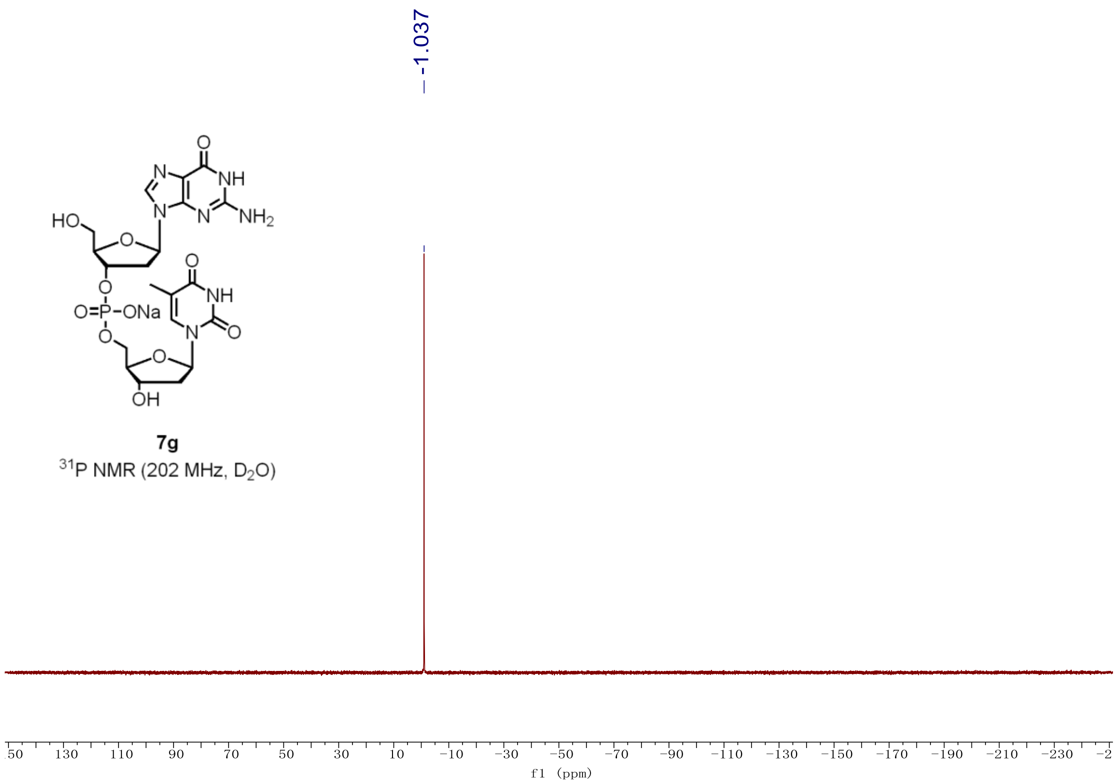

Supplementary Figure 134. ^{31}P NMR spectra of compound **7g**

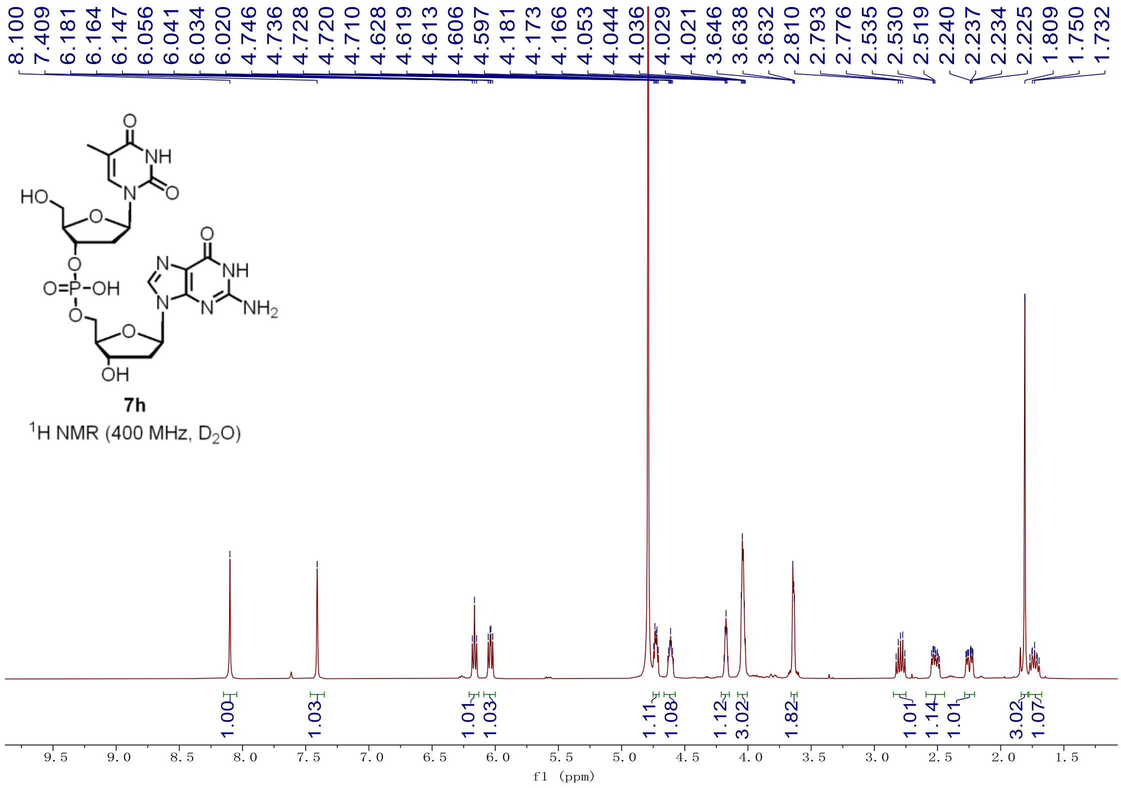

Supplementary Figure 135. ^{1}H NMR spectra of compound **7h**

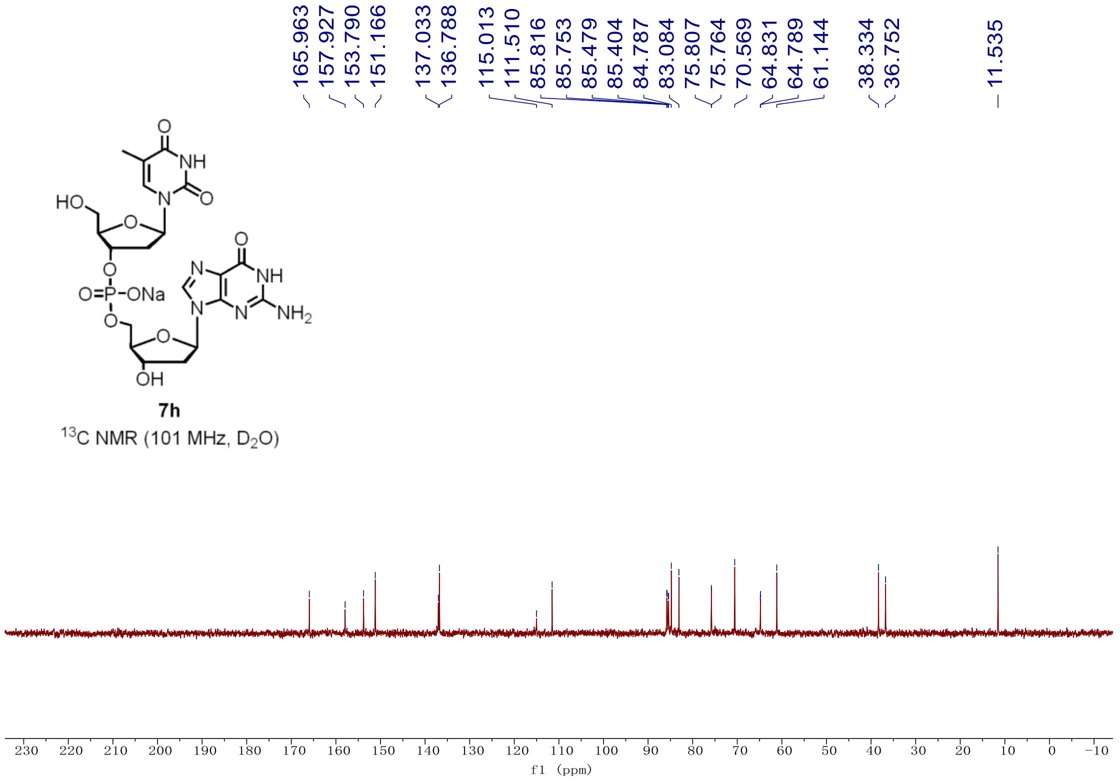

Supplementary Figure 136. ^{13}C NMR spectra of compound **7h**

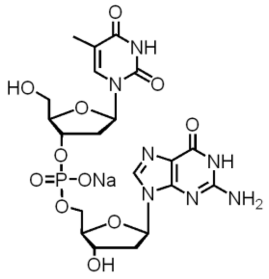

7h

^{31}P NMR (202 MHz, D_2O)

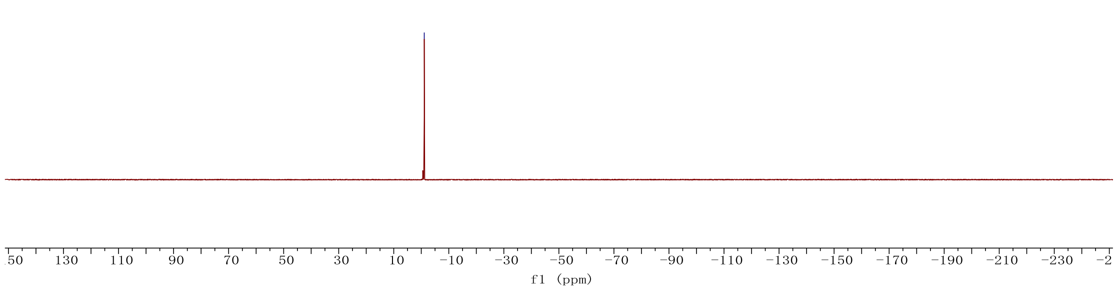

Supplementary Figure 137. ^{31}P NMR spectra of compound **7h**

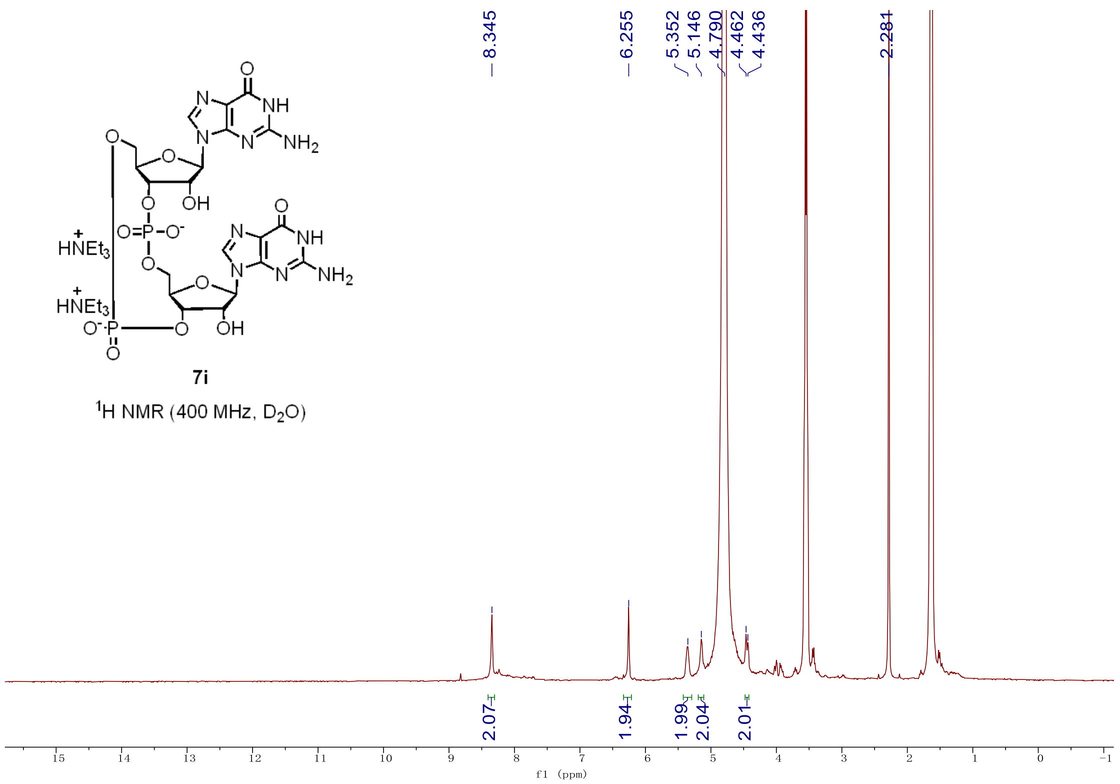

Supplementary Figure 138. ^{1}H NMR spectra of compound **7i**

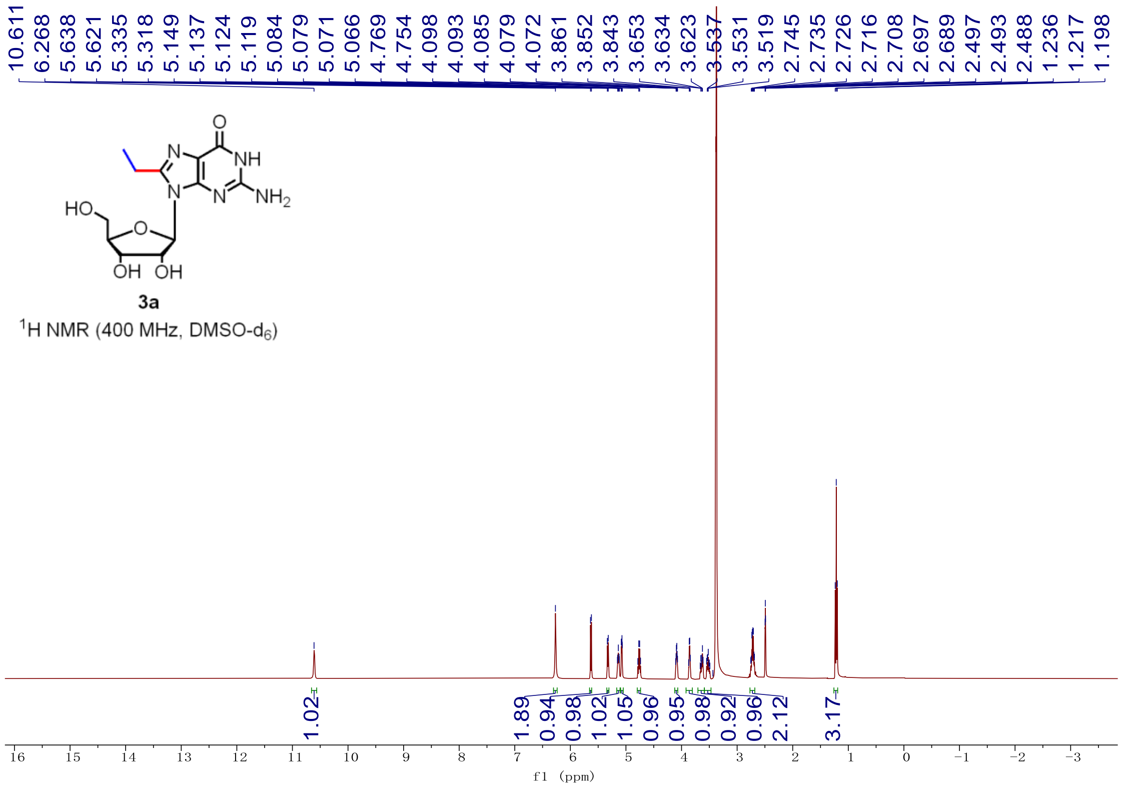

Supplementary Figure 139. ^{1}H NMR spectra of compound **3a**

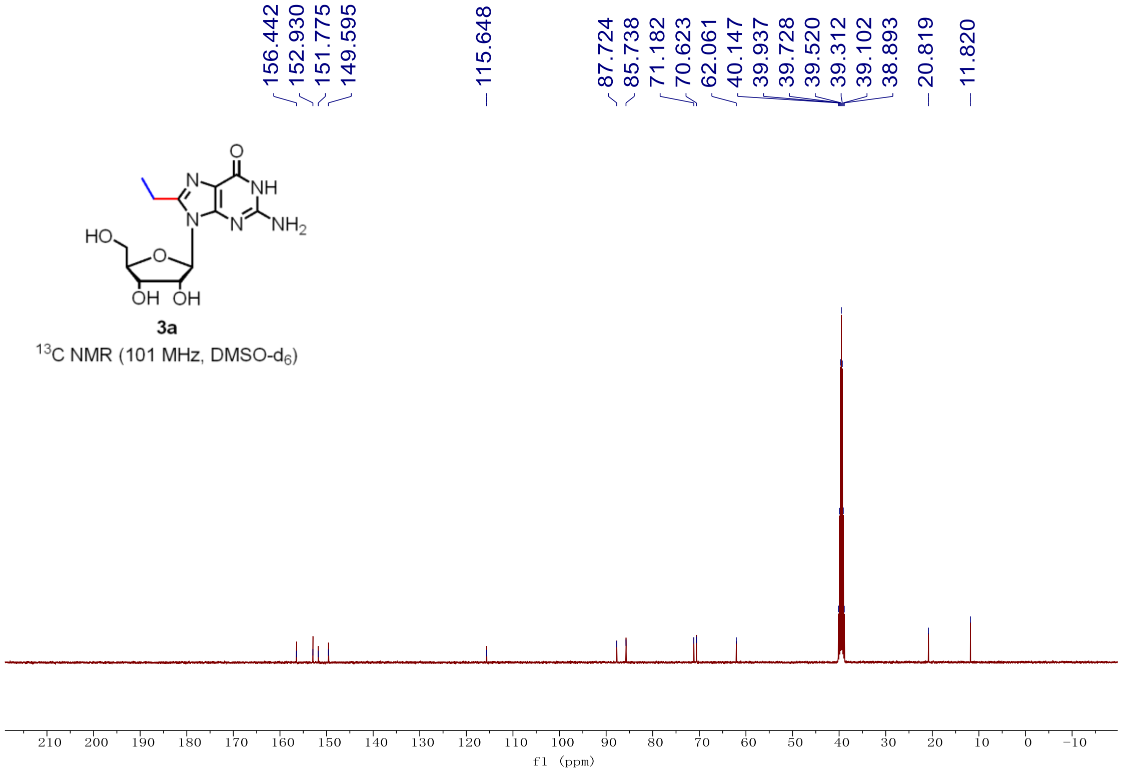

Supplementary Figure 140. ^{13}C NMR spectra of compound **3a**

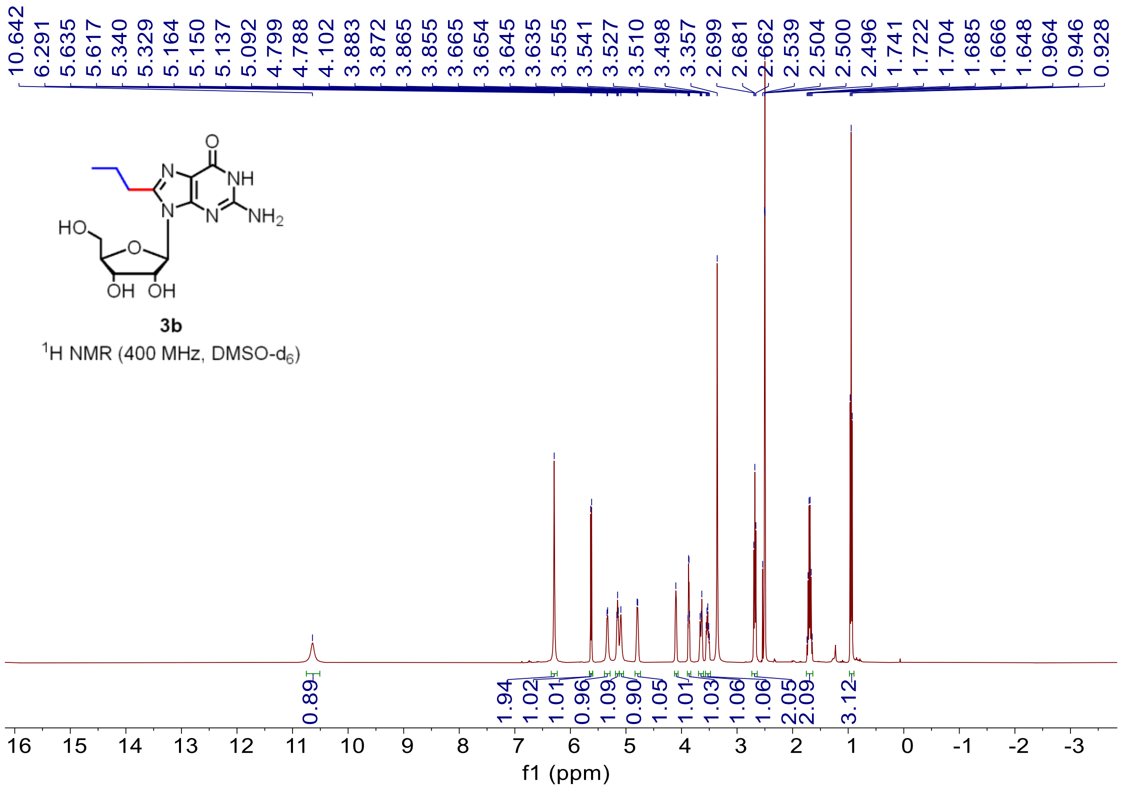

Supplementary Figure 141. ^{1}H NMR spectra of compound **3b**

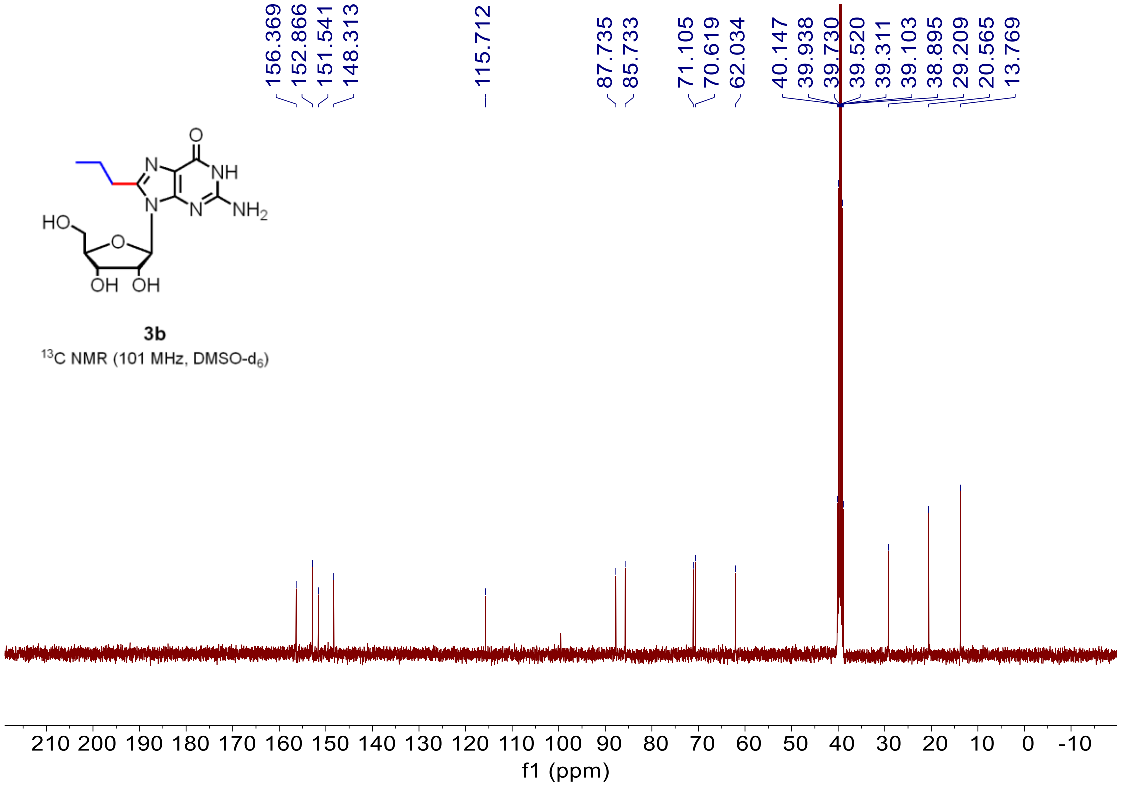

Supplementary Figure 142. ^{13}C NMR spectra of compound **3b**

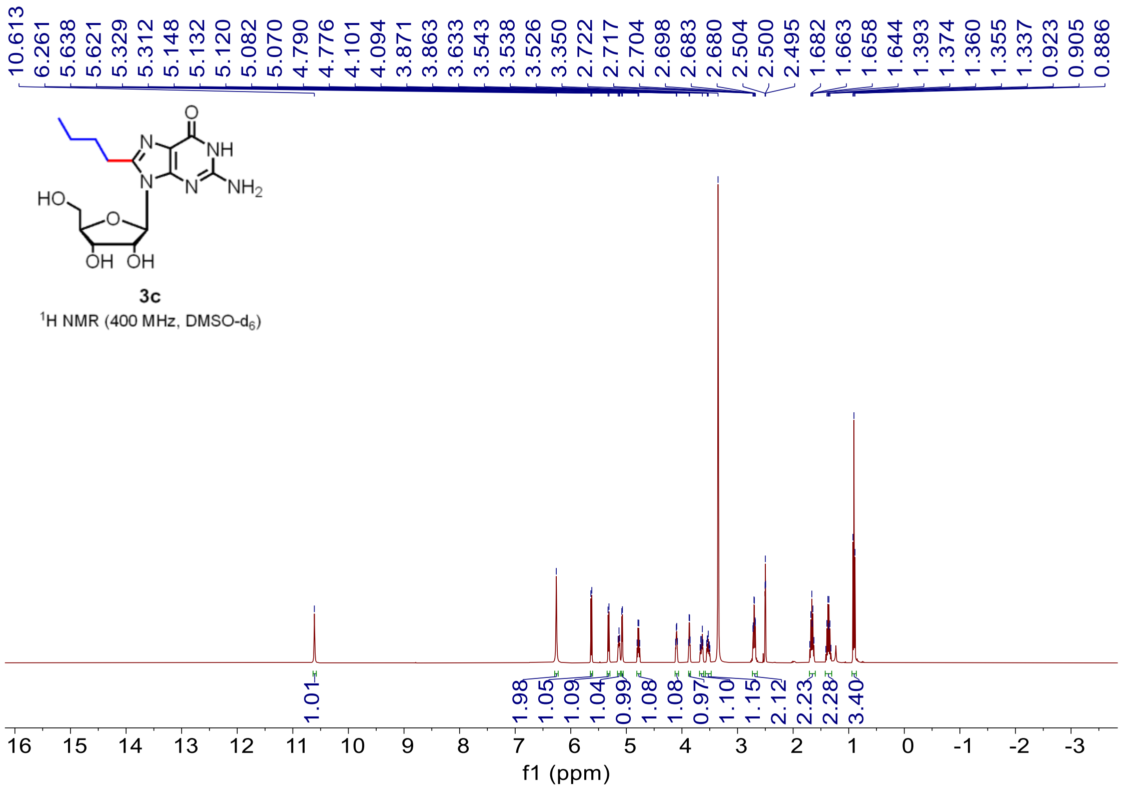

Supplementary Figure 143. ^{1}H NMR spectra of compound **3c**

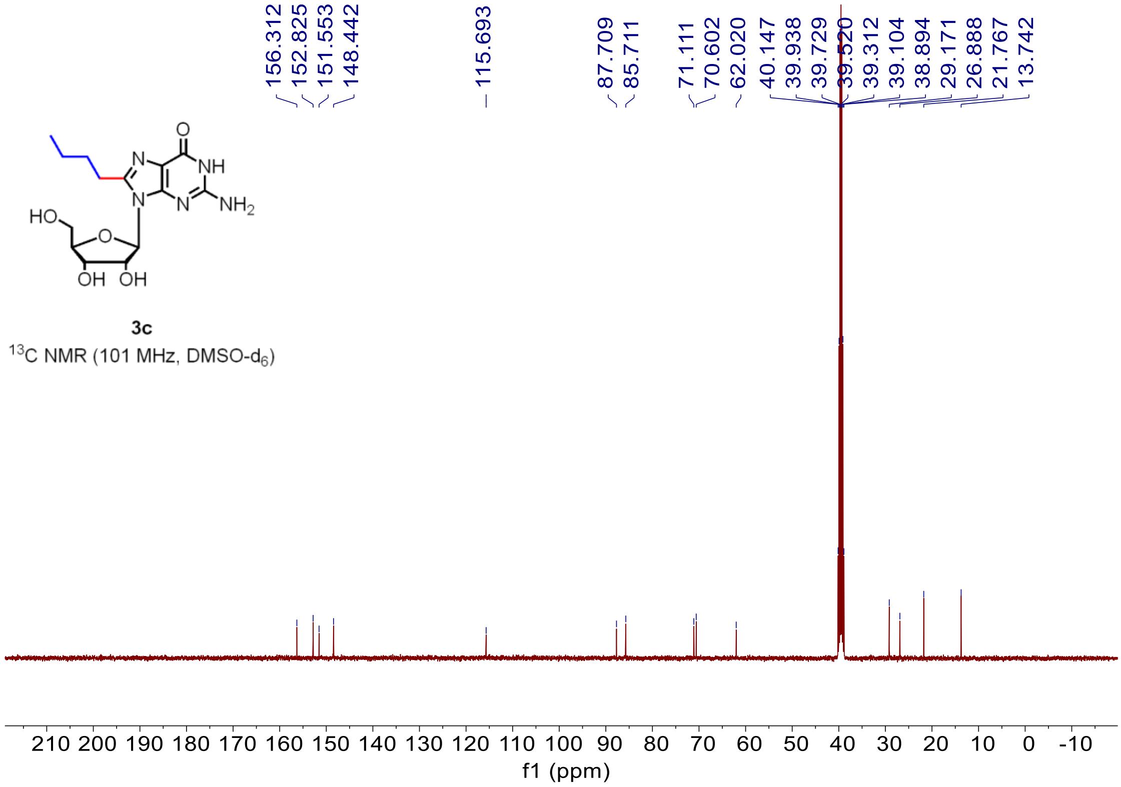

Supplementary Figure 144. ^{13}C NMR spectra of compound **3c**

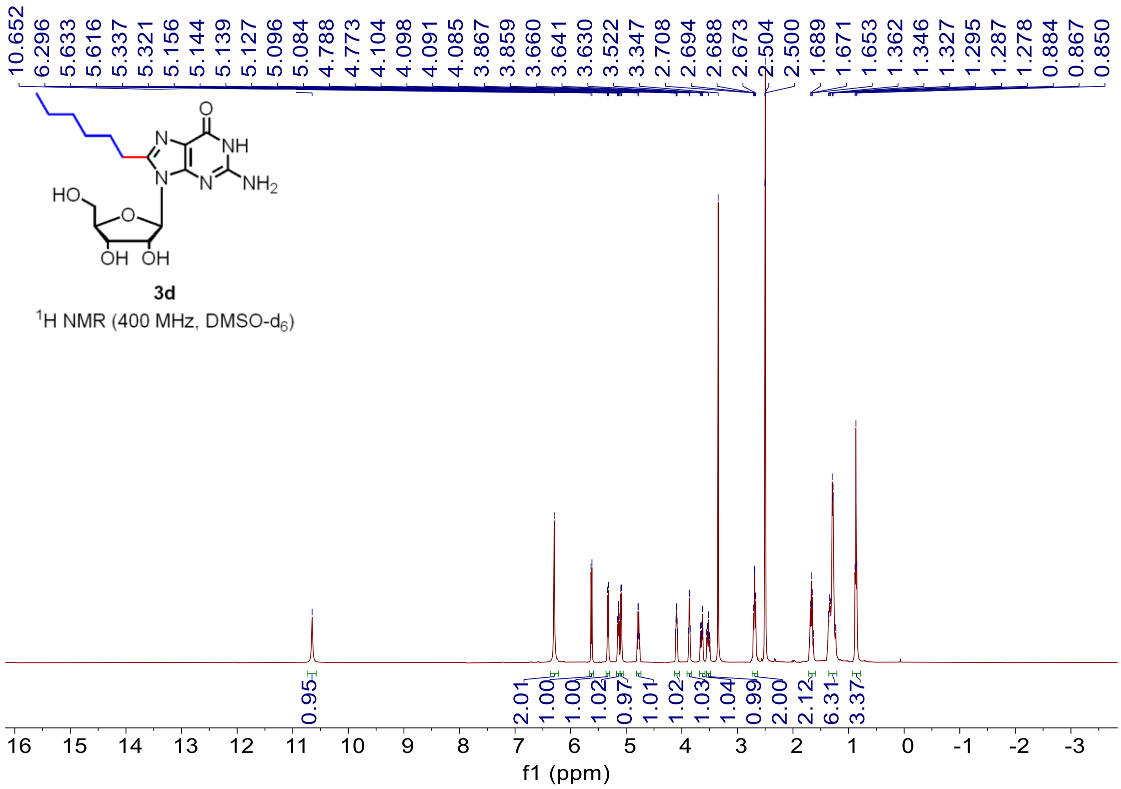

Supplementary Figure 145. ^{1}H NMR spectra of compound **3d**

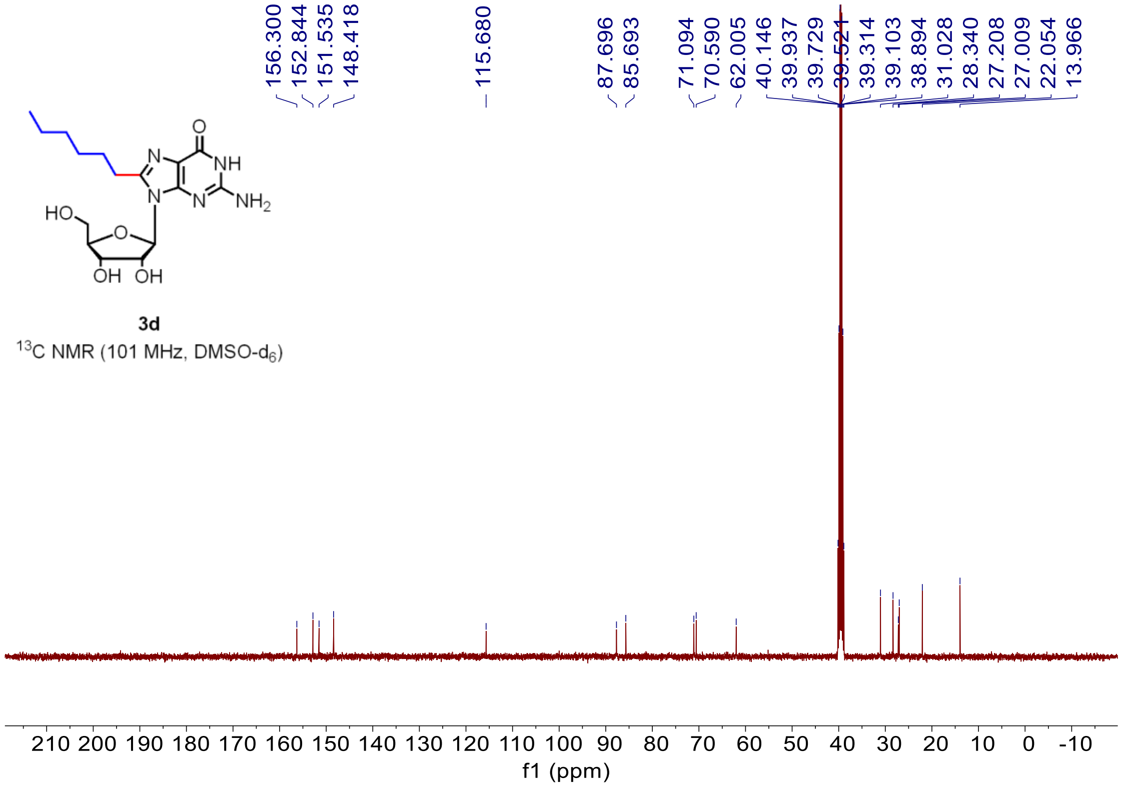

Supplementary Figure 146. ^{13}C NMR spectra of compound **3d**

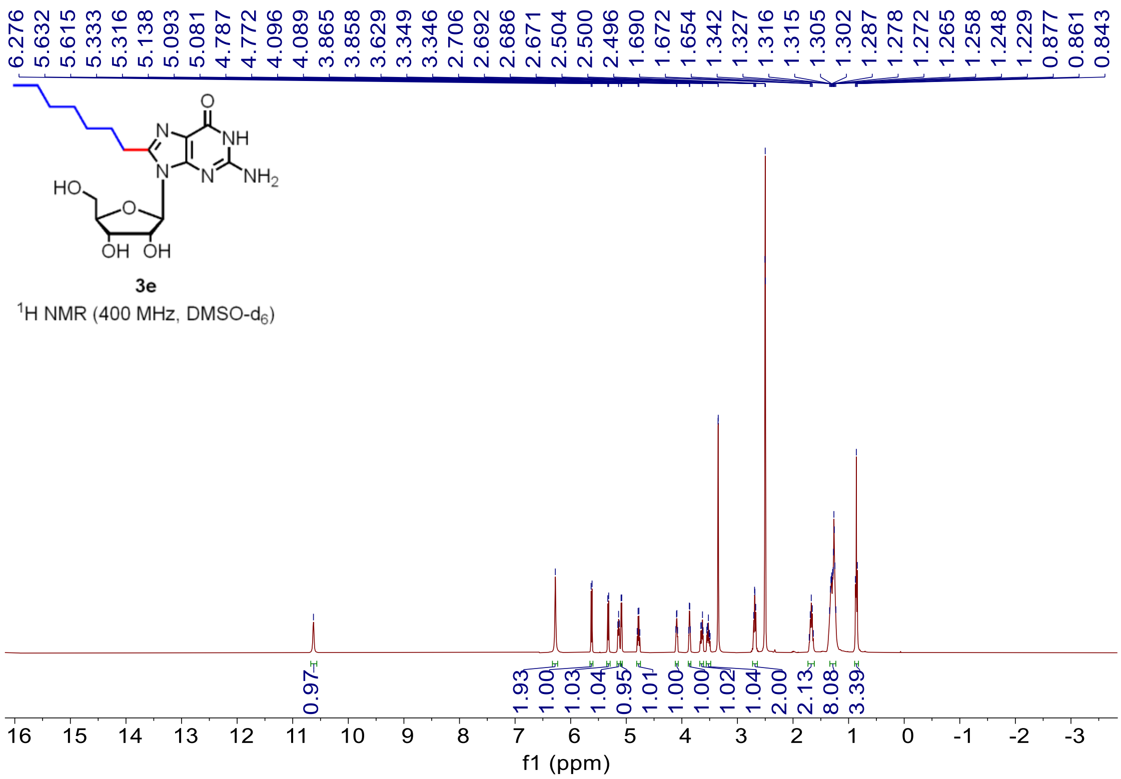

Supplementary Figure 147. ^{1}H NMR spectra of compound **3e**

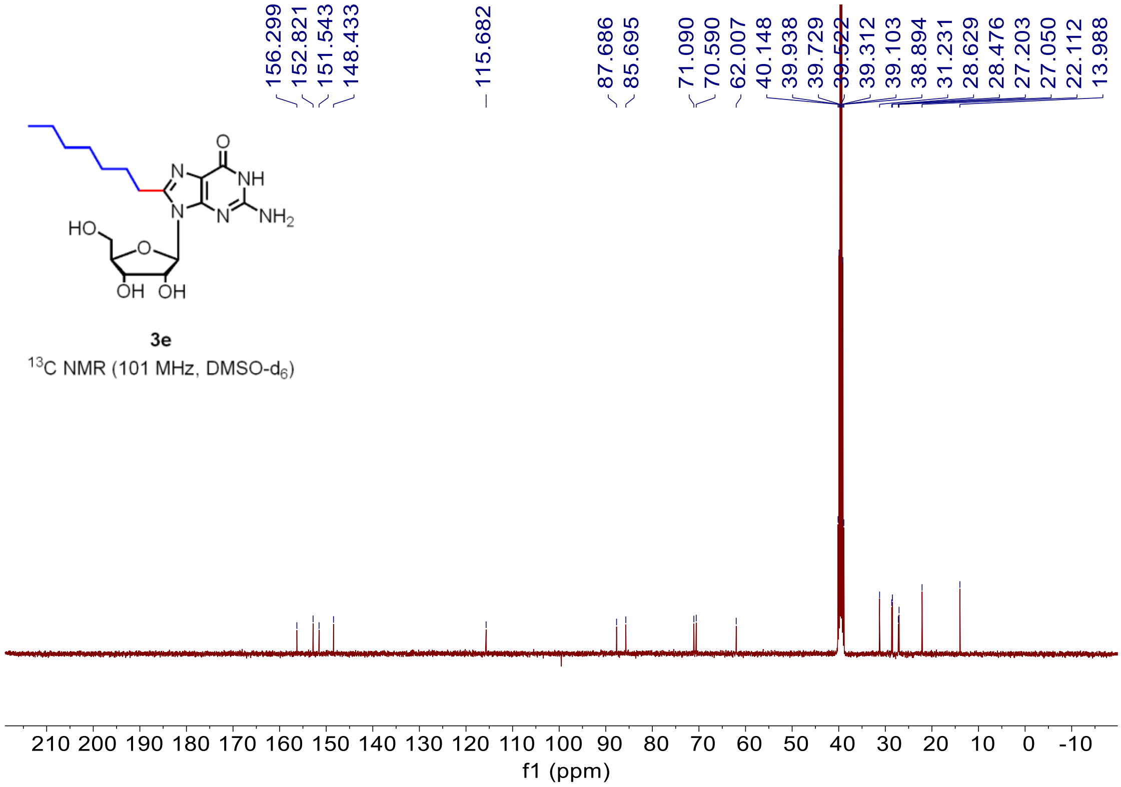

Supplementary Figure 148. ^{13}C NMR spectra of compound **3e**

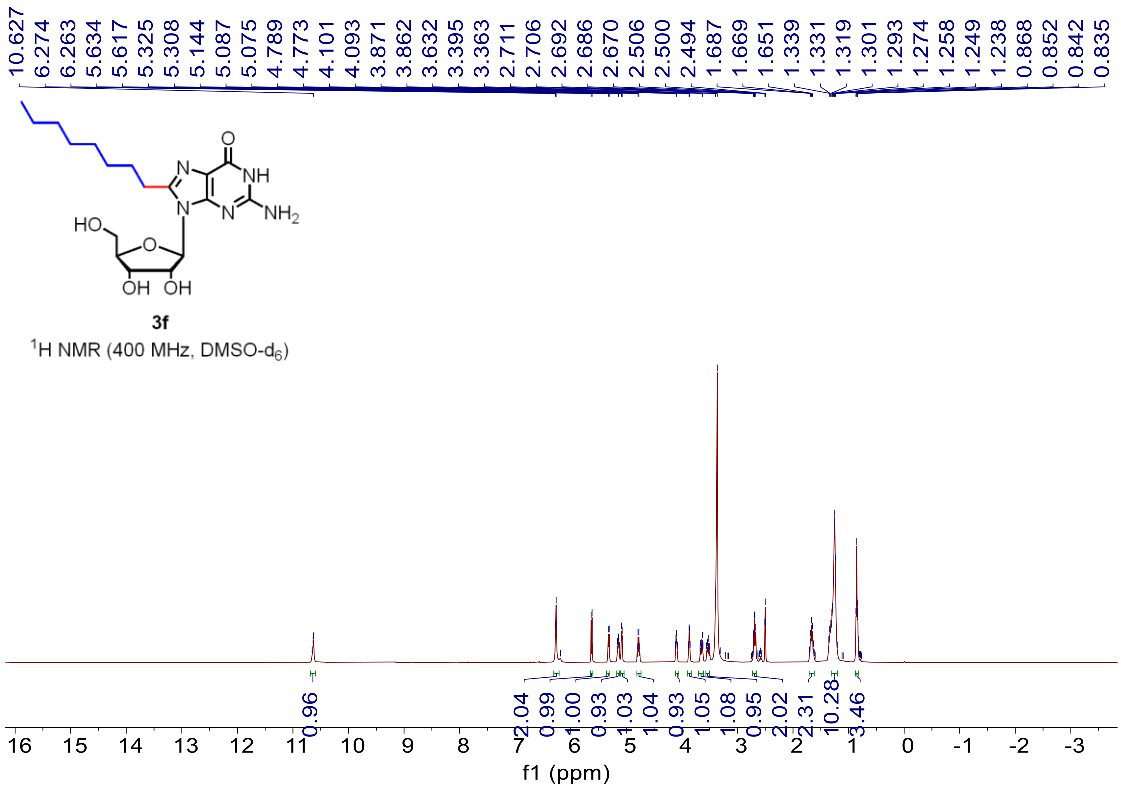

Supplementary Figure 149. ^{1}H NMR spectra of compound **3f**

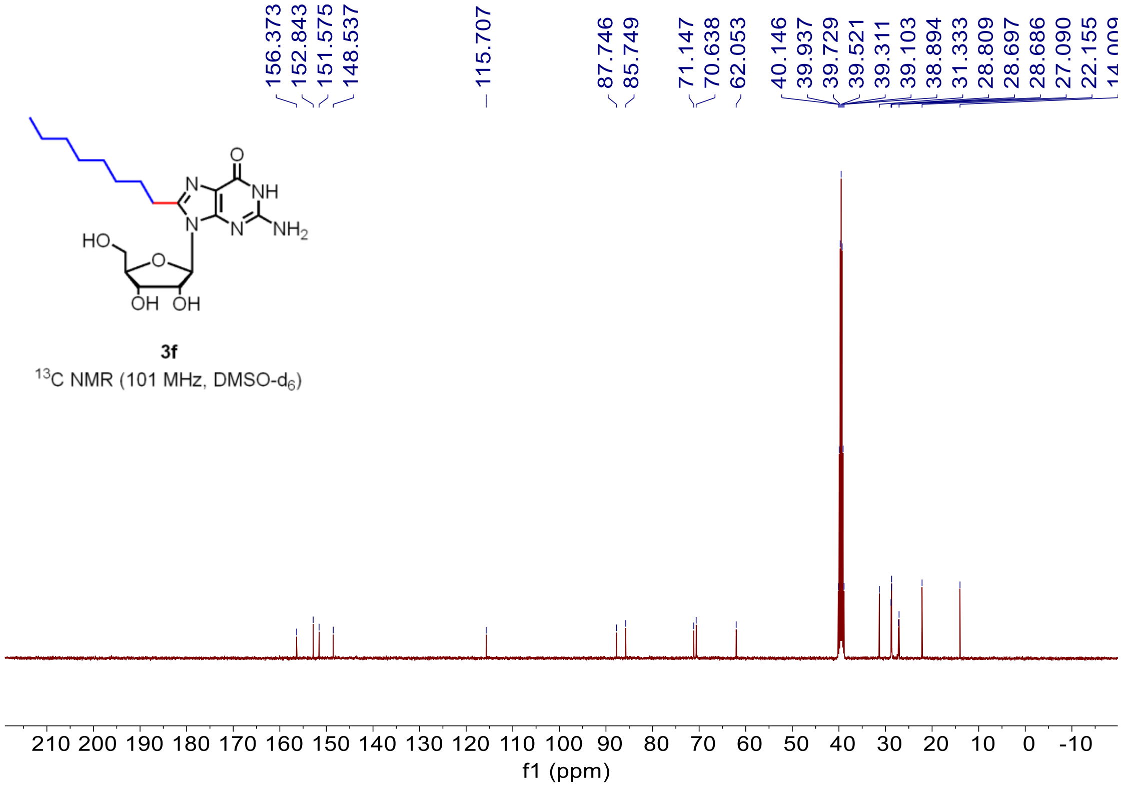

Supplementary Figure 150. ^{13}C NMR spectra of compound **3f**

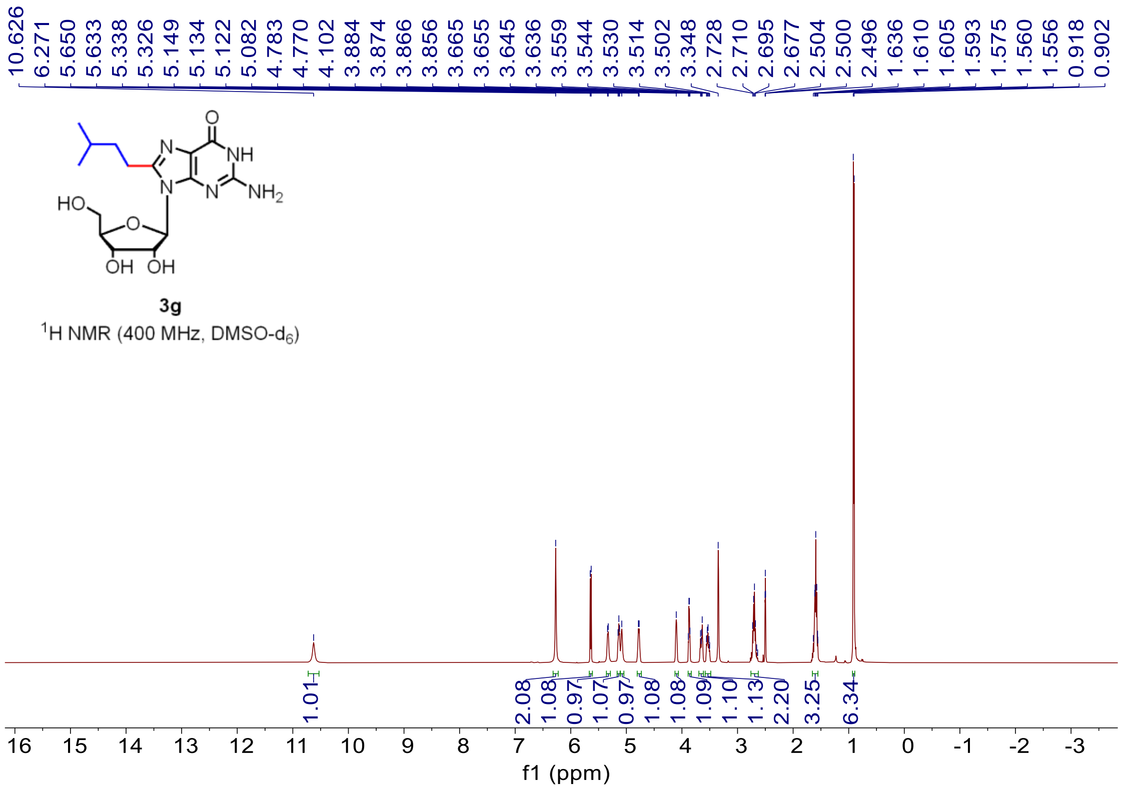

Supplementary Figure 151. ^{1}H NMR spectra of compound **3g**

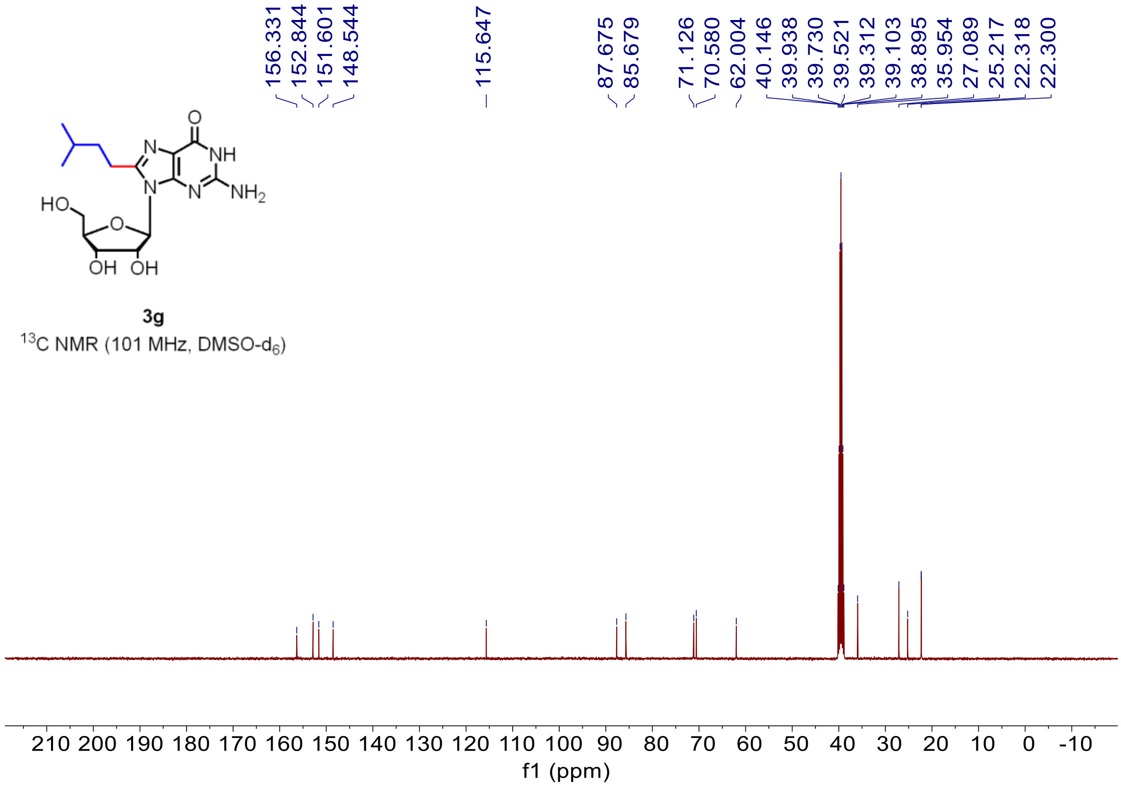

Supplementary Figure 152. ^{13}C NMR spectra of compound **3g**

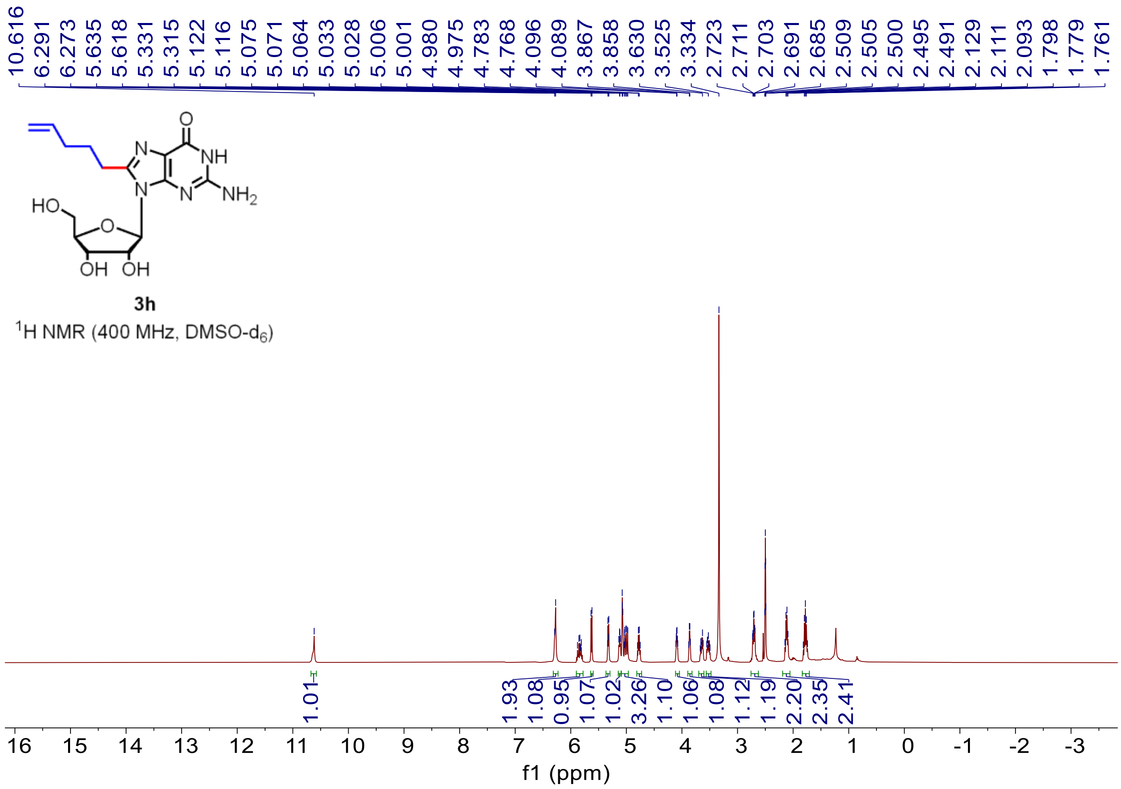

Supplementary Figure 153. ^{1}H NMR spectra of compound **3h**

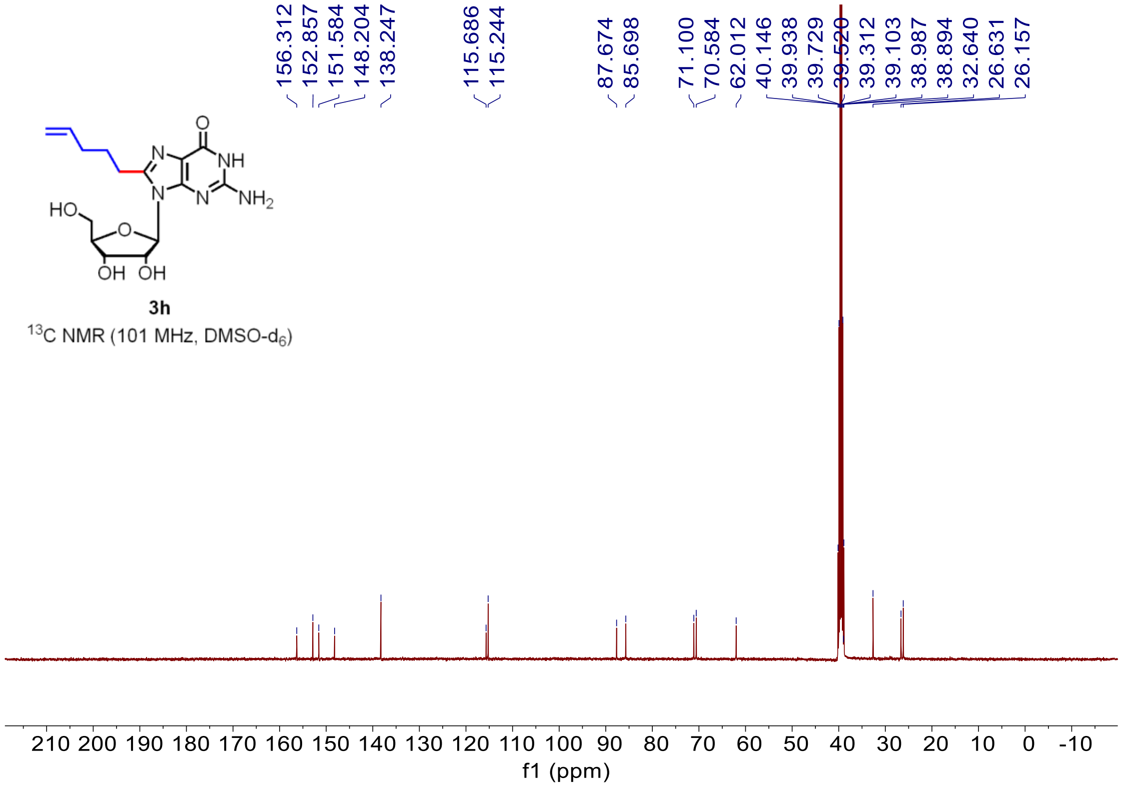

Supplementary Figure 154. ^{13}C NMR spectra of compound **3h**

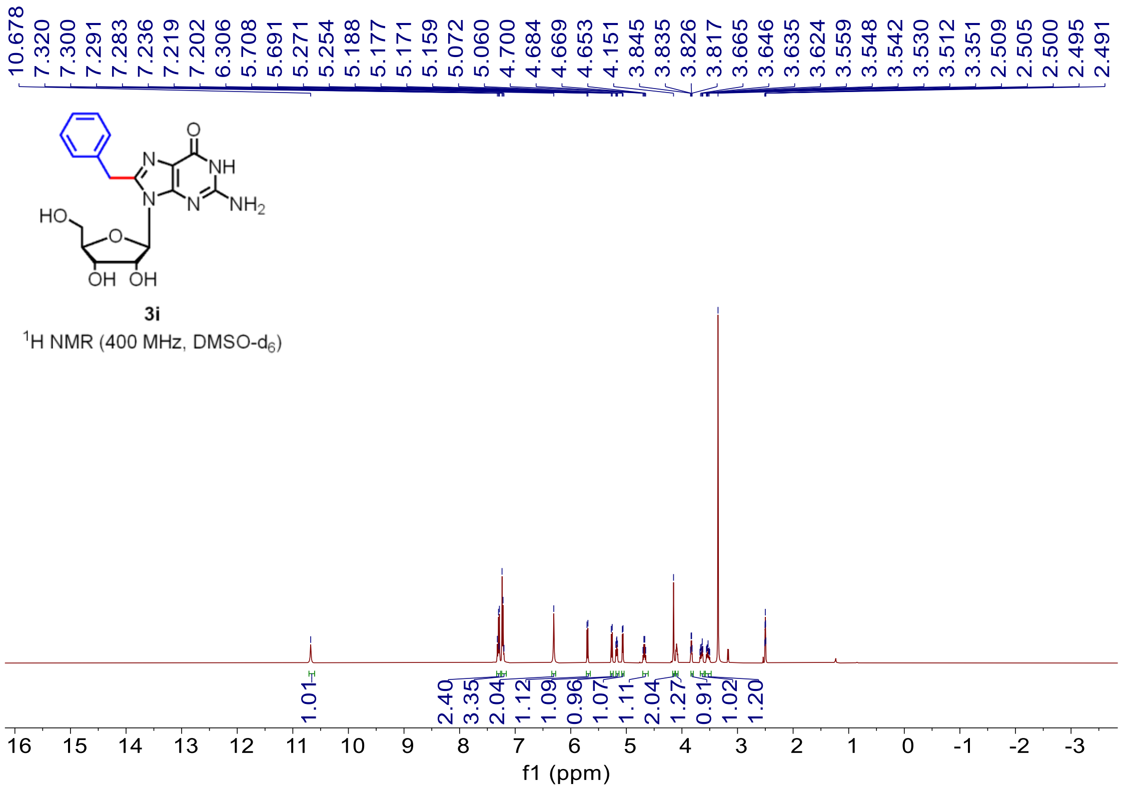

Supplementary Figure 155. ^{1}H NMR spectra of compound **3i**

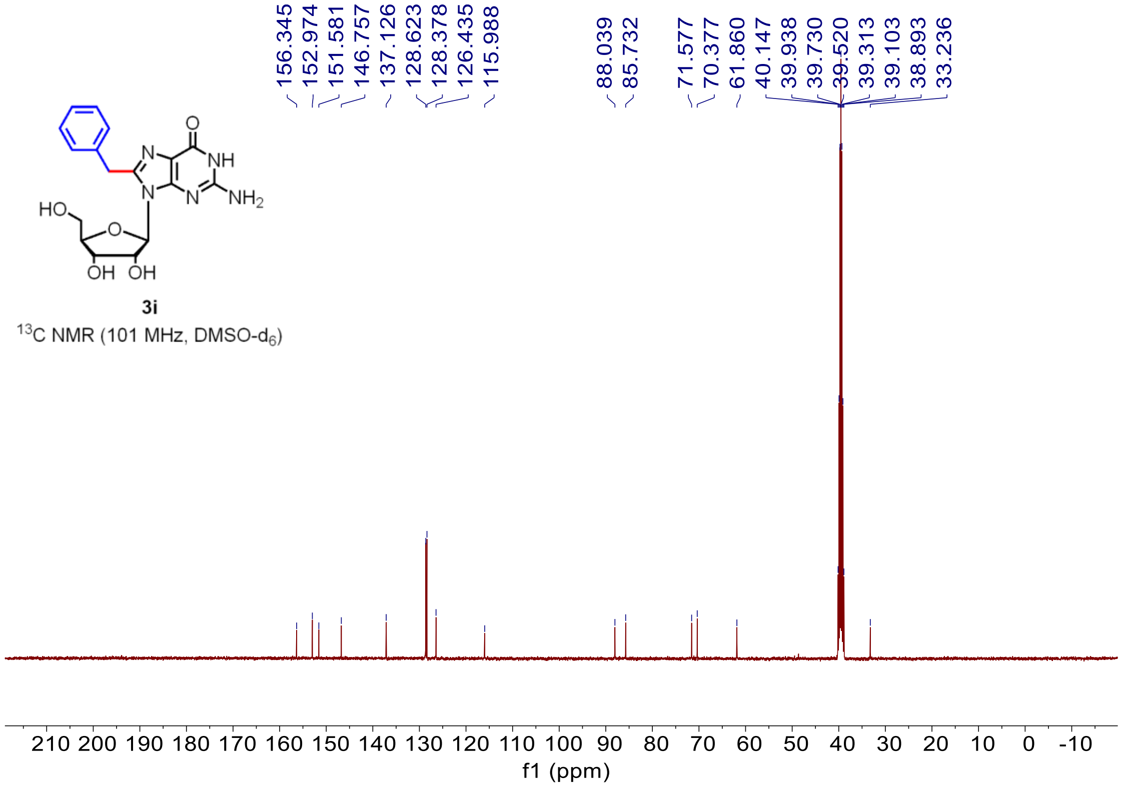

Supplementary Figure 156. ^{13}C NMR spectra of compound **3i**

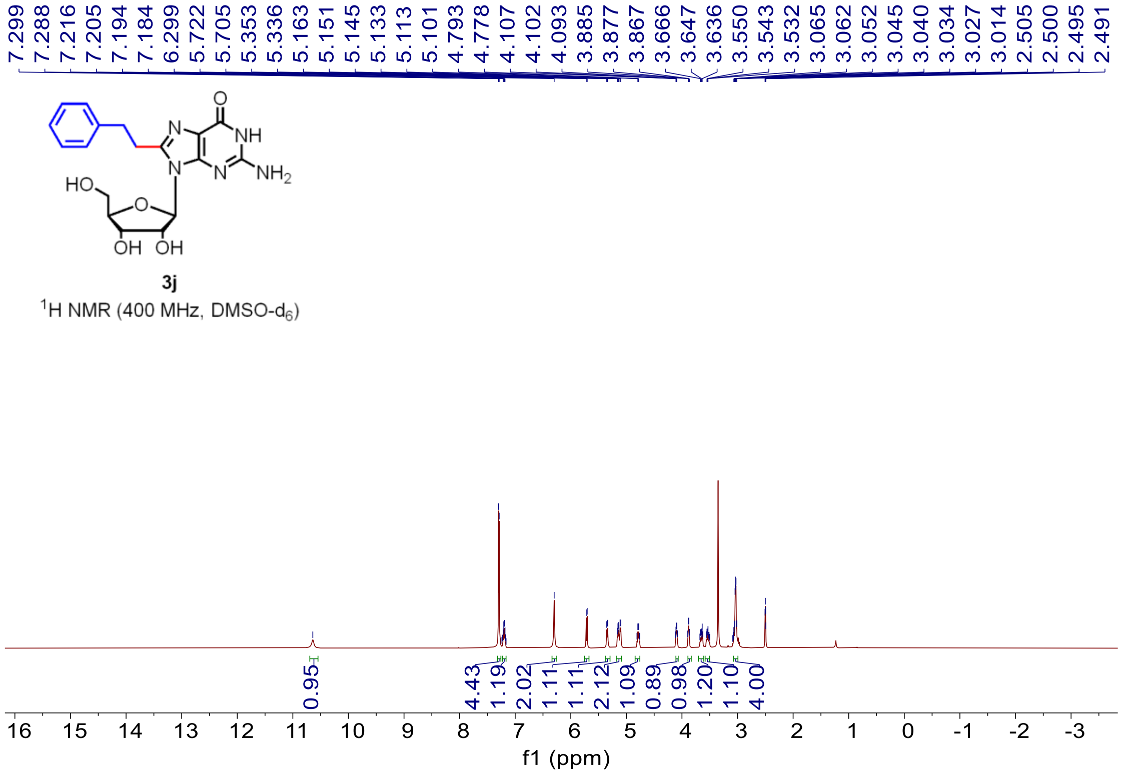

Supplementary Figure 157. ^{1}H NMR spectra of compound **3j**

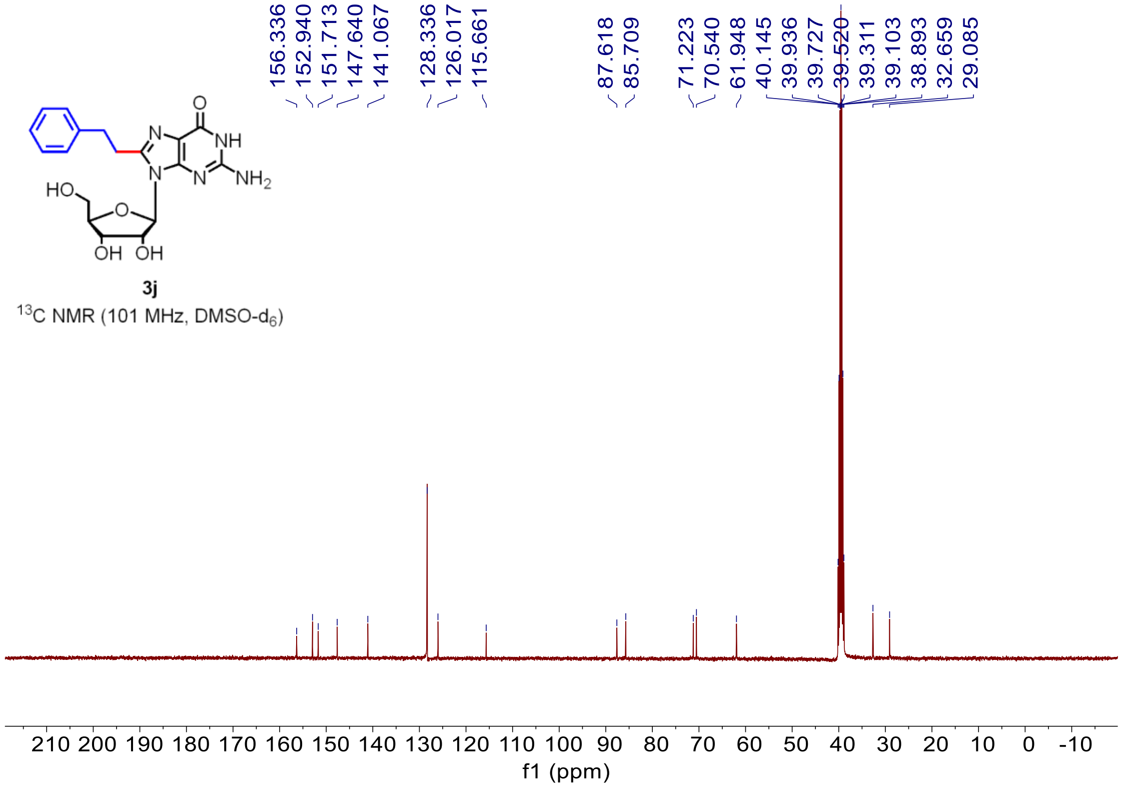

Supplementary Figure 158. ^{13}C NMR spectra of compound **3j**

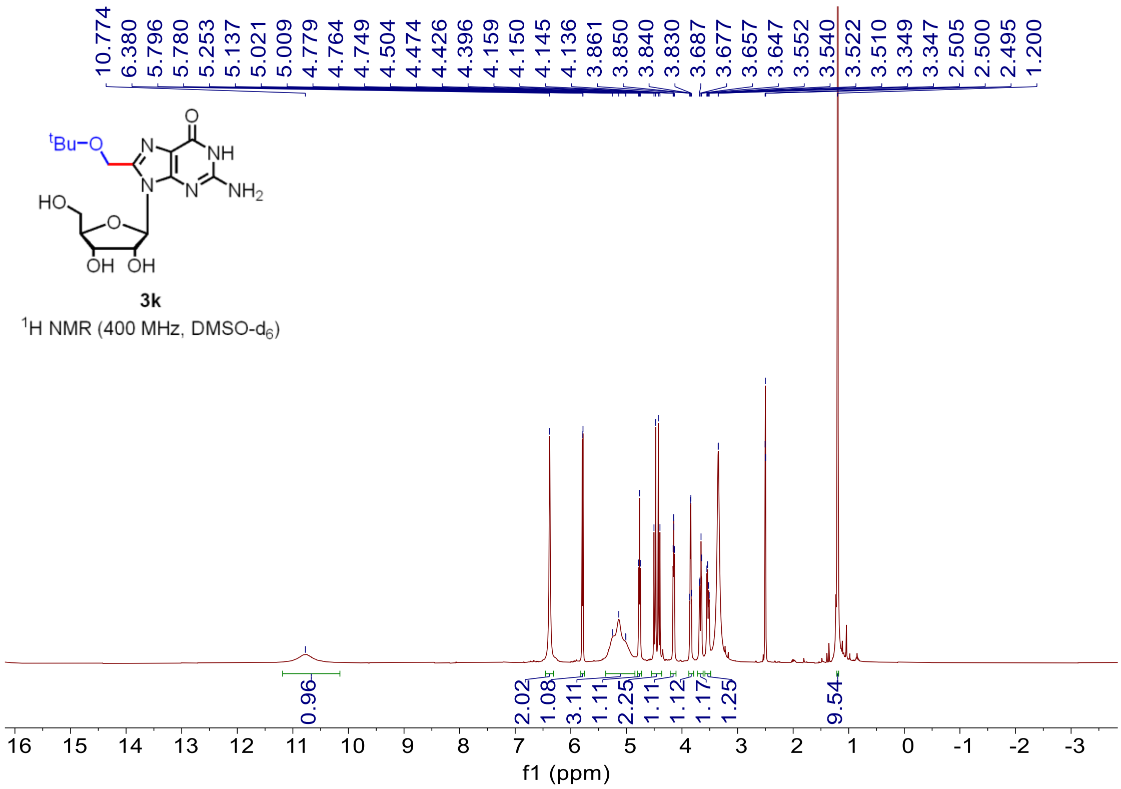

Supplementary Figure 159. ^{1}H NMR spectra of compound **3k**

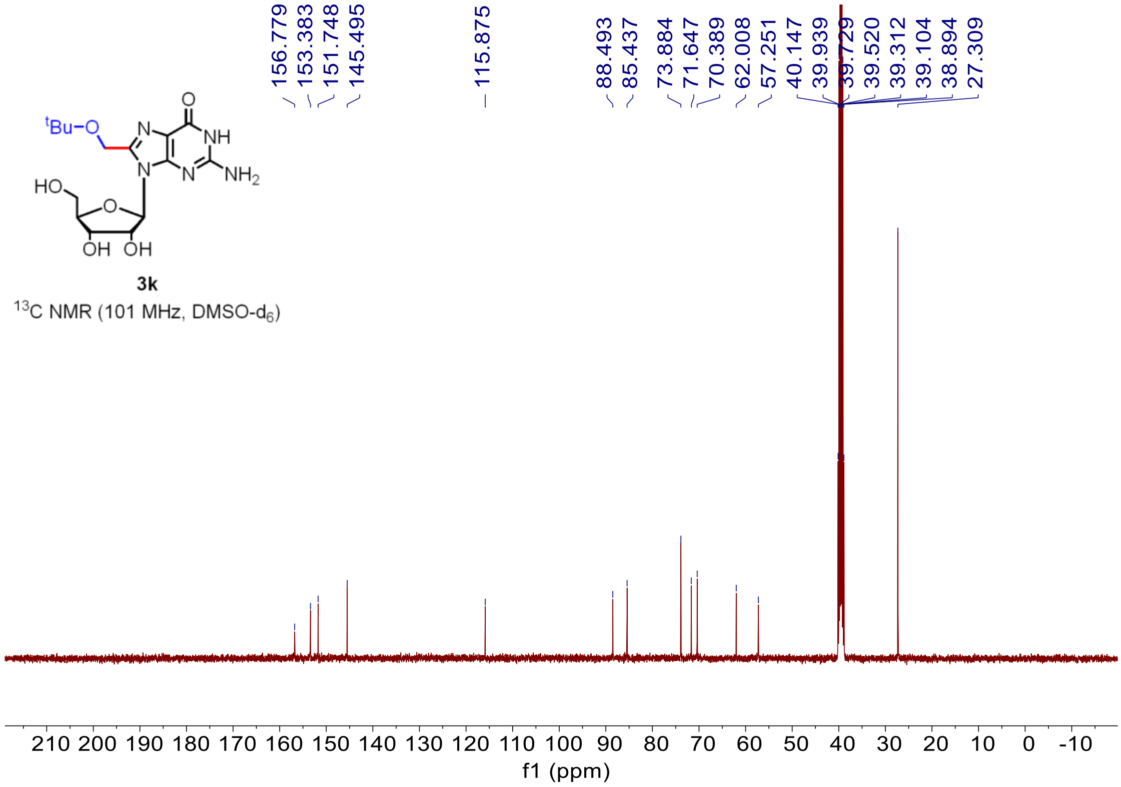

Supplementary Figure 160. ^{13}C NMR spectra of compound **3k**

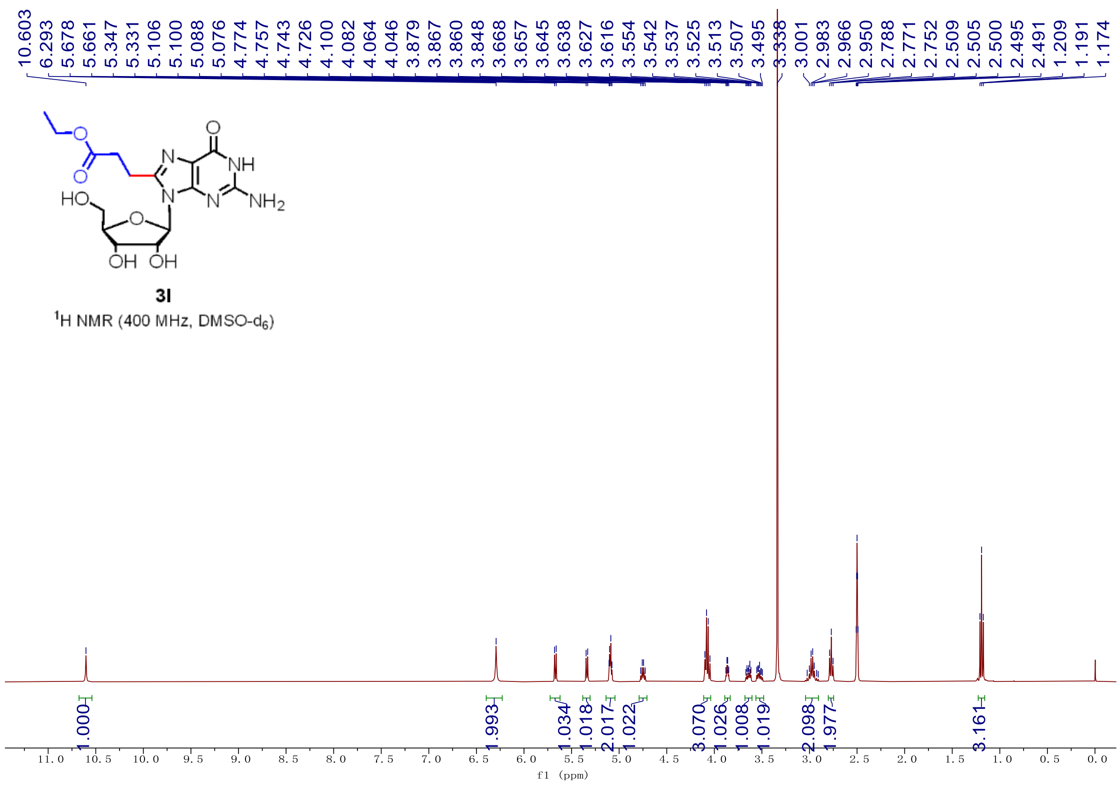

Supplementary Figure 161. ^{1}H NMR spectra of compound **3l**

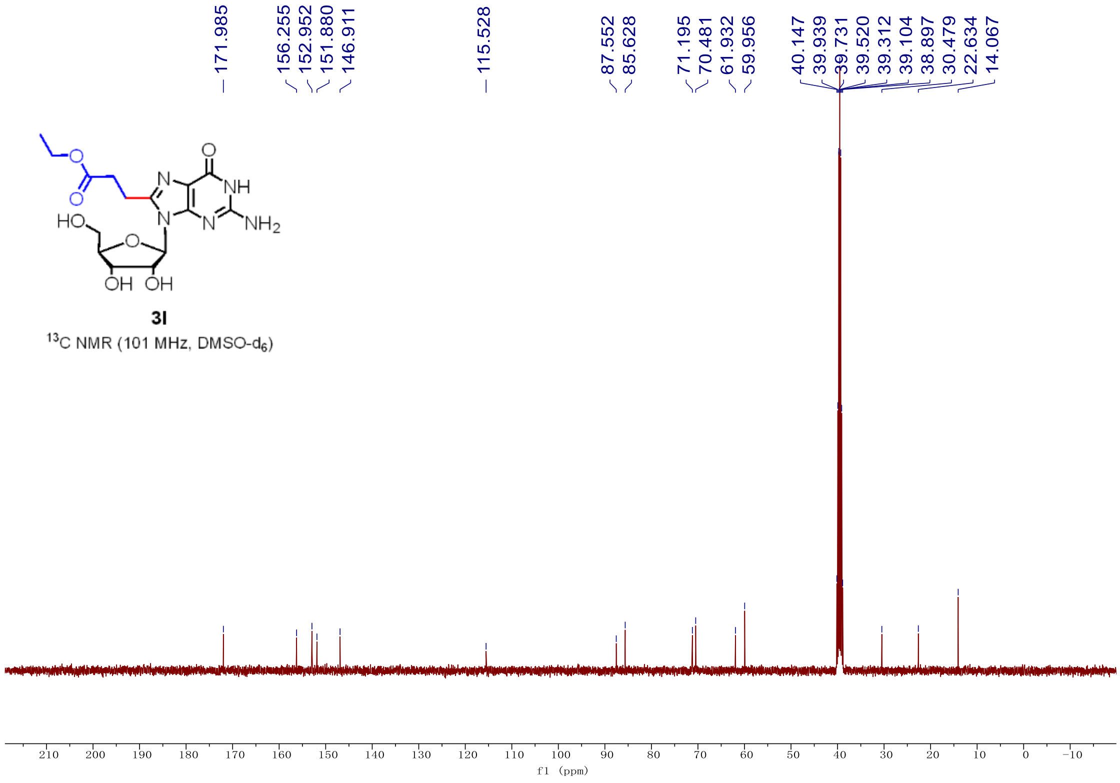

Supplementary Figure 162. ^{13}C NMR spectra of compound **3l**

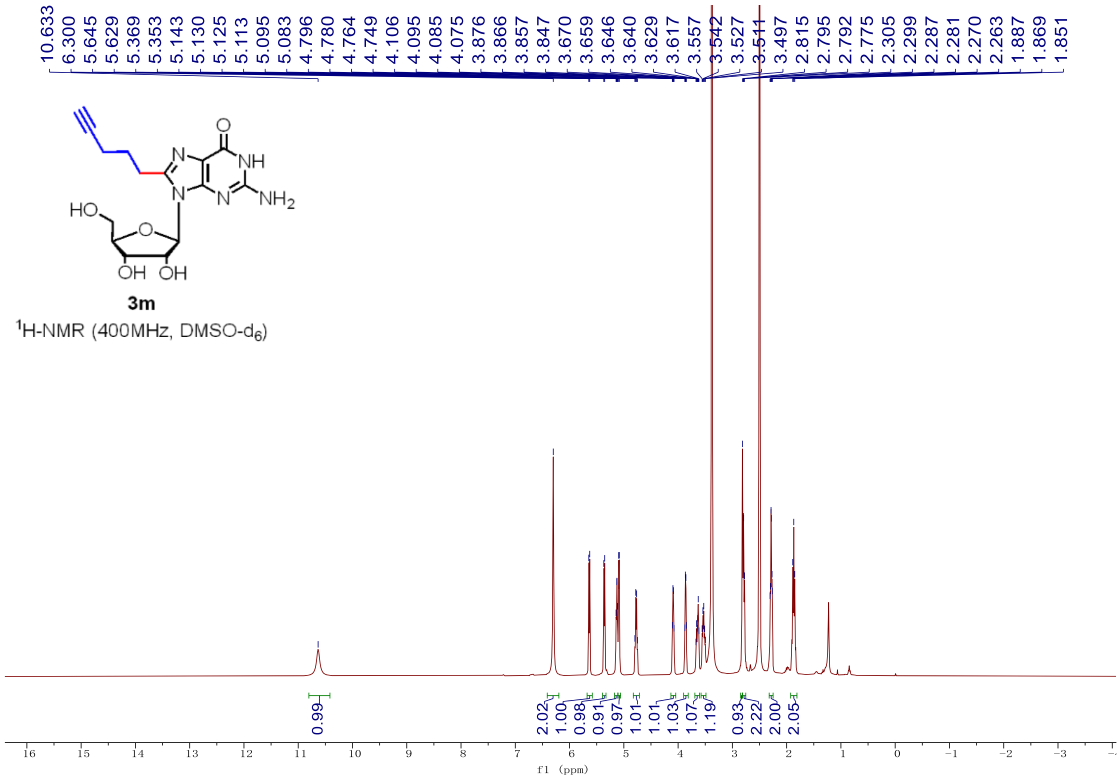

Supplementary Figure 163. ^{1}H NMR spectra of compound **3m**

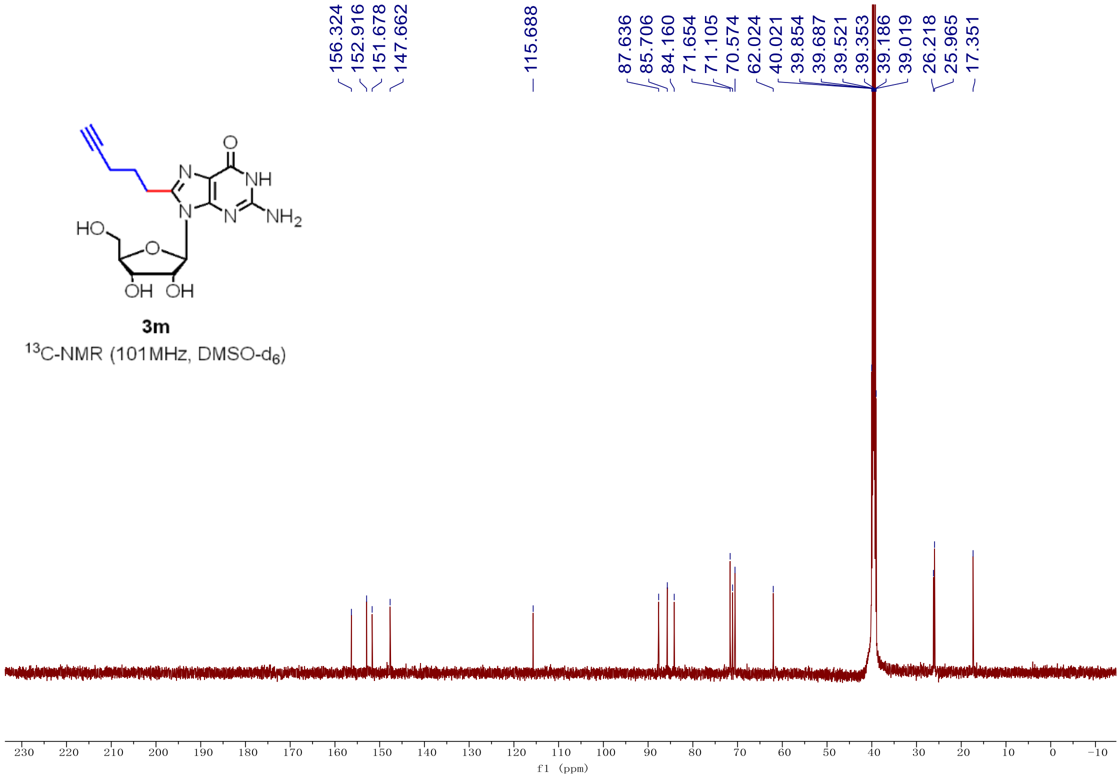

Supplementary Figure 164. ^{13}C NMR spectra of compound **3m**

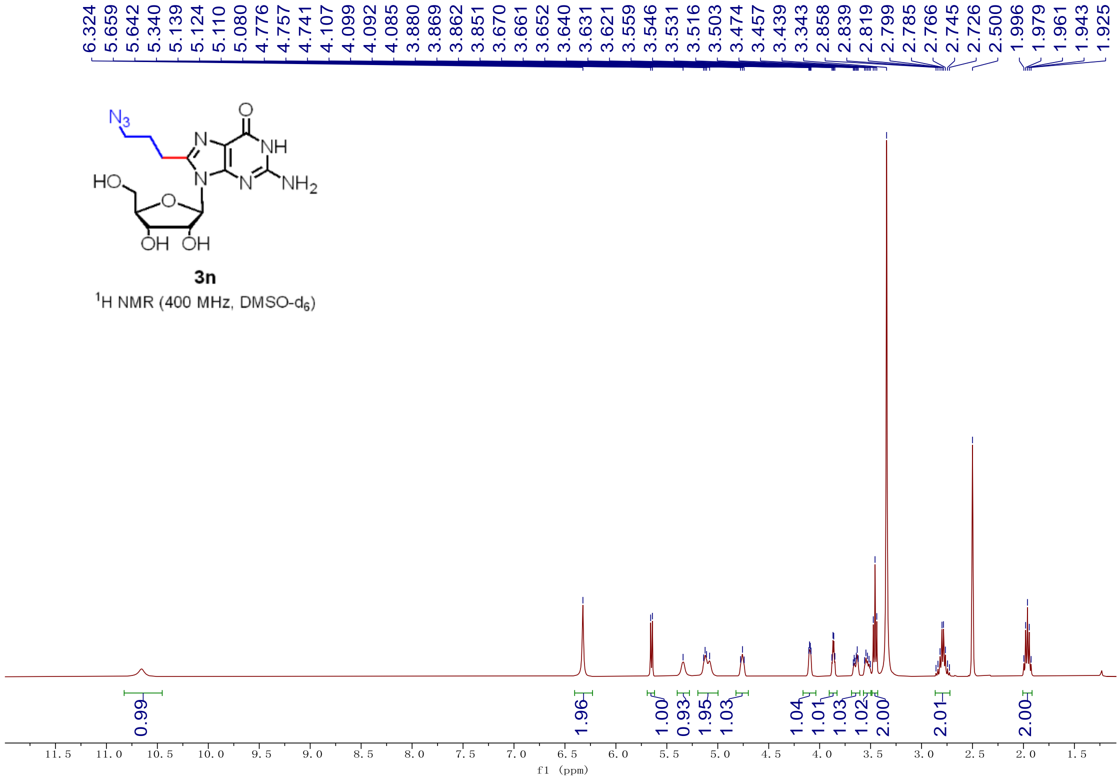

Supplementary Figure 165. ^{1}H NMR spectra of compound **3n**

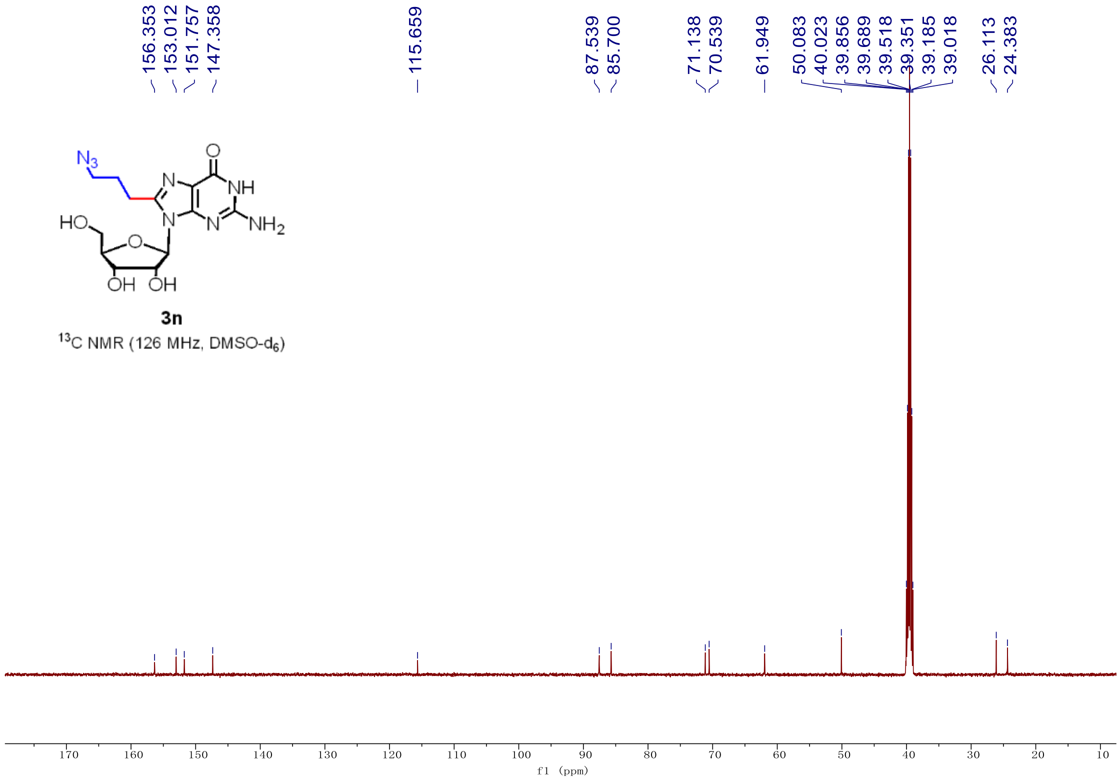

Supplementary Figure 166. ^{13}C NMR spectra of compound **3n**

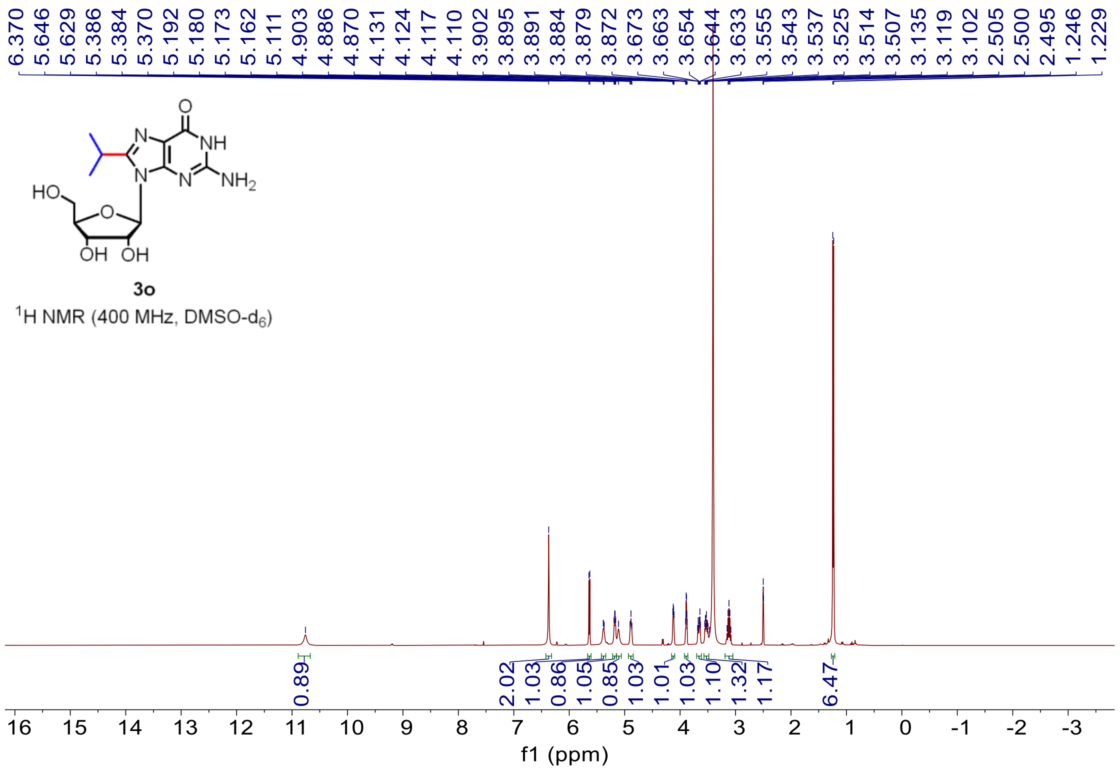

Supplementary Figure 167. ^{1}H NMR spectra of compound **3o**

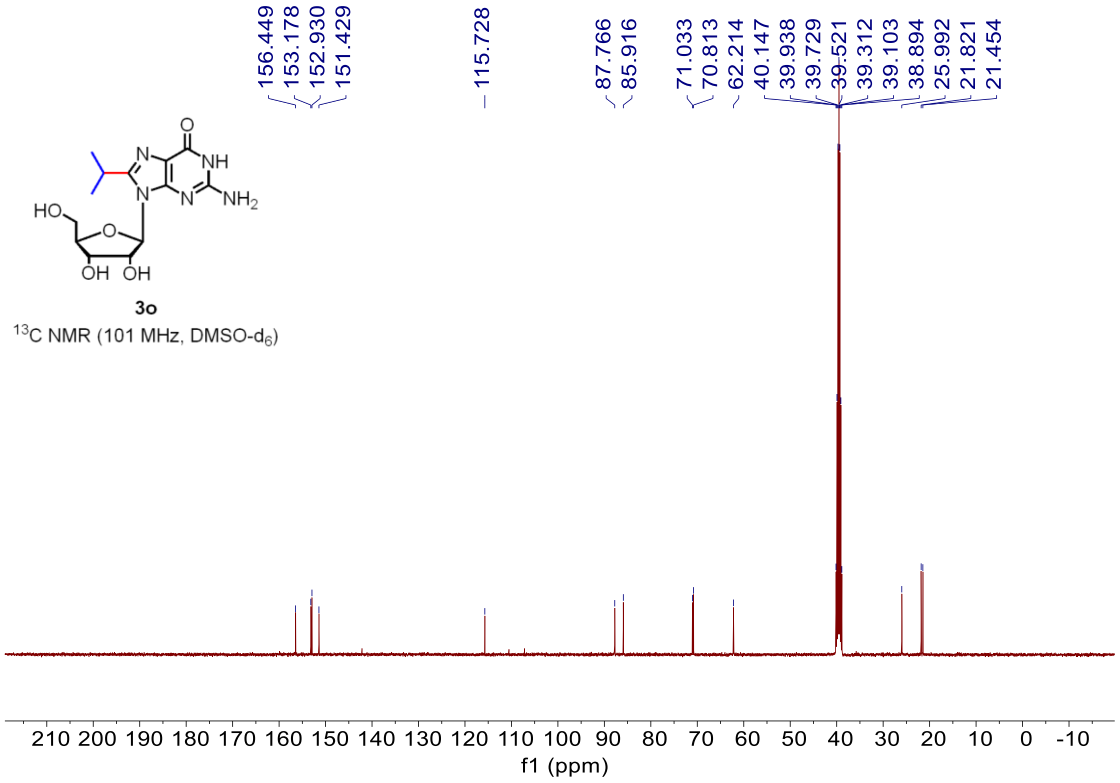

Supplementary Figure 168. ^{13}C NMR spectra of compound **3o**

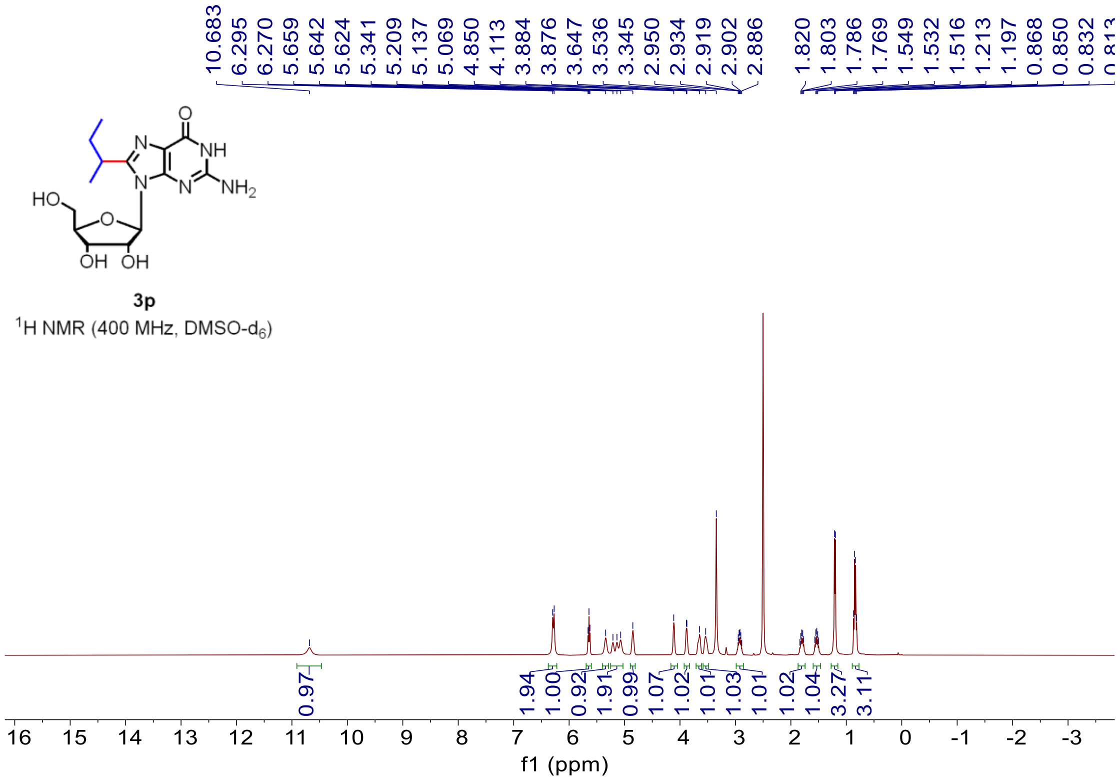

Supplementary Figure 169. ^{1}H NMR spectra of compound **3p**

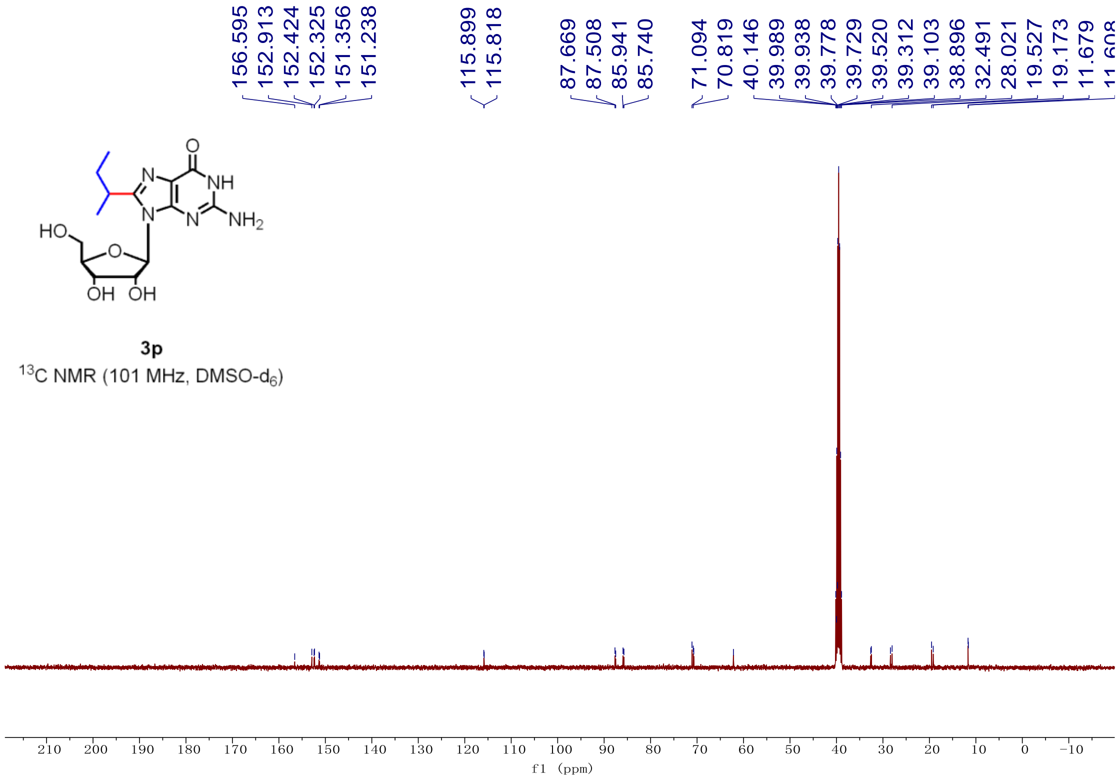

Supplementary Figure 170. ^{13}C NMR spectra of compound **3p**

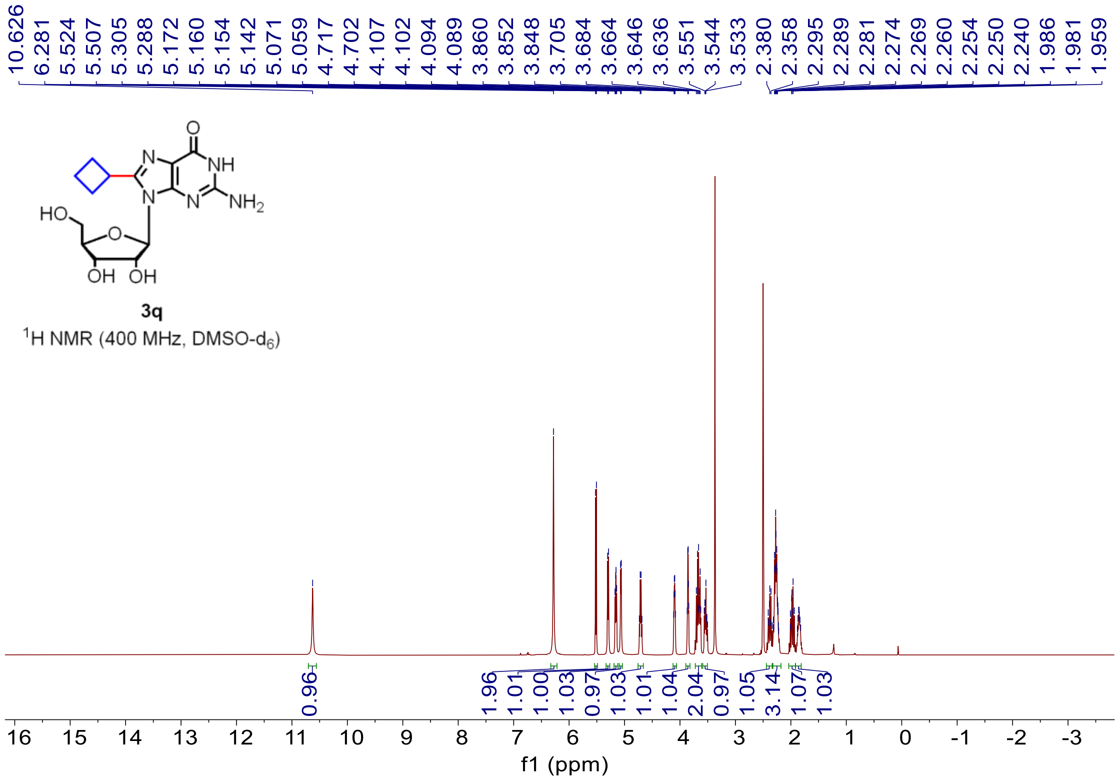

Supplementary Figure 171. ^{1}H NMR spectra of compound **3q**

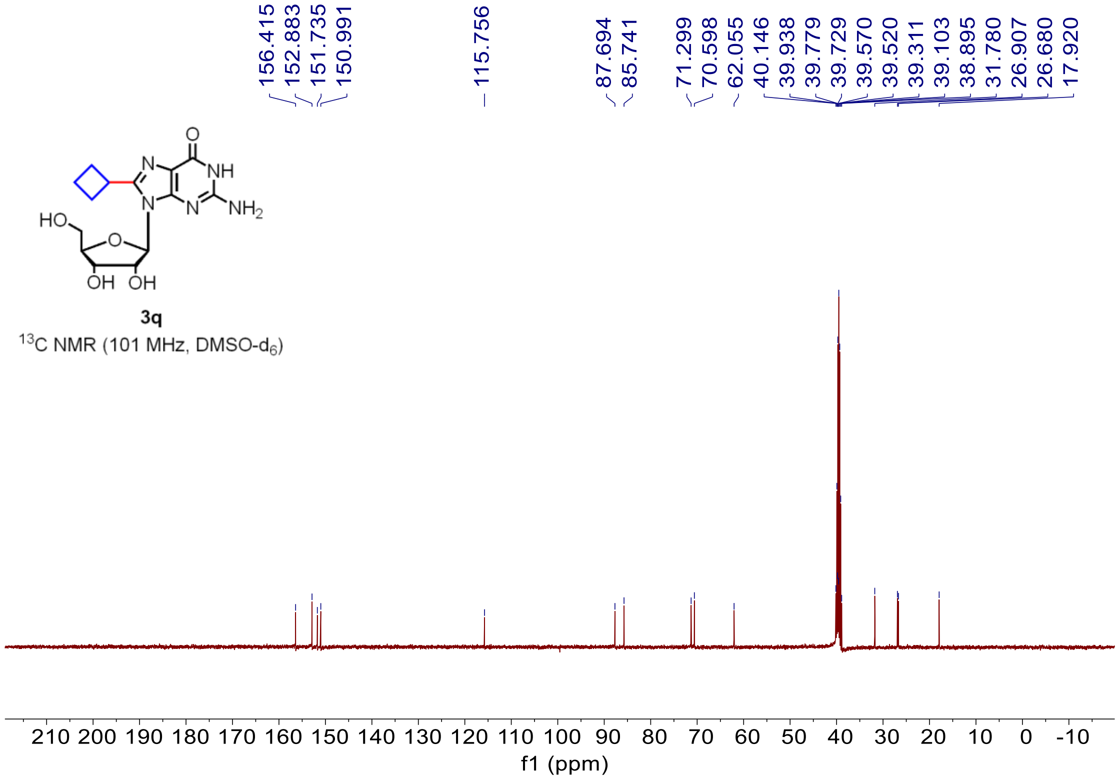

Supplementary Figure 172. ^{13}C NMR spectra of compound **3q**

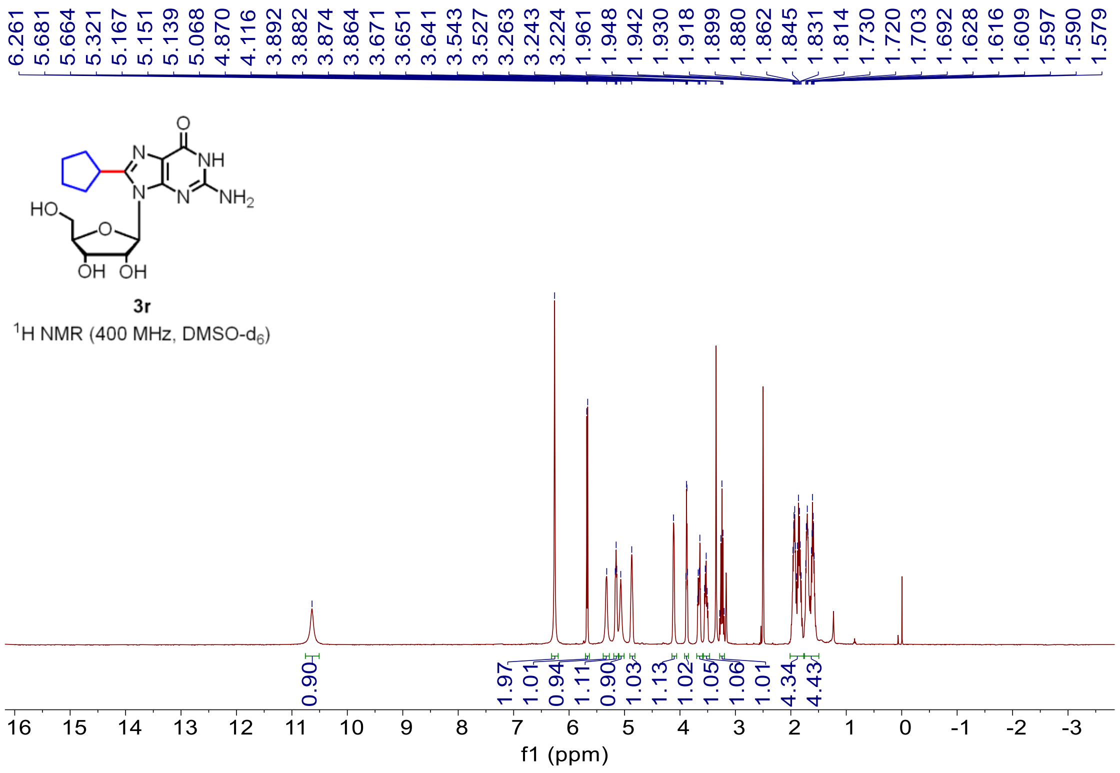

Supplementary Figure 173. ^{1}H NMR spectra of compound **3r**

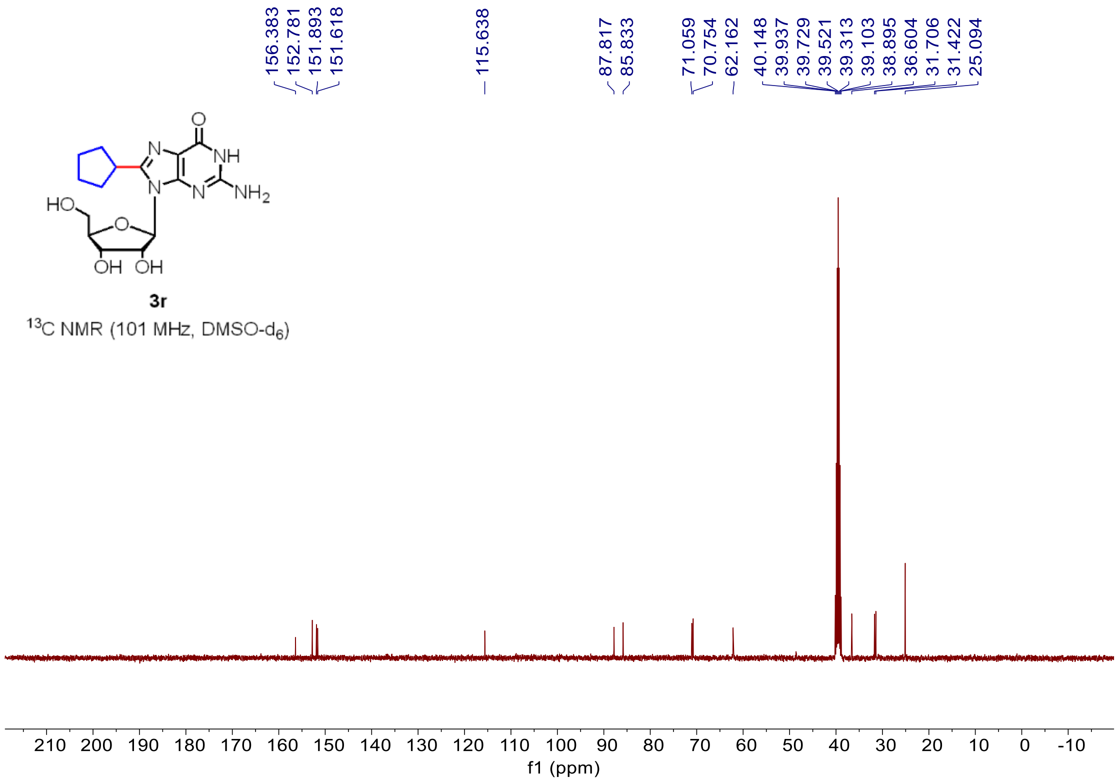

Supplementary Figure 174. ^{13}C NMR spectra of compound **3r**

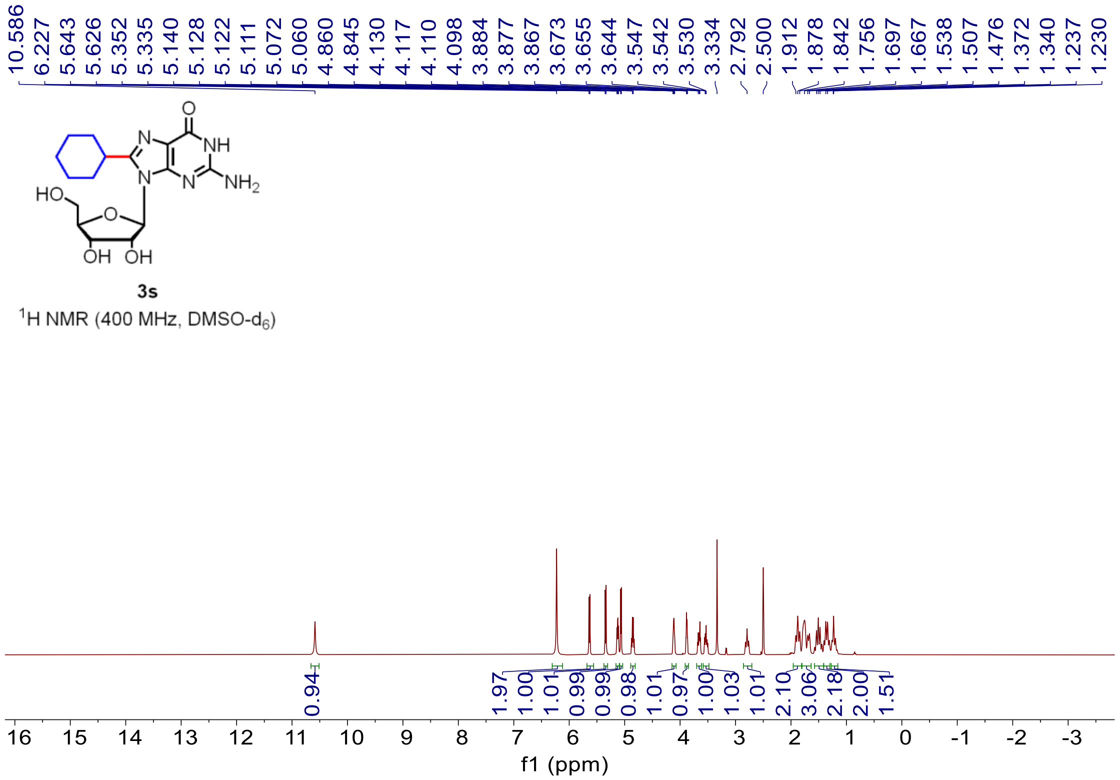

Supplementary Figure 175. ^{1}H NMR spectra of compound **3s**

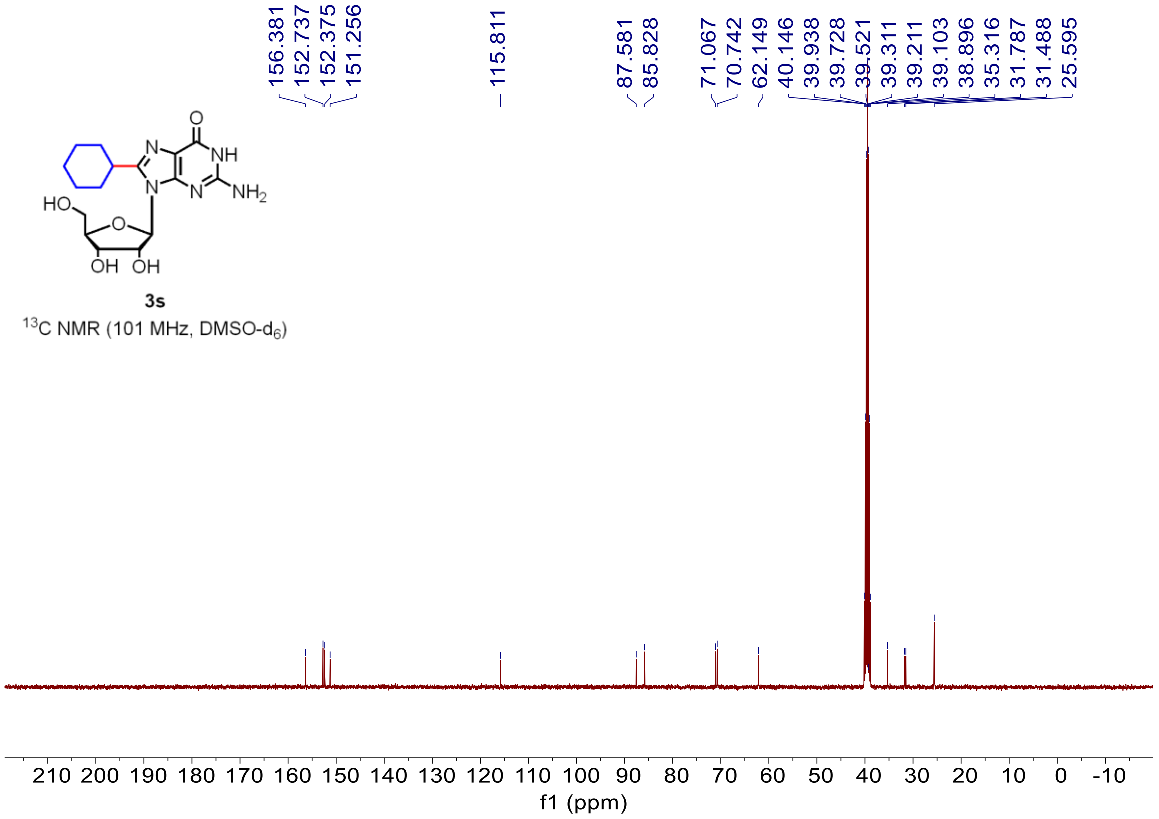

Supplementary Figure 176. ^{13}C NMR spectra of compound **3s**

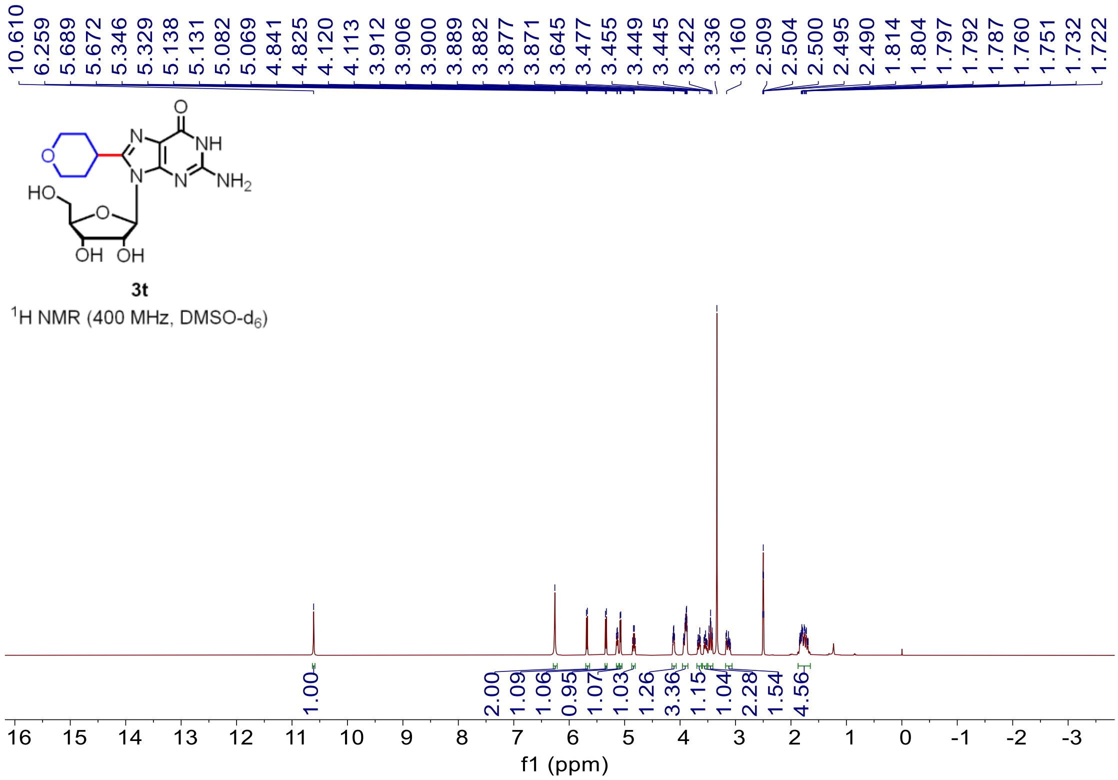

Supplementary Figure 177. ^{1}H NMR spectra of compound **3t**

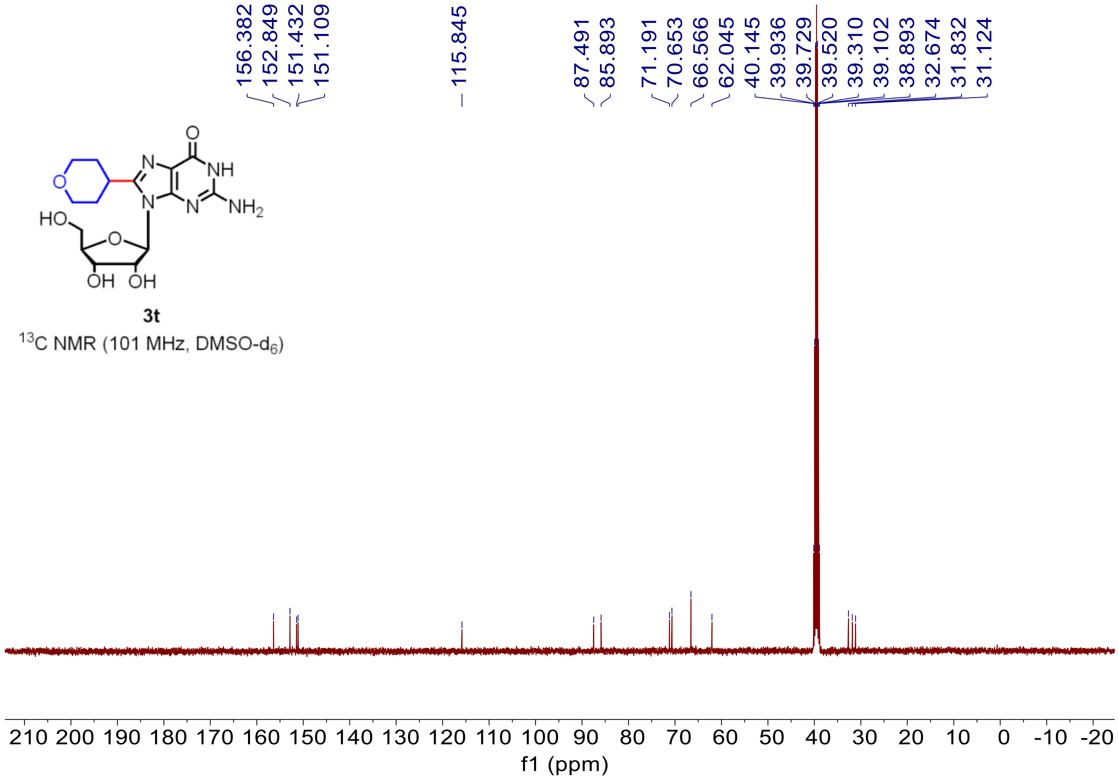

Supplementary Figure 178. ^{13}C NMR spectra of compound **3t**

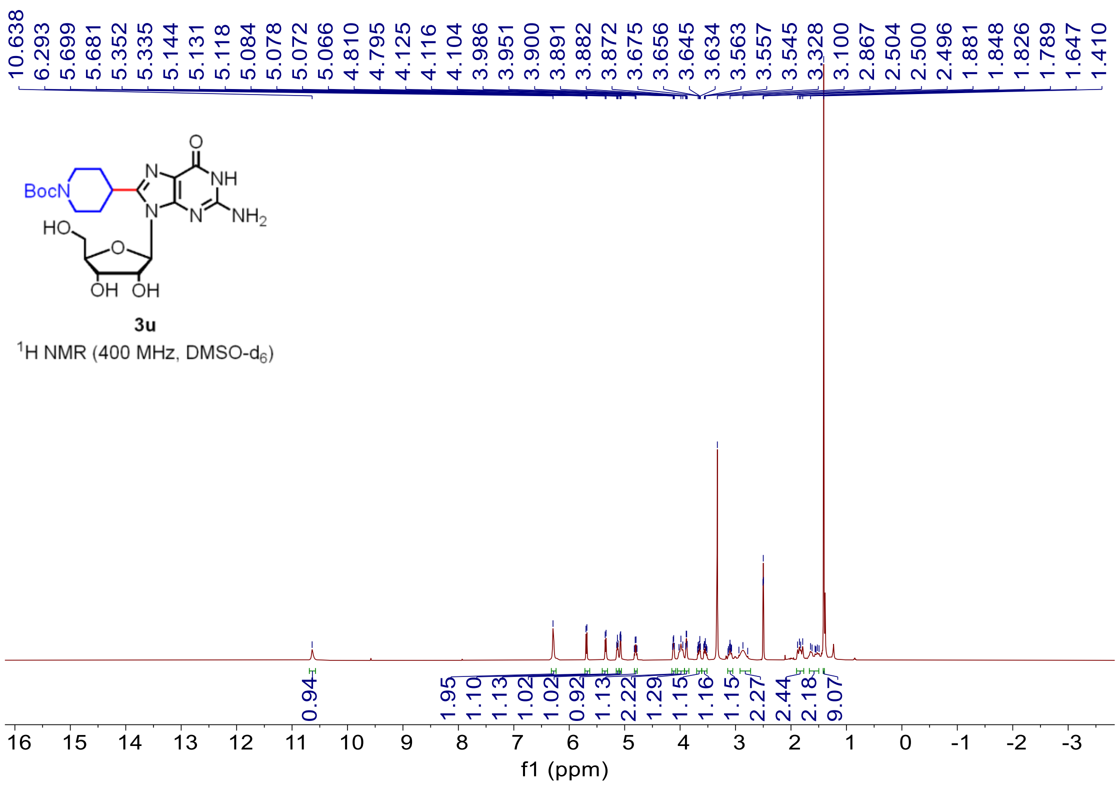

Supplementary Figure 179. ^{1}H NMR spectra of compound **3u**

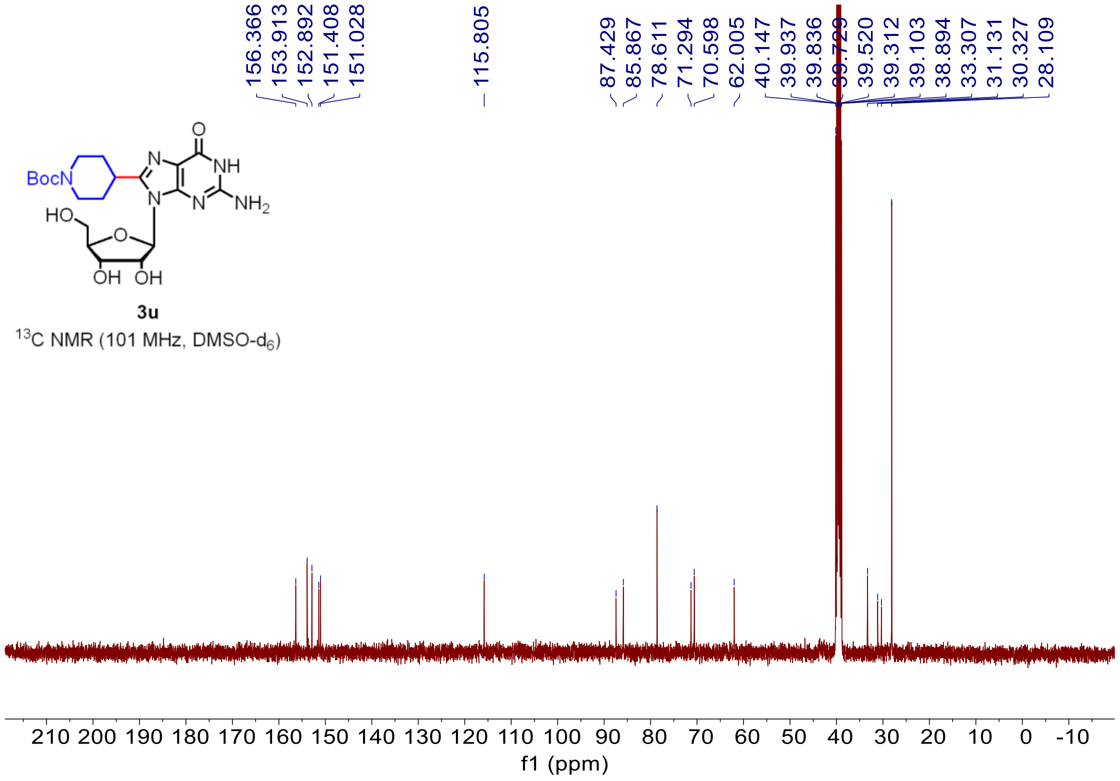

Supplementary Figure 180. ^{13}C NMR spectra of compound **3u**

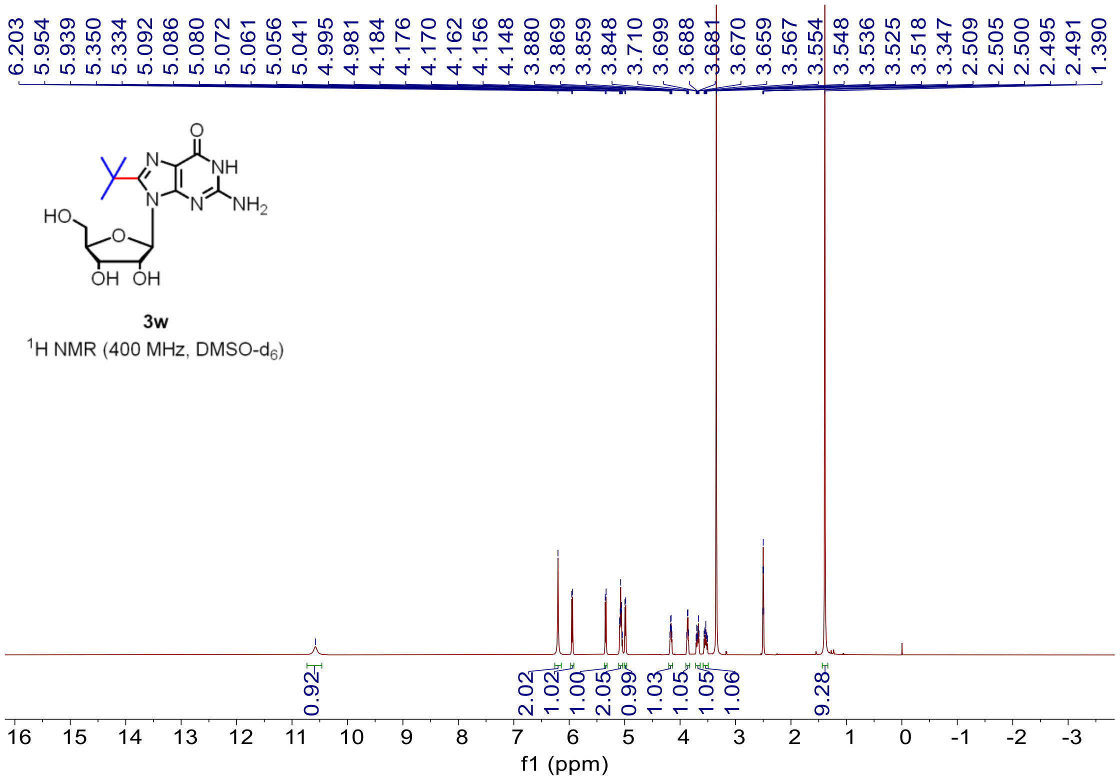

Supplementary Figure 181. ^{1}H NMR spectra of compound **3w**

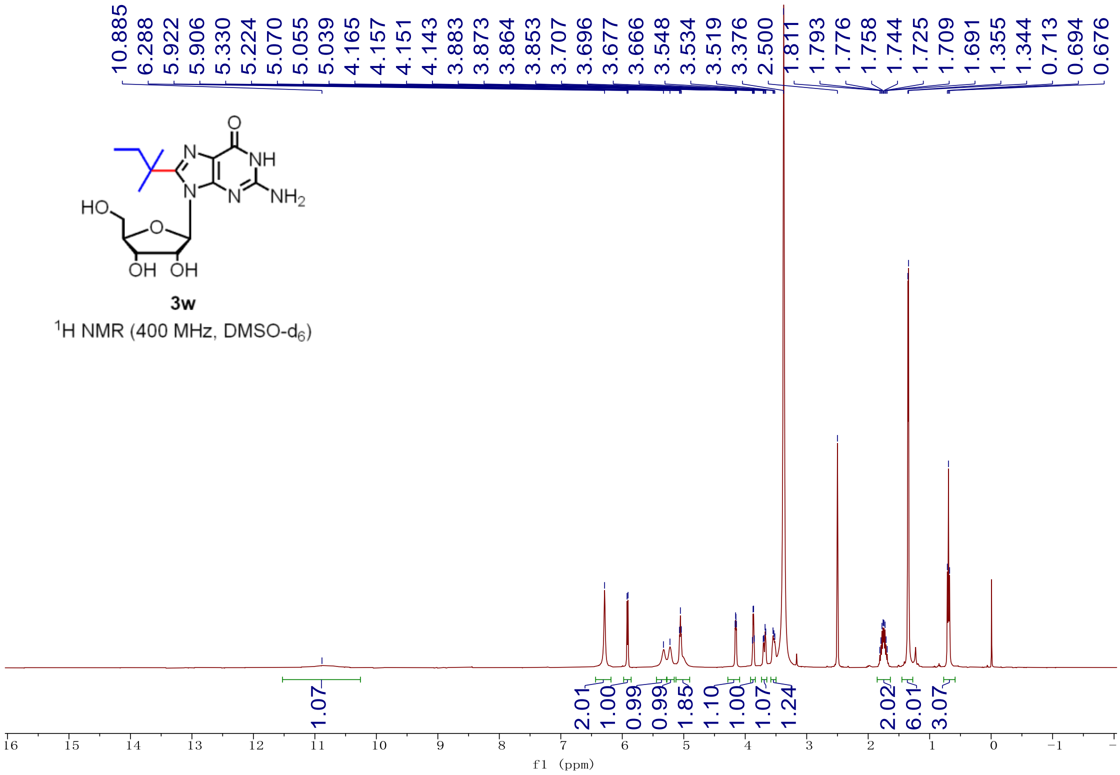

Supplementary Figure 182. ^{1}H NMR spectra of compound **3w**

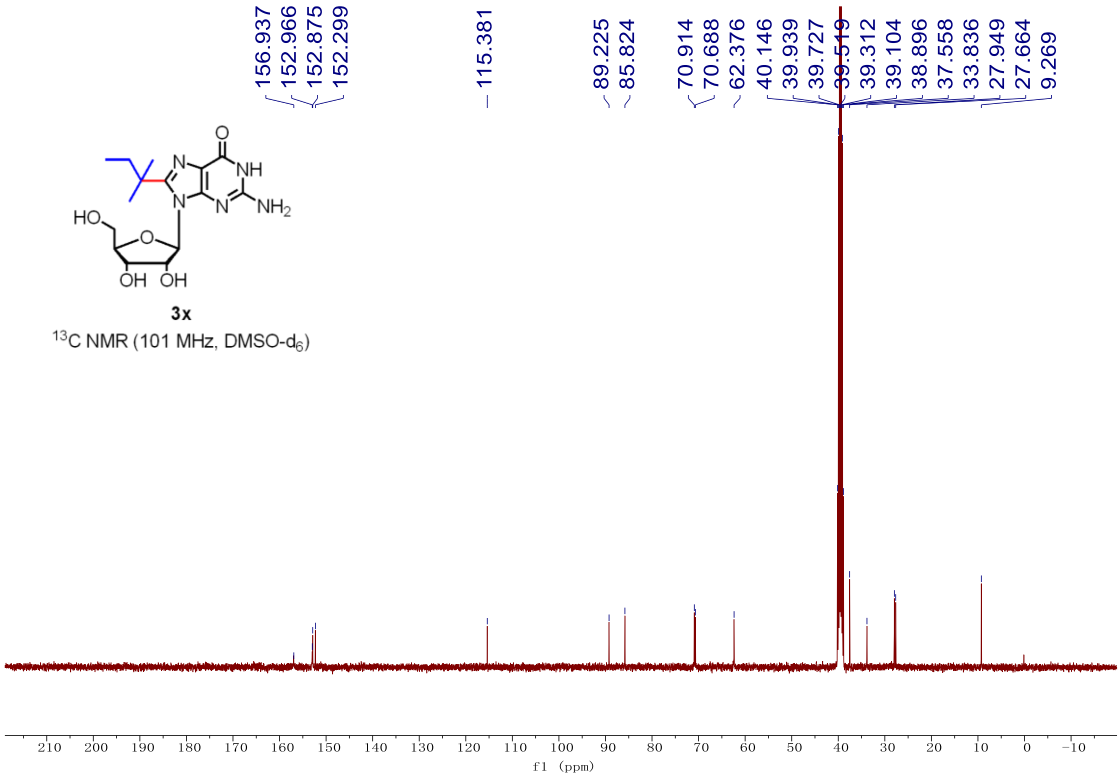

Supplementary Figure 183. ^{13}C NMR spectra of compound **3x**

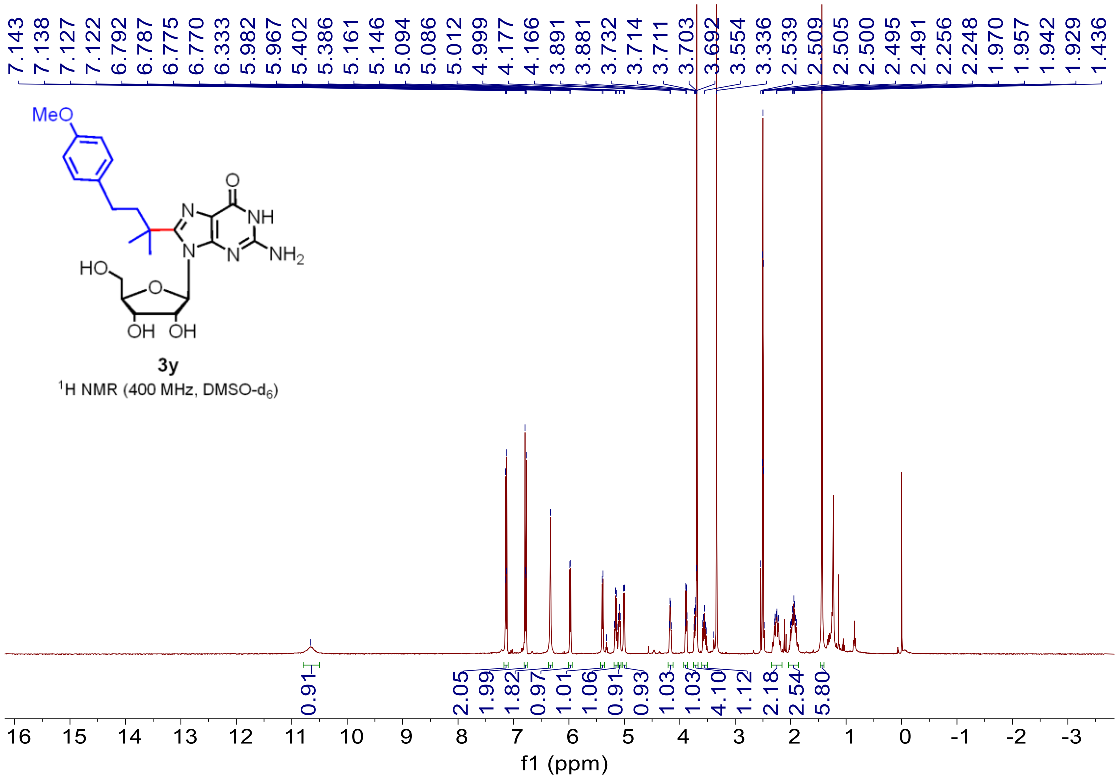

Supplementary Figure 184. ^{1}H NMR spectra of compound **3y**

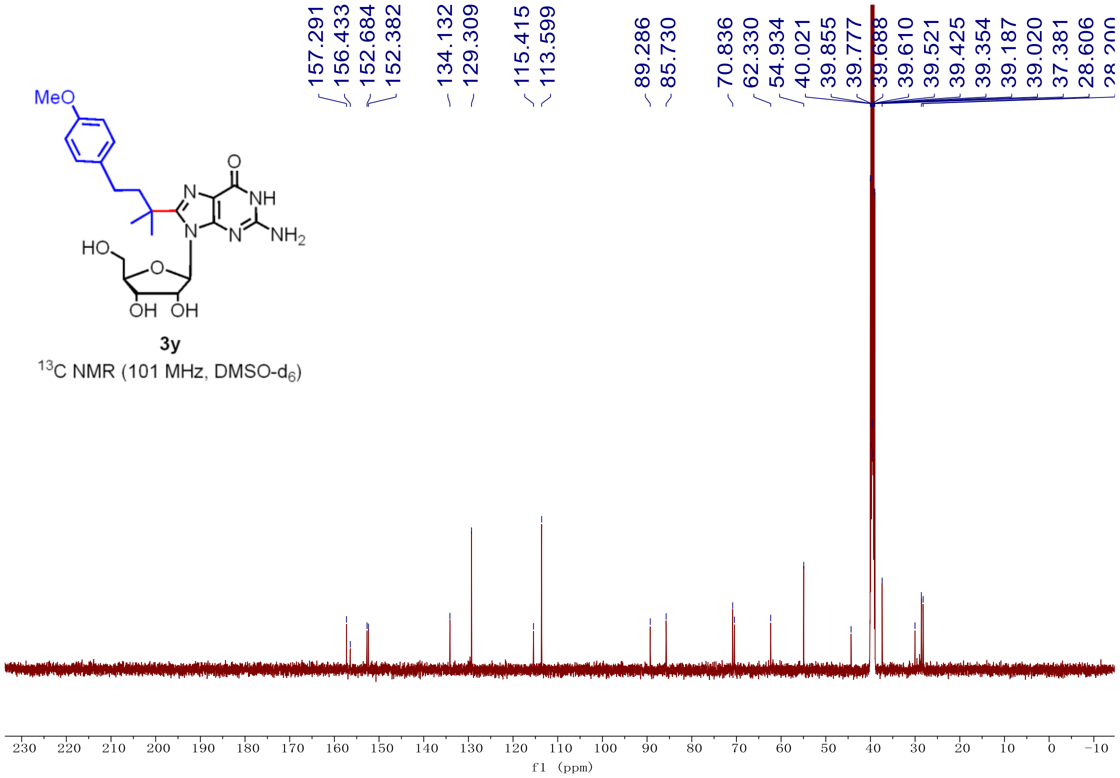

Supplementary Figure 185. ^{13}C NMR spectra of compound **3y**

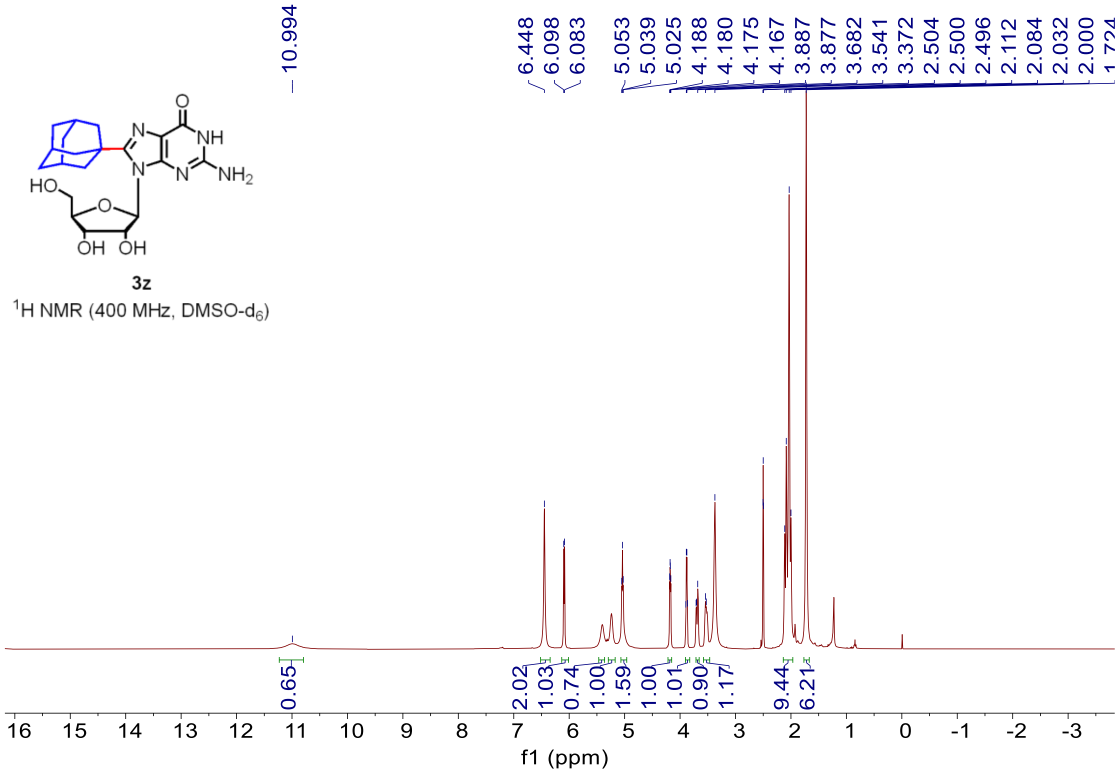

Supplementary Figure 186. ^{1}H NMR spectra of compound **3z**

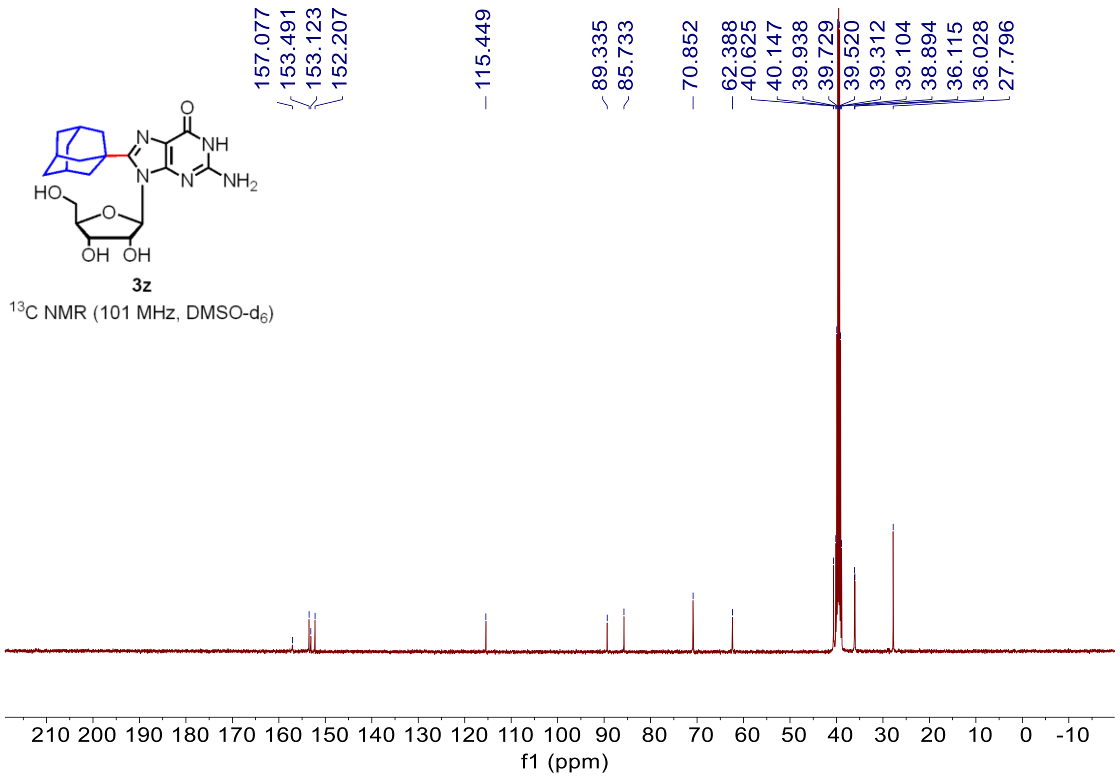

Supplementary Figure 187. ^{13}C NMR spectra of compound **3z**

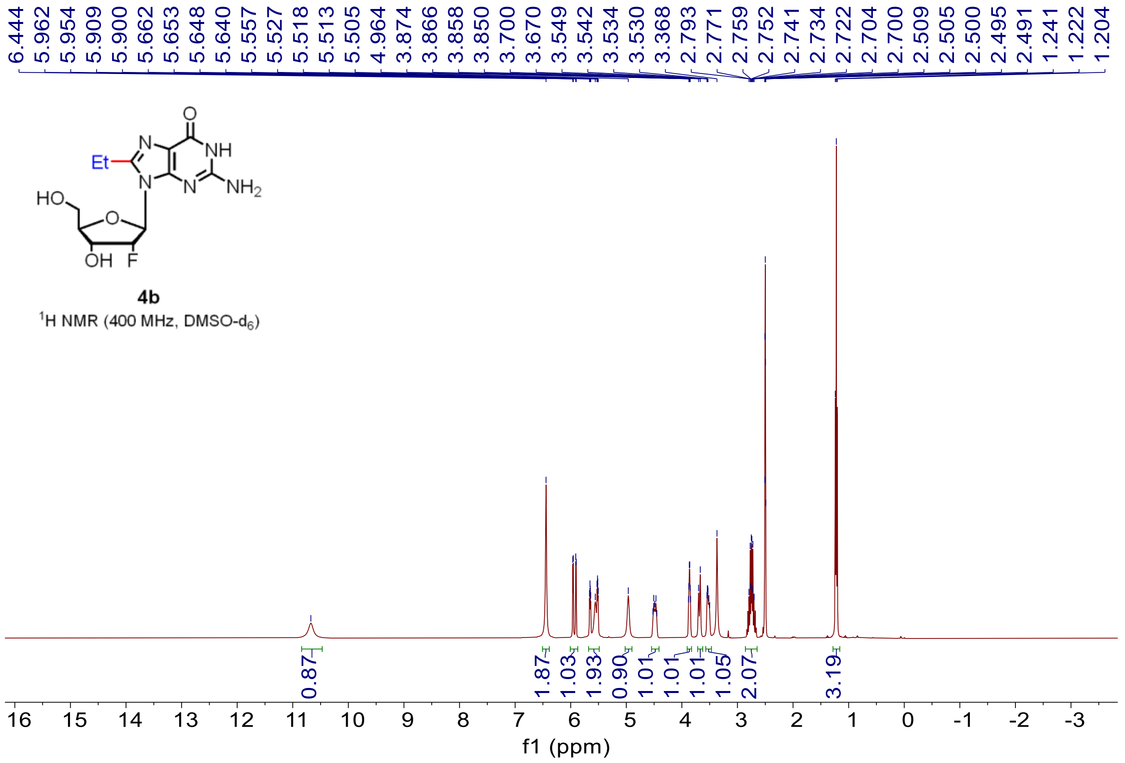

Supplementary Figure 188. ^{1}H NMR spectra of compound **4b**

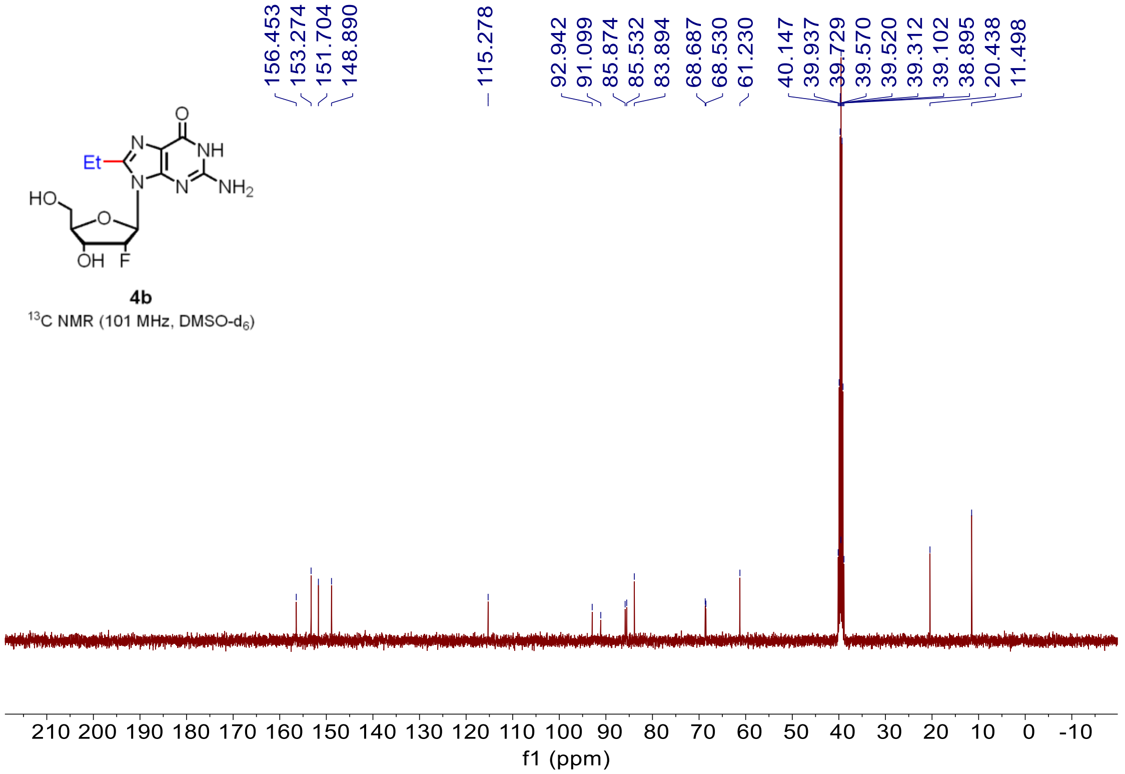

Supplementary Figure 189. ^{13}C NMR spectra of compound **4b**

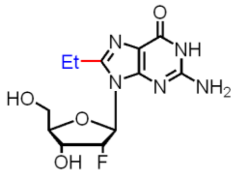

4b

^{19}F NMR (377 MHz, DMSO-d$_6$)

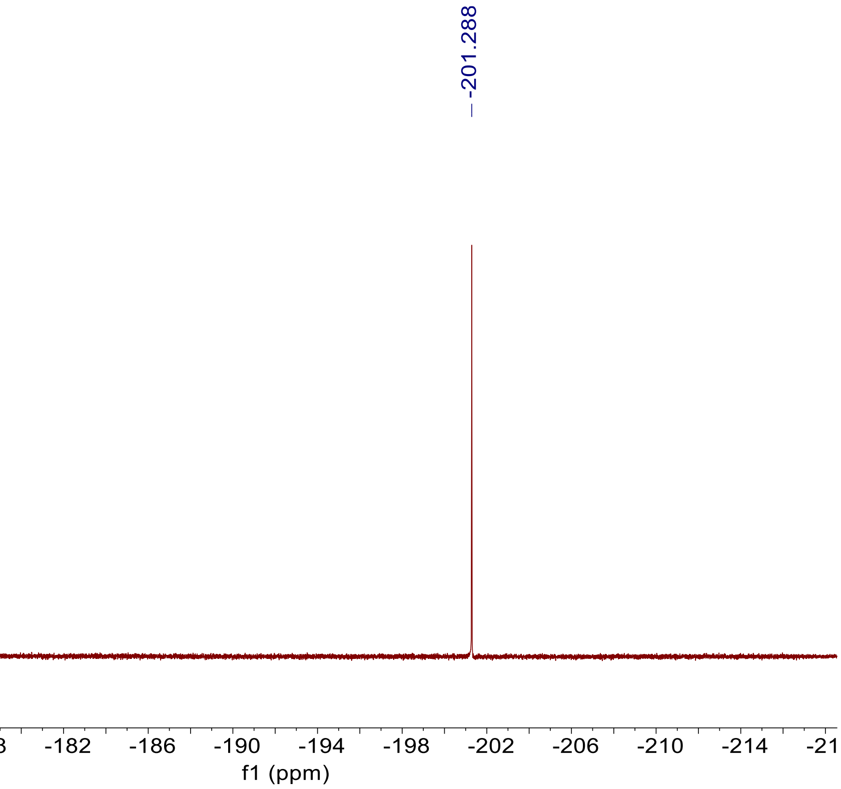

Supplementary Figure 190. ^{19}F NMR spectra of compound **4b**

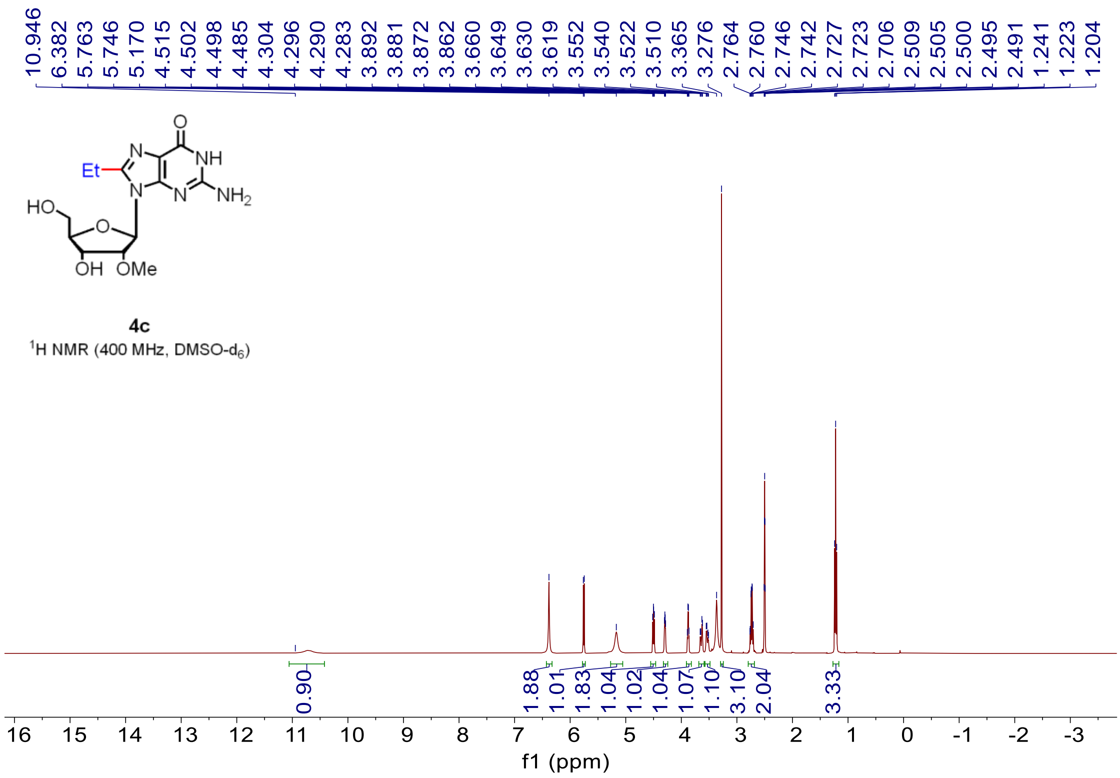

Supplementary Figure 191. ^{1}H NMR spectra of compound **4c**

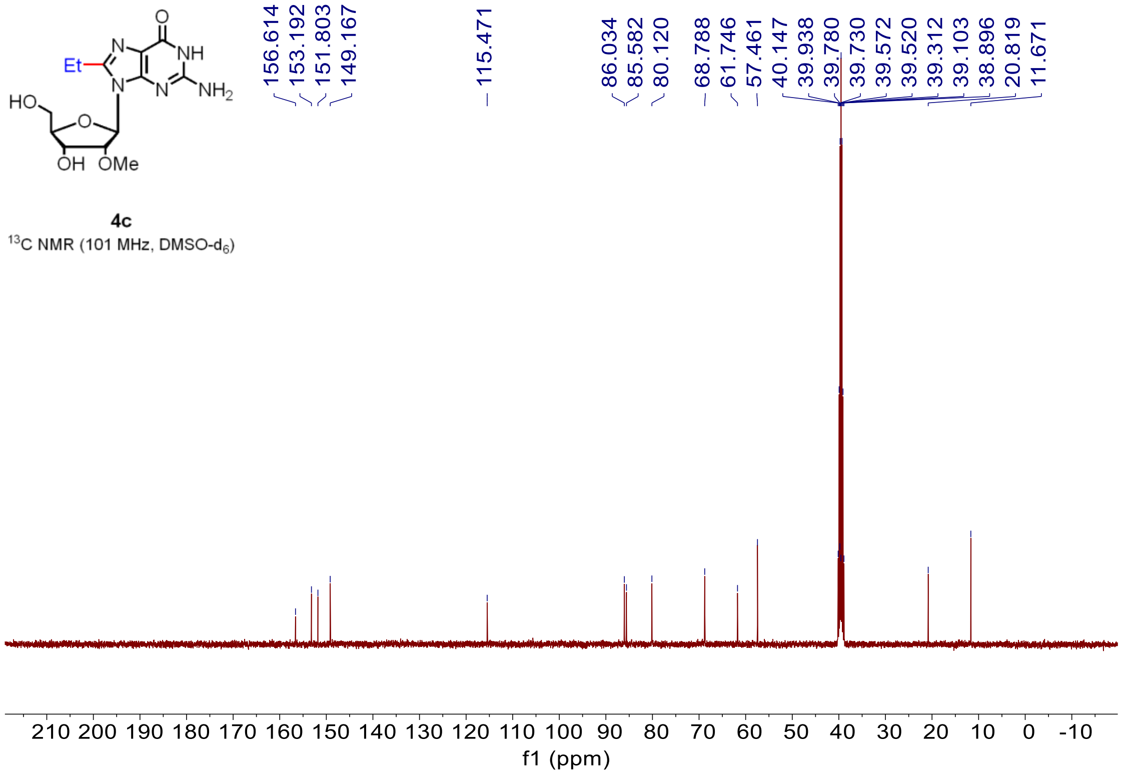

Supplementary Figure 192. ^{13}C NMR spectra of compound **4c**

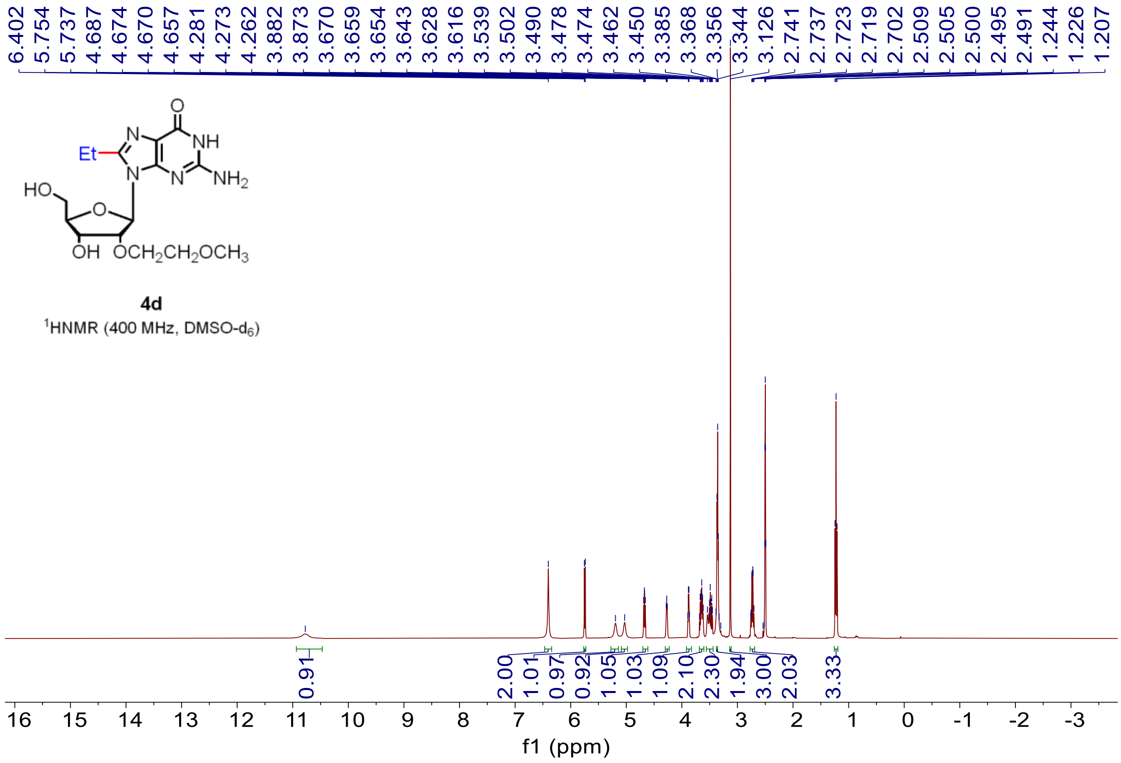

Supplementary Figure 193. ^{1}H NMR spectra of compound **4d**

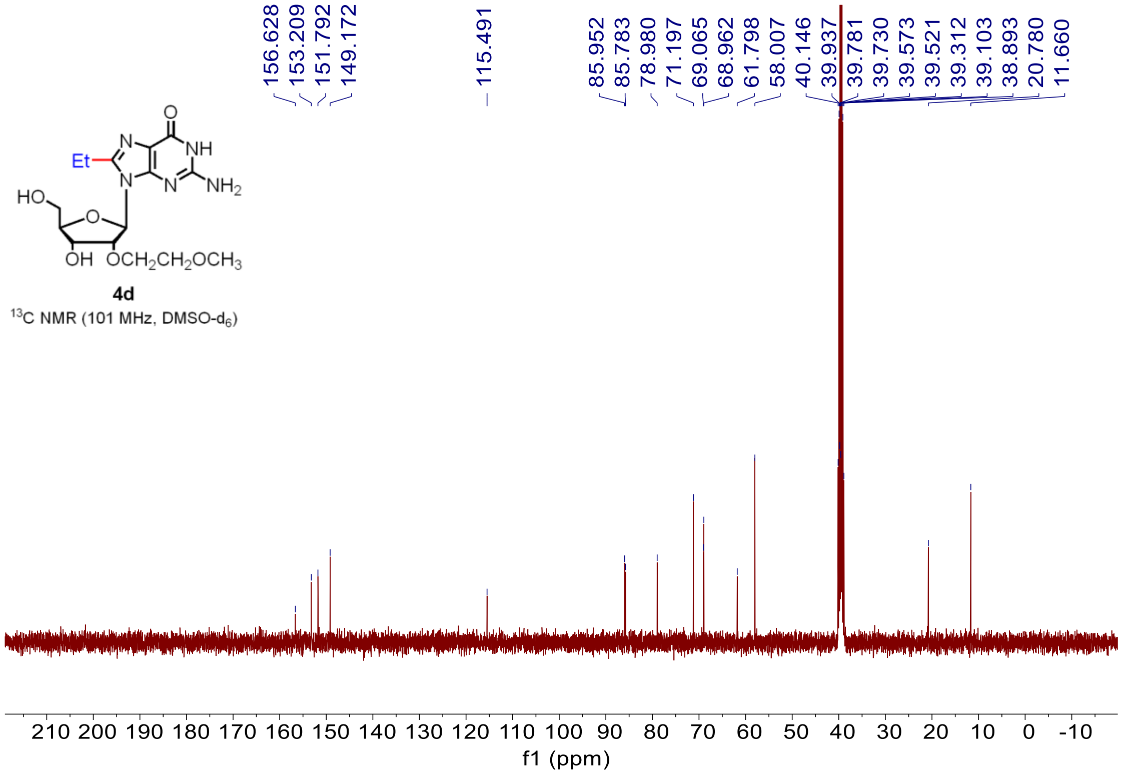

Supplementary Figure 194. ^{13}C NMR spectra of compound **4d**

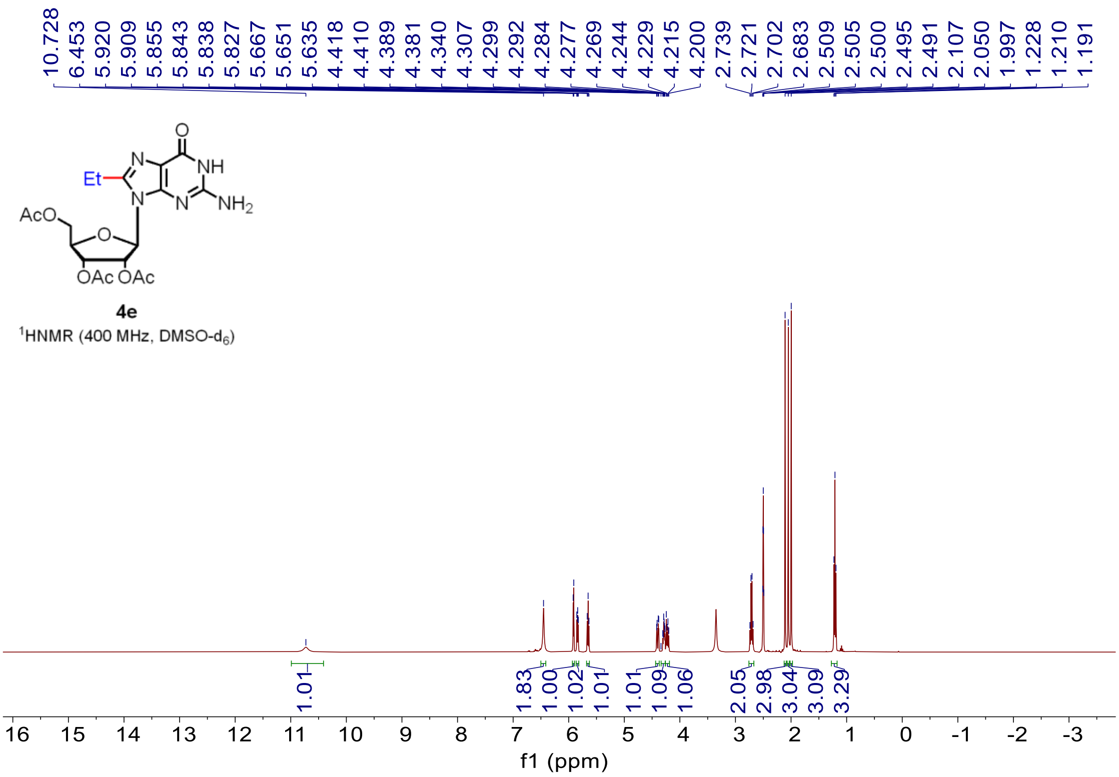

Supplementary Figure 195. ^{1}H NMR spectra of compound **4e**

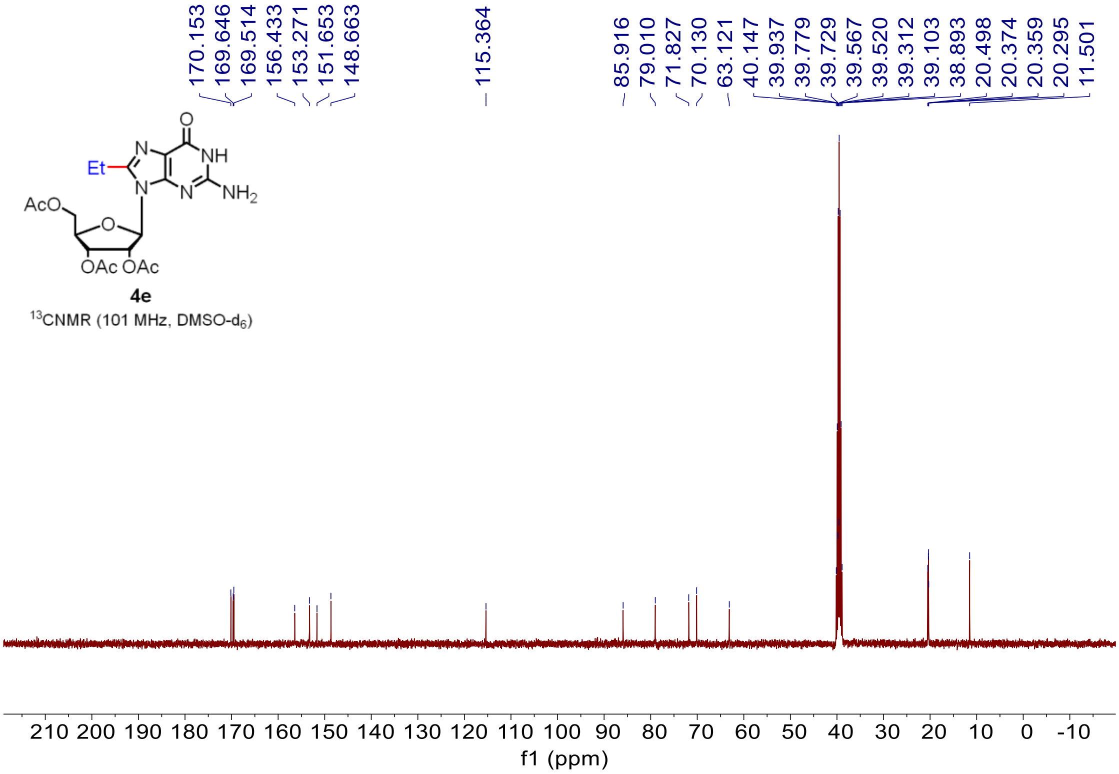

Supplementary Figure 196. ^{13}C NMR spectra of compound **4e**

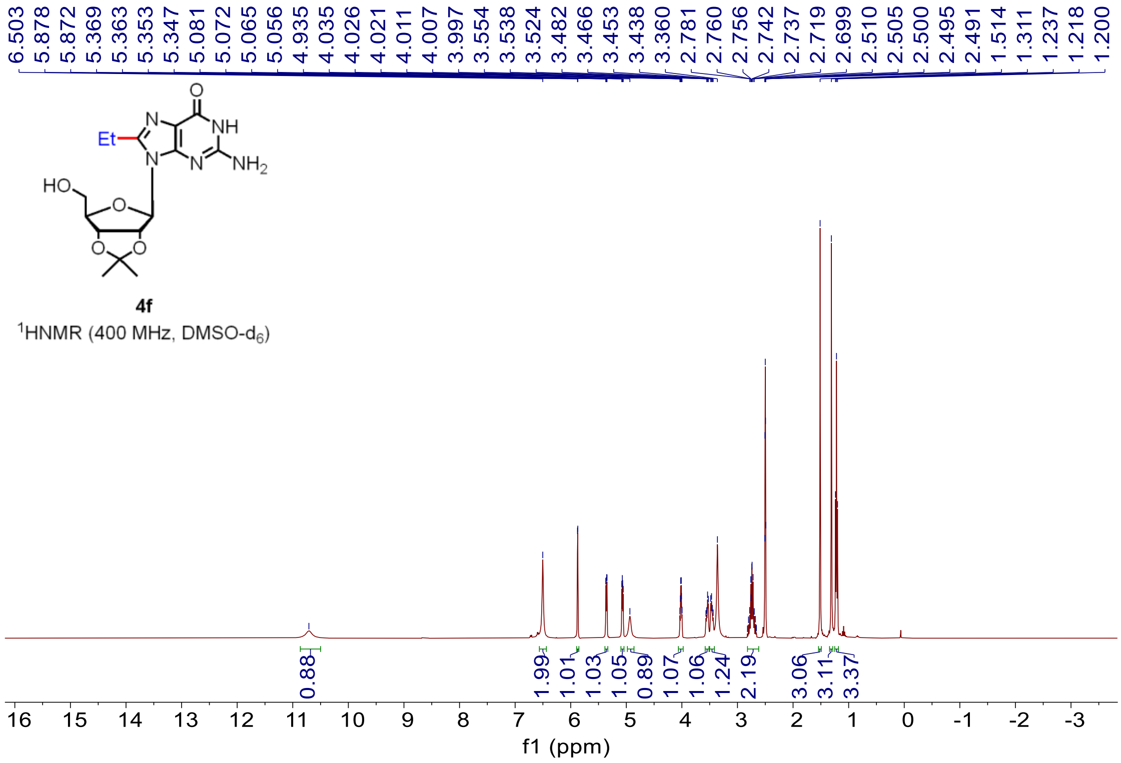

Supplementary Figure 197. ^{1}H NMR spectra of compound **4f**

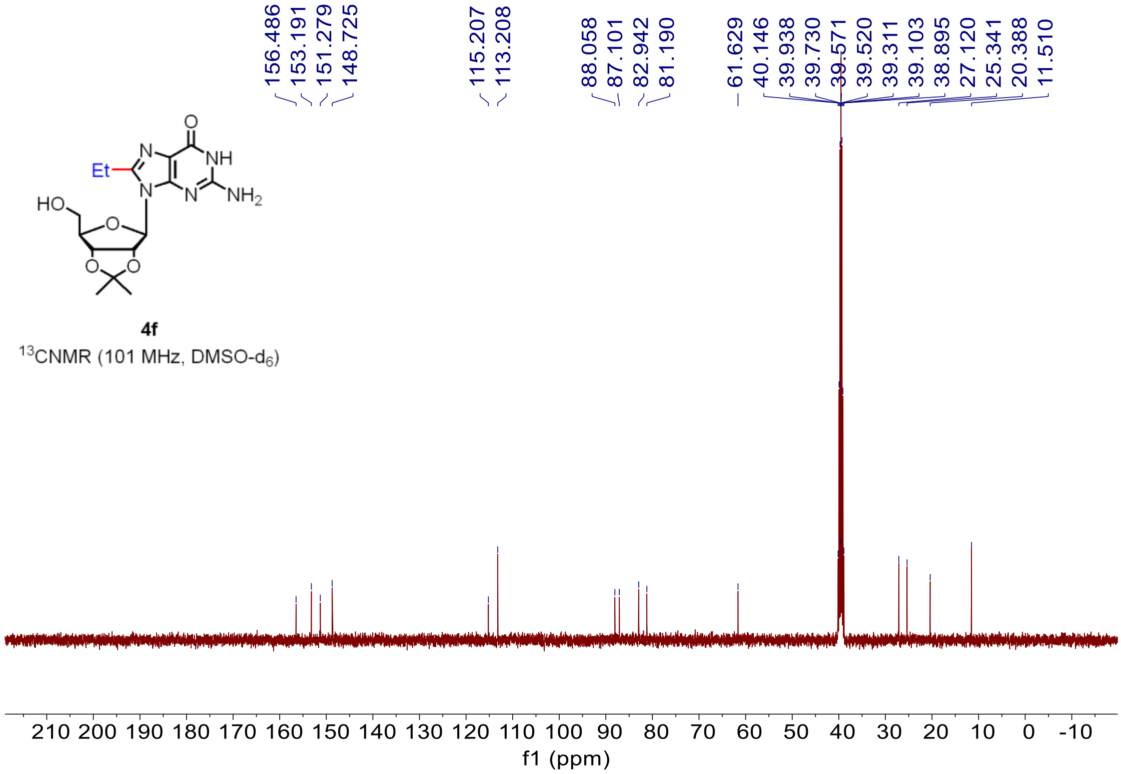

Supplementary Figure 198. ^{13}C NMR spectra of compound **4f**

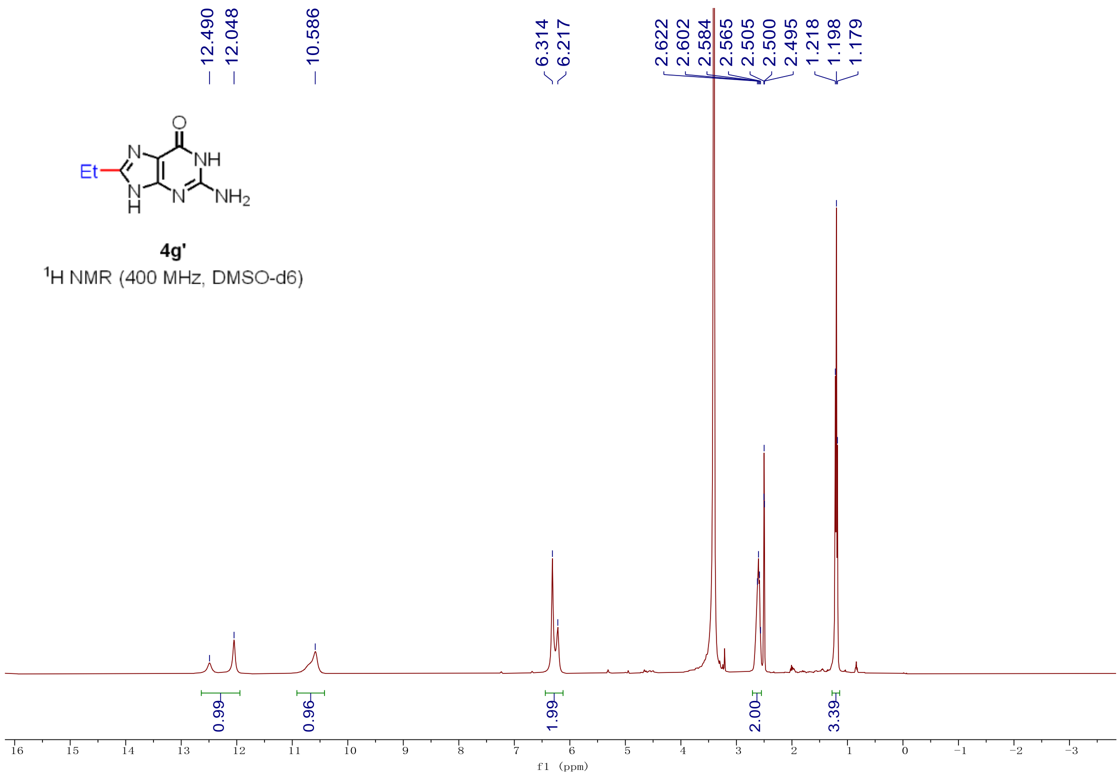

Supplementary Figure 199. ^{1}H NMR spectra of compound **4g'**

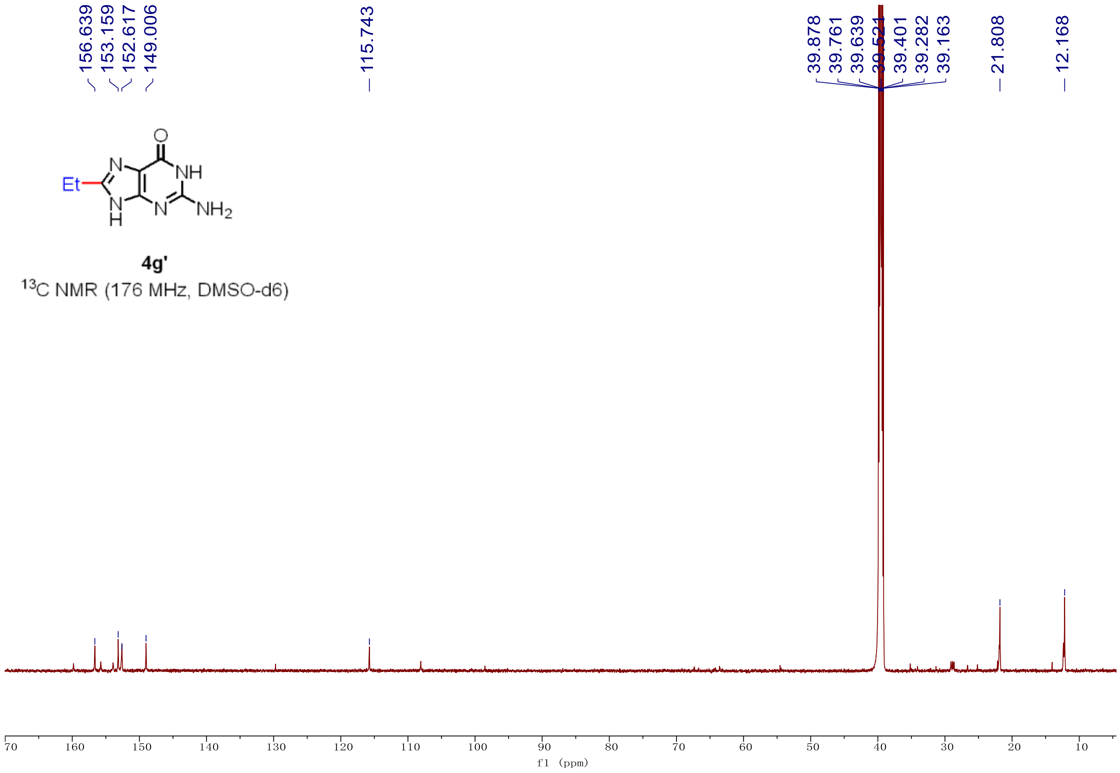

Supplementary Figure 200. ^{13}C NMR spectra of compound **4g'**

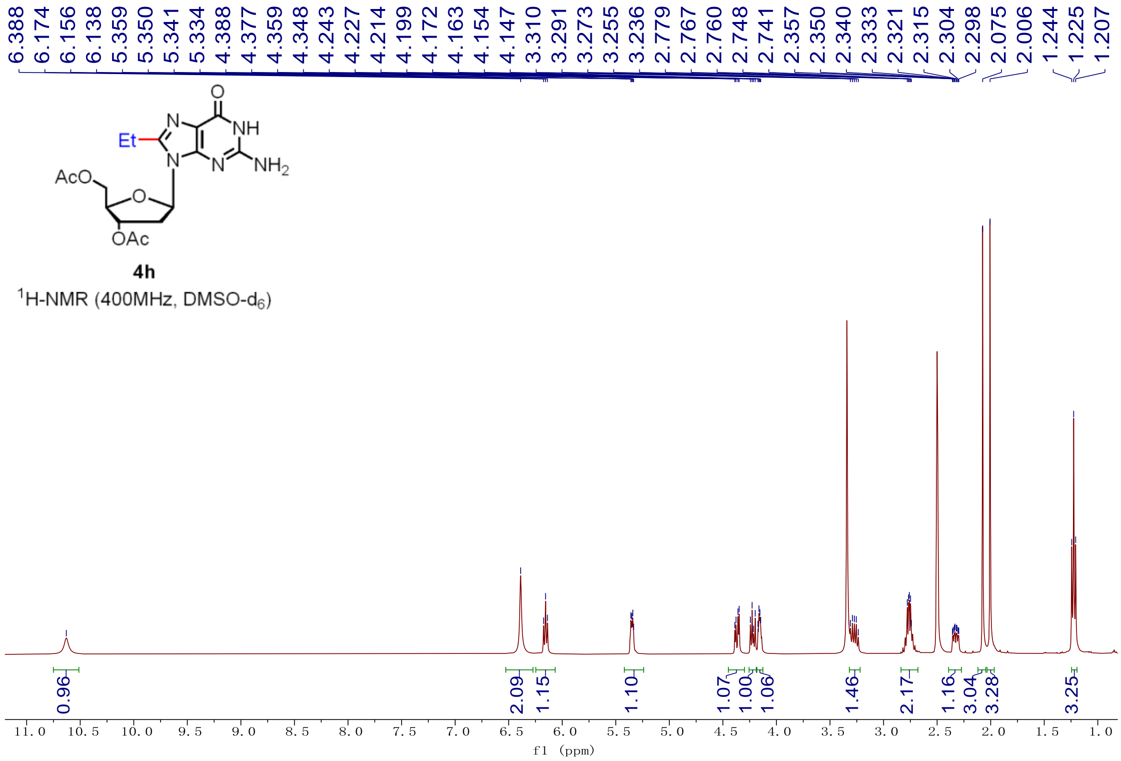

Supplementary Figure 201. ^{1}H NMR spectra of compound **4h**

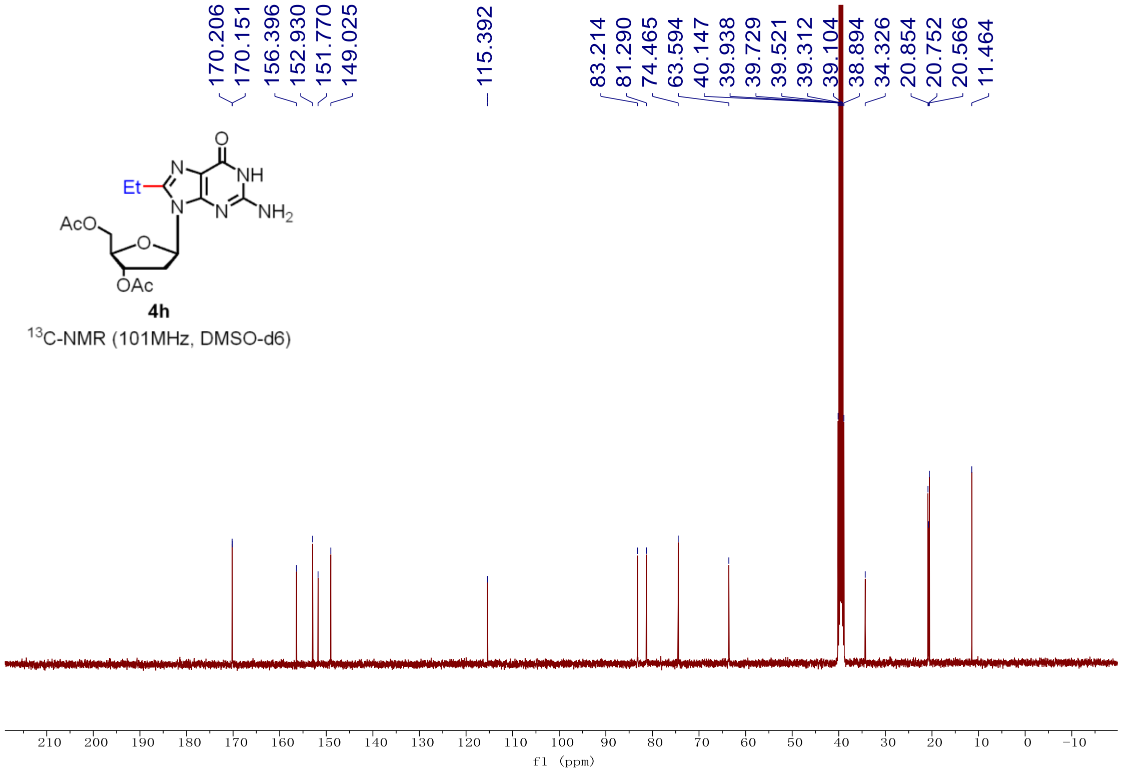

Supplementary Figure 202. ^{13}C NMR spectra of compound **4h**

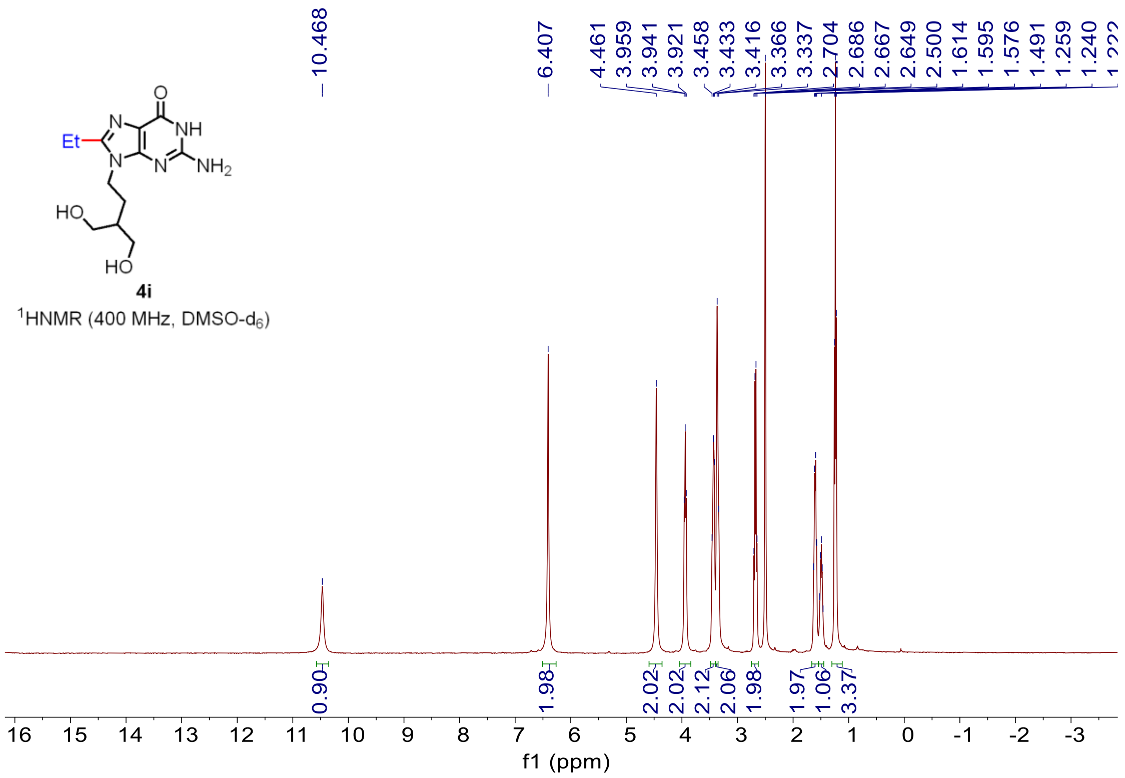

Supplementary Figure 203. ^{1}H NMR spectra of compound **4i**

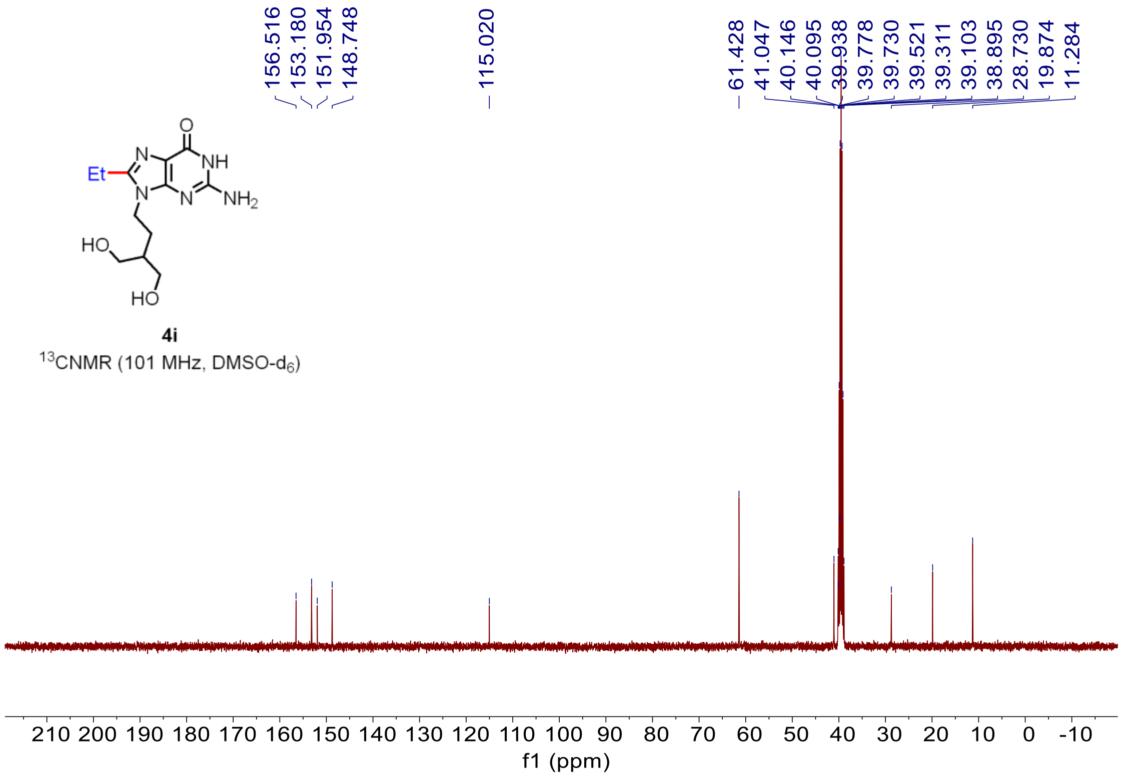

Supplementary Figure 204. ^{13}C NMR spectra of compound **4i**

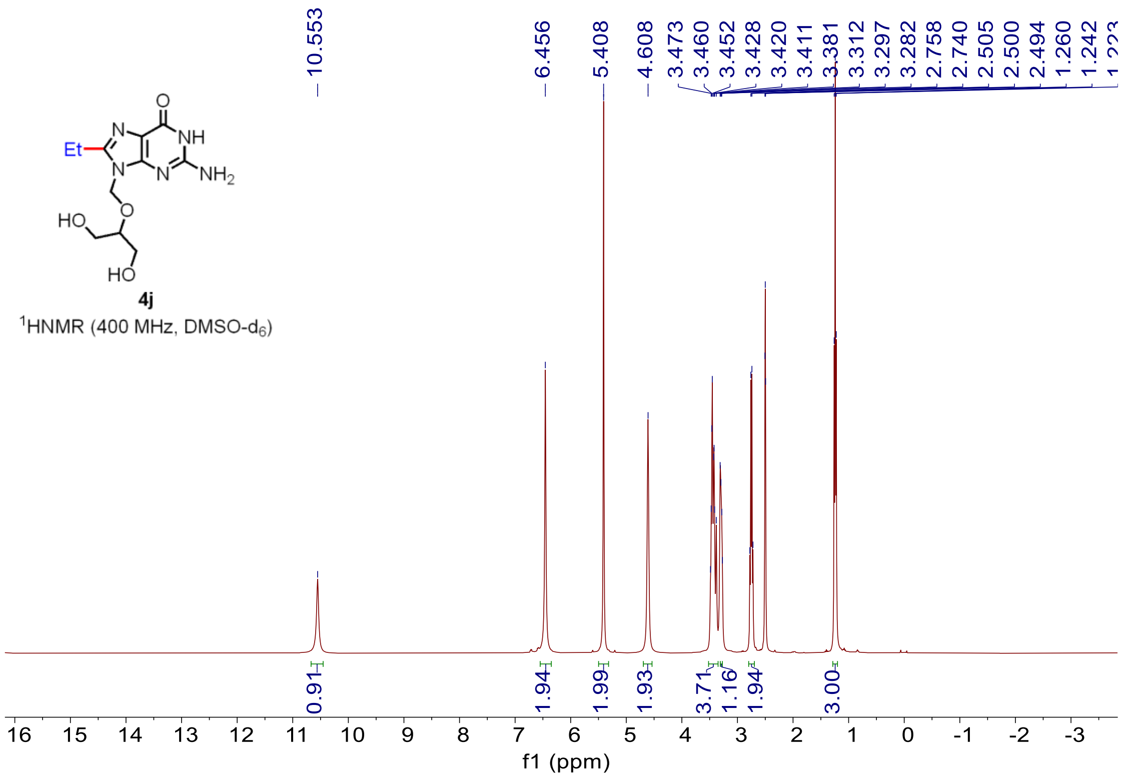

Supplementary Figure 205. ^{1}H NMR spectra of compound **4j**

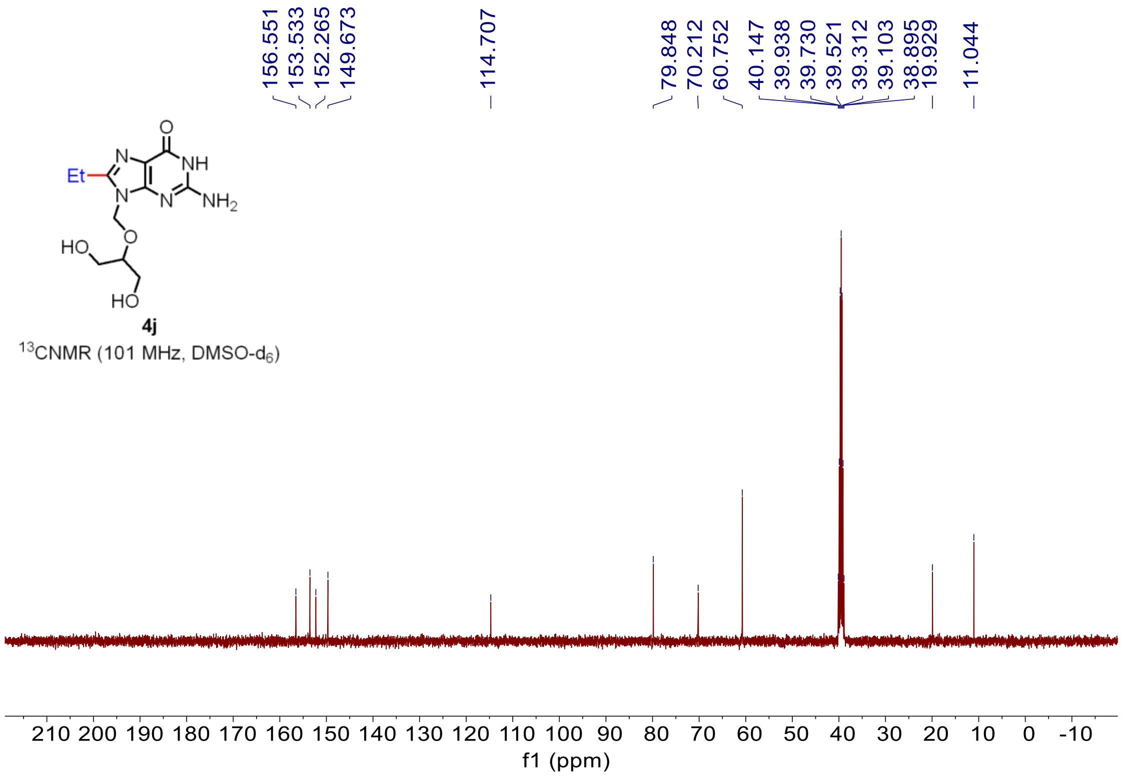

Supplementary Figure 206. ^{13}C NMR spectra of compound **4j**

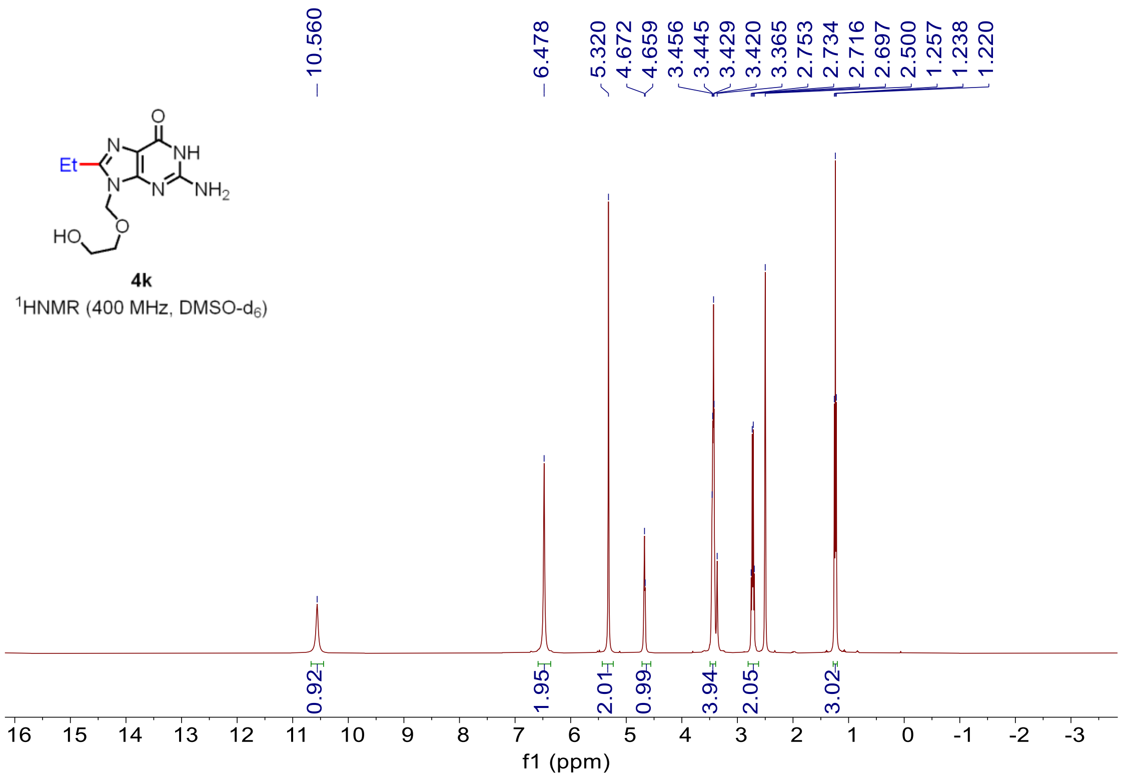

Supplementary Figure 207. ^{1}H NMR spectra of compound **4k**

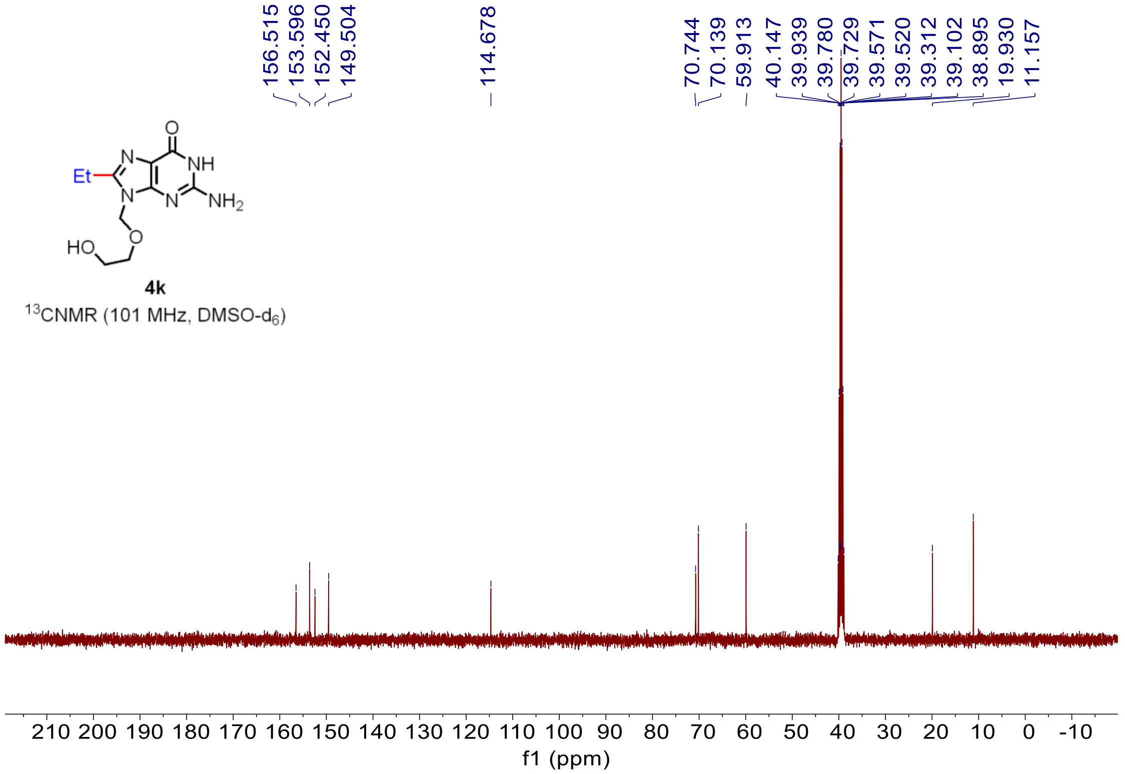

Supplementary Figure 208. ^{13}C NMR spectra of compound **4k**

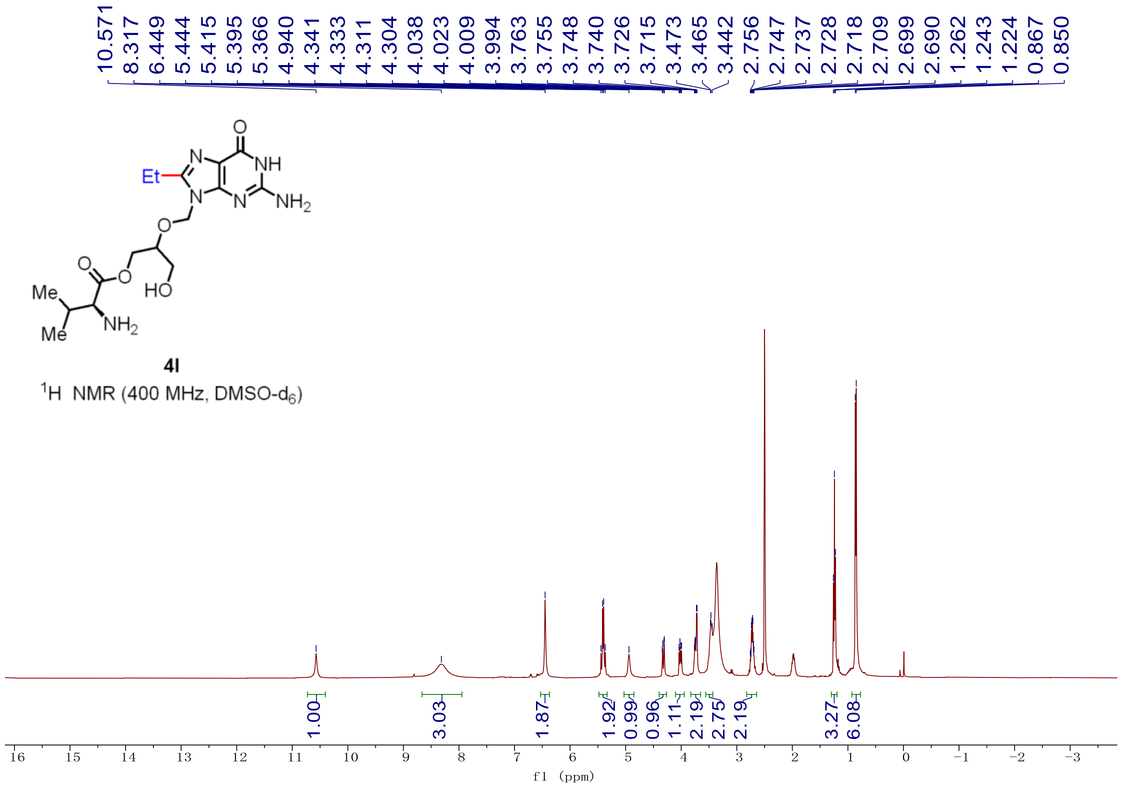

Supplementary Figure 209. ^{1}H NMR spectra of compound **4l**

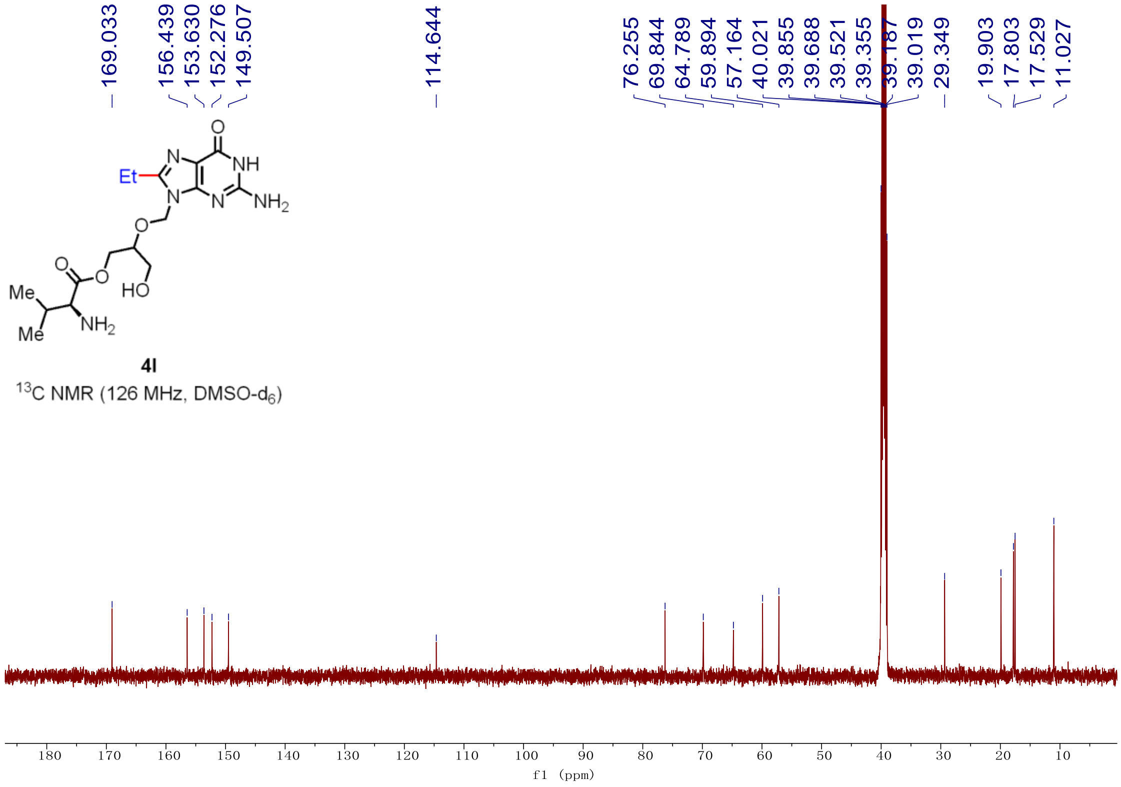

Supplementary Figure 210. ^{13}C NMR spectra of compound **4l**

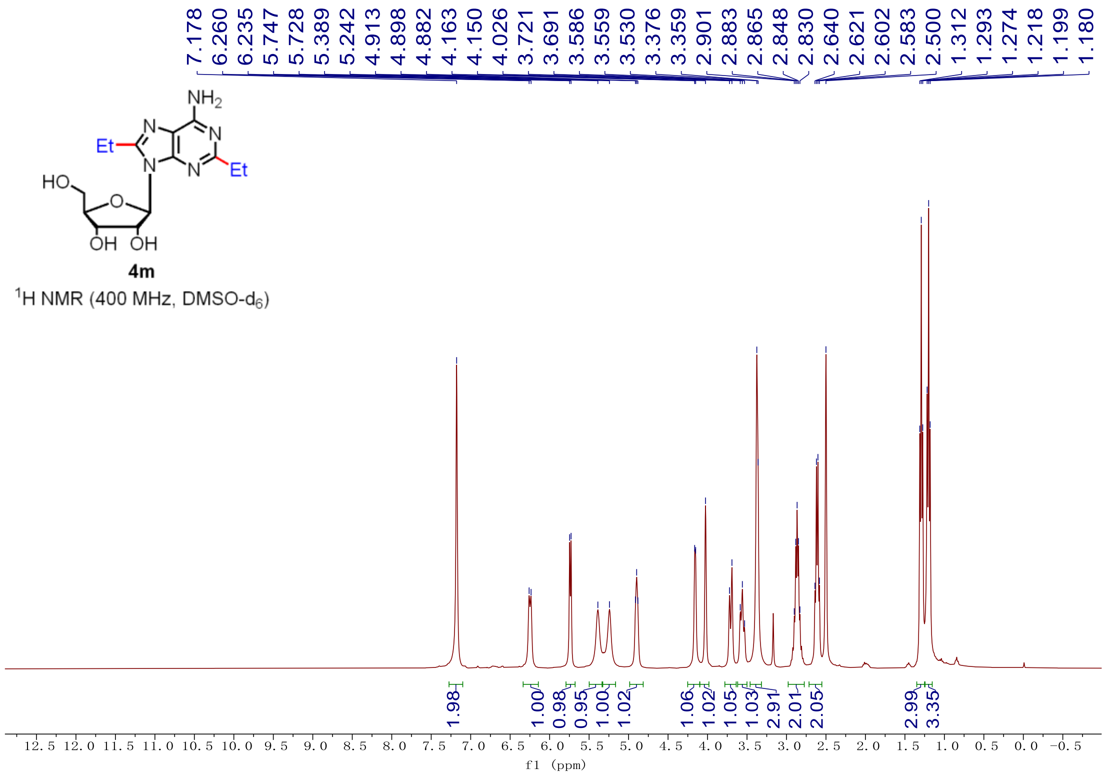

Supplementary Figure 211. ^{1}H NMR spectra of compound **4m**

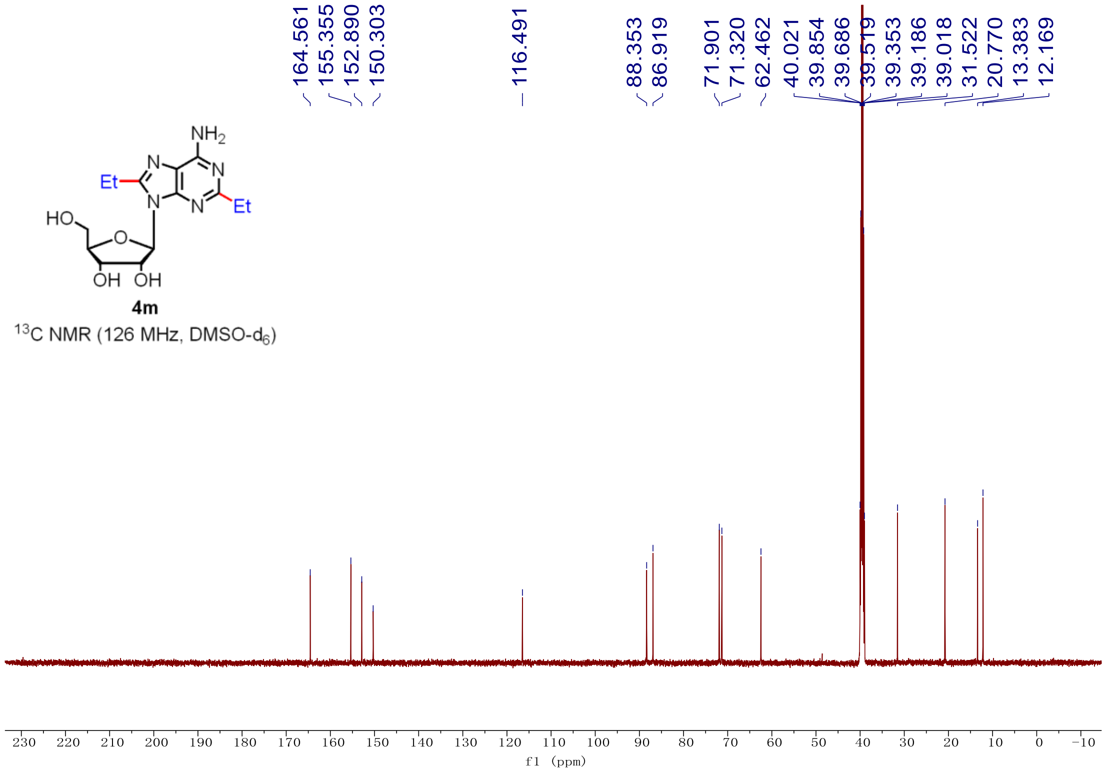

Supplementary Figure 212. ^{13}C NMR spectra of compound **4m**

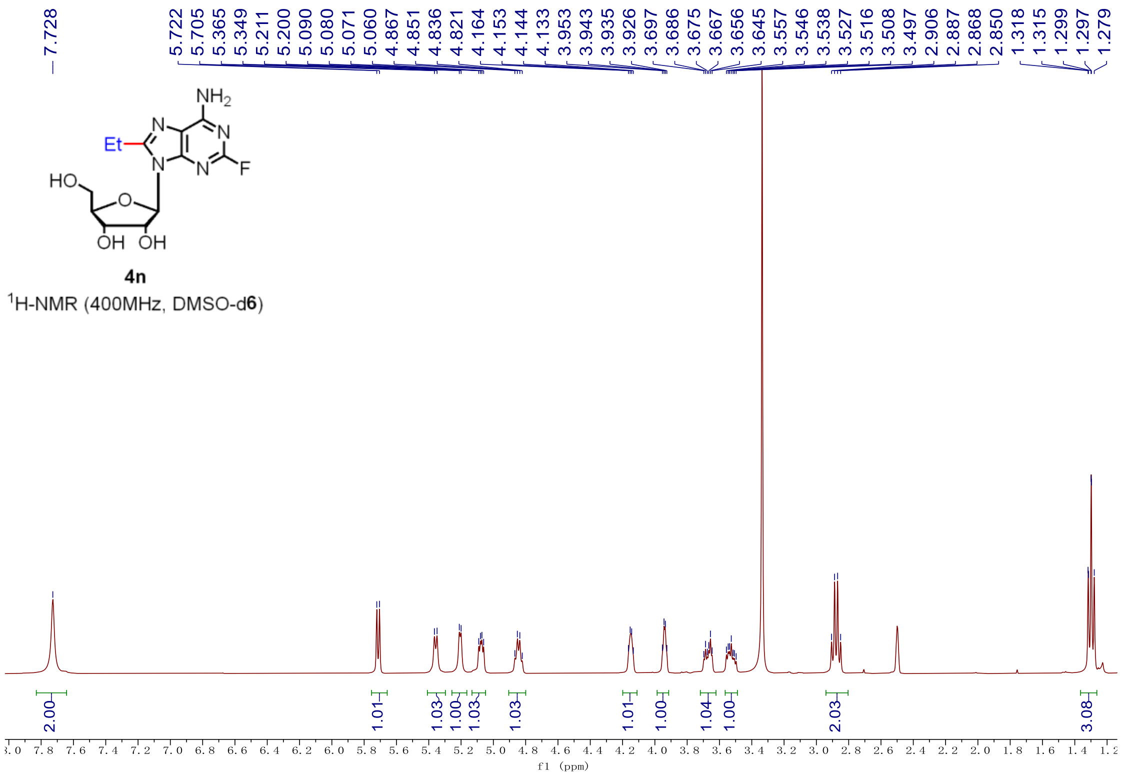

Supplementary Figure 213. ^{1}H NMR spectra of compound **4n**

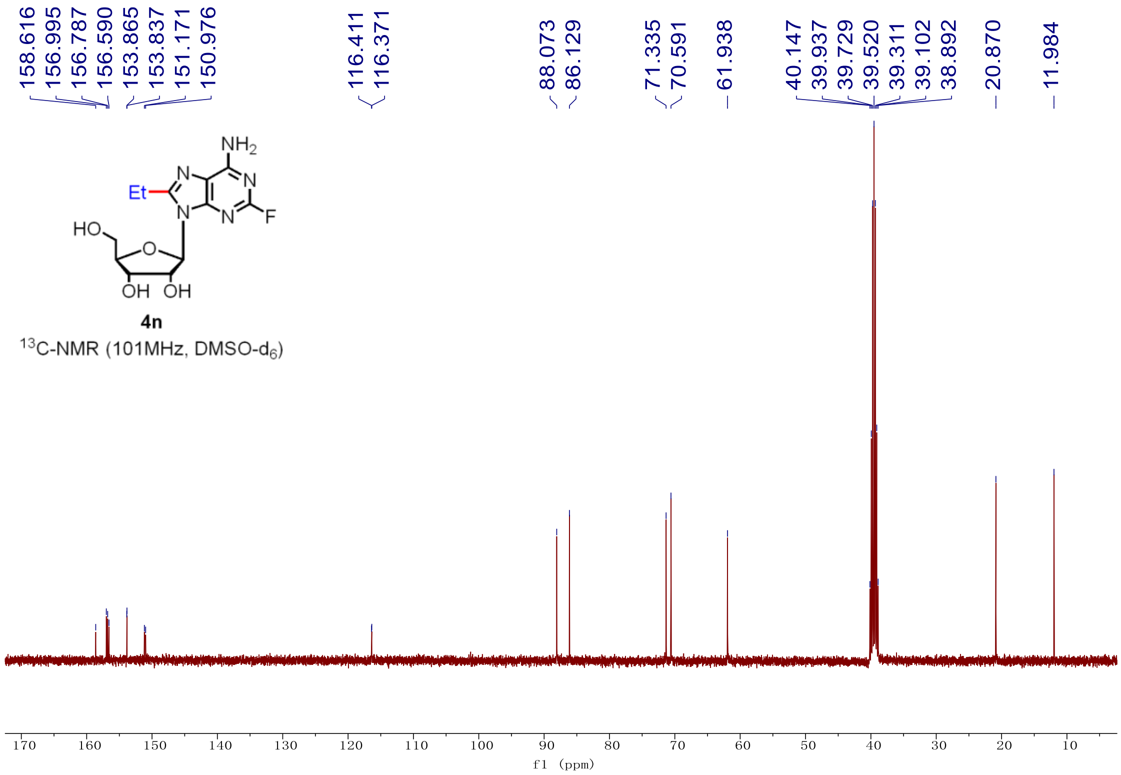

Supplementary Figure 214. ^{13}C NMR spectra of compound **4n**

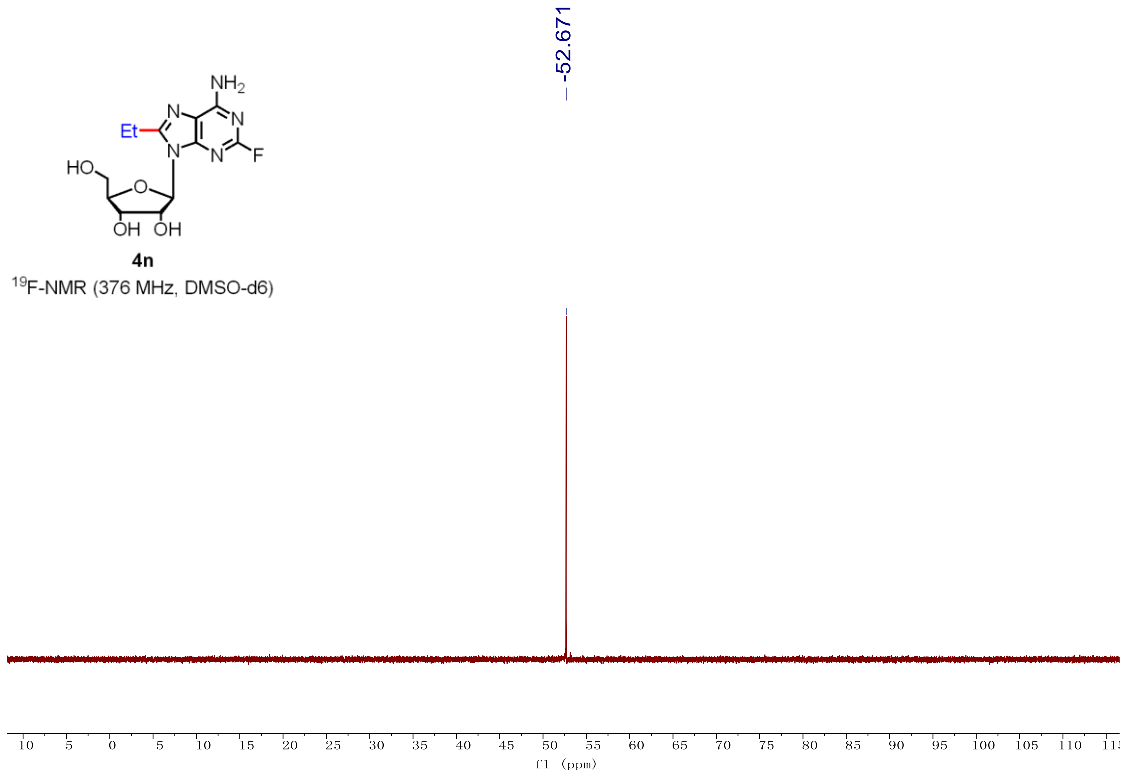

Supplementary Figure 215. ^{19}F NMR spectra of compound **4n**

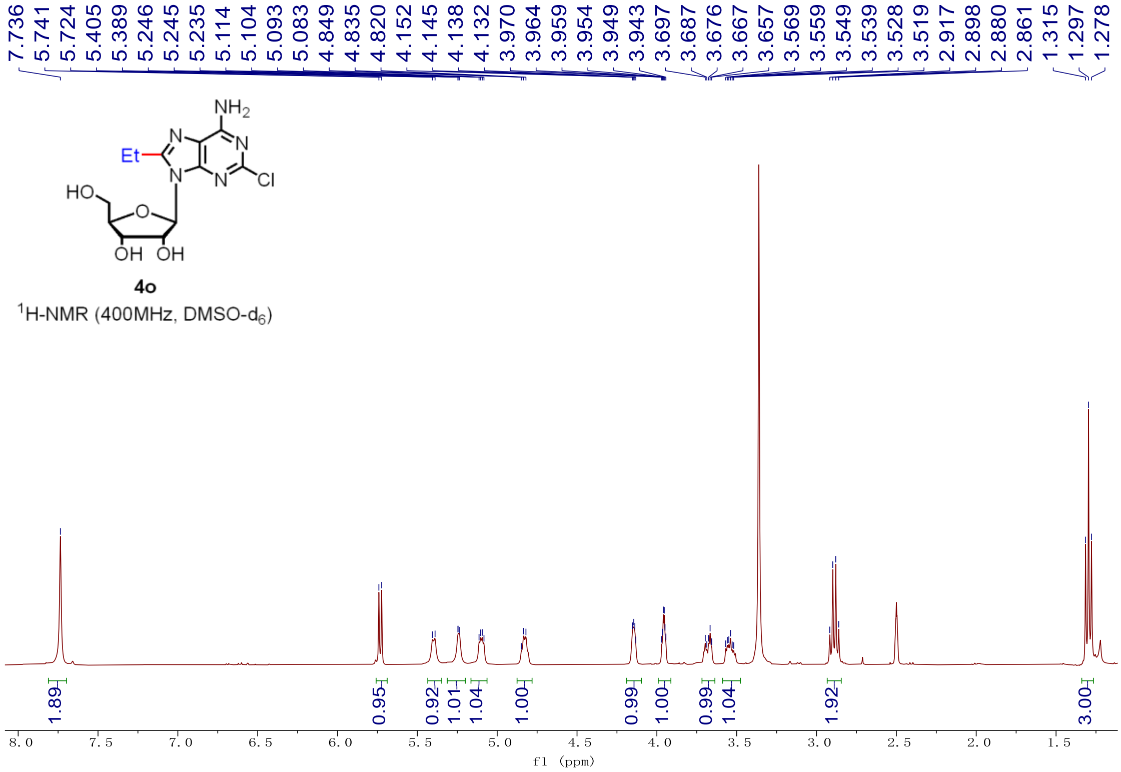

Supplementary Figure 216. ^{1}H NMR spectra of compound **4o**

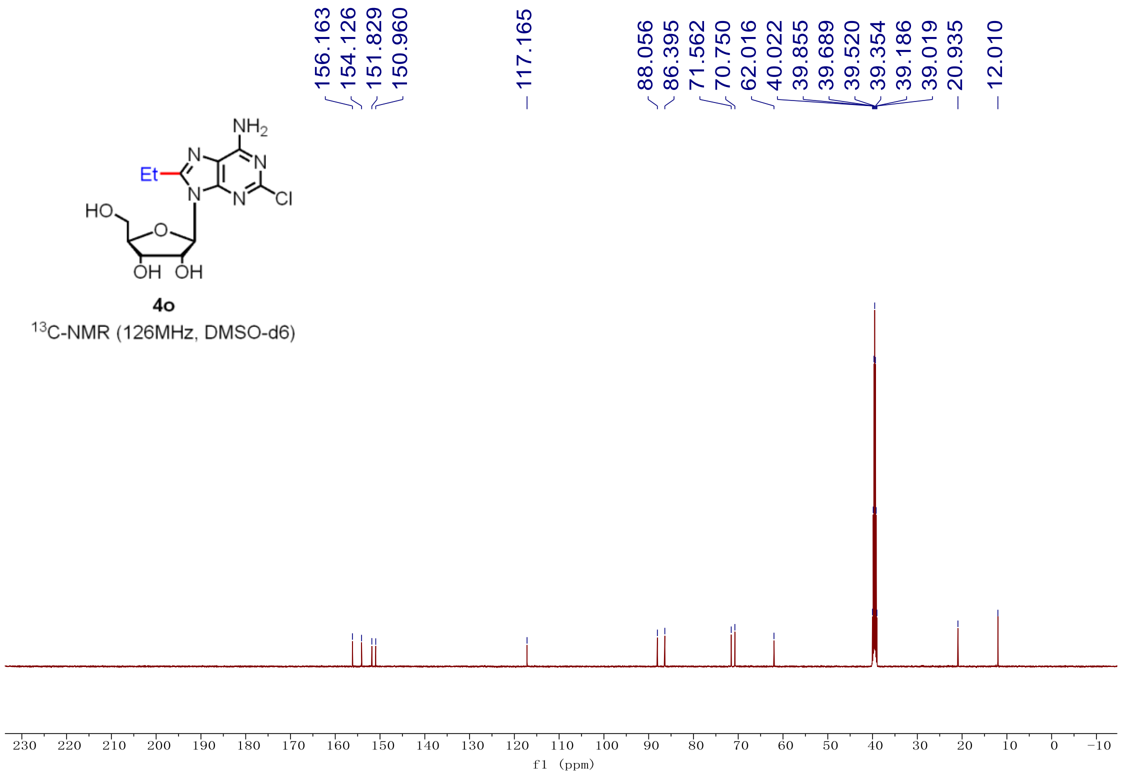

Supplementary Figure 217. ^{13}C NMR spectra of compound **4o**

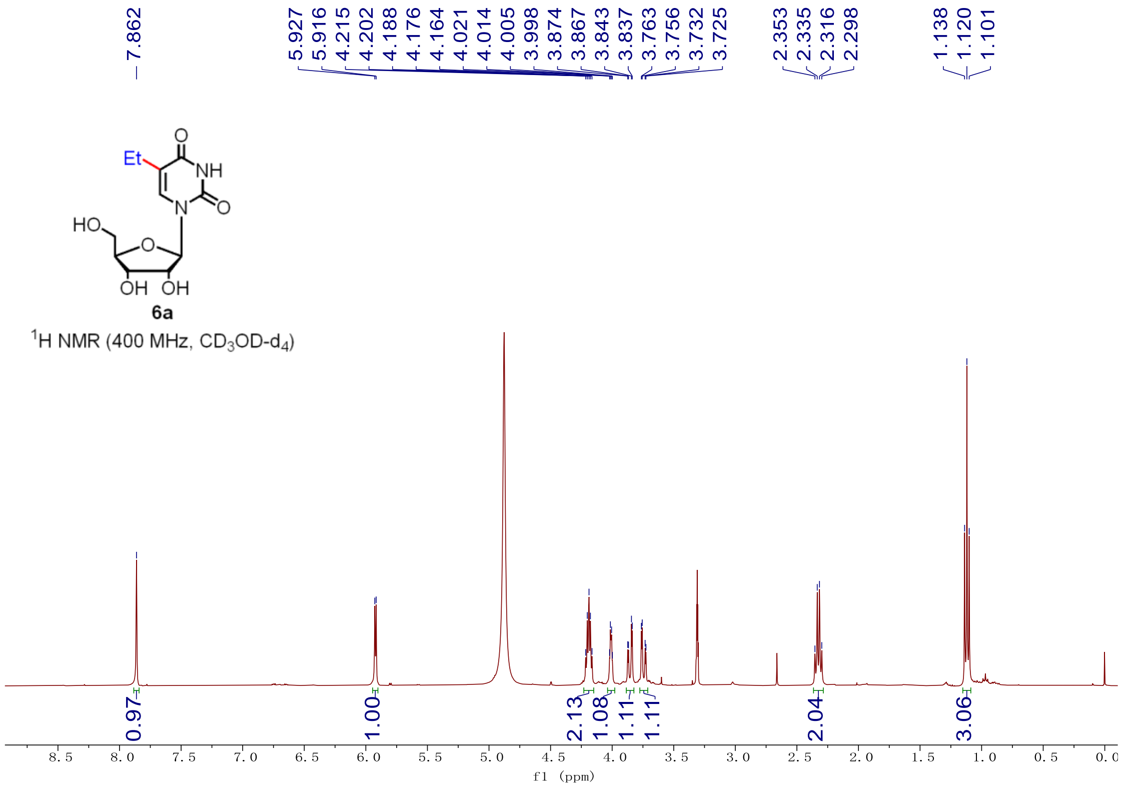

Supplementary Figure 218. ^{1}H NMR spectra of compound **6a**

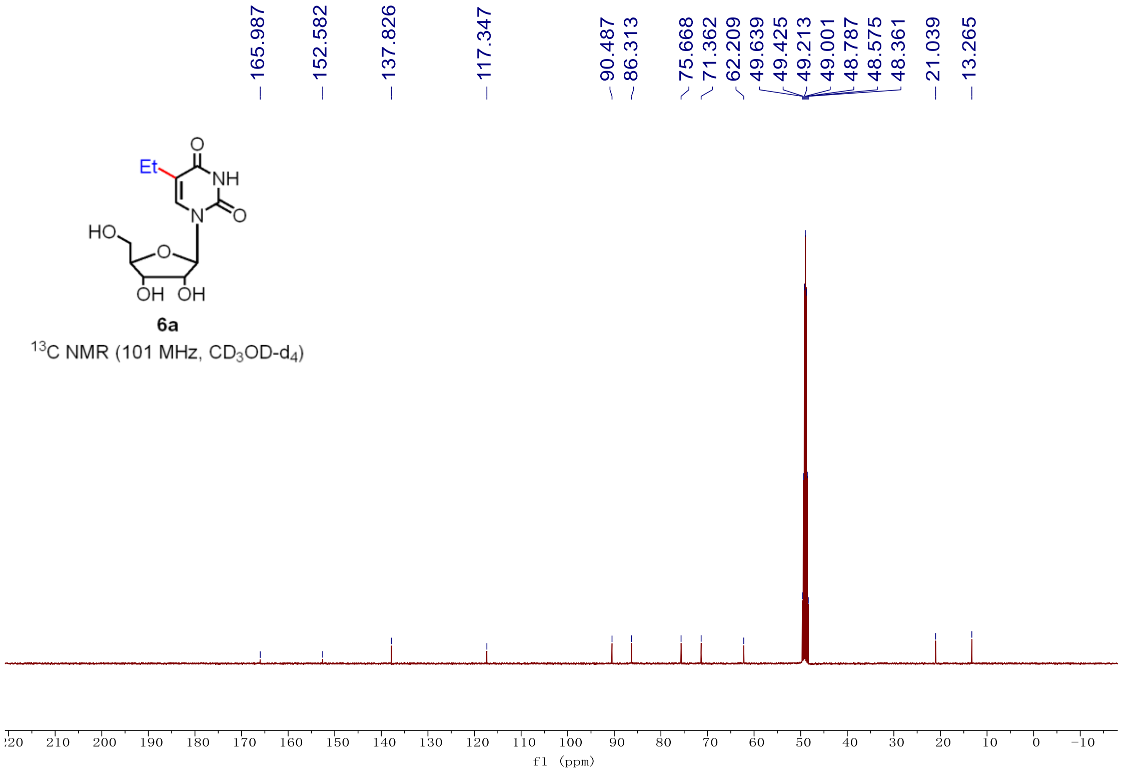

Supplementary Figure 219. ^{13}C NMR spectra of compound **6a**

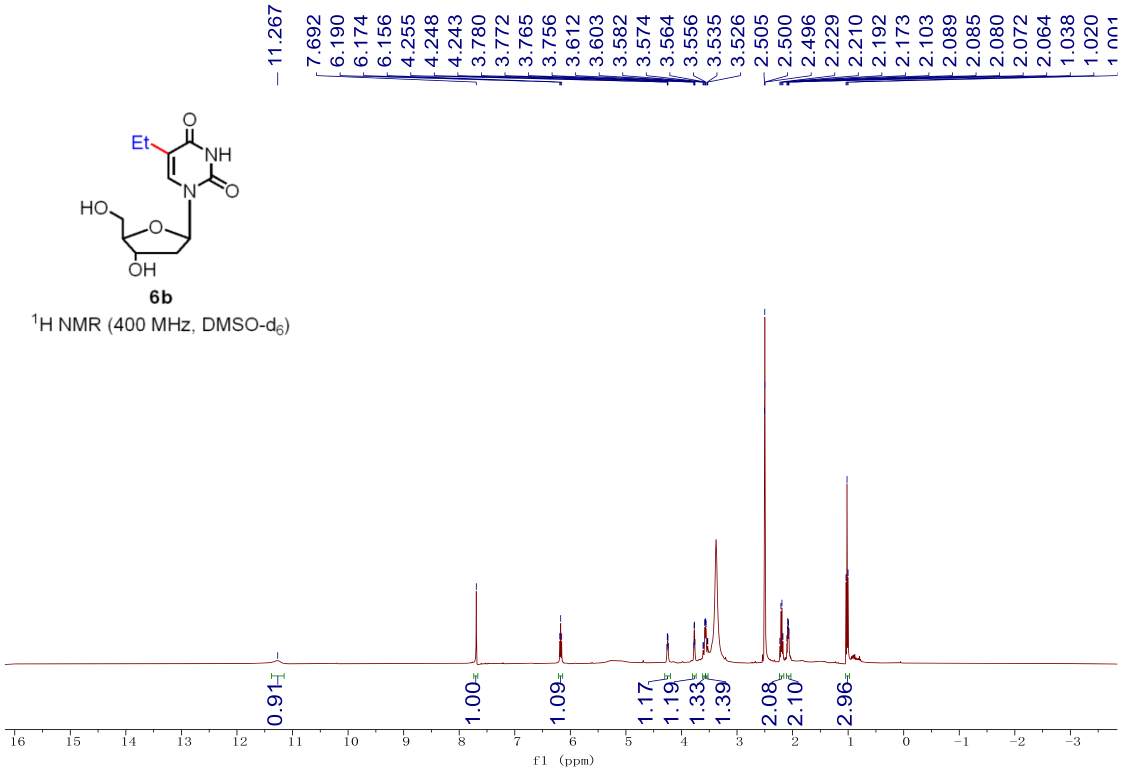

Supplementary Figure 220. ^{1}H NMR spectra of compound **6b**

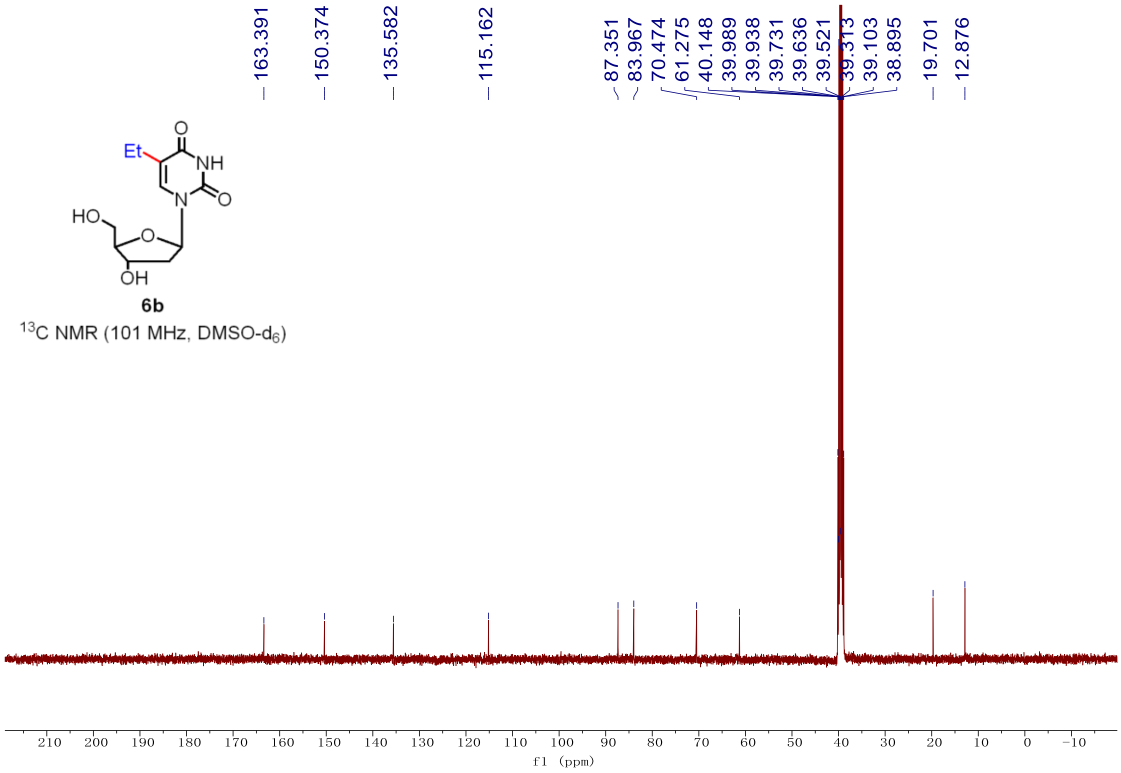

Supplementary Figure 221. ^{13}C NMR spectra of compound **6b**

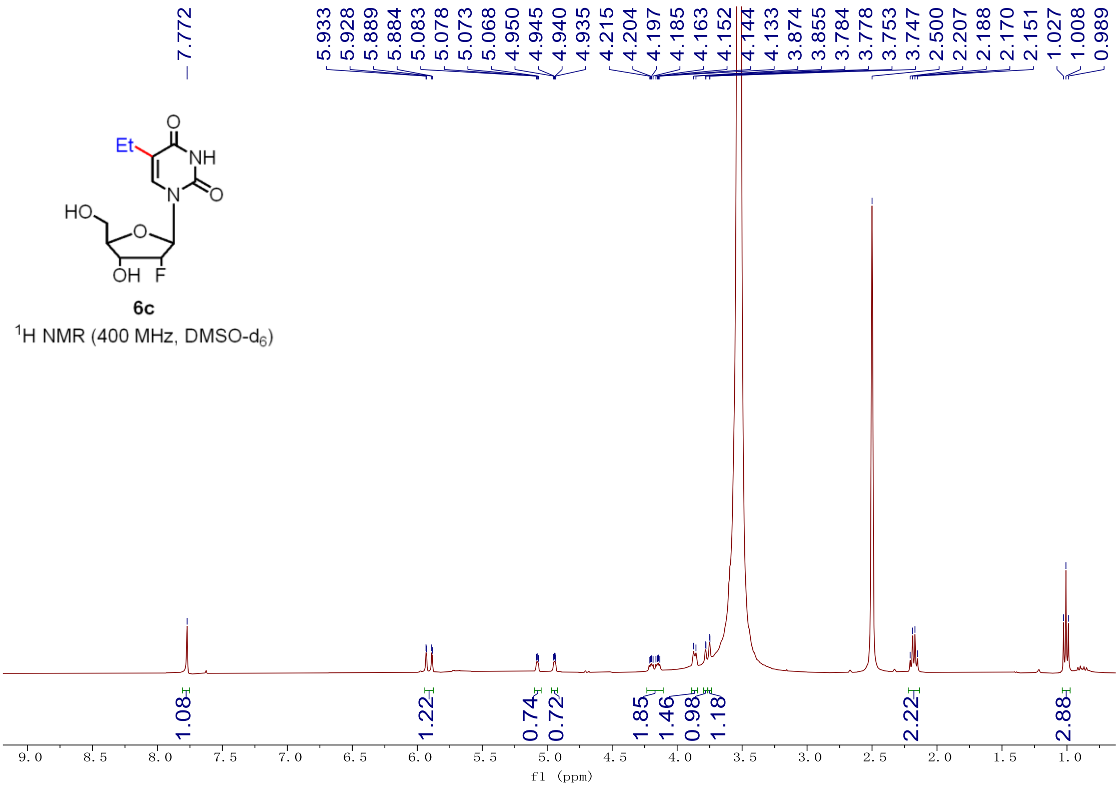

Supplementary Figure 222. ^{1}H NMR spectra of compound **6c**

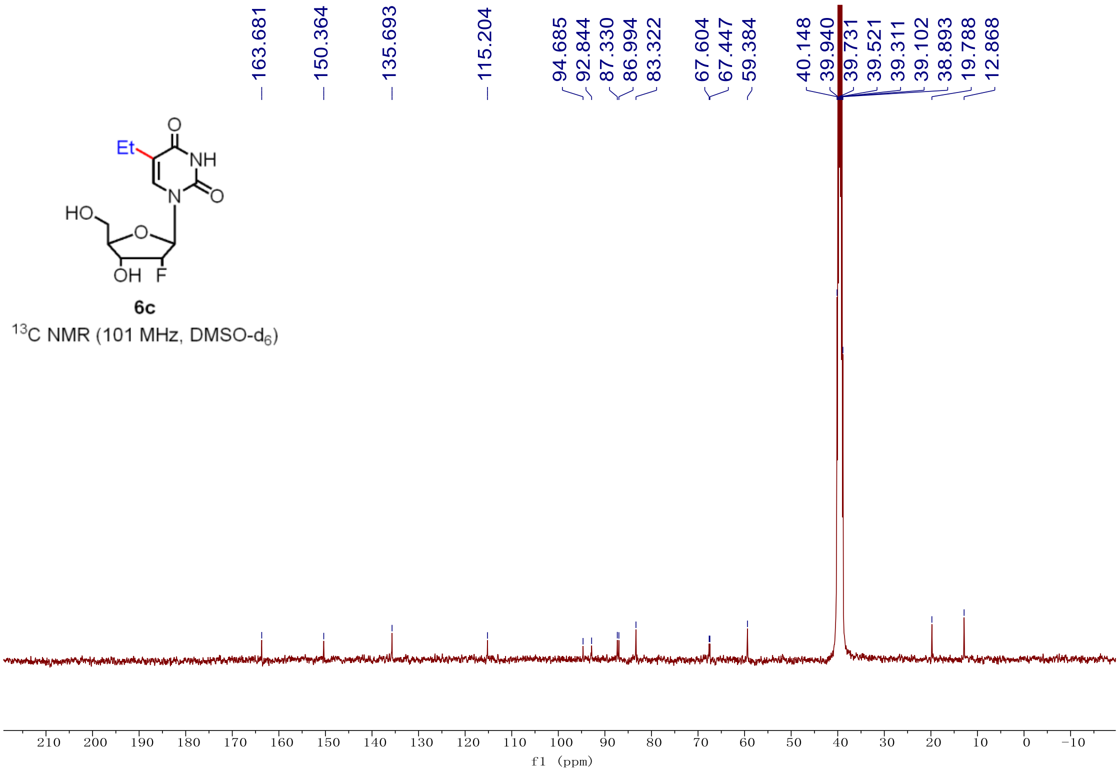

Supplementary Figure 223. ^{13}C NMR spectra of compound **6c**

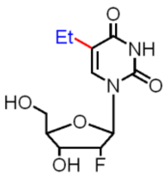

6c

^{19}F NMR (376 MHz, DMSO-d_6)

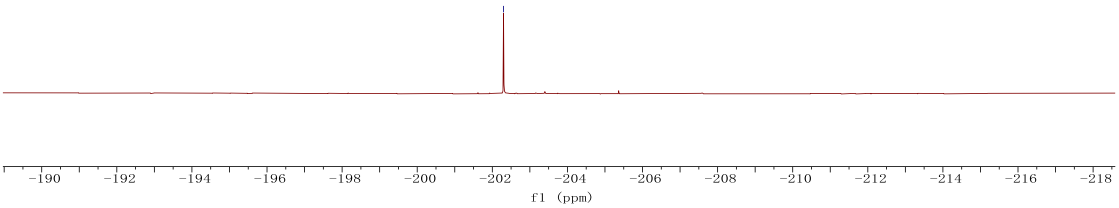

Supplementary Figure 224. ^{19}F NMR spectra of compound **6c**

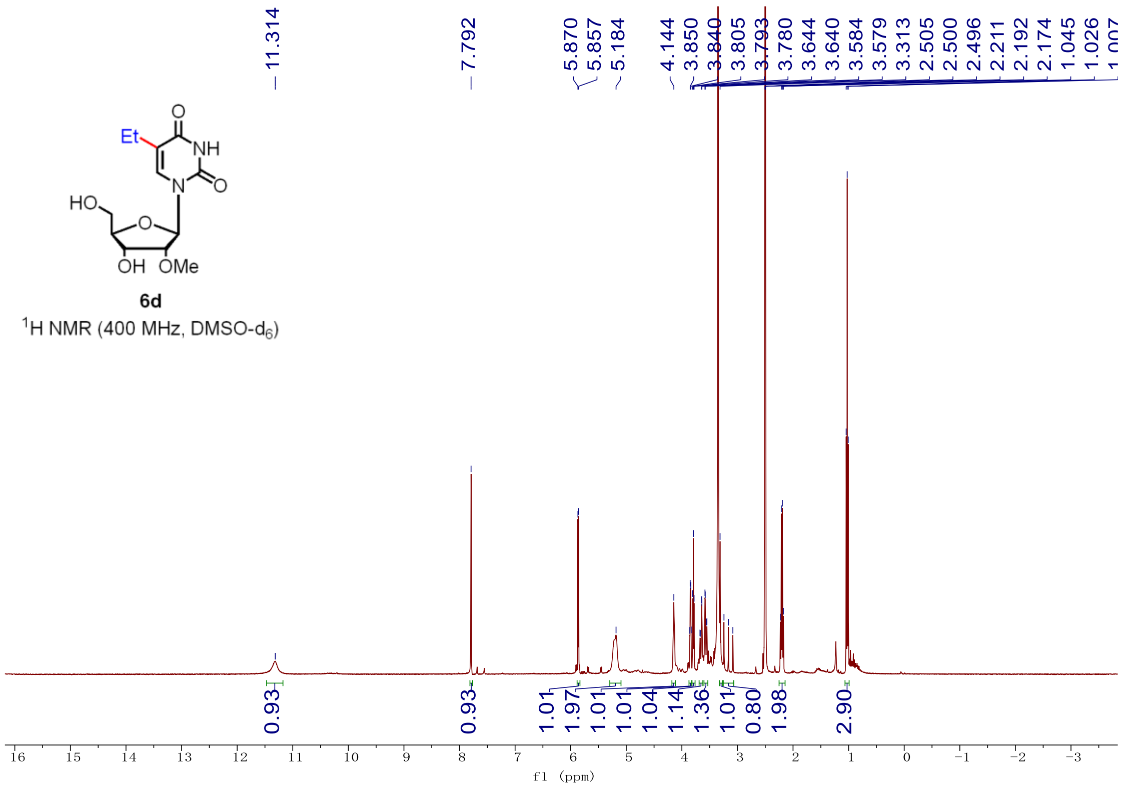

Supplementary Figure 225. ^{1}H NMR spectra of compound **6d**

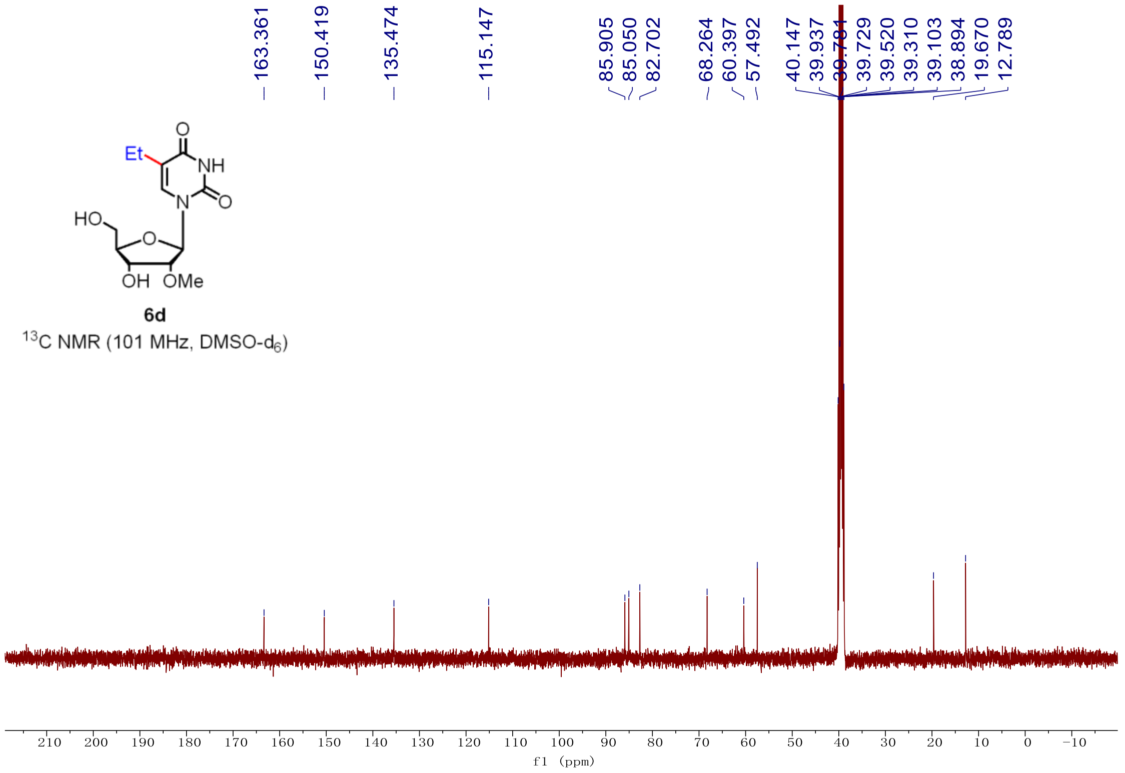

Supplementary Figure 226. ^{13}C NMR spectra of compound **6d**

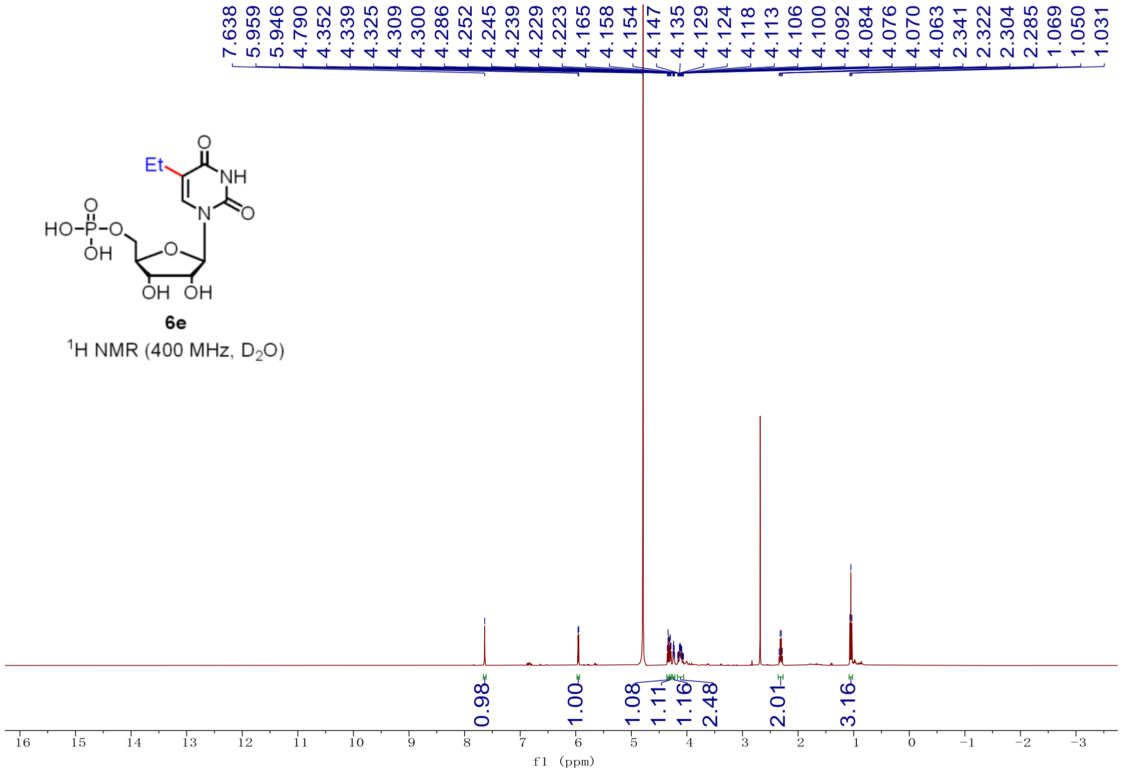

Supplementary Figure 227. ^{1}H NMR spectra of compound **6e**

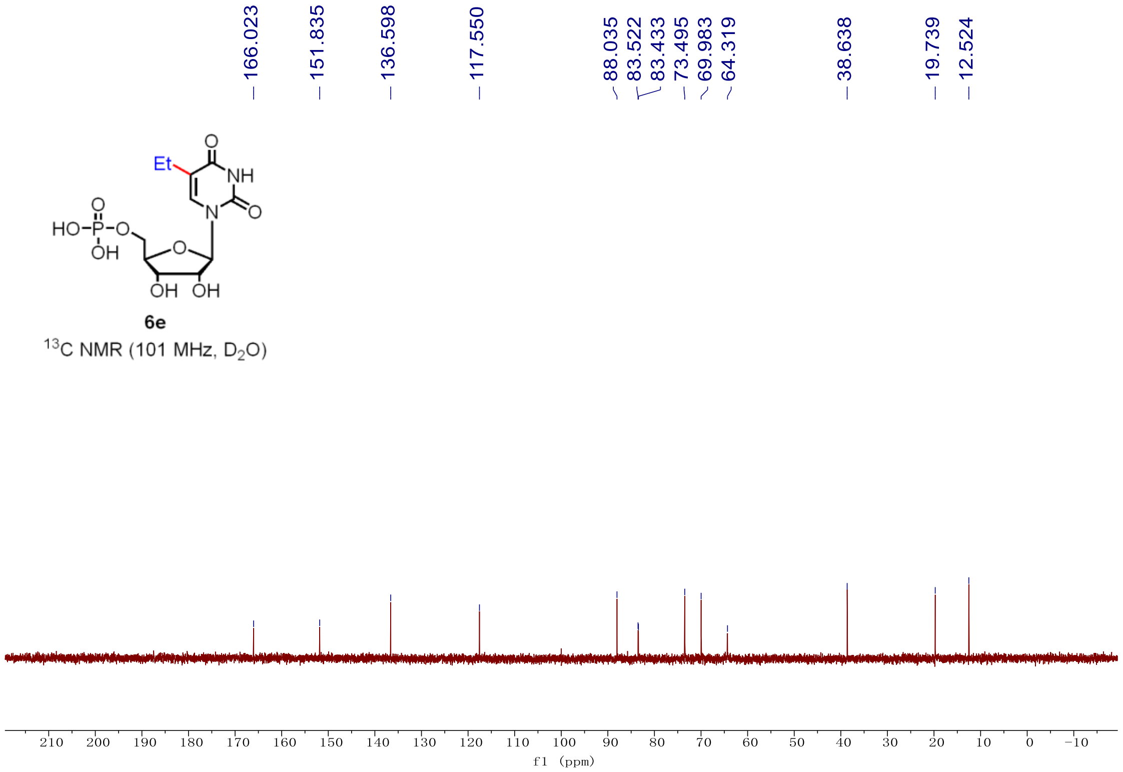

Supplementary Figure 228. ^{13}C NMR spectra of compound **6e**

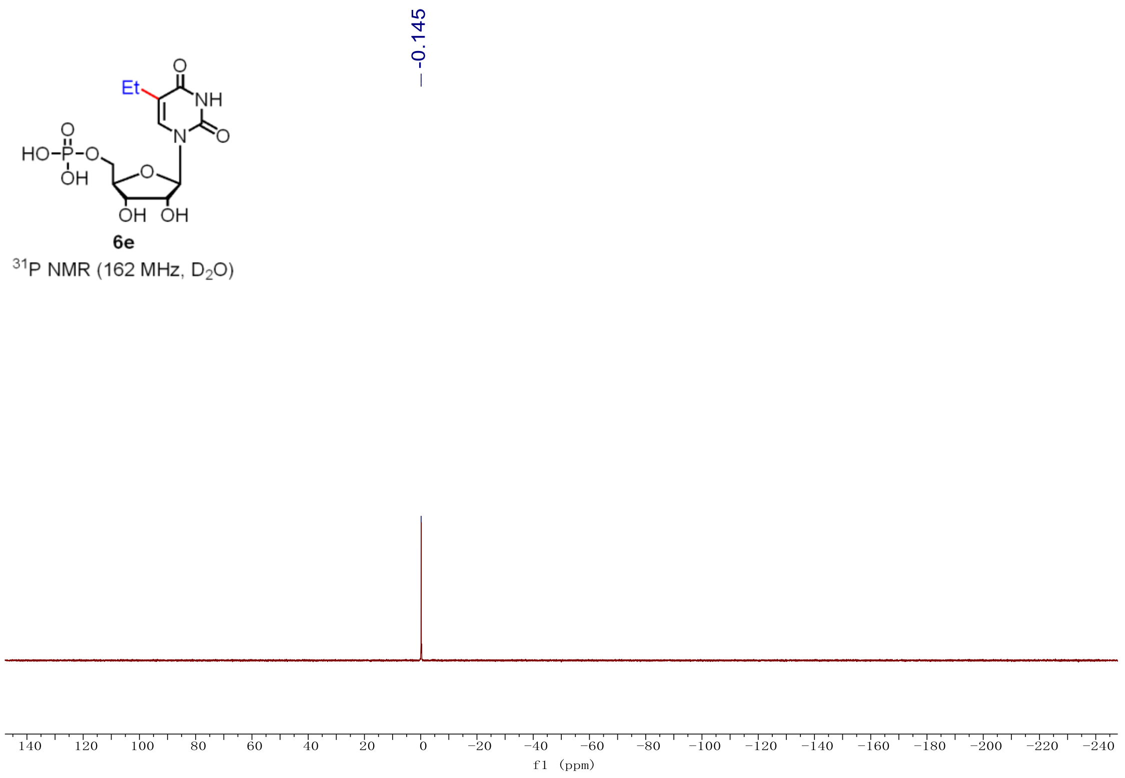

Supplementary Figure 229. ^{31}P NMR spectra of compound **6e**

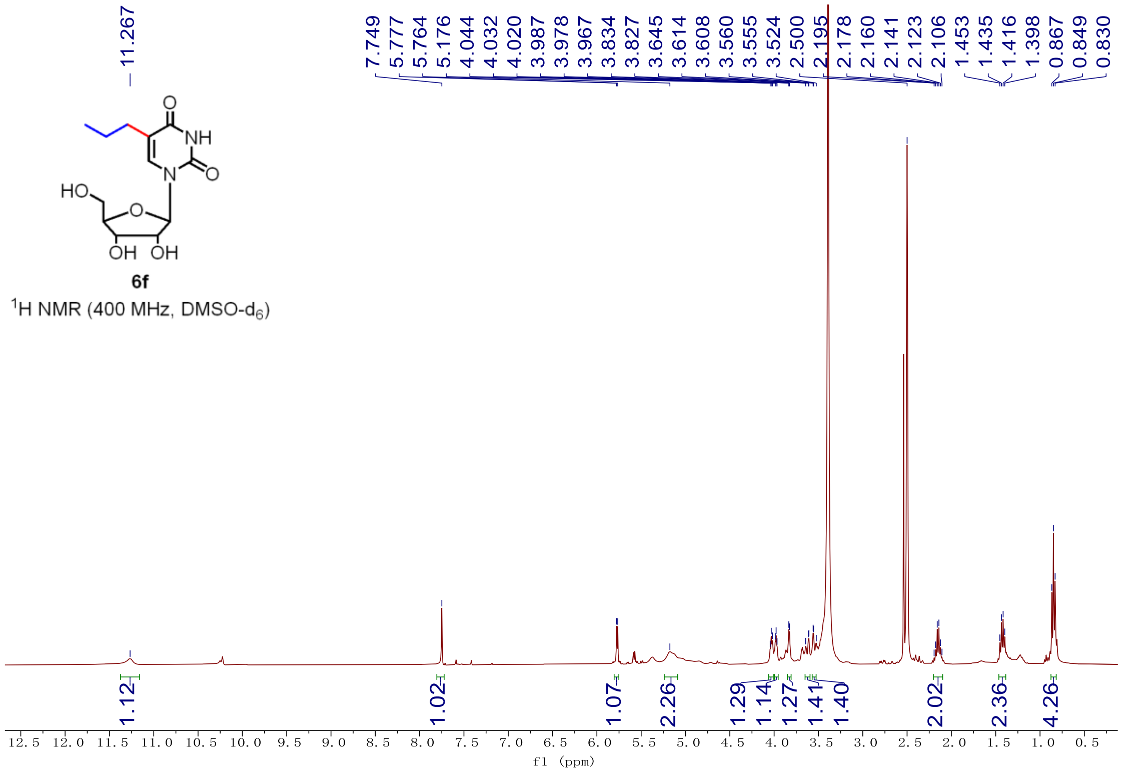

Supplementary Figure 230. ^{1}H NMR spectra of compound **6f**

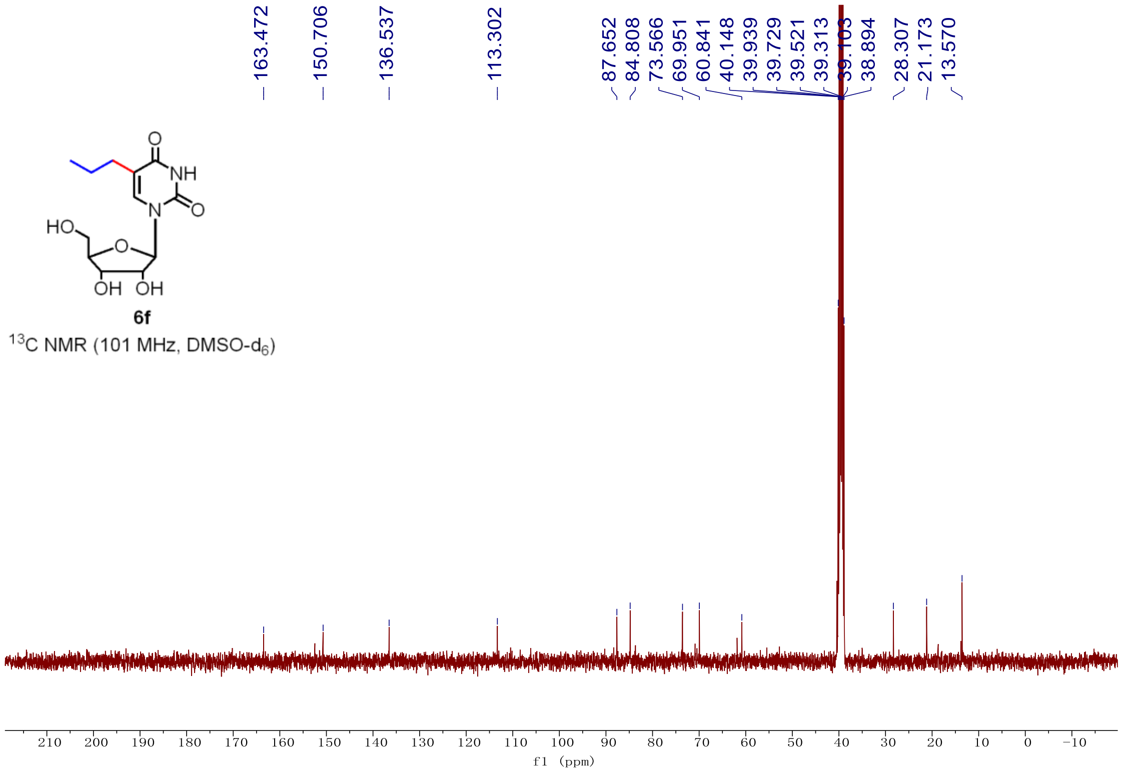

Supplementary Figure 231. ^{13}C NMR spectra of compound **6f**

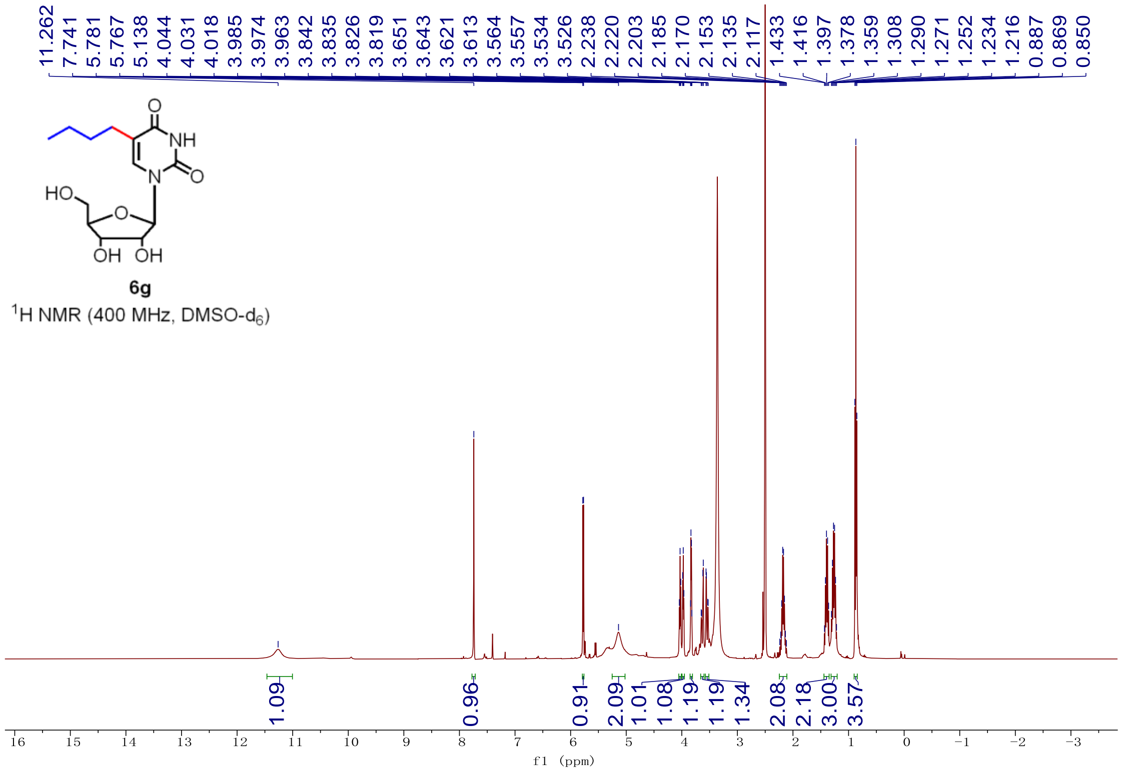

Supplementary Figure 232. ^{1}H NMR spectra of compound **6g**

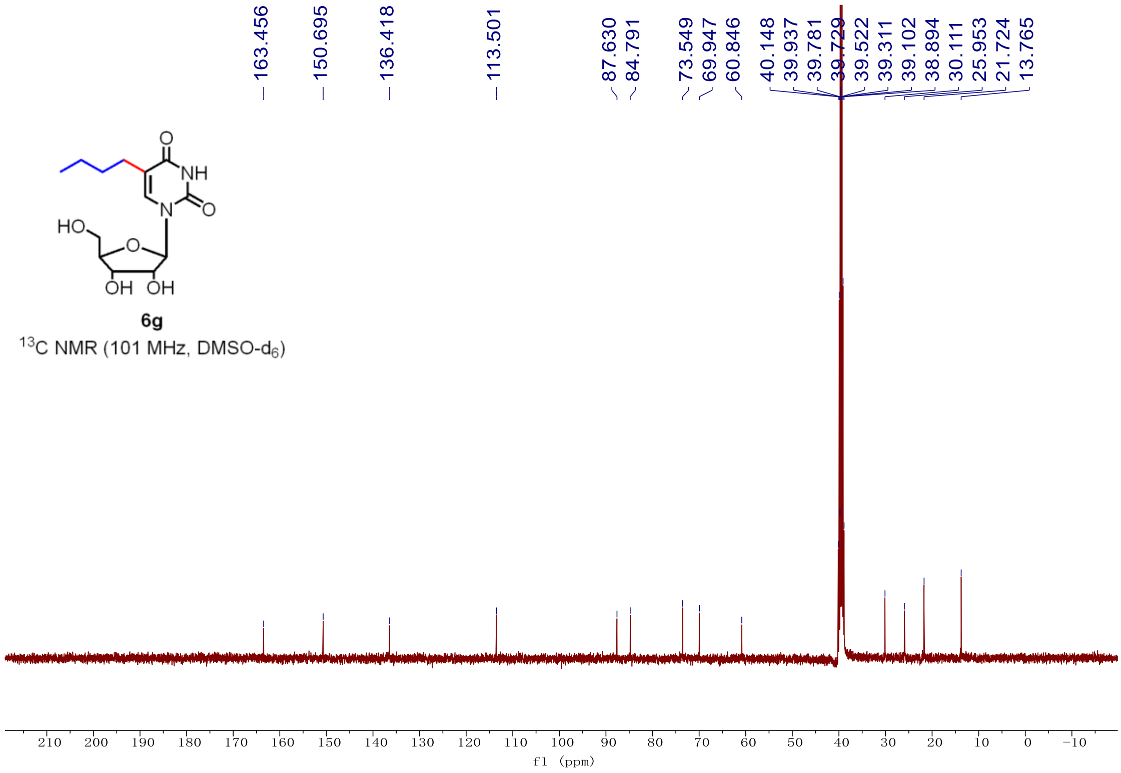

Supplementary Figure 233. ^{13}C NMR spectra of compound **6g**

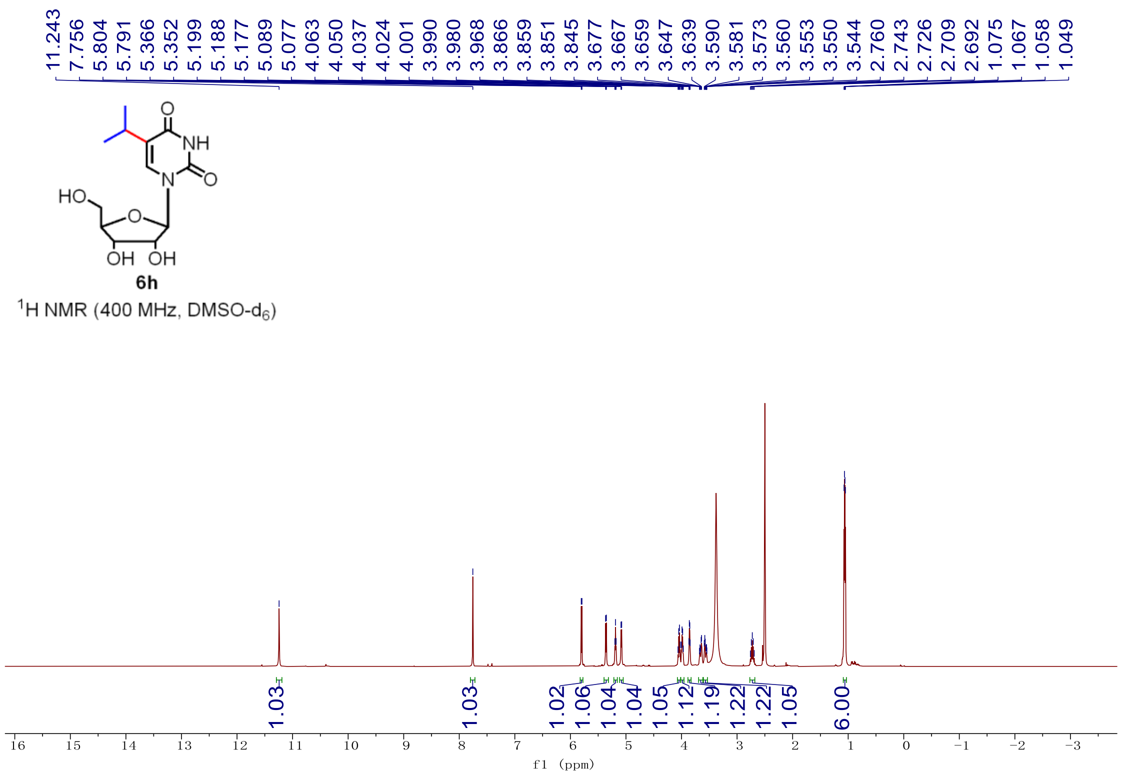

Supplementary Figure 234. ^{1}H NMR spectra of compound **6h**

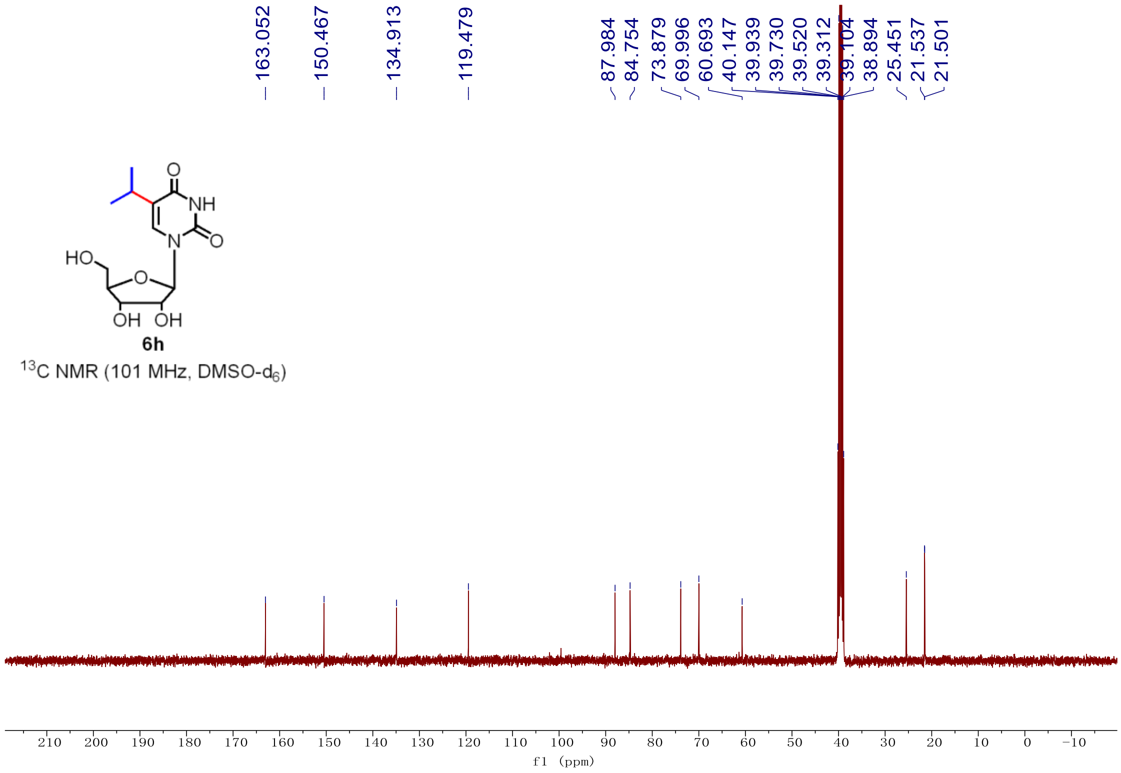

Supplementary Figure 235. ^{13}C NMR spectra of compound **6h**

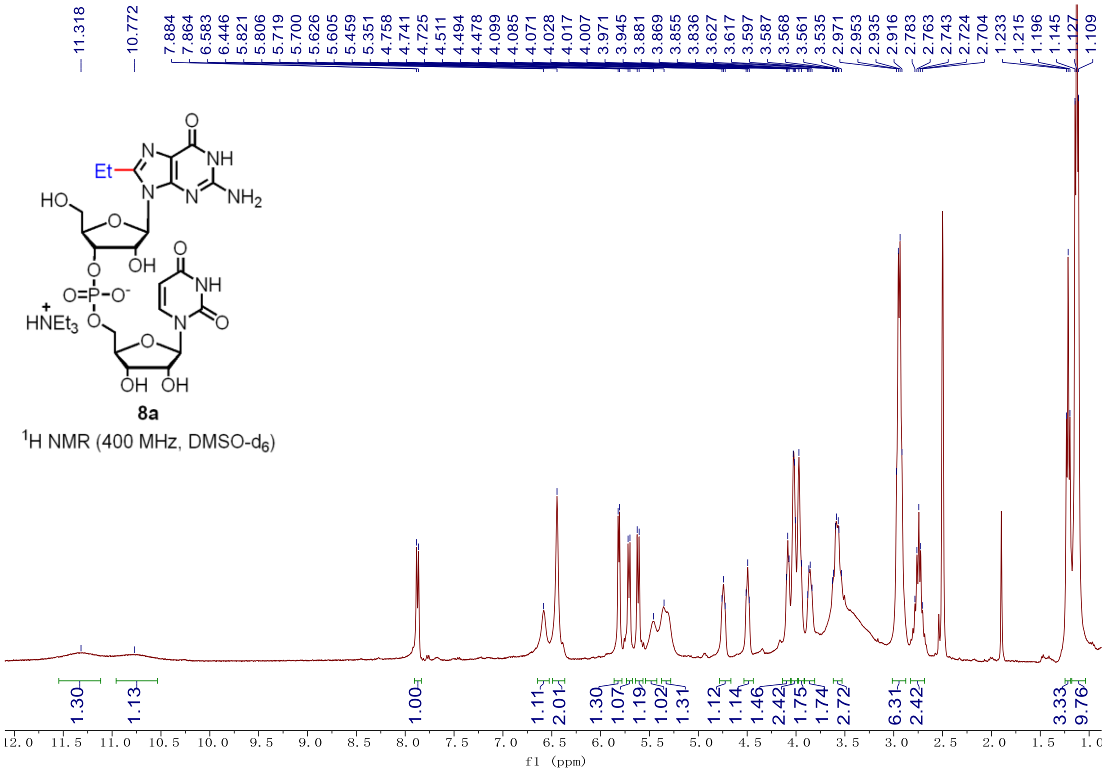

Supplementary Figure 236. ^{1}H NMR spectra of compound **8a**

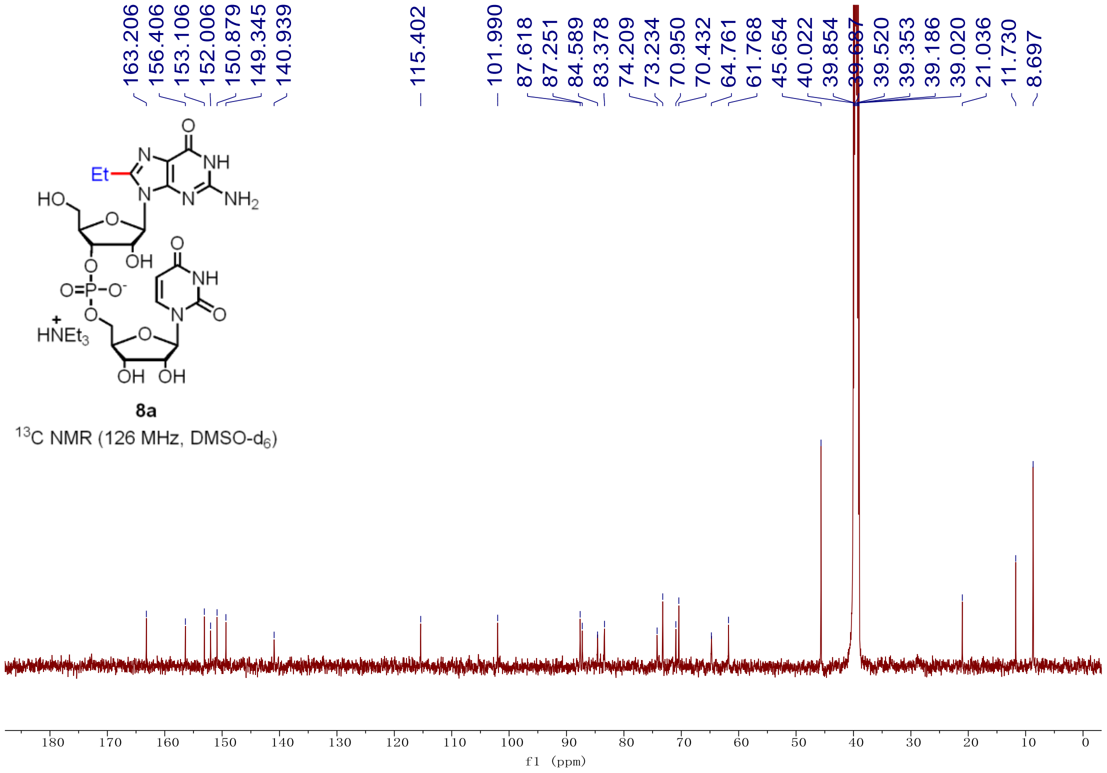

Supplementary Figure 237. ^{13}C NMR spectra of compound **8a**

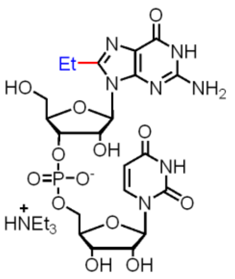

8a

^{31}P NMR (202 MHz, DMSO-d_6)

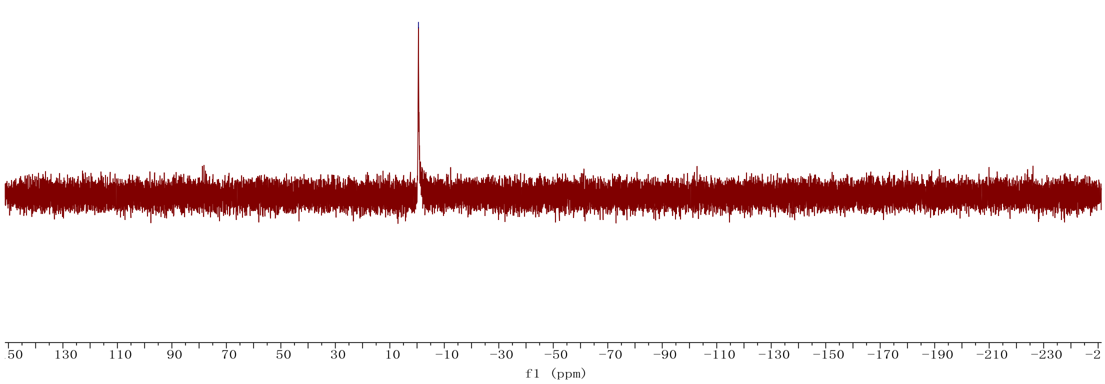

Supplementary Figure 238. ^{31}P NMR spectra of compound **8a**

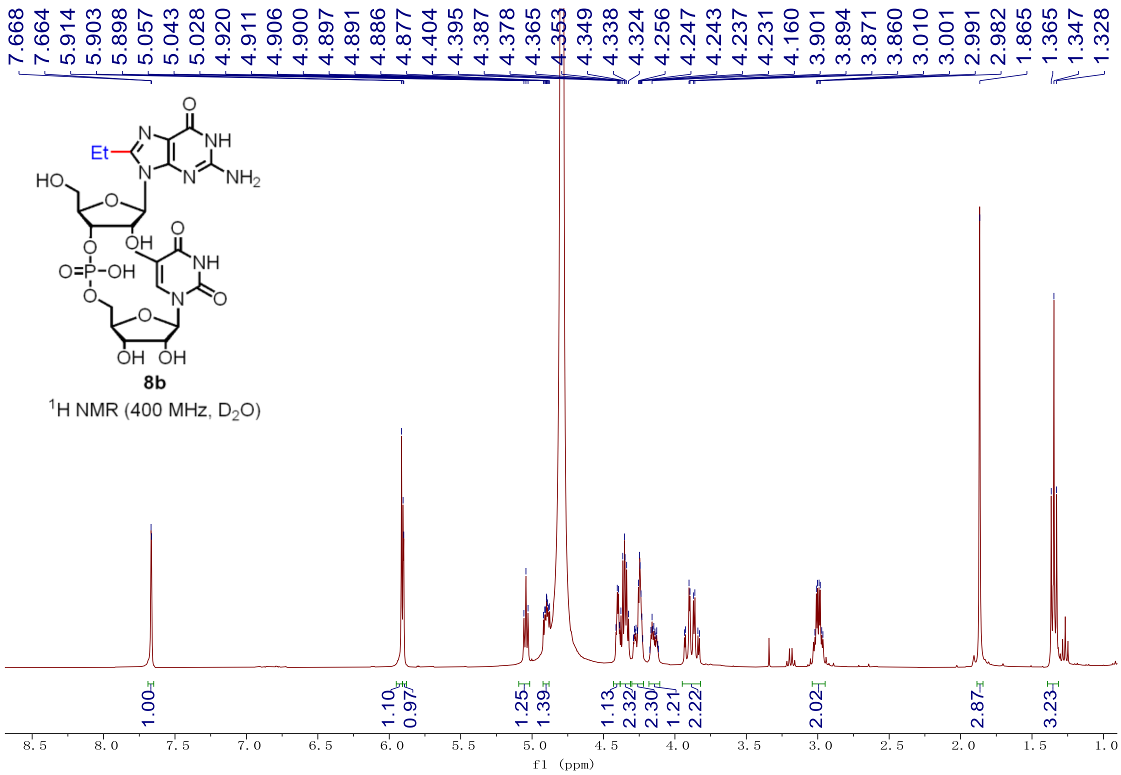

Supplementary Figure 239. ^{1}H NMR spectra of compound **8b**

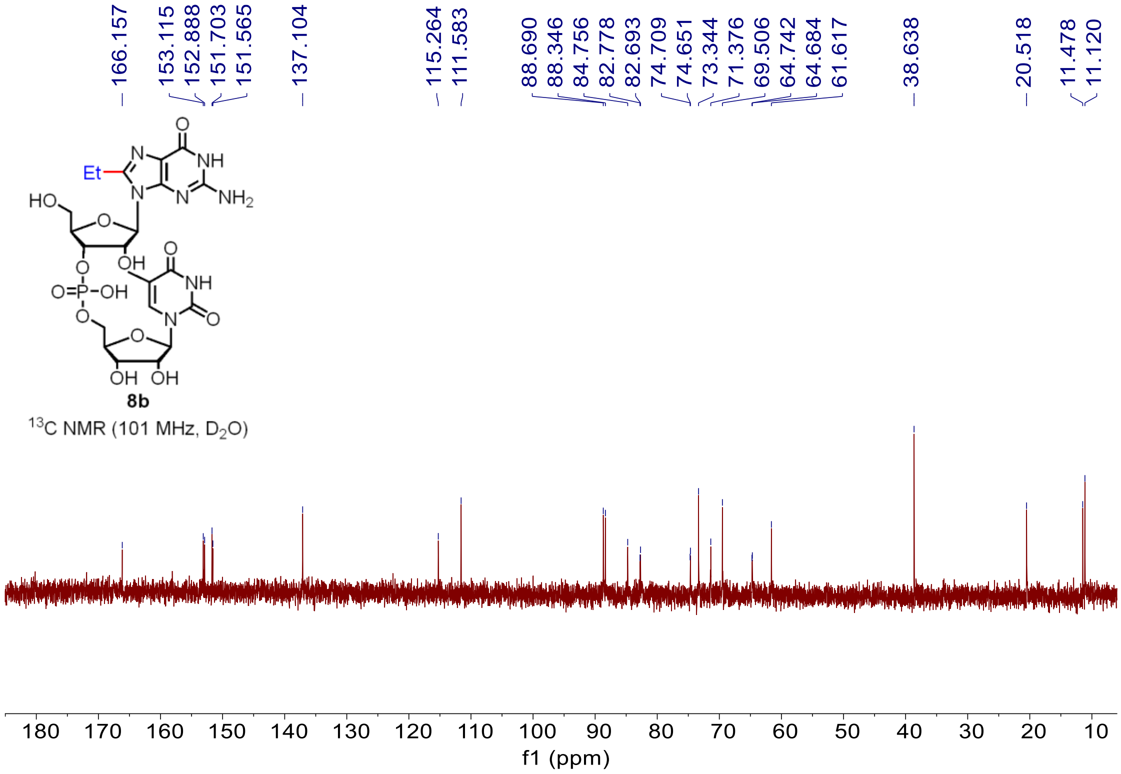

Supplementary Figure 240. ^{13}C NMR spectra of compound **8b**

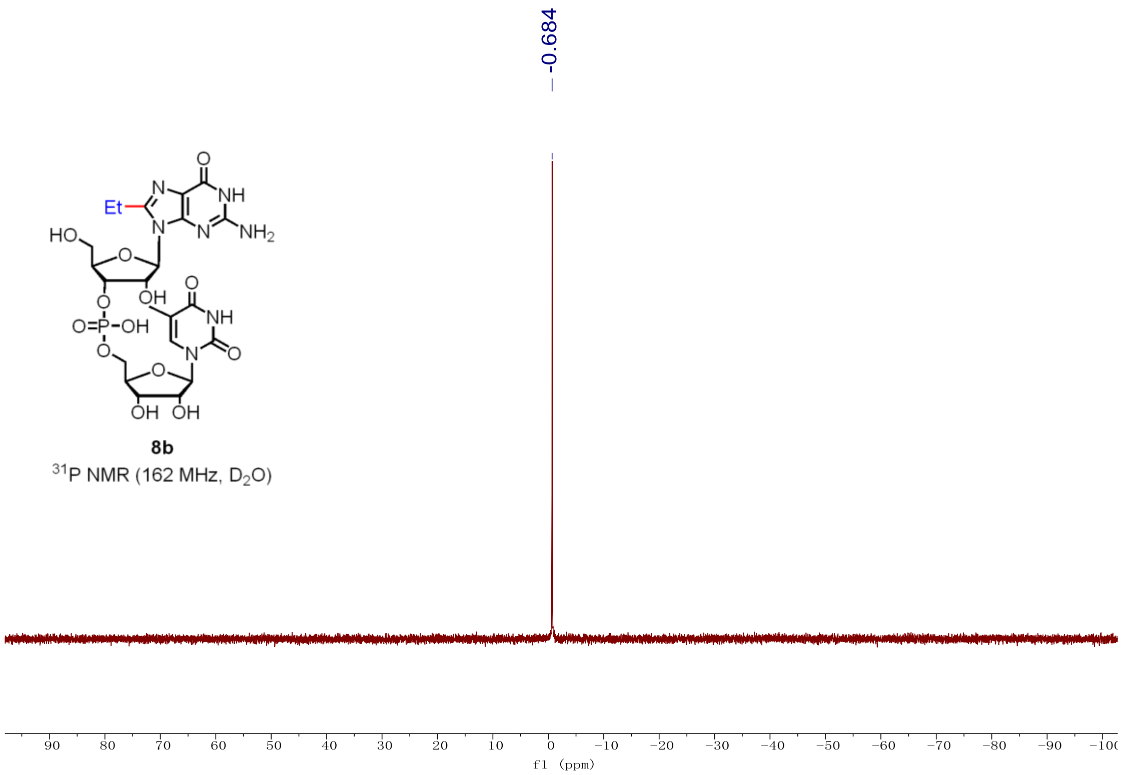

Supplementary Figure 241. ^{31}P NMR spectra of compound **8b**

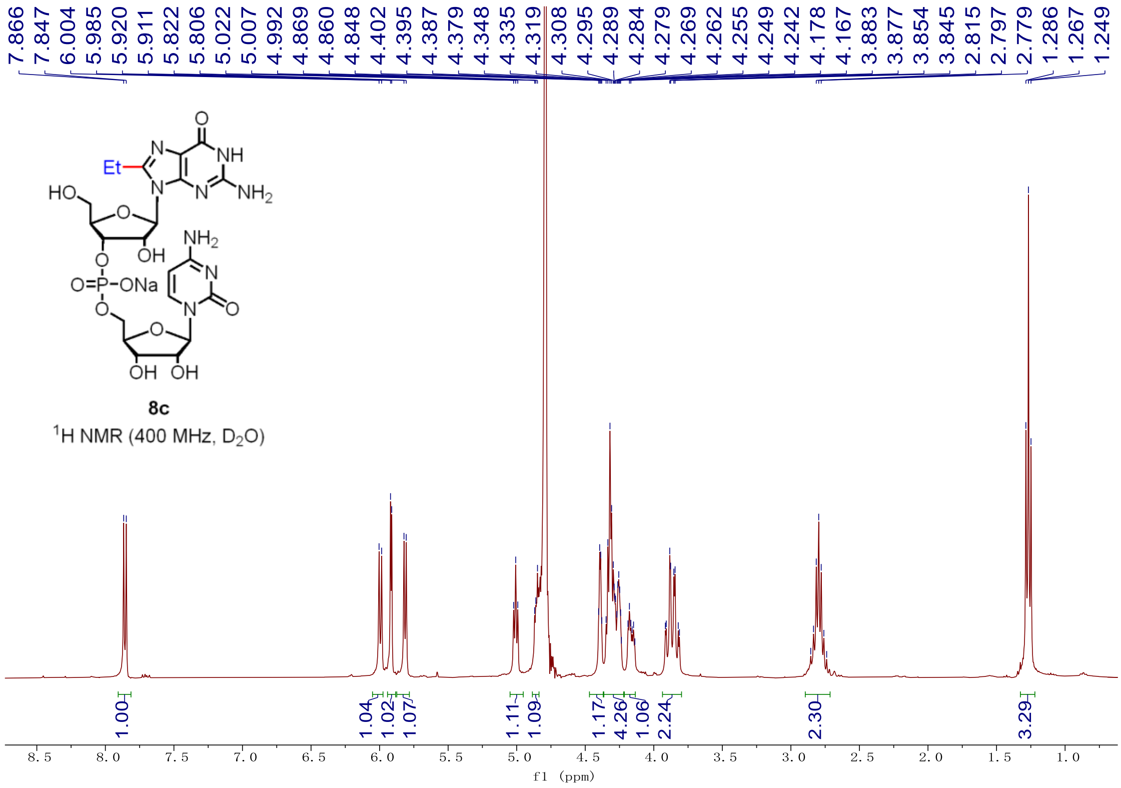

Supplementary Figure 242. ^{1}H NMR spectra of compound **8c**

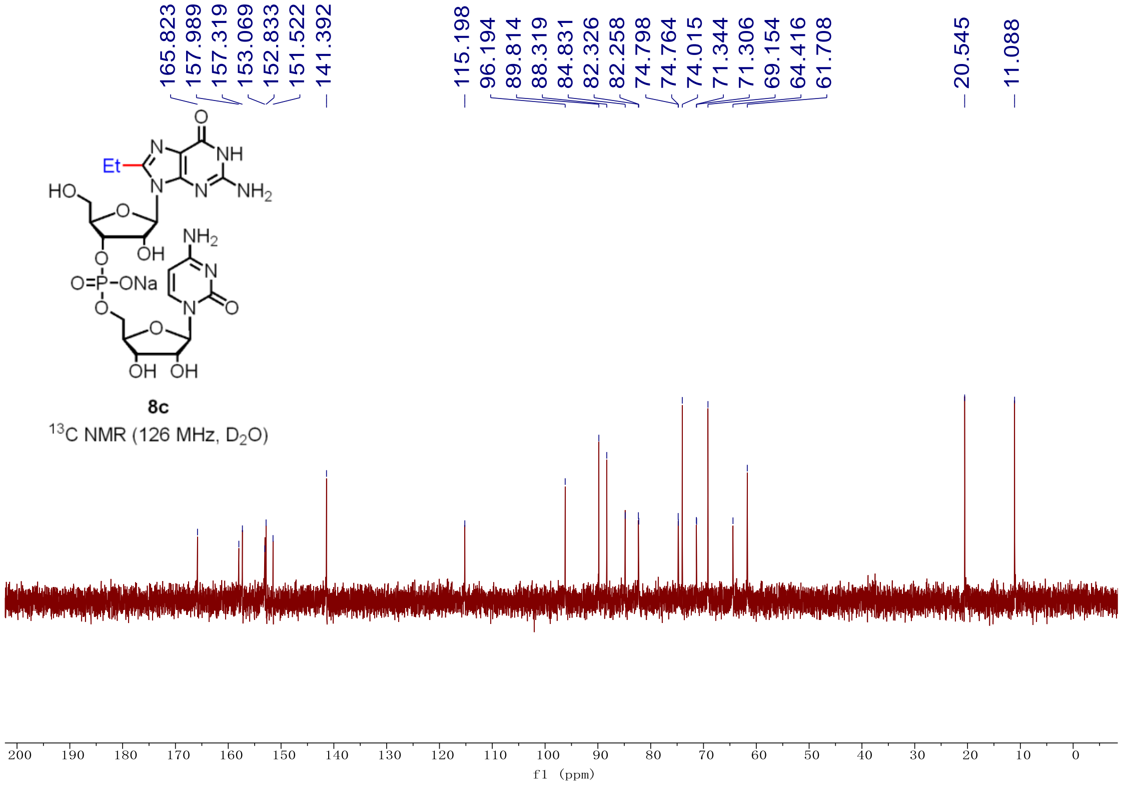

Supplementary Figure 243. ^{13}C NMR spectra of compound **8c**

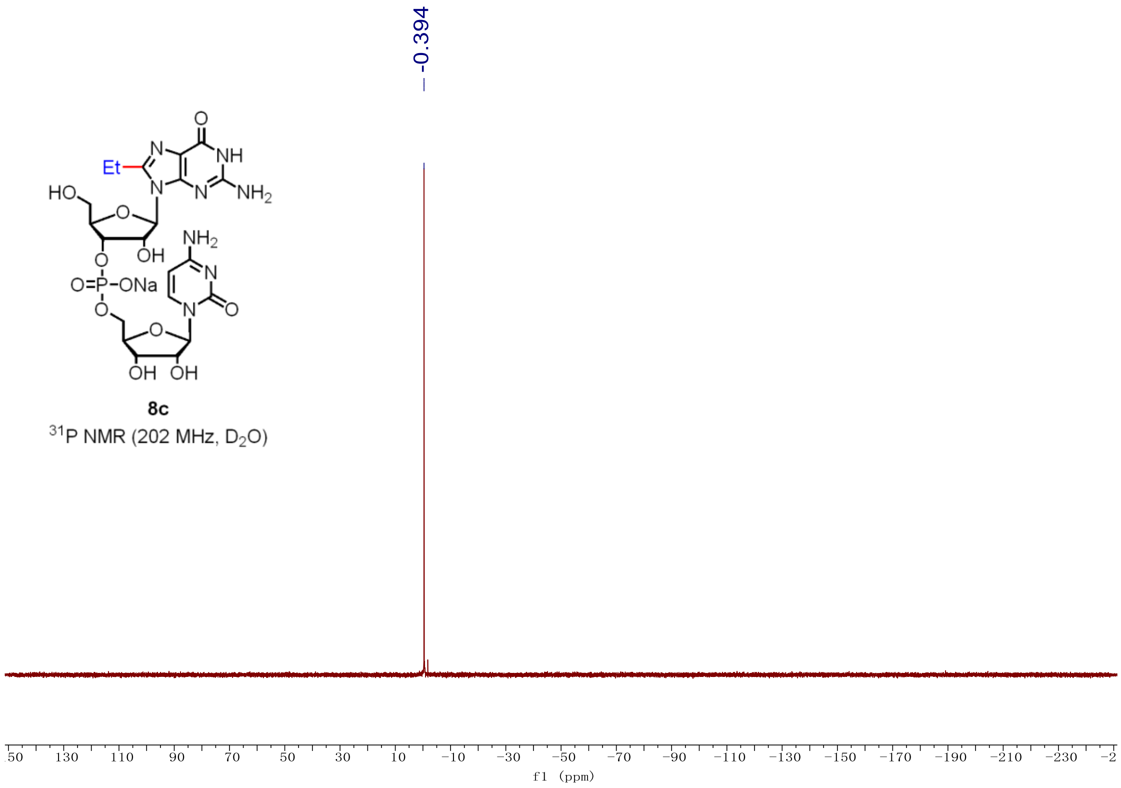

Supplementary Figure 244. ^{31}P NMR spectra of compound **8c**

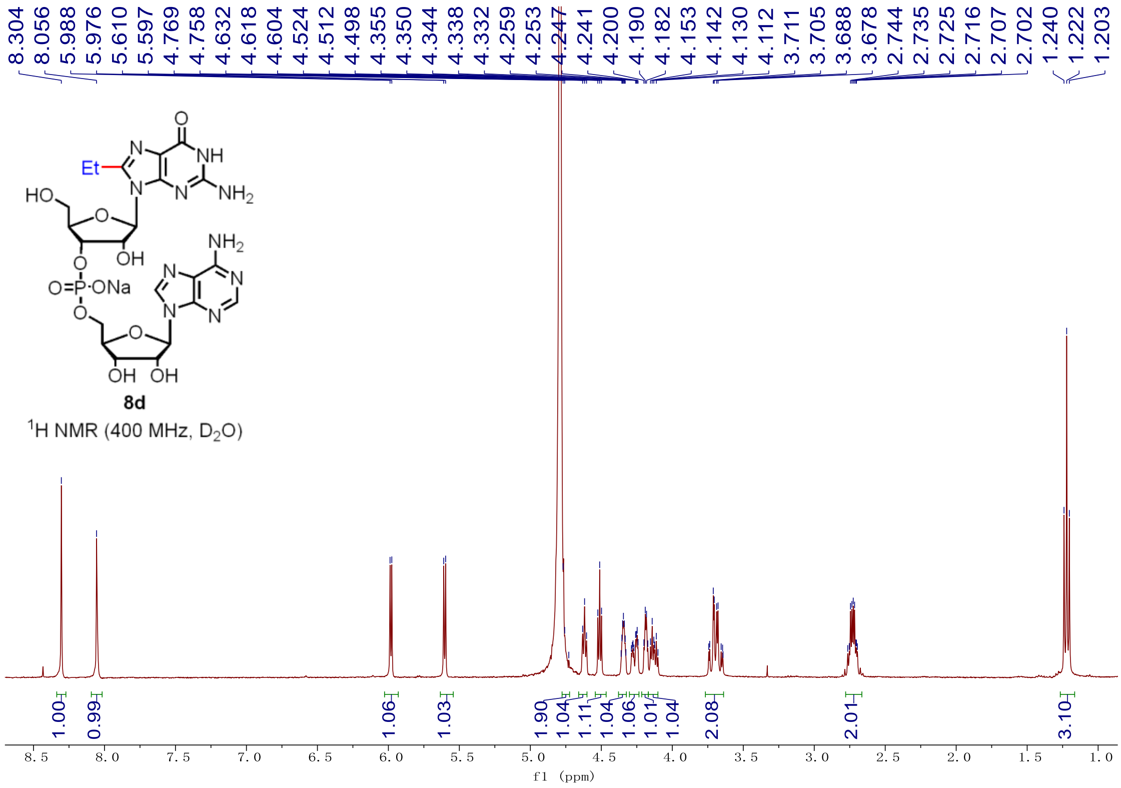

Supplementary Figure 245. ^{1}H NMR spectra of compound **8d**

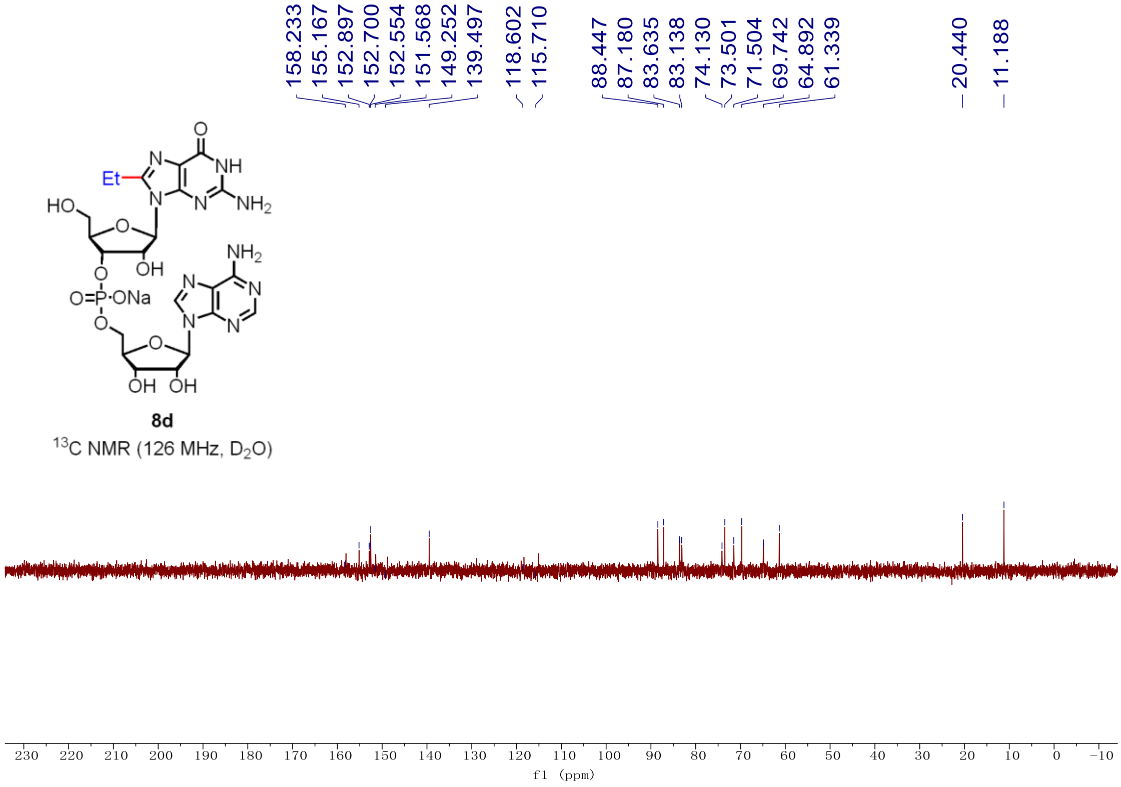

Supplementary Figure 246. ^{13}C NMR spectra of compound **8d**

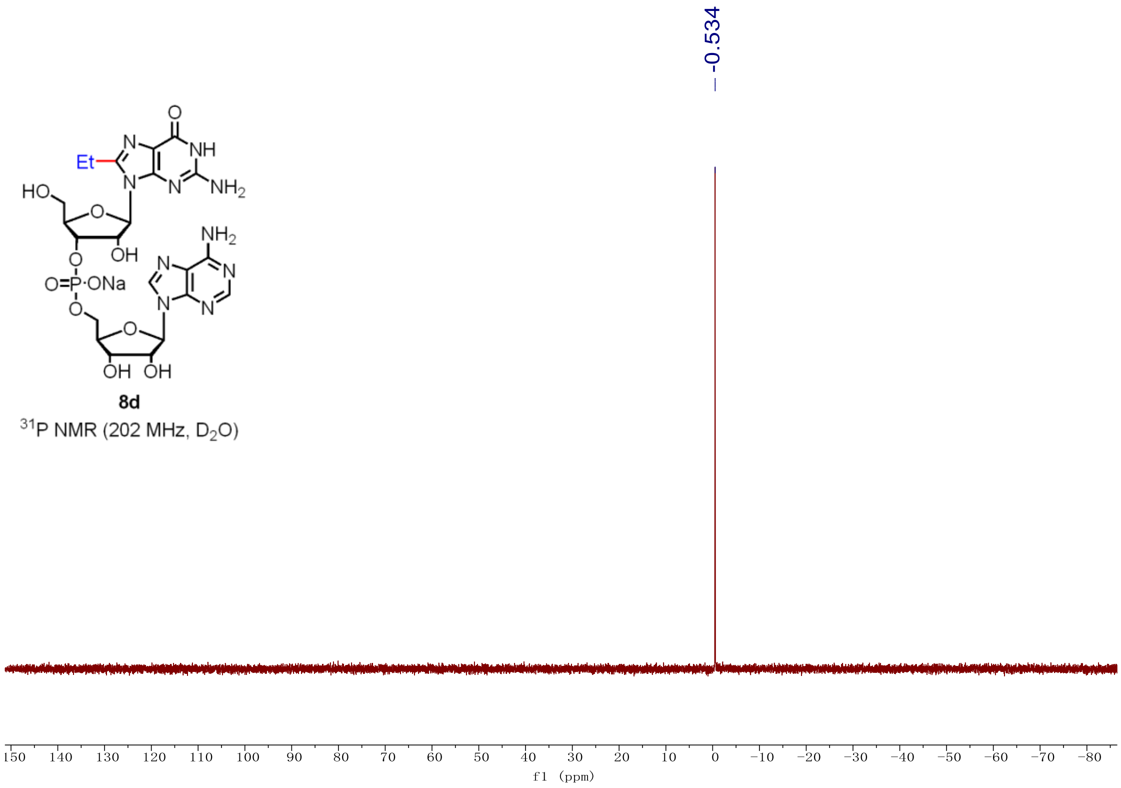

Supplementary Figure 247. ^{31}P NMR spectra of compound **8d**

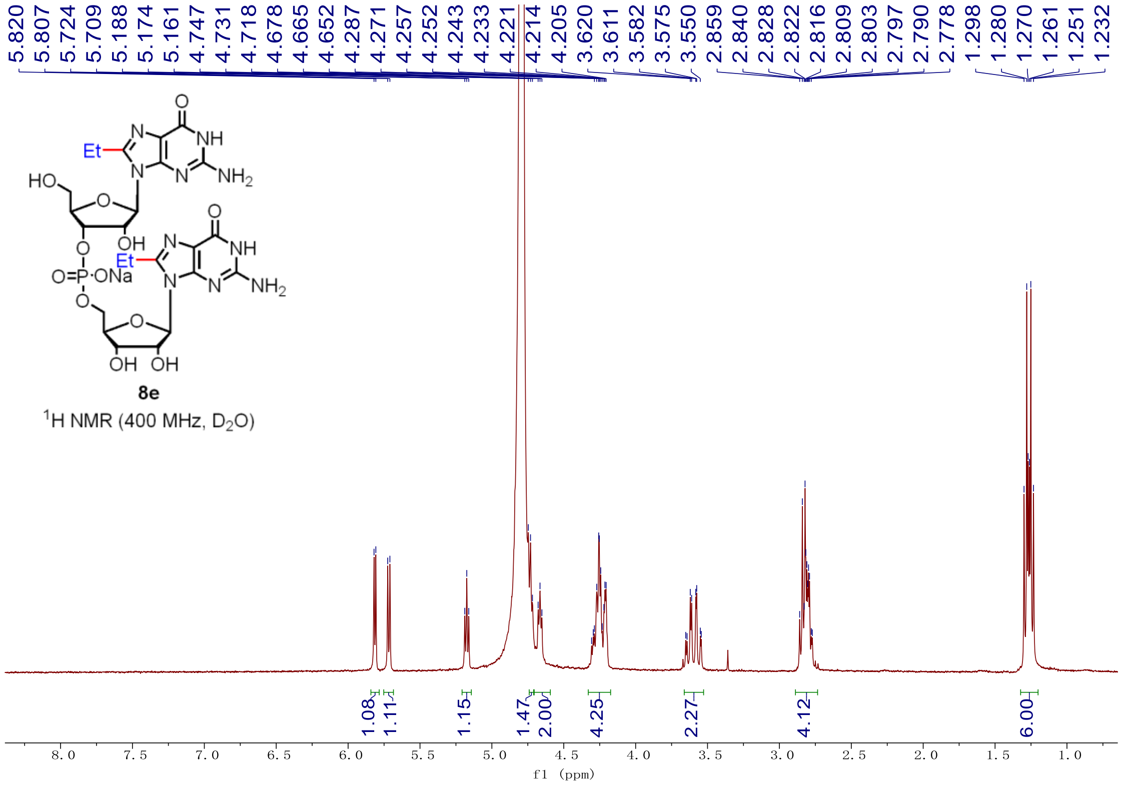

Supplementary Figure 248. ^{1}H NMR spectra of compound **8e**

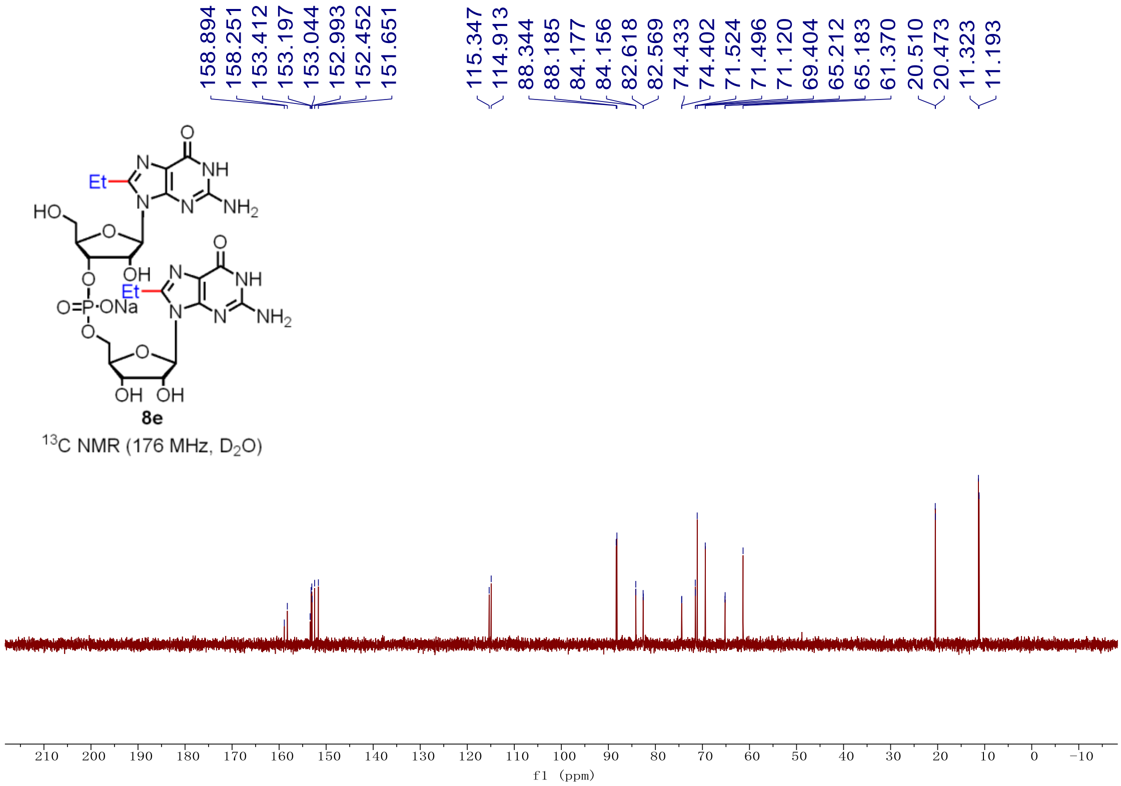

Supplementary Figure 249. ^{13}C NMR spectra of compound **8e**

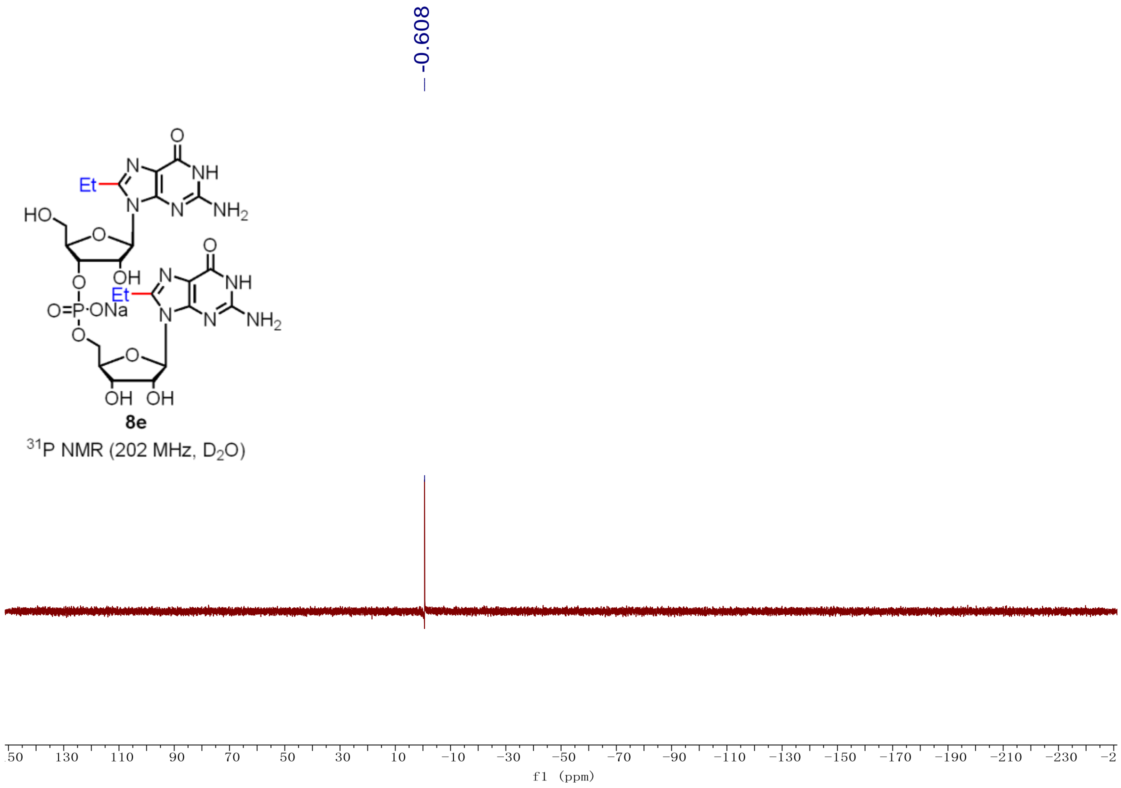

Supplementary Figure 250. ^{31}P NMR spectra of compound **8e**

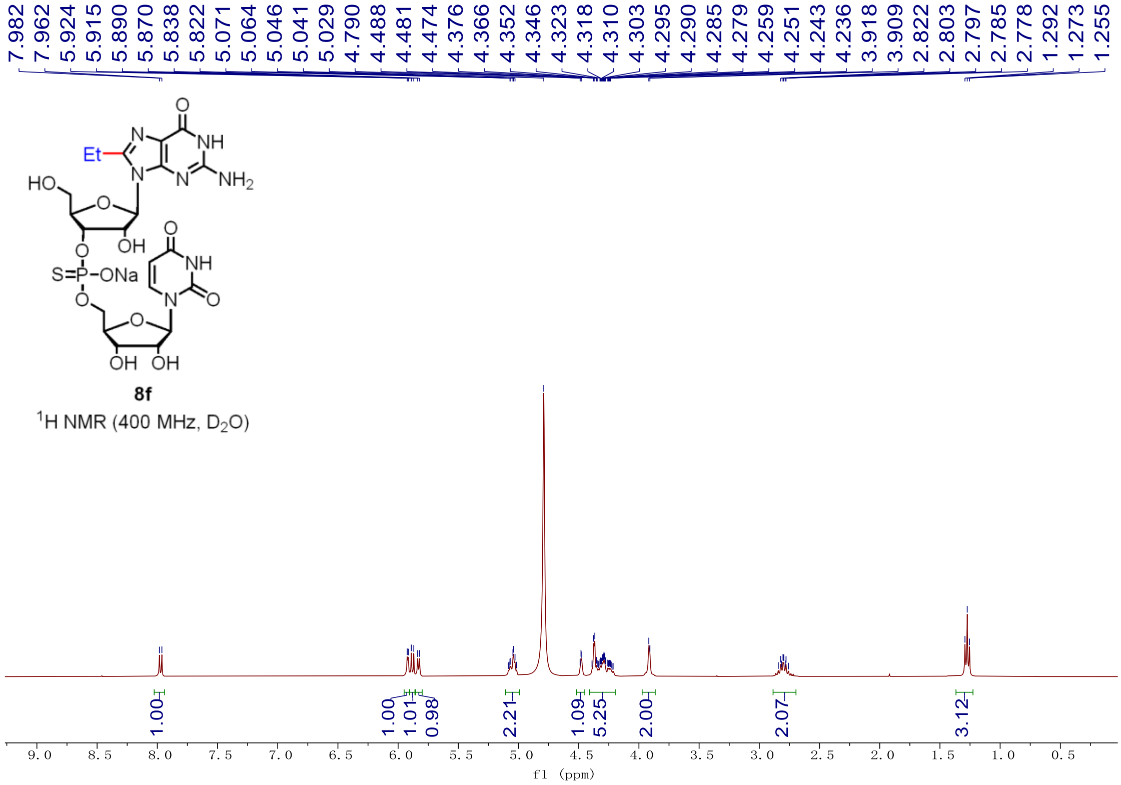

Supplementary Figure 251. ^{1}H NMR spectra of compound **8f**

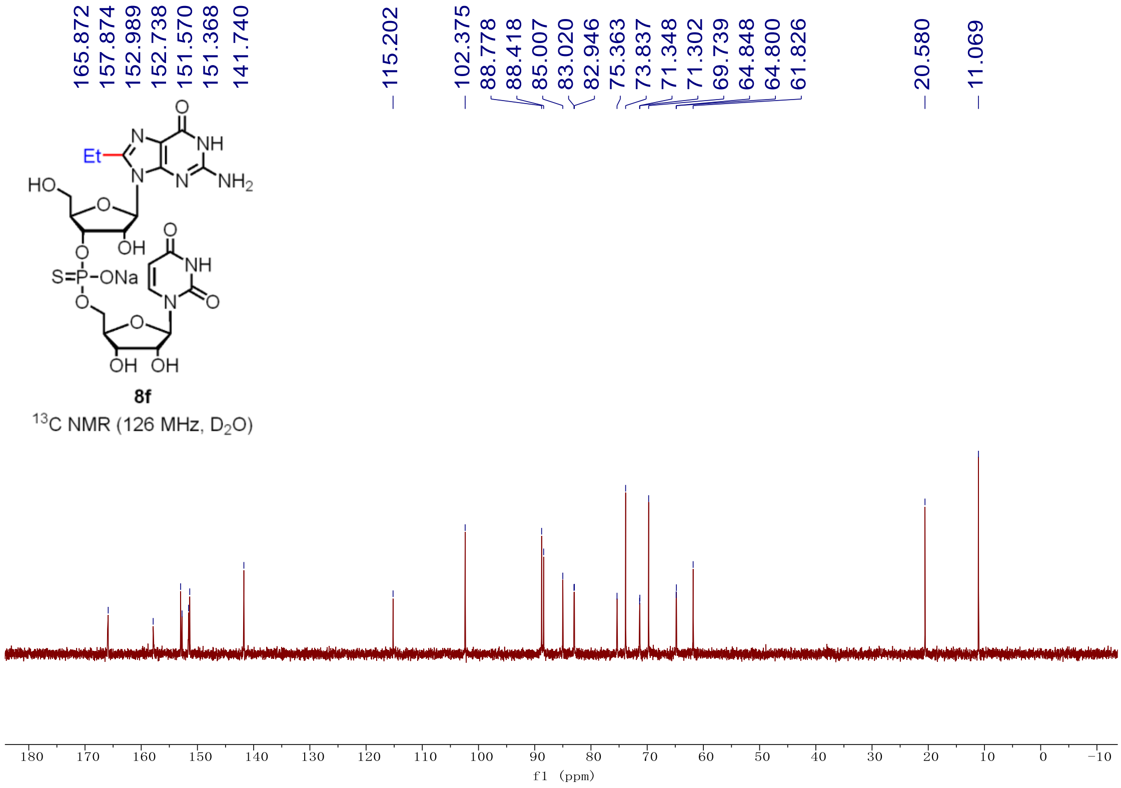

Supplementary Figure 252. ^{13}C NMR spectra of compound **8f**

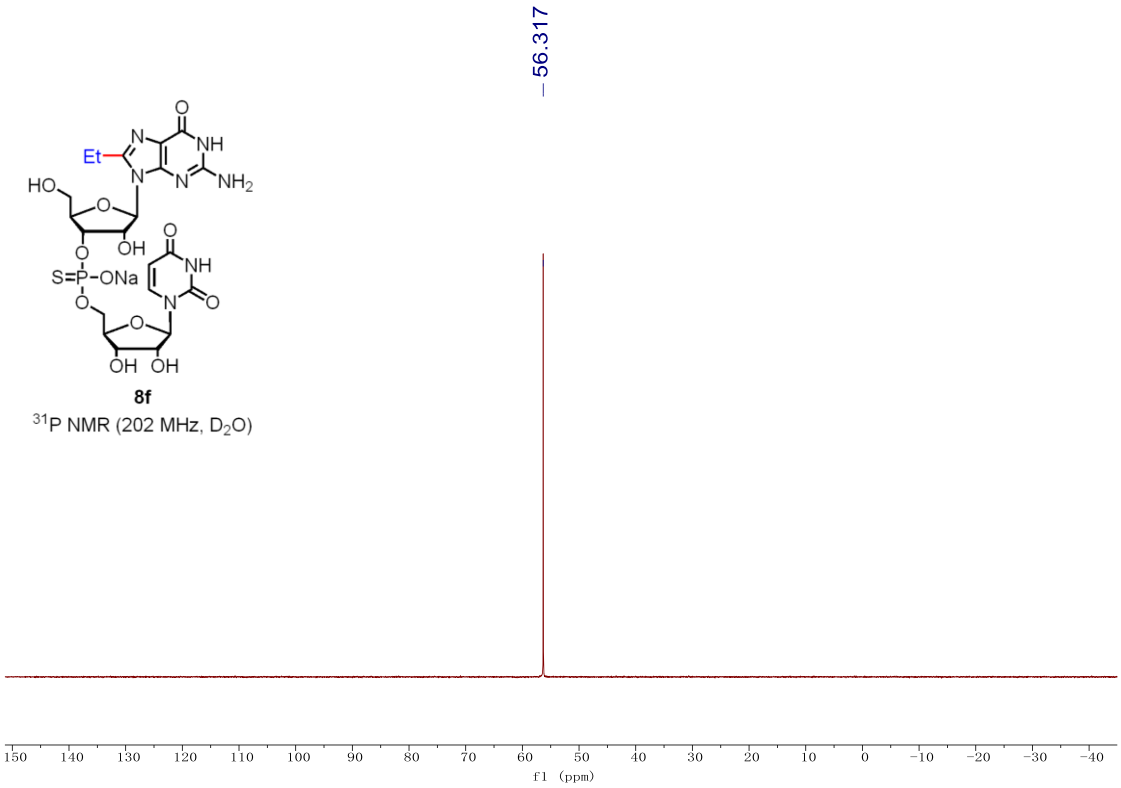

Supplementary Figure 253. ^{31}P NMR spectra of compound **8f**

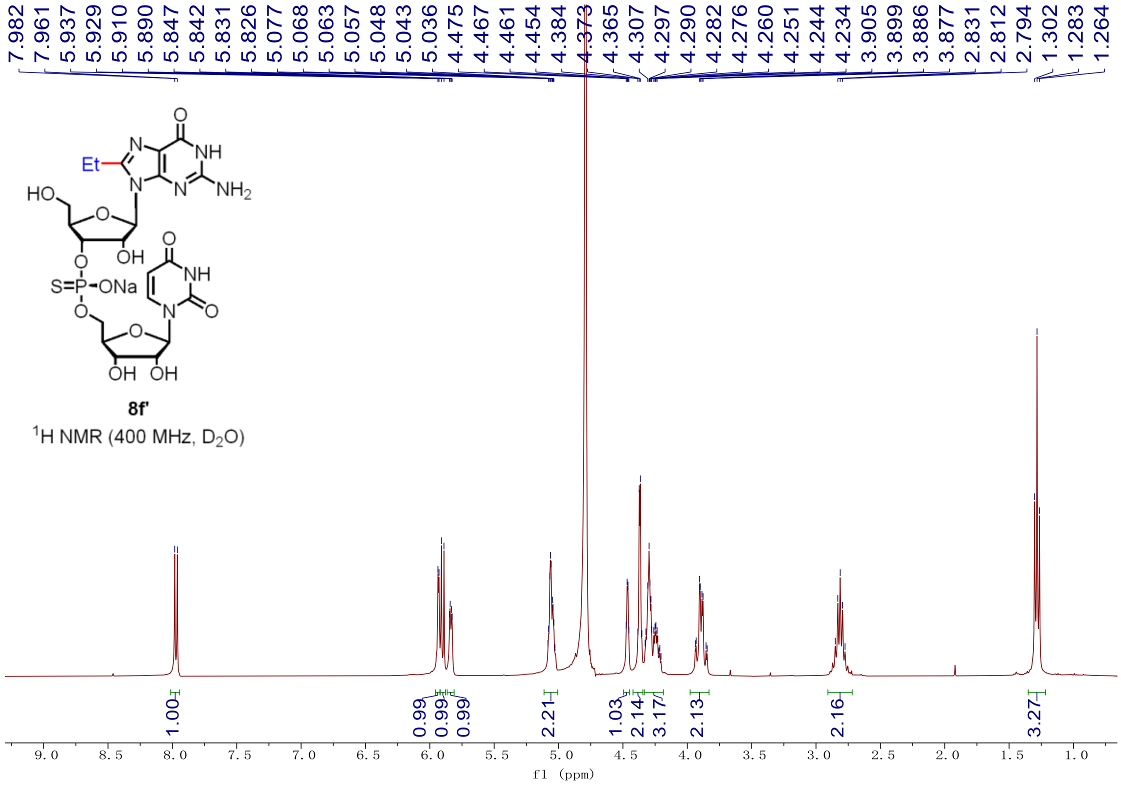

Supplementary Figure 254. ^{1}H NMR spectra of compound **8f'**

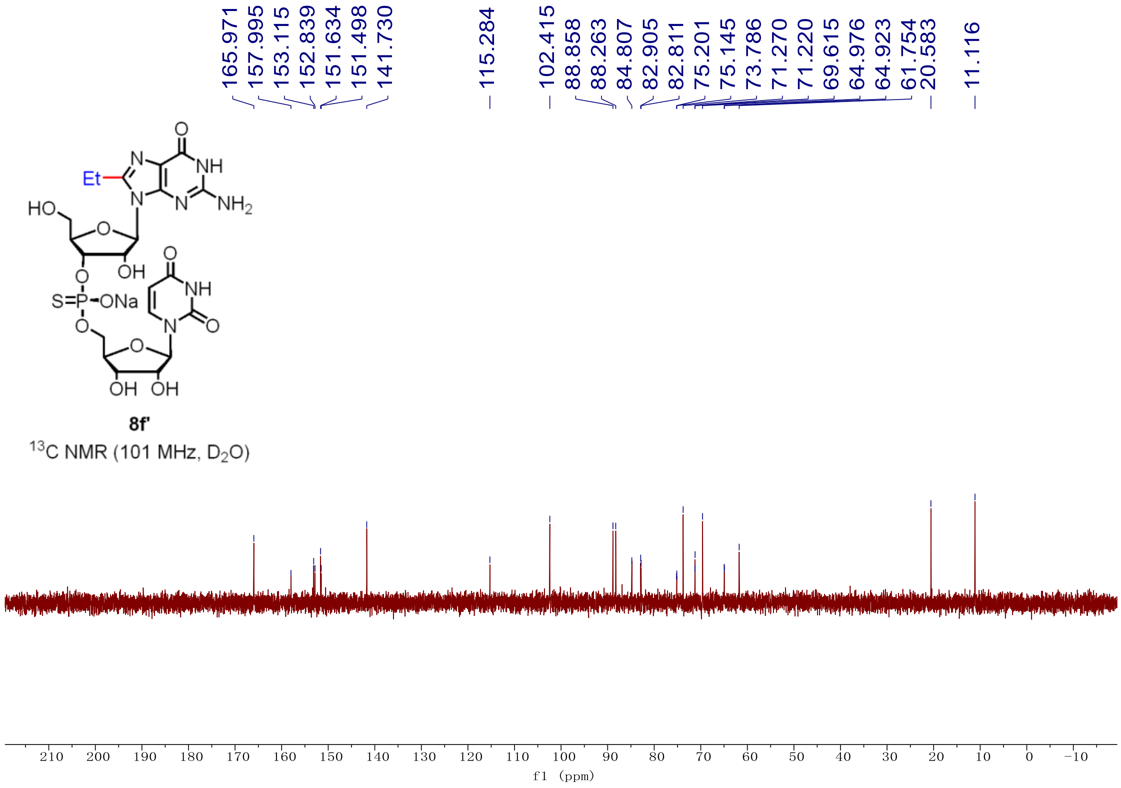

Supplementary Figure 255. ^{13}C NMR spectra of compound **8f'**

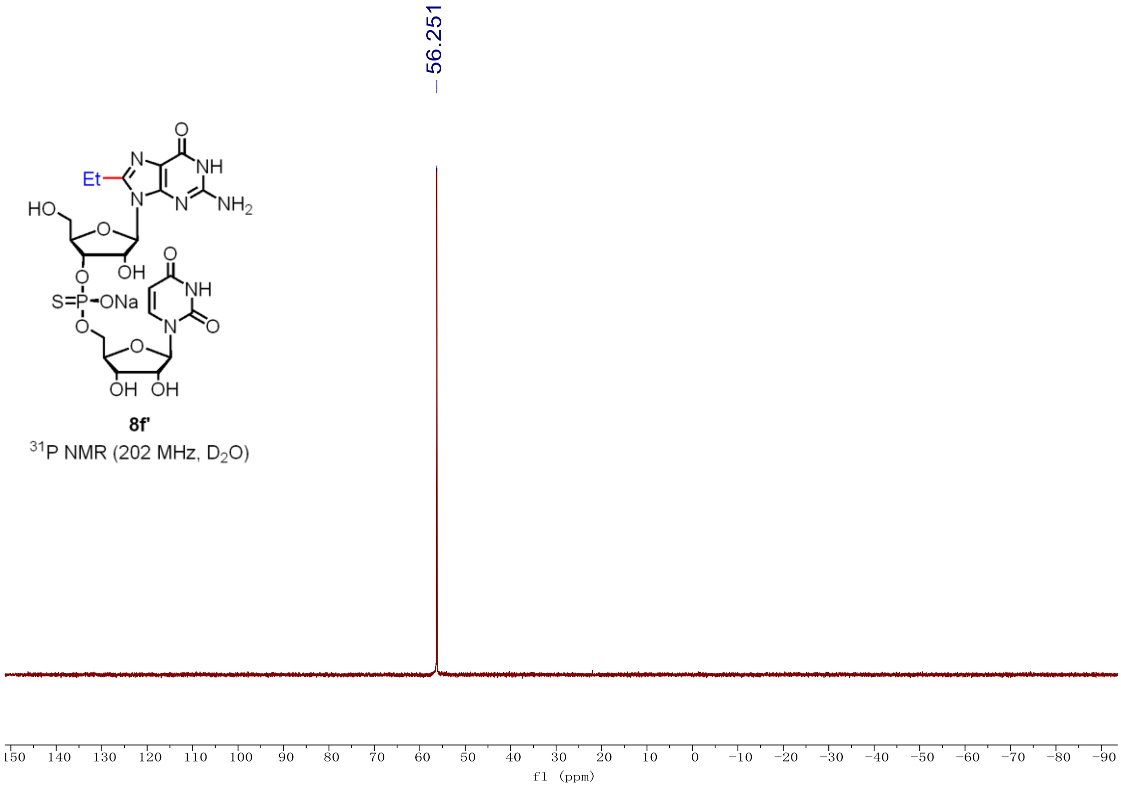

Supplementary Figure 256. ^{31}P NMR spectra of compound **8f'**

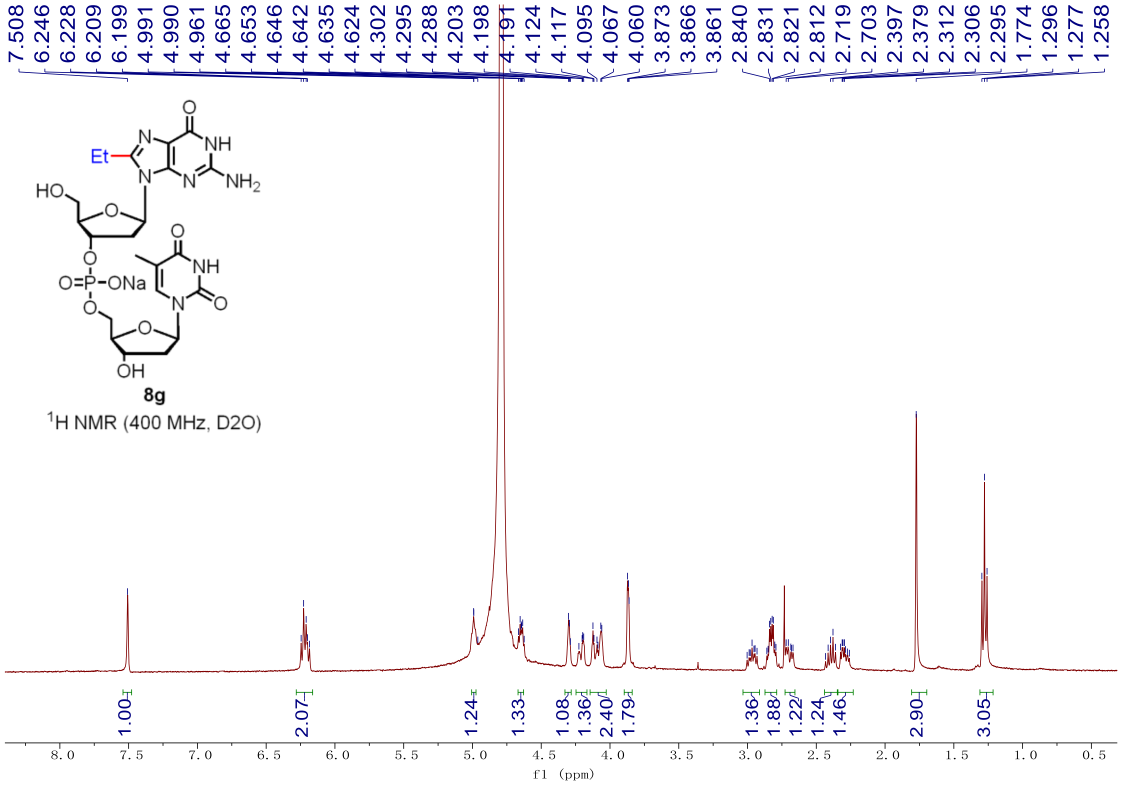

Supplementary Figure 257. ^{1}H NMR spectra of compound **8g**

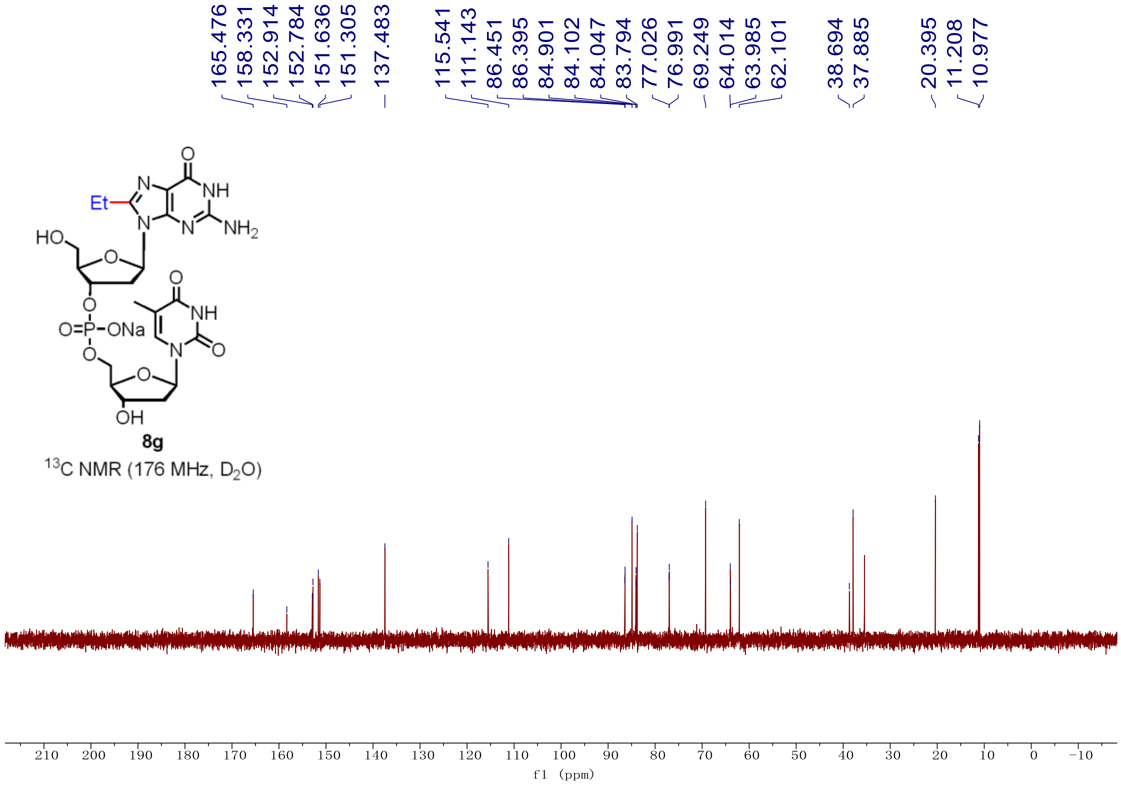

Supplementary Figure 258. ^{13}C NMR spectra of compound **8g**

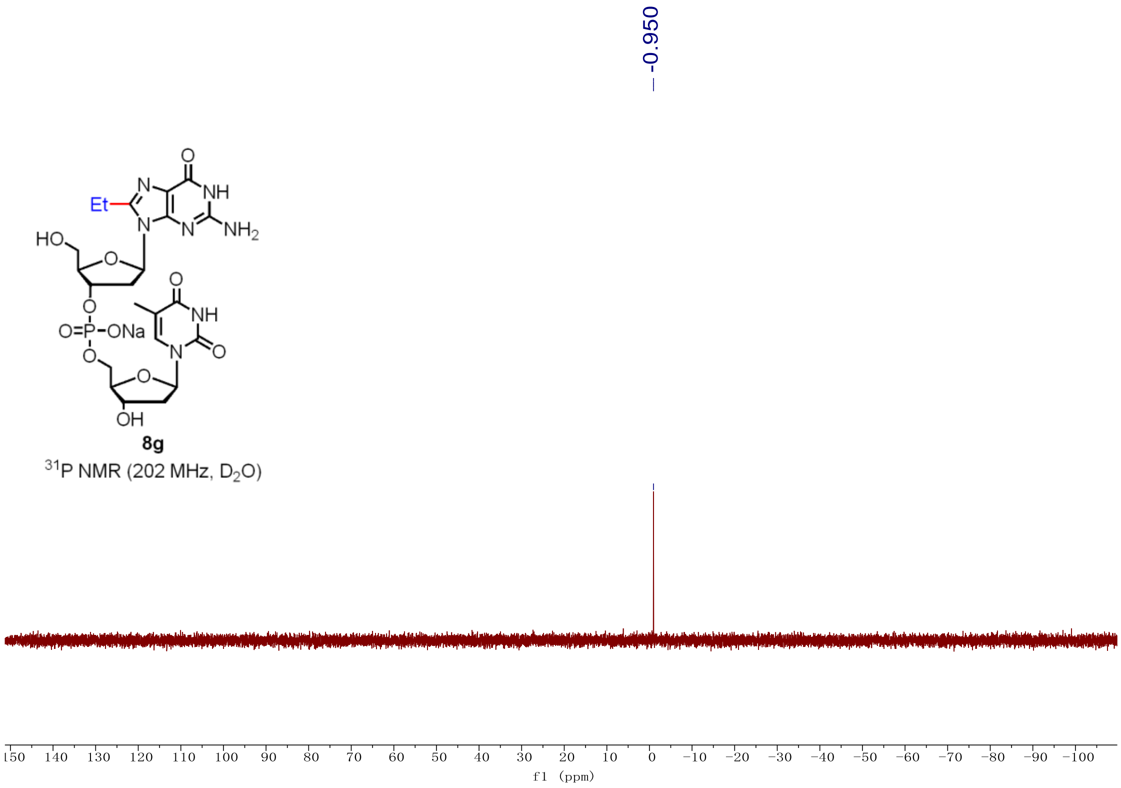

Supplementary Figure 259. ^{31}P NMR spectra of compound **8g**

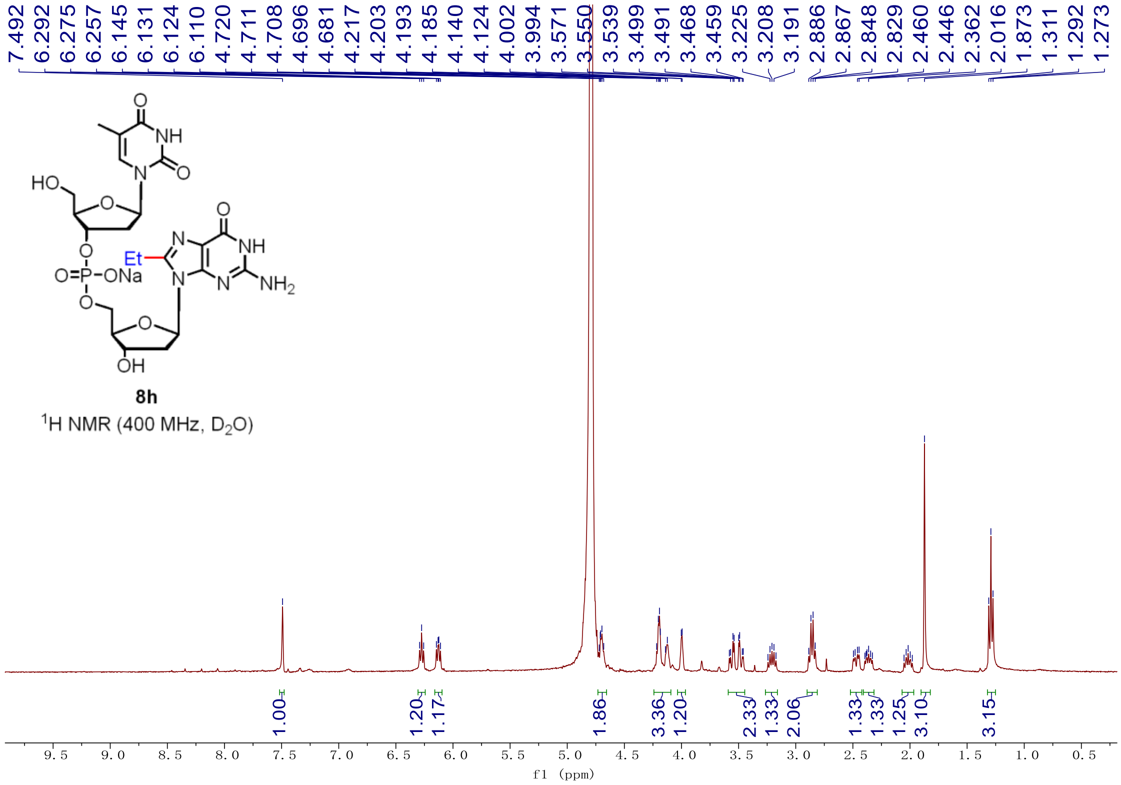

Supplementary Figure 260. ^{1}H NMR spectra of compound **8h**

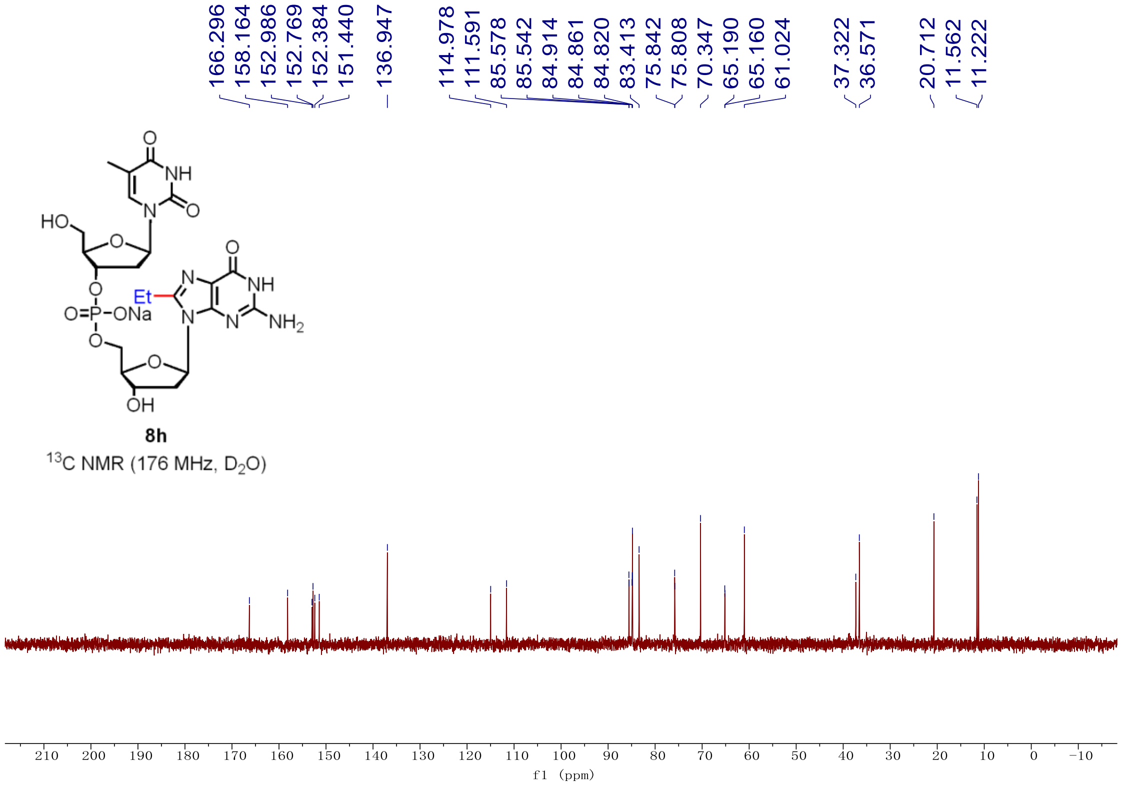

Supplementary Figure 261. ^{13}C NMR spectra of compound **8h**

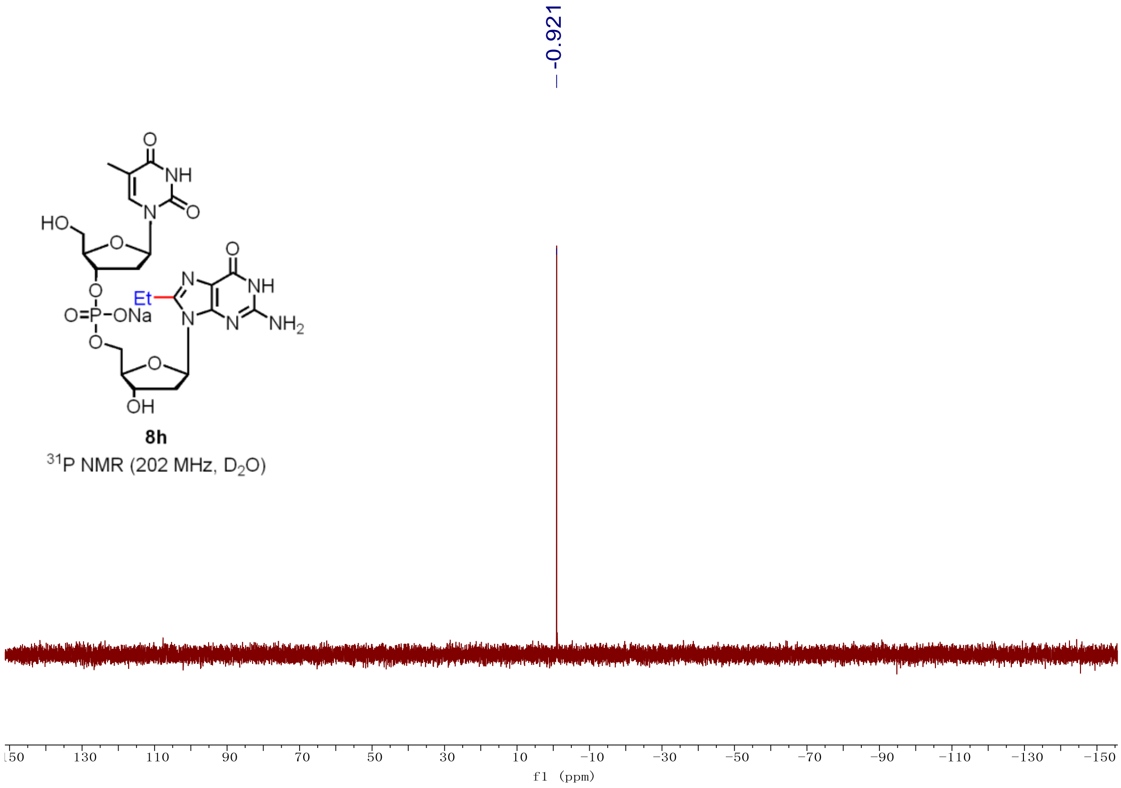

Supplementary Figure 262. ^{31}P NMR spectra of compound **8h**

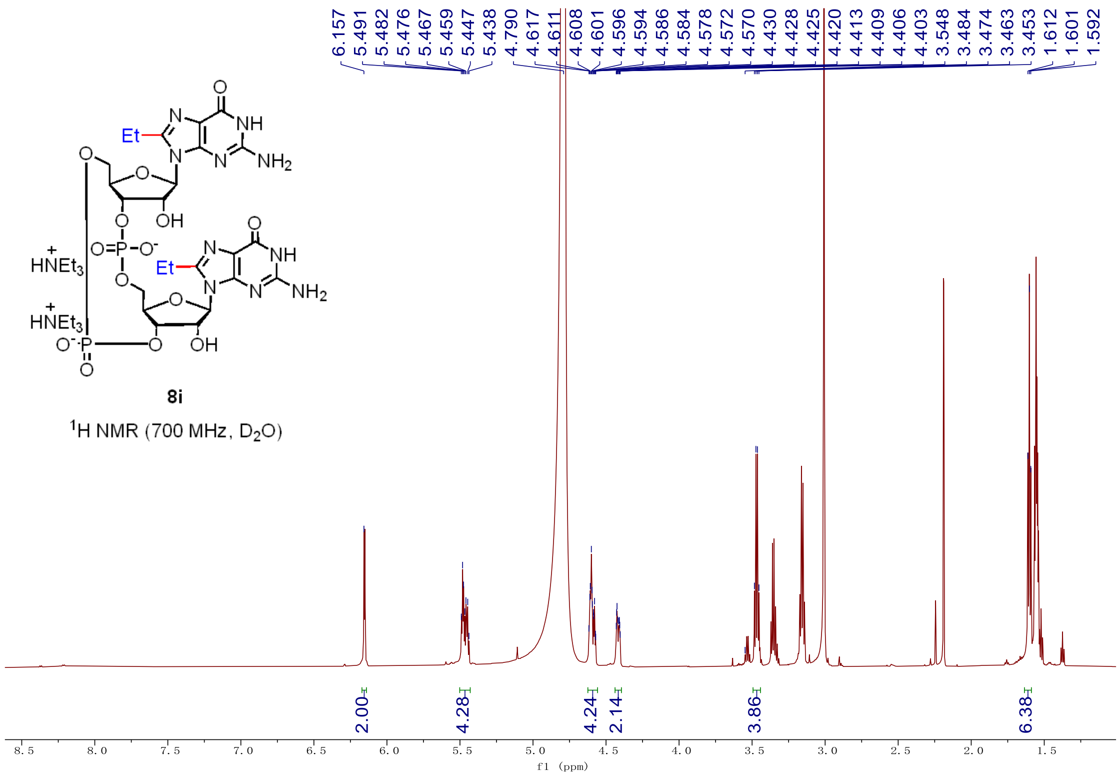

Supplementary Figure 263. ^{1}H NMR spectra of compound **8i**

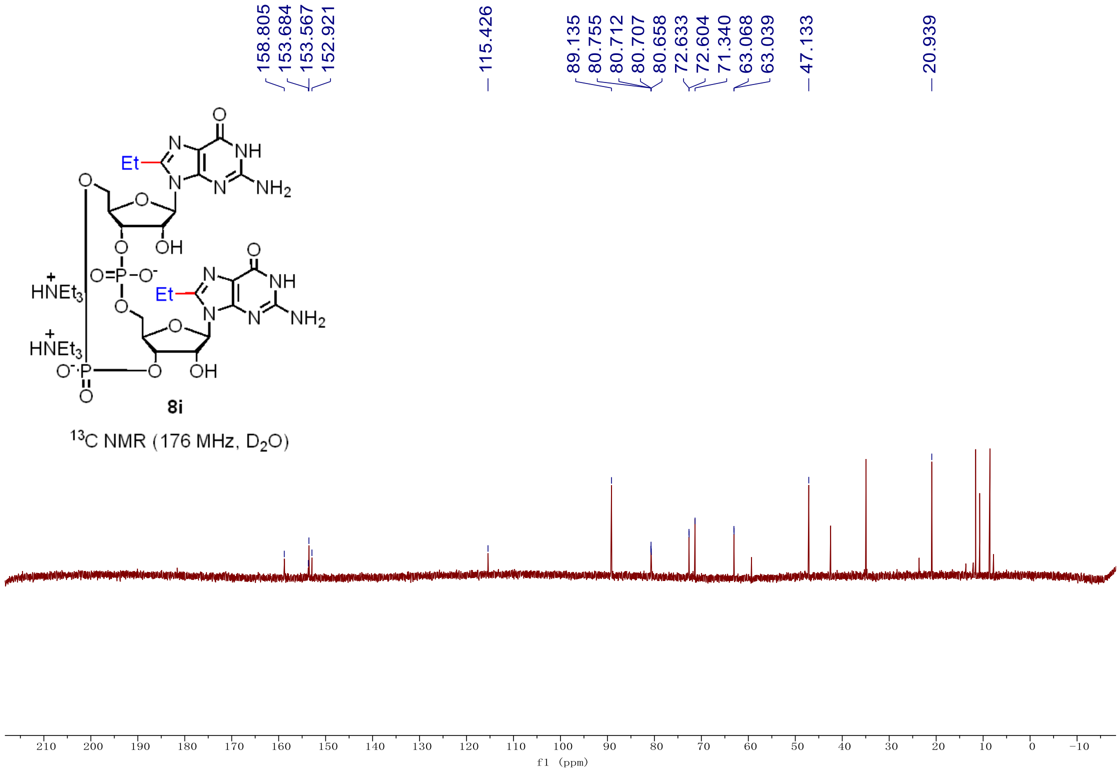

Supplementary Figure 264. ^{13}C NMR spectra of compound **8i**

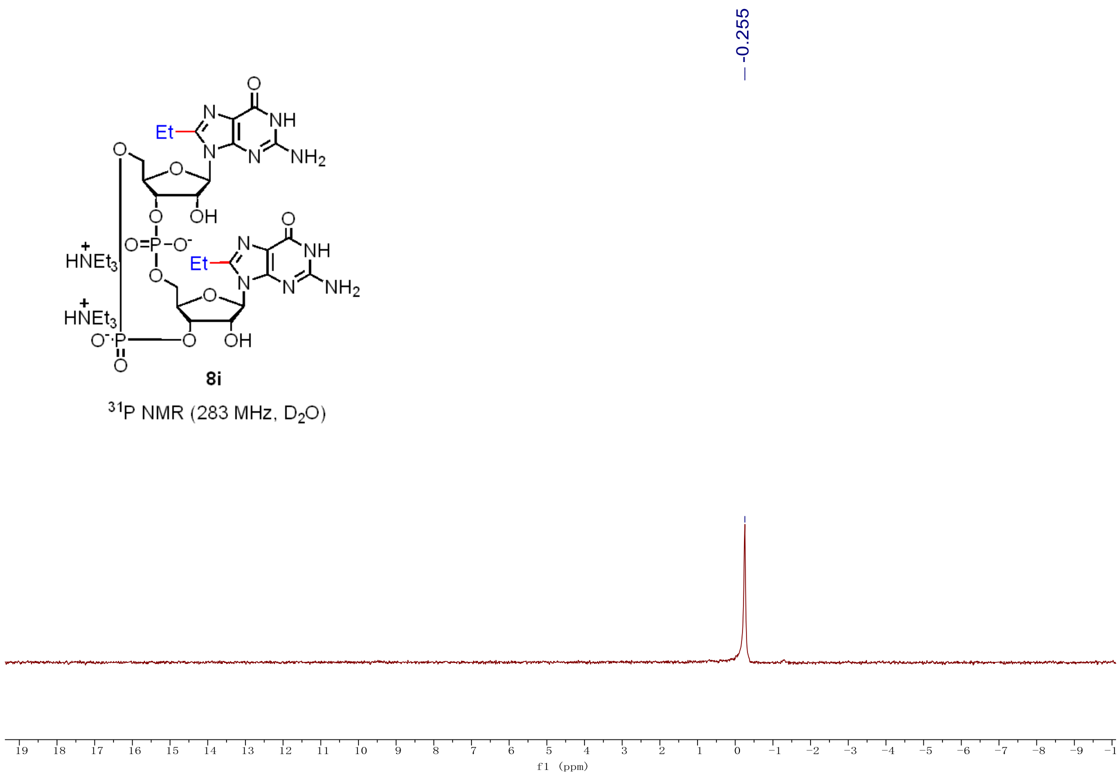

Supplementary Figure 265. ^{31}P NMR spectra of compound **8i**

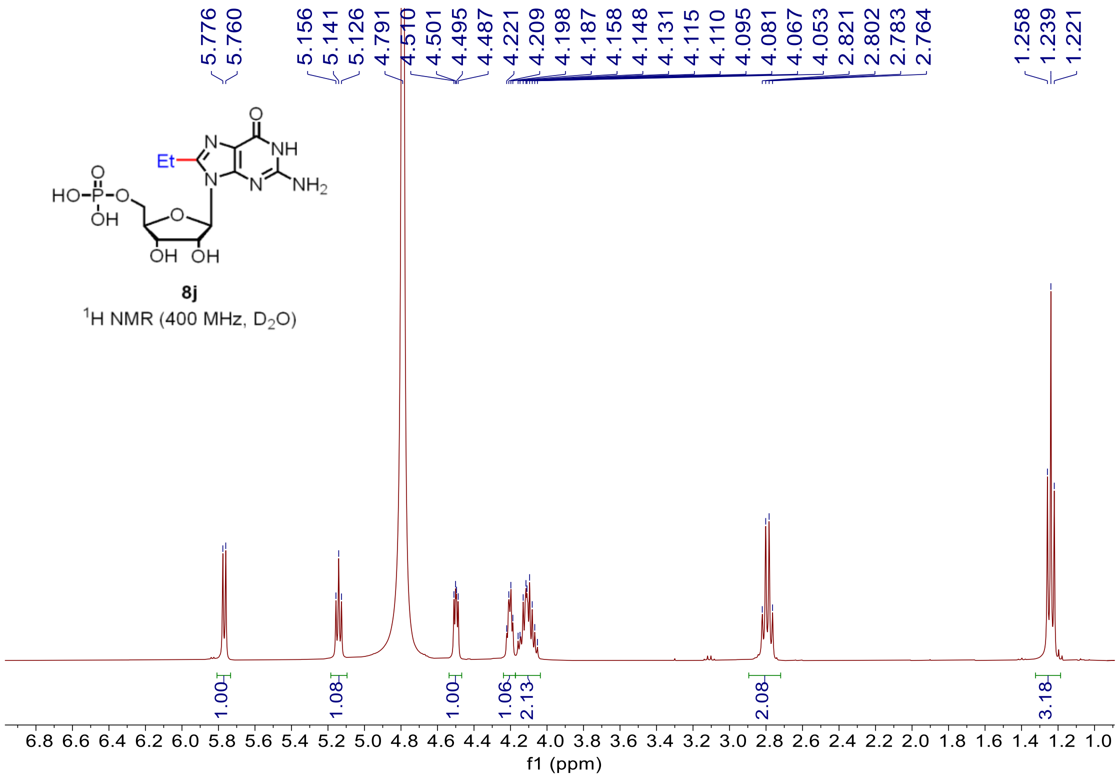

Supplementary Figure 266. ^{1}H NMR spectra of compound **8j**

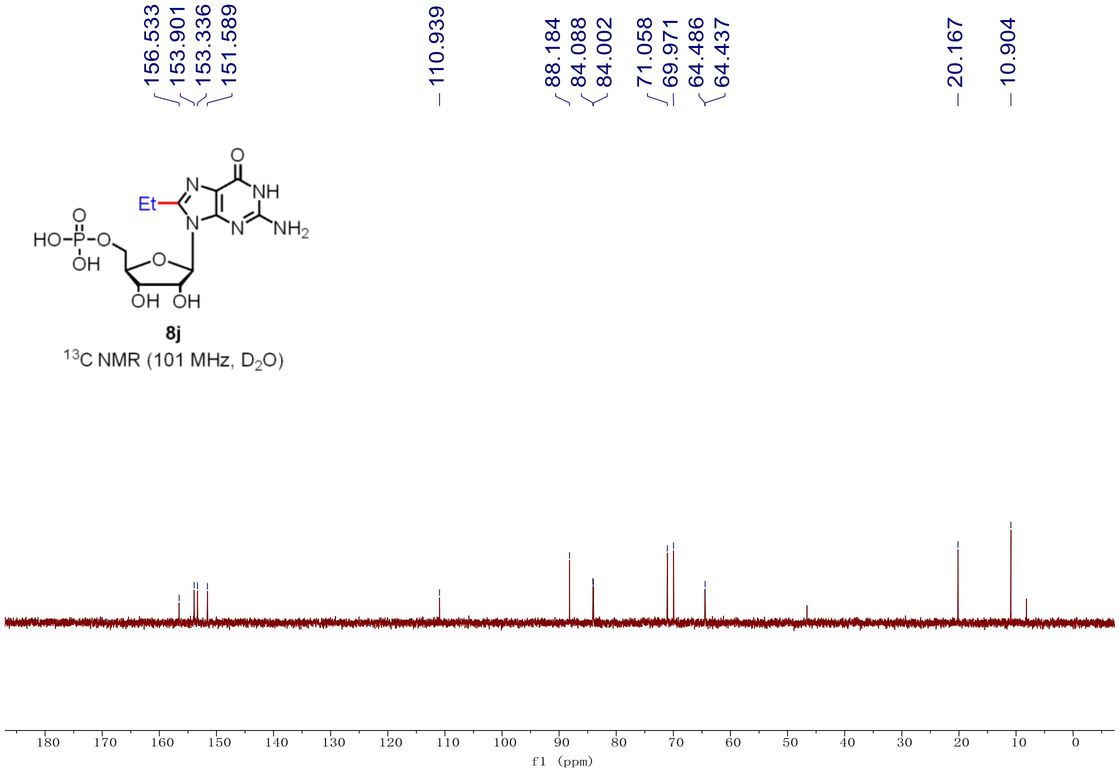

Supplementary Figure 267. ^{13}C NMR spectra of compound **8j**

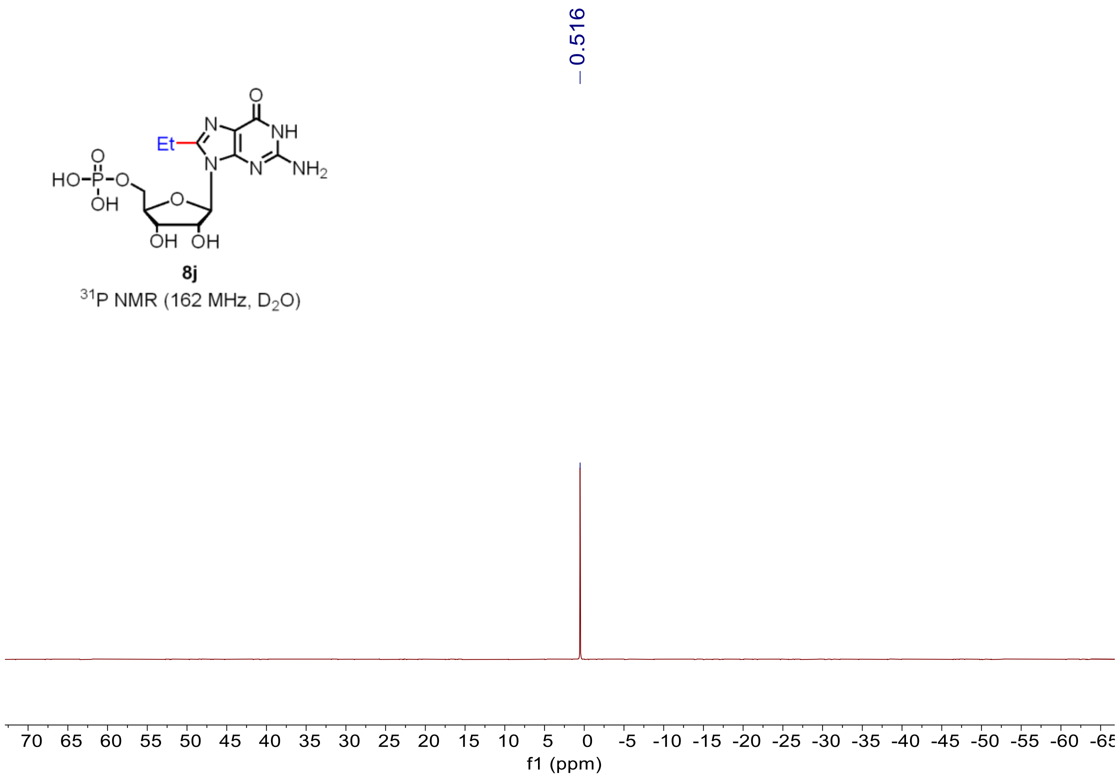

Supplementary Figure 268. ^{31}P NMR spectra of compound **8j**

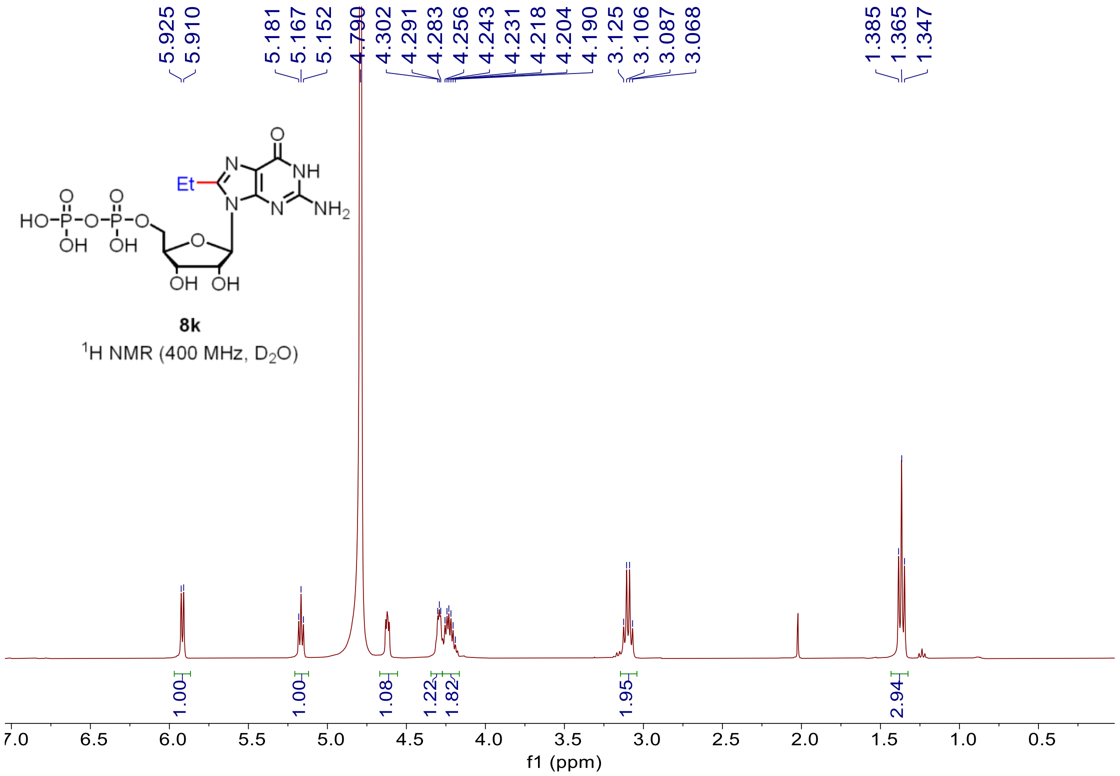

Supplementary Figure 269. ^{1}H NMR spectra of compound **8k**

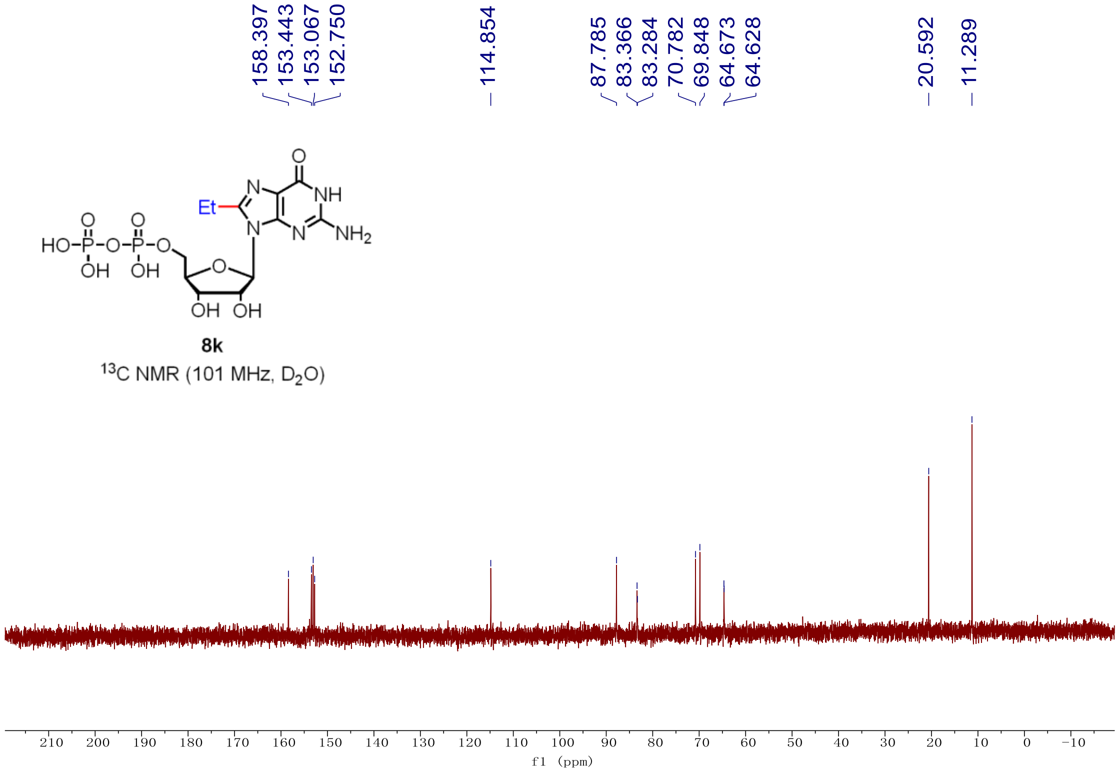

Supplementary Figure 270. ^{13}C NMR spectra of compound **8k**

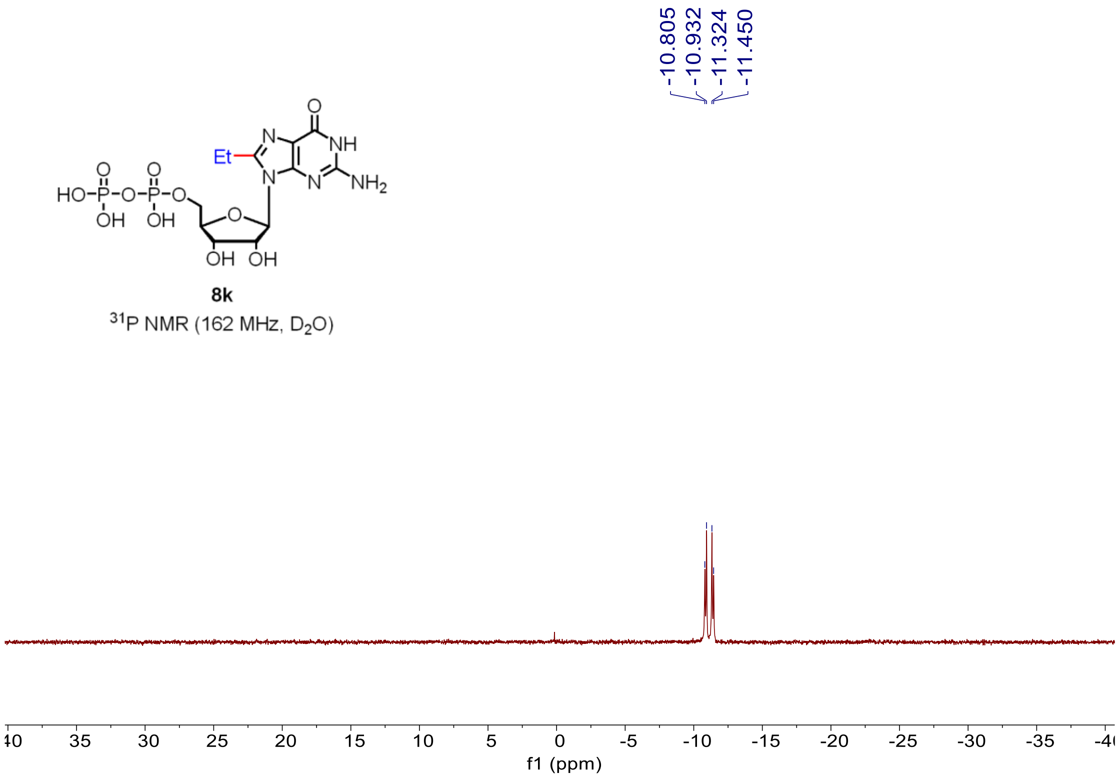

Supplementary Figure 271. ^{31}P NMR spectra of compound **8k**

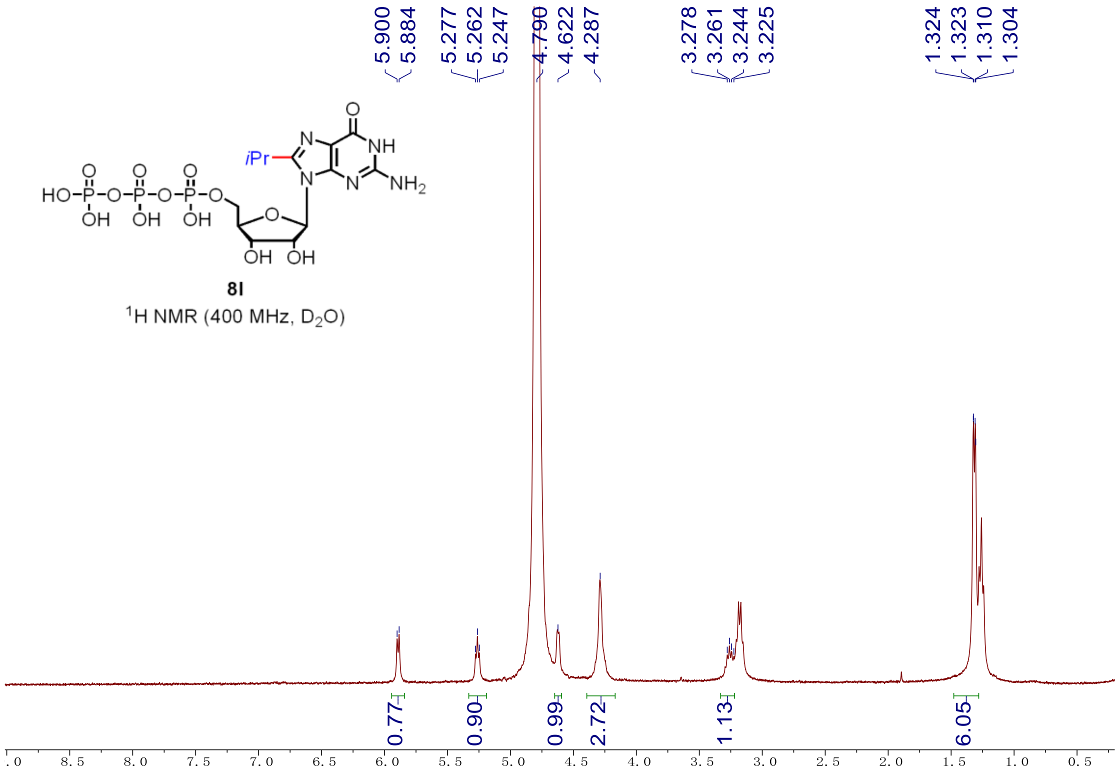

Supplementary Figure 272. ^{1}H NMR spectra of compound **8l**

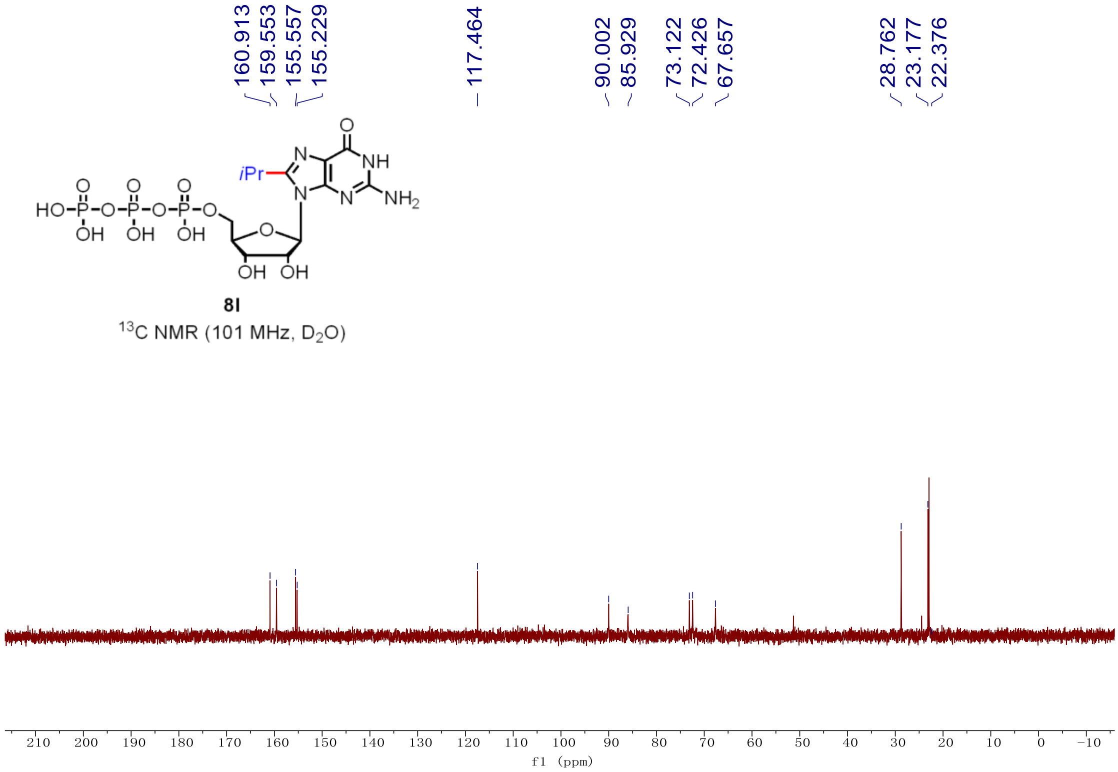

Supplementary Figure 273. ^{13}C NMR spectra of compound **8l**

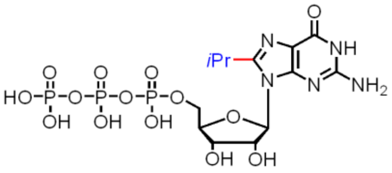

8l

^{31}P NMR (162 MHz, D_2O)

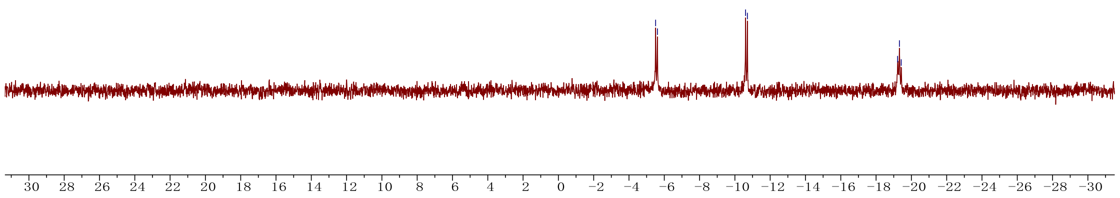

Supplementary Figure 274. ^{31}P NMR spectra of compound **8l**

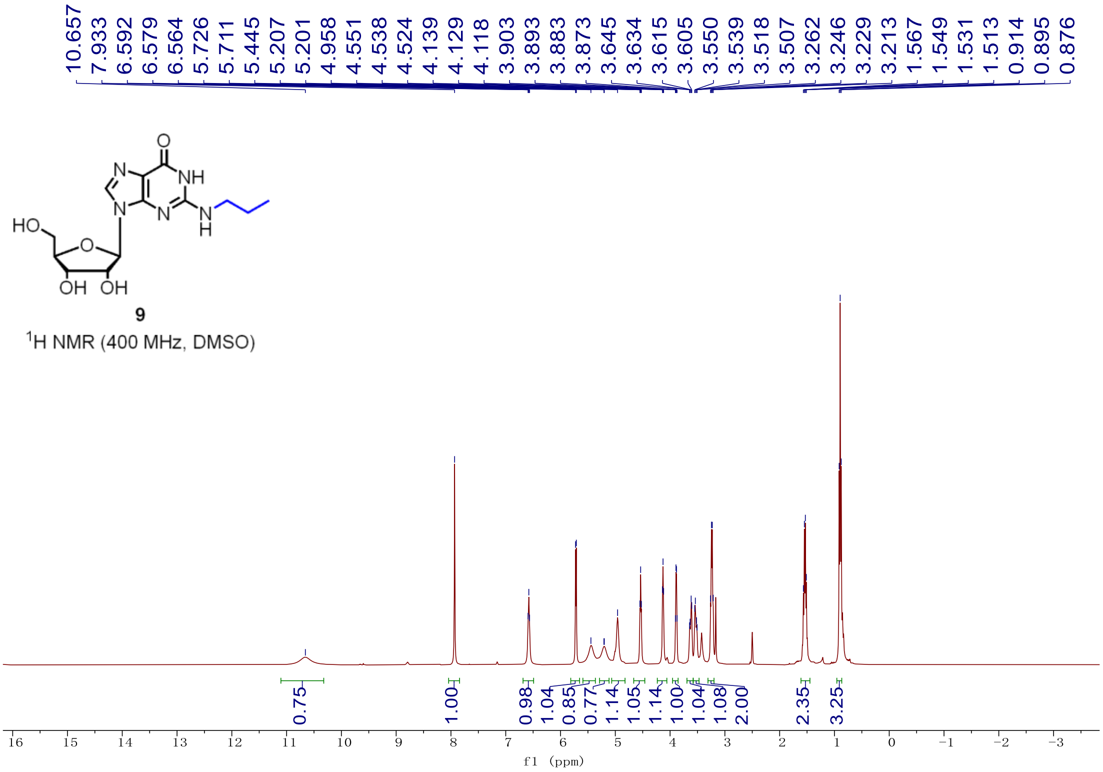

Supplementary Figure 275. ^{1}H NMR spectra of compound **9**

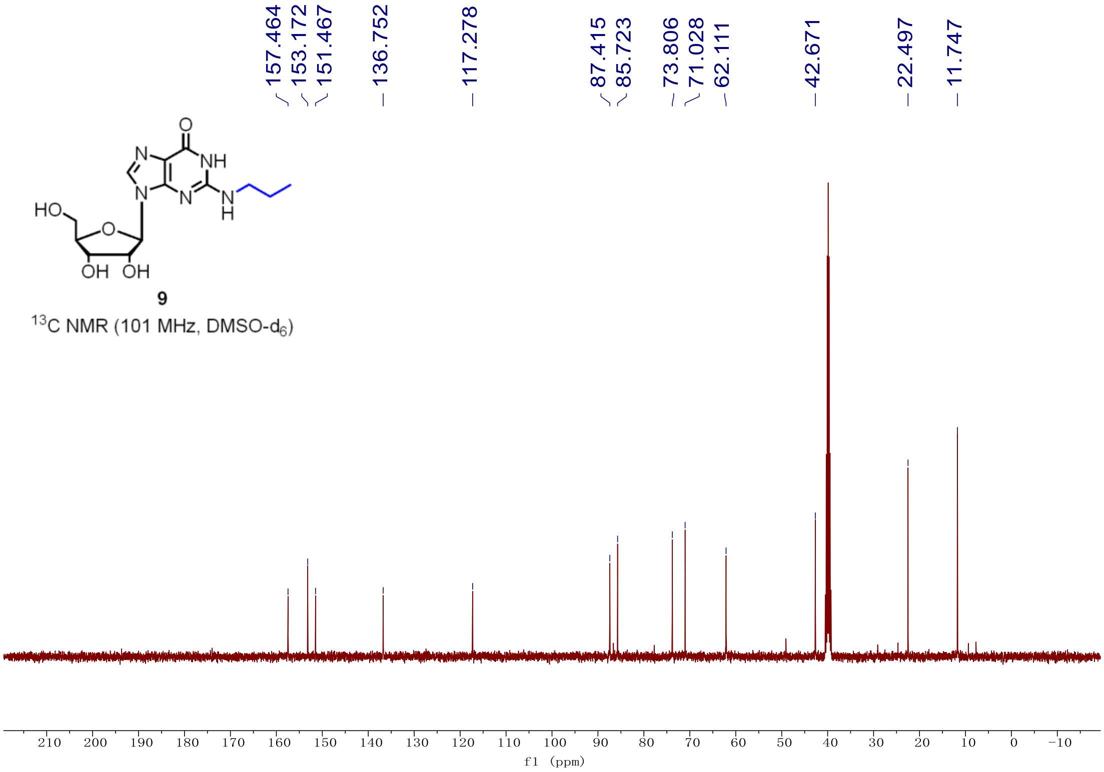

Supplementary Figure 276. ^{13}C NMR spectra of compound **9**

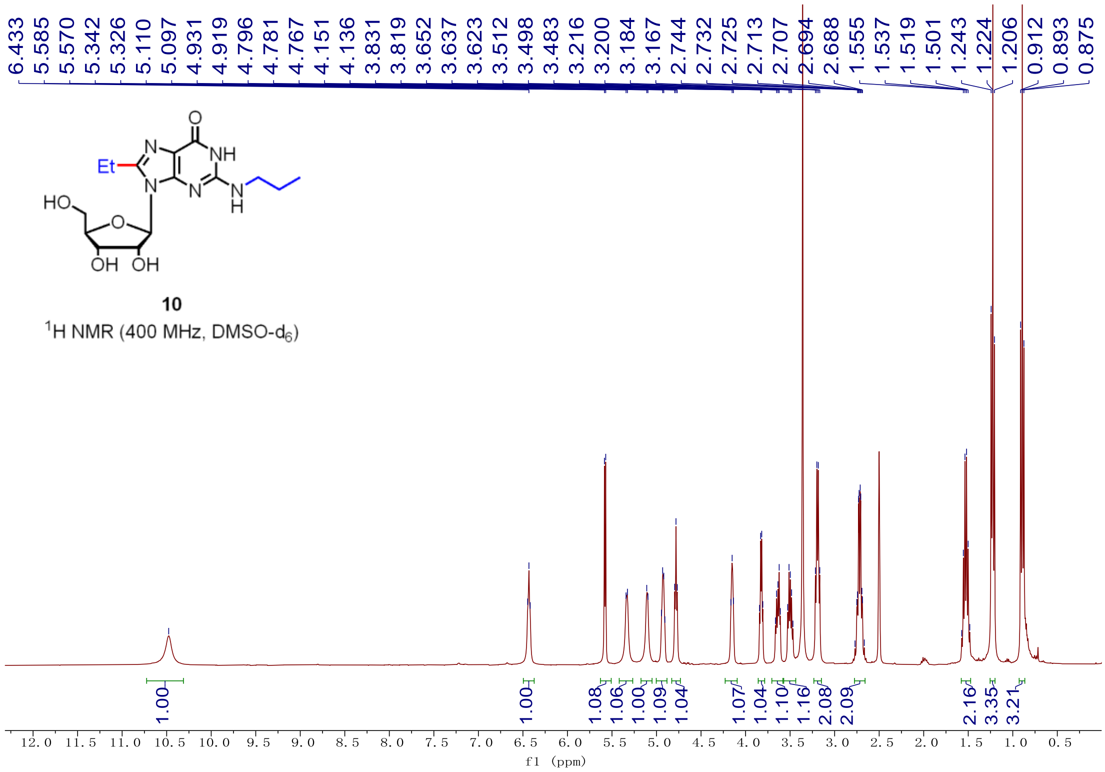

Supplementary Figure 277. ^{1}H NMR spectra of compound **10**

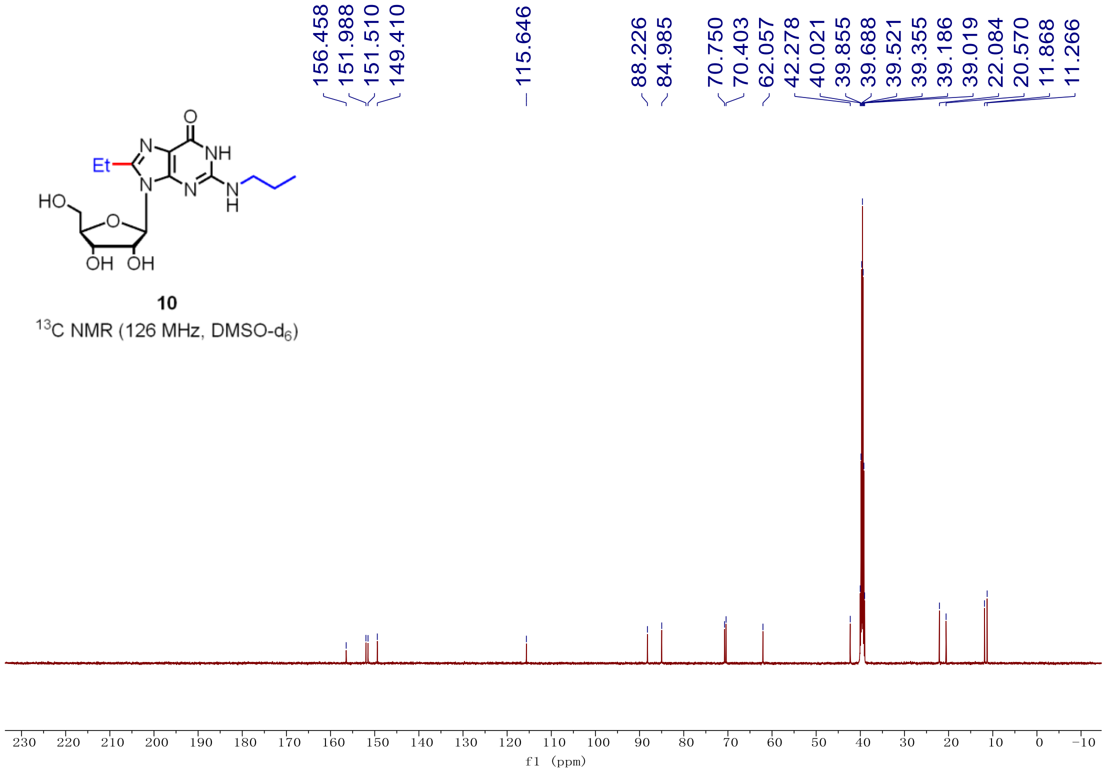

Supplementary Figure 278. ^{13}C NMR spectra of compound **10**

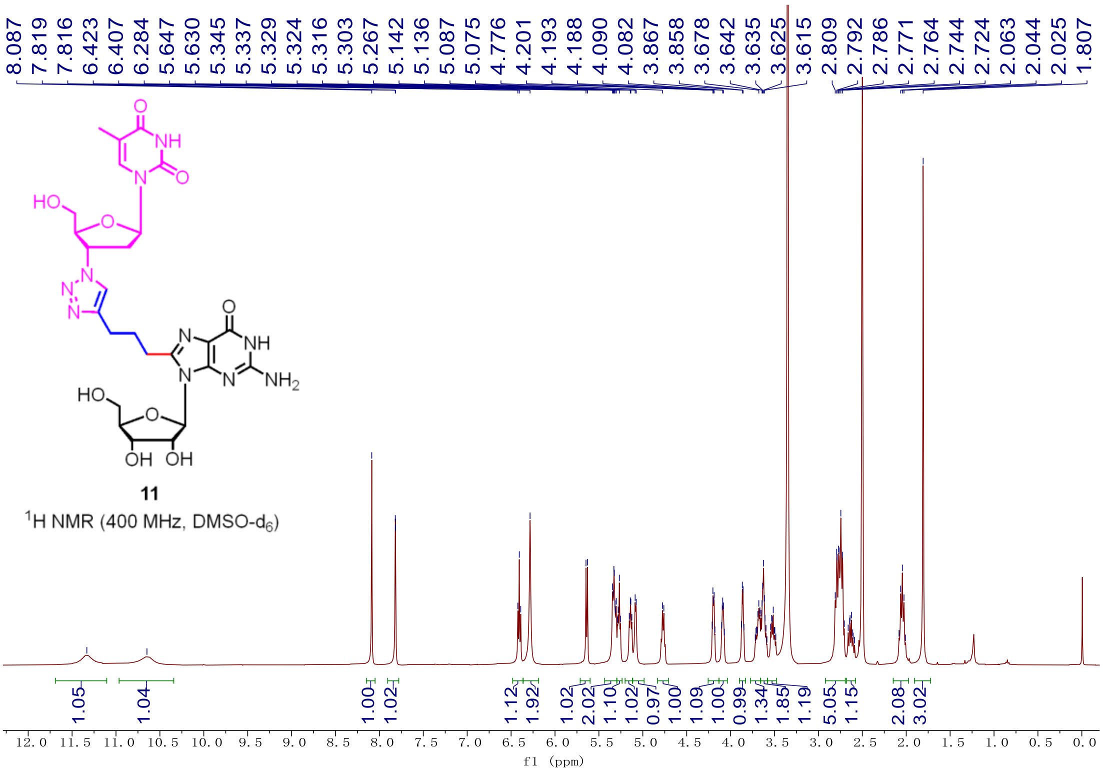

Supplementary Figure 279. ^{1}H NMR spectra of compound **11**

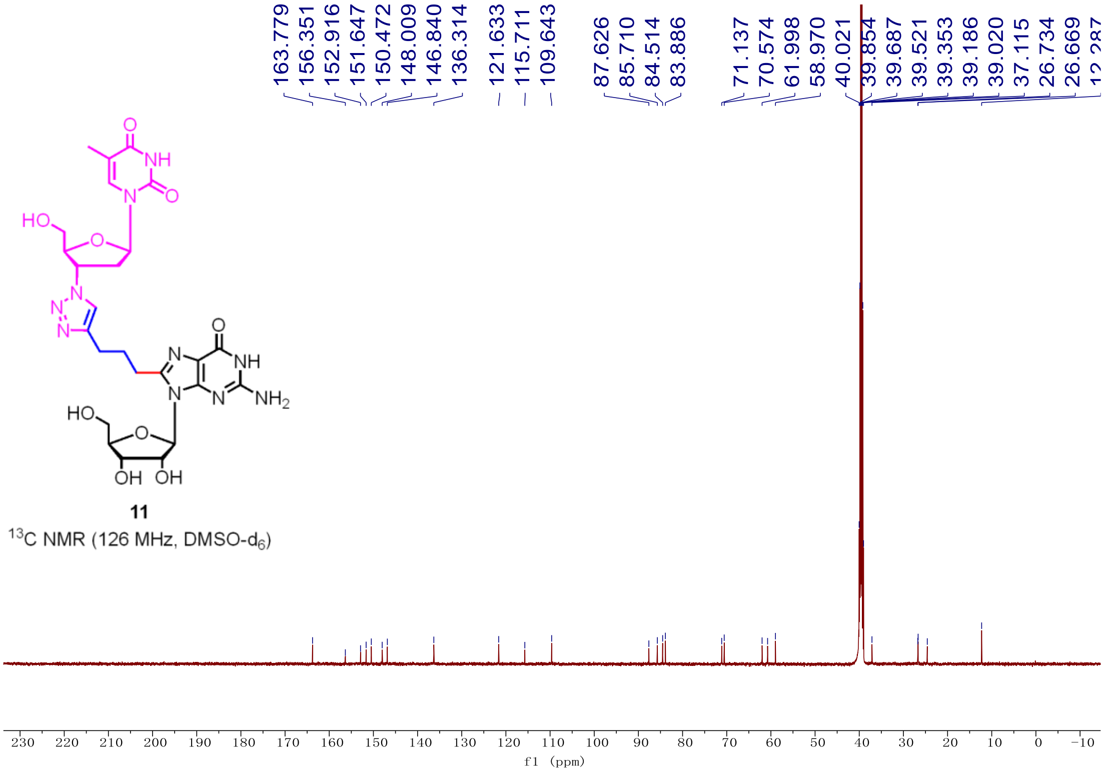

Supplementary Figure 280. ^{13}C NMR spectra of compound **11**

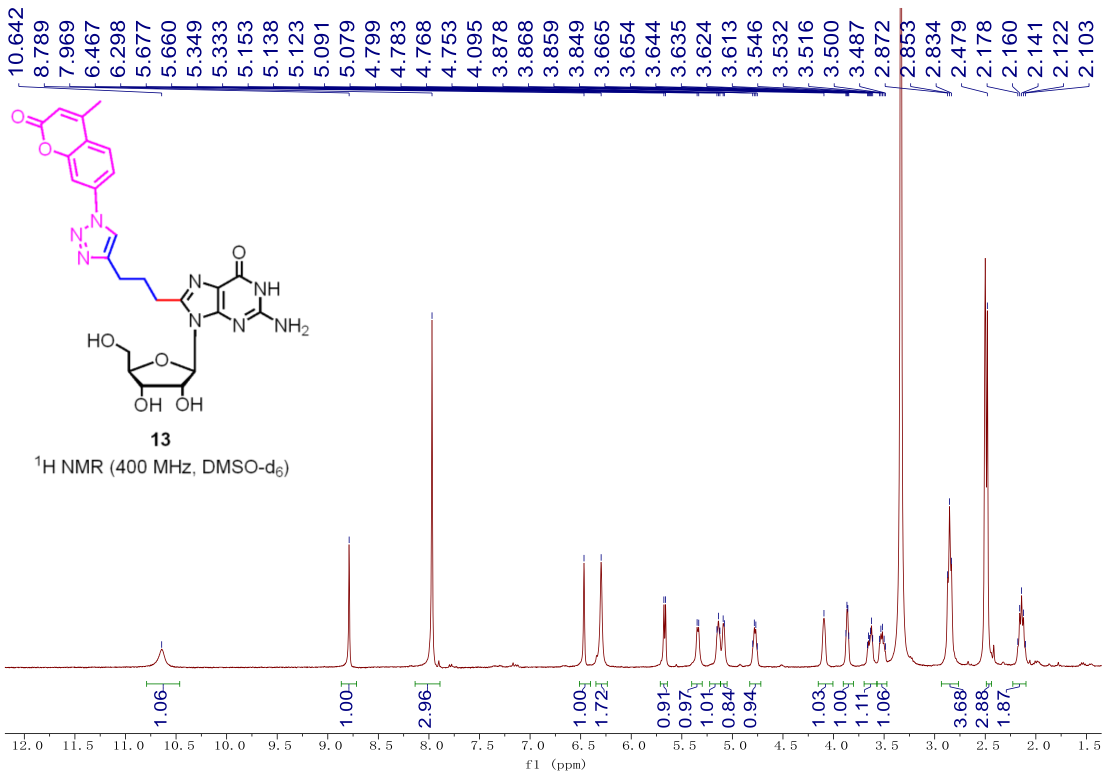

Supplementary Figure 281. ^{1}H NMR spectra of compound **13**

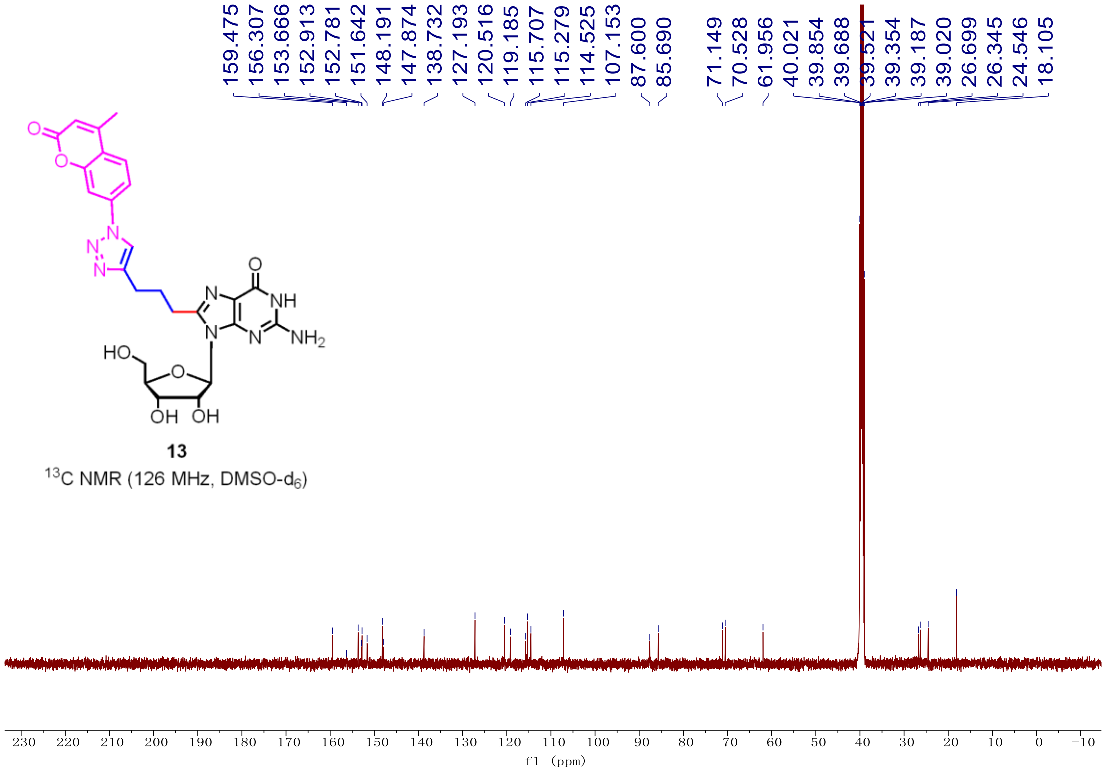

Supplementary Figure 282. ^{13}C NMR spectra of compound **13**

References

[1] Sinha, N. D. & Jung, K. E. Analysis and purification of synthetic nucleic acids using HPLC. *Curr Protoc Nucleic Acid Chem* **61**, (2015).

[2] Hirota, K., Kitade, Y., Kanbe, Y. & Maki, Y. Convenient method for the synthesis of C-alkylated purine nucleosides: palladium-catalyzed cross-coupling reaction of halogenopurine nucleosides with trialkylaluminums. *J. Org. Chem.* **57**, 5268–5270 (1992).

[3] Cahová, H., Pohl, R., Bednárová, L., Nováková, K., Cvačka, J., Hocek, M. Synthesis of 8-bromo-, 8-methyl- and 8-phenyl-dATP and their polymerase incorporation into DNA. *Org. Biomol. Chem.* **6**, 3657-3660 (2008).

[4] Maeda, M., Nushi, K. & Kawazoe, Y. Studies on chemical alterations of nucleic acids and their components—VII. *Tetrahedron* **30**, 2677–2682 (1974).

[5] Pless, R., Dudycz, L., Stolarski, R. & Shugar, D. Purine nucleosides and nucleotides unequivocally in the syn conformation: guanosine and 5'-GM P with 8-tert-butyl and 8-(α-Hydroxyisopropyl) Substituents. *Z. Naturforsch., C, J. Biosci.* **33**, 902–907 (1978).

[6] Xia, R., Niu, H.-Y., Qu, G.-R. & Guo, H.-M. CuI controlled C–C and C–N bond formation of heteroaromatics through C(sp^3)–H activation. *Org. Lett.* **14**, 5546–5549 (2012).

[7] Revil-Baudard, V. L., Vors, J.-P. & Zard, S. Z. Xanthate-mediated incorporation of quaternary centers into heteroarenes. *Org. Lett.* **20**, 3531–3535 (2018).

[8] Chrominski, M., Baranowski, M. R., Chmielinski, S., Kowalska, J. & Jemielity, J. Synthesis of trifluoromethylated purine ribonucleotides and their evaluation as ^{19}F NMR Probes. *J. Org. Chem.* **85**, 3440–3453 (2020).

[9] Bergstrom, D. E. & Ogawa, M. K. C-5 substituted pyrimidine nucleosides. 2. Synthesis *via* olefin coupling to organopalladium intermediates derived from uridine and 2'-deoxyuridine. *J. Am. Chem. Soc.* **100**, 8106–8112 (1978).

[10] Hassan, M. E. Photochemical synthesis of 5-alkylpyrimidine nucleosides. *Rec. Trav. Chim. Pays-Bas.* **105**, 30–32 (1986).

[11] Larock, R. C., Wang, Y., Dong, X. & Yao, T. Synthesis of C-5 substituted nucleosides *via* palladium-catalyzed coupling of dienes and amines. *Tetrahedron* **61**, 11427–11439 (2005).

[12] Wang, D.-C., Xia, R., Xie, M.-S., Qu, G.-R. & Guo, H.-M. Synthesis of cycloalkyl substituted purine nucleosides *via* a metal-free radical route. *Org. Biomol. Chem.* **14**, 4189–4193 (2016).

[13] Purohit, V. & Basu, A. K. Synthesis and characterization of oligodeoxynucleotides containing the major DNA adducts formed by 1,6- and 1,8-Dinitropyrene. *Org. Lett.* **2**, 1871–1874 (2000).

[14] Sakurai, M., Yano, T., Kawabata, H., Ueda, H., & Suzuki, T. Inosine cyanoethylation identifies A-to-I RNA editing sites in the human transcriptome. *Nature chemical biology*, **6(10)**, 733–740

(2010).
[15] Omumi, A., Beach, D. G., Baker, M., Gabryelski, W. & Manderville, R. A. Postsynthetic Guanine arylation of DNA by Suzuki−Miyaura cross-coupling. *J. Am. Chem. Soc.* **133**, 42–50 (2010).
[16] Geigle, S. N., Wyss, L. A., Sturla, S. J. & Gillingham, D. G. Copper carbenes alkylate guanine chemoselectively through a substrate directed reaction. *Chem. Sci.* **8**, 499–506 (2017).
[17] Xie, L. *et al.* Identification of flavin mononucleotide as a cell-active artificial N6-methyladenosine RNA demethylase. *Angew. Chem, Int. Ed.* **58**, 5028–5032 (2019).
[18] Li, Y., Göhl, M., Ke, K., Vanderwal, C. D. & Spitale, R. C. Identification of adenosine-to-inosine RNA editing with acrylonitrile reagents. *Org. Lett.* **21**, 7948–7951 (2019).
[19] Nappi, M., Hofer, A., Balasubramanian, S. & Gaunt, M. J. Selective chemical functionalization at N6-methyladenosine residues in DNA enabled by visible-light-mediated photoredox catalysis. *J. Am. Chem. Soc.* **142**, 21484–21492 (2020).
[20] Bhoge, B. A., Mala, P., Kurian, J. S., Srinivasan, V. & Saraogi, I. Selective functionalization at N2-position of guanine in oligonucleotides *via* reductive amination. *Chem. Comm.* **56**, 13832–13835 (2020).
[21] Weng, X. *et al.* Keth-seq for transcriptome-wide RNA structure mapping. *Nat. Chem. Biol* **16**, 489–492 (2020).
[22] Ravi Kumara, G. S., Pandith, A. & Seo, Y. J. Direct and selective metal-free N^6-arylation of adenosine residues for simple fluorescence labeling of DNA and RNA. *Chem. Comm.* **57**, 5450–5453 (2021).
[23] Lee, Y.-H., Yu, E. & Park, C.-M. Programmable site-selective labeling of oligonucleotides based on carbene catalysis. *Nat. Commun.* **12**, 1681–1690 (2021).
[24] Sýkorová, V., Tichý, M. & Hocek, M. Polymerase Synthesis of DNA Containing Iodinated Pyrimidine or 7-Deazapurine Nucleobases and Their Post-synthetic Modifications through the Suzuki-Miyaura Cross-Coupling Reactions. *Chem. Bio. Chem.* **23**, 1-8 (2021).
[25] Xie, Y. *et al*. 6-iodopurine as a versatile building block for RNA purine architecture modifications. *Bioconjug. Chem.* **33**, 353–362 (2022).
[26] Presset, M., Fleury-Brégeot, N., Oehlrich, D., Rombouts, F. & Molander, G. A. Synthesis and minisci reactions of organotrifluoroborato building blocks. *J. Org. Chem.* **78**, 4615–4619 (2013).
[27] Atack, T. C. & Cook, S. P. Manganese-catalyzed borylation of unactivated Alkyl Chlorides. *J. Am. Chem. Soc.* **138**, 6139–6142 (2016).
[28] Go, S. Y. *et al.* A Unified synthetic strategy to introduce heteroatoms via electrochemical functionalization of alkyl organoboron reagents. *J. Am. Chem. Soc.* **144**, 9149–9160 (2022).
[29] Mun, S., Lee, J.-E. & Yun, J. Copper-Catalyzed β-Boration of α,β-Unsaturated Carbonyl Compounds: Rate Acceleration by Alcohol Additives. *Org. Lett.* **8**, 4887–4889 (2006).

[30] Wang, J. *et al.* Cu-catalyzed decarboxylative borylation. *ACS Catalysis* **8**, 9537–9542 (2018).

[31] Hu, D., Wang, L. & Li, P. Decarboxylative borylation of aliphatic esters under visible-light photoredox conditions. *Org. Lett.* **19**, 2770–2773 (2017).

[32] Aichhorn, S., Bigler, R., Myers, E. L. & Aggarwal, V. K. Enantiospecific synthesis of ortho-substituted benzylic boronic esters by a 1,2-metalate rearrangement/1,3-borotropic shift sequence. *J. Am. Chem. Soc.* **139**, 9519–9522 (2017).

[33] Friese, F. W. & Studer, A. Deoxygenative borylation of secondary and tertiary alcohols. *Angew. Chem, Int. Ed.* **58**, 9561–9564 (2019).

[34] Panferova, L. I. & Dilman, A. D. Light-mediated sulfur–boron exchange. *Org. Lett.* **23**, 3919–3922 (2021).

[35] von Watzdorf, J., Leitner, K. & Marx, A. Modified nucleotides for discrimination between cytosine and the epigenetic marker 5-methylcytosine. *Angew. Chem, Int. Ed.* **55**, 3229–3232 (2016).

[36] Gaffney, B. L., Veliath, E., Zhao, J. & Jones, R. A. One-flask syntheses of c-di-GMP and the [Rp,Rp] and [Rp,Sp] thiophosphate analogues. *Org. Lett.* **12**, 3269–3271 (2010).

[37] Wang, C., Hao, M., Qi, Q., Chen, Y. & Hartig, J. S. Chemical synthesis, purification, and characterization of 3′-5′-linked canonical cyclic dinucleotides (CDNs). *Meth. Enzymol.* **625**, 41–59 (2019).

[38] Featherston, A. L. *et al.* Catalytic asymmetric and stereodivergent oligonucleotide synthesis. *Science* **371**, 702–707 (2021).

[39] Hsu, L.-Y. & Yang, K.-T. Synthesis and nuclease stability of dinucleotides containing an *Anti*-conformationally constrained acyclic thymidine. *Nucleosides and Nucleotides* **18**, 2031–2042 (1999).

[40] Jian, Y. *et al.* Indications of 5′ to 3′ interbase electron transfer as the first step of pyrimidine dimer formation probed by a dinucleotide analog. *Chem. Eur. J.* **23**, 7526–7537 (2017).

[41] Buchko, G. W. & Cadet, J. Identification of the α and β anomers of 1-(2-deoxy-d-erythro-pentofuranosyl)-oxaluric acid at the site of riboflavin-mediated photooxidation of guanine in 2′-deoxyguanosine and thymidylyl-(3′-5′)-2′-deoxyguanosine. *Photochem. Photobiol.* **82**, 191-199 (2006).

[42] Higashi, F., Hoshio, A. & Kiyoshige, J. Preparation of aromatic polyesters by the direct polycondensation reaction with diphenyl chlorophosphate in pyridine. *J. Appl. Polym. Sci.* **21**, 3241–3247 (1983).

[43] Higashi, F., Komatsu, M. & Takizawa, J. Preparation of the thermotropic copoly(amide ester) ofp-aminobenzoic acid ANDM-hydroxybenzoic acid with diphenyl chlorophosphate/pyridine. *J. Polym. Sci., Part A: Polym. Chem.* **40**, 1775–1780 (2002).

[44] Stawinski, J., Stromberg, R., Thelin, M. & Westman, E. Reactions of nucleoside hydrogenphosphonates with diphenyl chlorophosphate and sterically hindered aromatic acyl

chlorides. *Nucleosides Nucleotides Nucleic Acids* **7**, 601–604 (1988).
[45] Revil-Baudard, V. L., Vors, J.-P. & Zard, S. Z. Xanthate-mediated incorporation of quaternary centers into heteroarenes. *Org. Lett.* **20**, 3531–3535 (2018).
[46] Lyu, X.-L., Huang, S.-S., Song, H.-J., Liu, Y.-X. & Wang, Q.-M. Visible-light-induced copper-catalyzed decarboxylative coupling of redox-active esters with *N*-heteroarenes. *Org. Lett.* **21**, 5728–5732 (2019).
[47] Matsui, J. K., Primer, D. N. & Molander, G. A. Metal-free C–H alkylation of heteroarenes with alkyltrifluoroborates: a general protocol for 1°, 2°and 3°alkylation. *Chem. Sci.* **8**, 3512–3522 (2017).
[48] Josephson, B. *et al.* Light-driven post-translational installation of reactive protein side chains. *Nature* **585**, 530–537 (2020).
[49] Andersen, A., Chen, Y. & Birkedal, H. Bioinspired metal–polyphenol materials: self-healing and beyond. *Biomimetics* **4**, 30-49 (2019).
[50] Hinkes, S. P. A. & Klein, C. D. P. Virtues of volatility: a facile transesterification approach to boronic acids. *Org. Lett.* **21**, 3048–3052 (2019).
[51] Ezra, F. S., Lee, C.-H., Kondo, N. S., Danyluk, S. S. & Sarma, R. H. Conformational properties of purine-pyrimidine and pyrimidine-purine dinucleoside monophosphates. *Biochemistry* **16**, 1977–1987 (1977).
[52] Zhang, Q. & Wang, Y. Independent generation of the 5-Hydroxy-5,6-dihydrothymidin-6-yl radical and its reactivity in dinucleoside monophosphates. *J. Am. Chem. Soc.* **126**, 13287–13297 (2004).
[53] Läppchen, T. *et al.* Probing FtsZ and tubulin with C8-substituted GTP analogs reveals differences in their nucleotide binding sites. *Chem. Biol.* **15**, 189–199 (2008).
[54] Van Poecke, S. *et al.* 3′-[4-Aryl-(1,2,3-triazol-1-yl)]-3′-deoxythymidine analogues as potent and selective inhibitors of human mitochondrial thymidine kinase. *J. Med. Chem.* **53**, 2902–2912 (2010).
[55] Chan, T. R., Hilgraf, R., Sharpless, K. B. & Fokin, V. V. Polytriazoles as copper(I)-stabilizing ligands in catalysis. *Org. Lett.* **6**, 2853–2855 (2004).
